# Supplementary material for: Exposure to high-altitude hypobaric hypoxic environment induces low-frequency hearing loss in C57BL/6J mice: Mediated by slowing down the postsynaptic electrical signal transmission speed in the cochlear-inferior colliculus auditory signaling pathway
Source: PLoS One. 2026 Mar 11;21(3):e0342321. doi: 10.1371/journal.pone.0342321 (PMC12978441; doi:10.1371/journal.pone.0342321)
Supplement: S1 File — (ZIP) [file pone.0342321.s001.zip › 2025-06-20-30d-3.pdf]

Exam report

Patient: 2025-06-20-30d-3**,** -
(-)**Date:** June 12, 2025

ABR: ABR 2 CLICK 1: Cz-M1

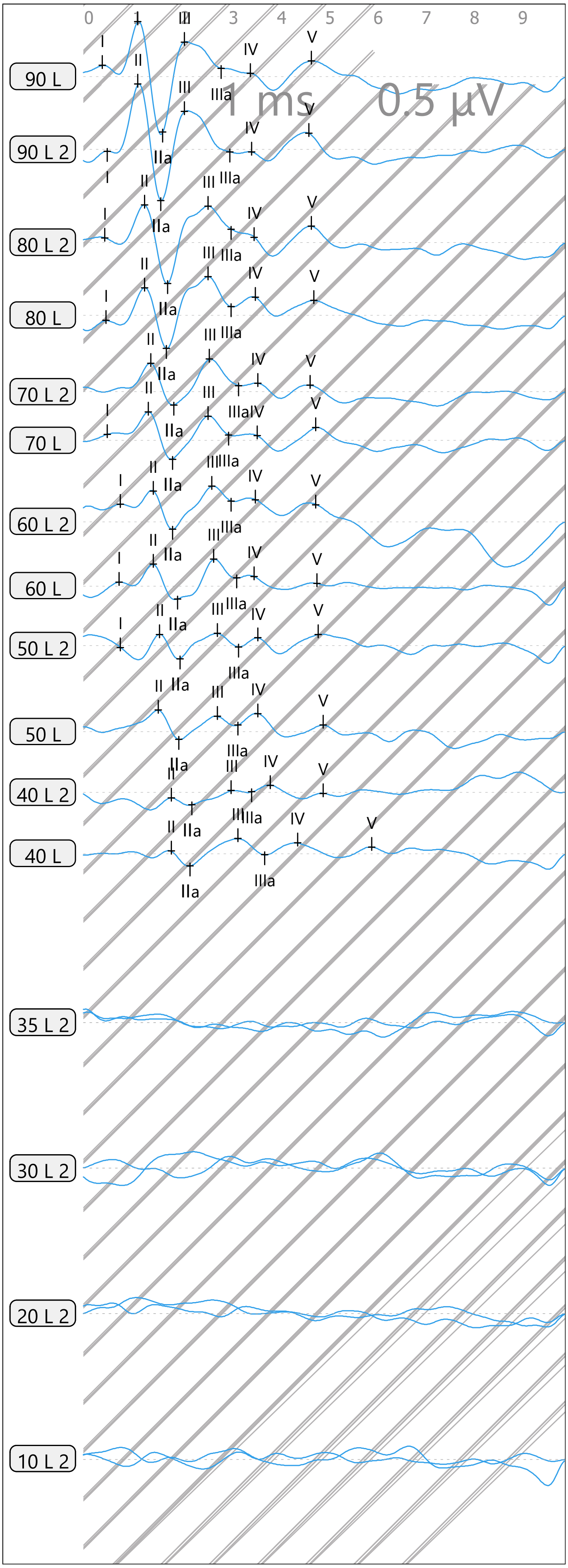
0
1
2
3
4
5
6
7
8
9
1 ms
0.5 µV
90 L
90 L 2
80 L 2
80 L
70 L 2
70 L
60 L 2
60 L
50 L 2
50 L
40 L 2
40 L
35 L 2
30 L 2
20 L 2
10 L 2

latency&& amplitude (left ear

N	I (ms)	II (ms)	III (ms)	IV (ms)	V (ms)
90 L	0.40	1.14	2.09	3.47	4.74
90 L 2	0.50	1.14	2.09	3.49	4.68
80 L	0.48	1.27	2.59	3.57	4.79
80 L 2	0.45	1.27	2.59	3.55	4.74
70 L	0.50	1.35	2.59	3.60	4.82
70 L 2		1.40	2.62	3.62	4.71
60 L	0.74	1.46	2.70	3.55	4.84
60 L 2	0.77	1.46	2.67	3.57	4.82
50 L		1.56	2.78	3.62	4.97
50 L 2	0.77	1.59	2.78	3.62	4.87
40 L		1.83	3.20	4.45	5.98
40 L 2		1.83	3.07	3.89	4.97

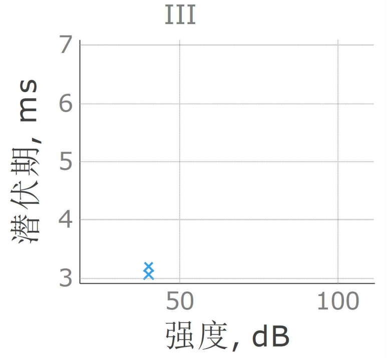

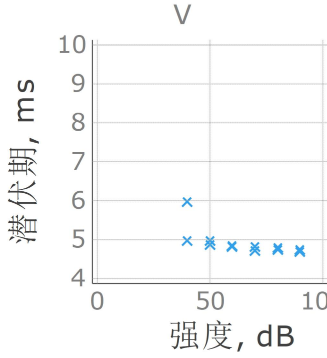

Trace parameters

N	Electr.	HPF, Hz	LPF, Hz	50 Hz	Rejection ±μV	Aver.	Reject.
90 L	Cz-M1	100	2000		10	1000	0
90 L 2	Cz-M1	100	2000		10	1000	0
80 L	Cz-M1	100	2000		10	1000	0
80 L 2	Cz-M1	100	2000		10	1000	0
70 L	Cz-M1	100	2000		10	1000	0
70 L 2	Cz-M1	100	2000		10	1000	0
60 L	Cz-M1	100	2000		10	1000	0
60 L 2	Cz-M1	100	2000		10	1000	0
50 L	Cz-M1	100	2000		10	1000	0
50 L 2	Cz-M1	100	2000		10	1000	0
40 L	Cz-M1	100	2000		10	1000	0
40 L 2	Cz-M1	100	2000		10	1000	0
35 L	Cz-M1	100	2000		10	1000	0
35 L 2	Cz-M1	100	2000		10	1000	0
30 L	Cz-M1	100	2000		10	1000	0
30 L 2	Cz-M1	100	2000		10	1000	0
20 L	Cz-M1	100	2000		10	1000	0
20 L 2	Cz-M1	100	2000		10	1000	0
10 L	Cz-M1	100	2000		10	1000	0
10 L 2	Cz-M1	100	2000		10	1000	0

ABR: ABR 2 tone burst 4000Hz 1
: Cz-M1

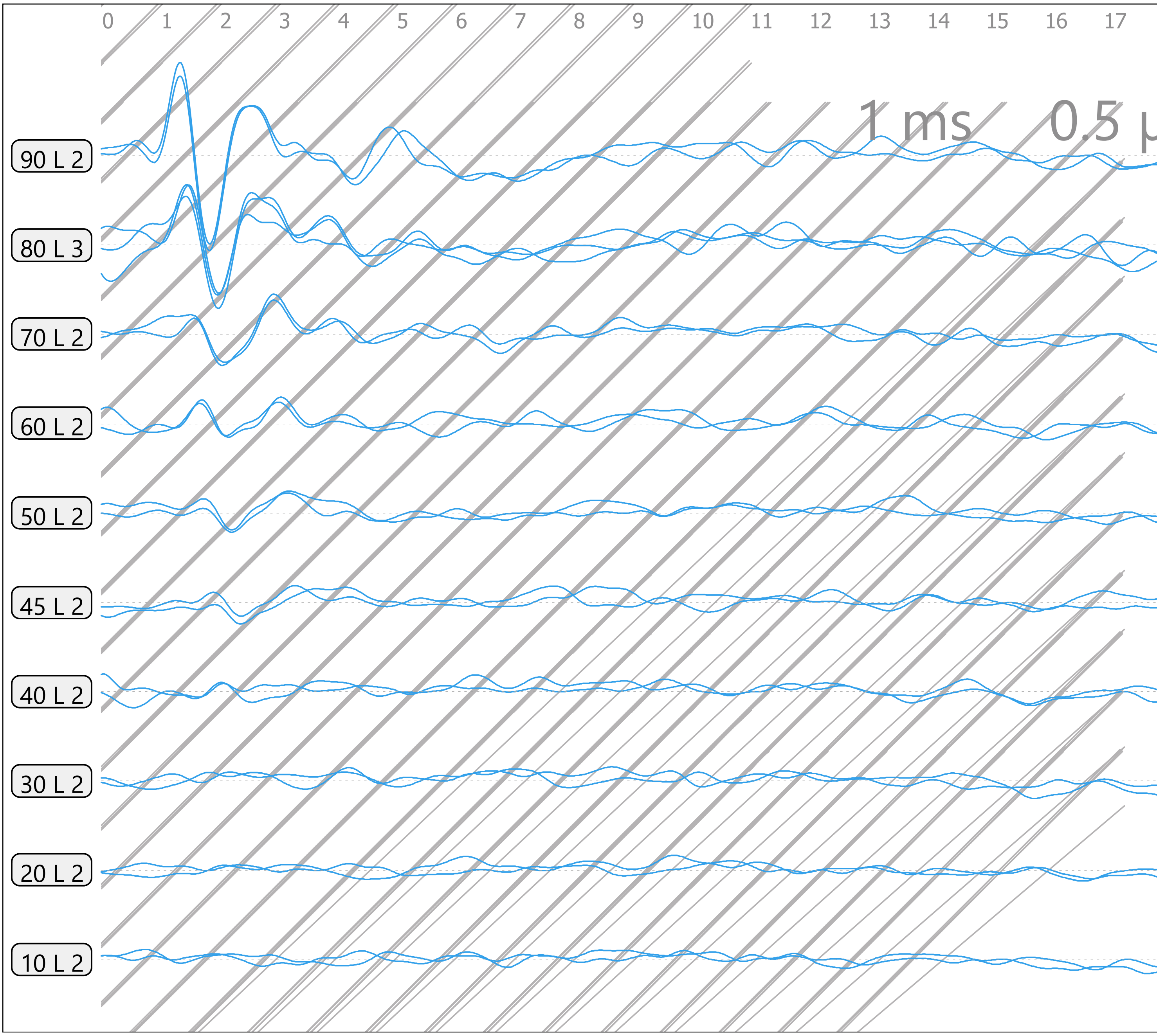

Trace parameters

N	Electr.	HPF, Hz	LPF, Hz	50 Hz	Rejection ±μV	Aver.	Reject.
90 L	Cz-M1	200	2000		10	1000	0
90 L 2	Cz-M1	200	2000		10	1000	0
80 L	Cz-M1	200	2000		10	1000	0
80 L 2	Cz-M1	200	2000		10	1000	0
80 L 3	Cz-M1	200	2000		10	1000	0
70 L	Cz-M1	200	2000		10	1000	0
70 L 2	Cz-M1	200	2000		10	1000	0
60 L	Cz-M1	200	2000		10	1000	0
60 L 2	Cz-M1	200	2000		10	1000	0
50 L	Cz-M1	200	2000		10	1000	0

50 L 2	Cz-M1	200	2000		10	1000	0
45 L	Cz-M1	200	2000		10	1000	0
45 L 2	Cz-M1	200	2000		10	1000	0
40 L	Cz-M1	200	2000		10	1000	0
40 L 2	Cz-M1	200	2000		10	1000	0
30 L	Cz-M1	200	2000		10	1000	0
30 L 2	Cz-M1	200	2000		10	1000	0
20 L	Cz-M1	200	2000		10	1000	0
20 L 2	Cz-M1	200	2000		10	1000	0
10 L	Cz-M1	200	2000		10	1000	0
10 L 2	Cz-M1	200	2000		10	1000	0

ABR: ABR 2 tone burst 8000Hz 1
: Cz-M1

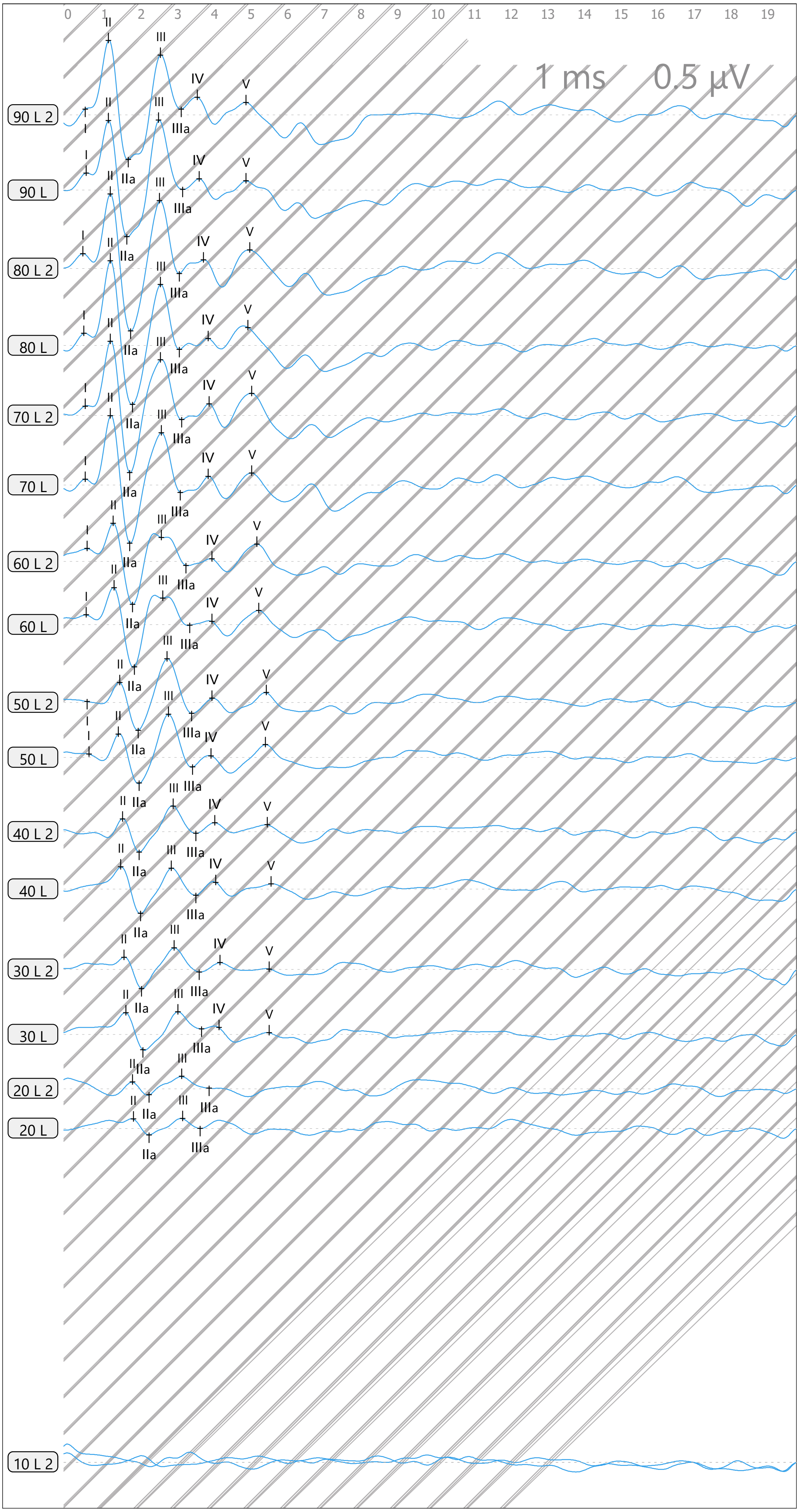
0 1 2 3 4 5 6 7 8 9 10 11 12 13 14 15 16 17 18 19
1 ms
0.5 µV
90 L 2
90 L
80 L 2
80 L
70 L 2
70 L
60 L 2
60 L
50 L 2
50 L
40 L 2
40 L
30 L 2
30 L
20 L 2
20 L
10 L 2
I
II
IIa
III
IIIa
IV
V

latency&& amplitude (left ear

N	I (ms)	II (ms)	III (ms)	IV (ms)	V (ms)
90 L	0.61	1.22	2.59	3.70	4.97
90 L 2	0.58	1.22	2.65	3.65	4.97
80 L	0.56	1.27	2.65	3.94	5.03
80 L 2	0.53	1.27	2.62	3.81	5.08
70 L	0.58	1.27	2.67	3.94	5.13
70 L 2	0.58	1.27	2.65	3.97	5.13
60 L	0.61	1.38	2.70	4.05	5.32
60 L 2	0.64	1.35	2.67	4.05	5.27
50 L	0.69	1.48	2.86	4.02	5.50
50 L 2	0.64	1.53	2.83	4.05	5.53
40 L		1.56	2.94	4.15	5.66
40 L 2		1.61	2.99	4.13	5.56
30 L		1.69	3.12	4.23	5.61
30 L 2		1.64	3.02	4.26	5.61
20 L		1.91	3.25		
20 L 2		1.88	3.23		

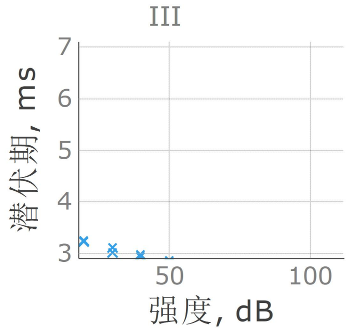

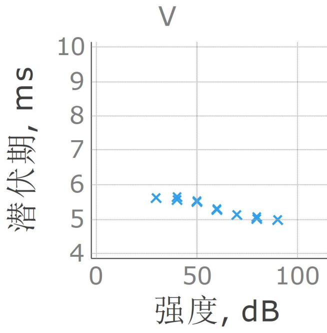

Trace parameters

N	Electr.	HPF, Hz	LPF, Hz	50 Hz	Rejection ±μV	Aver.	Reject.
90 L	Cz-M1	200	2000		10	1000	0
90 L 2	Cz-M1	200	2000		10	1000	0
80 L	Cz-M1	200	2000		10	1000	0
80 L 2	Cz-M1	200	2000		10	1000	0
70 L	Cz-M1	200	2000		10	1000	0
70 L 2	Cz-M1	200	2000		10	1000	0
60 L	Cz-M1	200	2000		10	1000	0
60 L 2	Cz-M1	200	2000		10	1000	0
50 L	Cz-M1	200	2000		10	1000	0
50 L 2	Cz-M1	200	2000		10	1000	0
40 L	Cz-M1	200	2000		10	1000	0
40 L 2	Cz-M1	200	2000		10	1000	0
30 L	Cz-M1	200	2000		10	1000	0
30 L 2	Cz-M1	200	2000		10	1000	0

20 L	Cz-M1	200	2000		10	1002	0
20 L 2	Cz-M1	200	2000		10	1000	0
10 L	Cz-M1	200	2000		10	1000	0
10 L 2	Cz-M1	200	2000		10	1000	0

ABR: ABR 2 CLICK 2: Cz-M2

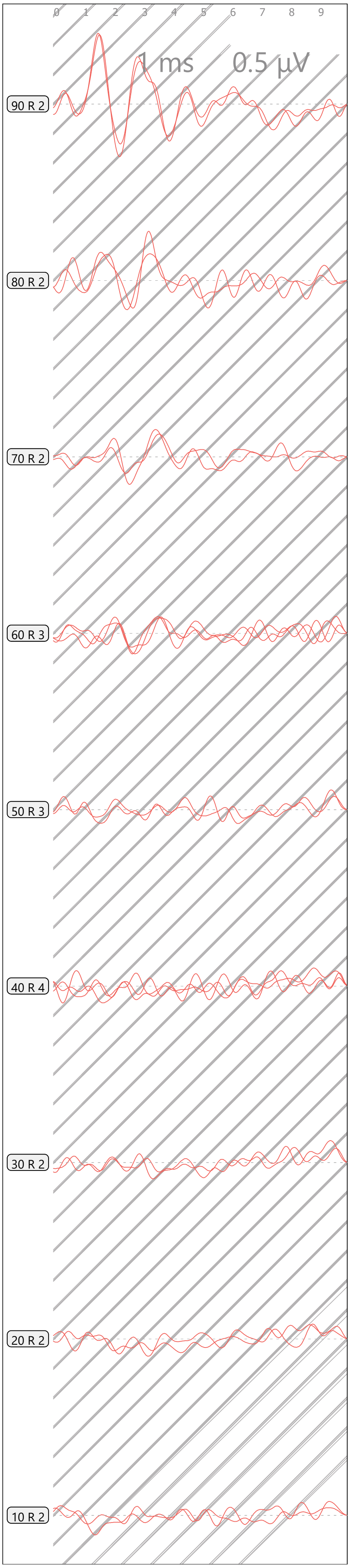
0 1 2 3 4 5 6 7 8 9
1 ms
0.5 µV
90 R 2
80 R 2
70 R 2
60 R 3
50 R 3
40 R 4
30 R 2
20 R 2
10 R 2

Trace parameters

N	Electr.	HPF, Hz	LPF, Hz	50 Hz	Rejection ±μV	Aver.	Reject
90 R	Cz-M2	100	2000		10	1000	0
90 R 2	Cz-M2	100	2000		10	1000	0
80 R	Cz-M2	100	2000		10	1000	0
80 R 2	Cz-M2	100	2000		10	1000	0
70 R	Cz-M2	100	2000		10	1000	0
70 R 2	Cz-M2	100	2000		10	1000	0
60 R	Cz-M2	100	2000		10	1000	0
60 R 2	Cz-M2	100	2000		10	1000	0
60 R 3	Cz-M2	100	2000		10	1000	0
50 R	Cz-M2	100	2000		10	1000	0
50 R 3	Cz-M2	100	2000		10	1000	0
40 R	Cz-M2	100	2000		10	1000	0
40 R 2	Cz-M2	100	2000		10	1000	0
40 R 4	Cz-M2	100	2000		10	1000	0
30 R	Cz-M2	100	2000		10	1000	0
30 R 2	Cz-M2	100	2000		10	1000	0
20 R	Cz-M2	100	2000		10	1000	0
20 R 2	Cz-M2	100	2000		10	1000	0
10 R	Cz-M2	100	2000		10	1000	0
10 R 2	Cz-M2	100	2000		10	1000	0

ABR: ABR 2 4000Hz 2: Cz-M2

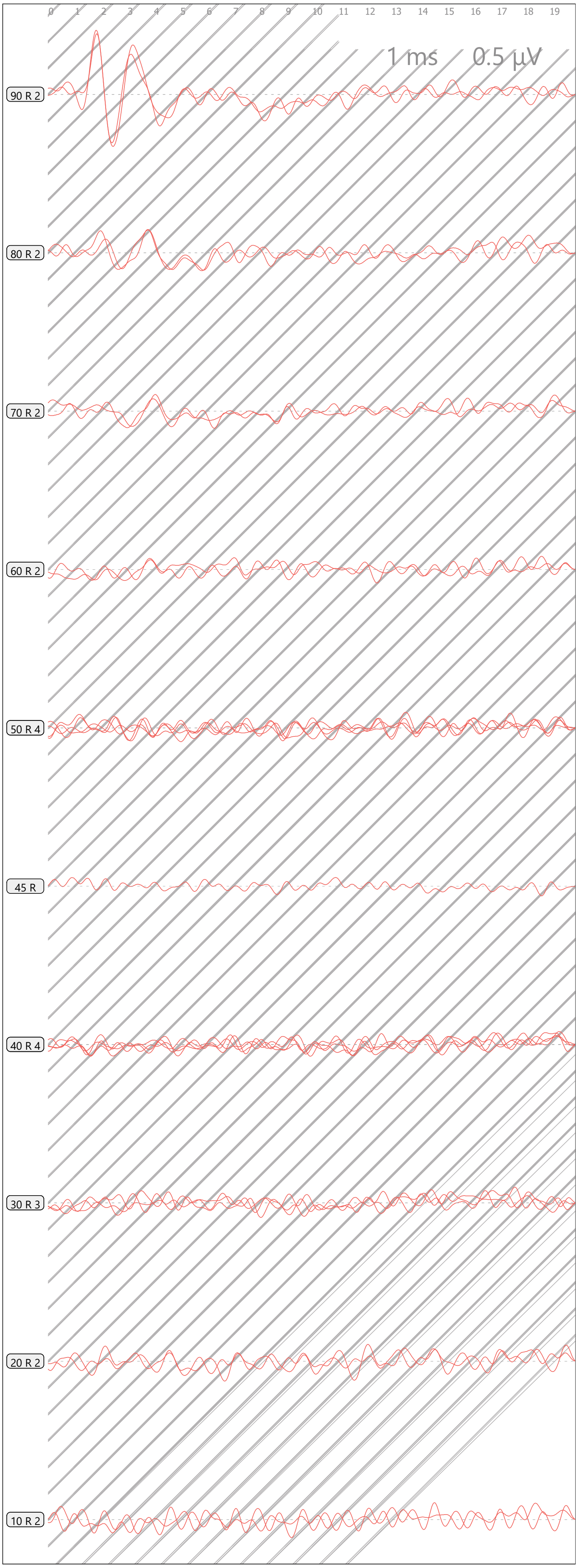
0 1 2 3 4 5 6 7 8 9 10 11 12 13 14 15 16 17 18 19
1 ms
0.5 µV
90 R 2
80 R 2
70 R 2
60 R 2
50 R 4
45 R
40 R 4
30 R 3
20 R 2
10 R 2

Trace parameters

N	Electr.	HPF, Hz	LPF, Hz	50 Hz	Rejection ±μV	Aver.	Reject
90 R	Cz-M2	200	2000		10	1000	0
90 R 2	Cz-M2	200	2000		10	1000	0
80 R	Cz-M2	200	2000		10	1000	0
80 R 2	Cz-M2	200	2000		10	1000	0
70 R	Cz-M2	200	2000		10	1000	0
70 R 2	Cz-M2	200	2000		10	1000	0
60 R	Cz-M2	200	2000		10	1000	0
60 R 2	Cz-M2	200	2000		10	1000	0
50 R	Cz-M2	200	2000		10	1000	0
50 R 2	Cz-M2	200	2000		10	1000	0
50 R 3	Cz-M2	200	2000		10	1000	0
50 R 4	Cz-M2	200	2000		10	1000	0
45 R	Cz-M2	200	2000		10	1000	0
40 R	Cz-M2	200	2000		10	1000	0
40 R 2	Cz-M2	200	2000		10	1000	0
40 R 3	Cz-M2	200	2000		10	1000	0
40 R 4	Cz-M2	200	2000		10	1000	0
30 R	Cz-M2	200	2000		10	1000	0
30 R 2	Cz-M2	200	2000		10	1000	0
30 R 3	Cz-M2	200	2000		10	1000	0
20 R	Cz-M2	200	2000		10	1000	0
20 R 2	Cz-M2	200	2000		10	1000	0
10 R	Cz-M2	200	2000		10	1000	0
10 R 2	Cz-M2	200	2000		10	1000	0

ABR: ABR 2 8000Hz 2: Cz-M2

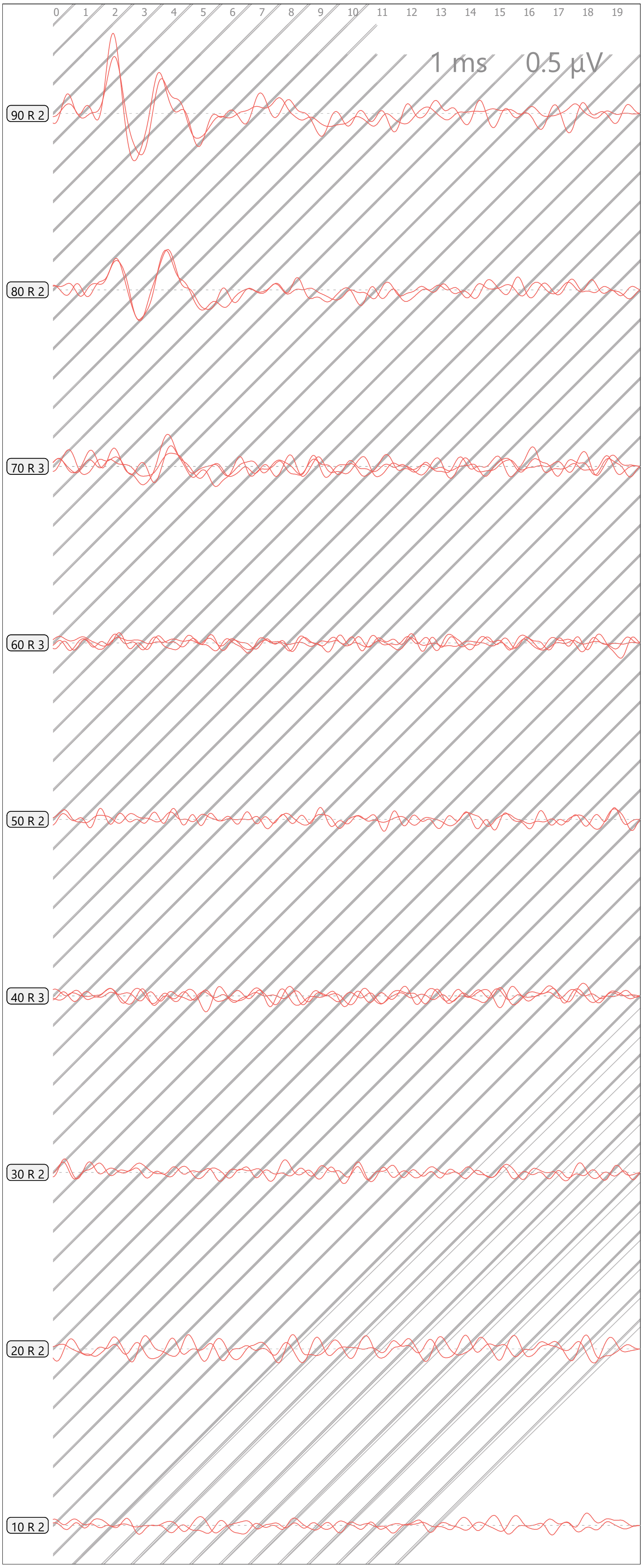
0
1
2
3
4
5
6
7
8
9
10
11
12
13
14
15
16
17
18
19
1 ms
0.5 µV
90 R 2
80 R 2
70 R 3
60 R 3
50 R 2
40 R 3
30 R 2
20 R 2
10 R 2

Trace parameters

N	Electr.	HPF, Hz	LPF, Hz	50 Hz	Rejection ±μV	Aver.	Reject
90 R	Cz-M2	200	2000		10	1000	0
90 R 2	Cz-M2	200	2000		10	1000	0
80 R	Cz-M2	200	2000		10	1000	0
80 R 2	Cz-M2	200	2000		10	1000	0
70 R	Cz-M2	200	2000		10	1000	0
70 R 2	Cz-M2	200	2000		10	1000	0
70 R 3	Cz-M2	200	2000		10	1000	0
60 R	Cz-M2	200	2000		10	1000	0
60 R 2	Cz-M2	200	2000		10	1000	0
60 R 3	Cz-M2	200	2000		10	1000	0
50 R	Cz-M2	200	2000		10	1000	0
50 R 2	Cz-M2	200	2000		10	1000	0
40 R	Cz-M2	200	2000		10	1000	0
40 R 2	Cz-M2	200	2000		10	1000	0
40 R 3	Cz-M2	200	2000		10	1000	0
30 R	Cz-M2	200	2000		10	1000	0
30 R 2	Cz-M2	200	2000		10	1000	0
20 R	Cz-M2	200	2000		10	1000	0
20 R 2	Cz-M2	200	2000		10	1000	0
10 R	Cz-M2	200	2000		10	1000	0
10 R 2	Cz-M2	200	2000		10	1000	0

ECochG: ECochG 1:
Fpz-M1

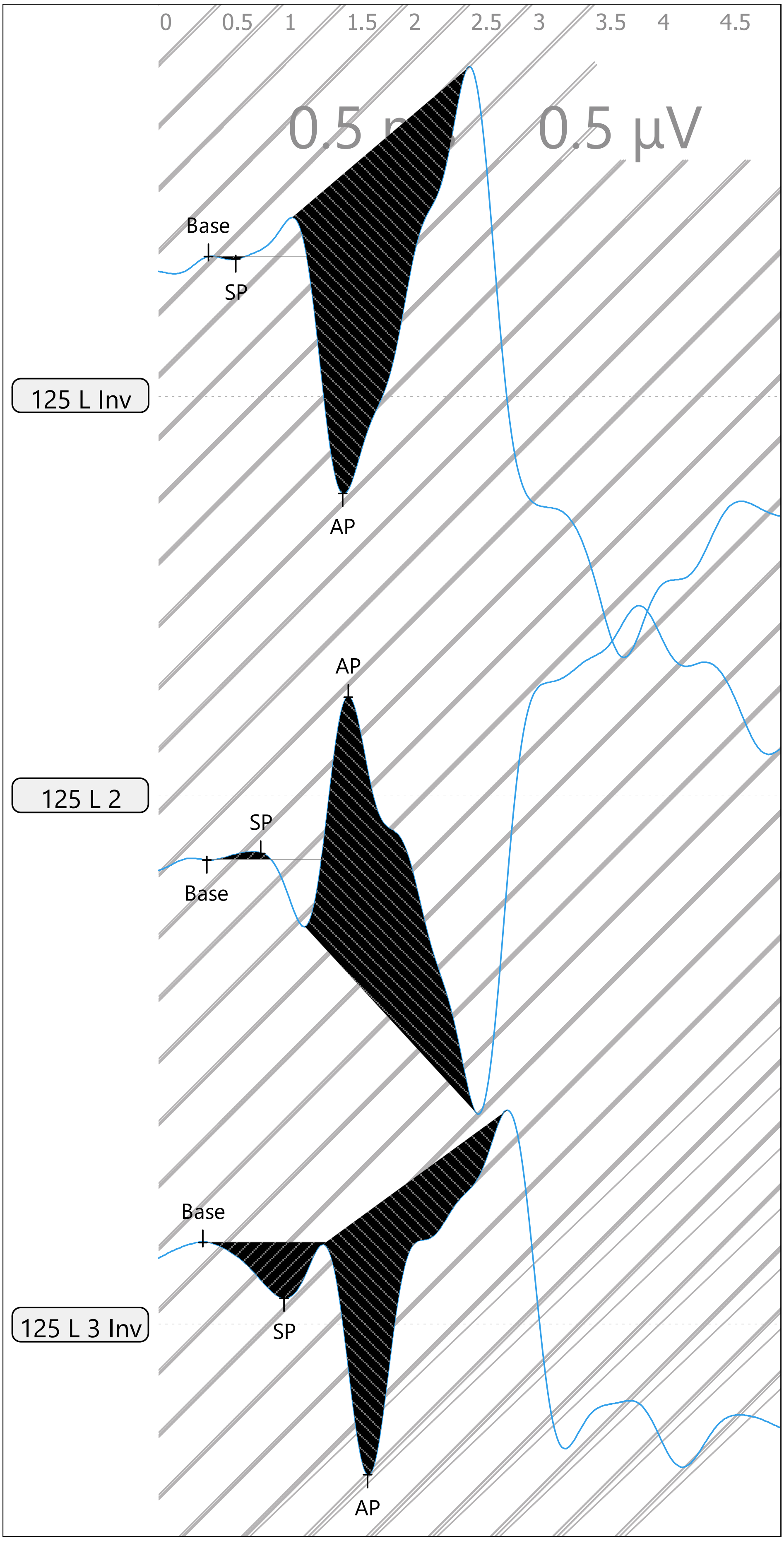

&& (left ear

N	Base (ms)	SP (ms)	AP (ms)	SP–Base (ms)	AP–Base (ms)	SP–Base (μV)	AP–Base (μV)

125 L Inv	0.40	0.62	1.48	0.22	1.08	0.02	1.90	0
125 L 2	0.38	0.82	1.52	0.44	1.14	0.05	1.31	0
125 L 3 Inv	0.36	1.01	1.68	0.65	1.32	0.45	1.86	0

Trace parameters

N	Electr.	HPF, Hz	LPF, Hz	50 Hz	Rejection ±µV	Aver.	R
125 L Inv	Fpz-M1	5	2000		50	1500	
125 L 2	Fpz-M1	5	2000		50	1500	
125 L 3 Inv	Fpz-M1	5	2000		50	1500	

ECochG: ECochG 2:
Fpz-M2

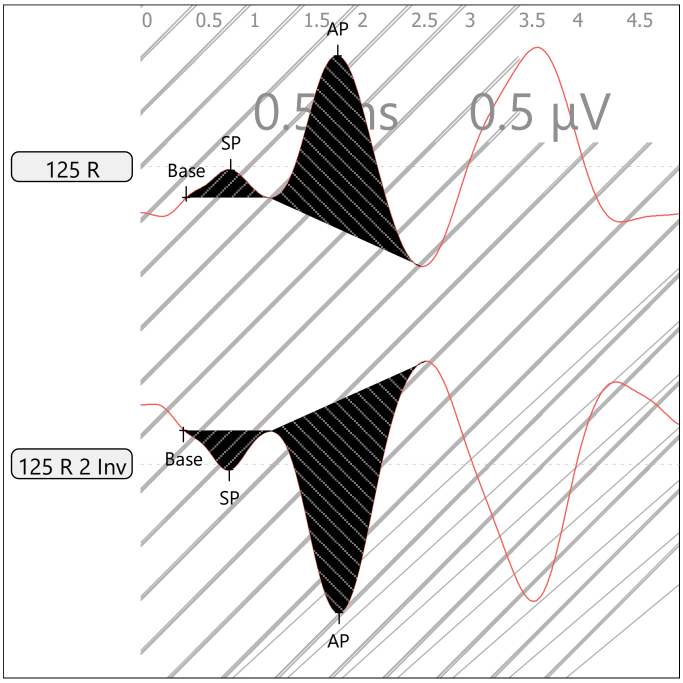

&& (right ear

N	Base (ms)	SP (ms)	AP (ms)	SP–Base (ms)	AP–Base (ms)	SP–Base (µV)	AP–Base (µV)	
125 R	0.42	0.83	1.83	0.41	1.40	0.25	1.31	0
125 R 2 Inv	0.40	0.82	1.84	0.42	1.44	0.37	1.69	0

Trace parameters

N	Electr.	HPF, Hz	LPF, Hz	50 Hz	Rejection ±µV	Aver.	R
125 R	Fpz-M2	5	2000		50	1500	
125 R 2 Inv	Fpz-M2	5	2000		50	1500	

CONCLUSION:

Doctor:
