# Supplementary material for: Exposure to high-altitude hypobaric hypoxic environment induces low-frequency hearing loss in C57BL/6J mice: Mediated by slowing down the postsynaptic electrical signal transmission speed in the cochlear-inferior colliculus auditory signaling pathway
Source: PLoS One. 2026 Mar 11;21(3):e0342321. doi: 10.1371/journal.pone.0342321 (PMC12978441; doi:10.1371/journal.pone.0342321)
Supplement: S1 File — (ZIP) [file pone.0342321.s001.zip › 2025-6-11-10d-01.pdf]

Exam report

Patient: 2025-6-11-10d-01**,** - (-)
Date: June 11， 2025

ABR: ABR 2 CLICK
1: Cz-M1

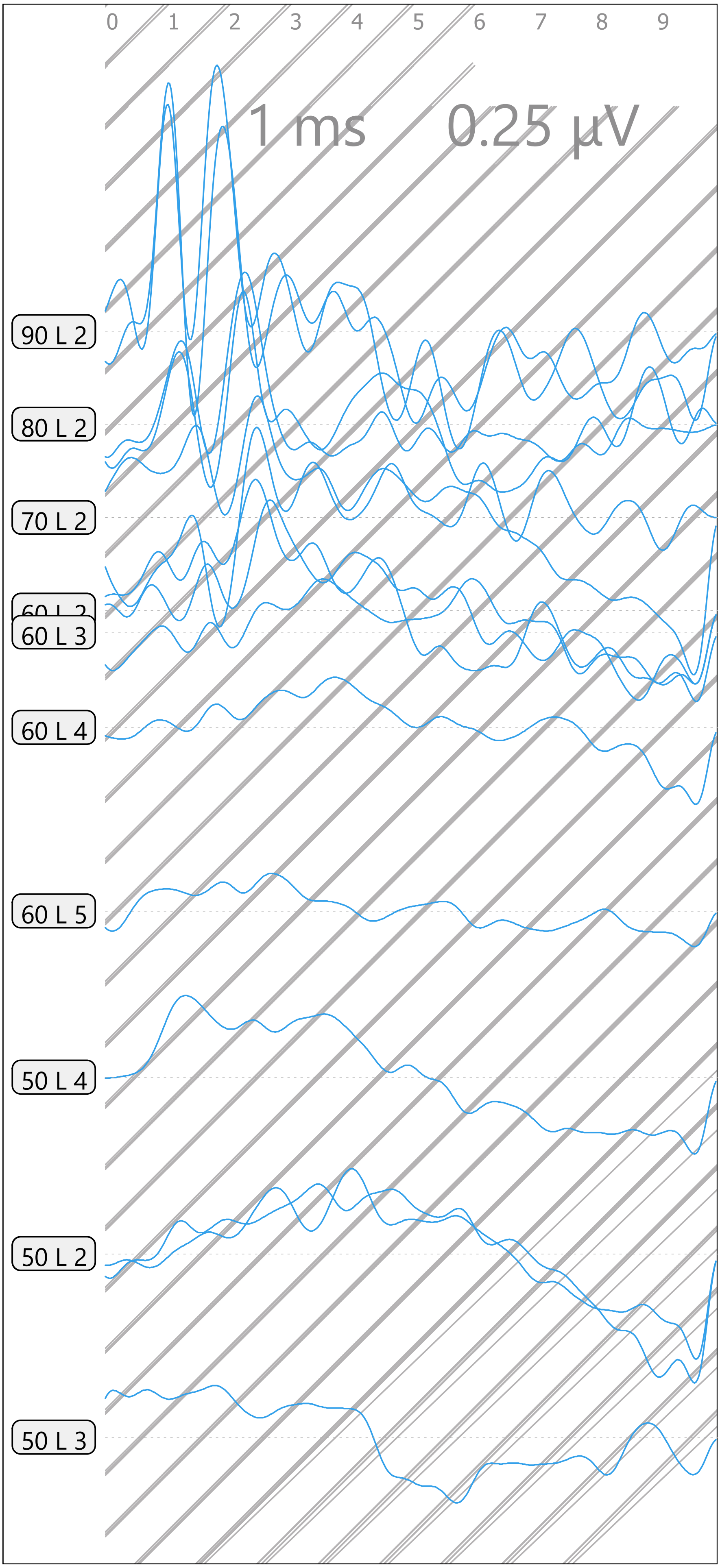

Trace parameters

N	Electr.	HPF, Hz	LPF, Hz	50 Hz	Rejection ±µV	Aver.	Reject.
90 L	Cz-M1	100	2000		10	1000	0
90 L 2	Cz-M1	100	2000		10	1000	0
80 L	Cz-M1	100	2000		10	1000	0
80 L 2	Cz-M1	100	2000		10	1000	0
70 L	Cz-M1	100	2000		10	1000	0
70 L 2	Cz-M1	100	2000		10	1000	0
60 L	Cz-M1	100	2000		10	1000	0
60 L 2	Cz-M1	100	2000		10	1000	0
60 L 3	Cz-M1	100	2000		10	1000	0
60 L 4	Cz-M1	100	2000		10	1000	0
60 L 5	Cz-M1	100	2000		10	1000	0
50 L	Cz-M1	100	2000		10	1000	0
50 L 2	Cz-M1	100	2000		10	1000	0
50 L 3	Cz-M1	100	2000		10	1000	0
50 L 4	Cz-M1	100	2000		10	1000	0

ABR: ABR 2 tone burst 4000Hz 1
: Cz-M1

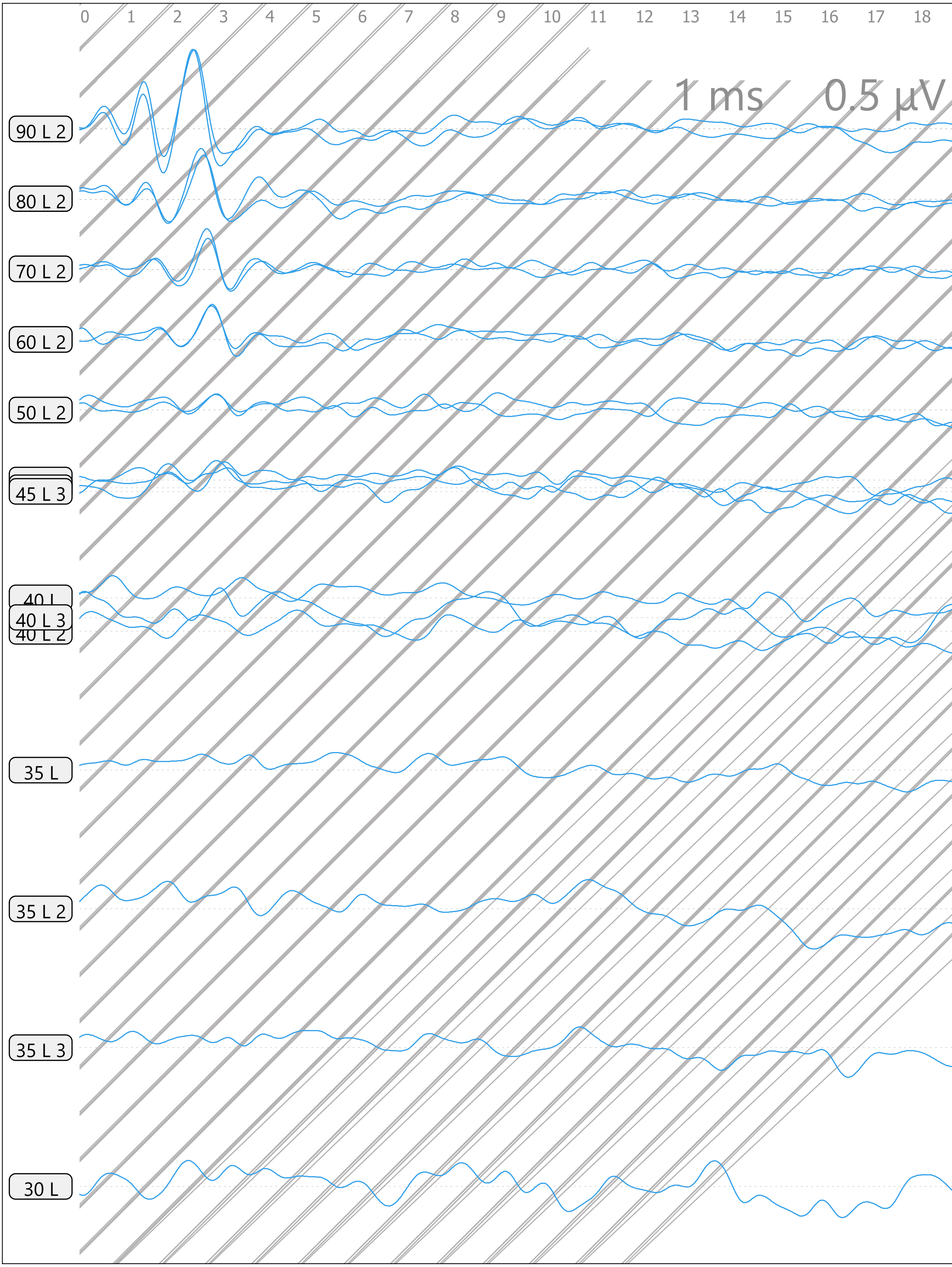
0
1
2
3
4
5
6
7
8
9
10
11
12
13
14
15
16
17
18
1 ms
0.5 µV
90 L 2
80 L 2
70 L 2
60 L 2
50 L 2
45 L 3
40 L 3
35 L
35 L 2
35 L 3
30 L

Trace parameters

N	Electr.	HPF, Hz	LPF, Hz	50 Hz	Rejection ±μV	Aver.	Reject.
90 L	Cz-M1	200	2000		10	1000	0
90 L 2	Cz-M1	200	2000		10	1000	0
80 L	Cz-M1	200	2000		10	1000	0
80 L 2	Cz-M1	200	2000		10	1000	0
70 L	Cz-M1	200	2000		10	1000	0
70 L 2	Cz-M1	200	2000		10	1000	0
60 L	Cz-M1	200	2000		10	1000	0
60 L 2	Cz-M1	200	2000		10	1000	0
50 L	Cz-M1	200	2000		10	1000	0
50 L 2	Cz-M1	200	2000		10	1000	0
45 L	Cz-M1	200	2000		10	1000	0
45 L 2	Cz-M1	200	2000		10	1000	0
45 L 3	Cz-M1	200	2000		10	1000	0
40 L	Cz-M1	200	2000		10	1000	0
40 L 2	Cz-M1	200	2000		10	1000	0
40 L 3	Cz-M1	200	2000		10	1000	0
35 L	Cz-M1	200	2000		10	1000	0
35 L 2	Cz-M1	200	2000		10	933	0
35 L 3	Cz-M1	200	2000		10	1000	0
30 L	Cz-M1	200	2000		10	1000	0

ABR: ABR 2 8000Hz 1: Cz-M1

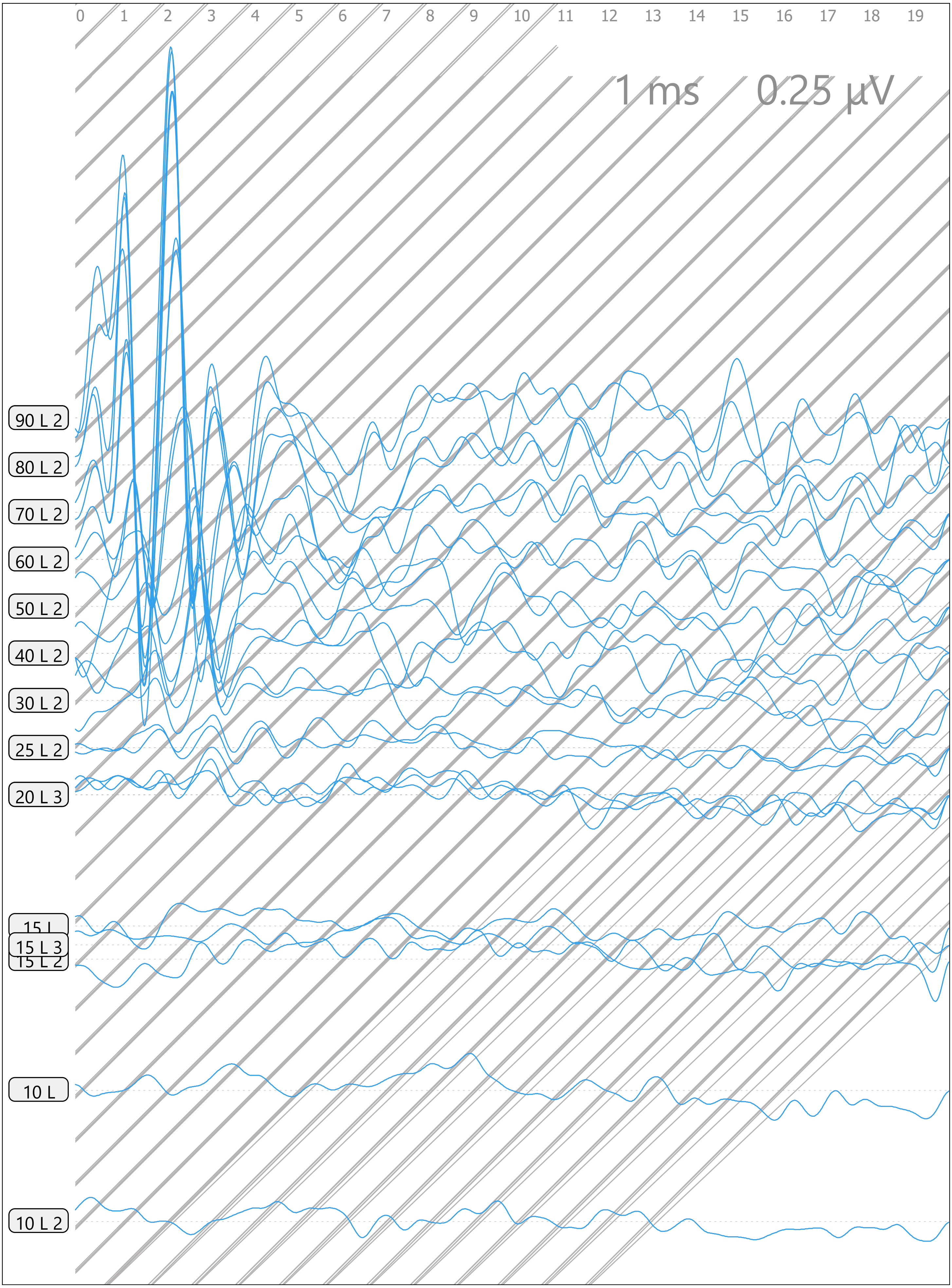

0 1 2 3 4 5 6 7 8 9 10 11 12 13 14 15 16 17 18 19
1 ms
0.25 µV
90 L 2
80 L 2
70 L 2
60 L 2
50 L 2
40 L 2
30 L 2
25 L 2
20 L 3
15 L
15 L 3
15 L 2
10 L
10 L 2

Trace parameters

N	Electr.	HPF, Hz	LPF, Hz	50 Hz	Rejection ±μV	Aver.	Reject.
90 L	Cz-M1	200	2000		10	1000	0
90 L 2	Cz-M1	200	2000		10	1000	0
80 L	Cz-M1	200	2000		10	1000	0
80 L 2	Cz-M1	200	2000		10	1000	0
70 L	Cz-M1	200	2000		10	1000	0
70 L 2	Cz-M1	200	2000		10	1000	0
60 L	Cz-M1	200	2000		10	1000	0
60 L 2	Cz-M1	200	2000		10	1000	0
50 L	Cz-M1	200	2000		10	1000	0
50 L 2	Cz-M1	200	2000		10	1000	0
40 L	Cz-M1	200	2000		10	1000	0
40 L 2	Cz-M1	200	2000		10	1000	0
30 L	Cz-M1	200	2000		10	1000	0
30 L 2	Cz-M1	200	2000		10	1000	0
25 L	Cz-M1	200	2000		10	1000	0
25 L 2	Cz-M1	200	2000		10	1000	0
20 L	Cz-M1	200	2000		10	1000	0
20 L 2	Cz-M1	200	2000		10	1000	0
20 L 3	Cz-M1	200	2000		10	1000	0
15 L	Cz-M1	200	2000		10	1000	0
15 L 2	Cz-M1	200	2000		10	1000	0
15 L 3	Cz-M1	200	2000		10	1000	0
10 L	Cz-M1	200	2000		10	815	0
10 L 2	Cz-M1	200	2000		10	1000	0

ABR: ABR 2 CLICK 2: Cz-M2

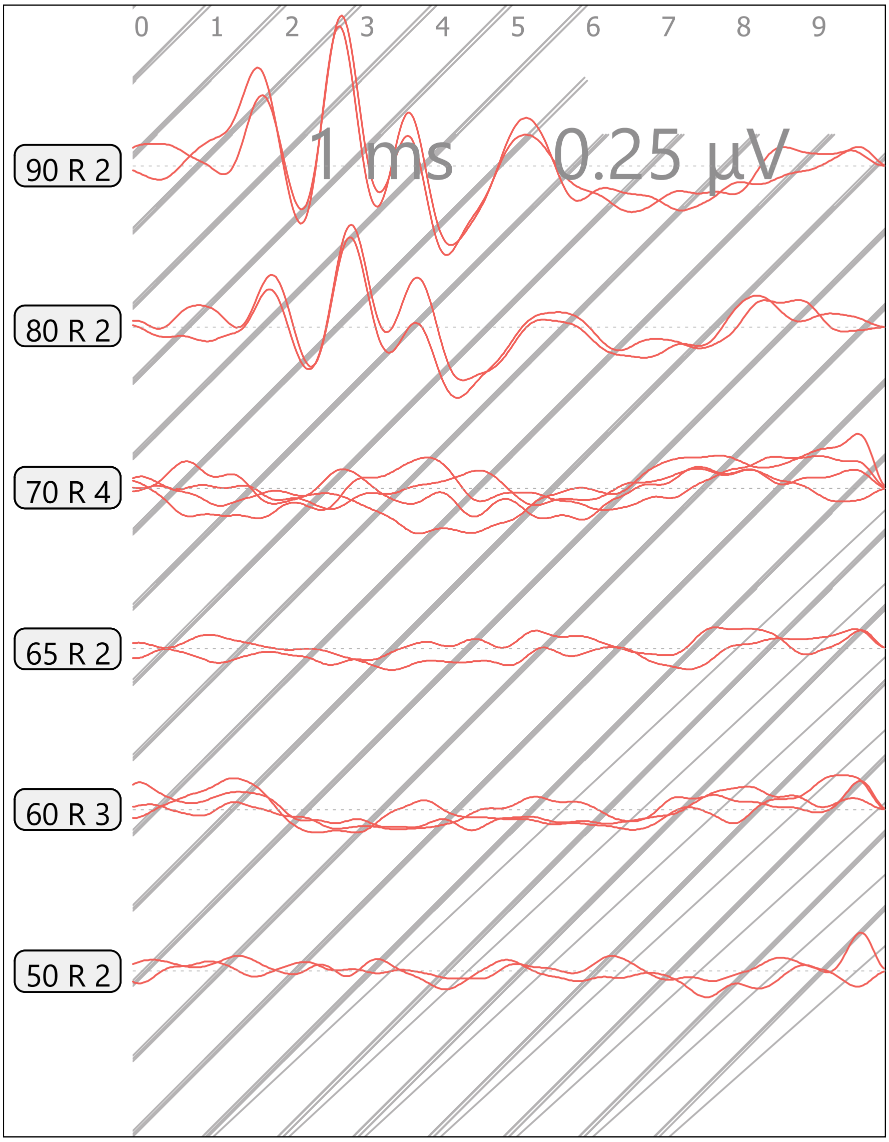

Trace parameters

N	Electr.	HPF, Hz	LPF, Hz	50 Hz	Rejection ±µV	Aver.	Reject
90 R	Cz-M2	100	2000		10	1000	0
90 R 2	Cz-M2	100	2000		10	1000	0
80 R	Cz-M2	100	2000		10	1000	0
80 R 2	Cz-M2	100	2000		10	1000	0
70 R	Cz-M2	100	2000		10	1000	0
70 R 2	Cz-M2	100	2000		10	1000	0
70 R 3	Cz-M2	100	2000		10	1000	0
70 R 4	Cz-M2	100	2000		10	1000	0
65 R	Cz-M2	100	2000		10	1000	0
65 R 2	Cz-M2	100	2000		10	1000	0
60 R	Cz-M2	100	2000		10	1000	0
60 R 2	Cz-M2	100	2000		10	1000	0
60 R 3	Cz-M2	100	2000		10	1000	0
50 R	Cz-M2	100	2000		10	613	0
50 R 2	Cz-M2	100	2000		10	634	0

ABR: ABR 2 4000Hz 2: Cz-M2

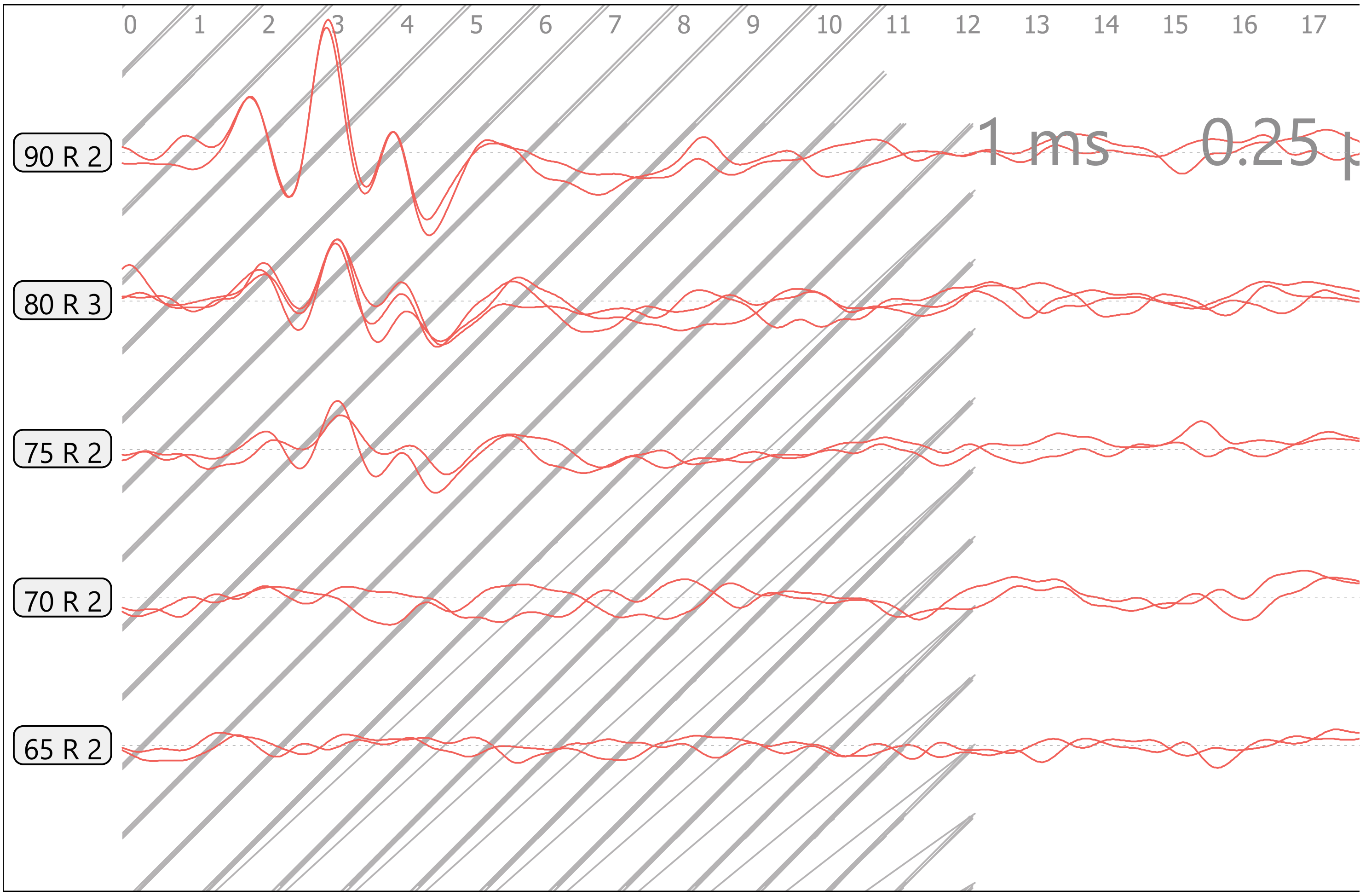

Trace parameters

N	Electr.	HPF, Hz	LPF, Hz	50 Hz	Rejection ±µV	Aver.	Reject
90 R	Cz-M2	200	2000		10	1000	0
90 R 2	Cz-M2	200	2000		10	1000	0
80 R	Cz-M2	200	2000		10	1000	0
80 R 2	Cz-M2	200	2000		10	1000	0
80 R 3	Cz-M2	200	2000		10	1000	0
75 R	Cz-M2	200	2000		10	1000	0
75 R 2	Cz-M2	200	2000		10	1000	0
70 R	Cz-M2	200	2000		10	1000	0
70 R 2	Cz-M2	200	2000		10	1000	0
65 R	Cz-M2	200	2000		10	1000	0
65 R 2	Cz-M2	200	2000		10	1000	0

ABR: ABR 2 8000Hz 2: Cz-M2

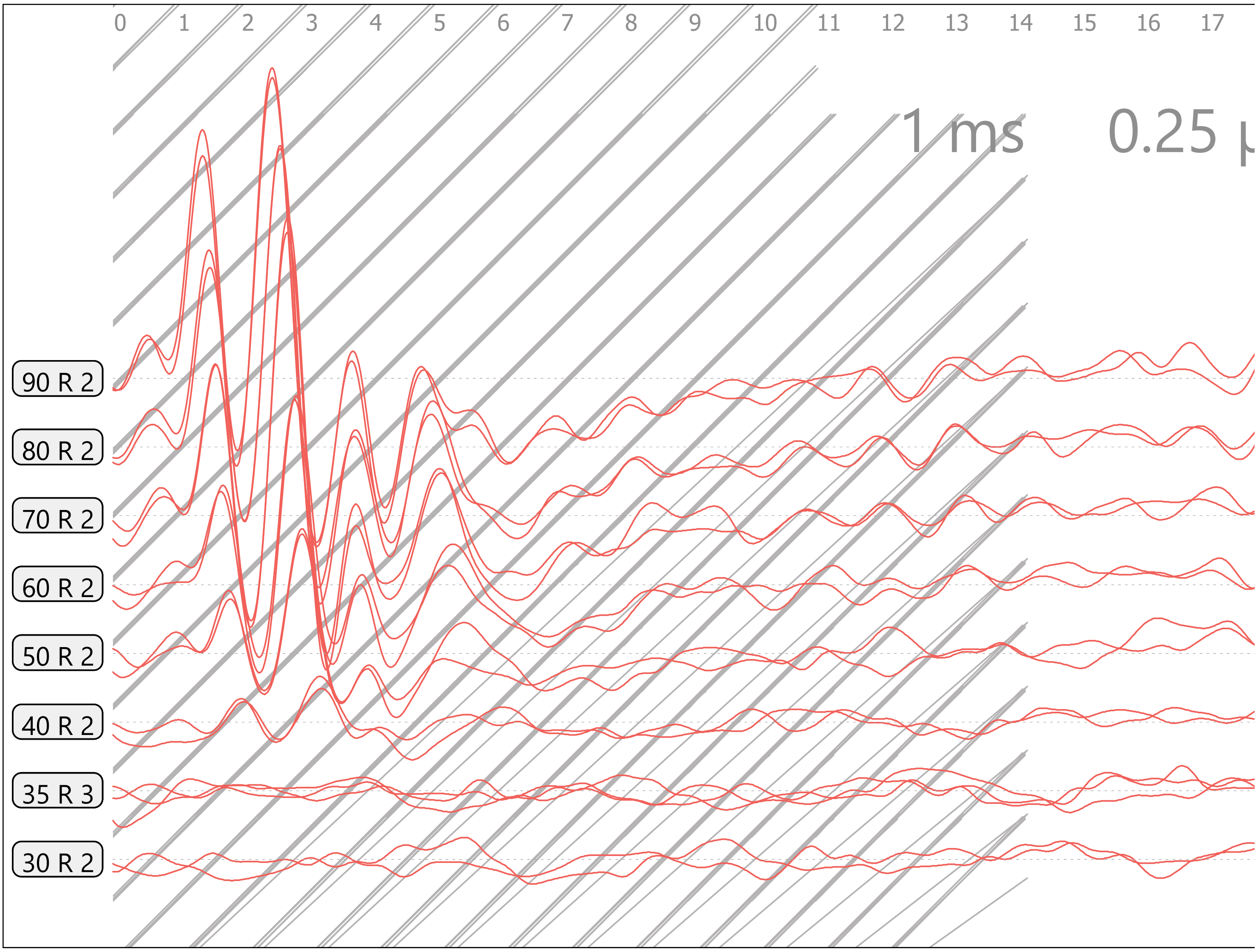

Trace parameters

N	Electr.	HPF, Hz	LPF, Hz	50 Hz	Rejection ±μV	Aver.	Reject
90 R	Cz-M2	200	2000		10	1000	0
90 R 2	Cz-M2	200	2000		10	1000	0
80 R	Cz-M2	200	2000		10	1000	0
80 R 2	Cz-M2	200	2000		10	1000	0
70 R	Cz-M2	200	2000		10	1000	0
70 R 2	Cz-M2	200	2000		10	1000	0
60 R	Cz-M2	200	2000		10	1000	0
60 R 2	Cz-M2	200	2000		10	1000	0
50 R	Cz-M2	200	2000		10	1000	0
50 R 2	Cz-M2	200	2000		10	1000	0
40 R	Cz-M2	200	2000		10	1000	0
40 R 2	Cz-M2	200	2000		10	1000	0
35 R	Cz-M2	200	2000		10	1000	0

35 R 2	Cz-M2	200	2000		10	1000	0
35 R 3	Cz-M2	200	2000		10	1000	0
30 R	Cz-M2	200	2000		10	1000	0
30 R 2	Cz-M2	200	2000		10	1000	0

DPOAE: 1-12 kHz 70/70 dB 3 points

Test result (right ear):

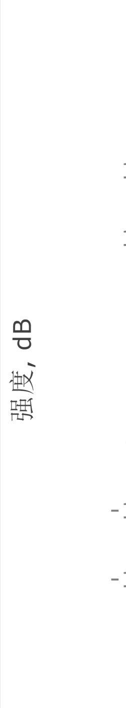

DPOAE (left ear)

F2, Hz	L1, dB	L2, dB	DP, dB	dB	SNR, dB	OAE
988	67.9	68.7	-18.18	-12.28	-5.9	✗
1270	68.8	69.5	-12.97	-15.00	2.0	✗
1778	69.6	70.2	-8.31	-15.00	6.7	✓
2222	70.0	70.5	-11.07	-15.00	3.9	✗
2500	70.1	70.6	-8.66	-15.00	6.3	✓
3200	70.4	71.0	-15.02	-15.00	0.0	✗
4444	70.9	71.1	7.10	-8.73	15.8	✓
5000	70.9	70.9	10.43	-11.64	22.1	✓
6154	70.7	71.0	9.62	-10.29	19.9	✓
8000	70.6	70.7	17.22	-9.38	26.6	✓
8889	71.1	66.8	18.73	-11.87	30.6	✓
10000	70.9	56.1	13.68	-8.17	21.9	✓
11429	57.4	60.5	3.74	-5.69	9.4	✓
(dB SPL)	:: 0.0					

DPOAE: 1-12 kHz 70/70 dB 3 points

Test result (right ear): REFER

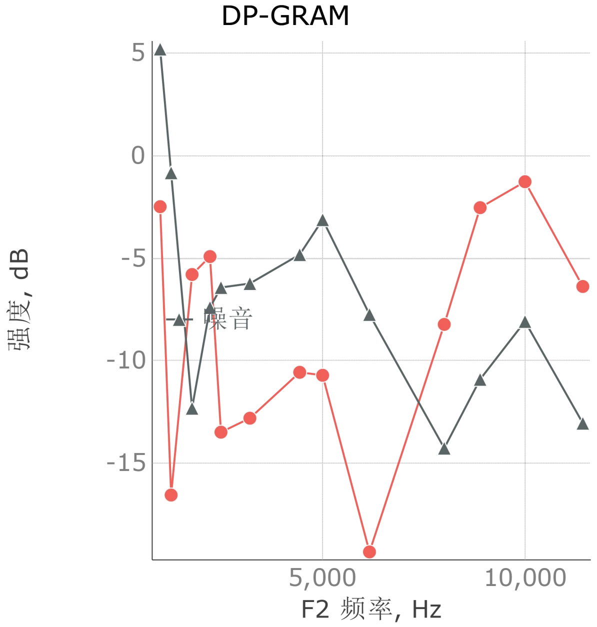

DPOAE

F2, Hz	L1, dB	L2, dB	DP, dB	dB	SNR, dB	OAE
988	68.1	68.4	-2.49	5.21	-7.7	✘
1270	68.7	69.1	-16.57	-0.83	-15.7	✘
1778	69.6	69.7	-5.78	-12.32	6.5	✘
2222	70.0	70.0	-4.92	-7.40	2.5	✘
2500	70.2	70.1	-13.49	-6.42	-7.1	✘
3200	70.4	70.4	-12.82	-6.23	-6.6	✘
4444	71.2	70.4	-10.54	-4.81	-5.7	✘
5000	71.4	70.1	-10.70	-3.13	-7.6	✘
6154	70.2	70.0	-19.32	-7.72	-11.6	✘
8000	70.2	71.0	-8.22	-14.27	6.0	✘
8889	70.4	69.6	-2.55	-10.89	8.3	✓
10000	71.4	56.1	-1.29	-8.06	6.8	✓
11429	57.1	53.8	-6.38	-13.04	6.7	✘
(dB SPL)	:: 0.0					

ECochG: ECochG 1:
Fpz-M1

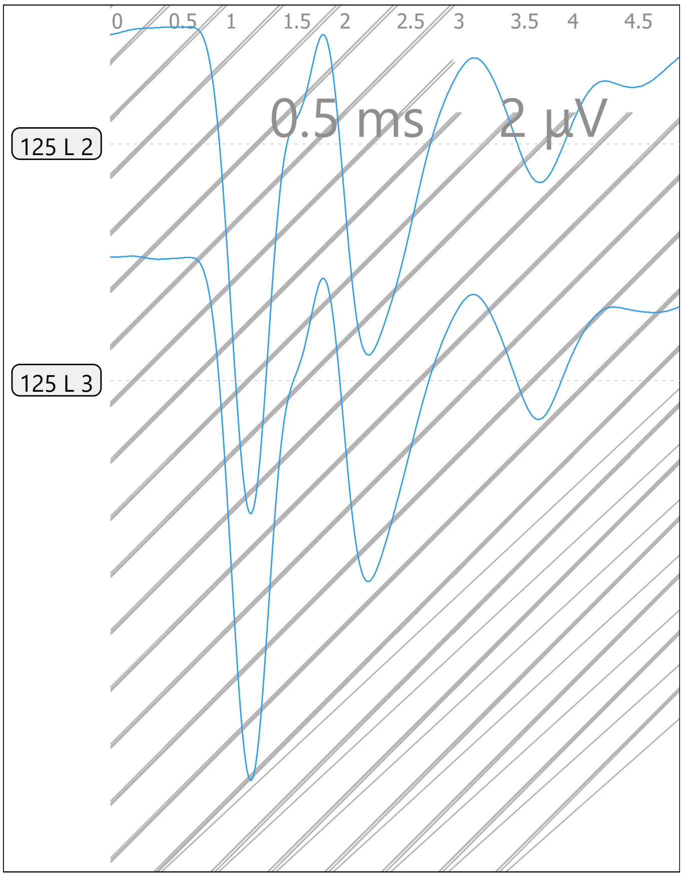

Trace parameters

N	Electr.	HPF, Hz	LPF, Hz	50 Hz	Rejection ±µV	Aver.	Rejec
125 L 2	Fpz-M1	5	2000		50	1027	68
125 L 3	Fpz-M1	5	2000		50	883	56

ECochG: ECochG 2:
Fpz-M2

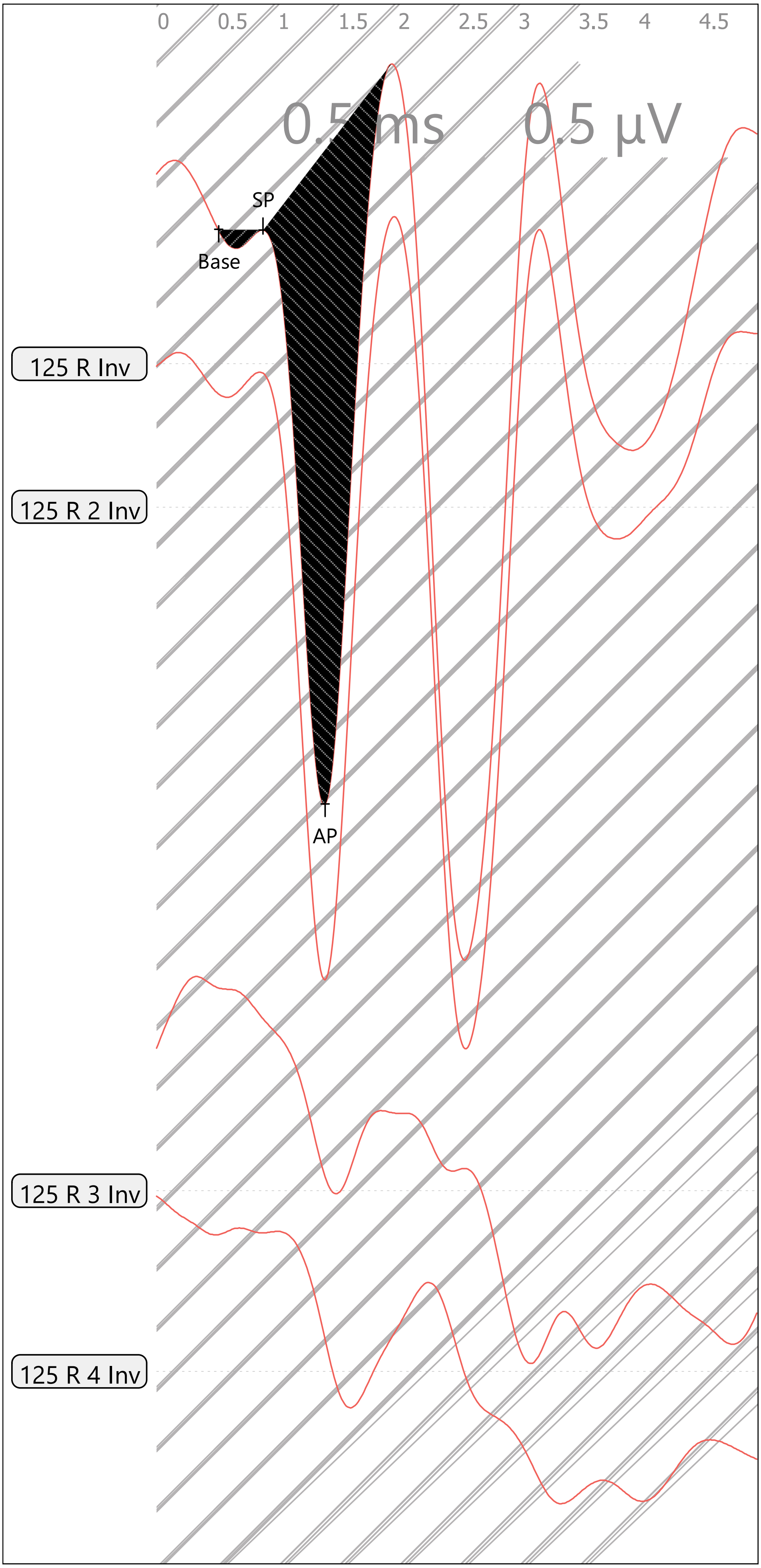
0
0.5
1
1.5
2
2.5
3
3.5
4
4.5
0.5 ms
0.5 µV
SP
Base
AP
125 R Inv
125 R 2 Inv
125 R 3 Inv
125 R 4 Inv

&& (right ear

N	Base (ms)	SP (ms)	AP (ms)	SP–Base (ms)	AP–Base (ms)	SP–Base (µV)	AP–Base (µV)	
125 R Inv	0.52	0.89	1.40	0.37	0.89	0.00	4.77	0.0

Trace parameters

N	Electr.	HPF, Hz	LPF, Hz	50 Hz	Rejection ±µV	Aver.	R
125 R Inv	Fpz-M2	5	2000		50	1500	
125 R 2 Inv	Fpz-M2	5	2000		50	1365	
125 R 3 Inv	Fpz-M2	5	2000		50	1367	
125 R 4 Inv	Fpz-M2	5	2000		50	1089	

CONCLUSION:

Doctor:
