# Supplementary material for: Exposure to high-altitude hypobaric hypoxic environment induces low-frequency hearing loss in C57BL/6J mice: Mediated by slowing down the postsynaptic electrical signal transmission speed in the cochlear-inferior colliculus auditory signaling pathway
Source: PLoS One. 2026 Mar 11;21(3):e0342321. doi: 10.1371/journal.pone.0342321 (PMC12978441; doi:10.1371/journal.pone.0342321)
Supplement: S1 File — (ZIP) [file pone.0342321.s001.zip › 2025-6-11-10d-02.pdf]

Exam report

Patient: 2025-6-11-10d-02, - (-)
Date: June 11, 2025

ABR: ABR 2 CLICK
2: Cz-M

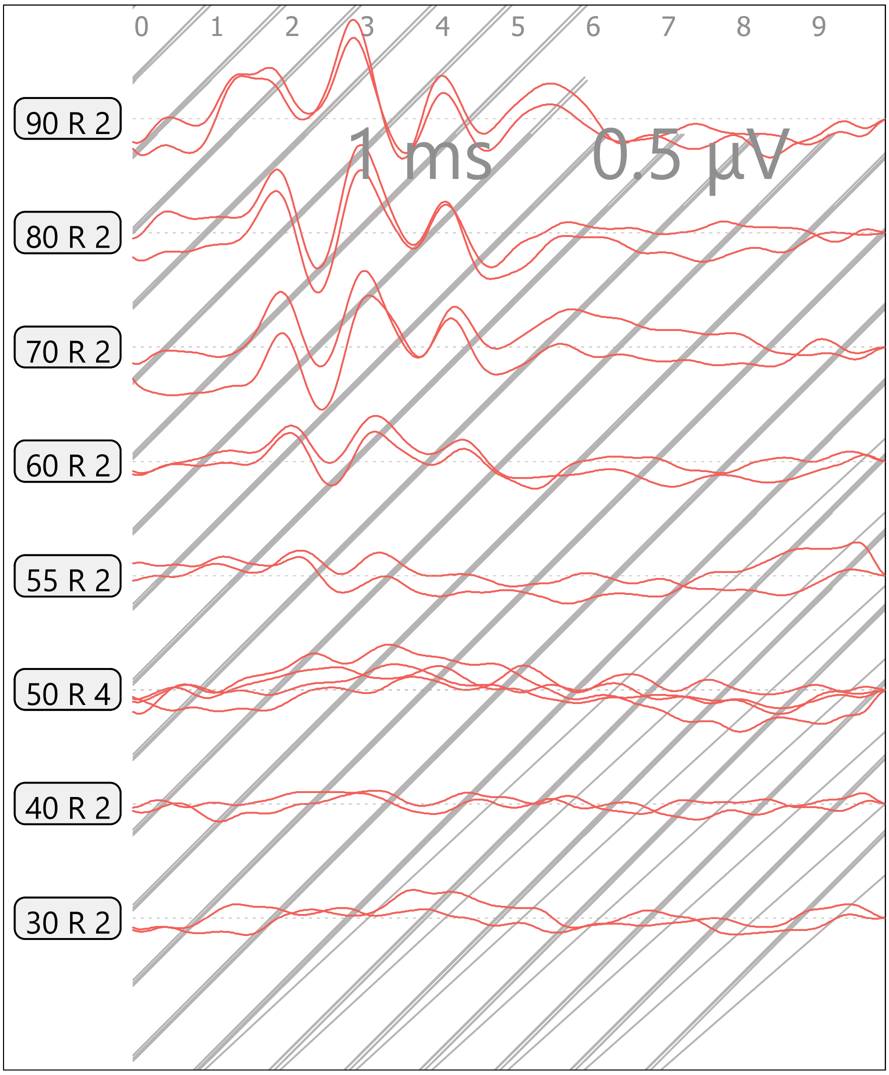

Trace parameters

N	Electr.	HPF, Hz	LPF, Hz	50 Hz	Rejection ±µV	Aver.	Reject
90 R	Cz-M	100	2000		10	1000	0
90 R 2	Cz-M	100	2000		10	1000	0
80 R	Cz-M	100	2000		10	1000	0
80 R 2	Cz-M	100	2000		10	1000	0
70 R	Cz-M	100	2000		10	1000	0
70 R 2	Cz-M	100	2000		10	1000	0
60 R	Cz-M	100	2000		10	1000	0
60 R 2	Cz-M	100	2000		10	1000	0
55 R	Cz-M	100	2000		10	1000	0
55 R 2	Cz-M	100	2000		10	1000	0
50 R	Cz-M	100	2000		10	1000	0
50 R 2	Cz-M	100	2000		10	1000	0
50 R 3	Cz-M	100	2000		10	1000	0
50 R 4	Cz-M	100	2000		10	1000	0
40 R	Cz-M	100	2000		10	1000	0
40 R 2	Cz-M	100	2000		10	1000	0

30 R	Cz-M	100	2000		10	1000	0
30 R 2	Cz-M	100	2000		10	1000	0

ABR: ABR 2 CLICK 1: Cz-M1

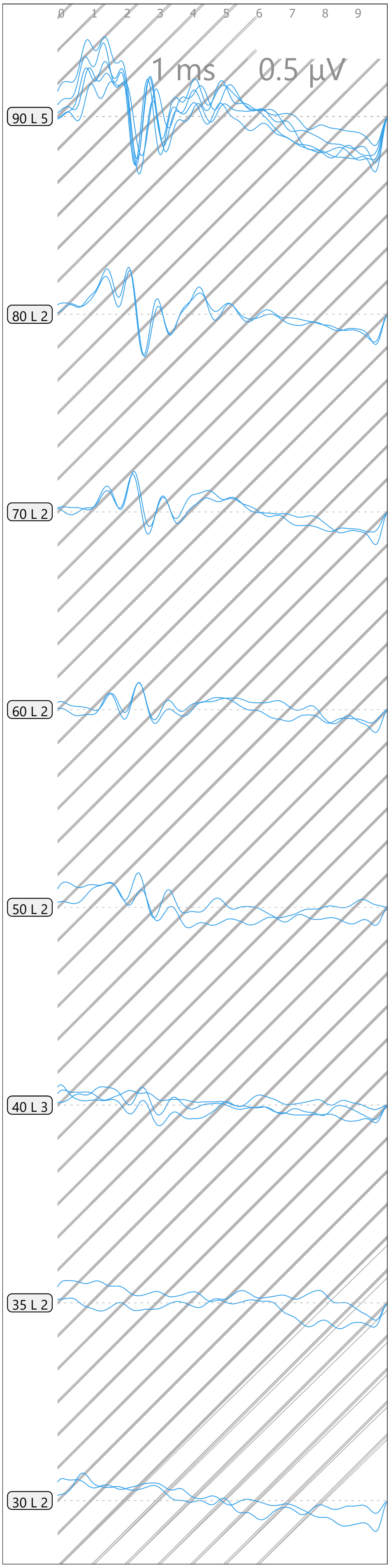
0
1
2
3
4
5
6
7
8
9
1 ms
0.5 µV
90 L 5
80 L 2
70 L 2
60 L 2
50 L 2
40 L 3
35 L 2
30 L 2

Trace parameters

N	Electr.	HPF, Hz	LPF, Hz	50 Hz	Rejection ±μV	Aver.	Reject.
90 L	Cz-M1	100	2000		10	1000	0
90 L 2	Cz-M1	100	2000		10	1000	0
90 L 3	Cz-M1	100	2000		10	1000	0
90 L 4	Cz-M1	100	2000		10	1000	0
90 L 5	Cz-M1	100	2000		10	1000	0
80 L	Cz-M1	100	2000		10	1000	0
80 L 2	Cz-M1	100	2000		10	1000	0
70 L	Cz-M1	100	2000		10	1000	0
70 L 2	Cz-M1	100	2000		10	1000	0
60 L	Cz-M1	100	2000		10	1000	0
60 L 2	Cz-M1	100	2000		10	1000	0
50 L	Cz-M1	100	2000		10	1000	0
50 L 2	Cz-M1	100	2000		10	1000	0
40 L	Cz-M1	100	2000		10	1000	0
40 L 2	Cz-M1	100	2000		10	1000	0
40 L 3	Cz-M1	100	2000		10	1000	0
35 L	Cz-M1	100	2000		10	1000	0
35 L 2	Cz-M1	100	2000		10	879	0
30 L	Cz-M1	100	2000		10	1000	0
30 L 2	Cz-M1	100	2000		10	743	0

ABR: ABR 2 tone burst 4000Hz 1
: Cz-M1

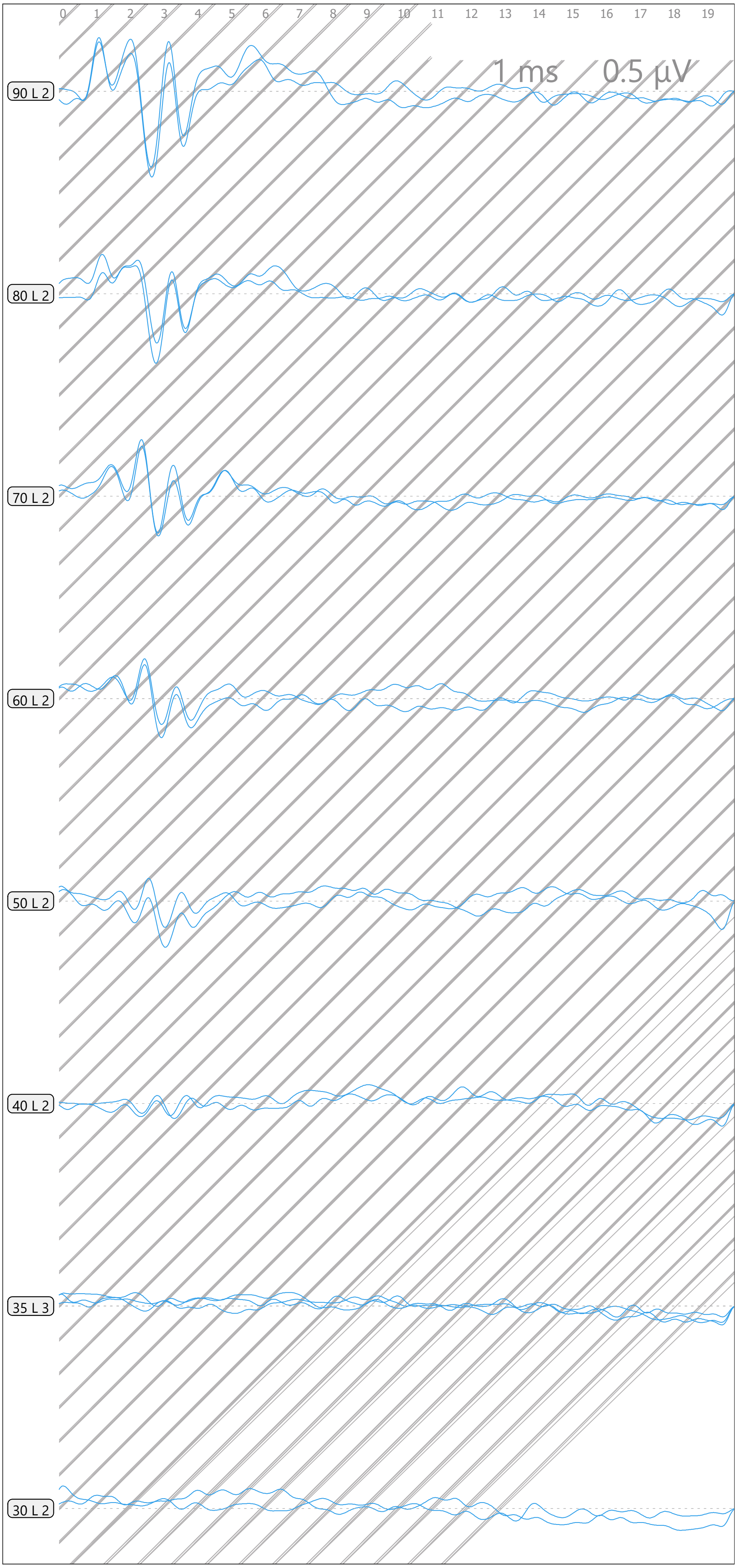

0 1 2 3 4 5 6 7 8 9 10 11 12 13 14 15 16 17 18 19
1 ms
0.5 µV
90 L 2
80 L 2
70 L 2
60 L 2
50 L 2
40 L 2
35 L 3
30 L 2

Trace parameters

N	Electr.	HPF, Hz	LPF, Hz	50 Hz	Rejection ±μV	Aver.	Reject.
90 L	Cz-M1	200	2000		10	1000	0
90 L 2	Cz-M1	200	2000		10	1000	0
80 L	Cz-M1	200	2000		10	1000	0
80 L 2	Cz-M1	200	2000		10	1000	0
70 L	Cz-M1	200	2000		10	1000	0
70 L 2	Cz-M1	200	2000		10	1000	0
60 L	Cz-M1	200	2000		10	1000	0
60 L 2	Cz-M1	200	2000		10	1000	0
50 L	Cz-M1	200	2000		10	1000	0
50 L 2	Cz-M1	200	2000		10	1000	0
40 L	Cz-M1	200	2000		10	1000	0
40 L 2	Cz-M1	200	2000		10	1000	0
35 L	Cz-M1	200	2000		10	1000	0
35 L 2	Cz-M1	200	2000		10	1000	0
35 L 3	Cz-M1	200	2000		10	1000	0
30 L	Cz-M1	200	2000		10	1000	0
30 L 2	Cz-M1	200	2000		10	1000	0

ABR: ABR 2 8000Hz 1: Cz-M1

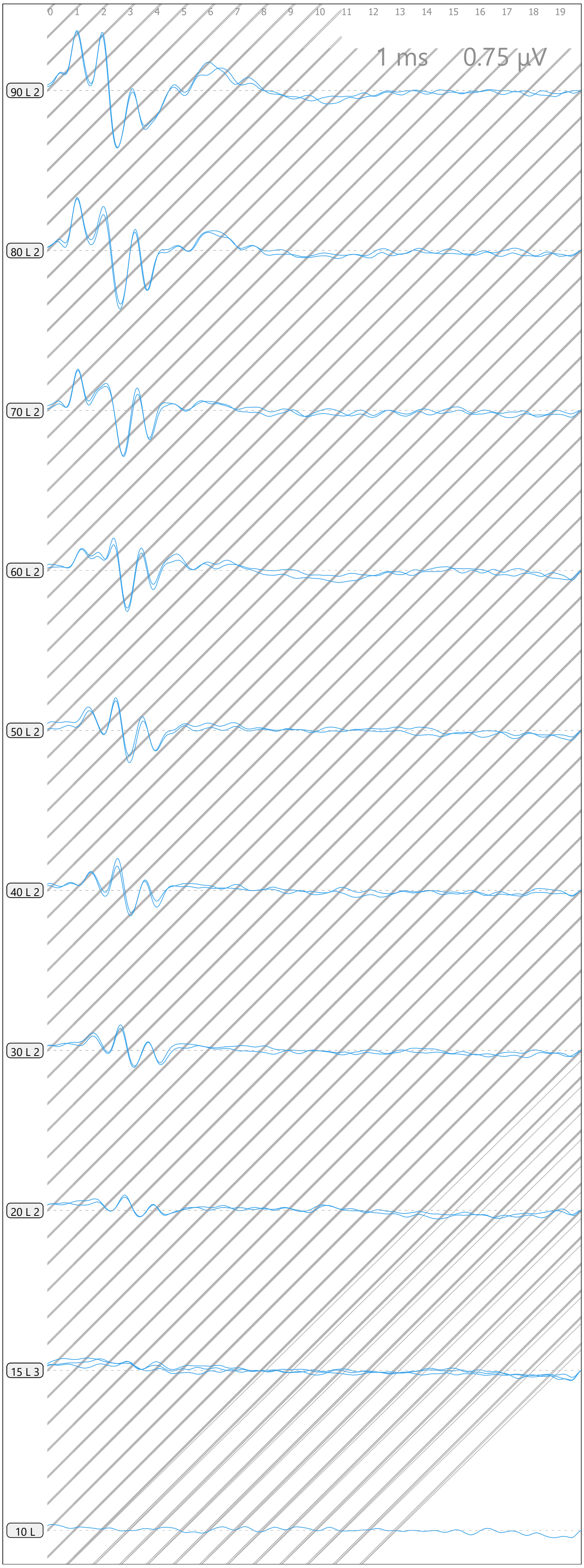

0 1 2 3 4 5 6 7 8 9 10 11 12 13 14 15 16 17 18 19
1 ms
0.75 µV
90 L 2
80 L 2
70 L 2
60 L 2
50 L 2
40 L 2
30 L 2
20 L 2
15 L 3
10 L

Trace parameters

N	Electr.	HPF, Hz	LPF, Hz	50 Hz	Rejection ±μV	Aver.	Reject.
90 L	Cz-M1	200	2000		10	1000	0
90 L 2	Cz-M1	200	2000		10	1000	0
80 L	Cz-M1	200	2000		10	1000	0
80 L 2	Cz-M1	200	2000		10	1000	0
70 L	Cz-M1	200	2000		10	1000	0
70 L 2	Cz-M1	200	2000		10	1000	0
60 L	Cz-M1	200	2000		10	1000	0
60 L 2	Cz-M1	200	2000		10	1000	0
50 L	Cz-M1	200	2000		10	1000	0
50 L 2	Cz-M1	200	2000		10	1000	0
40 L	Cz-M1	200	2000		10	1000	0
40 L 2	Cz-M1	200	2000		10	1000	0
30 L	Cz-M1	200	2000		10	1000	0
30 L 2	Cz-M1	200	2000		10	1000	0
20 L	Cz-M1	200	2000		10	1000	0
20 L 2	Cz-M1	200	2000		10	1000	0
15 L	Cz-M1	200	2000		10	1000	0
15 L 2	Cz-M1	200	2000		10	1000	0
15 L 3	Cz-M1	200	2000		10	1000	0
10 L	Cz-M1	200	2000		10	1000	0

ABR: ABR 2 tone burst 4000Hz 2
: Cz-M2

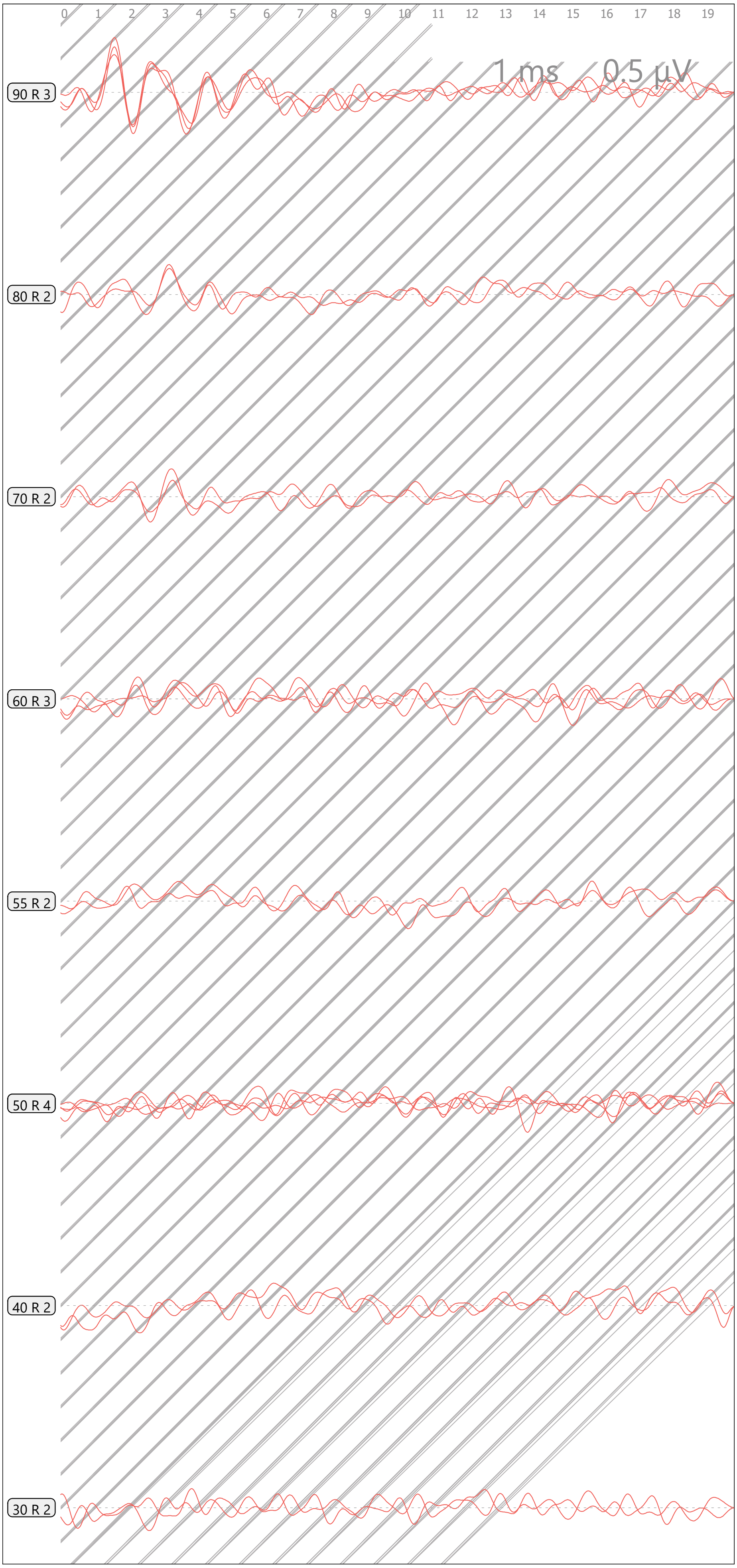
0 1 2 3 4 5 6 7 8 9 10 11 12 13 14 15 16 17 18 19
1 ms
0.5 µV
90 R 3
80 R 2
70 R 2
60 R 3
55 R 2
50 R 4
40 R 2
30 R 2

Trace parameters

N	Electr.	HPF, Hz	LPF, Hz	50 Hz	Rejection ±μV	Aver.	Reject
90 R	Cz-M2	200	2000		10	1000	0
90 R 2	Cz-M2	200	2000		10	1000	0
90 R 3	Cz-M2	200	2000		10	1000	0
80 R	Cz-M2	200	2000		10	1000	0
80 R 2	Cz-M2	200	2000		10	1000	0
70 R	Cz-M2	200	2000		10	1000	0
70 R 2	Cz-M2	200	2000		10	1000	0
60 R	Cz-M2	200	2000		10	1000	0
60 R 2	Cz-M2	200	2000		10	1000	0
60 R 3	Cz-M2	200	2000		10	1000	0
55 R	Cz-M2	200	2000		10	1000	0
55 R 2	Cz-M2	200	2000		10	1000	0
50 R	Cz-M2	200	2000		10	1000	0
50 R 2	Cz-M2	200	2000		10	1000	0
50 R 3	Cz-M2	200	2000		10	1000	0
50 R 4	Cz-M2	200	2000		10	1000	0
40 R	Cz-M2	200	2000		10	1000	0
40 R 2	Cz-M2	200	2000		10	1000	0
30 R	Cz-M2	200	2000		10	1000	0
30 R 2	Cz-M2	200	2000		10	1000	0

ABR: ABR 2 8000Hz 2: Cz-M2

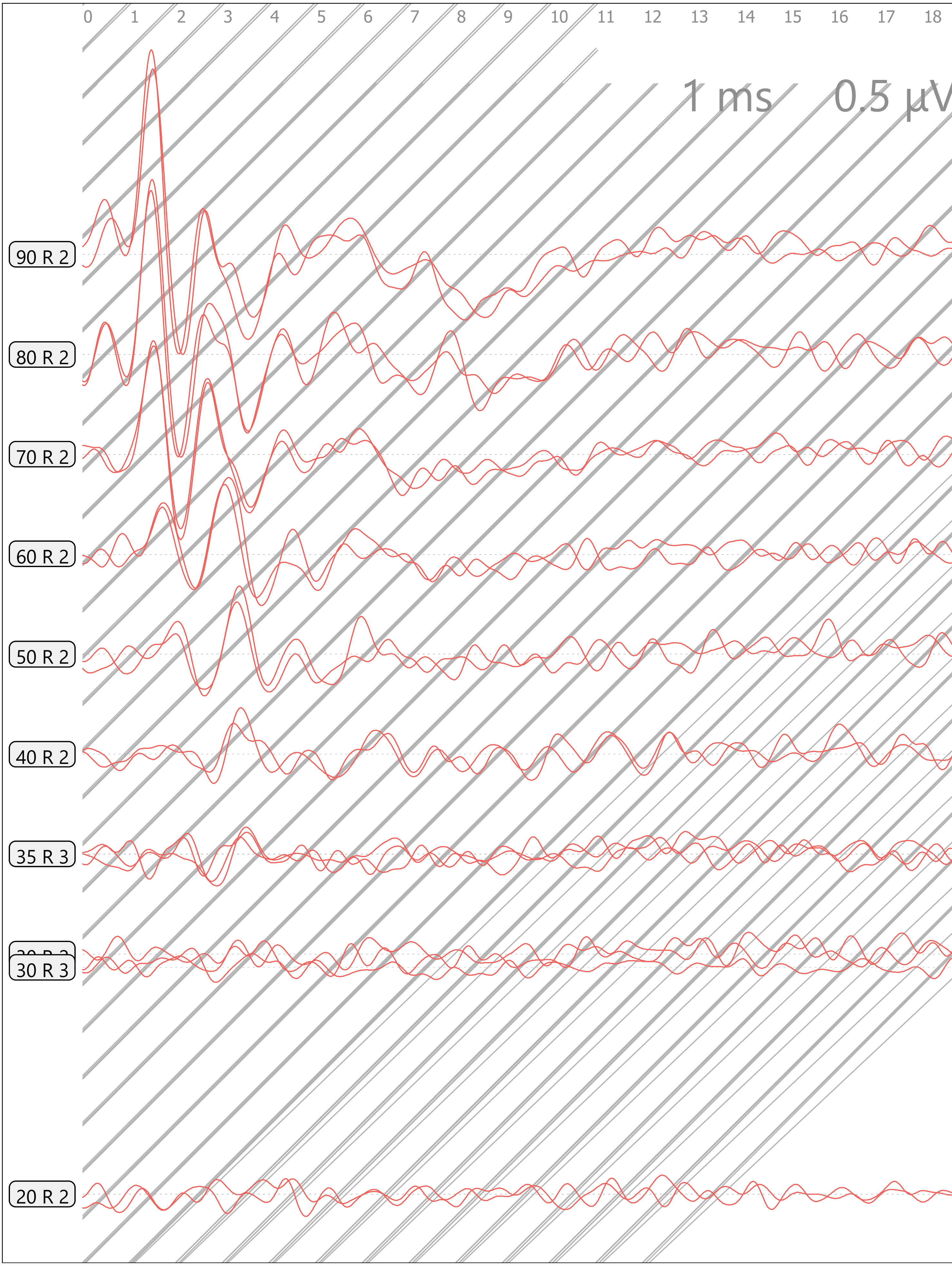
0
1
2
3
4
5
6
7
8
9
10
11
12
13
14
15
16
17
18
1 ms
0.5 µV
90 R 2
80 R 2
70 R 2
60 R 2
50 R 2
40 R 2
35 R 3
30 R 3
20 R 2

Trace parameters

N	Electr.	HPF, Hz	LPF, Hz	50 Hz	Rejection ±µV	Aver.	Reject
90 R	Cz-M2	200	2000		10	1000	0
90 R 2	Cz-M2	200	2000		10	1000	0
80 R	Cz-M2	200	2000		10	1000	0
80 R 2	Cz-M2	200	2000		10	1000	0
70 R	Cz-M2	200	2000		10	1000	0
70 R 2	Cz-M2	200	2000		10	1000	0
60 R	Cz-M2	200	2000		10	1000	0
60 R 2	Cz-M2	200	2000		10	1000	0
50 R	Cz-M2	200	2000		10	1000	0
50 R 2	Cz-M2	200	2000		10	1000	0
40 R	Cz-M2	200	2000		10	1000	0
40 R 2	Cz-M2	200	2000		10	1000	0
35 R	Cz-M2	200	2000		10	1000	0
35 R 2	Cz-M2	200	2000		10	1000	0
35 R 3	Cz-M2	200	2000		10	1000	0
30 R	Cz-M2	200	2000		10	1000	0
30 R 2	Cz-M2	200	2000		10	1000	0
30 R 3	Cz-M2	200	2000		10	1000	0
20 R	Cz-M2	200	2000		10	1000	0
20 R 2	Cz-M2	200	2000		10	1000	0

DPOAE: 1-12 kHz 70/70 dB 3 points

**Test result (right ear):**	

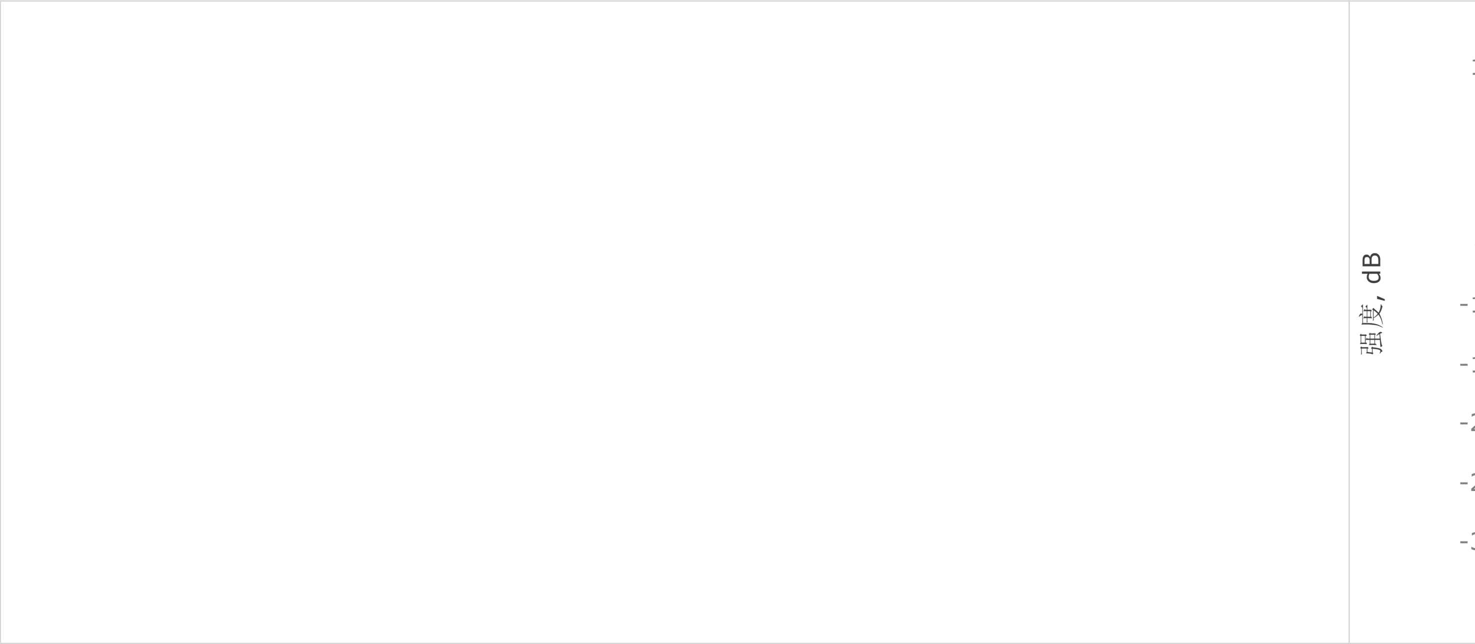

DPOAE

F2, Hz	L1, dB	L2, dB	DP, dB	噪声, dB	SNR, dB	OAE
988	67.9	68.3	-15.55	-10.48	-5.1	✘
1270	68.6	69.0	-13.08	-12.36	-0.7	✘
1778	69.6	69.7	-10.00	-5.40	-4.6	✘
2222	70.0	70.0	-11.59	-12.46	0.9	✘
2500	70.1	70.1	-11.25	-15.00	3.8	✘
3200	70.4	70.3	-13.02	-15.00	2.0	✘
4444	70.3	70.8	-31.15	-15.00	-16.2	✘
5000	69.8	70.8	-7.91	-14.47	6.6	✔
6154	71.1	70.5	-1.62	-12.29	10.7	✔
8000	70.1	71.5	4.29	-12.56	16.9	✔
8889	69.4	73.4	11.15	-9.96	21.1	✔
10000	71.8	62.9	9.62	-3.75	13.4	✔
11429	63.9	51.9	0.13	-6.76	6.9	✔
(dB SPL)	:: 0.0					

ECochG: ECochG 1:
Fpz-M1

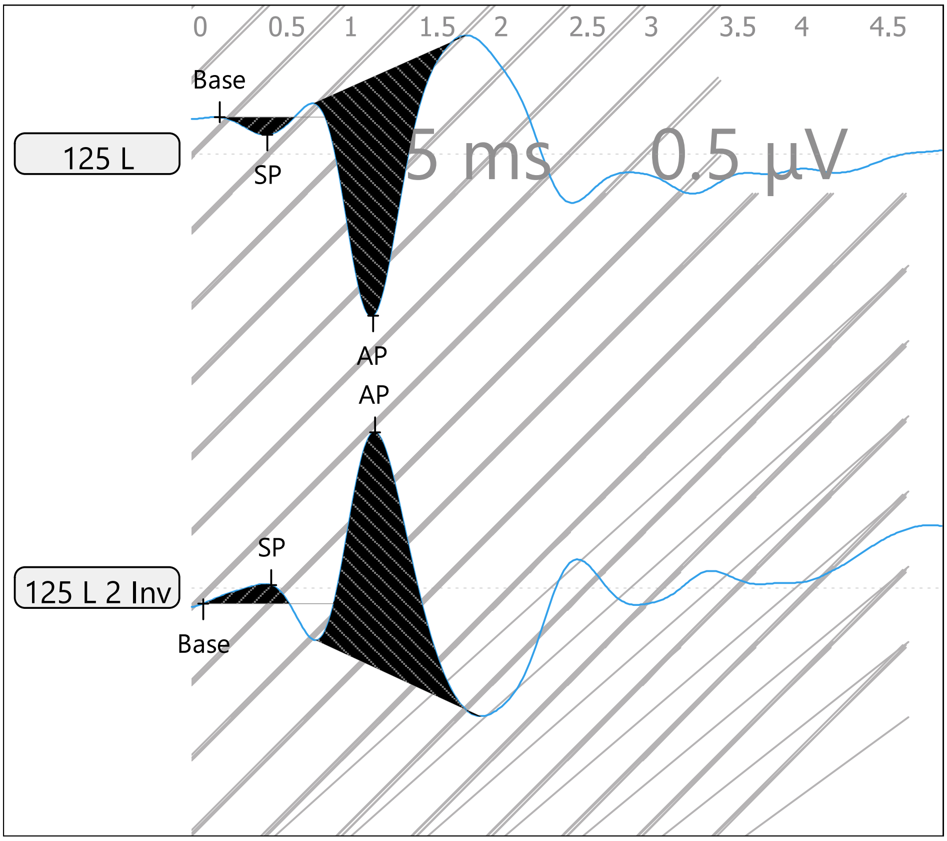

&& (left ear

N	Base (ms)	SP (ms)	AP (ms)	SP–Base (ms)	AP–Base (ms)	SP–Base (µV)	AP–Base (µV)	
125 L	0.19	0.50	1.20	0.32	1.02	0.11	1.32	0
125 L 2 Inv	0.08	0.53	1.22	0.45	1.14	0.12	1.14	0

Trace parameters

N	Electr.	HPF, Hz	LPF, Hz	50 Hz	Rejection ±µV	Aver.	R
125 L	Fpz-M1	5	2000		50	1009	
125 L 2 Inv	Fpz-M1	5	2000		50	1135	

ECochG: ECochG 2:
Fpz-M2

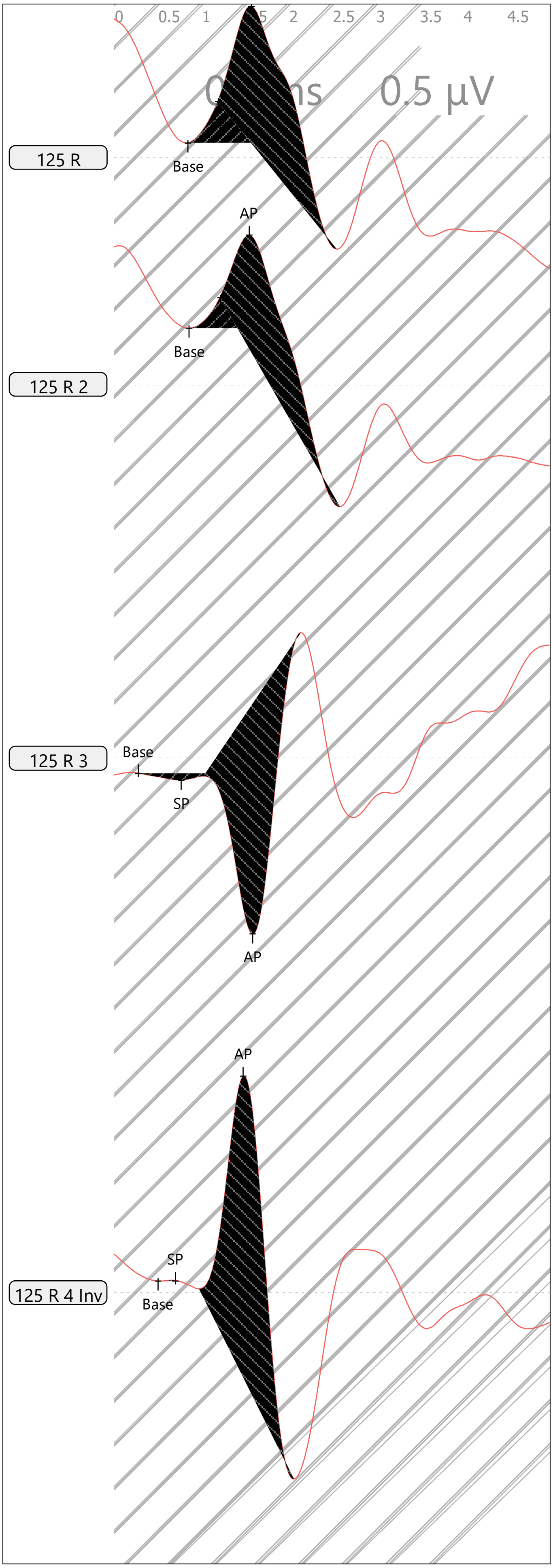
0 0.5 1 1.5 2 2.5 3 3.5 4 4.5
0.5 ms 0.5 µV
125 R
Base
AP
125 R 2
Base
125 R 3
Base
SP
AP
AP
SP
125 R 4 Inv
Base

&&

N	Base (ms)	SP (ms)	AP (ms)	SP–Base (ms)	AP–Base (ms)	SP–Base (µV)	AP–Base (µV)	[illegible]
125 R	0.85	1.19	1.57	0.34	0.73	0.47	1.56	0[illegible]
125 R 2	0.86	1.22	1.55	0.36	0.69	0.35	1.06	0[illegible]
125 R 3	0.28	0.77	1.59	0.49	1.31	0.08	1.84	0[illegible]
125 R 4 Inv	0.50	0.70	1.48	0.20	0.98	0.01	2.35	0[illegible]

Trace parameters

N	Electr.	HPF, Hz	LPF, Hz	50 Hz	Rejection ±µV	Aver.	R[illegible]
125 R	Fpz-M2	5	2000		50	1500	
125 R 2	Fpz-M2	5	2000		50	1500	
125 R 3	Fpz-M2	5	2000		50	1500	
125 R 4 Inv	Fpz-M2	5	2000		50	1500	

CONCLUSION:

Doctor:
