# Supplementary material for: Exposure to high-altitude hypobaric hypoxic environment induces low-frequency hearing loss in C57BL/6J mice: Mediated by slowing down the postsynaptic electrical signal transmission speed in the cochlear-inferior colliculus auditory signaling pathway
Source: PLoS One. 2026 Mar 11;21(3):e0342321. doi: 10.1371/journal.pone.0342321 (PMC12978441; doi:10.1371/journal.pone.0342321)
Supplement: S1 File — (ZIP) [file pone.0342321.s001.zip › 2025-6-11-10d-03.pdf]

Exam report

Patient: 2025-6-11-10d-03- (-)
Date: June 11, 2025

ABR: ABR 2 CLICK
1: Cz-M1

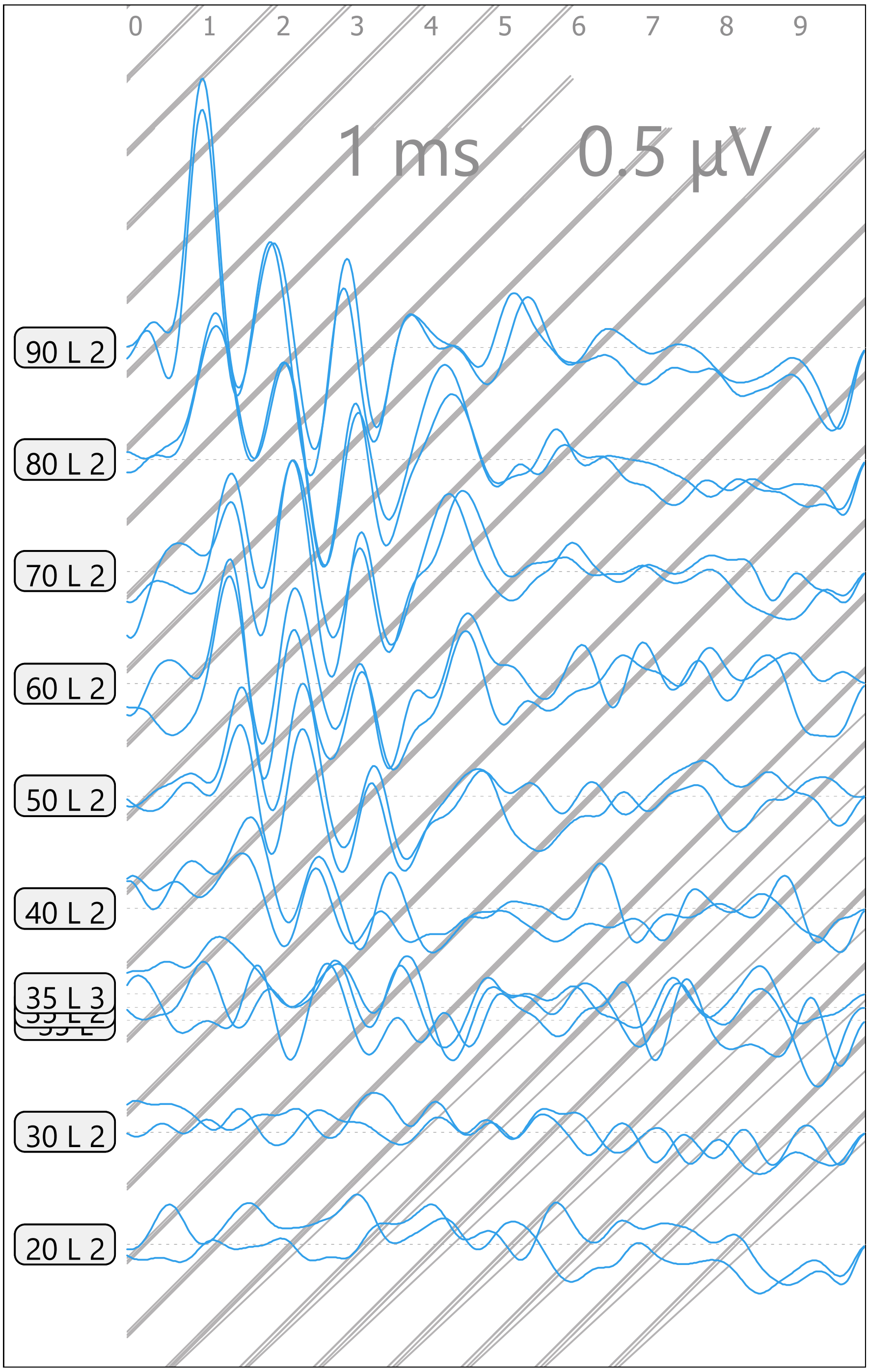

Trace parameters

N	Electr.	HPF, Hz	LPF, Hz	50 Hz	Rejection ±µV	Aver.	Reject.
90 L	Cz-M1	100	2000		10	1000	0
90 L 2	Cz-M1	100	2000		10	1000	0
80 L	Cz-M1	100	2000		10	1000	0
80 L 2	Cz-M1	100	2000		10	1000	0
70 L	Cz-M1	100	2000		10	1000	0
70 L 2	Cz-M1	100	2000		10	1000	0

60 L	Cz-M1	100	2000		10	1000	0
60 L 2	Cz-M1	100	2000		10	1000	0
50 L	Cz-M1	100	2000		10	1000	0
50 L 2	Cz-M1	100	2000		10	1000	0
40 L	Cz-M1	100	2000		10	1000	0
40 L 2	Cz-M1	100	2000		10	1000	0
35 L	Cz-M1	100	2000		10	1000	0
35 L 2	Cz-M1	100	2000		10	1000	0
35 L 3	Cz-M1	100	2000		10	1000	0
30 L	Cz-M1	100	2000		10	1000	0
30 L 2	Cz-M1	100	2000		10	1000	0
20 L	Cz-M1	100	2000		10	1000	0
20 L 2	Cz-M1	100	2000		10	1000	0

ABR: ABR 2 4000Hz 1: Cz-M1

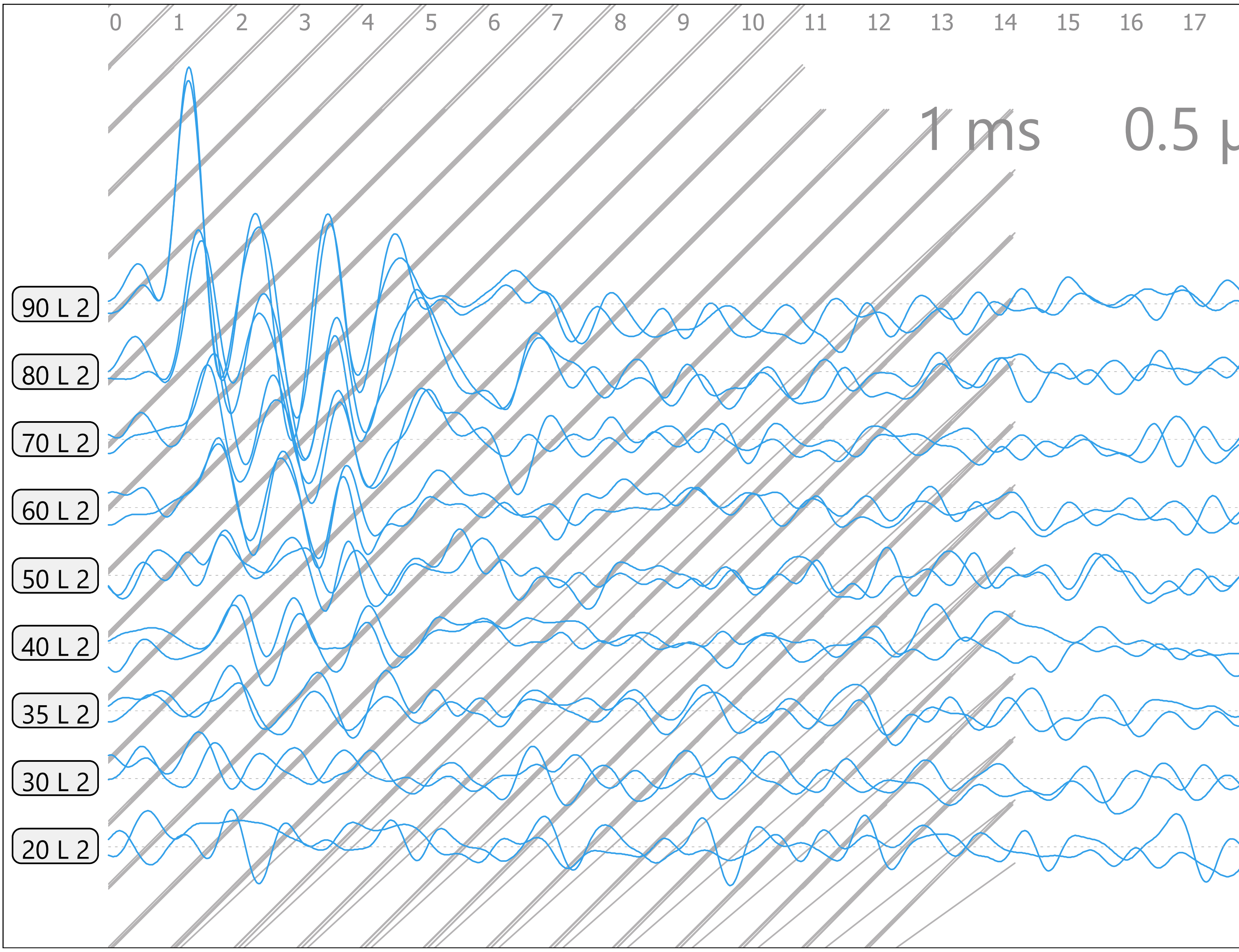

Trace parameters

N	Electr.	HPF, Hz	LPF, Hz	50 Hz	Rejection ±μV	Aver.	Reject.
90 L	Cz-M1	200	2000		10	1000	0
90 L 2	Cz-M1	200	2000		10	1000	0
80 L	Cz-M1	200	2000		10	1000	0

80 L 2	Cz-M1	200	2000		10	1000	0
70 L	Cz-M1	200	2000		10	1000	0
70 L 2	Cz-M1	200	2000		10	1000	0
60 L	Cz-M1	200	2000		10	1000	0
60 L 2	Cz-M1	200	2000		10	1000	0
50 L	Cz-M1	200	2000		10	1000	0
50 L 2	Cz-M1	200	2000		10	1000	0
40 L	Cz-M1	200	2000		10	1000	0
40 L 2	Cz-M1	200	2000		10	1000	0
35 L	Cz-M1	200	2000		10	1000	0
35 L 2	Cz-M1	200	2000		10	1000	0
30 L	Cz-M1	200	2000		10	1000	0
30 L 2	Cz-M1	200	2000		10	1000	0
20 L	Cz-M1	200	2000		10	1000	0
20 L 2	Cz-M1	200	2000		10	1000	0

ABR: ABR 2 8000Hz 1: Cz-M1

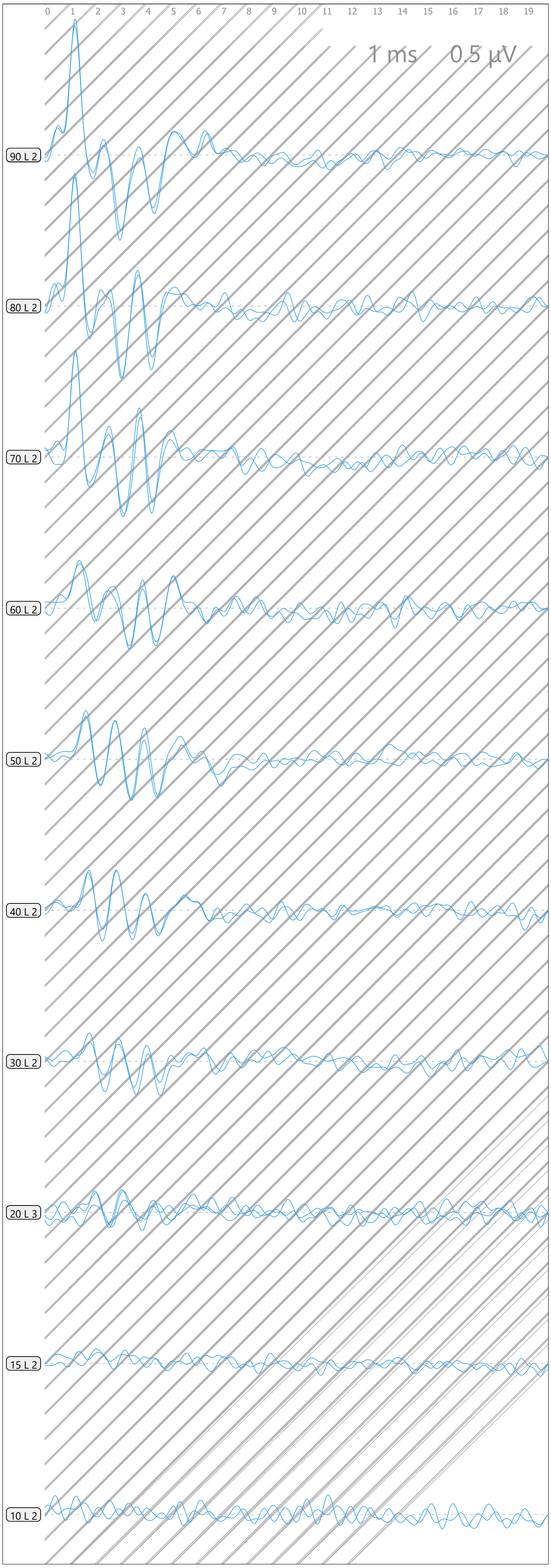

Trace parameters

N	Electr.	HPF, Hz	LPF, Hz	50 Hz	Rejection ±µV	Aver.	Reject.
90 L	Cz-M1	200	2000		10	1000	0
90 L 2	Cz-M1	200	2000		10	1000	0
80 L	Cz-M1	200	2000		10	1000	0
80 L 2	Cz-M1	200	2000		10	1000	0
70 L	Cz-M1	200	2000		10	1000	0
70 L 2	Cz-M1	200	2000		10	1000	0
60 L	Cz-M1	200	2000		10	1000	0
60 L 2	Cz-M1	200	2000		10	1000	0
50 L	Cz-M1	200	2000		10	1000	0
50 L 2	Cz-M1	200	2000		10	1000	0
40 L	Cz-M1	200	2000		10	1000	0
40 L 2	Cz-M1	200	2000		10	1000	0
30 L	Cz-M1	200	2000		10	1000	0
30 L 2	Cz-M1	200	2000		10	1000	0
20 L	Cz-M1	200	2000		10	1004	0
20 L 2	Cz-M1	200	2000		10	1000	0
20 L 3	Cz-M1	200	2000		10	1000	0
15 L	Cz-M1	200	2000		10	1000	0
15 L 2	Cz-M1	200	2000		10	1000	0
10 L	Cz-M1	200	2000		10	1000	0
10 L 2	Cz-M1	200	2000		10	1000	0

ABR: ABR 2 2000Hz
2: Cz-M2

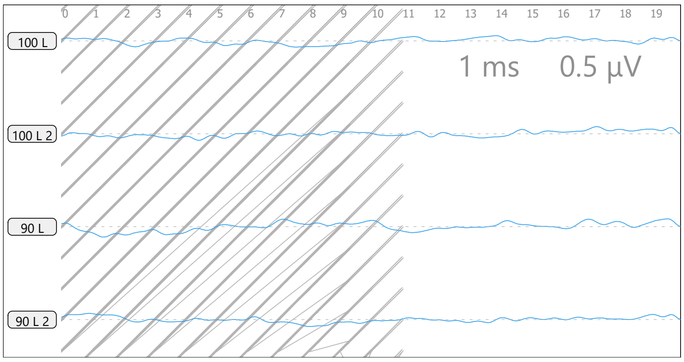

Trace parameters

N	Electr.	HPF, Hz	LPF, Hz	50 Hz	Rejection ±µV	Aver.	Rejec
100 L	Cz-M2	200	2000		10	1000	0
100 L 2	Cz-M2	200	2000		10	1000	0
90 L	Cz-M2	200	2000		10	1000	0

90 L 2	Cz-M2	200	2000		10	1000	0

ABR: ABR 2 4000Hz 2: Cz-M2

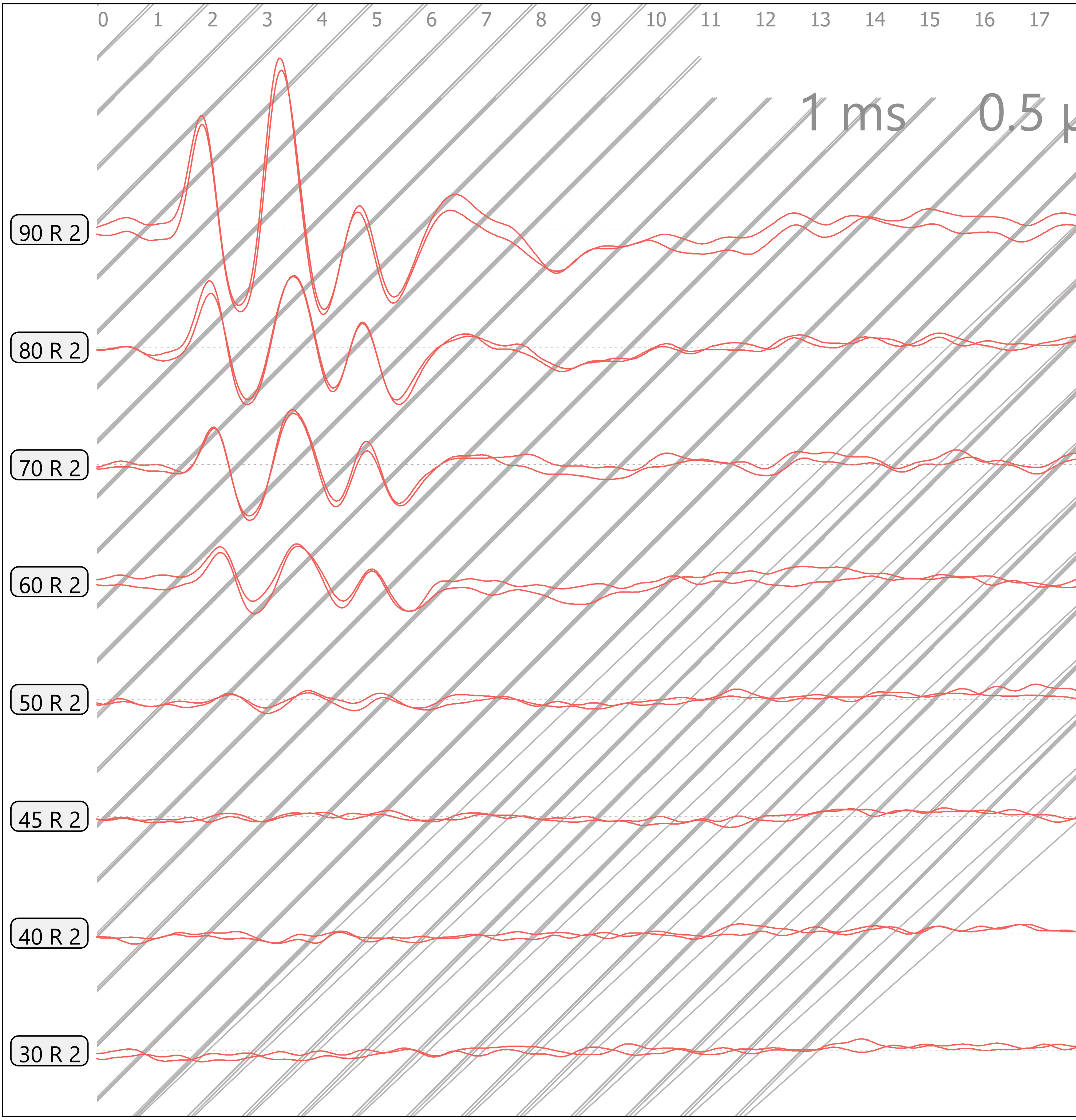

Trace parameters

N	Electr.	HPF, Hz	LPF, Hz	50 Hz	Rejection ±μV	Aver.	Reject
90 R	Cz-M2	200	2000		10	1000	0
90 R 2	Cz-M2	200	2000		10	1000	0
80 R	Cz-M2	200	2000		10	1000	0

80 R 2	Cz-M2	200	2000		10	1000	0
70 R	Cz-M2	200	2000		10	1000	0
70 R 2	Cz-M2	200	2000		10	1000	0
60 R	Cz-M2	200	2000		10	1000	0
60 R 2	Cz-M2	200	2000		10	1000	0
50 R	Cz-M2	200	2000		10	1000	0
50 R 2	Cz-M2	200	2000		10	1000	0
45 R	Cz-M2	200	2000		10	1000	0
45 R 2	Cz-M2	200	2000		10	1000	0
40 R	Cz-M2	200	2000		10	1000	0
40 R 2	Cz-M2	200	2000		10	1000	0
30 R	Cz-M2	200	2000		10	1000	0
30 R 2	Cz-M2	200	2000		10	1000	0

ABR: ABR 2 8000Hz 2: Cz-M2

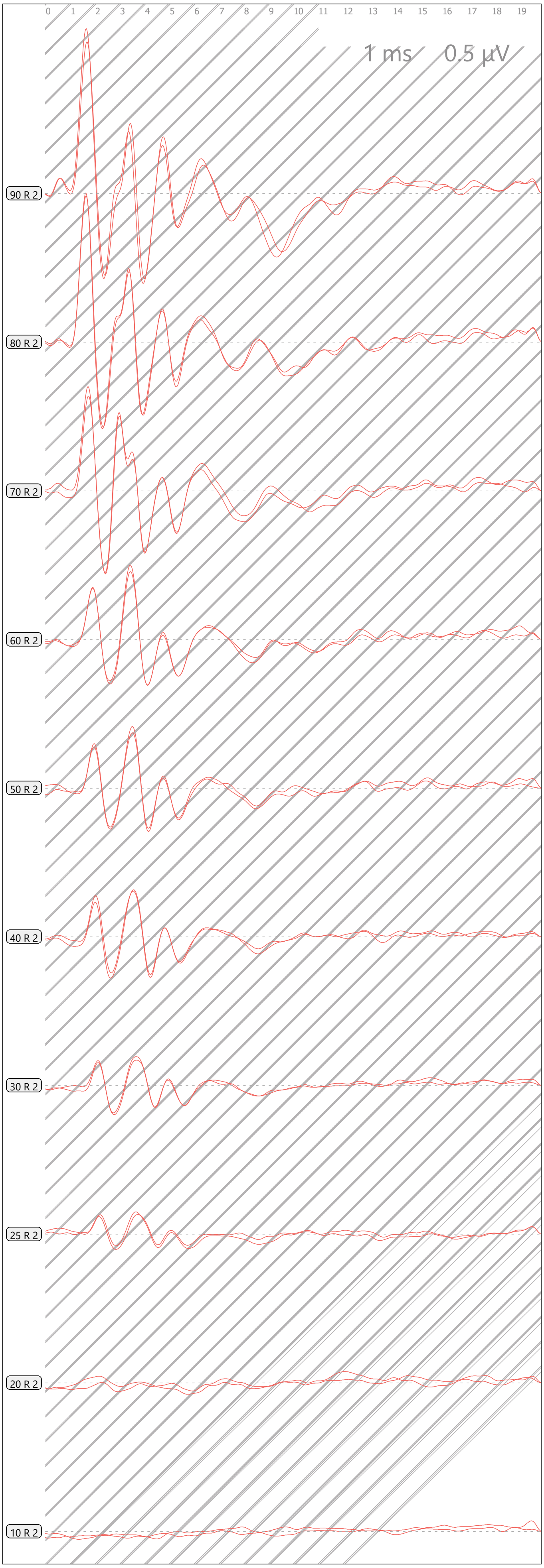

0 1 2 3 4 5 6 7 8 9 10 11 12 13 14 15 16 17 18 19
1 ms
0.5 µV
90 R 2
80 R 2
70 R 2
60 R 2
50 R 2
40 R 2
30 R 2
25 R 2
20 R 2
10 R 2

Trace parameters

N	Electr.	HPF, Hz	LPF, Hz	50 Hz	Rejection ±μV	Aver.	Reject.
90 R	Cz-M2	200	2000		10	1000	0
90 R 2	Cz-M2	200	2000		10	1000	0
80 R	Cz-M2	200	2000		10	1000	0
80 R 2	Cz-M2	200	2000		10	1000	0
70 R	Cz-M2	200	2000		10	1000	0
70 R 2	Cz-M2	200	2000		10	1000	0
60 R	Cz-M2	200	2000		10	1000	0
60 R 2	Cz-M2	200	2000		10	1000	0
50 R	Cz-M2	200	2000		10	1000	0
50 R 2	Cz-M2	200	2000		10	1000	0
40 R	Cz-M2	200	2000		10	1000	0
40 R 2	Cz-M2	200	2000		10	1000	0
30 R	Cz-M2	200	2000		10	1000	0
30 R 2	Cz-M2	200	2000		10	1000	0
25 R	Cz-M2	200	2000		10	1000	0
25 R 2	Cz-M2	200	2000		10	1000	0
20 R	Cz-M2	200	2000		10	1000	0
20 R 2	Cz-M2	200	2000		10	1000	0
10 R	Cz-M2	200	2000		10	1000	0
10 R 2	Cz-M2	200	2000		10	1000	0

ABR: ABR 2 CLICK 2: Fpz-M2

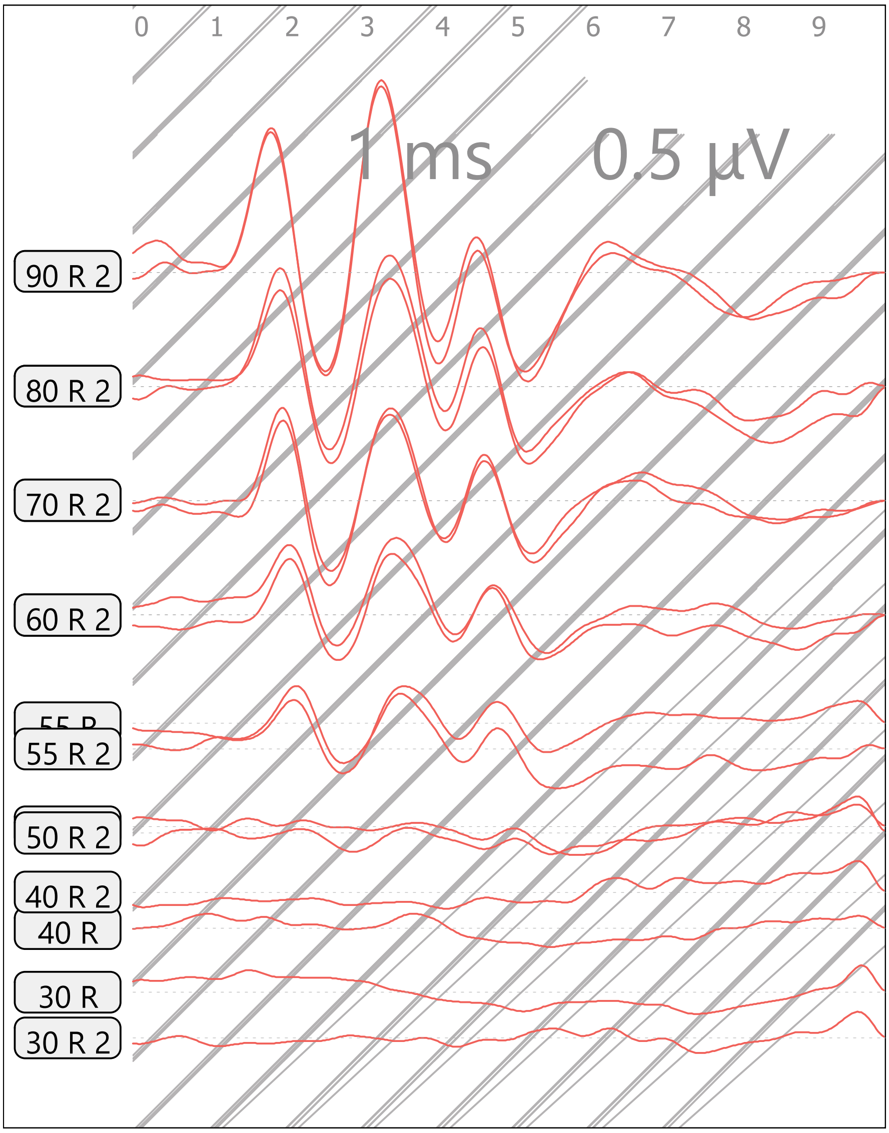

Trace parameters

N	Electr.	HPF, Hz	LPF, Hz	50 Hz	Rejection ±µV	Aver.	Reject.
90 R	Fpz-M2	100	2000		10	1000	0
90 R 2	Fpz-M2	100	2000		10	1000	0
80 R	Fpz-M2	100	2000		10	1000	0
80 R 2	Fpz-M2	100	2000		10	1000	0
70 R	Fpz-M2	100	2000		10	1000	0
70 R 2	Fpz-M2	100	2000		10	1000	0
60 R	Fpz-M2	100	2000		10	1000	0
60 R 2	Fpz-M2	100	2000		10	1000	0
55 R	Fpz-M2	100	2000		10	1000	0
55 R 2	Fpz-M2	100	2000		10	1000	0
50 R	Fpz-M2	100	2000		10	1000	0
50 R 2	Fpz-M2	100	2000		10	1000	0
40 R	Fpz-M2	100	2000		10	1000	0
40 R 2	Fpz-M2	100	2000		10	1000	0
30 R	Fpz-M2	100	2000		10	1000	0
30 R 2	Fpz-M2	100	2000		10	1000	0

DPOAE: 1-12 kHz 70/70 dB 3 points

Test result (right ear):

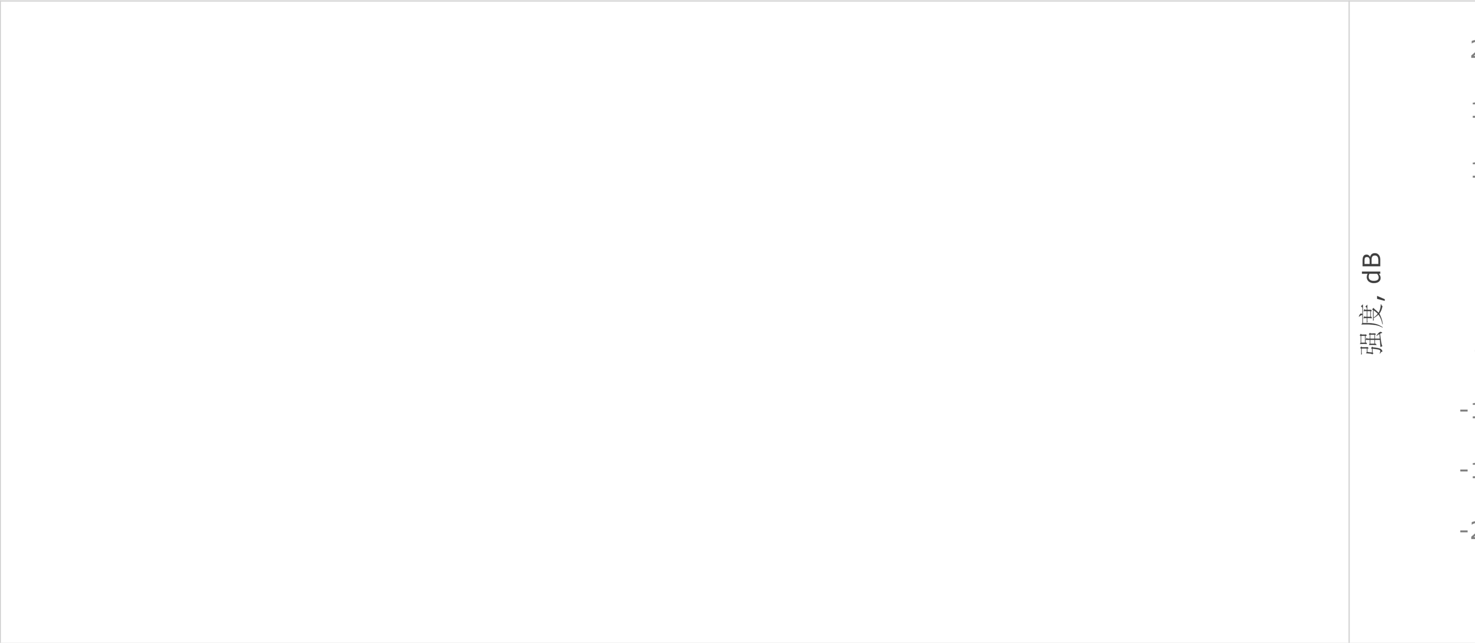

DPOAE

F2, Hz	L1, dB	L2, dB	DP, dB	dB	SNR, dB	OAE
988	68.0	68.4	-0.75	3.63	-4.4	✗
1270	68.9	69.1	-12.57	-4.94	-7.6	✗
1778	69.7	69.7	-10.50	-7.99	-2.5	✗
2222	70.0	70.0	-15.39	-9.40	-6.0	✗
2500	70.1	70.1	-11.04	-15.00	4.0	✗
3200	70.4	70.4	-5.78	-11.85	6.1	✓
4444	70.8	70.9	-22.11	-15.00	-7.1	✗
5000	70.5	70.6	-5.40	-11.81	6.4	✓
6154	70.9	70.4	7.97	-10.83	18.8	✓
8000	69.9	71.2	14.69	-8.08	22.8	✓
8889	69.7	71.6	19.74	1.57	18.2	✓
10000	71.5	60.4	11.74	-3.05	14.8	✓
11429	62.2	53.0	7.59	-6.78	14.4	✓
(dB SPL)	:: 0.0					

ECochG: ECochG 1:
Fpz-M1

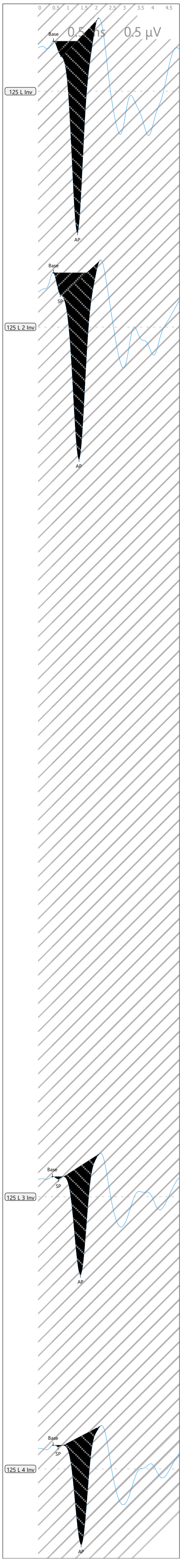

0
0.5
1
1.5
2
2.5
3
3.5
4
4.5
0.5 ms
0.5 µV
Base
125 L Inv
AP
Base
SP
125 L 2 Inv
AP
Base
SP
125 L 3 Inv
AP
Base
SP
125 L 4 Inv
AP

	Base (ms)	SP (ms)	AP (ms)	SP–Base (ms)	AP–Base (ms)	SP–Base (µV)	AP–Base (µV)	
125 L Inv	0.54	0.81	1.39	0.26	0.85	0.53	6.77	[illegible]
125 L 2 Inv	0.54	0.78	1.44	0.24	0.90	0.76	6.59	[illegible]
125 L 3 Inv	0.50	0.71	1.49	0.21	0.99	0.08	3.47	[illegible]
125 L 4 Inv	0.53	0.70	1.52	0.17	0.99	0.05	3.51	[illegible]

Trace parameters

N	Electr.	HPF, Hz	LPF, Hz	50 Hz	Rejection ±µV	Aver.	Re
125 L Inv	Fpz-M1	5	2000		50	1500	
125 L 2 Inv	Fpz-M1	5	2000		50	1500	
125 L 3 Inv	Fpz-M1	5	2000		50	1500	
125 L 4 Inv	Fpz-M1	5	2000		50	1203	

ECochG: ECochG 2:
Fpz-M2

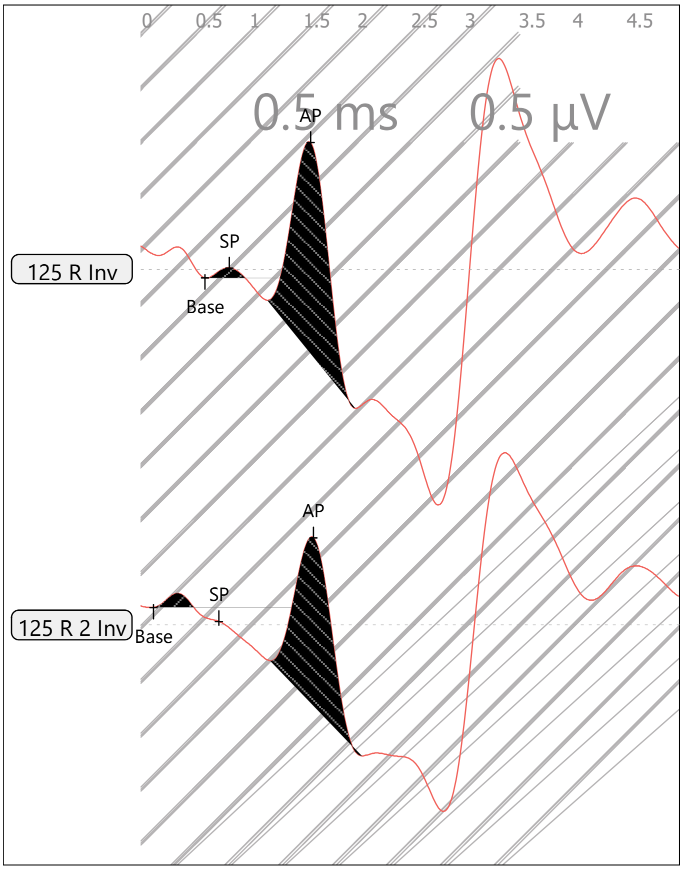

N	Base (ms)	SP (ms)	AP (ms)	SP–Base (ms)	AP–Base (ms)	SP–Base (µV)	AP–Base (µV)	
125 R Inv	0.60	0.82	1.57	0.22	0.98	0.09	1.25	[illegible]
125 R 2 Inv	0.12	0.73	1.60	0.61	1.48	0.13	0.64	[illegible]

Trace parameters

N	Electr.	HPF, Hz	LPF, Hz	50 Hz	Rejection ±µV	Aver.	R
125 R Inv	Fpz-M2	5	2000		50	1500	
125 R 2 Inv	Fpz-M2	5	2000		50	1500	

CONCLUSION:

Doctor:
