# Supplementary material for: Exposure to high-altitude hypobaric hypoxic environment induces low-frequency hearing loss in C57BL/6J mice: Mediated by slowing down the postsynaptic electrical signal transmission speed in the cochlear-inferior colliculus auditory signaling pathway
Source: PLoS One. 2026 Mar 11;21(3):e0342321. doi: 10.1371/journal.pone.0342321 (PMC12978441; doi:10.1371/journal.pone.0342321)
Supplement: S1 File — (ZIP) [file pone.0342321.s001.zip › 2025-6-15-35d-3.pdf]

Exam report

Patient: 2025-6-15-35d-3- (-)
Date: June 16, 2025

ABR: ABR 2 CLICK
1: Cz-M1

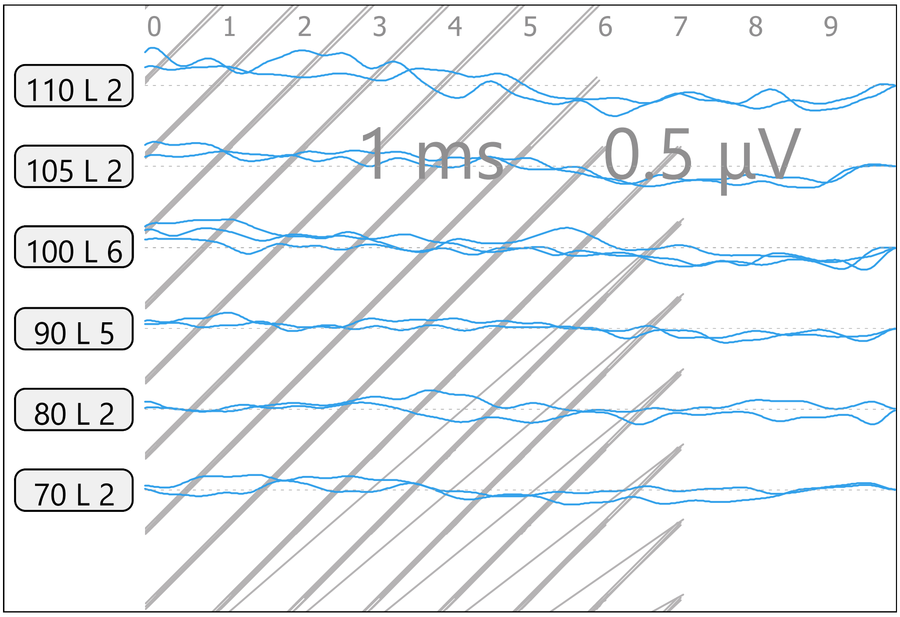

Trace parameters

N	Electr.	HPF, Hz	LPF, Hz	50 Hz	Rejection ±µV	Aver.	Rejec
110 L	Cz-M1	100	2000		10	1000	0
110 L 2	Cz-M1	100	2000		10	259	0
105 L	Cz-M1	100	2000		10	1000	0
105 L 2	Cz-M1	100	2000		10	1000	0
100 L 4	Cz-M1	100	2000		10	1000	0
100 L 5	Cz-M1	100	2000		10	1000	0
100 L 6	Cz-M1	100	2000		10	1000	0
90 L 4	Cz-M1	100	2000		10	1000	0
90 L 5	Cz-M1	100	2000		10	1000	0
80 L	Cz-M1	100	2000		10	1000	0
80 L 2	Cz-M1	100	2000		10	1000	0
70 L	Cz-M1	100	2000		10	1000	0
70 L 2	Cz-M1	100	2000		10	1000	0

ABR: ABR 2 4000Hz 1: Cz-M1

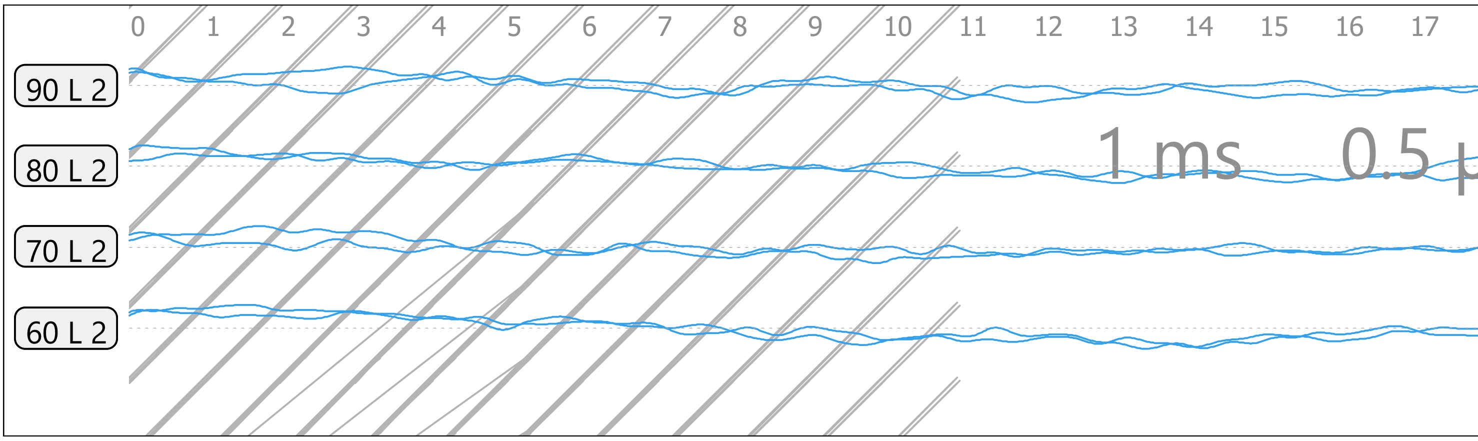

Trace parameters

N	Electr.	HPF, Hz	LPF, Hz	50 Hz	Rejection ±μV	Aver.	Reject.
90 L	Cz-M1	200	2000		10	1000	0
90 L 2	Cz-M1	200	2000		10	1000	0
80 L	Cz-M1	200	2000		10	1000	0
80 L 2	Cz-M1	200	2000		10	1000	0
70 L	Cz-M1	200	2000		10	1000	0
70 L 2	Cz-M1	200	2000		10	1000	0
60 L	Cz-M1	200	2000		10	1000	0
60 L 2	Cz-M1	200	2000		10	1000	0

ABR: ABR 2 8000Hz 1: Cz-M1

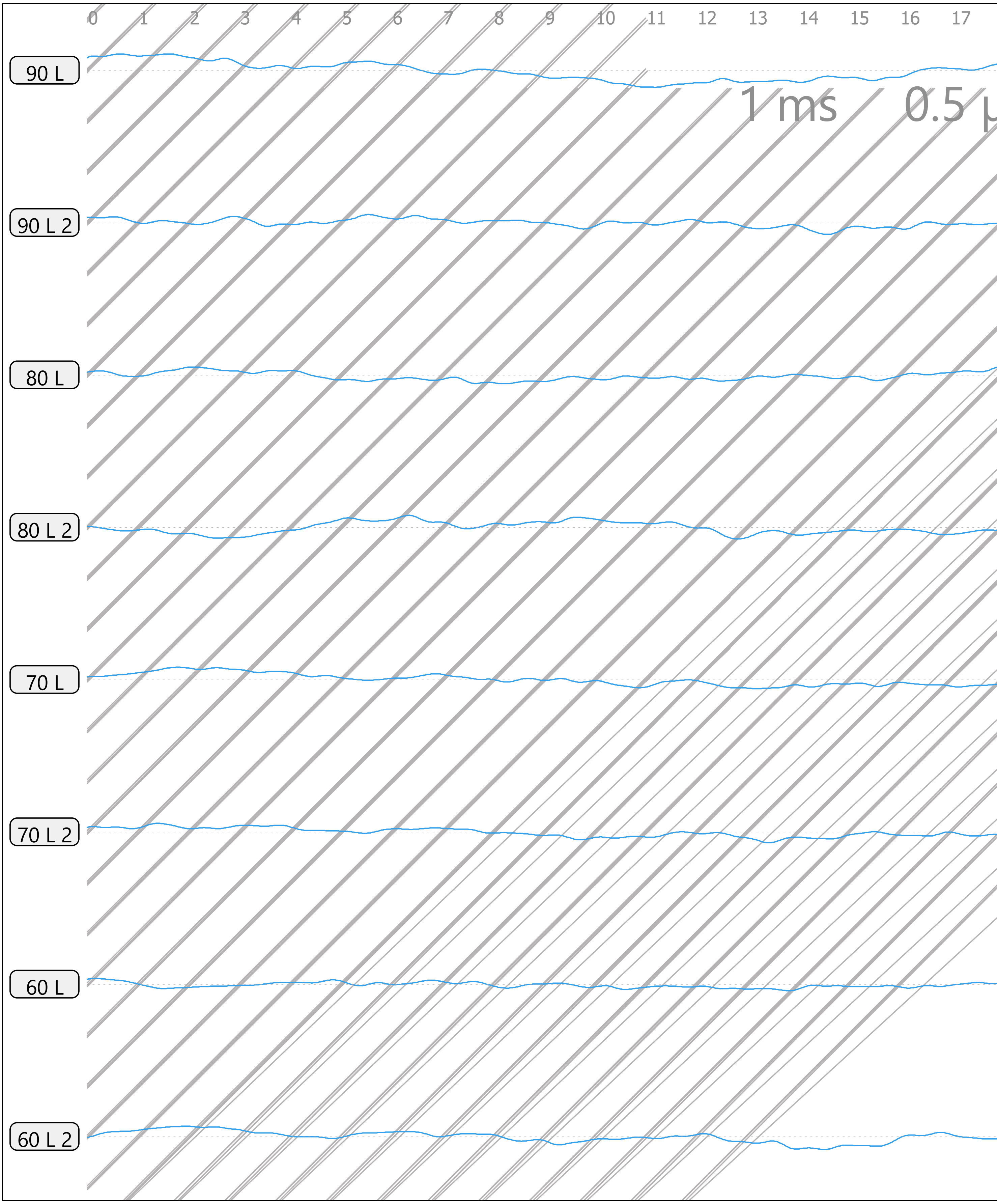

Trace parameters

N	Electr.	HPF, Hz	LPF, Hz	50 Hz	Rejection ±μV	Aver.	Reject.
90 L	Cz-M1	200	2000		10	1000	0
90 L 2	Cz-M1	200	2000		10	1000	0

80 L	Cz-M1	200	2000		10	1000	0
80 L 2	Cz-M1	200	2000		10	1000	0
70 L	Cz-M1	200	2000		10	1000	0
70 L 2	Cz-M1	200	2000		10	1000	0
60 L	Cz-M1	200	2000		10	1000	0
60 L 2	Cz-M1	200	2000		10	1000	0

EcochG: ECochG
1: Fpz-M1

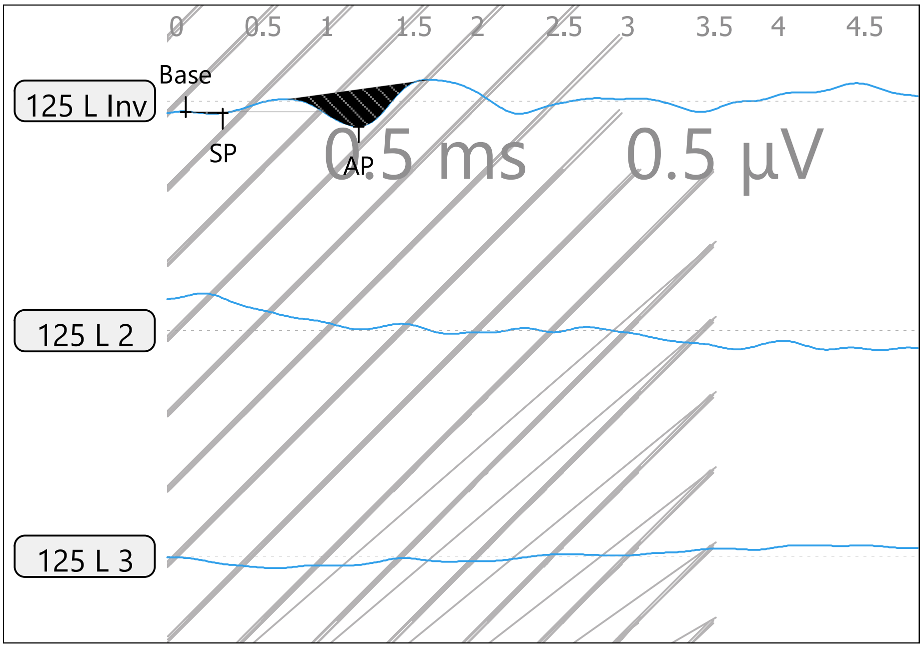

&&

N	Base (ms)	SP (ms)	AP (ms)	SP–Base (ms)	AP–Base (ms)	SP–Base (µV)	AP–Base (µV)	
125 L Inv	0.12	0.37	1.27	0.25	1.15	0.01	0.10	0.09

Trace parameters

N	Electr.	HPF, Hz	LPF, Hz	50 Hz	Rejection ±µV	Aver.	Rej
125 L Inv	Fpz-M1	5	2000		50	1500	4
125 L 2	Fpz-M1	5	2000		50	1500	[illegible]
125 L 3	Fpz-M1	5	2000		50	1070	0

CONCLUSION:

Doctor:
