# Supplementary material for: Exposure to high-altitude hypobaric hypoxic environment induces low-frequency hearing loss in C57BL/6J mice: Mediated by slowing down the postsynaptic electrical signal transmission speed in the cochlear-inferior colliculus auditory signaling pathway
Source: PLoS One. 2026 Mar 11;21(3):e0342321. doi: 10.1371/journal.pone.0342321 (PMC12978441; doi:10.1371/journal.pone.0342321)
Supplement: S1 File — (ZIP) [file pone.0342321.s001.zip › 2025-6-15-7d-2.pdf]

Exam report

Patient: 2025-6-15-7d-2- (-)
Date: June 15, 2025

ABR: ABR 2 CLICK
1: Cz-M1

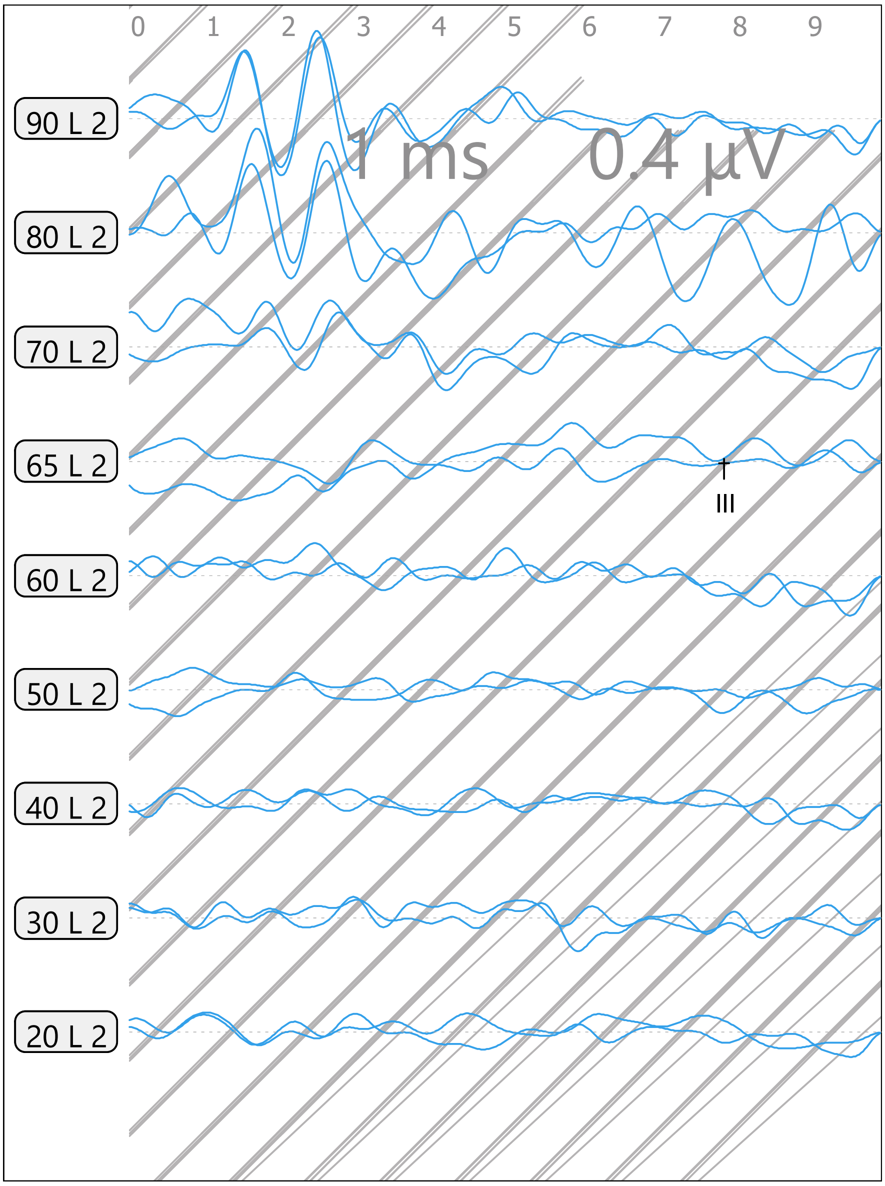

N	I (ms)	II (ms)	III (ms)	IV (ms)	(
65 L			7.91		

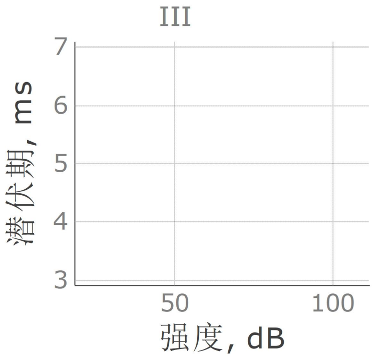

Trace parameters

N	Electr.	HPF, Hz	LPF, Hz	50 Hz	Rejection ±μV	Aver.	Reject.
90 L	Cz-M1	100	2000		10	1000	0
90 L 2	Cz-M1	100	2000		10	1000	0
80 L	Cz-M1	100	2000		10	1000	0
80 L 2	Cz-M1	100	2000		10	1000	0
70 L	Cz-M1	100	2000		10	1000	0
70 L 2	Cz-M1	100	2000		10	1000	0
65 L	Cz-M1	100	2000		10	1000	0
65 L 2	Cz-M1	100	2000		10	1000	0
60 L	Cz-M1	100	2000		10	1000	0
60 L 2	Cz-M1	100	2000		10	1000	0
50 L	Cz-M1	100	2000		10	1000	0
50 L 2	Cz-M1	100	2000		10	1000	0
40 L	Cz-M1	100	2000		10	1000	0
40 L 2	Cz-M1	100	2000		10	1000	0
30 L	Cz-M1	100	2000		10	1000	0
30 L 2	Cz-M1	100	2000		10	1000	0
20 L	Cz-M1	100	2000		10	1000	0
20 L 2	Cz-M1	100	2000		10	1000	0

ABR: ABR 2 4000Hz 1: Cz-M1

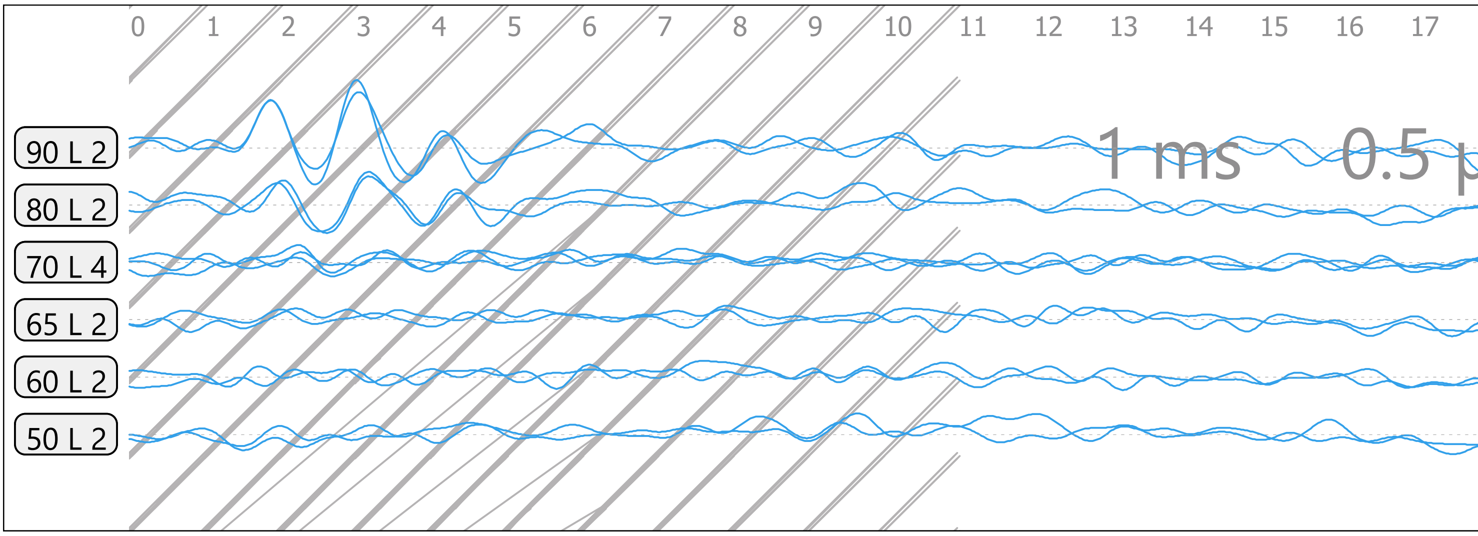

Trace parameters

N	Electr.	HPF, Hz	LPF, Hz	50 Hz	Rejection ±μV	Aver.	Reject.
90 L	Cz-M1	200	2000		10	1000	0
90 L 2	Cz-M1	200	2000		10	1000	0

80 L	Cz-M1	200	2000		10	1000	0
80 L 2	Cz-M1	200	2000		10	1000	0
70 L	Cz-M1	200	2000		10	1000	0
70 L 3	Cz-M1	200	2000		10	1000	0
70 L 4	Cz-M1	200	2000		10	1000	0
65 L	Cz-M1	200	2000		10	1000	0
65 L 2	Cz-M1	200	2000		10	1000	0
60 L	Cz-M1	200	2000		10	1000	0
60 L 2	Cz-M1	200	2000		10	1000	0
50 L	Cz-M1	200	2000		10	1000	0
50 L 2	Cz-M1	200	2000		10	1000	0

ABR: ABR 2 8000Hz 1: Cz-M1

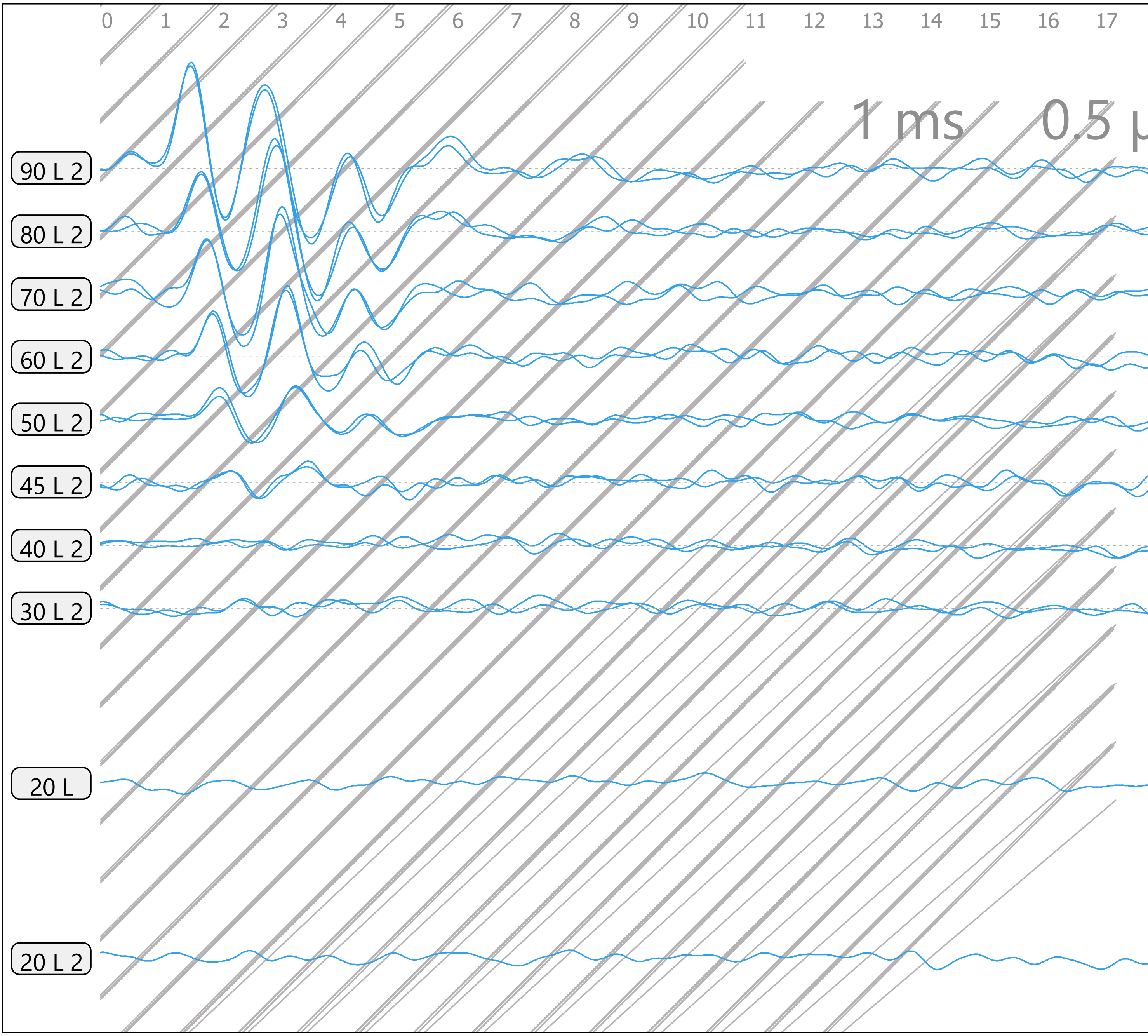

Trace parameters

N	Electr.	HPF, Hz	LPF, Hz	50 Hz	Rejection ±μV	Aver.	Reject.
90 L	Cz-M1	200	2000		10	1000	0
90 L 2	Cz-M1	200	2000		10	1000	0
80 L	Cz-M1	200	2000		10	1000	0
80 L 2	Cz-M1	200	2000		10	1000	0
70 L	Cz-M1	200	2000		10	1000	0
70 L 2	Cz-M1	200	2000		10	1000	0
60 L	Cz-M1	200	2000		10	1000	0
60 L 2	Cz-M1	200	2000		10	1000	0
50 L	Cz-M1	200	2000		10	1000	0
50 L 2	Cz-M1	200	2000		10	1000	0

45 L	Cz-M1	200	2000		10	1000	0
45 L 2	Cz-M1	200	2000		10	1000	0
40 L	Cz-M1	200	2000		10	1000	0
40 L 2	Cz-M1	200	2000		10	1000	0
30 L	Cz-M1	200	2000		10	1000	0
30 L 2	Cz-M1	200	2000		10	1000	0
20 L	Cz-M1	200	2000		10	1000	0
20 L 2	Cz-M1	200	2000		10	1000	0

ABR: ABR 2 CLICK
2: Fpz-M2

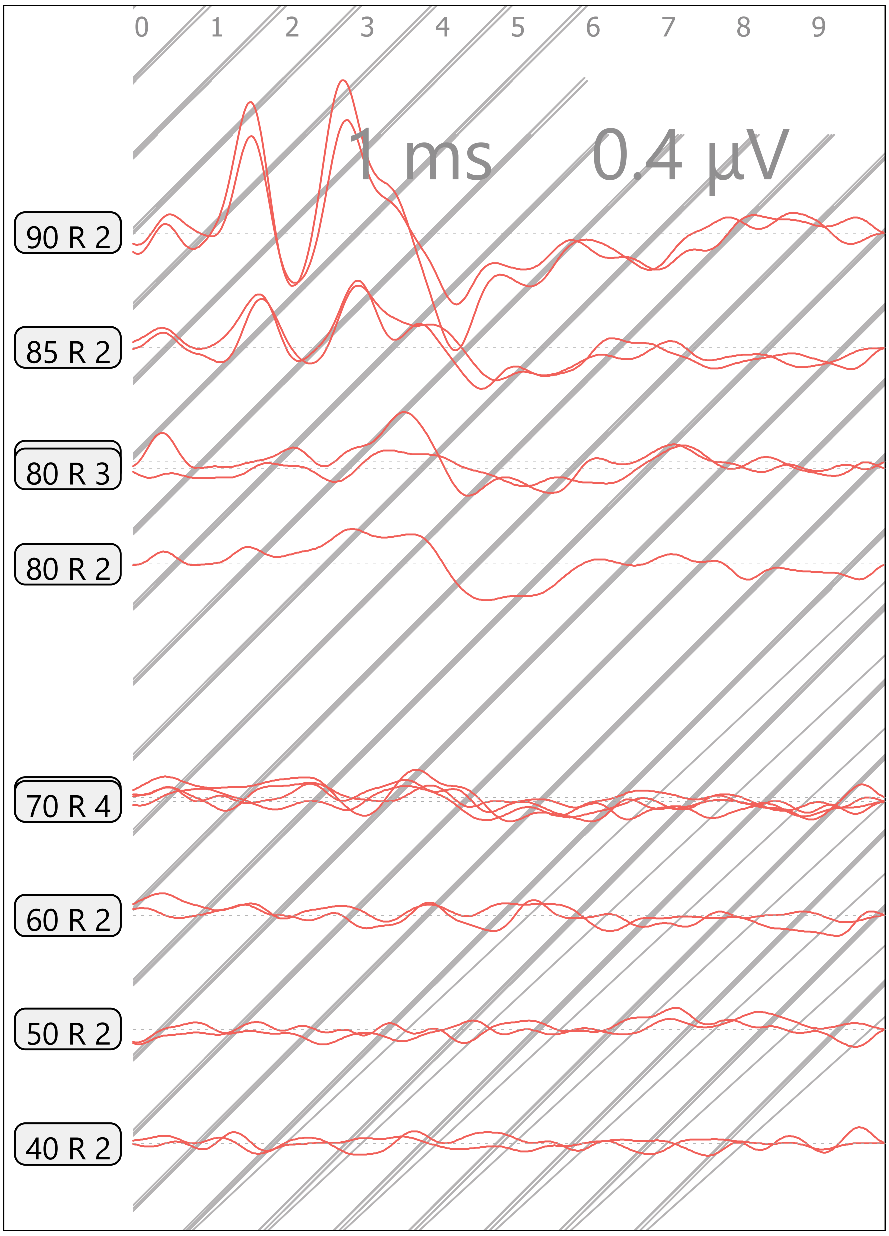

Trace parameters

N	Electr.	HPF, Hz	LPF, Hz	50 Hz	Rejection ±µV	Aver.	Reject
90 R	Fpz-M2	100	2000		10	1000	0

90 R 2	Fpz-M2	100	2000		10	1000	0
85 R	Fpz-M2	100	2000		10	1000	0
85 R 2	Fpz-M2	100	2000		10	1000	0
80 R	Fpz-M2	100	2000		10	1000	0
80 R 2	Fpz-M2	100	2000		10	1000	0
80 R 3	Fpz-M2	100	2000		10	1000	0
70 R	Fpz-M2	100	2000		10	1000	0
70 R 2	Fpz-M2	100	2000		10	1000	0
70 R 3	Fpz-M2	100	2000		10	1000	0
70 R 4	Fpz-M2	100	2000		10	1000	0
60 R	Fpz-M2	100	2000		10	1000	0
60 R 2	Fpz-M2	100	2000		10	1000	0
50 R	Fpz-M2	100	2000		10	1000	0
50 R 2	Fpz-M2	100	2000		10	1000	0
40 R	Fpz-M2	100	2000		10	1000	0
40 R 2	Fpz-M2	100	2000		10	1000	0

ABR: ABR 2 4000Hz 2: Fpz-M2

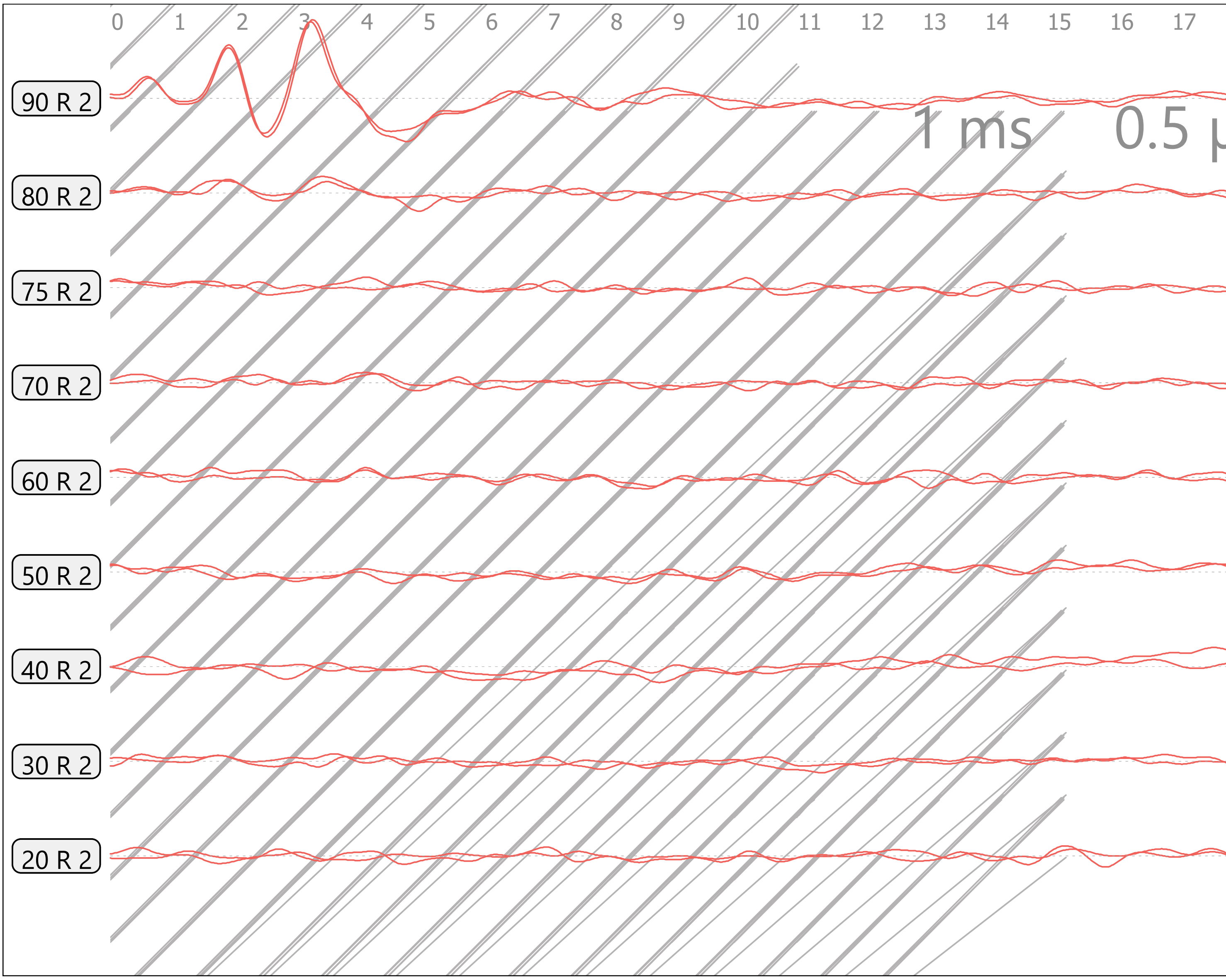

Trace parameters

N	Electr.	HPF, Hz	LPF, Hz	50 Hz	Rejection ±µV	Aver.	Reject
90 R	Fpz-M2	200	2000		10	1000	0

90 R 2	Fpz-M2	200	2000		10	1000	0
80 R	Fpz-M2	200	2000		10	1000	0
80 R 2	Fpz-M2	200	2000		10	1000	0
75 R	Fpz-M2	200	2000		10	1000	0
75 R 2	Fpz-M2	200	2000		10	1000	0
70 R	Fpz-M2	200	2000		10	1000	0
70 R 2	Fpz-M2	200	2000		10	1000	0
60 R	Fpz-M2	200	2000		10	1000	0
60 R 2	Fpz-M2	200	2000		10	1000	0
50 R	Fpz-M2	200	2000		10	1000	0
50 R 2	Fpz-M2	200	2000		10	1000	0
40 R	Fpz-M2	200	2000		10	1000	0
40 R 2	Fpz-M2	200	2000		10	1000	0
30 R	Fpz-M2	200	2000		10	1000	0
30 R 2	Fpz-M2	200	2000		10	1000	0
20 R	Fpz-M2	200	2000		10	1000	0
20 R 2	Fpz-M2	200	2000		10	1000	0

ABR: ABR 2 tone burst 8000Hz 2
: Fpz-M2

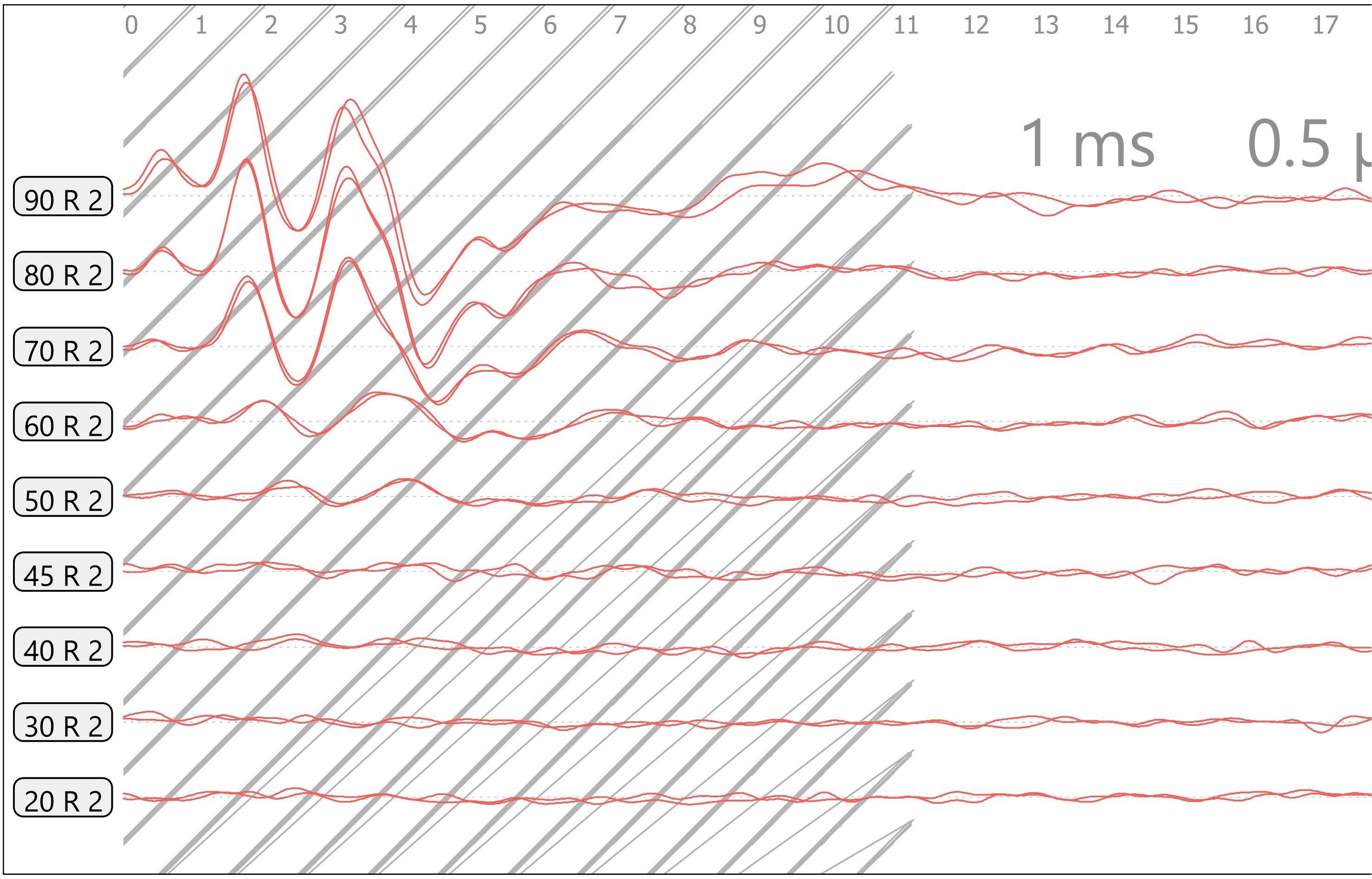

Trace parameters

N	Electr.	HPF, Hz	LPF, Hz	50 Hz	Rejection ±μV	Aver.	Reject
90 R	Fpz-M2	200	2000		10	1000	0
90 R 2	Fpz-M2	200	2000		10	1000	0
80 R	Fpz-M2	200	2000		10	1000	0
80 R 2	Fpz-M2	200	2000		10	1000	0
70 R	Fpz-M2	200	2000		10	1000	0
70 R 2	Fpz-M2	200	2000		10	1000	0
60 R	Fpz-M2	200	2000		10	1000	0
60 R 2	Fpz-M2	200	2000		10	1000	0
50 R	Fpz-M2	200	2000		10	1000	0
50 R 2	Fpz-M2	200	2000		10	1000	0
45 R	Fpz-M2	200	2000		10	1000	0
45 R 2	Fpz-M2	200	2000		10	1000	0
40 R	Fpz-M2	200	2000		10	1000	0
40 R 2	Fpz-M2	200	2000		10	1000	0
30 R	Fpz-M2	200	2000		10	1000	0
30 R 2	Fpz-M2	200	2000		10	1000	0

20 R	Fpz-M2	200	2000		10	1000	0
20 R 2	Fpz-M2	200	2000		10	1000	0

ECochG: ECochG
1: Fpz-M1

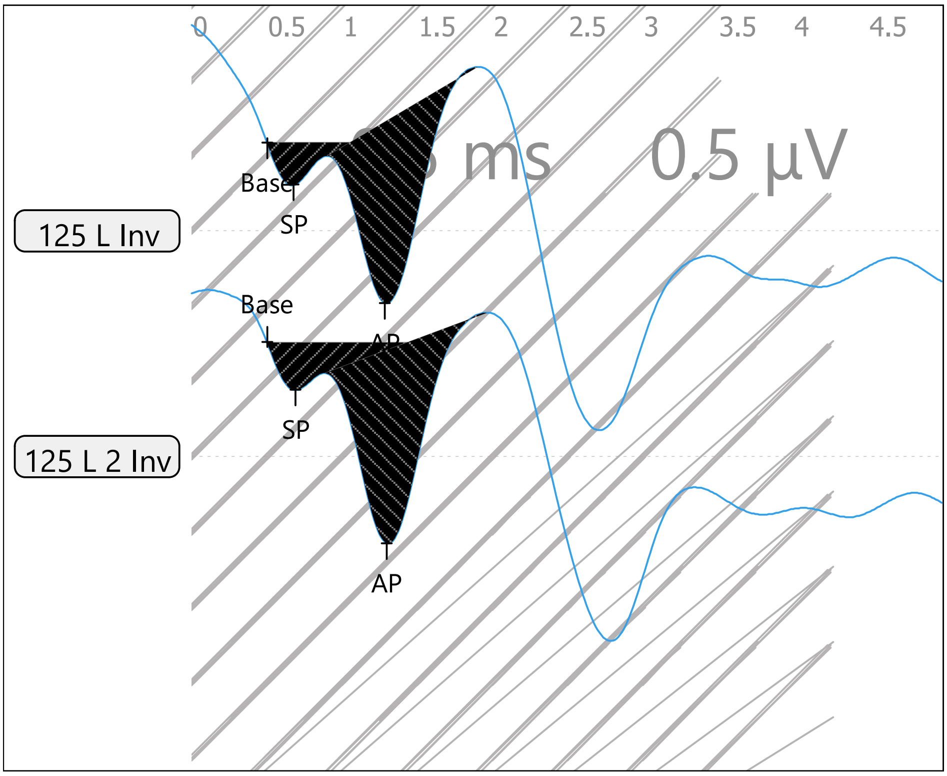

&&

N	Base (ms)	SP (ms)	AP (ms)	SP–Base (ms)	AP–Base (ms)	SP–Base (µV)	AP–Base (µV)	
125 L Inv	0.50	0.67	1.28	0.17	0.78	0.28	1.07	0
125 L 2 Inv	0.50	0.69	1.30	0.19	0.79	0.32	1.34	0

Trace parameters

N	Electr.	HPF, Hz	LPF, Hz	50 Hz	Rejection ±µV	Aver.	R
125 L Inv	Fpz-M1	5	2000		50	1500	
125 L 2 Inv	Fpz-M1	5	2000		50	1500	

ECochG: ECochG 2:
Fpz-M2

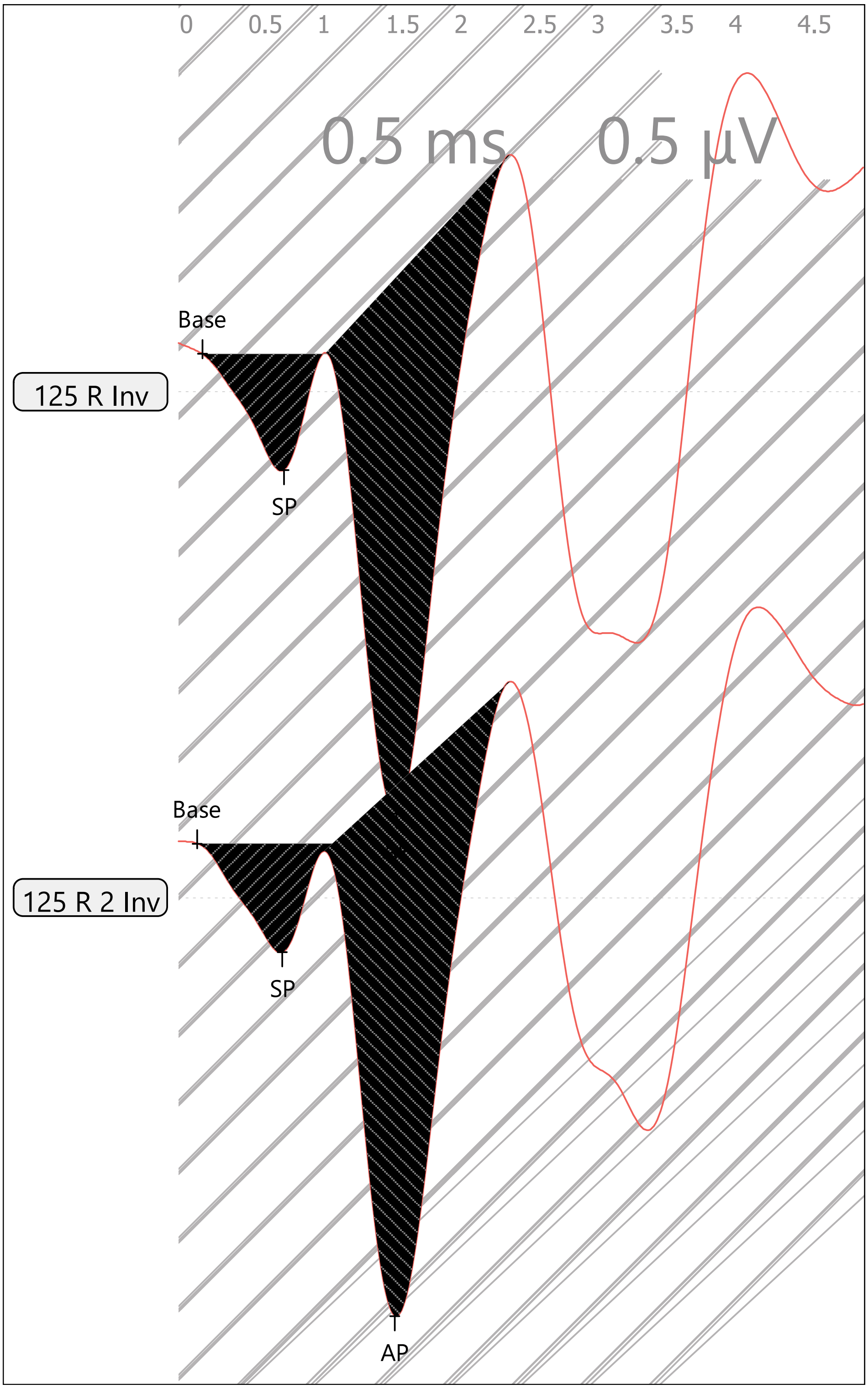

&&

N	Base (ms)	SP (ms)	AP (ms)	SP–Base (ms)	AP–Base (ms)	SP–Base (µV)	AP–Base (µV)	
125 R Inv	0.17	0.77	1.59	0.60	1.42	0.85	3.35	0
125 R 2 Inv	0.13	0.75	1.57	0.62	1.44	0.79	3.44	0

Trace parameters

N	Electr.	HPF, Hz	LPF, Hz	50 Hz	Rejection ±µV	Aver.	R
125 R Inv	Fpz-M2	5	2000		50	1500	
125 R 2 Inv	Fpz-M2	5	2000		50	1500	

CONCLUSION:

Doctor:
