# Supplementary material for: Exposure to high-altitude hypobaric hypoxic environment induces low-frequency hearing loss in C57BL/6J mice: Mediated by slowing down the postsynaptic electrical signal transmission speed in the cochlear-inferior colliculus auditory signaling pathway
Source: PLoS One. 2026 Mar 11;21(3):e0342321. doi: 10.1371/journal.pone.0342321 (PMC12978441; doi:10.1371/journal.pone.0342321)
Supplement: S1 File — (ZIP) [file pone.0342321.s001.zip › 2025-6-15-7d-3.pdf]

Exam report

Patient: 2025-6-15-7d-3, - (-)
Date: June 15, 2025

ABR: ABR 2 CLICK 1: Cz-M1

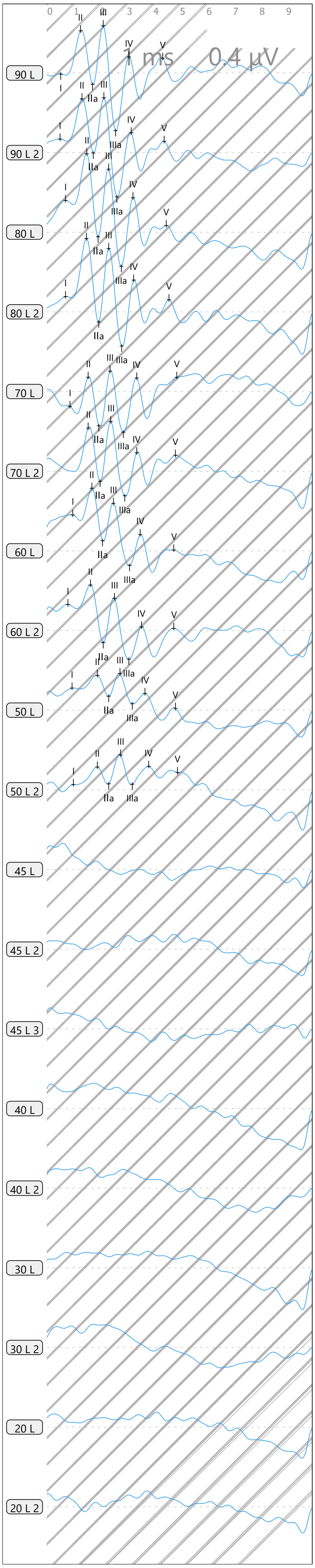

0
1
2
3
4
5
6
7
8
9
1 ms
0.4 µV
90 L
90 L 2
80 L
80 L 2
70 L
70 L 2
60 L
60 L 2
50 L
50 L 2
45 L
45 L 2
45 L 3
40 L
40 L 2
30 L
30 L 2
20 L
20 L 2
I
II
IIa
III
IIIa
IV
V

&&

N	I (ms)	II (ms)	III (ms)	IV (ms)	V (ms)
90 L	0.53	1.27	2.12	3.10	4.37
90 L 2	0.50	1.32	2.14	3.18	4.42
80 L	0.71	1.51	2.33	3.25	4.50
80 L 2	0.71	1.48	2.33	3.28	4.60
70 L	0.87	1.56	2.38	3.39	4.89
70 L 2		1.56	2.41	3.39	4.84
60 L	0.98	1.69	2.51	3.52	4.79
60 L 2	0.79	1.64	2.54	3.57	4.79
50 L	0.95	1.91	2.75	3.70	4.84
50 L 2	1.01	1.91	2.78	3.84	4.92

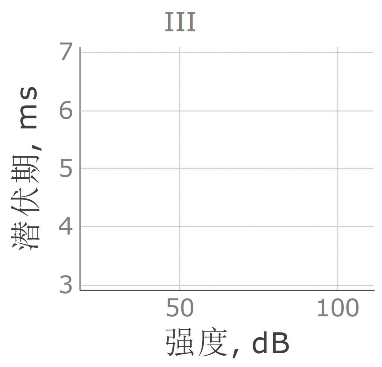

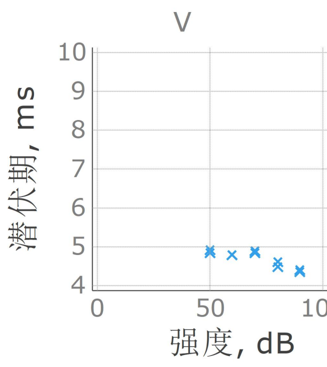

Trace parameters

N	Electr.	HPF, Hz	LPF, Hz	50 Hz	Rejection ±μV	Aver.	Reject.
90 L	Cz-M1	100	2000		10	1000	0
90 L 2	Cz-M1	100	2000		10	1000	0
80 L	Cz-M1	100	2000		10	1000	0
80 L 2	Cz-M1	100	2000		10	1000	0
70 L	Cz-M1	100	2000		10	1000	0
70 L 2	Cz-M1	100	2000		10	1000	0
60 L	Cz-M1	100	2000		10	1000	0
60 L 2	Cz-M1	100	2000		10	1000	0
50 L	Cz-M1	100	2000		10	1000	0
50 L 2	Cz-M1	100	2000		10	1000	0
45 L	Cz-M1	100	2000		10	1000	0
45 L 2	Cz-M1	100	2000		10	1000	0
45 L 3	Cz-M1	100	2000		10	1000	0
40 L	Cz-M1	100	2000		10	1000	0
40 L 2	Cz-M1	100	2000		10	1000	0
30 L	Cz-M1	100	2000		10	1000	0
30 L 2	Cz-M1	100	2000		10	1000	0
20 L	Cz-M1	100	2000		10	1000	0
20 L 2	Cz-M1	100	2000		10	1000	0

ABR: ABR 2 4000Hz 1: Cz-M1

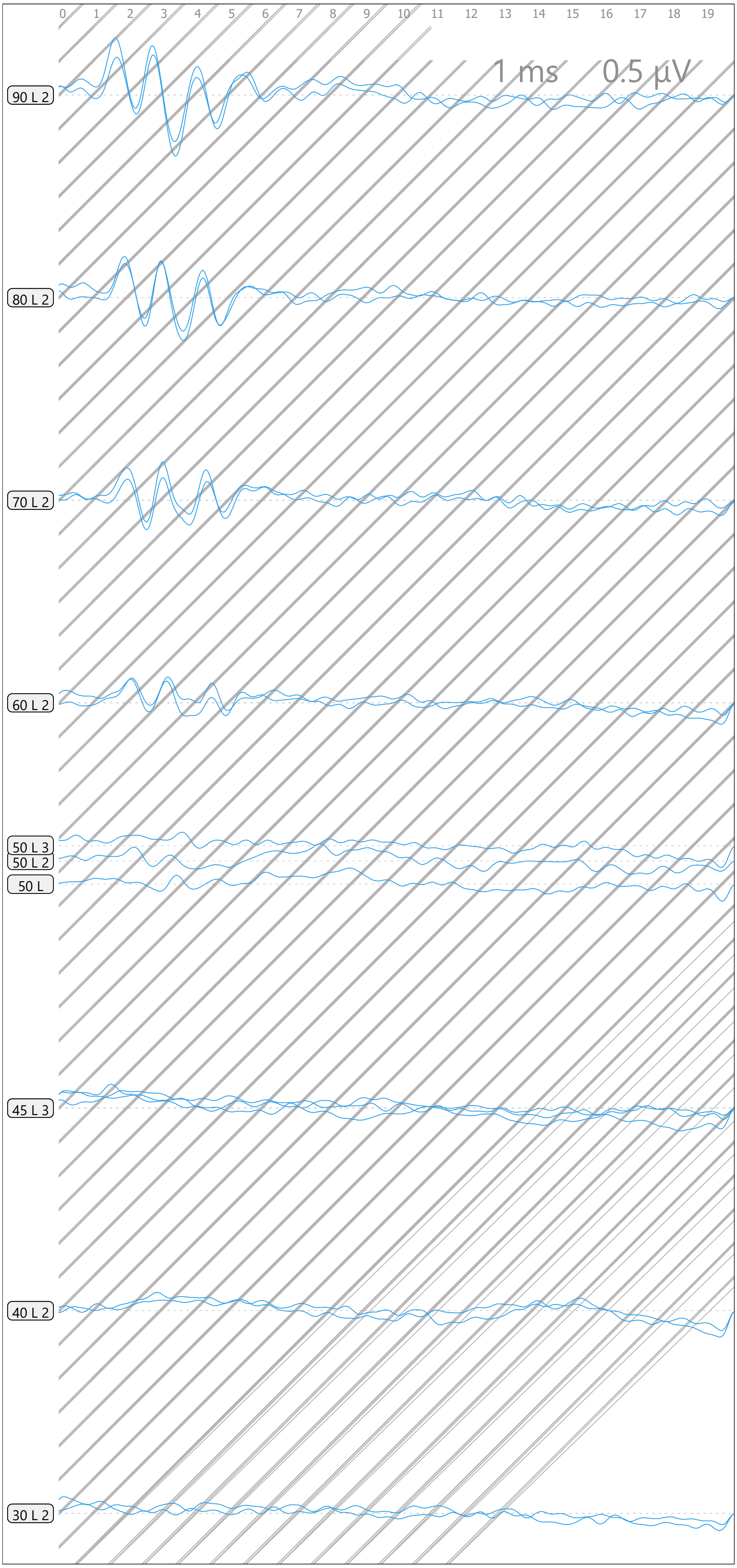

0 1 2 3 4 5 6 7 8 9 10 11 12 13 14 15 16 17 18 19
1 ms
0.5 µV
90 L 2
80 L 2
70 L 2
60 L 2
50 L 3
50 L 2
50 L
45 L 3
40 L 2
30 L 2

Trace parameters

N	Electr.	HPF, Hz	LPF, Hz	50 Hz	Rejection ±μV	Aver.	Reject.
90 L	Cz-M1	200	2000		10	1000	0
90 L 2	Cz-M1	200	2000		10	1000	0
80 L	Cz-M1	200	2000		10	1000	0
80 L 2	Cz-M1	200	2000		10	1000	0
70 L	Cz-M1	200	2000		10	1000	0
70 L 2	Cz-M1	200	2000		10	1000	0
60 L	Cz-M1	200	2000		10	1000	0
60 L 2	Cz-M1	200	2000		10	1000	0
50 L	Cz-M1	200	2000		10	1000	0
50 L 2	Cz-M1	200	2000		10	1000	0
50 L 3	Cz-M1	200	2000		10	1000	0
45 L	Cz-M1	200	2000		10	1000	0
45 L 2	Cz-M1	200	2000		10	1000	0
45 L 3	Cz-M1	200	2000		10	1000	0
40 L	Cz-M1	200	2000		10	1000	0
40 L 2	Cz-M1	200	2000		10	1000	0
30 L	Cz-M1	200	2000		10	1000	0
30 L 2	Cz-M1	200	2000		10	1000	0

ABR: ABR 2 8000Hz 1: Cz-M1

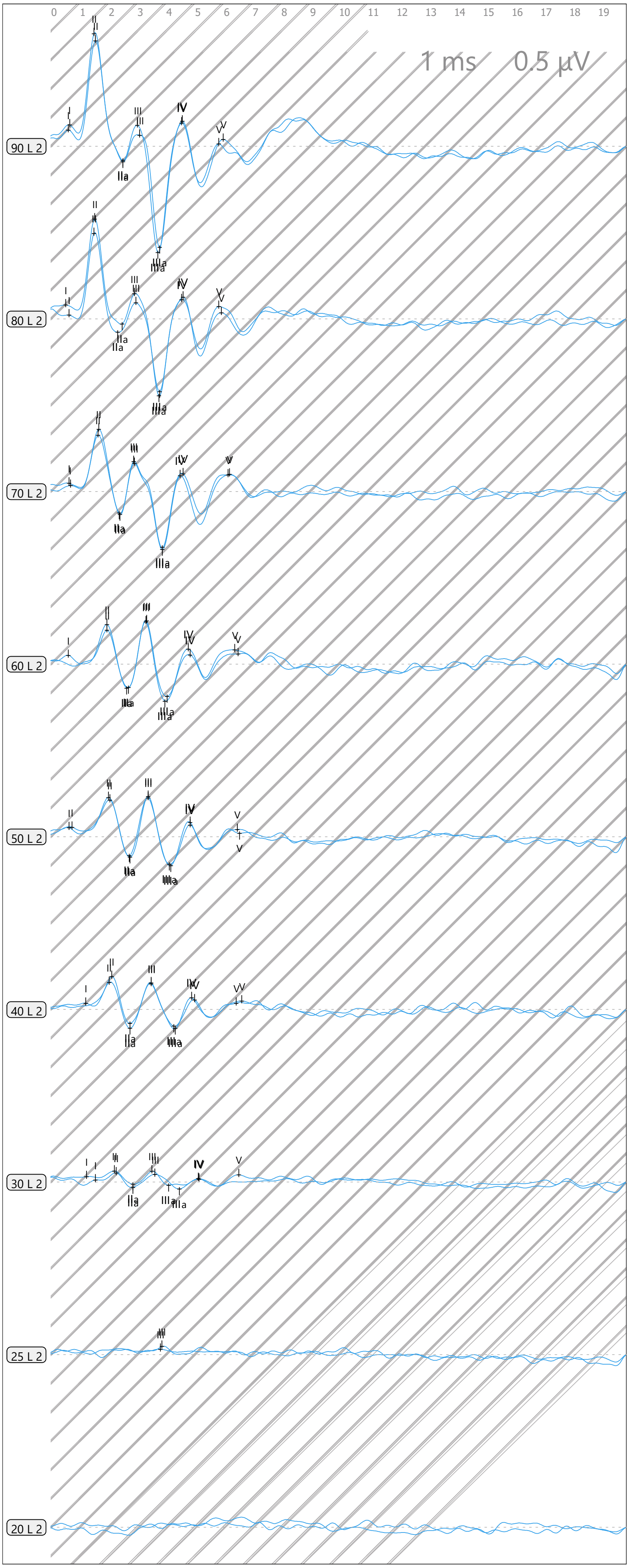
0 1 2 3 4 5 6 7 8 9 10 11 12 13 14 15 16 17 18 19
1 ms
0.5 µV
90 L 2
80 L 2
70 L 2
60 L 2
50 L 2
40 L 2
30 L 2
25 L 2
20 L 2
I
II
IIa
III
IIIa
IV
V

&&

N	I (ms)	II (ms)	III (ms)	Ⅳ (ms)	V (ms)
90 L	0.66	1.51	3.02	4.55	5.85
90 L 2	0.61	1.56	3.10	4.58	6.01
80 L	0.53	1.53	2.91	4.60	5.93
80 L 2	0.64	1.51	2.96	4.55	5.85
70 L	0.64	1.67	2.91	4.50	6.22
70 L 2	0.69	1.64	2.88	4.60	6.16
60 L		1.96	3.33	4.79	6.40
60 L 2	0.61	1.96	3.31	4.84	6.51
50 L	0.74	2.01	3.39	4.84	6.56
50 L 2	0.64	2.06	3.39	4.84	6.48
40 L		2.12	3.49	4.89	6.46
40 L 2	1.22	2.04	3.49	5.00	6.64
30 L	1.24	2.22	3.52	5.13	
30 L 2	1.56	2.28	3.62	5.16	6.54
25 L			3.81		
25 L 2			3.86		

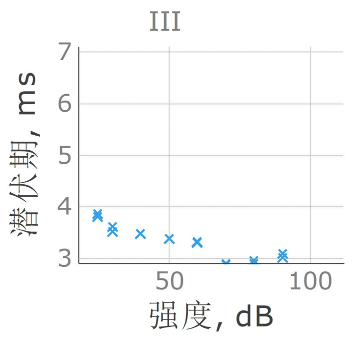

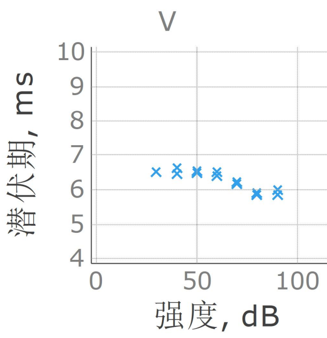

Trace parameters

N	Electr.	HPF, Hz	LPF, Hz	50 Hz	Rejection ±μV	Aver.	Reject.
90 L	Cz-M1	200	2000		10	1000	0
90 L 2	Cz-M1	200	2000		10	1000	0
80 L	Cz-M1	200	2000		10	1000	0
80 L 2	Cz-M1	200	2000		10	1000	0
70 L	Cz-M1	200	2000		10	1000	0
70 L 2	Cz-M1	200	2000		10	1000	0
60 L	Cz-M1	200	2000		10	1000	0
60 L 2	Cz-M1	200	2000		10	1000	0
50 L	Cz-M1	200	2000		10	1000	0
50 L 2	Cz-M1	200	2000		10	1000	0
40 L	Cz-M1	200	2000		10	1000	0
40 L 2	Cz-M1	200	2000		10	1000	0
30 L	Cz-M1	200	2000		10	1000	0
30 L 2	Cz-M1	200	2000		10	1000	0

25 L	Cz-M1	200	2000		10	1000	0
25 L 2	Cz-M1	200	2000		10	1000	0
20 L	Cz-M1	200	2000		10	1000	0
20 L 2	Cz-M1	200	2000		10	1000	0

ABR: ABR 2 CLICK 2: Cz-M2

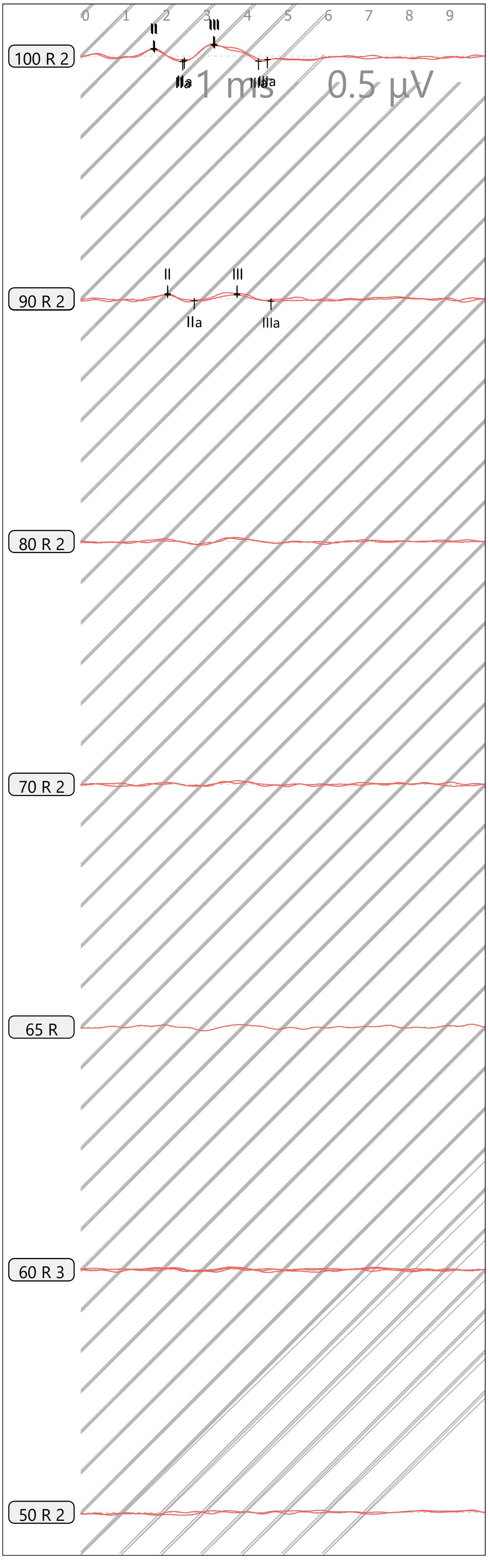

0 1 2 3 4 5 6 7 8 9
1 ms
0.5 µV
100 R 2
II
III
IIa
IIIa
90 R 2
II
III
IIa
IIIa
80 R 2
70 R 2
65 R
60 R 3
50 R 2

	IV (ms)	V (ms)	I–III (ms)	I–V (ms)	III–V (ms)	

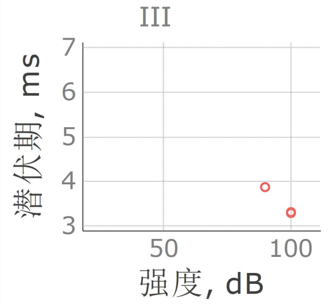

Trace parameters

N	Electr.	HPF, Hz	LPF, Hz	50 Hz	Rejection ±μV	Aver.	Rejec
100 R	Cz-M2	100	2000		10	1000	33
100 R 2	Cz-M2	100	2000		10	1000	47
90 R	Cz-M2	100	2000		10	1000	91
90 R 2	Cz-M2	100	2000		10	1000	48
80 R	Cz-M2	100	2000		10	1000	109
80 R 2	Cz-M2	100	2000		10	1000	63
70 R	Cz-M2	100	2000		10	1000	44
70 R 2	Cz-M2	100	2000		10	1000	43
65 R	Cz-M2	100	2000		10	1000	37
60 R	Cz-M2	100	2000		10	1000	49
60 R 2	Cz-M2	100	2000		10	1000	47
60 R 3	Cz-M2	100	2000		10	1000	43
50 R	Cz-M2	100	2000		10	1000	37
50 R 2	Cz-M2	100	2000		10	1000	18

ABR: ABR 2 4000Hz 2: Cz-M2

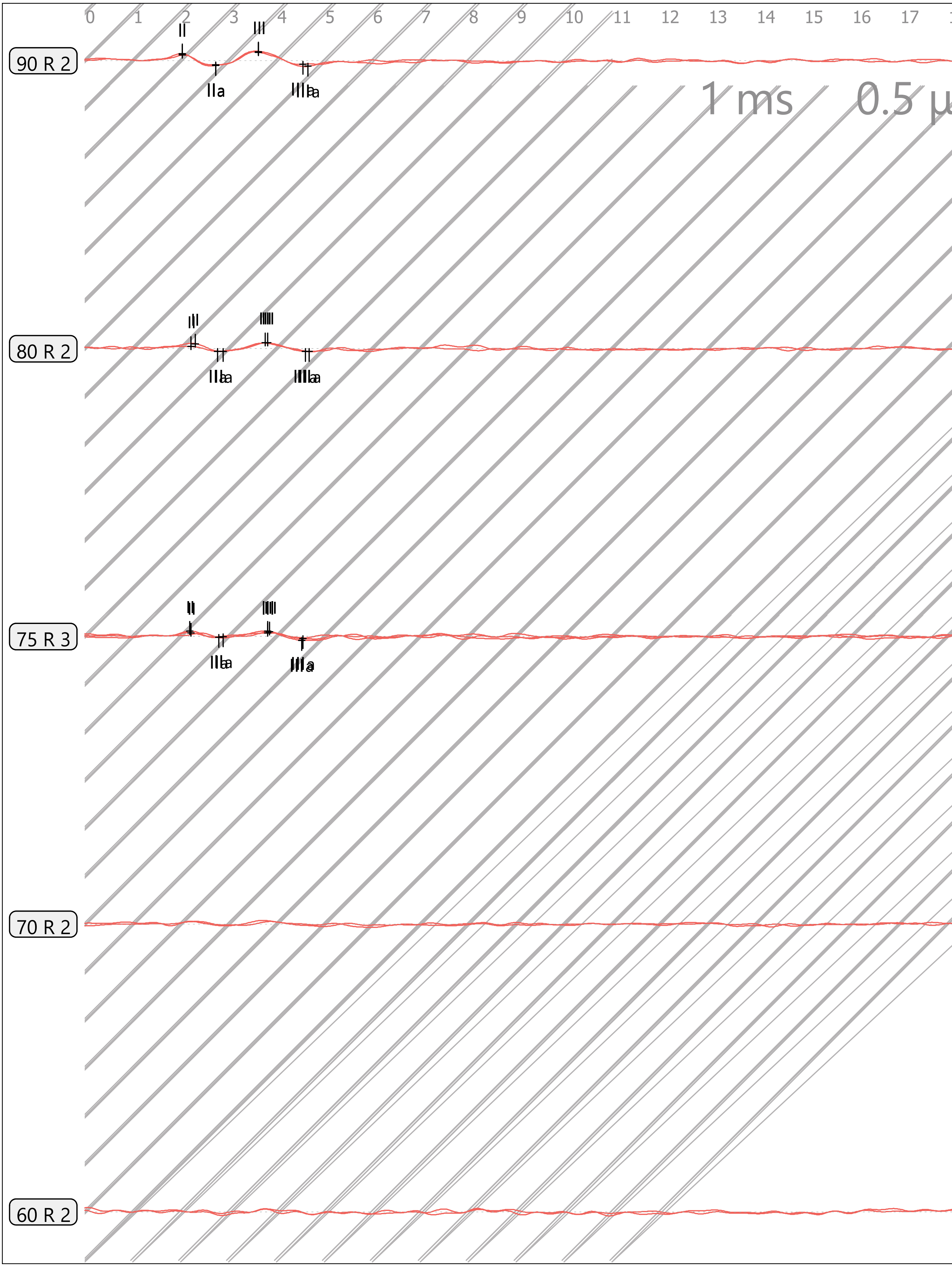

0 1 2 3 4 5 6 7 8 9 10 11 12 13 14 15 16 17
1 ms
0.5 µ
90 R 2
II
IIa
III
IIIa
80 R 2
IIa
IIIa
75 R 3
IIa
IIIa
70 R 2
60 R 2

IV (ms)	V (ms)	I–III (ms)	I–V (ms)	III–V (ms)	

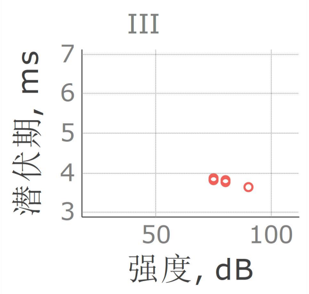

Trace parameters

N	Electr.	HPF, Hz	LPF, Hz	50 Hz	Rejection ±μV	Aver.	Reject
90 R	Cz-M2	200	2000		10	1000	2
90 R 2	Cz-M2	200	2000		10	1000	0
80 R	Cz-M2	200	2000		10	1000	2
80 R 2	Cz-M2	200	2000		10	1000	4
75 R	Cz-M2	200	2000		10	1000	5
75 R 2	Cz-M2	200	2000		10	1000	2
75 R 3	Cz-M2	200	2000		10	1000	3
70 R	Cz-M2	200	2000		10	1000	3
70 R 2	Cz-M2	200	2000		10	1000	2
60 R	Cz-M2	200	2000		10	1000	10
60 R 2	Cz-M2	200	2000		10	1000	5

ABR: ABR 2 8000Hz 2: Cz-M2

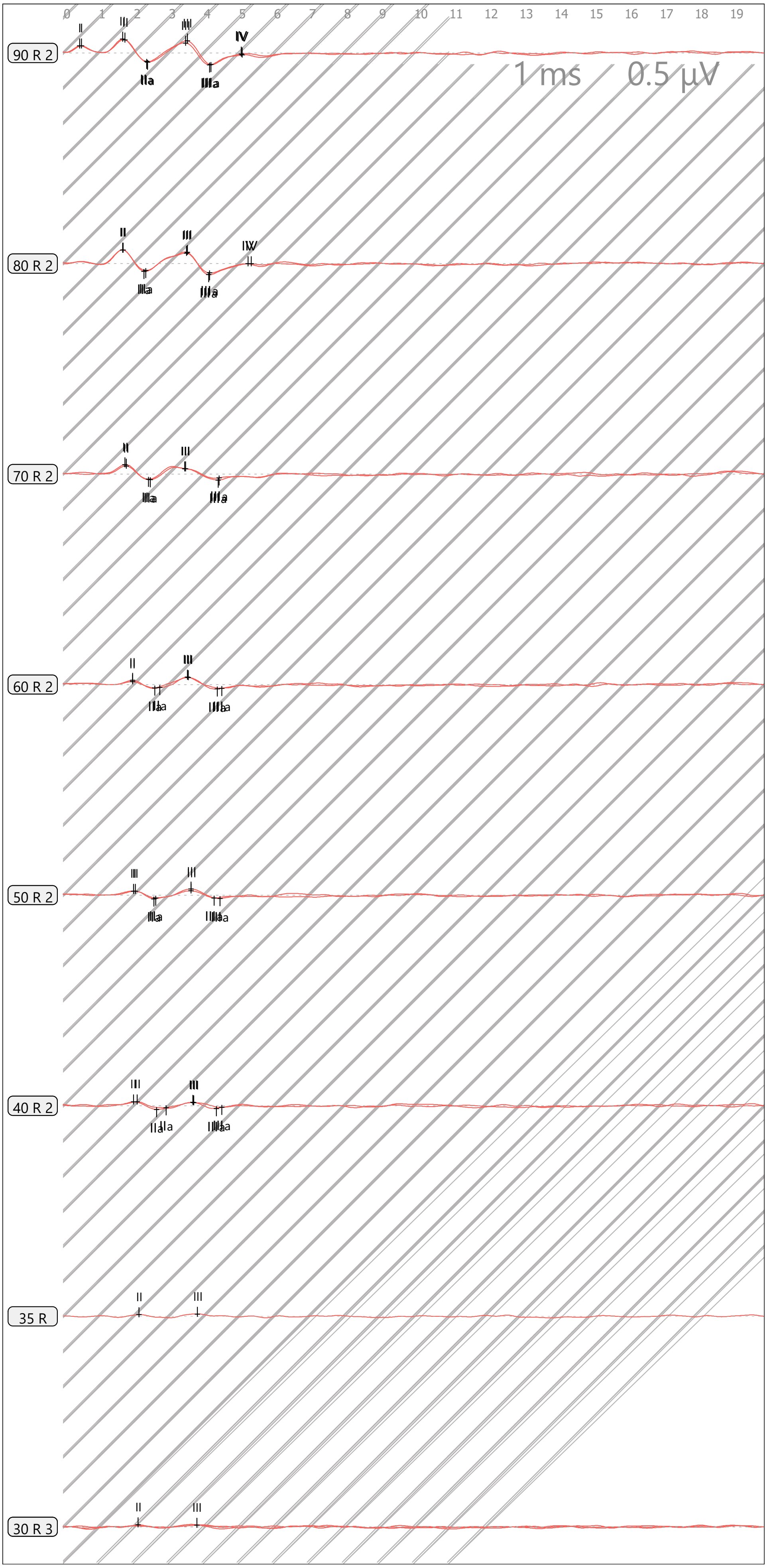
0 1 2 3 4 5 6 7 8 9 10 11 12 13 14 15 16 17 18 19
1 ms
0.5 µV
90 R 2
II
IIa
III
IIIa
IV
80 R 2
II
IIa
III
IIIa
IV
70 R 2
II
IIa
III
IIIa
60 R 2
II
IIa
III
IIIa
50 R 2
II
IIa
III
IIIa
40 R 2
II
IIa
III
IIIa
35 R
II
III
30 R 3
II
III

IV (ms)	V (ms)	I–III (ms)	I–V (ms)	III–V (ms)	
5.11		3.02			
5.08		3.02			
5.27					
5.37					

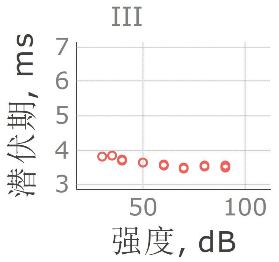

Trace parameters

N	Electr.	HPF, Hz	LPF, Hz	50 Hz	Rejection ±μV	Aver.	Reject
90 R	Cz-M2	200	2000		10	1000	39
90 R 2	Cz-M2	200	2000		10	1000	42
80 R	Cz-M2	200	2000		10	1000	53
80 R 2	Cz-M2	200	2000		10	1000	56
70 R	Cz-M2	200	2000		10	1000	26
70 R 2	Cz-M2	200	2000		10	1000	79
60 R	Cz-M2	200	2000		10	1000	75
60 R 2	Cz-M2	200	2000		10	1000	76
50 R	Cz-M2	200	2000		10	1000	59
50 R 2	Cz-M2	200	2000		10	1000	30
40 R	Cz-M2	200	2000		10	1000	35
40 R 2	Cz-M2	200	2000		10	1000	53
35 R	Cz-M2	200	2000		10	1000	117
30 R	Cz-M2	200	2000		10	1000	46
30 R 2	Cz-M2	200	2000		10	1000	62
30 R 3	Cz-M2	200	2000		10	1000	103

ECochG: ECochG
1: Fpz-M1

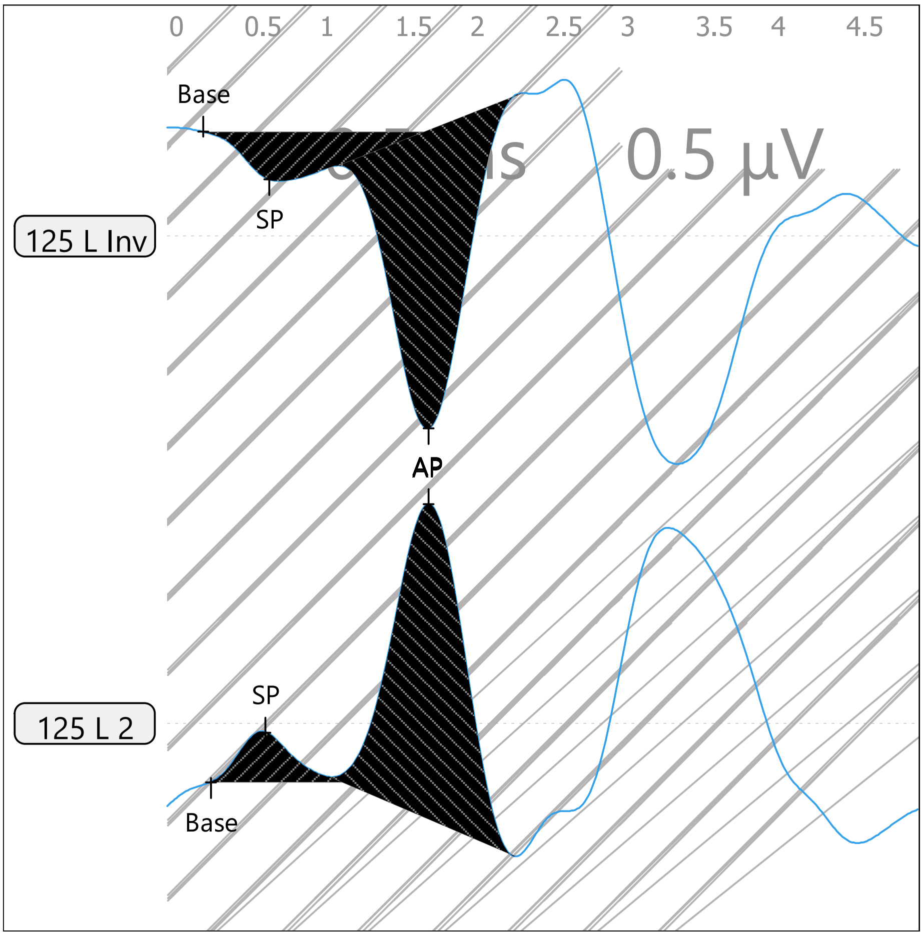

&&

N	Base (ms)	SP (ms)	AP (ms)	SP–Base (ms)	AP–Base (ms)	SP–Base (µV)	AP–Base (µV)	
125 L Inv	0.24	0.67	1.73	0.44	1.49	0.32	1.97	0.1[illegible]
125 L 2	0.29	0.65	1.73	0.36	1.44	0.33	1.85	0.1[illegible]

Trace parameters

N	Electr.	HPF, Hz	LPF, Hz	50 Hz	Rejection ±µV	Aver.	Rej[illegible]
125 L Inv	Fpz-M1	5	2000		50	1500	7[illegible]
125 L 2	Fpz-M1	5	2000		50	1500	6[illegible]

ECochG: ECochG 2:
Fpz-M2

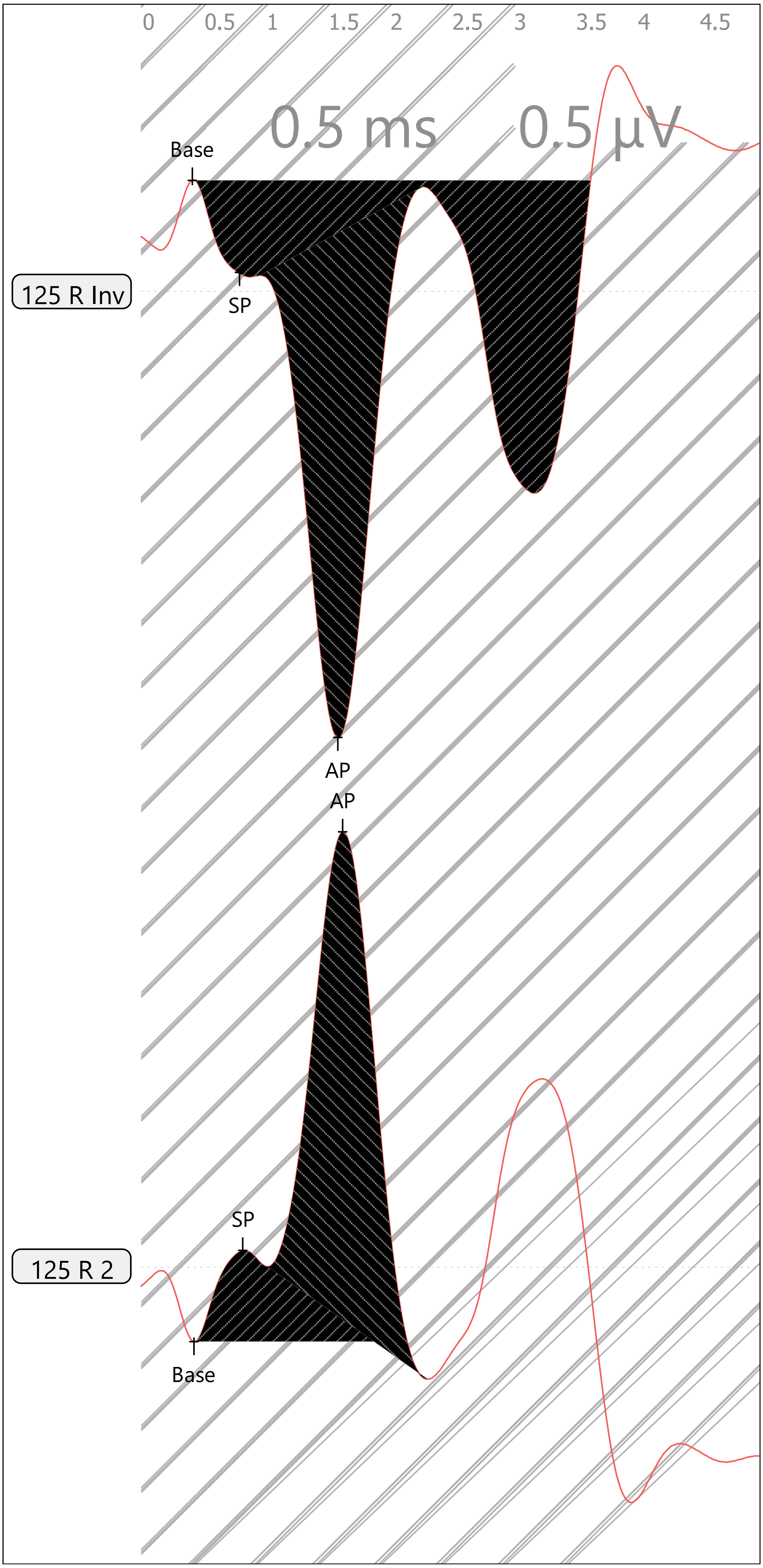

&&

N	Base	SP	AP	SP–Base	AP–Base	SP–Base	AP–Base

	(ms)	(ms)	(ms)	(ms)	(ms)	(µV)	(µV)	
125 R Inv	0.41	0.79	1.59	0.38	1.18	0.76	4.50	0.1
125 R 2	0.42	0.82	1.63	0.40	1.20	0.74	4.12	0.1

Trace parameters

N	Electr.	HPF, Hz	LPF, Hz	50 Hz	Rejection ±µV	Aver.	Rej
125 R Inv	Fpz-M2	5	2000		50	1500	6
125 R 2	Fpz-M2	5	2000		50	1500	4

CONCLUSION:

Doctor:
