# Supplementary material for: Exposure to high-altitude hypobaric hypoxic environment induces low-frequency hearing loss in C57BL/6J mice: Mediated by slowing down the postsynaptic electrical signal transmission speed in the cochlear-inferior colliculus auditory signaling pathway
Source: PLoS One. 2026 Mar 11;21(3):e0342321. doi: 10.1371/journal.pone.0342321 (PMC12978441; doi:10.1371/journal.pone.0342321)
Supplement: S1 File — (ZIP) [file pone.0342321.s001.zip › 2025-6-15-7d-4.pdf]

Exam report

Patient: 2025-6-15-7d-4, - (-)
Date: June 15, 2025

ABR: ABR 2 CLICK
1: Cz-M1

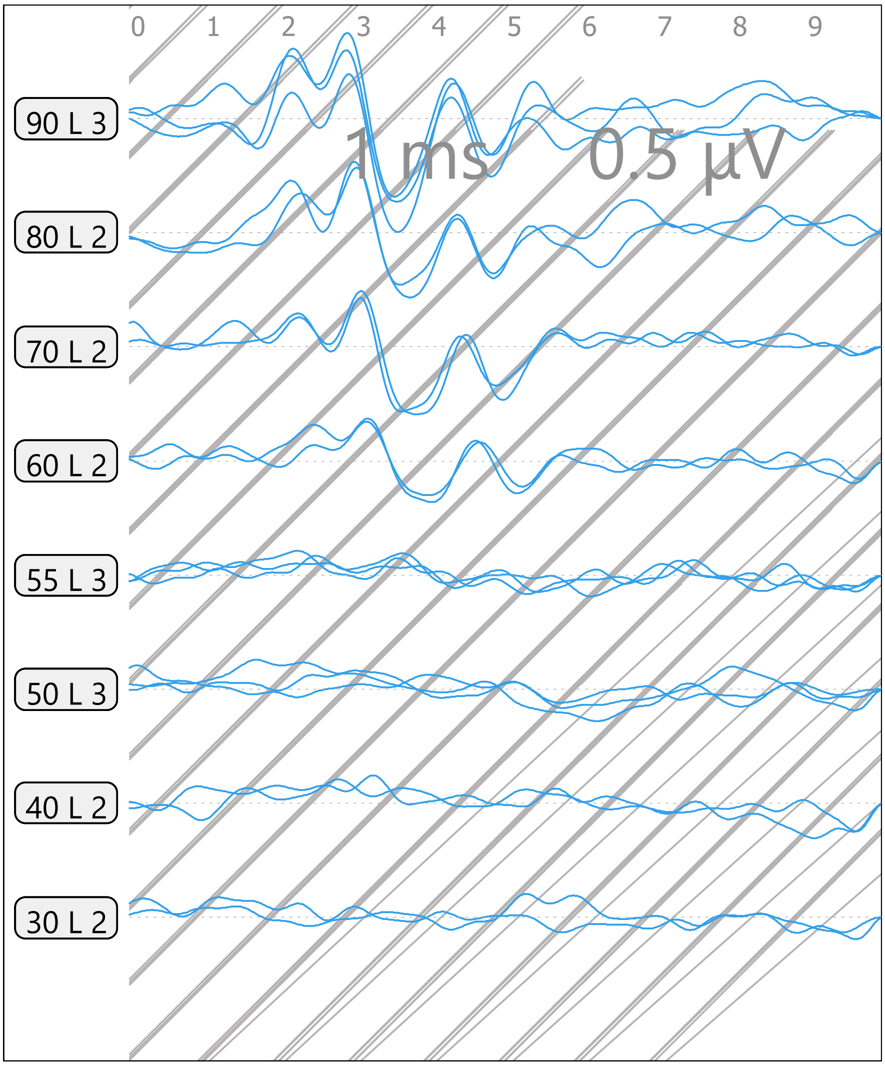

Trace parameters

N	Electr.	HPF, Hz	LPF, Hz	50 Hz	Rejection ±µV	Aver.	Reject.
90 L	Cz-M1	100	2000		10	1000	0
90 L 2	Cz-M1	100	2000		10	1000	0
90 L 3	Cz-M1	100	2000		10	1000	0
80 L	Cz-M1	100	2000		10	1000	0
80 L 2	Cz-M1	100	2000		10	1000	0
70 L	Cz-M1	100	2000		10	1000	0
70 L 2	Cz-M1	100	2000		10	1000	0
60 L	Cz-M1	100	2000		10	1000	0
60 L 2	Cz-M1	100	2000		10	1000	0
55 L	Cz-M1	100	2000		10	1000	0
55 L 2	Cz-M1	100	2000		10	1000	0
55 L 3	Cz-M1	100	2000		10	1000	0
50 L	Cz-M1	100	2000		10	1000	0
50 L 2	Cz-M1	100	2000		10	1000	0
50 L 3	Cz-M1	100	2000		10	1000	0
40 L	Cz-M1	100	2000		10	1000	0

40 L 2	Cz-M1	100	2000		10	1000	0
30 L	Cz-M1	100	2000		10	1000	0
30 L 2	Cz-M1	100	2000		10	1000	0

ABR: ABR 2 tone burst 4000Hz 1
: Cz-M1

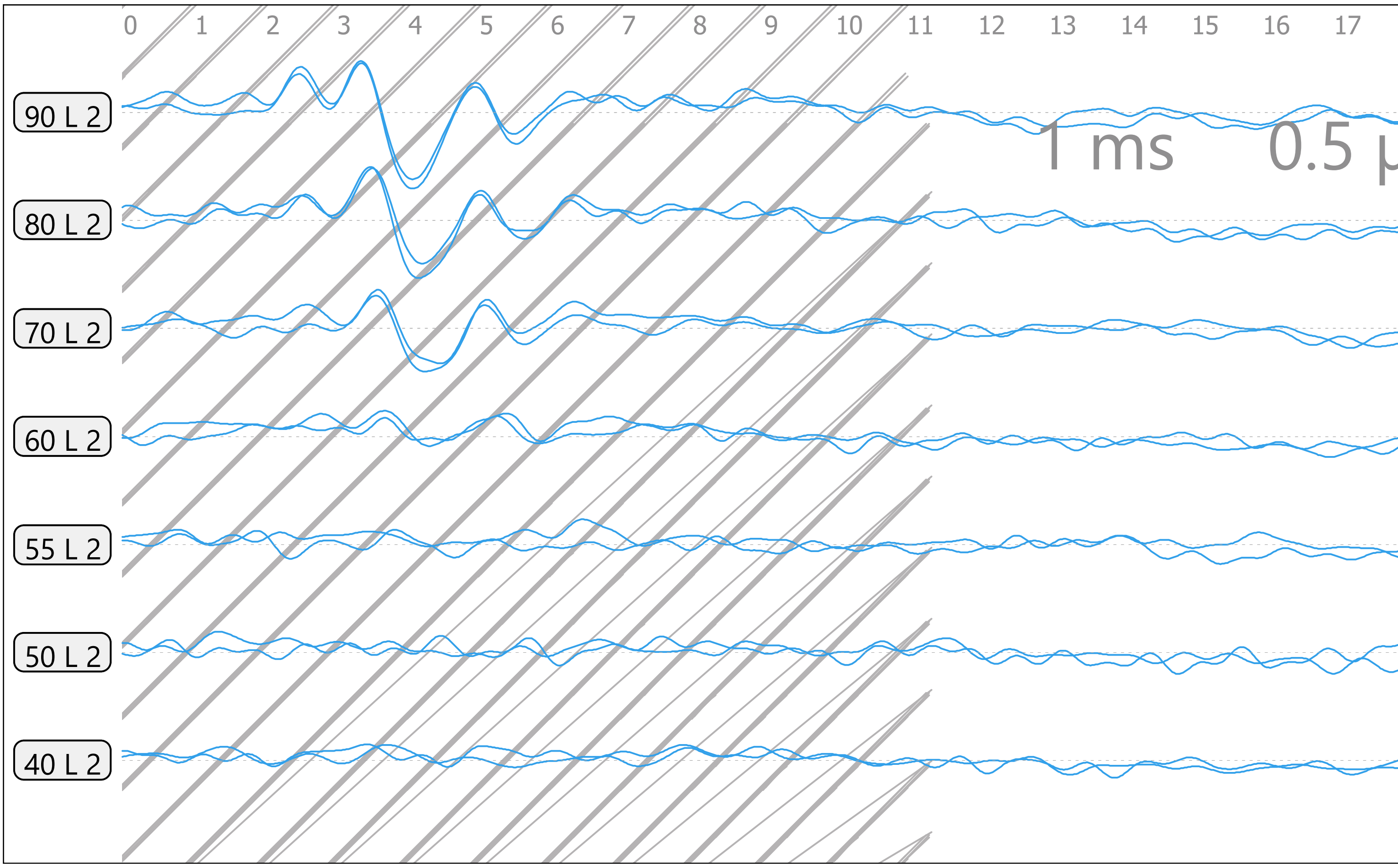

Trace parameters

N	Electr.	HPF, Hz	LPF, Hz	50 Hz	Rejection ±µV	Aver.	Reject.
90 L	Cz-M1	200	2000		10	1000	0
90 L 2	Cz-M1	200	2000		10	1000	0
80 L	Cz-M1	200	2000		10	1000	0
80 L 2	Cz-M1	200	2000		10	1000	0
70 L	Cz-M1	200	2000		10	1000	0
70 L 2	Cz-M1	200	2000		10	1000	0
60 L	Cz-M1	200	2000		10	1000	0
60 L 2	Cz-M1	200	2000		10	1000	0
55 L	Cz-M1	200	2000		10	1000	0
55 L 2	Cz-M1	200	2000		10	1000	0
50 L	Cz-M1	200	2000		10	1000	0
50 L 2	Cz-M1	200	2000		10	1000	0
40 L	Cz-M1	200	2000		10	1000	0

40 L 2	Cz-M1	200	2000		10	1000	0

ABR: ABR 2 8000Hz 1: Cz-M1

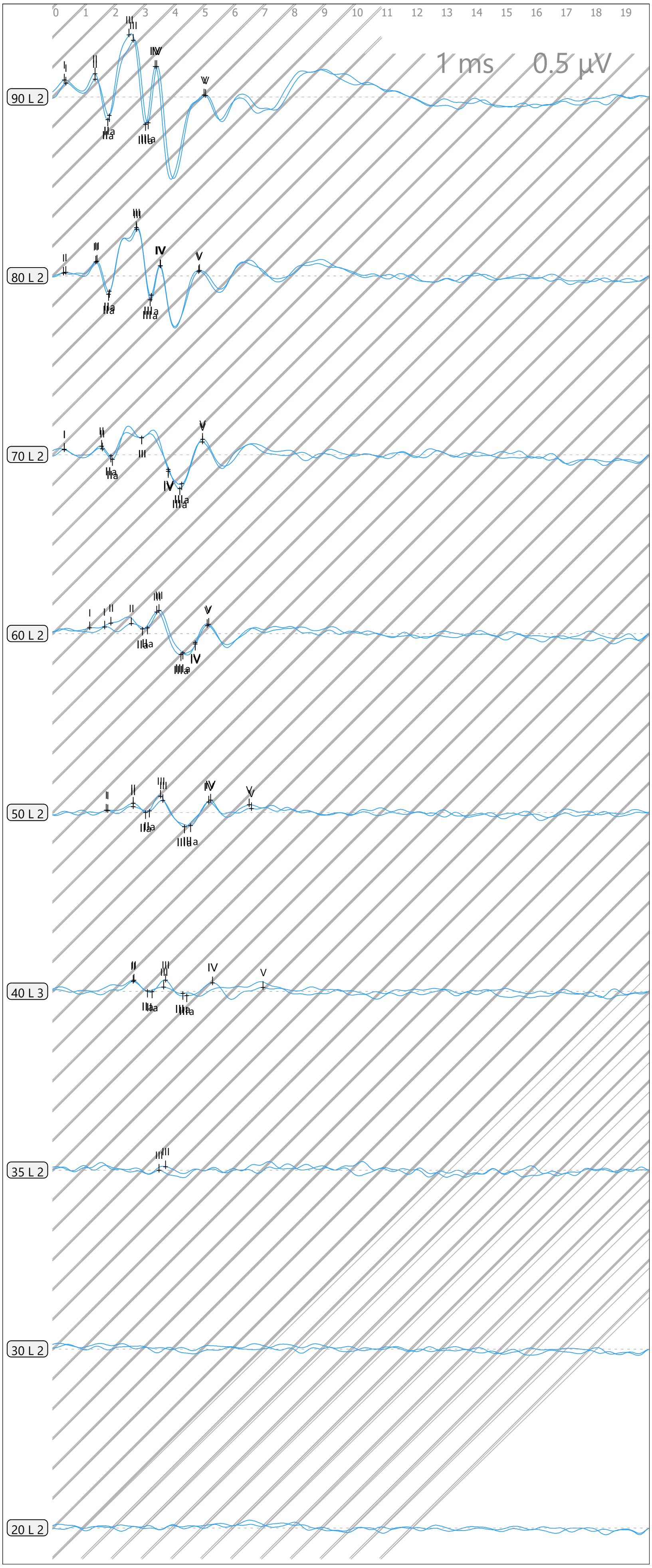
0 1 2 3 4 5 6 7 8 9 10 11 12 13 14 15 16 17 18 19
1 ms
0.5 µV
90 L 2
80 L 2
70 L 2
60 L 2
50 L 2
40 L 3
35 L 2
30 L 2
20 L 2

&&

N	I (ms)	II (ms)	III (ms)	IV (ms)	V (ms)
90 L	0.40	1.43	2.57	3.44	5.08
90 L 2	0.45	1.43	2.70	3.49	5.13
80 L	0.45	1.48	2.83	3.62	4.89
80 L 2	0.37	1.46	2.80	3.60	4.92
70 L	0.40	1.67	2.99	3.89	5.03
70 L 2	0.40	1.64	2.99	3.89	5.03
60 L	1.24	1.96	3.49	4.79	5.19
60 L 2	1.75	2.65	3.57	4.79	5.24
50 L	1.80	2.70	3.62	5.29	6.67
50 L 2	1.85	2.70	3.70	5.24	6.59
40 L		2.70	3.78	5.37	7.06
40 L 3		2.73	3.73		
35 L			3.78		
35 L 2			3.57		

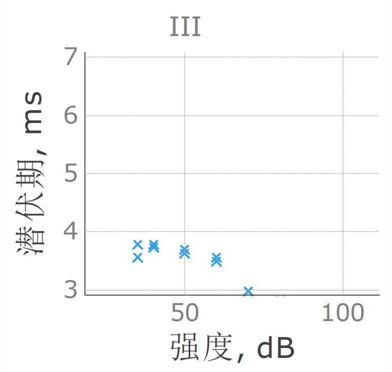

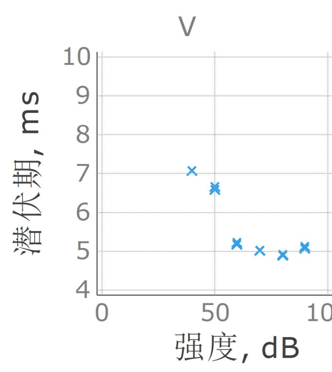

Trace parameters

N	Electr.	HPF, Hz	LPF, Hz	50 Hz	Rejection ±μV	Aver.	Reject.
90 L	Cz-M1	200	2000		10	1000	0
90 L 2	Cz-M1	200	2000		10	1000	0
80 L	Cz-M1	200	2000		10	1000	0
80 L 2	Cz-M1	200	2000		10	1000	0
70 L	Cz-M1	200	2000		10	1000	0
70 L 2	Cz-M1	200	2000		10	1000	0
60 L	Cz-M1	200	2000		10	1000	0
60 L 2	Cz-M1	200	2000		10	1000	0
50 L	Cz-M1	200	2000		10	1000	0
50 L 2	Cz-M1	200	2000		10	1000	0
40 L	Cz-M1	200	2000		10	1000	0
40 L 3	Cz-M1	200	2000		10	1000	0
35 L	Cz-M1	200	2000		10	1000	0
35 L 2	Cz-M1	200	2000		10	1000	0
30 L	Cz-M1	200	2000		10	1000	0

30 L 2	Cz-M1	200	2000		10	1000	0
20 L	Cz-M1	200	2000		10	1000	0
20 L 2	Cz-M1	200	2000		10	1000	0

ABR: ABR 2 CLICK
2: Cz-M2

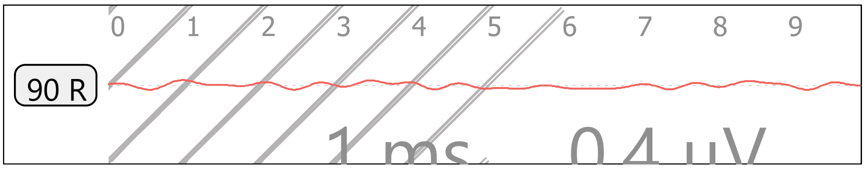

Trace parameters

N	Electr.	HPF, Hz	LPF, Hz	50 Hz	Rejection ±µV	Aver.	Reject.
90 R	Cz-M2	100	2000		10	1000	0

ECochG: ECochG
1: Cz-M1

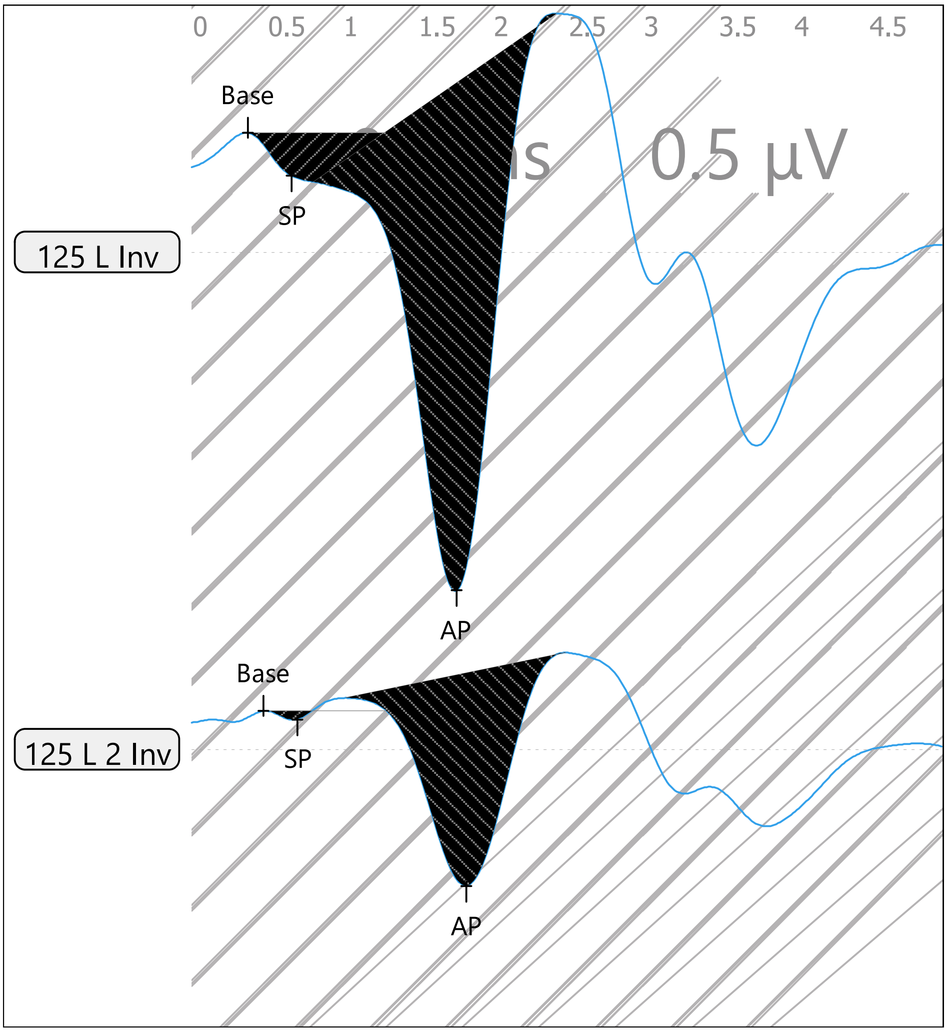

	Base (ms)	SP (ms)	AP (ms)	SP–Base (ms)	AP–Base (ms)	SP–Base (µV)	AP–Base (µV)	
125 L Inv	0.37	0.66	1.76	0.29	1.39	0.28	3.04	0
125 L 2 Inv	0.48	0.70	1.83	0.22	1.35	0.05	1.16	0

Trace parameters

N	Electr.	HPF, Hz	LPF, Hz	50 Hz	Rejection ±µV	Aver.	R

125 L Inv	Cz-M1	5	2000		50	1383	
125 L 2 Inv	Cz-M1	5	2000		50	1451	

CONCLUSION:

Doctor:
