# Supplementary material for: Exposure to high-altitude hypobaric hypoxic environment induces low-frequency hearing loss in C57BL/6J mice: Mediated by slowing down the postsynaptic electrical signal transmission speed in the cochlear-inferior colliculus auditory signaling pathway
Source: PLoS One. 2026 Mar 11;21(3):e0342321. doi: 10.1371/journal.pone.0342321 (PMC12978441; doi:10.1371/journal.pone.0342321)
Supplement: S1 File — (ZIP) [file pone.0342321.s001.zip › 2025-6-16-20d-2.pdf]

Exam report

Patient: 2025-6-16-20d-2**,** - (-)
Date: June 16, 2025

ABR: ABR 2 CLICK
1: Cz-M1

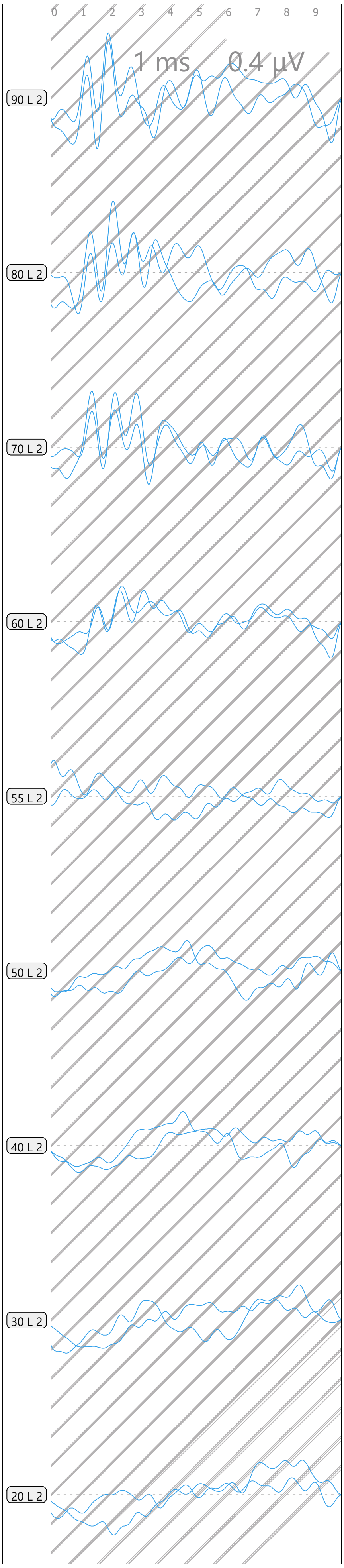

0 1 2 3 4 5 6 7 8 9
1 ms
0.4 µV
90 L 2
80 L 2
70 L 2
60 L 2
55 L 2
50 L 2
40 L 2
30 L 2
20 L 2

Trace parameters

N	Electr.	HPF, Hz	LPF, Hz	50 Hz	Rejection ±µV	Aver.	Reject.
90 L	Cz-M1	100	2000		10	1000	0
90 L 2	Cz-M1	100	2000		10	1000	0
80 L	Cz-M1	100	2000		10	1000	0
80 L 2	Cz-M1	100	2000		10	1000	0
70 L	Cz-M1	100	2000		10	1000	0
70 L 2	Cz-M1	100	2000		10	1000	0
60 L	Cz-M1	100	2000		10	1000	0
60 L 2	Cz-M1	100	2000		10	1000	0
55 L	Cz-M1	100	2000		10	1000	0
55 L 2	Cz-M1	100	2000		10	1000	0
50 L	Cz-M1	100	2000		10	1000	0
50 L 2	Cz-M1	100	2000		10	1000	0
40 L	Cz-M1	100	2000		10	1000	0
40 L 2	Cz-M1	100	2000		10	1000	0
30 L	Cz-M1	100	2000		10	1000	0
30 L 2	Cz-M1	100	2000		10	1000	0
20 L	Cz-M1	100	2000		10	1000	0
20 L 2	Cz-M1	100	2000		10	1000	0

ABR: ABR 2 4000Hz 1: Cz-M1

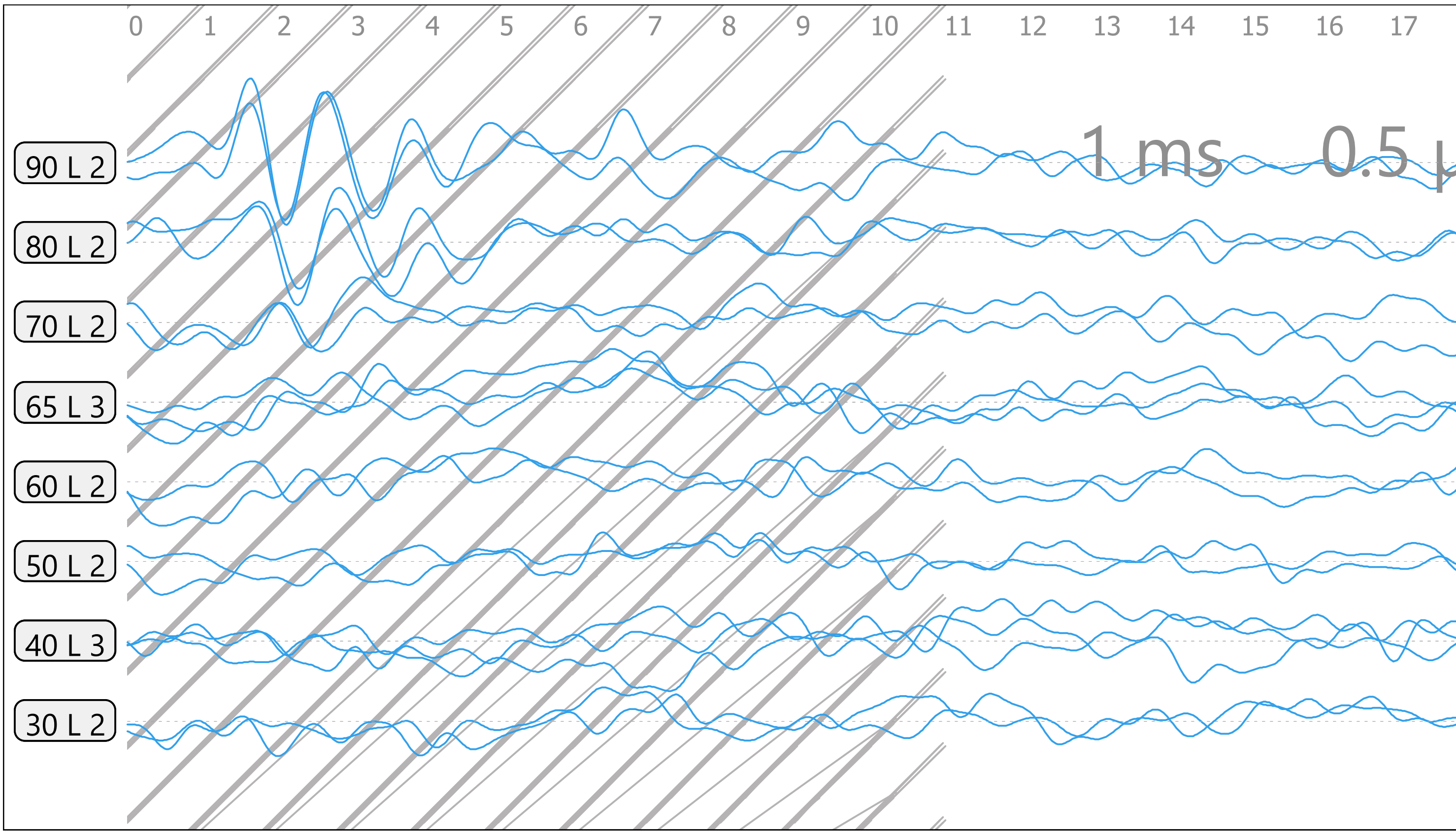

Trace parameters

N	Electr.	HPF, Hz	LPF, Hz	50 Hz	Rejection ±µV	Aver.	Reject.
90 L	Cz-M1	200	2000		10	1000	0
90 L 2	Cz-M1	200	2000		10	1000	0
80 L	Cz-M1	200	2000		10	1000	0
80 L 2	Cz-M1	200	2000		10	1000	0

70 L	Cz-M1	200	2000		10	1000	0
70 L 2	Cz-M1	200	2000		10	1000	0
65 L	Cz-M1	200	2000		10	1000	0
65 L 2	Cz-M1	200	2000		10	1000	0
65 L 3	Cz-M1	200	2000		10	1000	0
60 L	Cz-M1	200	2000		10	1000	0
60 L 2	Cz-M1	200	2000		10	1000	0
50 L	Cz-M1	200	2000		10	1000	0
50 L 2	Cz-M1	200	2000		10	1000	0
40 L	Cz-M1	200	2000		10	1000	0
40 L 2	Cz-M1	200	2000		10	1000	0
40 L 3	Cz-M1	200	2000		10	1000	0
30 L	Cz-M1	200	2000		10	1000	0
30 L 2	Cz-M1	200	2000		10	1000	0

ABR: ABR 2 8000Hz 1: Cz-M1

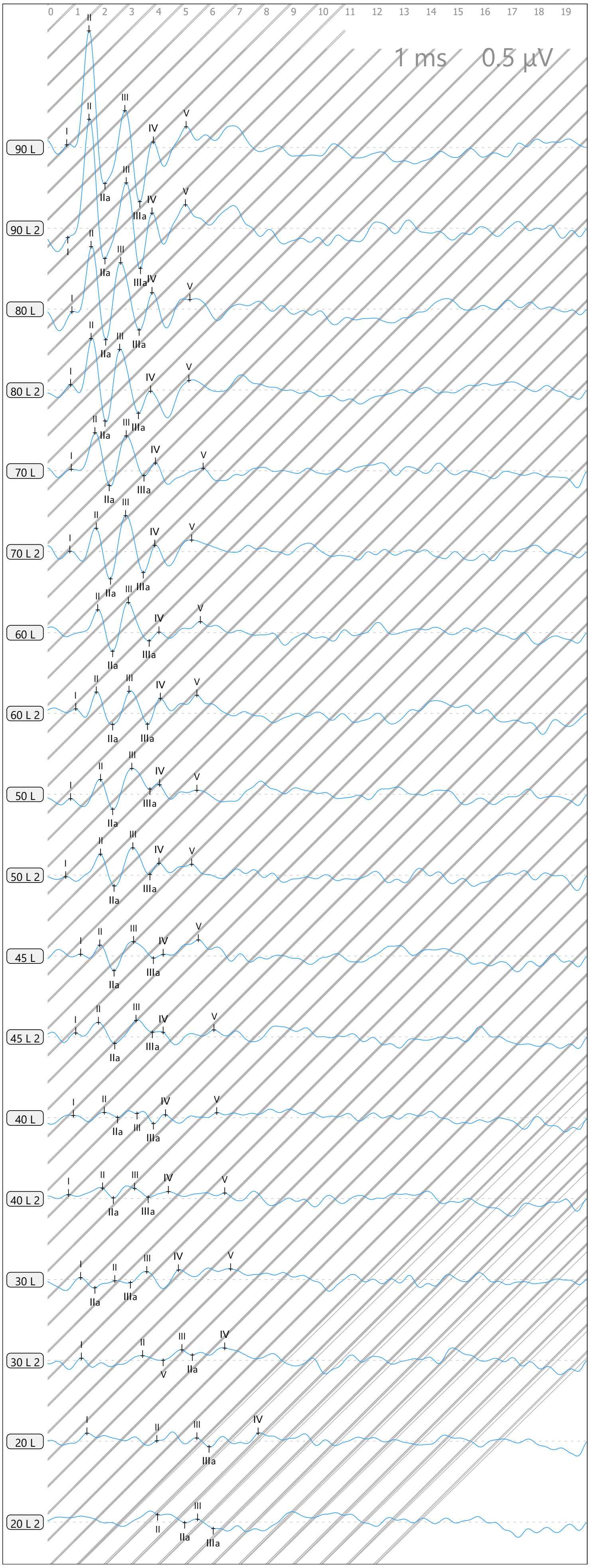
0 1 2 3 4 5 6 7 8 9 10 11 12 13 14 15 16 17 18 19
1 ms
0.5 µV
90 L
90 L 2
80 L
80 L 2
70 L
70 L 2
60 L
60 L 2
50 L
50 L 2
45 L
45 L 2
40 L
40 L 2
30 L
30 L 2
20 L
20 L 2

&&

N	I (ms)	II (ms)	III (ms)	IV (ms)	V (ms)
90 L	0.71	1.53	2.86	3.92	5.13
90 L 2	0.74	1.53	2.91	3.86	5.11
80 L	0.90	1.61	2.70	3.86	5.27
80 L 2	0.85	1.61	2.67	3.81	5.24
70 L	0.87	1.75	2.91	4.00	5.77
70 L 2	0.82	1.80	2.88	3.97	5.34
60 L		1.85	2.99	4.13	5.66
60 L 2	1.03	1.80	3.02	4.18	5.53
50 L	0.85	1.96	3.12	4.15	5.53
50 L 2	0.66	1.96	3.15	4.13	5.34
45 L	1.22	1.93	3.18	4.29	5.58
45 L 2	1.03	1.88	3.28	4.29	6.16
40 L	0.95	2.09	3.31	4.37	6.27
40 L 2	0.77	2.04	3.23	4.47	6.56
30 L	1.22	2.49	3.68	4.84	6.77
30 L 2	1.24	3.52	4.97	6.56	4.29
20 L	1.46	4.05	5.53	7.81	
20 L 2		4.07	5.56		

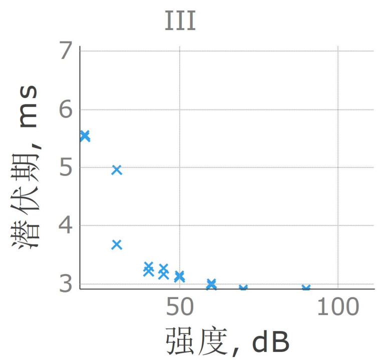

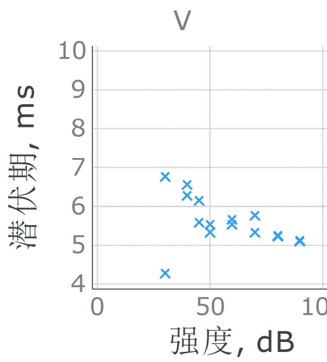

Trace parameters

N	Electr.	HPF, Hz	LPF, Hz	50 Hz	Rejection ±μV	Aver.	Reject.
90 L	Cz-M1	200	2000		10	1000	0
90 L 2	Cz-M1	200	2000		10	1000	0
80 L	Cz-M1	200	2000		10	1000	0
80 L 2	Cz-M1	200	2000		10	1000	0
70 L	Cz-M1	200	2000		10	1000	0
70 L 2	Cz-M1	200	2000		10	1000	0
60 L	Cz-M1	200	2000		10	1000	0
60 L 2	Cz-M1	200	2000		10	1000	0
50 L	Cz-M1	200	2000		10	1000	0
50 L 2	Cz-M1	200	2000		10	1000	0
45 L	Cz-M1	200	2000		10	1000	0
45 L 2	Cz-M1	200	2000		10	1000	0

40 L	Cz-M1	200	2000		10	1000	0
40 L 2	Cz-M1	200	2000		10	1000	0
30 L	Cz-M1	200	2000		10	1000	0
30 L 2	Cz-M1	200	2000		10	1000	0
20 L	Cz-M1	200	2000		10	1000	0
20 L 2	Cz-M1	200	2000		10	1000	0

ABR: ABR 2 CLICK
2: Cz-M2

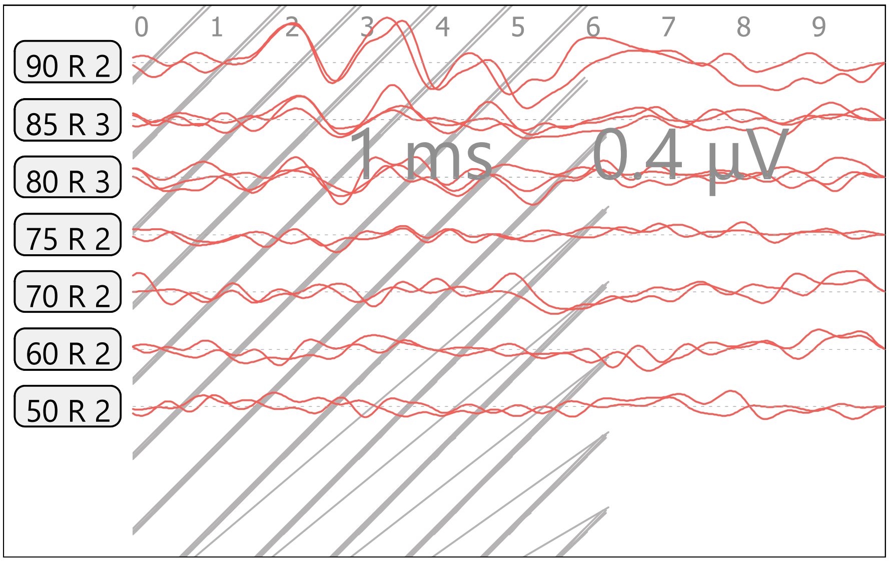

Trace parameters

N	Electr.	HPF, Hz	LPF, Hz	50 Hz	Rejection ±µV	Aver.	Reject
90 R	Cz-M2	100	2000		10	1000	0
90 R 2	Cz-M2	100	2000		10	1000	0
85 R	Cz-M2	100	2000		10	1000	0
85 R 2	Cz-M2	100	2000		10	1000	0
85 R 3	Cz-M2	100	2000		10	1000	0
80 R	Cz-M2	100	2000		10	1000	0
80 R 2	Cz-M2	100	2000		10	1000	0
80 R 3	Cz-M2	100	2000		10	1000	0
75 R	Cz-M2	100	2000		10	1000	0
75 R 2	Cz-M2	100	2000		10	1000	0
70 R	Cz-M2	100	2000		10	1000	0
70 R 2	Cz-M2	100	2000		10	1000	0
60 R	Cz-M2	100	2000		10	1000	0
60 R 2	Cz-M2	100	2000		10	1000	0
50 R	Cz-M2	100	2000		10	1000	0
50 R 2	Cz-M2	100	2000		10	1000	0

ABR: ABR 2 4000Hz 2: Cz-M2

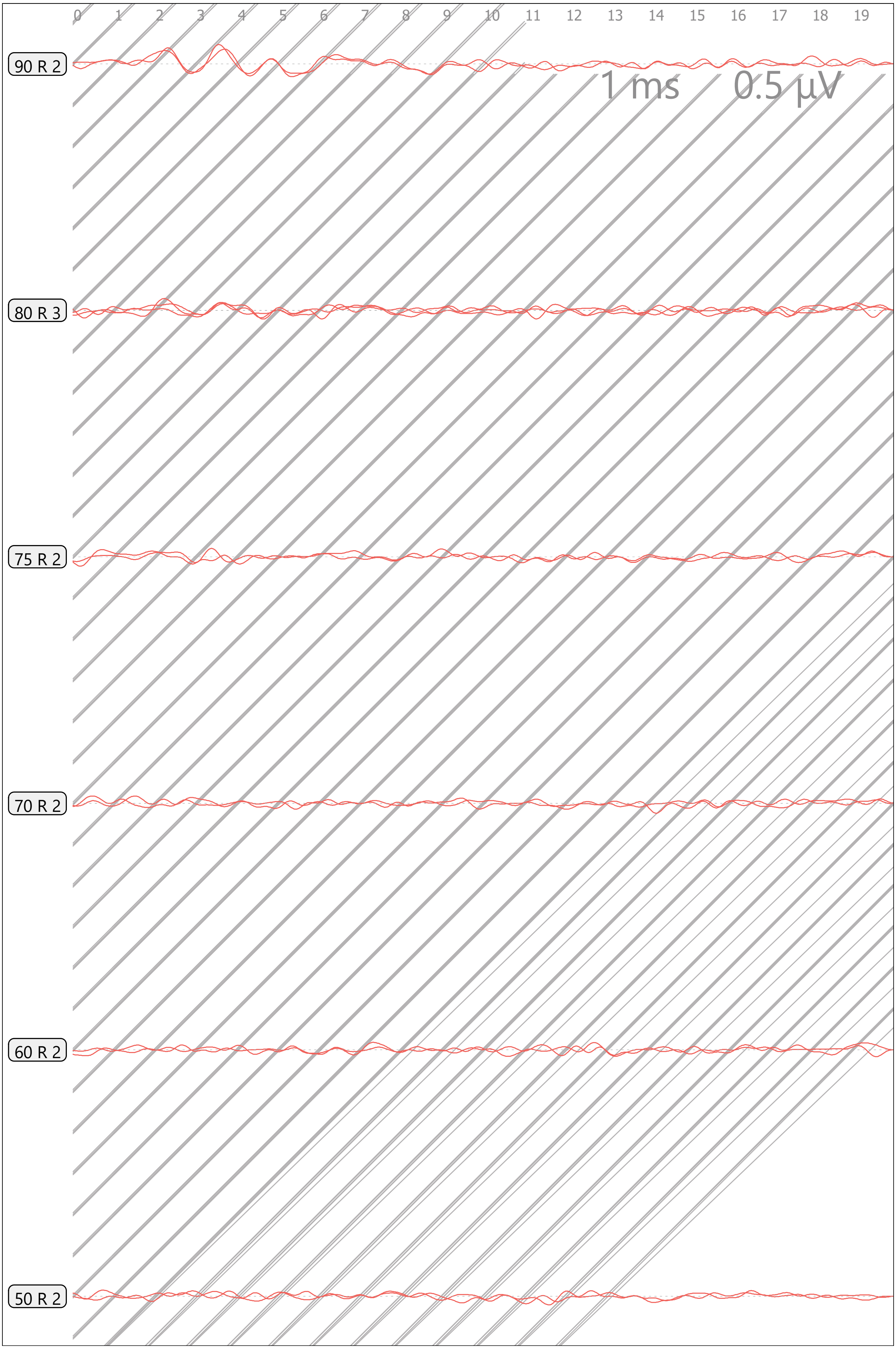
0 1 2 3 4 5 6 7 8 9 10 11 12 13 14 15 16 17 18 19
1 ms
0.5 µV
90 R 2
80 R 3
75 R 2
70 R 2
60 R 2
50 R 2

Trace parameters

N	Electr.	HPF, Hz	LPF, Hz	50 Hz	Rejection ±μV	Aver.	Reject
90 R	Cz-M2	200	2000		10	1000	0
90 R 2	Cz-M2	200	2000		10	1000	0
80 R	Cz-M2	200	2000		10	1000	0
80 R 2	Cz-M2	200	2000		10	1000	0
80 R 3	Cz-M2	200	2000		10	1000	0
75 R	Cz-M2	200	2000		10	1000	0
75 R 2	Cz-M2	200	2000		10	1000	0
70 R	Cz-M2	200	2000		10	1000	0
70 R 2	Cz-M2	200	2000		10	1000	0
60 R	Cz-M2	200	2000		10	1000	0
60 R 2	Cz-M2	200	2000		10	1000	0
50 R	Cz-M2	200	2000		10	1000	0
50 R 2	Cz-M2	200	2000		10	1000	0

ABR: ABR 2 8000Hz 2: Cz-M2

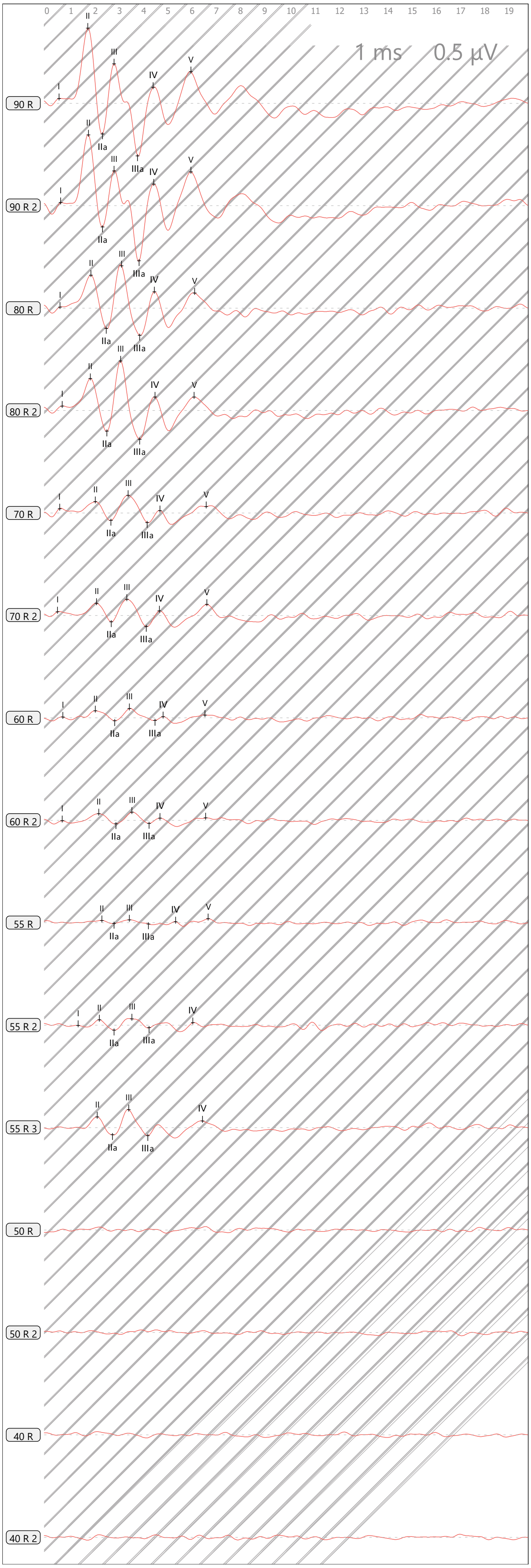
0 1 2 3 4 5 6 7 8 9 10 11 12 13 14 15 16 17 18 19
1 ms
0.5 µV
90 R
90 R 2
80 R
80 R 2
70 R
70 R 2
60 R
60 R 2
55 R
55 R 2
55 R 3
50 R
50 R 2
40 R
40 R 2

IV (ms)	V (ms)	I–III (ms)	I–V (ms)	III–V (ms)
4.50	6.06	2.28	5.45	3.18
4.52	6.06	2.20	5.37	3.18
4.55	6.22	2.54	5.56	3.02
4.58	6.19	2.41	5.45	3.04
4.79	6.69	2.83	6.06	3.23
4.76	6.72	2.86	6.16	3.31
4.92	6.64	2.75	5.87	3.12
4.79	6.67	2.88	5.93	3.04
5.42	6.77			3.25
6.14		2.22		
6.54				

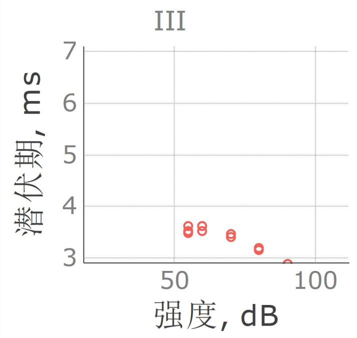

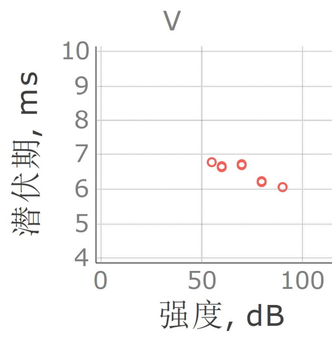

Trace parameters

N	Electr.	HPF, Hz	LPF, Hz	50 Hz	Rejection ±μV	Aver.	Reject
90 R	Cz-M2	200	2000		10	1000	0
90 R 2	Cz-M2	200	2000		10	1000	0
80 R	Cz-M2	200	2000		10	1000	0
80 R 2	Cz-M2	200	2000		10	1000	0
70 R	Cz-M2	200	2000		10	1000	0
70 R 2	Cz-M2	200	2000		10	1000	0
60 R	Cz-M2	200	2000		10	1000	0
60 R 2	Cz-M2	200	2000		10	1000	0
55 R	Cz-M2	200	2000		10	1000	0
55 R 2	Cz-M2	200	2000		10	1000	0
55 R 3	Cz-M2	200	2000		10	1000	0
50 R	Cz-M2	200	2000		10	1000	0
50 R 2	Cz-M2	200	2000		10	1000	0
40 R	Cz-M2	200	2000		10	1000	0
40 R 2	Cz-M2	200	2000		10	1000	0

ECochG: ECochG 1:
Fpz-M1

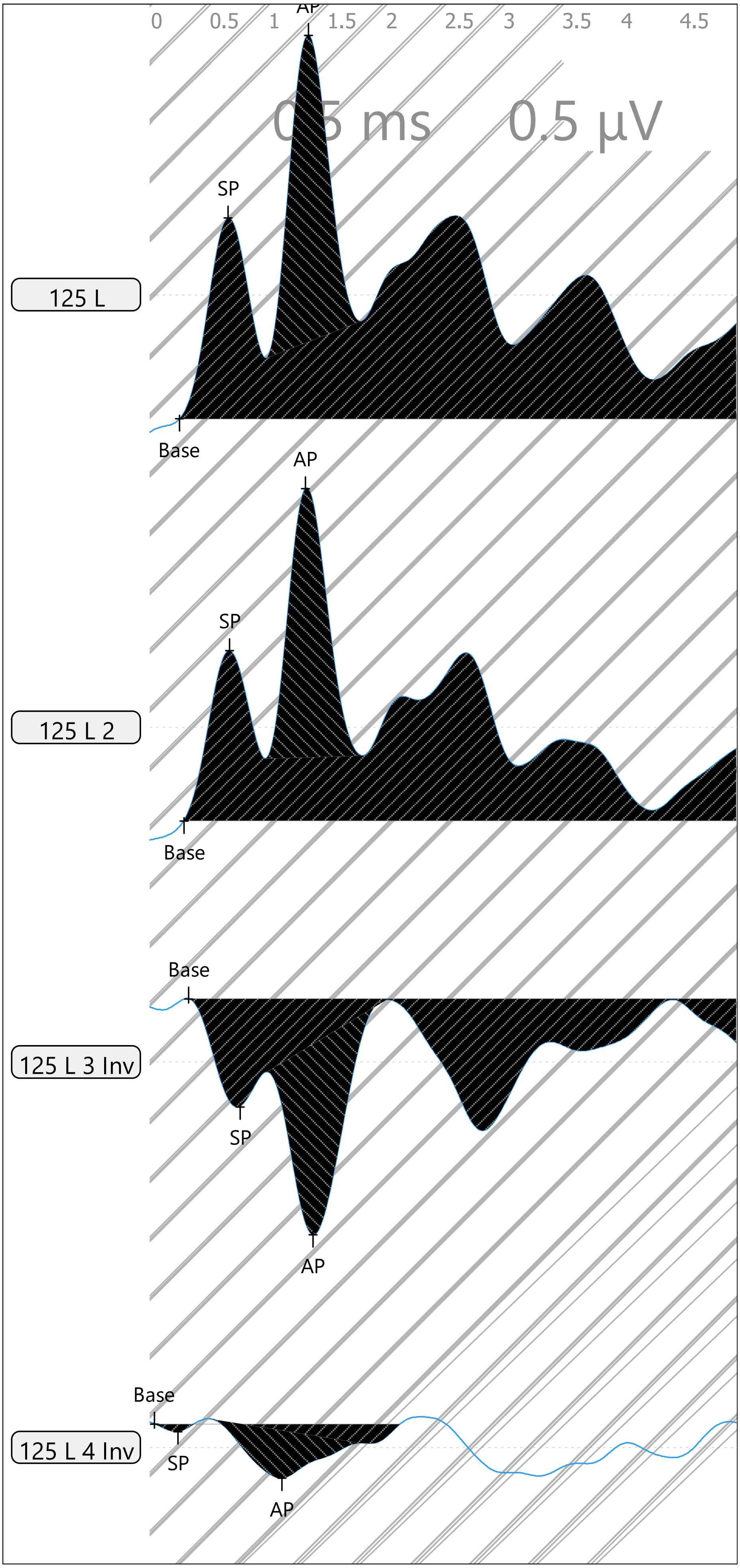

0
0.5
1
1.5
2
2.5
3
3.5
4
4.5
0.5 ms
0.5 µV
AP
SP
Base
125 L
AP
SP
Base
125 L 2
Base
SP
AP
125 L 3 Inv
Base
SP
AP
125 L 4 Inv

&&

N	Base (ms)	SP (ms)	AP (ms)	SP–Base (ms)	AP–Base (ms)	SP–Base (µV)	AP–Base (µV)	
125 L	0.25	0.66	1.35	0.41	1.10	1.71	3.26	0
125 L 2	0.29	0.67	1.32	0.38	1.03	1.44	2.83	0
125 L 3 Inv	0.33	0.77	1.39	0.44	1.06	0.92	2.01	0
125 L 4 Inv	0.04	0.24	1.12	0.20	1.08	0.06	0.46	0

Trace parameters

N	Electr.	HPF, Hz	LPF, Hz	50 Hz	Rejection ±µV	Aver.	R
125 L	Fpz-M1	5	2000		50	1500	
125 L 2	Fpz-M1	5	2000		50	1500	
125 L 3 Inv	Fpz-M1	5	2000		50	1500	
125 L 4 Inv	Fpz-M1	5	2000		50	1500	

EcochG: ECochG
2: Fpz-M2

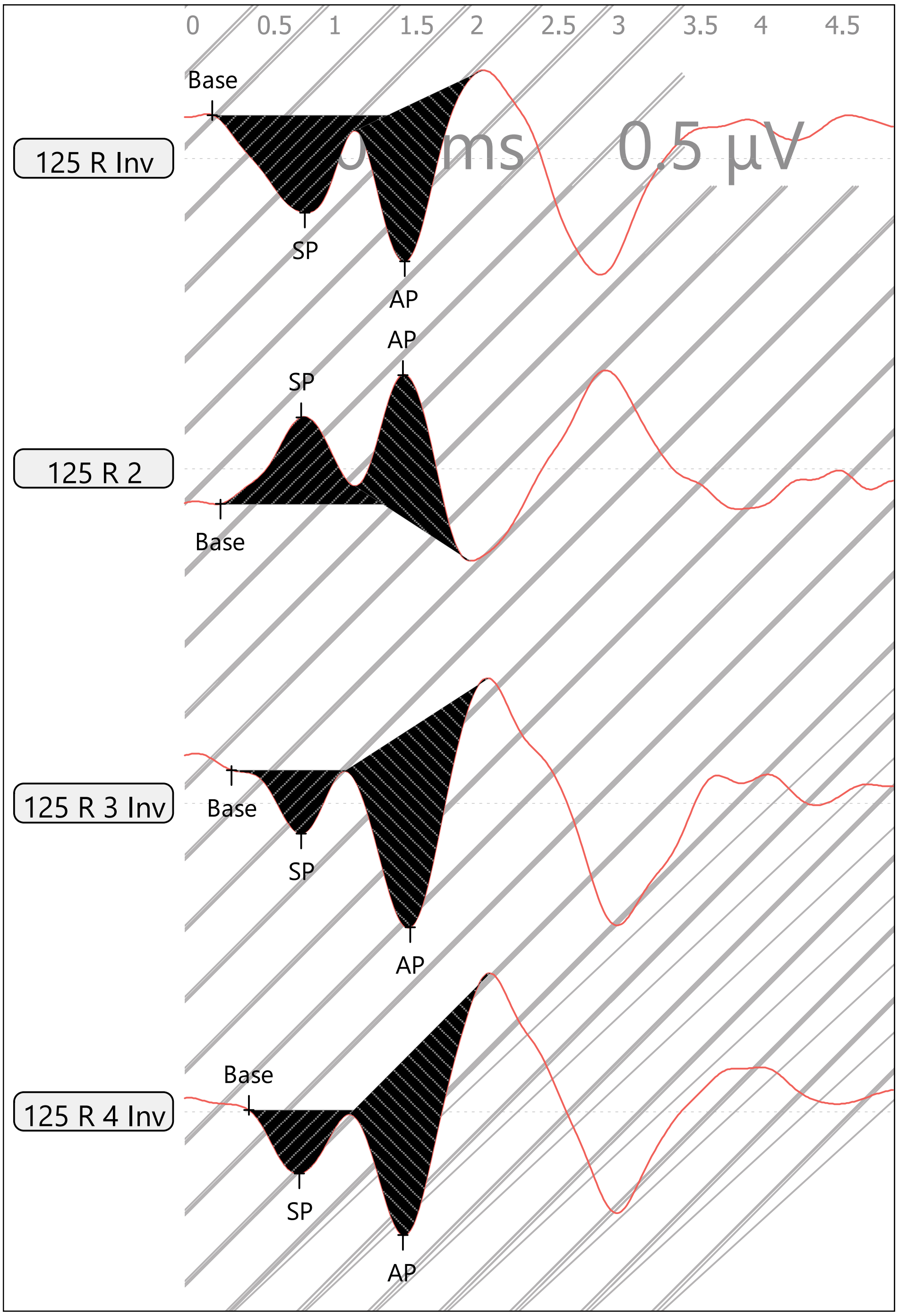

&&

N	Base (ms)	SP (ms)	AP (ms)	SP–Base (ms)	AP–Base (ms)	SP–Base (μV)	AP–Base (μV)	
125 R Inv	0.20	0.85	1.55	0.65	1.35	0.68	1.03	0
125 R 2	0.25	0.82	1.53	0.57	1.28	0.61	0.91	0
125 R 3 Inv	0.33	0.82	1.59	0.49	1.26	0.45	1.11	0
125 R 4 Inv	0.45	0.81	1.53	0.36	1.08	0.45	0.88	0

Trace parameters

N	Electr.	HPF, Hz	LPF, Hz	50 Hz	Rejection ±μV	Aver.	R
125 R Inv	Fpz-M2	5	2000		50	1500	
125 R 2	Fpz-M2	5	2000		50	693	
125 R 3 Inv	Fpz-M2	5	2000		50	702	
125 R 4 Inv	Fpz-M2	5	2000		50	420	

CONCLUSION:

Doctor:
