# Supplementary material for: Exposure to high-altitude hypobaric hypoxic environment induces low-frequency hearing loss in C57BL/6J mice: Mediated by slowing down the postsynaptic electrical signal transmission speed in the cochlear-inferior colliculus auditory signaling pathway
Source: PLoS One. 2026 Mar 11;21(3):e0342321. doi: 10.1371/journal.pone.0342321 (PMC12978441; doi:10.1371/journal.pone.0342321)
Supplement: S1 File — (ZIP) [file pone.0342321.s001.zip › 2025-6-17-15d-1.pdf]

# Exam report

**Patient:** 2025-6-17-15d-1- ( - )
**Date:** June 17, 2025

**ABR**: ABR 2 CLICK
1: Cz-M1

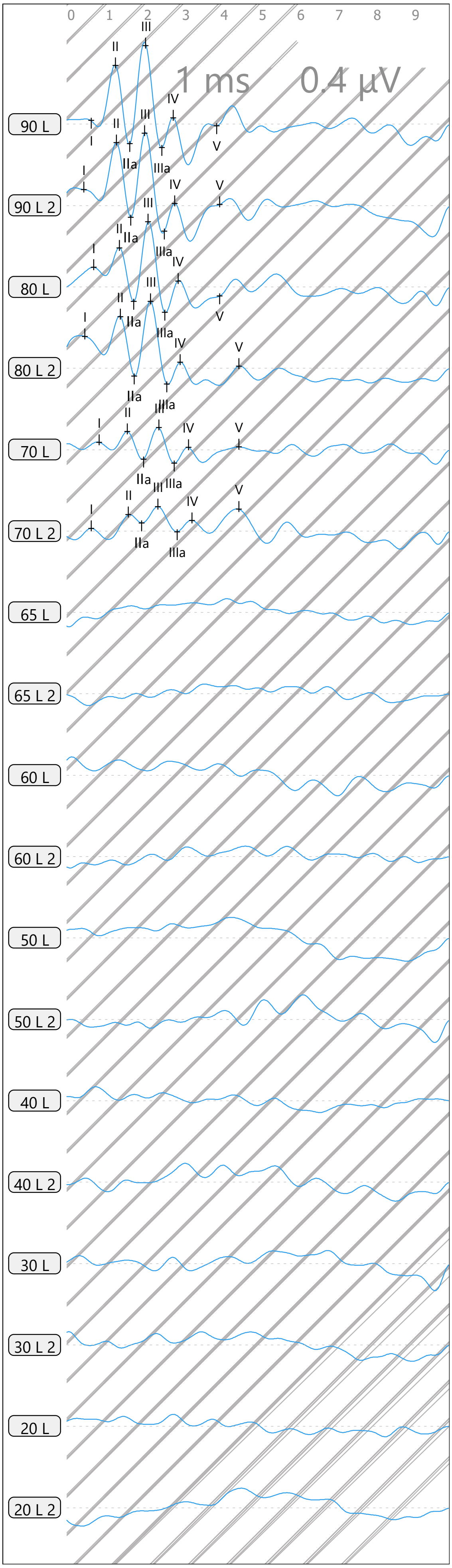
0 1 2 3 4 5 6 7 8 9
1 ms
0.4 µV
90 L
90 L 2
80 L
80 L 2
70 L
70 L 2
65 L
65 L 2
60 L
60 L 2
50 L
50 L 2
40 L
40 L 2
30 L
30 L 2
20 L
20 L 2
I
II
IIa
III
IIIa
IV
V

&&

| N | I (ms) | II (ms) | III (ms) | IV (ms) | V (ms) |
|---|---|---|---|---|---|
| 90 L | 0.64 | 1.27 | 2.06 | 2.78 | 3.92 |
| 90 L 2 | 0.45 | 1.30 | 2.04 | 2.83 | 4.00 |
| 80 L | 0.71 | 1.38 | 2.12 | 2.91 | 4.00 |
| 80 L 2 | 0.48 | 1.40 | 2.20 | 2.96 | 4.50 |
| 70 L | 0.85 | 1.59 | 2.41 | 3.18 | 4.50 |
| 70 L 2 | 0.64 | 1.61 | 2.38 | 3.28 | 4.50 |

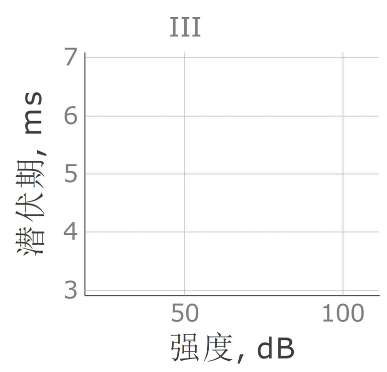


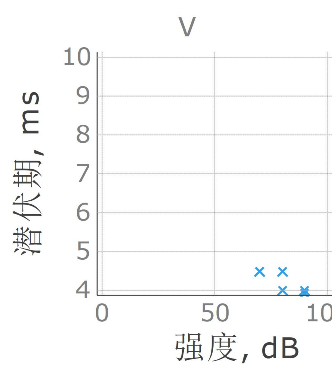


Trace parameters

| N | Electr. | HPF, Hz | LPF, Hz | 50 Hz | Rejection ±µV | Aver. | Reject. |
|---|---|---|---|---|---|---|---|
| 90 L | Cz-M1 | 100 | 2000 | | 10 | 1000 | 0 |
| 90 L 2 | Cz-M1 | 100 | 2000 | | 10 | 1000 | 0 |
| 80 L | Cz-M1 | 100 | 2000 | | 10 | 1000 | 0 |
| 80 L 2 | Cz-M1 | 100 | 2000 | | 10 | 1000 | 0 |
| 70 L | Cz-M1 | 100 | 2000 | | 10 | 1000 | 0 |
| 70 L 2 | Cz-M1 | 100 | 2000 | | 10 | 1000 | 0 |
| 65 L | Cz-M1 | 100 | 2000 | | 10 | 1000 | 0 |
| 65 L 2 | Cz-M1 | 100 | 2000 | | 10 | 1000 | 0 |
| 60 L | Cz-M1 | 100 | 2000 | | 10 | 1000 | 0 |
| 60 L 2 | Cz-M1 | 100 | 2000 | | 10 | 1000 | 0 |
| 50 L | Cz-M1 | 100 | 2000 | | 10 | 1000 | 0 |
| 50 L 2 | Cz-M1 | 100 | 2000 | | 10 | 1000 | 0 |
| 40 L | Cz-M1 | 100 | 2000 | | 10 | 1000 | 0 |
| 40 L 2 | Cz-M1 | 100 | 2000 | | 10 | 1000 | 0 |
| 30 L | Cz-M1 | 100 | 2000 | | 10 | 1000 | 0 |
| 30 L 2 | Cz-M1 | 100 | 2000 | | 10 | 1000 | 0 |
| 20 L | Cz-M1 | 100 | 2000 | | 10 | 1000 | 0 |
| 20 L 2 | Cz-M1 | 100 | 2000 | | 10 | 1000 | 0 |

**ABR**: ABR 2 4000Hz 1: Cz-M1

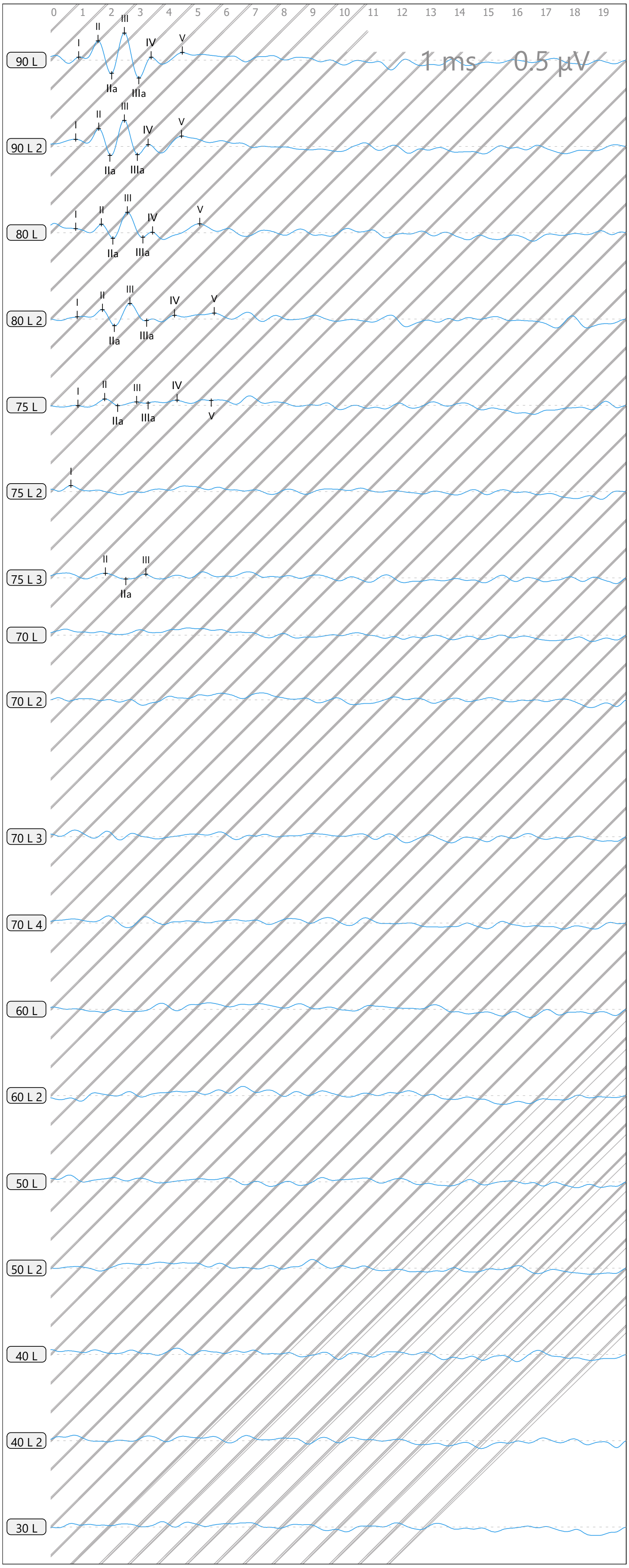
0 1 2 3 4 5 6 7 8 9 10 11 12 13 14 15 16 17 18 19
1 ms
0.5 µV
90 L
90 L 2
80 L
80 L 2
75 L
75 L 2
75 L 3
70 L
70 L 2
70 L 3
70 L 4
60 L
60 L 2
50 L
50 L 2
40 L
40 L 2
30 L
I
II
IIa
III
IIIa
IV
V

&&

| N | I (ms) | II (ms) | III (ms) | IV (ms) | V (ms) |
|---|---|---|---|---|---|
| 90 L | 0.98 | 1.64 | 2.57 | 3.49 | 4.58 |
| 90 L 2 | 0.87 | 1.67 | 2.57 | 3.39 | 4.55 |
| 80 L | 0.87 | 1.77 | 2.67 | 3.55 | 5.19 |
| 80 L 2 | 0.93 | 1.80 | 2.75 | 4.31 | 5.69 |
| 75 L | 0.95 | 1.88 | 2.99 | 4.39 | 5.58 |
| 75 L 2 | 0.71 | | | | |
| 75 L 3 | | 1.91 | 3.31 | | |

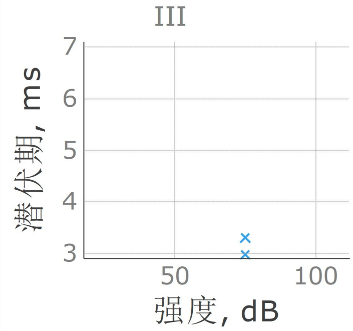


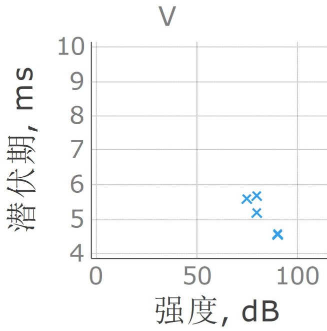


Trace parameters

| N | Electr. | HPF, Hz | LPF, Hz | 50 Hz | Rejection ±μV | Aver. | Reject. |
|---|---|---|---|---|---|---|---|
| 90 L | Cz-M1 | 200 | 2000 | | 10 | 1000 | 0 |
| 90 L 2 | Cz-M1 | 200 | 2000 | | 10 | 1000 | 0 |
| 80 L | Cz-M1 | 200 | 2000 | | 10 | 1000 | 0 |
| 80 L 2 | Cz-M1 | 200 | 2000 | | 10 | 1000 | 0 |
| 75 L | Cz-M1 | 200 | 2000 | | 10 | 1000 | 0 |
| 75 L 2 | Cz-M1 | 200 | 2000 | | 10 | 1000 | 0 |
| 75 L 3 | Cz-M1 | 200 | 2000 | | 10 | 1000 | 0 |
| 70 L | Cz-M1 | 200 | 2000 | | 10 | 1000 | 0 |
| 70 L 2 | Cz-M1 | 200 | 2000 | | 10 | 1000 | 0 |
| 70 L 3 | Cz-M1 | 200 | 2000 | | 10 | 1000 | 0 |
| 70 L 4 | Cz-M1 | 200 | 2000 | | 10 | 1000 | 0 |
| 60 L | Cz-M1 | 200 | 2000 | | 10 | 1000 | 0 |
| 60 L 2 | Cz-M1 | 200 | 2000 | | 10 | 1000 | 0 |
| 50 L | Cz-M1 | 200 | 2000 | | 10 | 1000 | 0 |
| 50 L 2 | Cz-M1 | 200 | 2000 | | 10 | 1000 | 0 |
| 40 L | Cz-M1 | 200 | 2000 | | 10 | 1000 | 0 |
| 40 L 2 | Cz-M1 | 200 | 2000 | | 10 | 1000 | 0 |
| 30 L | Cz-M1 | 200 | 2000 | | 10 | 1000 | 0 |

**ABR**: ABR 2 8000Hz

1: Cz-M1

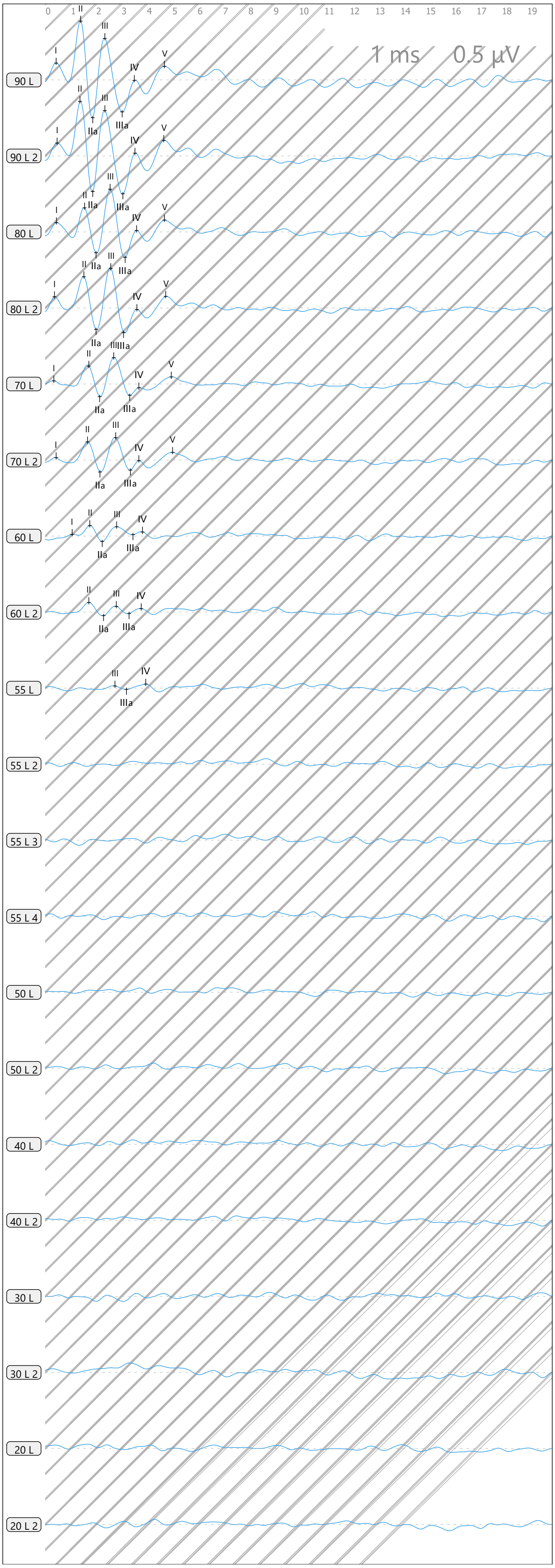
0 1 2 3 4 5 6 7 8 9 10 11 12 13 14 15 16 17 18 19
1 ms
0.5 µV
90 L
90 L 2
80 L
80 L 2
70 L
70 L 2
60 L
60 L 2
55 L
55 L 2
55 L 3
55 L 4
50 L
50 L 2
40 L
40 L 2
30 L
30 L 2
20 L
20 L 2

&&

| N | I (ms) | II (ms) | III (ms) | IV (ms) | V (ms) |
|---|---|---|---|---|---|
| 90 L | 0.42 | 1.40 | 2.35 | 3.52 | 4.71 |
| 90 L 2 | 0.48 | 1.38 | 2.35 | 3.55 | 4.68 |
| 80 L | 0.45 | 1.56 | 2.57 | 3.60 | 4.71 |
| 80 L 2 | 0.37 | 1.53 | 2.59 | 3.62 | 4.76 |
| 70 L | 0.34 | 1.72 | 2.70 | 3.70 | 4.97 |
| 70 L 2 | 0.42 | 1.67 | 2.78 | 3.70 | 5.03 |
| 60 L | 1.06 | 1.77 | 2.83 | 3.84 | |
| 60 L 2 | | 1.72 | 2.80 | 3.78 | |
| 55 L | | | 2.75 | 3.97 | |

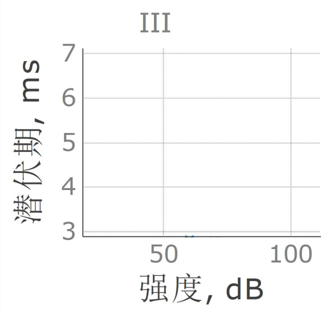


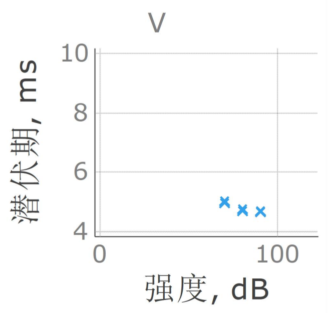


Trace parameters

| N | Electr. | HPF, Hz | LPF, Hz | 50 Hz | Rejection ±μV | Aver. | Reject. |
|---|---|---|---|---|---|---|---|
| 90 L | Cz-M1 | 200 | 2000 | | 10 | 1000 | 0 |
| 90 L 2 | Cz-M1 | 200 | 2000 | | 10 | 1000 | 0 |
| 80 L | Cz-M1 | 200 | 2000 | | 10 | 1000 | 0 |
| 80 L 2 | Cz-M1 | 200 | 2000 | | 10 | 1000 | 0 |
| 70 L | Cz-M1 | 200 | 2000 | | 10 | 1000 | 0 |
| 70 L 2 | Cz-M1 | 200 | 2000 | | 10 | 1000 | 0 |
| 60 L | Cz-M1 | 200 | 2000 | | 10 | 1000 | 0 |
| 60 L 2 | Cz-M1 | 200 | 2000 | | 10 | 1000 | 0 |
| 55 L | Cz-M1 | 200 | 2000 | | 10 | 1000 | 0 |
| 55 L 2 | Cz-M1 | 200 | 2000 | | 10 | 1000 | 0 |
| 55 L 3 | Cz-M1 | 200 | 2000 | | 10 | 1000 | 0 |
| 55 L 4 | Cz-M1 | 200 | 2000 | | 10 | 1000 | 0 |
| 50 L | Cz-M1 | 200 | 2000 | | 10 | 1000 | 0 |
| 50 L 2 | Cz-M1 | 200 | 2000 | | 10 | 1000 | 0 |
| 40 L | Cz-M1 | 200 | 2000 | | 10 | 1000 | 0 |
| 40 L 2 | Cz-M1 | 200 | 2000 | | 10 | 1000 | 0 |
| 30 L | Cz-M1 | 200 | 2000 | | 10 | 1000 | 0 |
| 30 L 2 | Cz-M1 | 200 | 2000 | | 10 | 1000 | 0 |
| 20 L | Cz-M1 | 200 | 2000 | | 10 | 1000 | 0 |

| | | | | | | | |
|---|---|---|---|---|---|---|---|
| 20 L 2 | Cz-M1 | 200 | 2000 | | 10 | 1000 | 0 |

**ABR**: ABR 2 CLICK
2: Cz-M2

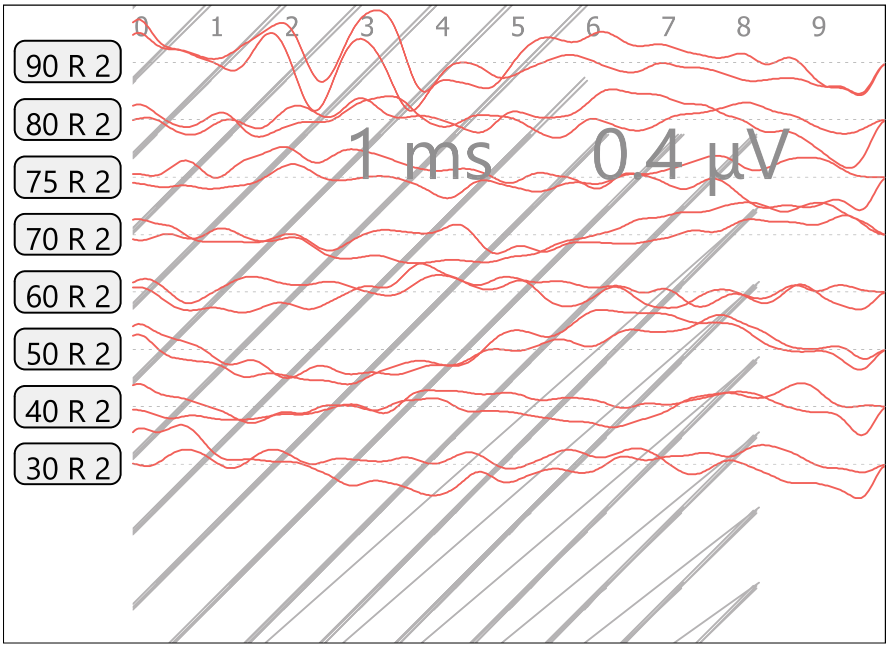


Trace parameters

| N | Electr. | HPF, Hz | LPF, Hz | 50 Hz | Rejection ±µV | Aver. | Reject |
|---|---|---|---|---|---|---|---|
| 90 R | Cz-M2 | 100 | 2000 | | 10 | 1000 | 0 |
| 90 R 2 | Cz-M2 | 100 | 2000 | | 10 | 1000 | 0 |
| 80 R | Cz-M2 | 100 | 2000 | | 10 | 1000 | 0 |
| 80 R 2 | Cz-M2 | 100 | 2000 | | 10 | 1000 | 0 |
| 75 R | Cz-M2 | 100 | 2000 | | 10 | 1000 | 0 |
| 75 R 2 | Cz-M2 | 100 | 2000 | | 10 | 1000 | 0 |
| 70 R | Cz-M2 | 100 | 2000 | | 10 | 1000 | 0 |
| 70 R 2 | Cz-M2 | 100 | 2000 | | 10 | 1000 | 0 |
| 60 R | Cz-M2 | 100 | 2000 | | 10 | 1000 | 0 |
| 60 R 2 | Cz-M2 | 100 | 2000 | | 10 | 1000 | 0 |
| 50 R | Cz-M2 | 100 | 2000 | | 10 | 1000 | 0 |
| 50 R 2 | Cz-M2 | 100 | 2000 | | 10 | 1000 | 0 |
| 40 R | Cz-M2 | 100 | 2000 | | 10 | 1000 | 0 |
| 40 R 2 | Cz-M2 | 100 | 2000 | | 10 | 1000 | 0 |
| 30 R | Cz-M2 | 100 | 2000 | | 10 | 1000 | 0 |
| 30 R 2 | Cz-M2 | 100 | 2000 | | 10 | 1000 | 0 |

**ABR**: ABR 2 4000Hz 2: Cz-M2

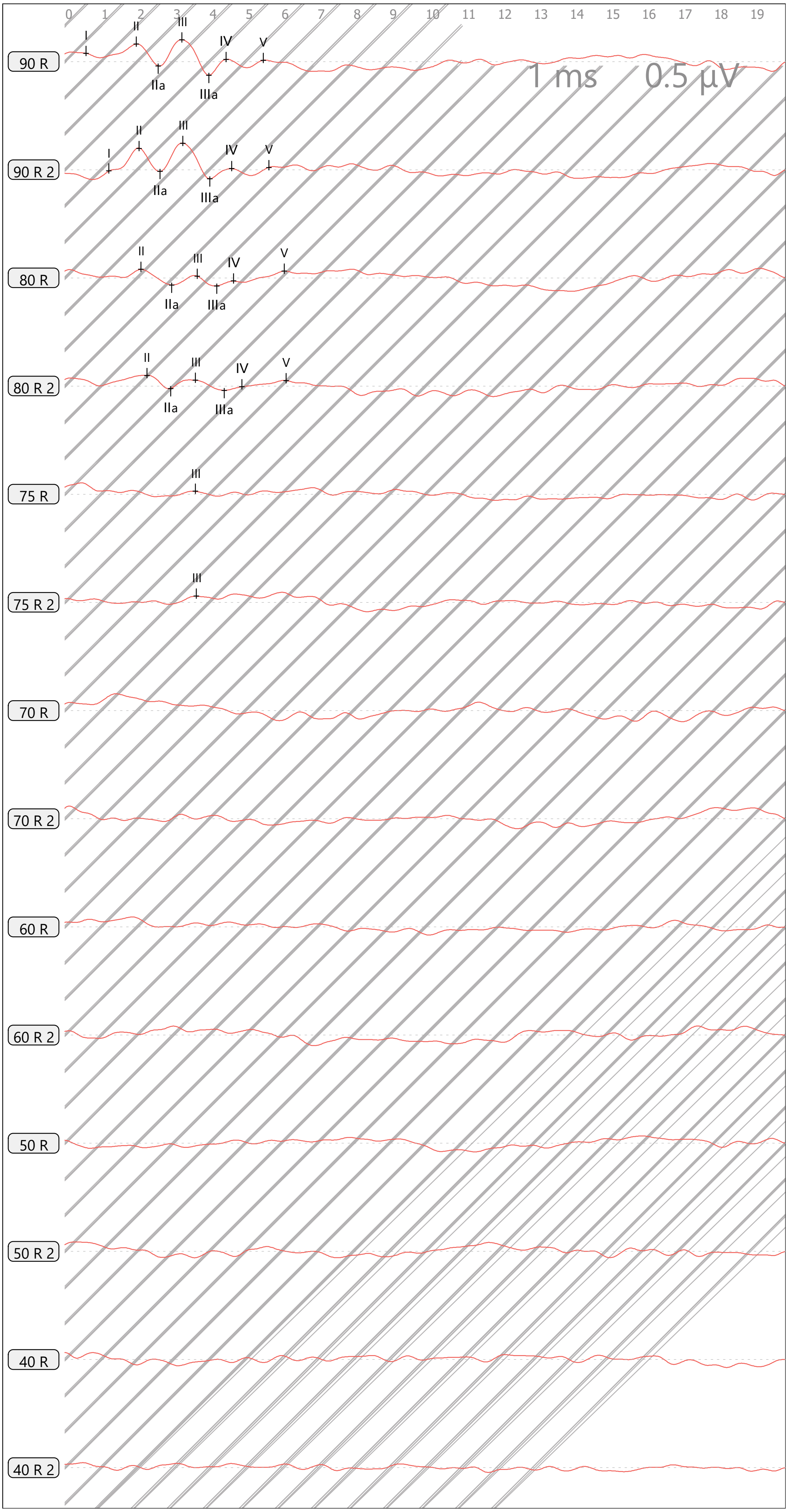
0 1 2 3 4 5 6 7 8 9 10 11 12 13 14 15 16 17 18 19
1 ms
0.5 µV
90 R
90 R 2
80 R
80 R 2
75 R
75 R 2
70 R
70 R 2
60 R
60 R 2
50 R
50 R 2
40 R
40 R 2
I
II
IIa
III
IIIa
IV
V

| IV (ms) | V (ms) | I–III (ms) | I–V (ms) | III–V (ms) | |
|---|---|---|---|---|---|
| 4.47 | 5.50 | 2.67 | 4.92 | 2.25 | |
| 4.63 | 5.66 | 2.06 | 4.45 | 2.38 | |
| 4.68 | 6.09 | | | 2.41 | |
| 4.92 | 6.14 | | | 2.51 | |
| | | | | | |
| | | | | | |

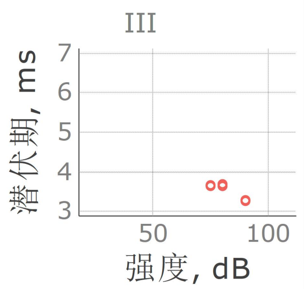


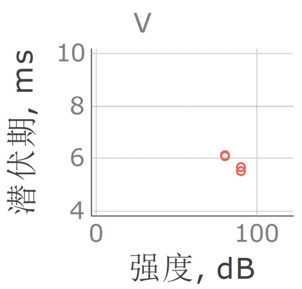


Trace parameters

| N | Electr. | HPF, Hz | LPF, Hz | 50 Hz | Rejection ±μV | Aver. | Reject |
|---|---|---|---|---|---|---|---|
| 90 R | Cz-M2 | 200 | 2000 | | 10 | 1000 | 0 |
| 90 R 2 | Cz-M2 | 200 | 2000 | | 10 | 1000 | 0 |
| 80 R | Cz-M2 | 200 | 2000 | | 10 | 1000 | 0 |
| 80 R 2 | Cz-M2 | 200 | 2000 | | 10 | 1000 | 0 |
| 75 R | Cz-M2 | 200 | 2000 | | 10 | 1000 | 0 |
| 75 R 2 | Cz-M2 | 200 | 2000 | | 10 | 1000 | 0 |
| 70 R | Cz-M2 | 200 | 2000 | | 10 | 1000 | 0 |
| 70 R 2 | Cz-M2 | 200 | 2000 | | 10 | 1000 | 0 |
| 60 R | Cz-M2 | 200 | 2000 | | 10 | 1000 | 0 |
| 60 R 2 | Cz-M2 | 200 | 2000 | | 10 | 1000 | 0 |
| 50 R | Cz-M2 | 200 | 2000 | | 10 | 1000 | 0 |
| 50 R 2 | Cz-M2 | 200 | 2000 | | 10 | 1000 | 0 |
| 40 R | Cz-M2 | 200 | 2000 | | 10 | 1000 | 0 |
| 40 R 2 | Cz-M2 | 200 | 2000 | | 10 | 1000 | 0 |

**ABR**: ABR 2 8000Hz 2: Cz-M2

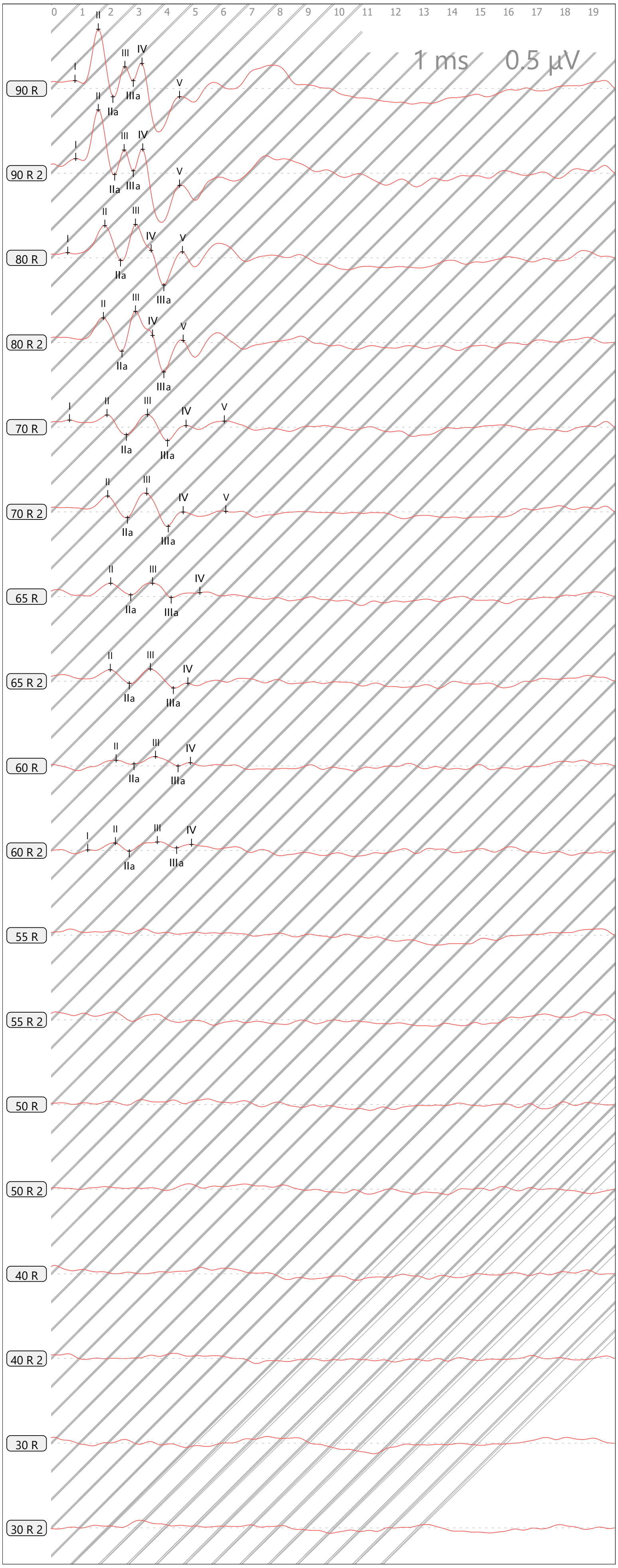
0 1 2 3 4 5 6 7 8 9 10 11 12 13 14 15 16 17 18 19
1 ms
0.5 µV
90 R
90 R 2
80 R
80 R 2
70 R
70 R 2
65 R
65 R 2
60 R
60 R 2
55 R
55 R 2
50 R
50 R 2
40 R
40 R 2
30 R
30 R 2

| IV (ms) | V (ms) | I–III (ms) | I–V (ms) | III–V (ms) |
|---|---|---|---|---|
| 3.23 | 4.55 | 1.77 | 3.70 | 1.93 |
| 3.25 | 4.55 | 1.72 | 3.68 | 1.96 |
| 3.55 | 4.66 | 2.41 | 4.07 | 1.67 |
| 3.60 | 4.68 | | | 1.69 |
| 4.79 | 6.14 | 2.75 | 5.48 | 2.73 |
| 4.68 | 6.19 | | | 2.80 |
| 5.27 | | | | |
| 4.84 | | | | |
| 4.95 | | | | |
| 4.97 | | 2.46 | | |

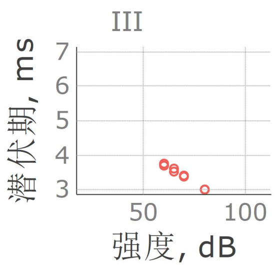


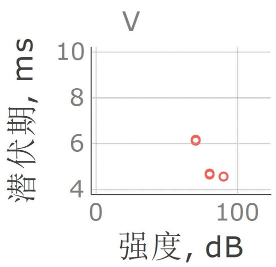


Trace parameters

| N | Electr. | HPF, Hz | LPF, Hz | 50 Hz | Rejection ±µV | Aver. | Reject |
|---|---|---|---|---|---|---|---|
| 90 R | Cz-M2 | 200 | 2000 | | 10 | 1000 | 0 |
| 90 R 2 | Cz-M2 | 200 | 2000 | | 10 | 1000 | 0 |
| 80 R | Cz-M2 | 200 | 2000 | | 10 | 1000 | 0 |
| 80 R 2 | Cz-M2 | 200 | 2000 | | 10 | 1000 | 0 |
| 70 R | Cz-M2 | 200 | 2000 | | 10 | 1000 | 0 |
| 70 R 2 | Cz-M2 | 200 | 2000 | | 10 | 1000 | 0 |
| 65 R | Cz-M2 | 200 | 2000 | | 10 | 1000 | 0 |
| 65 R 2 | Cz-M2 | 200 | 2000 | | 10 | 1000 | 0 |
| 60 R | Cz-M2 | 200 | 2000 | | 10 | 1000 | 0 |
| 60 R 2 | Cz-M2 | 200 | 2000 | | 10 | 1000 | 0 |
| 55 R | Cz-M2 | 200 | 2000 | | 10 | 1000 | 0 |
| 55 R 2 | Cz-M2 | 200 | 2000 | | 10 | 1000 | 0 |
| 50 R | Cz-M2 | 200 | 2000 | | 10 | 1000 | 0 |
| 50 R 2 | Cz-M2 | 200 | 2000 | | 10 | 1000 | 0 |
| 40 R | Cz-M2 | 200 | 2000 | | 10 | 1000 | 0 |
| 40 R 2 | Cz-M2 | 200 | 2000 | | 10 | 1000 | 0 |
| 30 R | Cz-M2 | 200 | 2000 | | 10 | 1000 | 0 |
| 30 R 2 | Cz-M2 | 200 | 2000 | | 10 | 1000 | 0 |

**ECochG**: ECochG
1: Fpz-M1

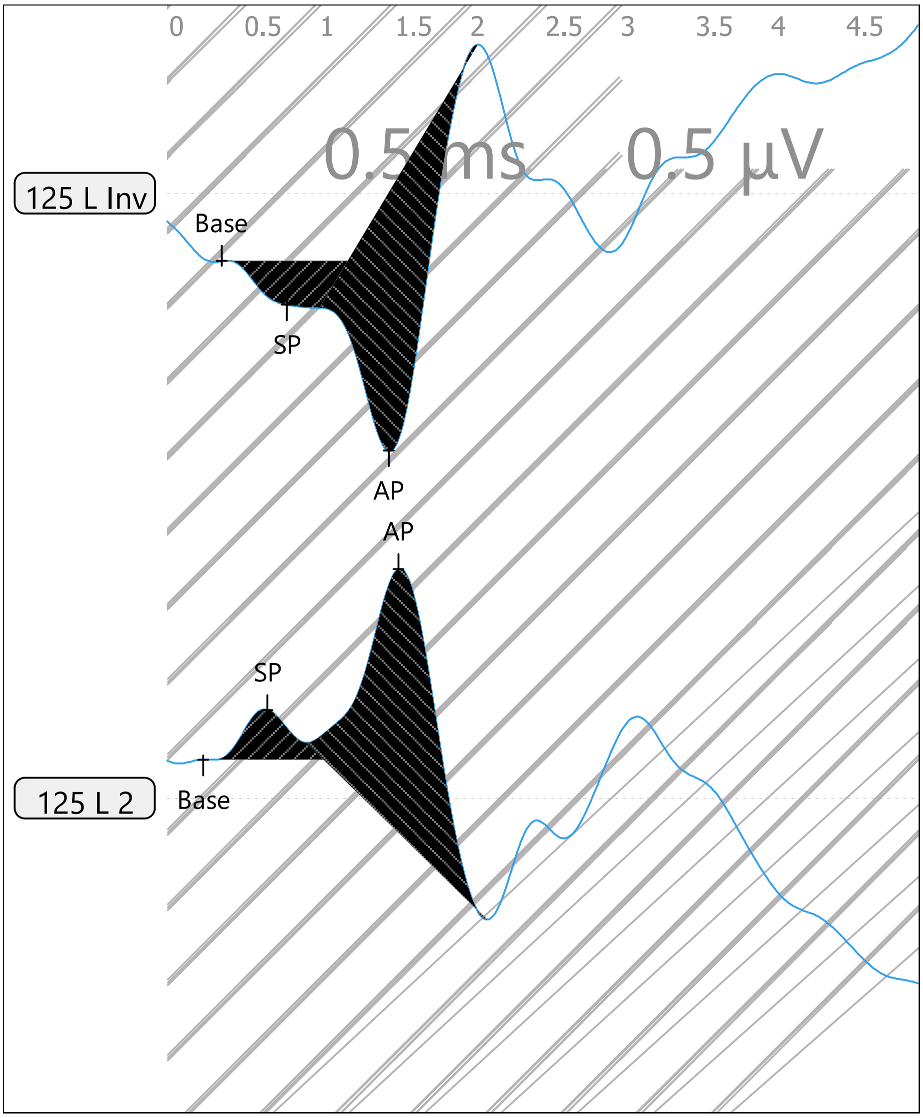


&&

| N | Base (ms) | SP (ms) | AP (ms) | SP–Base (ms) | AP–Base (ms) | SP–Base (µV) | AP–Base (µV) | |
|---|---|---|---|---|---|---|---|---|
| 125 L Inv | 0.36 | 0.79 | 1.47 | 0.44 | 1.11 | 0.29 | 1.26 | 0.2[illegible] |
| 125 L 2 | 0.24 | 0.66 | 1.53 | 0.42 | 1.30 | 0.33 | 1.27 | 0.2[illegible] |

Trace parameters

| N | Electr. | HPF, Hz | LPF, Hz | 50 Hz | Rejection ±µV | Aver. | Rej[illegible] |
|---|---|---|---|---|---|---|---|
| 125 L Inv | Fpz-M1 | 5 | 2000 | | 50 | 1500 | 1[illegible] |
| 125 L 2 | Fpz-M1 | 5 | 2000 | | 50 | 1500 | 1[illegible] |

**ECochG**: ECochG 2:
Fpz-M2

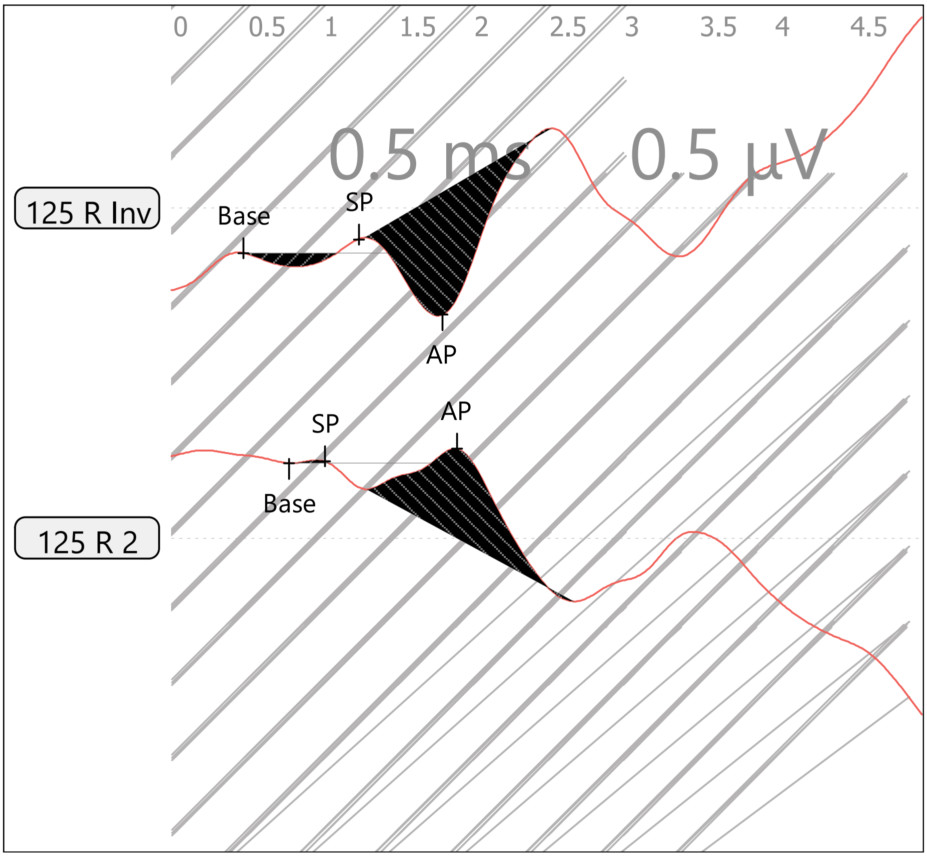


&&

| N | Base (ms) | SP (ms) | AP (ms) | SP–Base (ms) | AP–Base (ms) | SP–Base (µV) | AP–Base (µV) | [illegible] |
|---|---|---|---|---|---|---|---|---|
| 125 R Inv | 0.48 | 1.24 | 1.80 | 0.77 | 1.32 | 0.09 | 0.41 | 0.2 |
| 125 R 2 | 0.78 | 1.02 | 1.89 | 0.24 | 1.11 | 0.01 | 0.09 | 0.1 |

Trace parameters

| N | Electr. | HPF, Hz | LPF, Hz | 50 Hz | Rejection ±µV | Aver. | Rej |
|---|---|---|---|---|---|---|---|
| 125 R Inv | Fpz-M2 | 5 | 2000 | | 50 | 1500 | 14 |
| 125 R 2 | Fpz-M2 | 5 | 2000 | | 50 | 1500 | 11 |

**CONCLUSION:**

**Doctor:**
