# Supplementary material for: Exposure to high-altitude hypobaric hypoxic environment induces low-frequency hearing loss in C57BL/6J mice: Mediated by slowing down the postsynaptic electrical signal transmission speed in the cochlear-inferior colliculus auditory signaling pathway
Source: PLoS One. 2026 Mar 11;21(3):e0342321. doi: 10.1371/journal.pone.0342321 (PMC12978441; doi:10.1371/journal.pone.0342321)
Supplement: S1 File — (ZIP) [file pone.0342321.s001.zip › 2025-6-18-20d-4.pdf]

Exam report

Patient: 2025-6-18-20d-4**,** - (-)
Date: June 18, 2025

ABR: ABR 2 CLICK
1: Cz-M1

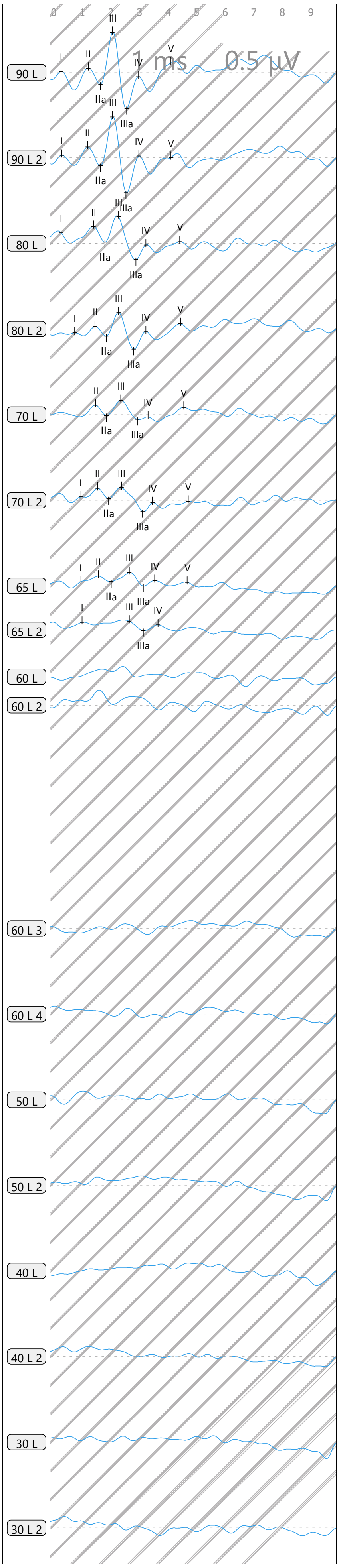

0 1 2 3 4 5 6 7 8 9
1 ms
0.5 µV
90 L
90 L 2
80 L
80 L 2
70 L
70 L 2
65 L
65 L 2
60 L
60 L 2
60 L 3
60 L 4
50 L
50 L 2
40 L
40 L 2
30 L
30 L 2
I
II
IIa
III
IIIa
IV
V

latency&& amplitude (左耳

N	I (ms)	II (ms)	III (ms)	IV (ms)	V (ms)
90 L	0.37	1.32	2.17	3.07	4.21
90 L 2	0.40	1.30	2.17	3.10	4.21
80 L	0.37	1.51	2.38	3.33	4.52
80 L 2	0.85	1.56	2.38	3.33	4.55
70 L		1.59	2.46	3.41	4.66
70 L 2	1.06	1.64	2.49	3.57	4.82
65 L	1.06	1.67	2.75	3.65	4.79
65 L 2	1.11		2.75	3.76	

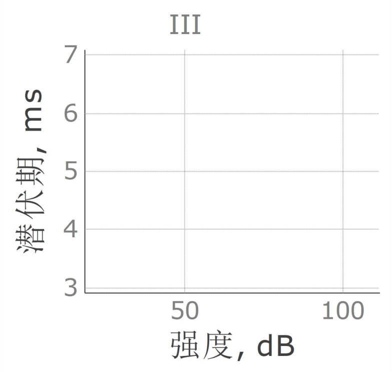

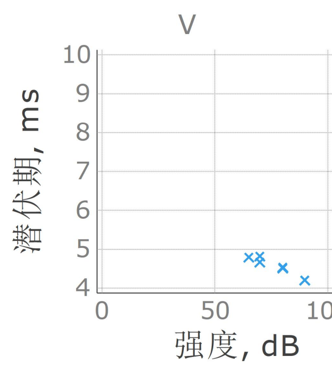

Trace parameters

N	Electr.	HPF, Hz	LPF, Hz	50 Hz	Rejection ±μV	Aver.	Reject.
90 L	Cz-M1	100	2000		10	1000	0
90 L 2	Cz-M1	100	2000		10	1000	0
80 L	Cz-M1	100	2000		10	1000	0
80 L 2	Cz-M1	100	2000		10	1000	0
70 L	Cz-M1	100	2000		10	1000	0
70 L 2	Cz-M1	100	2000		10	1000	0
65 L	Cz-M1	100	2000		10	1000	0
65 L 2	Cz-M1	100	2000		10	1000	0
60 L	Cz-M1	100	2000		10	1000	0
60 L 2	Cz-M1	100	2000		10	1000	0
60 L 3	Cz-M1	100	2000		10	1000	0
60 L 4	Cz-M1	100	2000		10	1000	0
50 L	Cz-M1	100	2000		10	1000	0
50 L 2	Cz-M1	100	2000		10	1000	0
40 L	Cz-M1	100	2000		10	1000	0
40 L 2	Cz-M1	100	2000		10	1000	0
30 L	Cz-M1	100	2000		10	1000	0
30 L 2	Cz-M1	100	2000		10	1000	0

ABR: ABR 2 4000Hz 1: Cz-M1

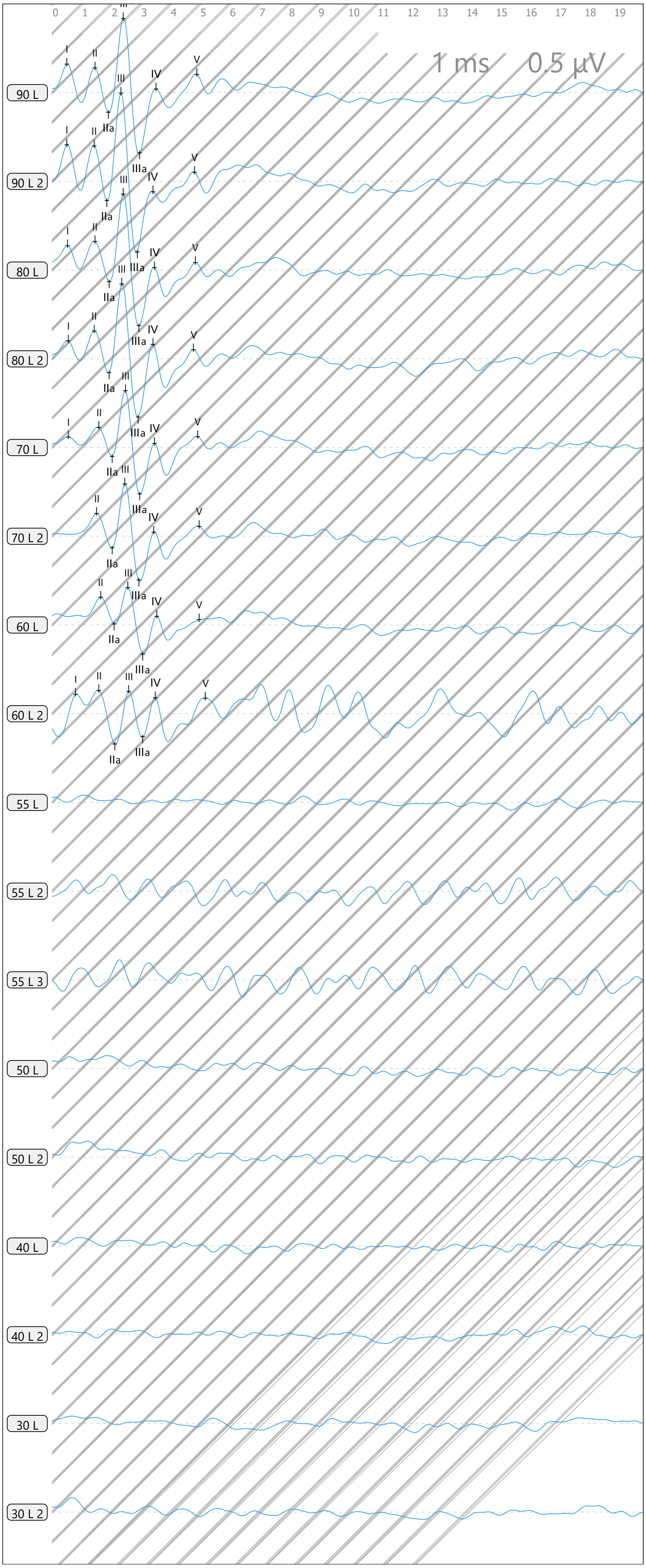
0 1 2 3 4 5 6 7 8 9 10 11 12 13 14 15 16 17 18 19
1 ms
0.5 µV
90 L
90 L 2
80 L
80 L 2
70 L
70 L 2
60 L
60 L 2
55 L
55 L 2
55 L 3
50 L
50 L 2
40 L
40 L 2
30 L
30 L 2
I
II
IIa
III
IIIa
IV
V

&&

N	I (ms)	II (ms)	III (ms)	IV (ms)	V (ms)
90 L	0.50	1.46	2.41	3.52	4.89
90 L 2	0.50	1.43	2.33	3.41	4.82
80 L	0.53	1.46	2.41	3.47	4.84
80 L 2	0.56	1.43	2.35	3.41	4.79
70 L	0.56	1.59	2.49	3.47	4.92
70 L 2		1.51	2.46	3.44	4.97
60 L		1.64	2.57	3.55	4.97
60 L 2	0.79	1.59	2.59	3.49	5.19

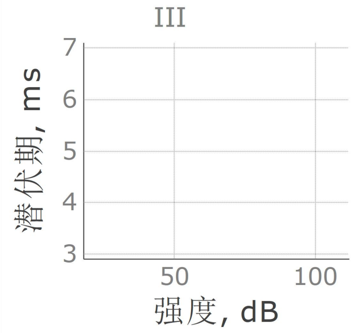

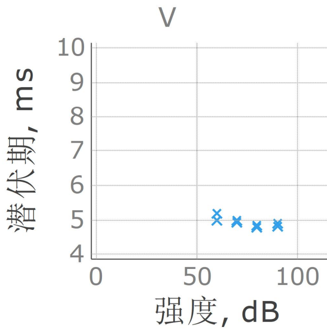

Trace parameters

N	Electr.	HPF, Hz	LPF, Hz	50 Hz	Rejection ±μV	Aver.	Reject.
90 L	Cz-M1	200	2000		10	1000	0
90 L 2	Cz-M1	200	2000		10	1000	0
80 L	Cz-M1	200	2000		10	1000	0
80 L 2	Cz-M1	200	2000		10	1000	0
70 L	Cz-M1	200	2000		10	1000	0
70 L 2	Cz-M1	200	2000		10	1000	0
60 L	Cz-M1	200	2000		10	1000	0
60 L 2	Cz-M1	200	2000		10	1000	0
55 L	Cz-M1	200	2000		10	1000	0
55 L 2	Cz-M1	200	2000		10	1000	0
55 L 3	Cz-M1	200	2000		10	1000	0
50 L	Cz-M1	200	2000		10	1000	0
50 L 2	Cz-M1	200	2000		10	1000	0
40 L	Cz-M1	200	2000		10	1000	0
40 L 2	Cz-M1	200	2000		10	1000	0
30 L	Cz-M1	200	2000		10	1000	0
30 L 2	Cz-M1	200	2000		10	1000	0

ABR: ABR 2 8000Hz 1: Cz-M1

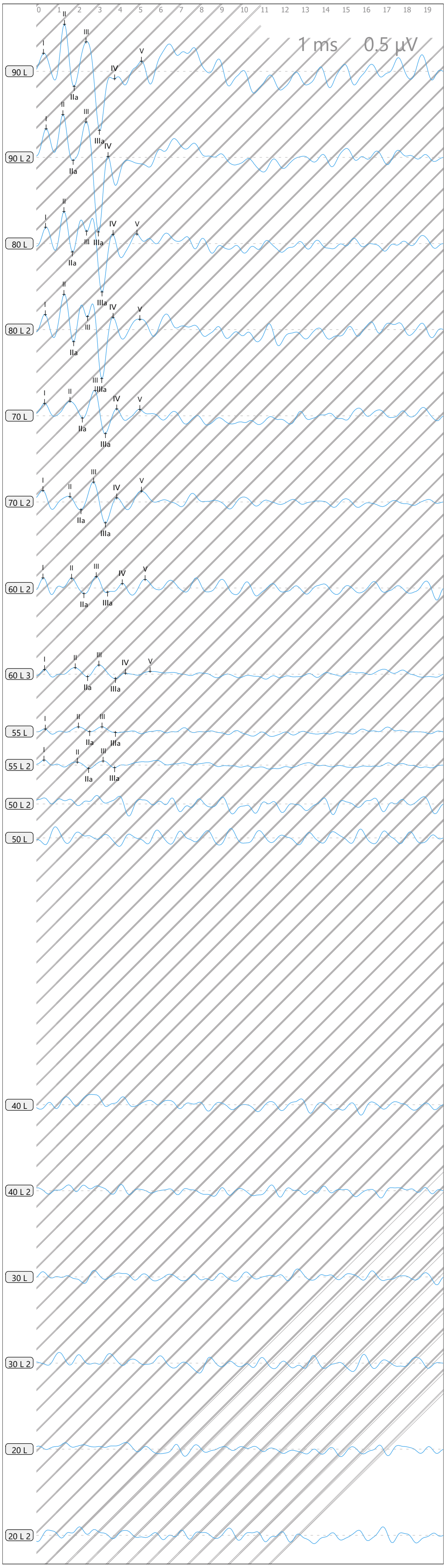
0 1 2 3 4 5 6 7 8 9 10 11 12 13 14 15 16 17 18 19
1 ms
0.5 µV
90 L
90 L 2
80 L
80 L 2
70 L
70 L 2
60 L 2
60 L 3
55 L
55 L 2
50 L 2
50 L
40 L
40 L 2
30 L
30 L 2
20 L
20 L 2
I
II
IIa
III
IIIa
IV
V

&&

N	I (ms)	II (ms)	III (ms)	IV (ms)	V (ms)
90 L	0.34	1.38	2.43	3.84	5.16
90 L 2	0.48	1.30	2.43	3.52	
80 L	0.45	1.35	2.46	3.76	4.95
80 L 2	0.42	1.35	2.51	3.76	5.08
70 L	0.40	1.64	2.88	3.94	5.08
70 L 2	0.32	1.64	2.80	3.94	5.16
60 L 2	0.32	1.72	2.94	4.21	5.34
60 L 3	0.40	1.91	3.07	4.37	5.58
55 L	0.42	2.06	3.23		
55 L 2	0.37	2.01	3.28		

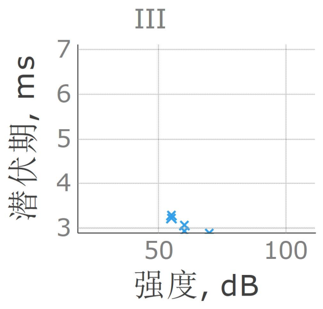

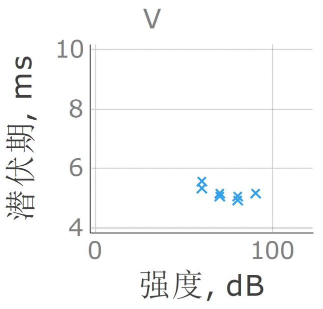

Trace parameters

N	Electr.	HPF, Hz	LPF, Hz	50 Hz	Rejection ±μV	Aver.	Reject.
90 L	Cz-M1	200	2000		10	1000	0
90 L 2	Cz-M1	200	2000		10	1000	0
80 L	Cz-M1	200	2000		10	1000	0
80 L 2	Cz-M1	200	2000		10	1000	0
70 L	Cz-M1	200	2000		10	1000	0
70 L 2	Cz-M1	200	2000		10	1000	0
60 L 2	Cz-M1	200	2000		10	1000	0
60 L 3	Cz-M1	200	2000		10	1000	0
55 L	Cz-M1	200	2000		10	1000	0
55 L 2	Cz-M1	200	2000		10	1000	0
50 L	Cz-M1	200	2000		10	1000	0
50 L 2	Cz-M1	200	2000		10	1000	0
40 L	Cz-M1	200	2000		10	1000	0
40 L 2	Cz-M1	200	2000		10	1000	0
30 L	Cz-M1	200	2000		10	1000	0
30 L 2	Cz-M1	200	2000		10	1000	0
20 L	Cz-M1	200	2000		10	1000	0
20 L 2	Cz-M1	200	2000		10	1000	0

ABR: ABR CLICK 2: Cz-M2

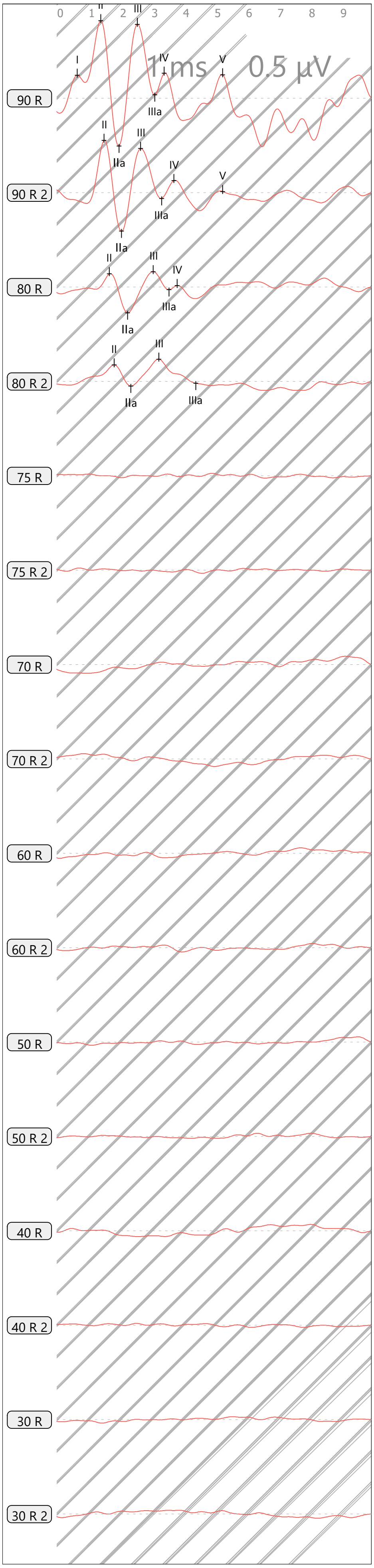
0 1 2 3 4 5 6 7 8 9
1 ms
0.5 µV
90 R
90 R 2
80 R
80 R 2
75 R
75 R 2
70 R
70 R 2
60 R
60 R 2
50 R
50 R 2
40 R
40 R 2
30 R
30 R 2
I
II
IIa
III
IIIa
IV
V

IV (ms)	V (ms)	I–III (ms)	I–V (ms)	III–V (ms)	
3.41	5.27	1.91	4.60	2.70	
3.73	5.27			2.59	
3.84					

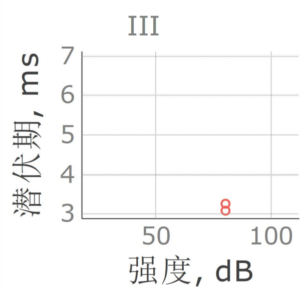

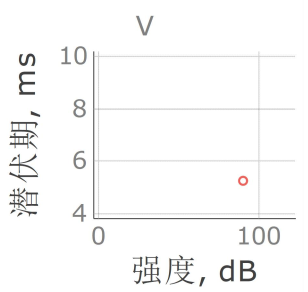

Trace parameters

N	Electr.	HPF, Hz	LPF, Hz	50 Hz	Rejection ±μV	Aver.	Reject
90 R	Cz-M2	100	2000		10	1000	0
90 R 2	Cz-M2	100	2000		10	1000	0
80 R	Cz-M2	100	2000		10	1000	0
80 R 2	Cz-M2	100	2000		10	1000	0
75 R	Cz-M2	100	2000		10	1000	0
75 R 2	Cz-M2	100	2000		10	1000	0
70 R	Cz-M2	100	2000		10	1000	0
70 R 2	Cz-M2	100	2000		10	1000	0
60 R	Cz-M2	100	2000		10	1000	0
60 R 2	Cz-M2	100	2000		10	1000	0
50 R	Cz-M2	100	2000		10	1000	0
50 R 2	Cz-M2	100	2000		10	1000	0
40 R	Cz-M2	100	2000		10	1000	0
40 R 2	Cz-M2	100	2000		10	1000	0
30 R	Cz-M2	100	2000		10	1000	0
30 R 2	Cz-M2	100	2000		10	1000	0

ABR: ABR 2 4000Hz 2: Cz-M2

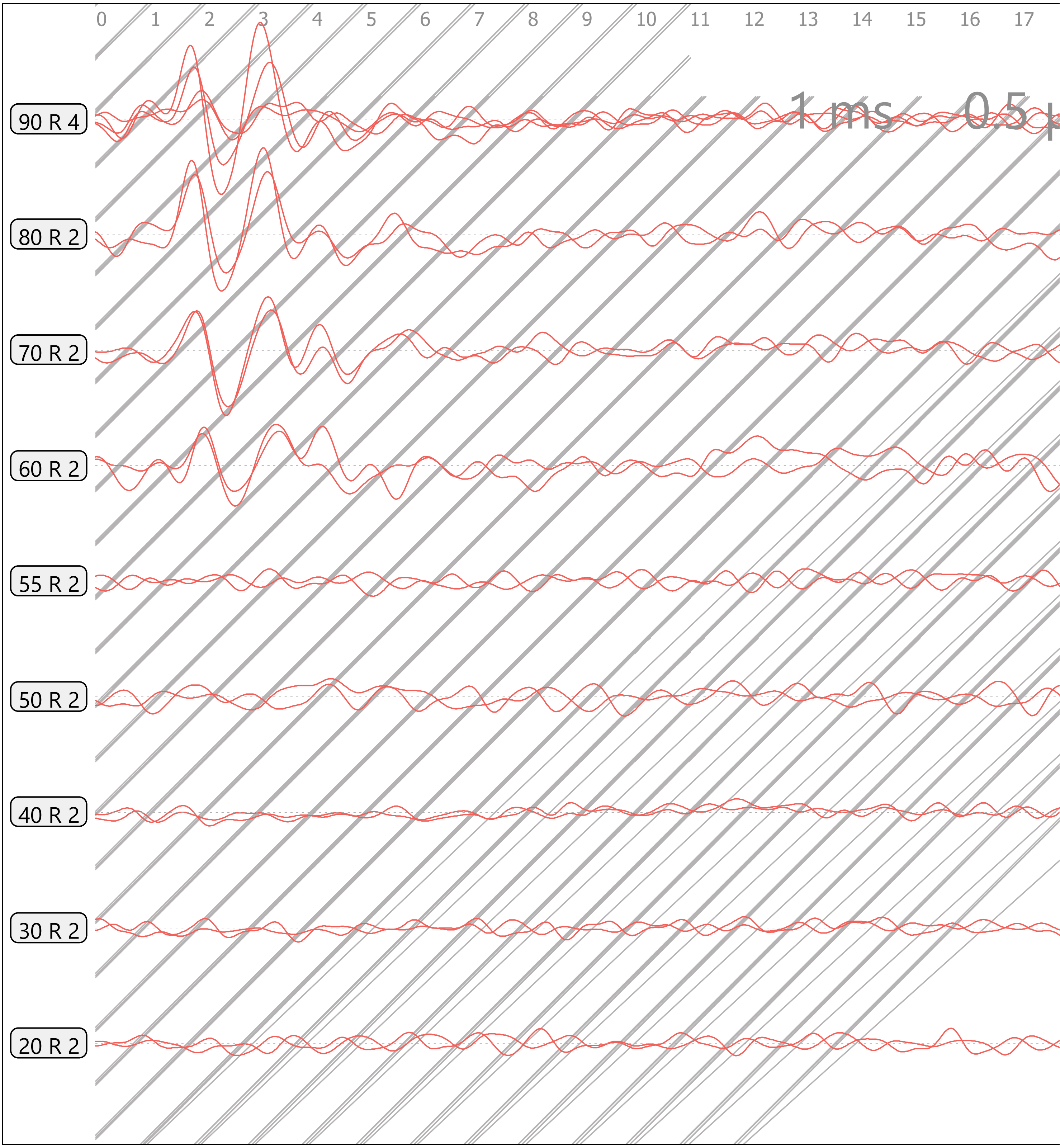

Trace parameters

N	Electr.	HPF, Hz	LPF, Hz	50 Hz	Rejection ±μV	Aver.	Reject
90 R	Cz-M2	200	2000		10	1000	0
90 R 2	Cz-M2	200	2000		10	1000	0
90 R 3	Cz-M2	200	2000		10	1000	0
90 R 4	Cz-M2	200	2000		10	1000	0
80 R	Cz-M2	200	2000		10	1000	0

80 R 2	Cz-M2	200	2000		10	1000	0
70 R	Cz-M2	200	2000		10	1000	0
70 R 2	Cz-M2	200	2000		10	1000	0
60 R	Cz-M2	200	2000		10	1000	0
60 R 2	Cz-M2	200	2000		10	1000	0
55 R	Cz-M2	200	2000		10	1000	0
55 R 2	Cz-M2	200	2000		10	1000	0
50 R	Cz-M2	200	2000		10	1000	0
50 R 2	Cz-M2	200	2000		10	1000	0
40 R	Cz-M2	200	2000		10	1000	0
40 R 2	Cz-M2	200	2000		10	1000	0
30 R	Cz-M2	200	2000		10	1000	0
30 R 2	Cz-M2	200	2000		10	1000	0
20 R	Cz-M2	200	2000		10	1000	0
20 R 2	Cz-M2	200	2000		10	1000	0

ABR: ABR 2 8000Hz 2: Cz-M2

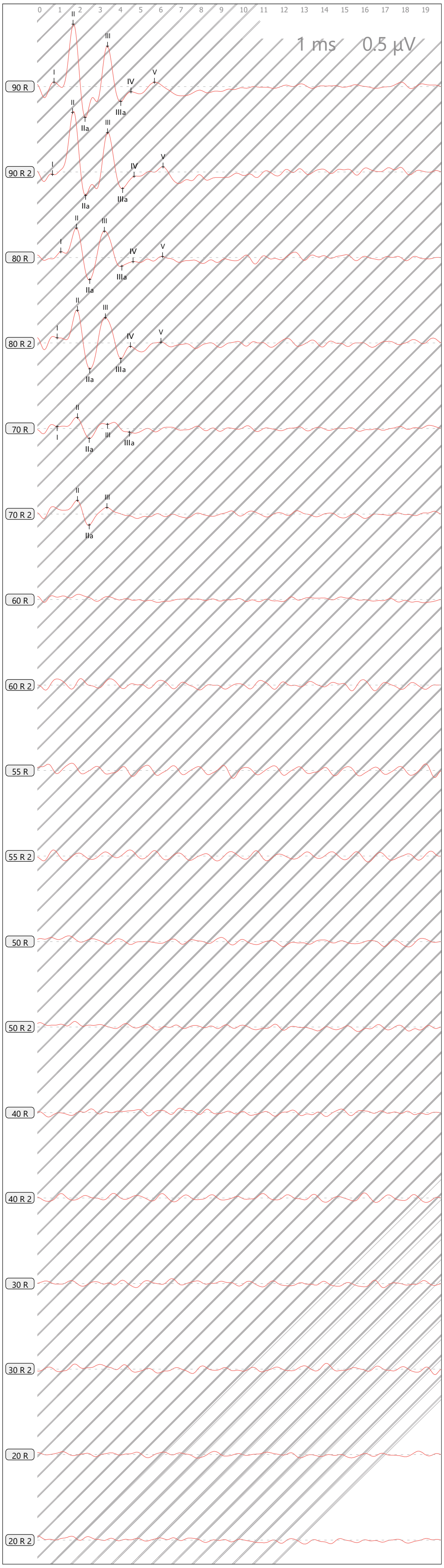
0 1 2 3 4 5 6 7 8 9 10 11 12 13 14 15 16 17 18 19
1 ms
0.5 µV
90 R
90 R 2
80 R
80 R 2
70 R
70 R 2
60 R
60 R 2
55 R
55 R 2
50 R
50 R 2
40 R
40 R 2
30 R
30 R 2
20 R
20 R 2
I
II
IIa
III
IIIa
IV
V

Ⅳ (ms)	V (ms)	I–III (ms)	I–V (ms)	III–V (ms)	
4.63	5.79	2.65	4.97	2.33	
4.79	6.22	2.73	5.48	2.75	
4.74	6.19	2.14	5.03	2.88	
4.60	6.11	2.38	5.13	2.75	
		2.49			

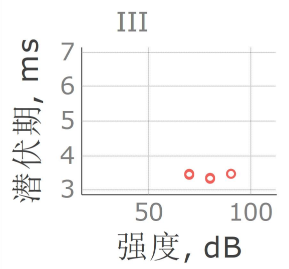

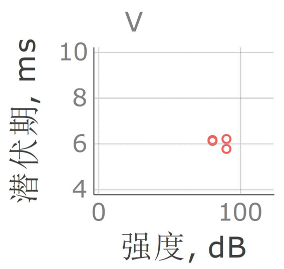

Trace parameters

N	Electr.	HPF, Hz	LPF, Hz	50 Hz	Rejection ±μV	Aver.	Reject
90 R	Cz-M2	200	2000		10	1000	0
90 R 2	Cz-M2	200	2000		10	1000	0
80 R	Cz-M2	200	2000		10	1000	0
80 R 2	Cz-M2	200	2000		10	1000	0
70 R	Cz-M2	200	2000		10	1000	0
70 R 2	Cz-M2	200	2000		10	1000	0
60 R	Cz-M2	200	2000		10	1000	0
60 R 2	Cz-M2	200	2000		10	1000	0
55 R	Cz-M2	200	2000		10	1000	0
55 R 2	Cz-M2	200	2000		10	1000	0
50 R	Cz-M2	200	2000		10	1000	0
50 R 2	Cz-M2	200	2000		10	1000	0
40 R	Cz-M2	200	2000		10	1000	0
40 R 2	Cz-M2	200	2000		10	1000	0
30 R	Cz-M2	200	2000		10	1000	0
30 R 2	Cz-M2	200	2000		10	1000	0
20 R	Cz-M2	200	2000		10	1000	0
20 R 2	Cz-M2	200	2000		10	1000	0

ECochG: ECochG 1:
Fpz-M1

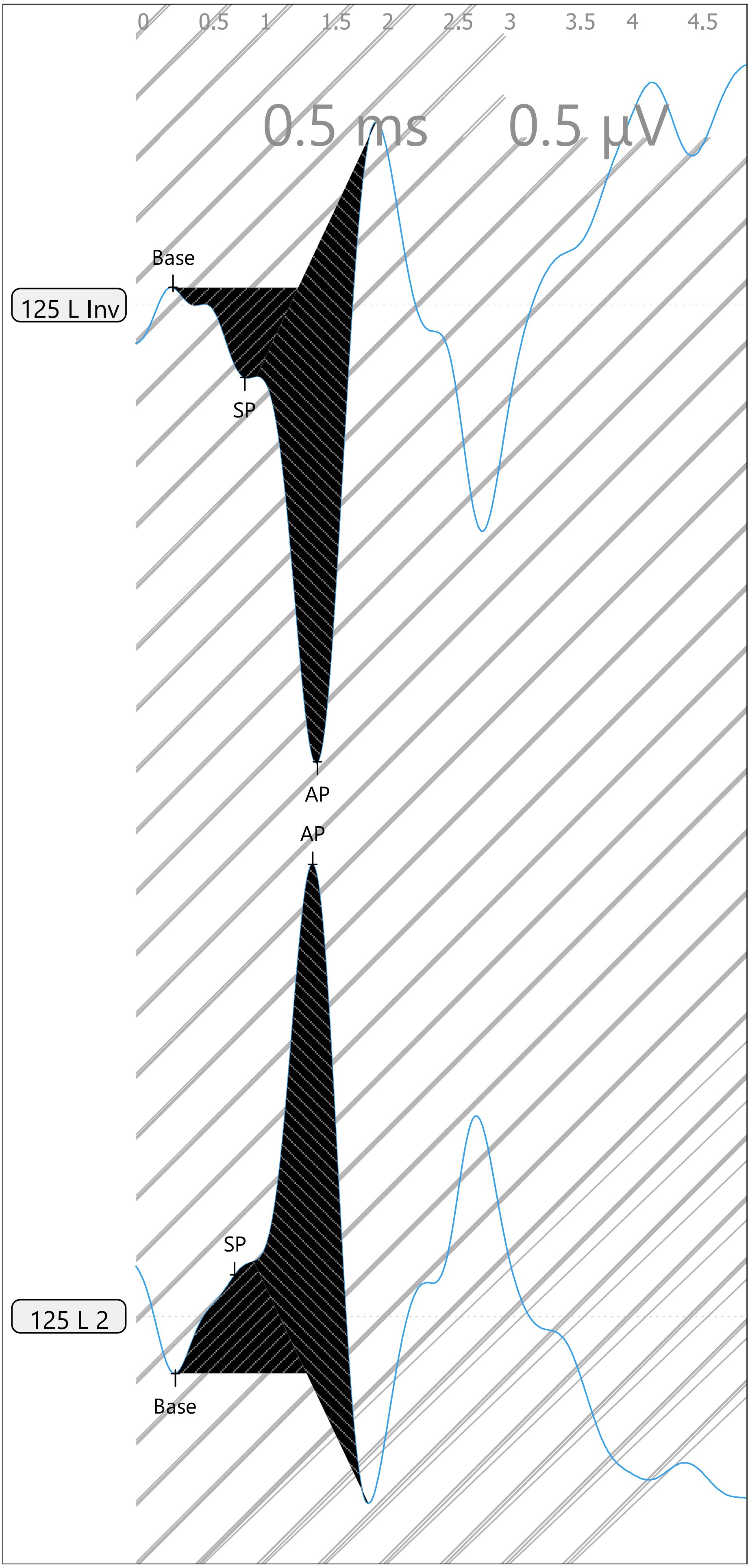
0
0.5
1
1.5
2
2.5
3
3.5
4
4.5
0.5 ms
0.5 µV
Base
125 L Inv
SP
AP
AP
SP
125 L 2
Base

&&

N	Base (ms)	SP (ms)	AP (ms)	SP–Base (ms)	AP–Base (ms)	SP–Base (µV)	AP–Base (µV)	[illegible]
125 L Inv	0.30	0.89	1.48	0.58	1.18	0.73	3.88	0.1[illegible]
125 L 2	0.32	0.81	1.44	0.49	1.12	0.81	4.16	0.1[illegible]

Trace parameters

N	Electr.	HPF, Hz	LPF, Hz	50 Hz	Rejection ±µV	Aver.	Rej[illegible]
125 L Inv	Fpz-M1	5	2000		50	1211	4[illegible]
125 L 2	Fpz-M1	5	2000		50	1042	2[illegible]

ECochG: ECochG 2:
Fpz-M2

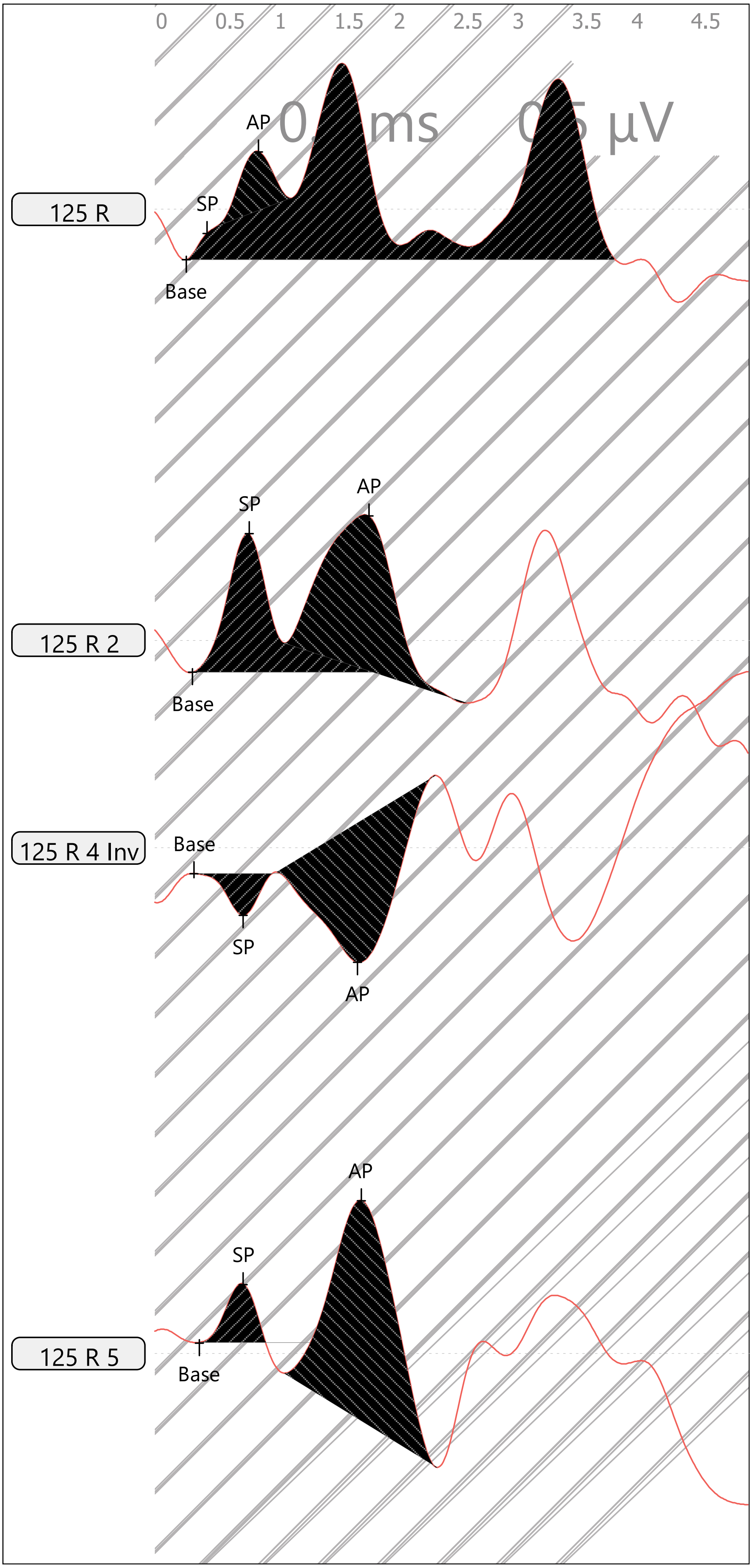

0
0.5
1
1.5
2
2.5
3
3.5
4
4.5
ms
µV
125 R
SP
AP
Base
125 R 2
SP
AP
Base
125 R 4 Inv
Base
SP
AP
125 R 5
SP
AP
Base

&&

N	Base (ms)	SP (ms)	AP (ms)	SP–Base (ms)	AP–Base (ms)	SP–Base (µV)	AP–Base (µV)	
125 R	0.26	0.44	0.87	0.17	0.61	0.22	0.91	0
125 R 2	0.32	0.79	1.80	0.48	1.48	1.15	1.31	0
125 R 4 Inv	0.33	0.74	1.71	0.41	1.38	0.35	0.75	0
125 R 5	0.37	0.74	1.73	0.37	1.36	0.49	1.19	0

Trace parameters

N	Electr.	HPF, Hz	LPF, Hz	50 Hz	Rejection ±µV	Aver.	R
125 R	Fpz-M2	5	2000		50	1500	
125 R 2	Fpz-M2	5	2000		50	1070	
125 R 4 Inv	Fpz-M2	5	2000		50	1185	
125 R 5	Fpz-M2	5	2000		50	1168	

CONCLUSION:

Doctor:
