# Supplementary material for: Exposure to high-altitude hypobaric hypoxic environment induces low-frequency hearing loss in C57BL/6J mice: Mediated by slowing down the postsynaptic electrical signal transmission speed in the cochlear-inferior colliculus auditory signaling pathway
Source: PLoS One. 2026 Mar 11;21(3):e0342321. doi: 10.1371/journal.pone.0342321 (PMC12978441; doi:10.1371/journal.pone.0342321)
Supplement: S1 File — (ZIP) [file pone.0342321.s001.zip › 2025-6-20-30d-1.pdf]

Exam report

Patient: 2025-6-20-30d-1**,** - (-)
Date: June 20, 2025

ABR: ABR 2 CLICK
1: Cz-M1

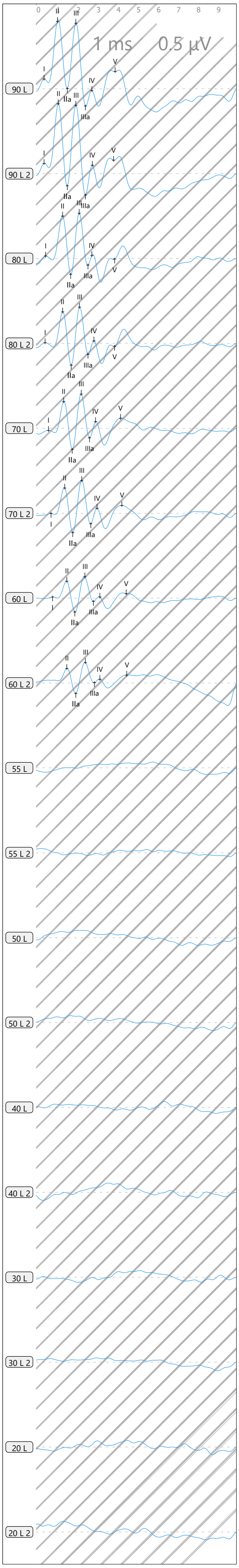
0 1 2 3 4 5 6 7 8 9
1 ms
0.5 µV
90 L
90 L 2
80 L
80 L 2
70 L
70 L 2
60 L
60 L 2
55 L
55 L 2
50 L
50 L 2
40 L
40 L 2
30 L
30 L 2
20 L
20 L 2

latency&& 波幅 (left ear

N	I (ms)	II (ms)	III (ms)	IV (ms)	V (ms)
90 L	0.40	1.06	1.98	2.78	3.94
90 L 2	0.40	1.11	1.98	2.80	3.86
80 L	0.48	1.32	2.14	2.78	3.92
80 L 2	0.45	1.32	2.17	2.88	3.92
70 L	0.61	1.38	2.25	2.96	4.21
70 L 2	0.74	1.43	2.28	3.04	4.29
60 L	0.82	1.53	2.43	3.18	4.50
60 L 2		1.53	2.46	3.18	4.52

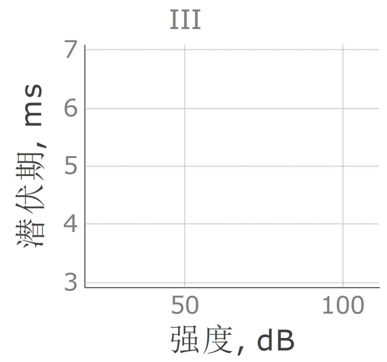

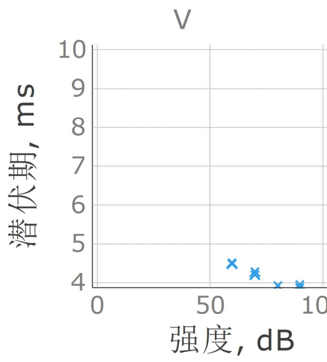

Trace parameters

N	Electr.	HPF, Hz	LPF, Hz	50 Hz	Rejection ±μV	Aver.	Reject.
90 L	Cz-M1	100	2000		10	1000	0
90 L 2	Cz-M1	100	2000		10	1000	0
80 L	Cz-M1	100	2000		10	1000	0
80 L 2	Cz-M1	100	2000		10	1000	0
70 L	Cz-M1	100	2000		10	1000	0
70 L 2	Cz-M1	100	2000		10	1000	0
60 L	Cz-M1	100	2000		10	1000	0
60 L 2	Cz-M1	100	2000		10	1000	0
55 L	Cz-M1	100	2000		10	1000	0
55 L 2	Cz-M1	100	2000		10	1000	0
50 L	Cz-M1	100	2000		10	1000	0
50 L 2	Cz-M1	100	2000		10	1000	0
40 L	Cz-M1	100	2000		10	804	0
40 L 2	Cz-M1	100	2000		10	394	0
30 L	Cz-M1	100	2000		10	494	0
30 L 2	Cz-M1	100	2000		10	982	0
20 L	Cz-M1	100	2000		10	305	0
20 L 2	Cz-M1	100	2000		10	359	0

ABR: ABR 2 4000Hz 1: Cz-M1

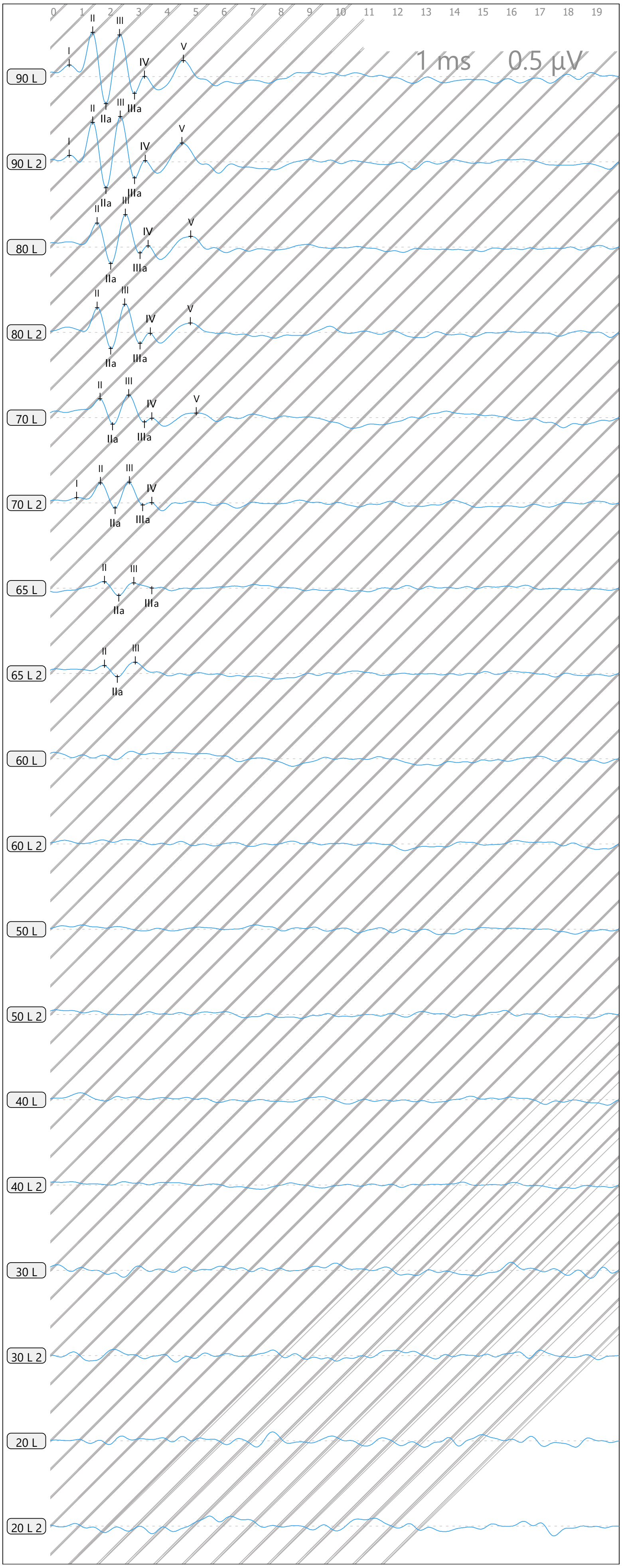

0 1 2 3 4 5 6 7 8 9 10 11 12 13 14 15 16 17 18 19
1 ms
0.5 µV
90 L
90 L 2
80 L
80 L 2
70 L
70 L 2
65 L
65 L 2
60 L
60 L 2
50 L
50 L 2
40 L
40 L 2
30 L
30 L 2
20 L
20 L 2
I
II
IIa
III
IIIa
IV
V

N	I (ms)	II (ms)	III (ms)	Ⅳ (ms)	V (ms)
90 L	0.66	1.48	2.43	3.31	4.68
90 L 2	0.66	1.48	2.46	3.33	4.63
80 L		1.64	2.65	3.44	4.95
80 L 2		1.64	2.62	3.52	4.92
70 L		1.75	2.75	3.57	5.13
70 L 2	0.93	1.77	2.78	3.57	
65 L		1.91	2.94		
65 L 2		1.91	2.99		

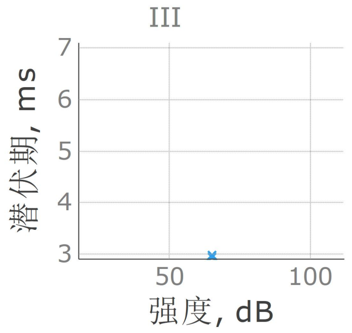

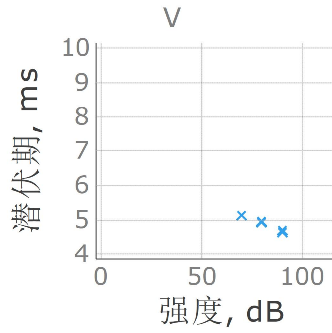

Trace parameters

N	Electr.	HPF, Hz	LPF, Hz	50 Hz	Rejection ±μV	Aver.	Reject.
90 L	Cz-M1	200	2000		10	1000	0
90 L 2	Cz-M1	200	2000		10	1000	0
80 L	Cz-M1	200	2000		10	1000	0
80 L 2	Cz-M1	200	2000		10	1000	0
70 L	Cz-M1	200	2000		10	1000	0
70 L 2	Cz-M1	200	2000		10	1000	0
65 L	Cz-M1	200	2000		10	1000	0
65 L 2	Cz-M1	200	2000		10	1000	0
60 L	Cz-M1	200	2000		10	1000	0
60 L 2	Cz-M1	200	2000		10	1000	0
50 L	Cz-M1	200	2000		10	1000	0
50 L 2	Cz-M1	200	2000		10	1000	0
40 L	Cz-M1	200	2000		10	1000	0
40 L 2	Cz-M1	200	2000		10	928	0
30 L	Cz-M1	200	2000		10	248	0
30 L 2	Cz-M1	200	2000		10	318	0
20 L	Cz-M1	200	2000		10	198	0
20 L 2	Cz-M1	200	2000		10	231	0

ABR: ABR 2 8000Hz 1: Cz-M1

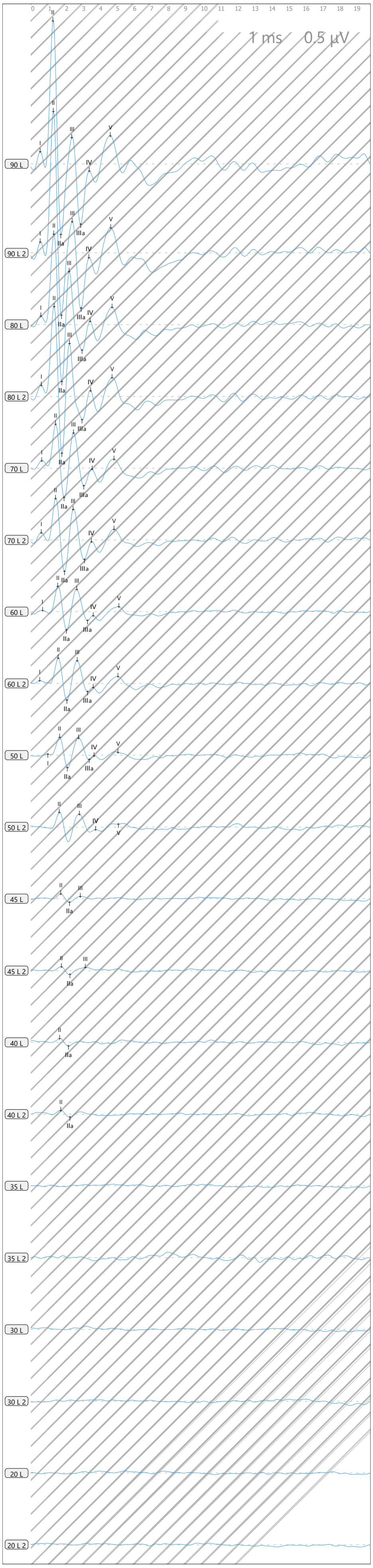
0 1 2 3 4 5 6 7 8 9 10 11 12 13 14 15 16 17 18 19
1 ms
0,5 µV
90 L
90 L 2
80 L
80 L 2
70 L
70 L 2
60 L
60 L 2
50 L
50 L 2
45 L
45 L 2
40 L
40 L 2
35 L
35 L 2
30 L
30 L 2
20 L
20 L 2
I
II
IIa
III
IIIa
IV
V

&&

N	I (ms)	II (ms)	III (ms)	IV (ms)	V (ms)
90 L	0.56	1.30	2.41	3.44	4.68
90 L 2	0.56	1.32	2.43	3.41	4.71
80 L	0.58	1.35	2.25	3.49	4.79
80 L 2	0.61	1.38	2.28	3.52	4.79
70 L	0.64	1.46	2.51	3.60	4.89
70 L 2	0.61	1.46	2.49	3.57	4.89
60 L	0.69	1.59	2.70	3.68	5.19
60 L 2	0.53	1.61	2.73	3.68	5.13
50 L	1.01	1.69	2.80	3.73	5.13
50 L 2		1.67	2.86	3.81	5.16
45 L		1.77	2.91		
45 L 2		1.80	3.20		
40 L		1.69			
40 L 2		1.77			

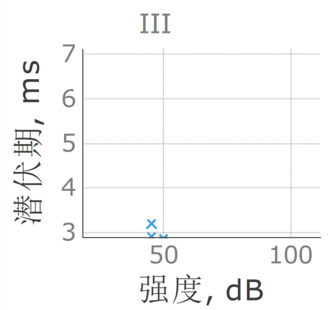

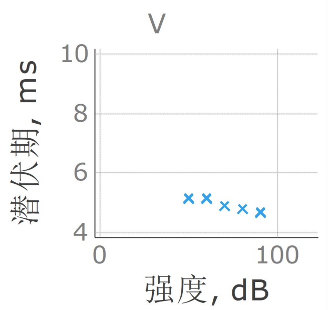

Trace parameters

N	Electr.	HPF, Hz	LPF, Hz	50 Hz	Rejection ±μV	Aver.	Reject.
90 L	Cz-M1	200	2000		10	1000	0
90 L 2	Cz-M1	200	2000		10	1000	0
80 L	Cz-M1	200	2000		10	1000	0
80 L 2	Cz-M1	200	2000		10	1000	0
70 L	Cz-M1	200	2000		10	1000	0
70 L 2	Cz-M1	200	2000		10	1000	0
60 L	Cz-M1	200	2000		10	1000	0
60 L 2	Cz-M1	200	2000		10	1000	0
50 L	Cz-M1	200	2000		10	1000	0
50 L 2	Cz-M1	200	2000		10	1000	0
45 L	Cz-M1	200	2000		10	1000	0
45 L 2	Cz-M1	200	2000		10	1000	0
40 L	Cz-M1	200	2000		10	1000	0
40 L 2	Cz-M1	200	2000		10	1000	0
35 L	Cz-M1	200	2000		10	1000	0
35 L 2	Cz-M1	200	2000		10	236	0

30 L	Cz-M1	200	2000		10	1000	0
30 L 2	Cz-M1	200	2000		10	1000	0
20 L	Cz-M1	200	2000		10	1000	0
20 L 2	Cz-M1	200	2000		10	1000	0

ABR: ABR 2 CLICK 2: Cz-M2

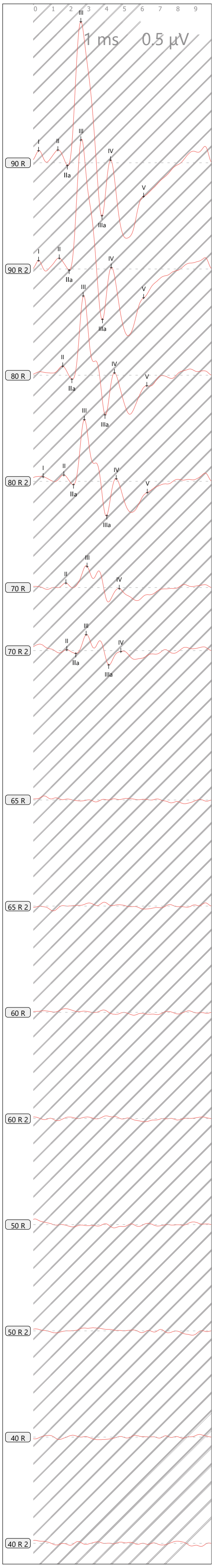

0 1 2 3 4 5 6 7 8 9
1 ms
0.5 µV
90 R
90 R 2
80 R
80 R 2
70 R
70 R 2
65 R
65 R 2
60 R
60 R 2
50 R
50 R 2
40 R
40 R 2
I
II
IIa
III
IIIa
IV
V

IV (ms)	V (ms)	I–III (ms)	I–V (ms)	III–V (ms)	
4.34	6.19	2.38	5.90	3.52	
4.37	6.19	2.38	5.90	3.52	
4.55	6.38			3.57	
4.66	6.40	2.30	5.85	3.55	
4.82					
4.92					

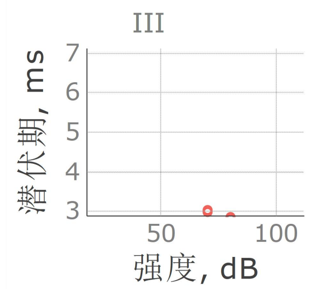

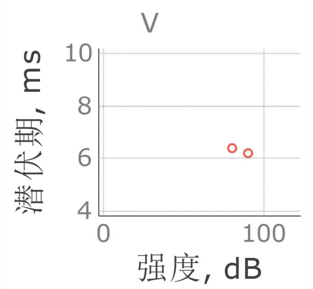

Trace parameters

N	Electr.	HPF, Hz	LPF, Hz	50 Hz	Rejection ±μV	Aver.	Reject
90 R	Cz-M2	100	2000		10	1000	0
90 R 2	Cz-M2	100	2000		10	1000	0
80 R	Cz-M2	100	2000		10	1000	0
80 R 2	Cz-M2	100	2000		10	1000	0
70 R	Cz-M2	100	2000		10	1000	0
70 R 2	Cz-M2	100	2000		10	1000	0
65 R	Cz-M2	100	2000		10	1000	0
65 R 2	Cz-M2	100	2000		10	1000	0
60 R	Cz-M2	100	2000		10	1000	0
60 R 2	Cz-M2	100	2000		10	1000	0
50 R	Cz-M2	100	2000		10	1000	0
50 R 2	Cz-M2	100	2000		10	1000	0
40 R	Cz-M2	100	2000		10	1000	0
40 R 2	Cz-M2	100	2000		10	1000	0

ABR: ABR 2 4000Hz 2: Cz-M2

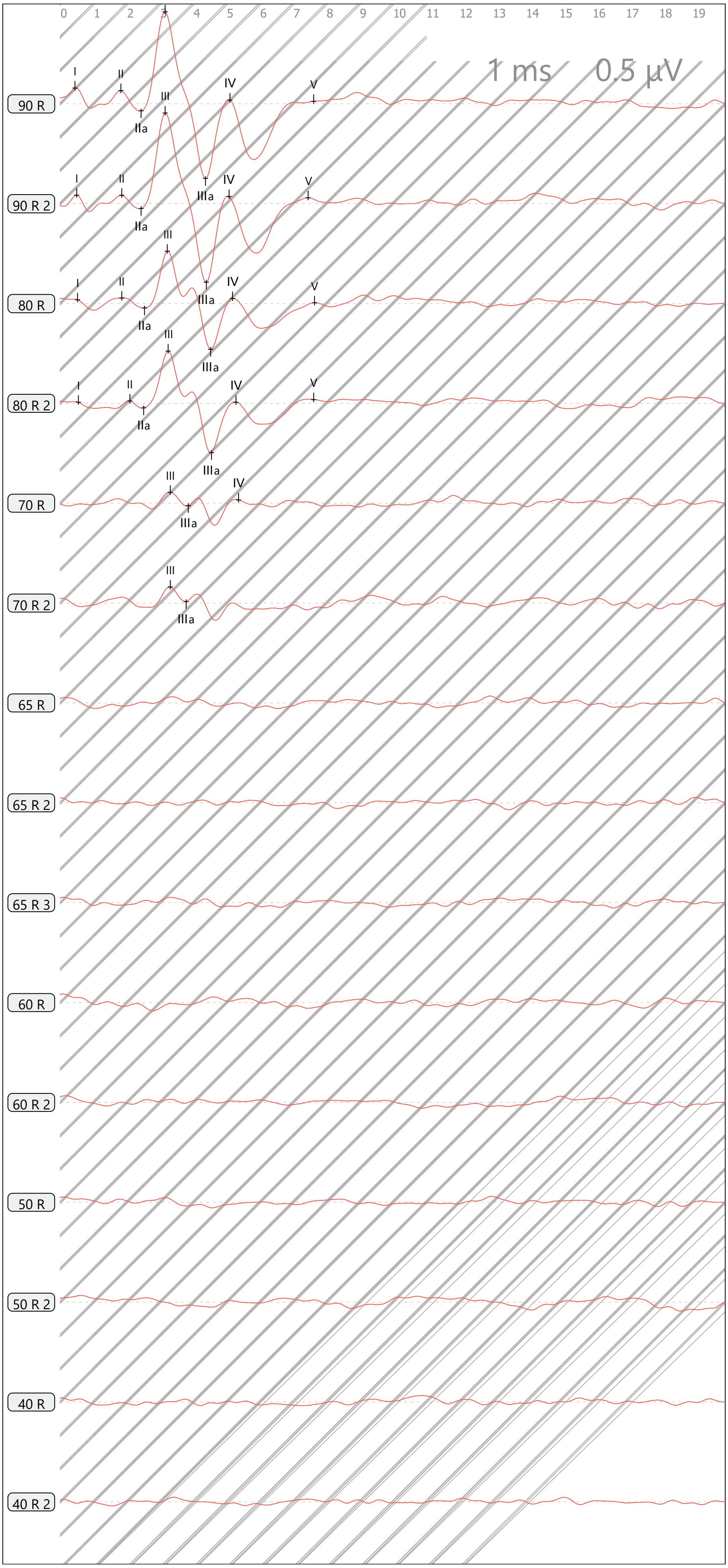

0 1 2 3 4 5 6 7 8 9 10 11 12 13 14 15 16 17 18 19
1 ms
0.5 µV
90 R
90 R 2
80 R
80 R 2
70 R
70 R 2
65 R
65 R 2
65 R 3
60 R
60 R 2
50 R
50 R 2
40 R
40 R 2
I
II
IIa
III
IIIa
IV
V

IV (ms)	V (ms)	I–III (ms)	I–V (ms)	III–V (ms)	
5.11	7.62	2.70	7.17	4.47	
5.08	7.46	2.65	6.96	4.31	
5.19	7.65	2.70	7.12	4.42	
5.29	7.62	2.70	7.06	4.37	
5.37					

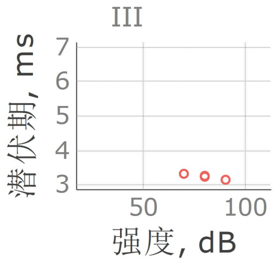

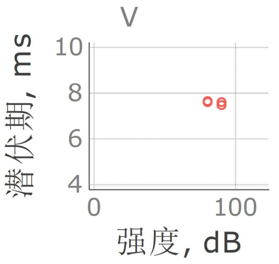

Trace parameters

N	Electr.	HPF, Hz	LPF, Hz	50 Hz	Rejection ±μV	Aver.	Reject
90 R	Cz-M2	200	2000		10	1000	0
90 R 2	Cz-M2	200	2000		10	1000	0
80 R	Cz-M2	200	2000		10	1000	0
80 R 2	Cz-M2	200	2000		10	1000	0
70 R	Cz-M2	200	2000		10	1000	0
70 R 2	Cz-M2	200	2000		10	1000	0
65 R	Cz-M2	200	2000		10	1000	0
65 R 2	Cz-M2	200	2000		10	1000	0
65 R 3	Cz-M2	200	2000		10	1000	0
60 R	Cz-M2	200	2000		10	1000	0
60 R 2	Cz-M2	200	2000		10	1000	0
50 R	Cz-M2	200	2000		10	1000	0
50 R 2	Cz-M2	200	2000		10	1000	0
40 R	Cz-M2	200	2000		10	1000	0
40 R 2	Cz-M2	200	2000		10	1000	0

ABR: ABR 2 8000Hz 2: Cz-M2

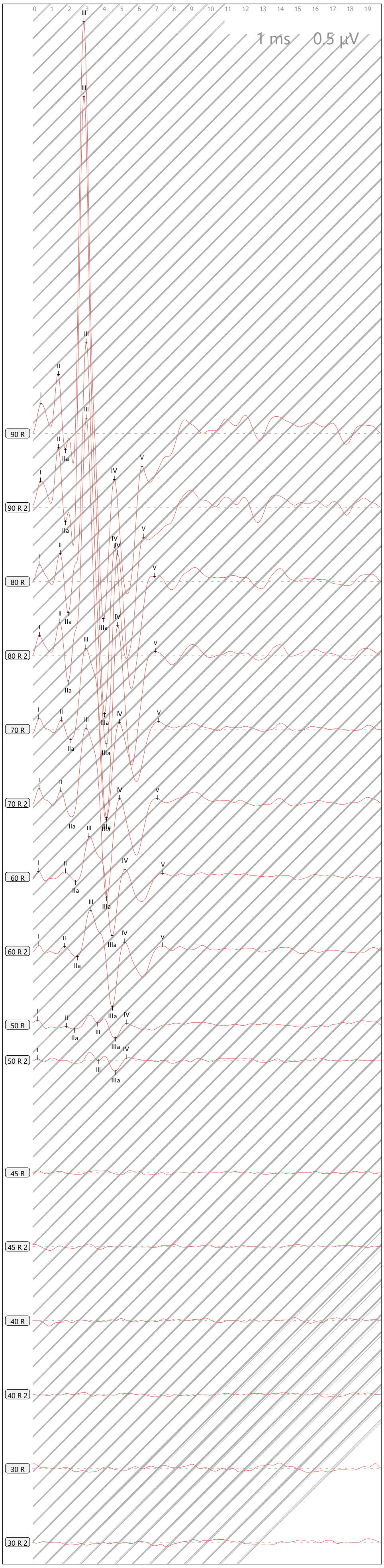
0 1 2 3 4 5 6 7 8 9 10 11 12 13 14 15 16 17 18 19
1 ms 0.5 µV
90 R
90 R 2
80 R
80 R 2
70 R
70 R 2
60 R
60 R 2
50 R
50 R 2
45 R
45 R 2
40 R
40 R 2
30 R
30 R 2

IV (ms)	V (ms)	I–III (ms)	I–V (ms)	III–V (ms)	
4.68	6.27	2.46	5.79	3.33	
4.71	6.35	2.49	5.90	3.41	
4.87	6.99	2.70	6.61	3.92	
4.87	7.04	2.67	6.64	3.97	
4.97	7.22	2.70	6.88	4.18	
4.97	7.14	2.70	6.77	4.07	
5.29	7.46	2.91	7.14	4.23	
5.27	7.43	3.02	7.12	4.10	
5.40		3.44			
5.37		3.47			

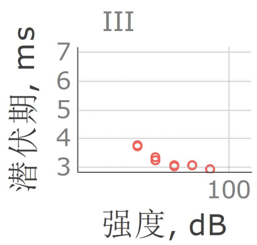

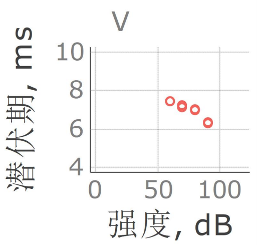

Trace parameters

N	Electr.	HPF, Hz	LPF, Hz	50 Hz	Rejection ±μV	Aver.	Reject.
90 R	Cz-M2	200	2000		10	1000	0
90 R 2	Cz-M2	200	2000		10	1000	0
80 R	Cz-M2	200	2000		10	1000	0
80 R 2	Cz-M2	200	2000		10	1000	0
70 R	Cz-M2	200	2000		10	1000	0
70 R 2	Cz-M2	200	2000		10	1000	0
60 R	Cz-M2	200	2000		10	1000	0
60 R 2	Cz-M2	200	2000		10	1000	0
50 R	Cz-M2	200	2000		10	1000	0
50 R 2	Cz-M2	200	2000		10	1000	0
45 R	Cz-M2	200	2000		10	1000	0
45 R 2	Cz-M2	200	2000		10	1000	0
40 R	Cz-M2	200	2000		10	1000	0
40 R 2	Cz-M2	200	2000		10	1000	0
30 R	Cz-M2	200	2000		10	568	0
30 R 2	Cz-M2	200	2000		10	660	0

EcochG: ECochG 1: Cz-M1

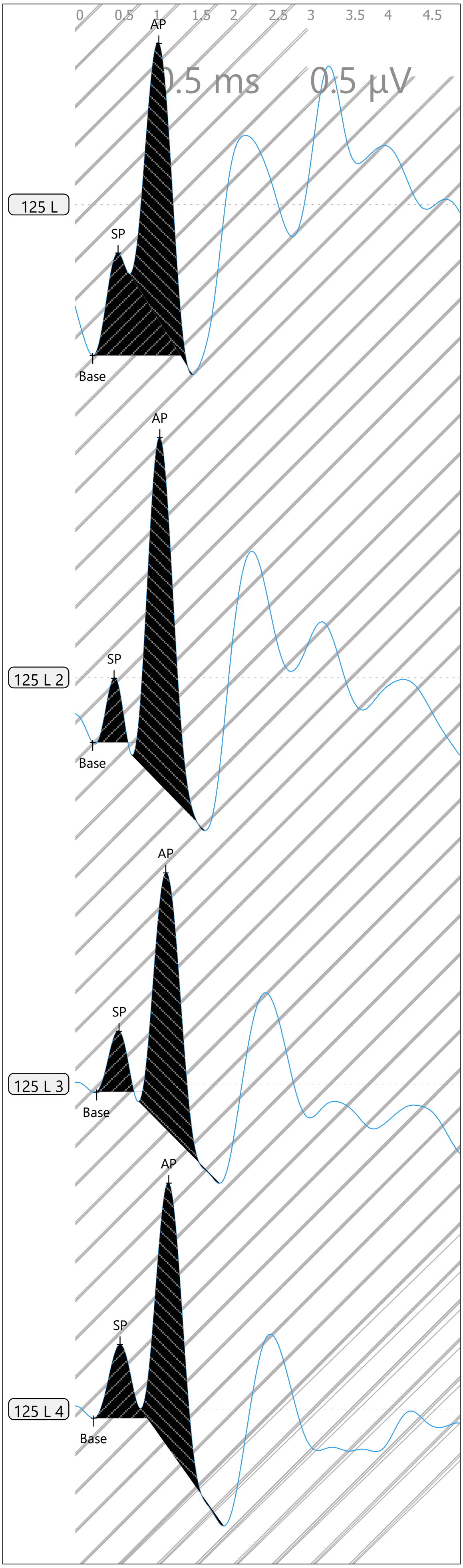
0
0.5
1
1.5
2
2.5
3
3.5
4
4.5
0.5 ms
0.5 µV
125 L
AP
SP
Base
125 L 2
AP
SP
Base
125 L 3
AP
SP
Base
125 L 4
AP
SP
Base

&&

N	Base (ms)	SP (ms)	AP (ms)	SP–Base (ms)	AP–Base (ms)	SP–Base (μV)	AP–Base (μV)	
125 L	0.22	0.56	1.08	0.33	0.86	1.33	4.05	0.33
125 L 2	0.22	0.50	1.10	0.28	0.87	0.84	3.96	0.21
125 L 3	0.28	0.57	1.18	0.29	0.90	0.79	2.85	0.28
125 L 4	0.24	0.58	1.22	0.34	0.98	0.95	3.05	0.31

Trace parameters

N	Electr.	HPF, Hz	LPF, Hz	50 Hz	Rejection ±μV	Aver.	Rejec
125 L	Cz-M1	5	2000		50	451	530
125 L 2	Cz-M1	5	2000		50	1372	616
125 L 3	Cz-M1	5	2000		50	1500	149
125 L 4	Cz-M1	5	2000		50	1141	71

ECochG: ECochG 2:
Fpz-M2

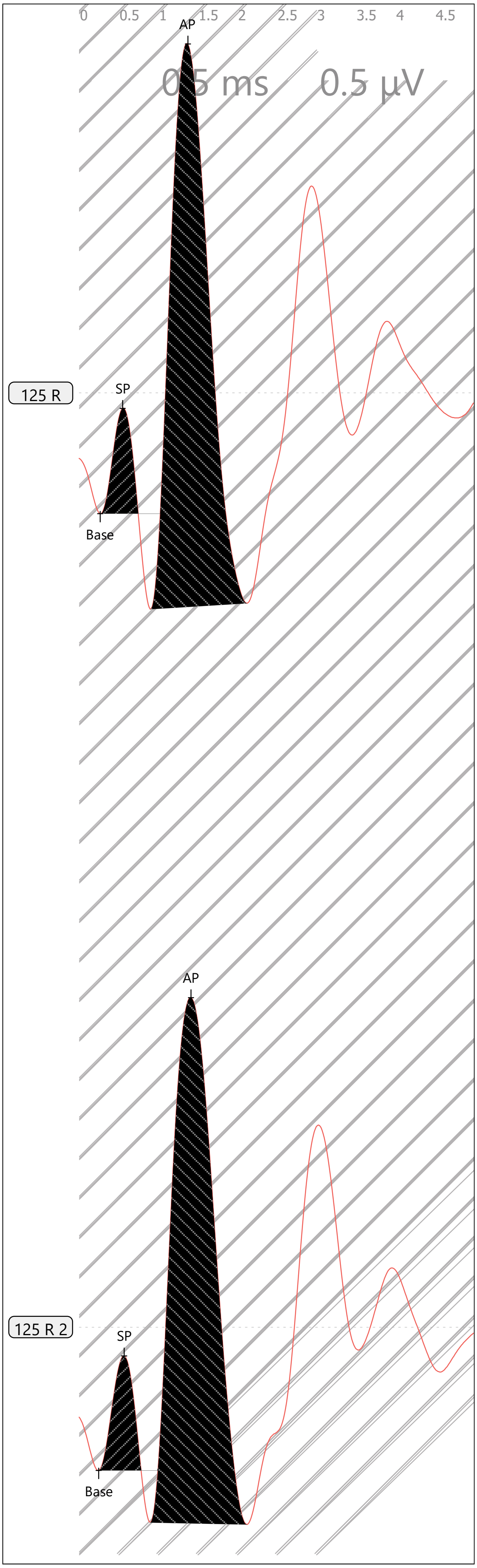
0
0.5
1
1.5
2
2.5
3
3.5
4
4.5
0.5 ms
0.5 µV
AP
125 R
SP
Base
AP
125 R 2
SP
Base

&&

N	ase (ms)	SP (ms)	AP (ms)	SP–Base (ms)	AP–Base (ms)	SP–Base (µV)	AP–Base (µV)	
125 R	0.26	0.56	1.38	0.29	1.11	1.33	5.94	0.22
125 R 2	0.25	0.57	1.42	0.32	1.16	1.45	5.98	0.24

Trace parameters

N	Electr.	HPF, Hz	LPF, Hz	50 Hz	Rejection ±µV	Aver.	Rejec
125 R	Fpz-M2	5	2000		50	1500	51
125 R 2	Fpz-M2	5	2000		50	1113	40

CONCLUSION:

Doctor:
