# Supplementary material for: Exposure to high-altitude hypobaric hypoxic environment induces low-frequency hearing loss in C57BL/6J mice: Mediated by slowing down the postsynaptic electrical signal transmission speed in the cochlear-inferior colliculus auditory signaling pathway
Source: PLoS One. 2026 Mar 11;21(3):e0342321. doi: 10.1371/journal.pone.0342321 (PMC12978441; doi:10.1371/journal.pone.0342321)
Supplement: S1 File — (ZIP) [file pone.0342321.s001.zip › 2025-6-20-30d-2.pdf]

Exam report

Patient: 2025-6-20-30d-2, - (-)
Date: June 20, 2025

ABR: ABR 2 CLICK
1: Cz-M1

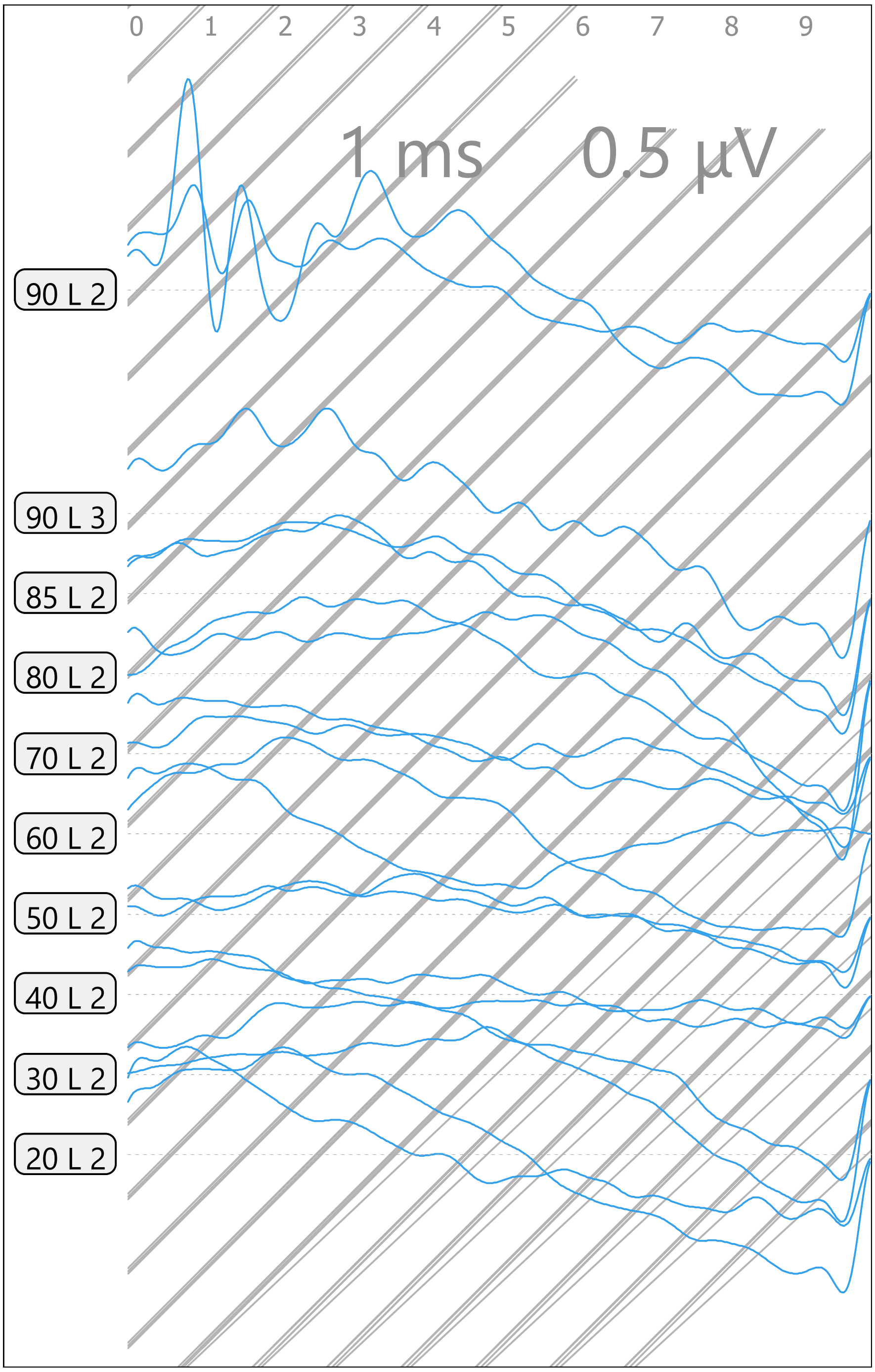

Trace parameters

N	Electr.	HPF, Hz	LPF, Hz	50 Hz	Rejection ±µV	Aver.	Reject.
90 L	Cz-M1	100	2000		10	1000	0
90 L 2	Cz-M1	100	2000		10	1000	0
90 L 3	Cz-M1	100	2000		10	1000	0
85 L	Cz-M1	100	2000		10	1000	0
85 L 2	Cz-M1	100	2000		10	1000	0
80 L	Cz-M1	100	2000		10	1000	0
80 L 2	Cz-M1	100	2000		10	1000	0

70 L	Cz-M1	100	2000		10	1000	0
70 L 2	Cz-M1	100	2000		10	1000	0
60 L	Cz-M1	100	2000		10	1000	0
60 L 2	Cz-M1	100	2000		10	1000	0
50 L	Cz-M1	100	2000		10	1000	0
50 L 2	Cz-M1	100	2000		10	1000	0
40 L	Cz-M1	100	2000		10	1000	0
40 L 2	Cz-M1	100	2000		10	1000	0
30 L	Cz-M1	100	2000		10	1000	0
30 L 2	Cz-M1	100	2000		10	1000	0
20 L	Cz-M1	100	2000		10	1000	0
20 L 2	Cz-M1	100	2000		10	1000	0

ABR: ABR 2 4000Hz 1: Cz-M1

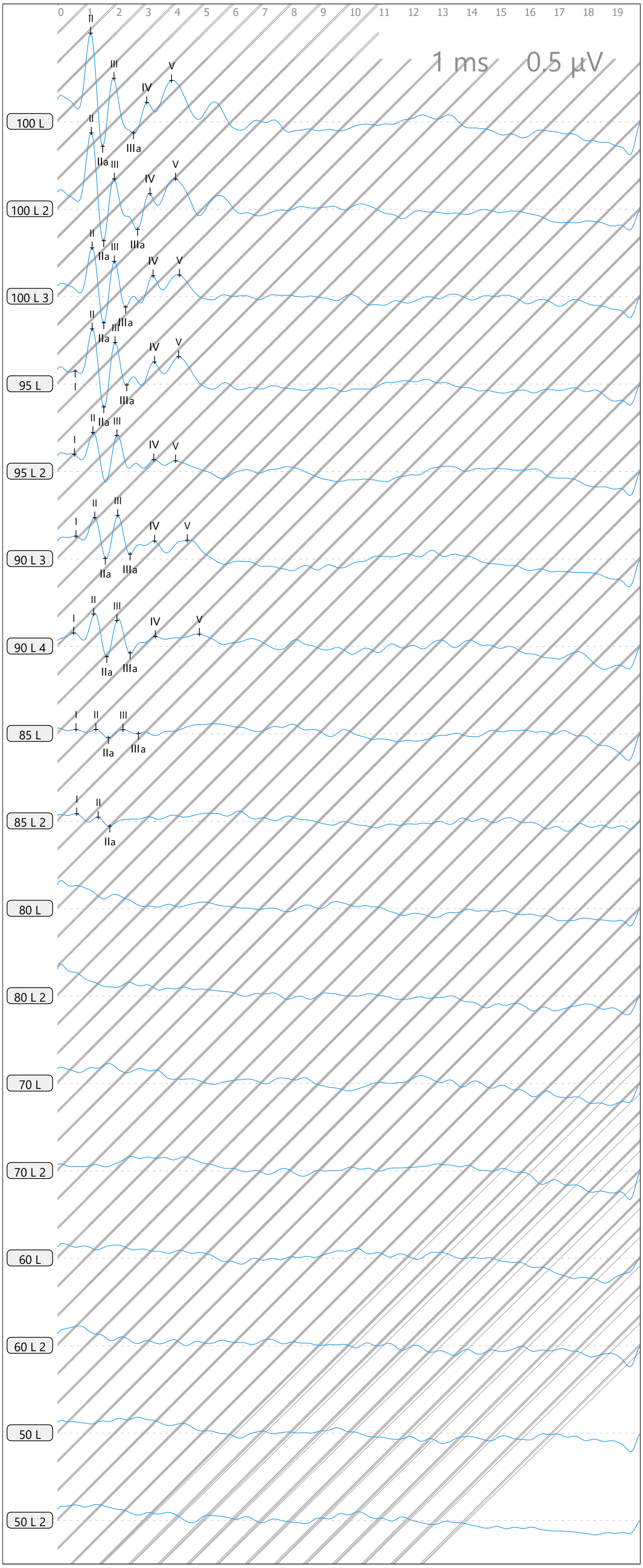
0 1 2 3 4 5 6 7 8 9 10 11 12 13 14 15 16 17 18 19
1 ms
0.5 µV
100 L
100 L 2
100 L 3
95 L
95 L 2
90 L 3
90 L 4
85 L
85 L 2
80 L
80 L 2
70 L
70 L 2
60 L
60 L 2
50 L
50 L 2

&&

N	I (ms)	II (ms)	III (ms)	IV (ms)	V (ms
100 L		1.14	1.93	3.07	3.92
100 L 2		1.16	1.96	3.18	4.05
100 L 3		1.19	1.96	3.28	4.18
95 L	0.61	1.19	1.98	3.33	4.15
95 L 2	0.58	1.22	2.04	3.31	4.05
90 L 3	0.64	1.27	2.06	3.33	4.45
90 L 4	0.56	1.24	2.04	3.36	4.87
85 L	0.64	1.32	2.25		
85 L 2	0.66	1.40			

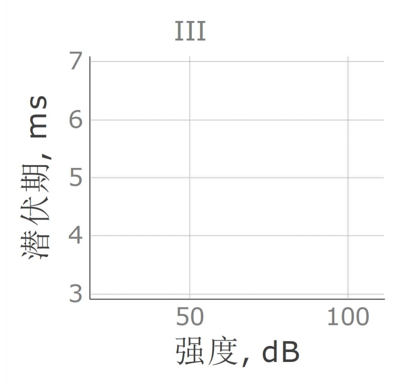

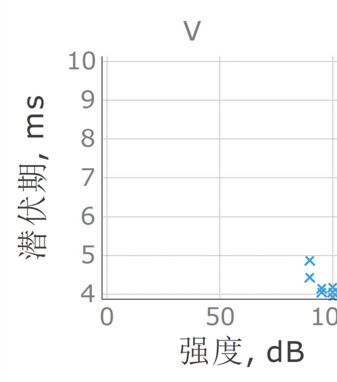

Trace parameters

N	Electr.	HPF, Hz	LPF, Hz	50 Hz	Rejection ±μV	Aver.	Rejec
100 L	Cz-M1	200	2000		10	1000	0
100 L 2	Cz-M1	200	2000		10	1000	0
100 L 3	Cz-M1	200	2000		10	1000	0
95 L	Cz-M1	200	2000		10	1003	0
95 L 2	Cz-M1	200	2000		10	1000	0
90 L 3	Cz-M1	200	2000		10	1000	0
90 L 4	Cz-M1	200	2000		10	1000	0
85 L	Cz-M1	200	2000		10	1000	0
85 L 2	Cz-M1	200	2000		10	1000	0
80 L	Cz-M1	200	2000		10	1000	0
80 L 2	Cz-M1	200	2000		10	1000	0
70 L	Cz-M1	200	2000		10	1000	0
70 L 2	Cz-M1	200	2000		10	1000	0
60 L	Cz-M1	200	2000		10	1000	0
60 L 2	Cz-M1	200	2000		10	1000	0
50 L	Cz-M1	200	2000		10	1000	0
50 L 2	Cz-M1	200	2000		10	1000	0

ABR: ABR 2 8000Hz 1: Cz-M1

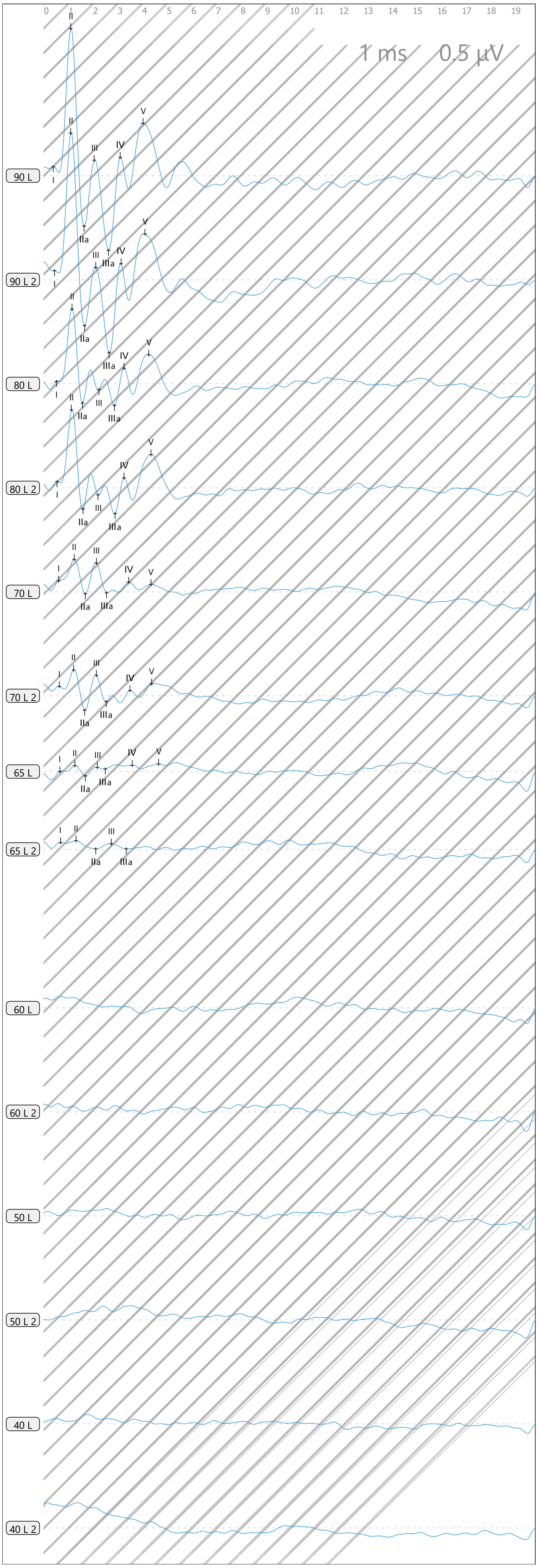

0 1 2 3 4 5 6 7 8 9 10 11 12 13 14 15 16 17 18 19
1 ms
0.5 µV
90 L
90 L 2
80 L
80 L 2
70 L
70 L 2
65 L
65 L 2
60 L
60 L 2
50 L
50 L 2
40 L
40 L 2

latency&& amplitude

N	I (ms)	II (ms)	III (ms)	Ⅳ (ms)	V (ms)
90 L	0.40	1.11	2.06	3.12	4.05
90 L 2	0.45	1.11	2.12	3.15	4.13
80 L	0.53	1.16	2.25	3.28	4.29
80 L 2	0.56	1.14	2.22	3.28	4.37
70 L	0.61	1.24	2.14	3.47	4.37
70 L 2	0.64	1.22	2.14	3.52	4.39
65 L	0.66	1.27	2.20	3.60	4.68
65 L 2	0.69	1.32	2.75		

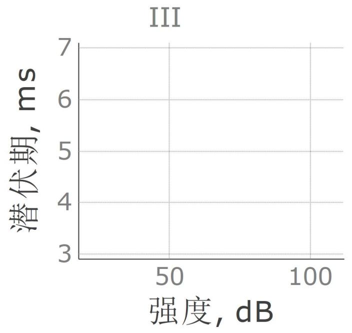

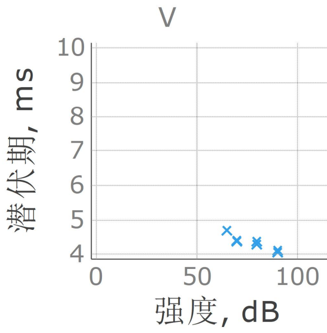

Trace parameters

N	Electr.	HPF, Hz	LPF, Hz	50 Hz	Rejection ±μV	Aver.	Reject.
90 L	Cz-M1	200	2000		10	1000	0
90 L 2	Cz-M1	200	2000		10	1000	0
80 L	Cz-M1	200	2000		10	1000	0
80 L 2	Cz-M1	200	2000		10	1000	0
70 L	Cz-M1	200	2000		10	1000	0
70 L 2	Cz-M1	200	2000		10	1000	0
65 L	Cz-M1	200	2000		10	1000	0
65 L 2	Cz-M1	200	2000		10	1000	0
60 L	Cz-M1	200	2000		10	1000	0
60 L 2	Cz-M1	200	2000		10	1000	0
50 L	Cz-M1	200	2000		10	1000	0
50 L 2	Cz-M1	200	2000		10	1000	0
40 L	Cz-M1	200	2000		10	1000	0
40 L 2	Cz-M1	200	2000		10	1000	0

ABR: ABR 2 CLICK 2: Cz-M2

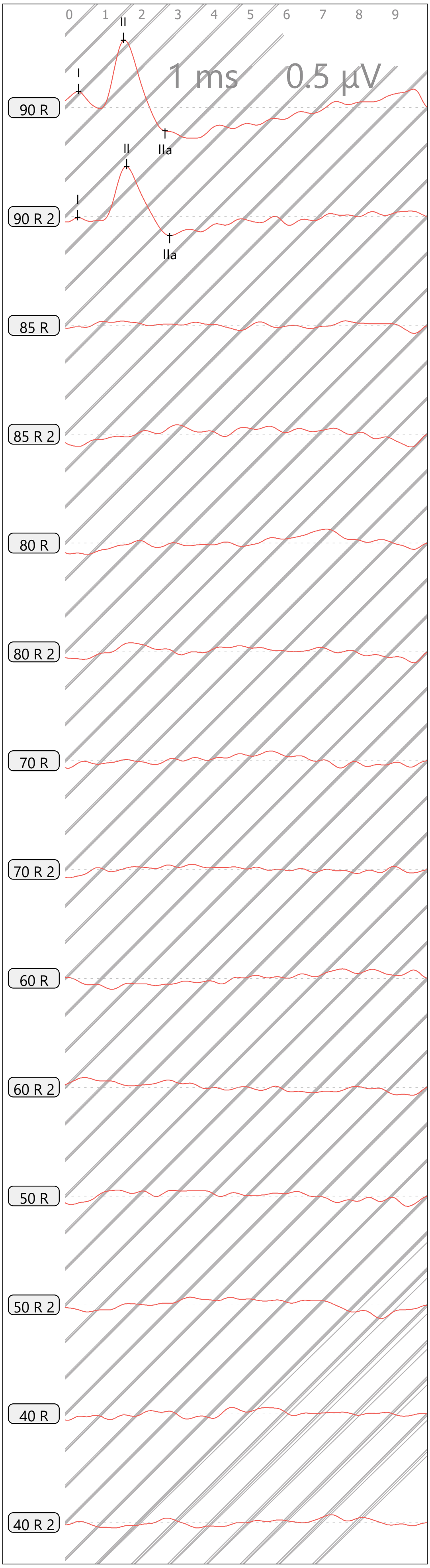

0 1 2 3 4 5 6 7 8 9
1 ms
0.5 µV
I
II
IIa
90 R
90 R 2
85 R
85 R 2
80 R
80 R 2
70 R
70 R 2
60 R
60 R 2
50 R
50 R 2
40 R
40 R 2

IV (ms)	V (ms)	I–III (ms)	I–V (ms)	III–V (ms)	

Trace parameters

N	Electr.	HPF, Hz	LPF, Hz	50 Hz	Rejection ±μV	Aver.	Reject
90 R	Cz-M2	100	2000		10	1000	0
90 R 2	Cz-M2	100	2000		10	1000	0
85 R	Cz-M2	100	2000		10	1000	0
85 R 2	Cz-M2	100	2000		10	1000	0
80 R	Cz-M2	100	2000		10	1000	0
80 R 2	Cz-M2	100	2000		10	1000	0
70 R	Cz-M2	100	2000		10	1000	0
70 R 2	Cz-M2	100	2000		10	1000	0
60 R	Cz-M2	100	2000		10	1000	0
60 R 2	Cz-M2	100	2000		10	1000	0
50 R	Cz-M2	100	2000		10	1000	0
50 R 2	Cz-M2	100	2000		10	1000	0
40 R	Cz-M2	100	2000		10	1000	0
40 R 2	Cz-M2	100	2000		10	1000	0

ABR: ABR 2 4000Hz 2: Cz-M2

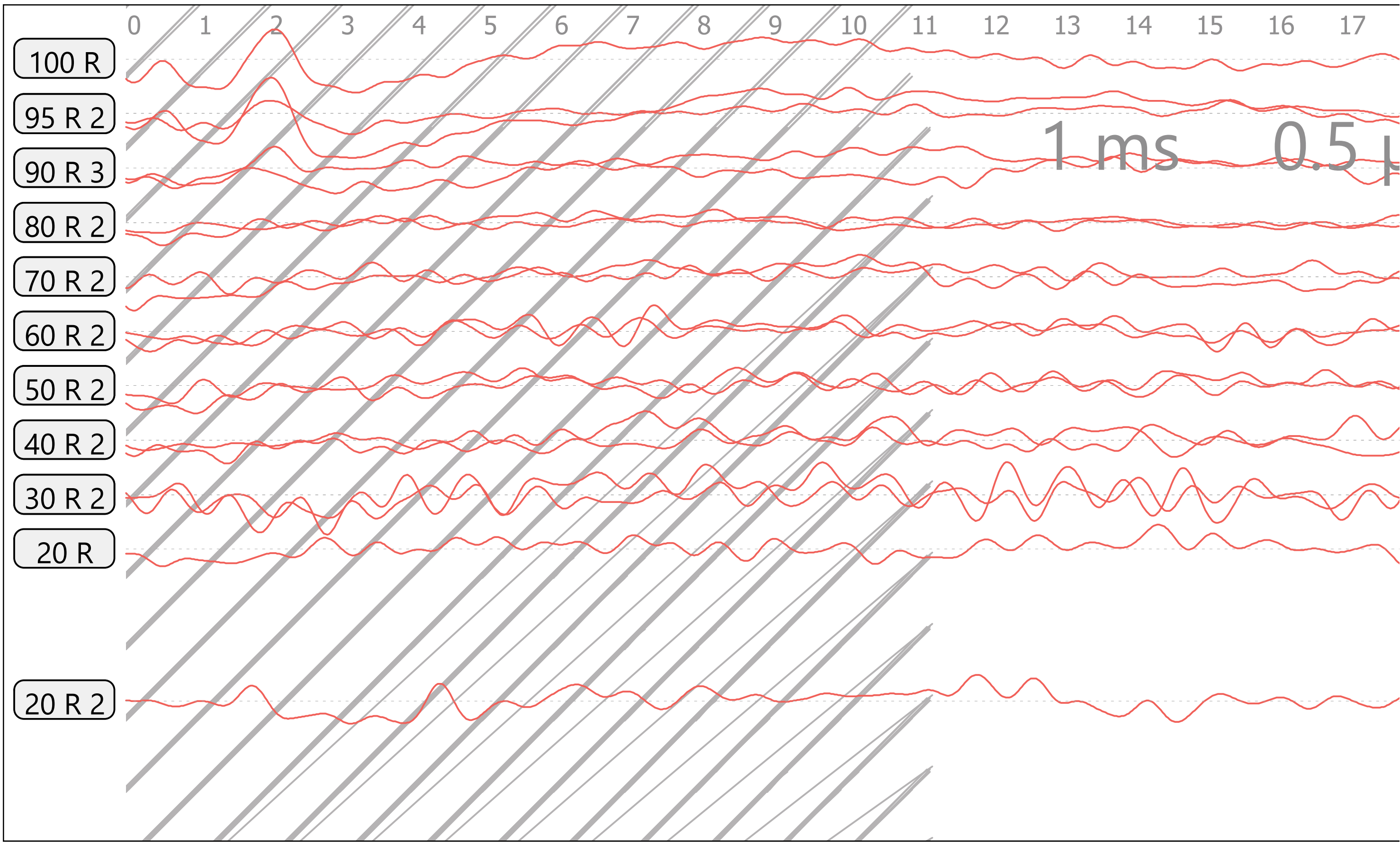

Trace parameters

N	Electr.	HPF, Hz	LPF, Hz	50 Hz	Rejection ±μV	Aver.	Reject
100 R	Cz-M2	200	2000		10	1000	0
95 R	Cz-M2	200	2000		10	1000	0
95 R 2	Cz-M2	200	2000		10	1000	0
90 R	Cz-M2	200	2000		10	1000	0
90 R 3	Cz-M2	200	2000		10	1000	0
80 R	Cz-M2	200	2000		10	1000	0
80 R 2	Cz-M2	200	2000		10	1000	0
70 R	Cz-M2	200	2000		10	1000	0
70 R 2	Cz-M2	200	2000		10	967	0
60 R	Cz-M2	200	2000		10	1000	0
60 R 2	Cz-M2	200	2000		10	658	0
50 R	Cz-M2	200	2000		10	1000	0
50 R 2	Cz-M2	200	2000		10	1000	0
40 R	Cz-M2	200	2000		10	631	0
40 R 2	Cz-M2	200	2000		10	1000	0
30 R	Cz-M2	200	2000		10	259	0
30 R 2	Cz-M2	200	2000		10	283	0

20 R	Cz-M2	200	2000		10	801	0
20 R 2	Cz-M2	200	2000		10	557	0

ABR: ABR 2 8000Hz 2: Cz-M2

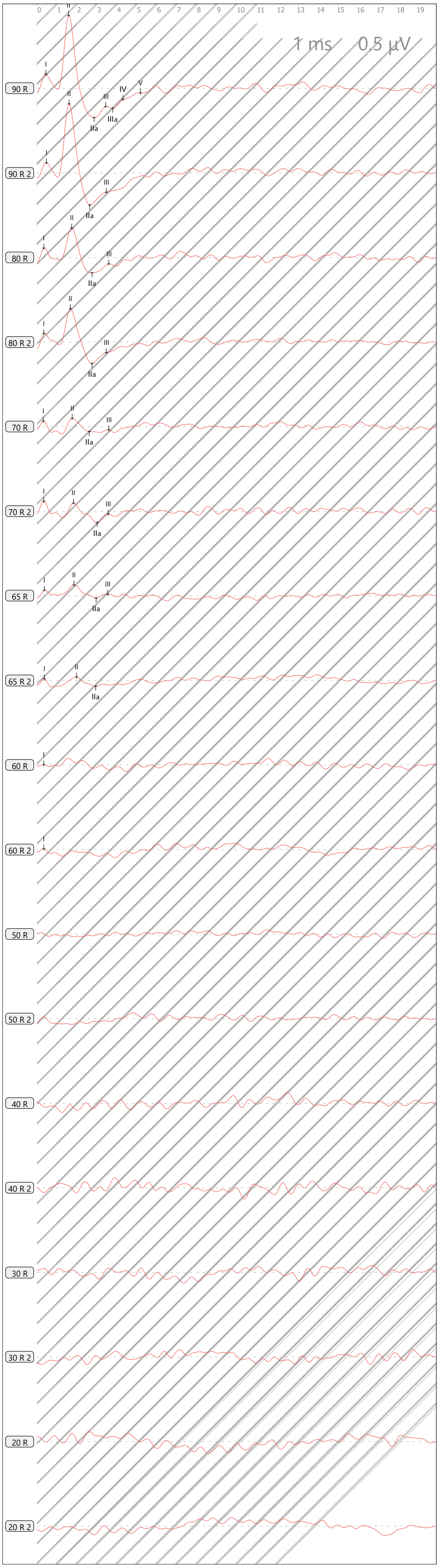
0 1 2 3 4 5 6 7 8 9 10 11 12 13 14 15 16 17 18 19
1 ms
0.5 µV
90 R
90 R 2
80 R
80 R 2
70 R
70 R 2
65 R
65 R 2
60 R
60 R 2
50 R
50 R 2
40 R
40 R 2
30 R
30 R 2
20 R
20 R 2
I
II
IIa
III
IIIa
IV
V

IV (ms)	V (ms)	I–III (ms)	I–V (ms)	III–V (ms)
4.31	5.19	2.99	4.74	1.75
		2.99		
		3.25		
		3.12		
		3.28		
		3.23		
		3.18		

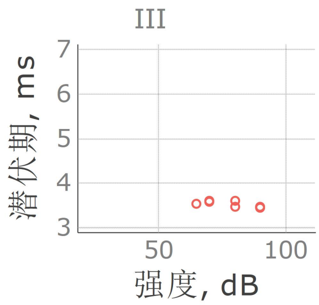

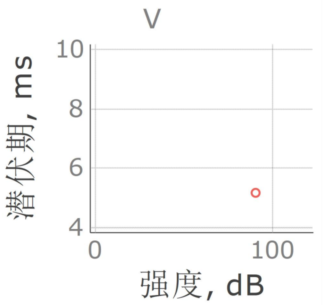

Trace parameters

N	Electr.	HPF, Hz	LPF, Hz	50 Hz	Rejection ±μV	Aver.	Reject
90 R	Cz-M2	200	2000		10	1000	0
90 R 2	Cz-M2	200	2000		10	1000	0
80 R	Cz-M2	200	2000		10	1000	0
80 R 2	Cz-M2	200	2000		10	1000	0
70 R	Cz-M2	200	2000		10	1000	0
70 R 2	Cz-M2	200	2000		10	1000	0
65 R	Cz-M2	200	2000		10	1000	0
65 R 2	Cz-M2	200	2000		10	1000	0
60 R	Cz-M2	200	2000		10	1000	0
60 R 2	Cz-M2	200	2000		10	1000	0
50 R	Cz-M2	200	2000		10	1000	0
50 R 2	Cz-M2	200	2000		10	1000	0
40 R	Cz-M2	200	2000		10	343	0
40 R 2	Cz-M2	200	2000		10	310	0
30 R	Cz-M2	200	2000		10	271	0
30 R 2	Cz-M2	200	2000		10	244	0
20 R	Cz-M2	200	2000		10	392	0
20 R 2	Cz-M2	200	2000		10	413	0

DPOAE: 1-12 kHz 70/70 dB 3 points

Test result (right ear):

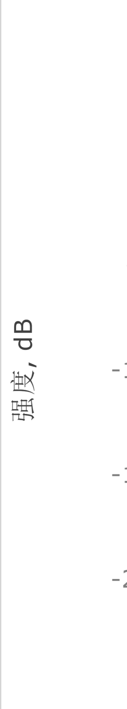

DPOAE

F2, Hz	L1, dB	L2, dB	DP, dB	噪声, dB	SNR, dB	OAE
988	67.9	68.4	-12.06	-4.44	-7.6	✘
1270	68.8	69.0	-18.70	-10.70	-8.0	✘
1778	69.6	69.7	-13.23	-15.00	1.8	✘
2222	70.0	70.0	-10.64	-14.16	3.5	✘
2500	70.2	70.1	-12.64	-15.00	2.4	✘
3200	70.4	70.1	-19.64	-15.00	-4.6	✘
4444	70.8	70.5	-11.03	-13.10	2.1	✘
5000	70.8	70.4	-3.21	-9.67	6.5	✓
6154	70.6	70.6	-22.06	-15.00	-7.1	✘
8000	70.8	70.2	-8.73	-15.00	6.3	✓
8889	71.1	67.9	-7.00	-13.09	6.1	✓
10000	70.7	56.4	1.97	-6.30	8.3	✓
11429	59.1	60.8	-1.69	-7.69	6.0	✓
(dB SPL)	:: 0.0					

ECochG: ECochG 1: Cz-M1

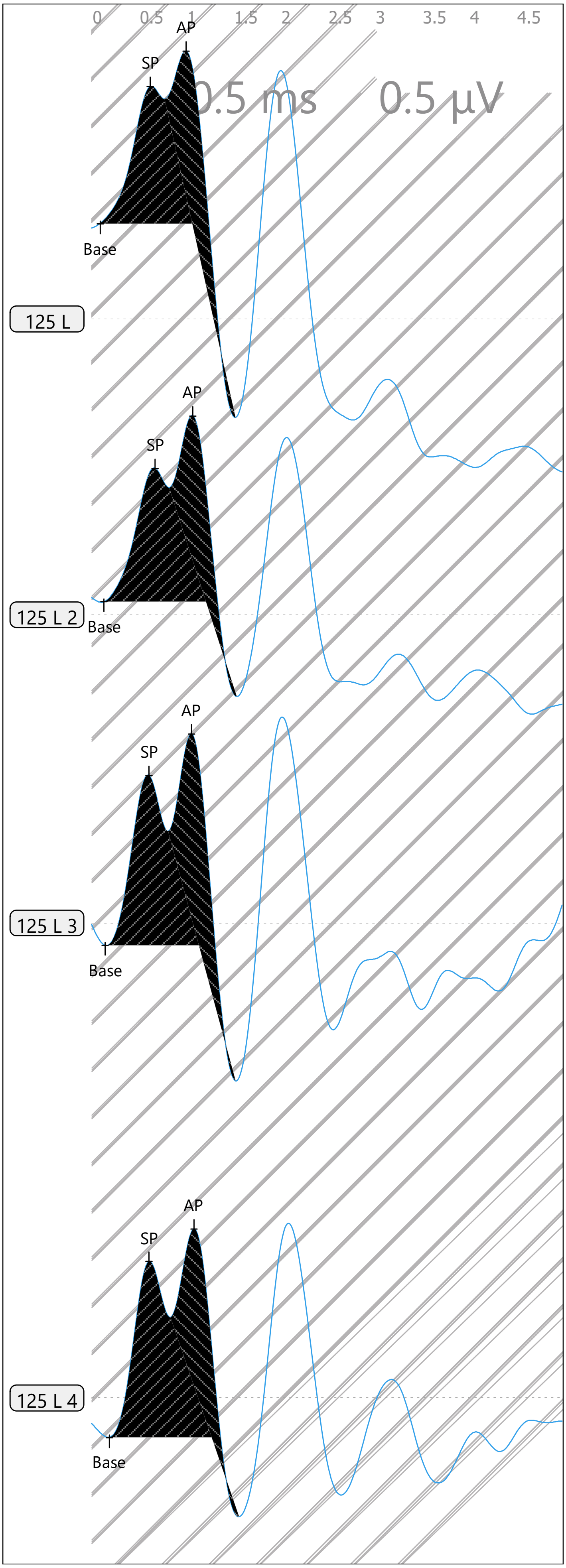

0
0.5
1
1.5
2
2.5
3
3.5
4
4.5
0.5 ms
0.5 µV
AP
SP
Base
125 L
AP
SP
125 L 2
Base
AP
SP
125 L 3
Base
AP
SP
125 L 4
Base

&&

N	Base (ms)	SP (ms)	AP (ms)	SP–Base (ms)	AP–Base (ms)	SP–Base (μV)	AP–Base (μV)	
125 L	0.09	0.62	1.01	0.53	0.91	1.45	1.83	0.79
125 L 2	0.13	0.67	1.07	0.54	0.94	1.41	1.97	0.72
125 L 3	0.15	0.61	1.06	0.46	0.91	1.80	2.24	0.80
125 L 4	0.19	0.61	1.08	0.42	0.90	1.86	2.21	0.84

Trace parameters

N	Electr.	HPF, Hz	LPF, Hz	50 Hz	Rejection ±μV	Aver.	Rejec
125 L	Cz-M1	5	2000		50	1330	221
125 L 2	Cz-M1	5	2000		50	1211	165
125 L 3	Cz-M1	5	2000		50	409	76
125 L 4	Cz-M1	5	2000		50	1500	298

ECochG: ECochG 2:
Fpz-M2

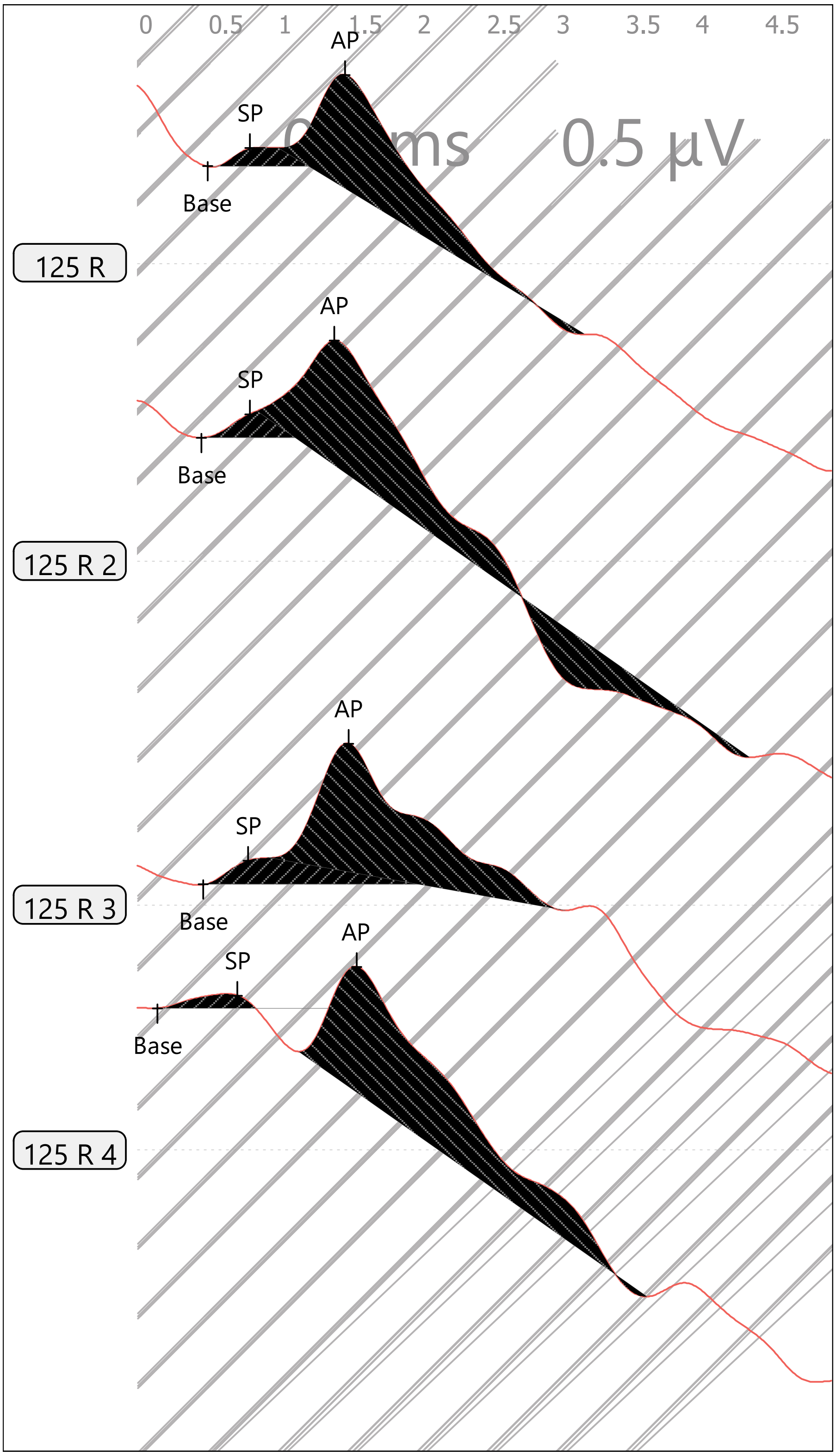

&&

N	ase (ms)	SP (ms)	AP (ms)	SP–Base (ms)	AP–Base (ms)	SP–Base (µV)	AP–Base (µV)	
125 R	0.50	0.81	1.49	0.30	0.99	0.13	0.66	0.20
125 R 2	0.46	0.81	1.42	0.34	0.95	0.17	0.70	0.25
125 R 3	0.48	0.79	1.52	0.32	1.05	0.17	1.01	0.17
125 R 4	0.15	0.71	1.57	0.57	1.43	0.09	0.30	0.31

Trace parameters

N	Electr.	HPF, Hz	LPF, Hz	50 Hz	Rejection ±µV	Aver.	Rejec
125 R	Fpz-M2	5	2000		50	1460	124
125 R 2	Fpz-M2	5	2000		50	1500	116

125 R 3	Fpz-M2	5	2000		50	1048	56
125 R 4	Fpz-M2	5	2000		50	1148	53

CONCLUSION:

Doctor:
