# Supplementary material for: Exposure to high-altitude hypobaric hypoxic environment induces low-frequency hearing loss in C57BL/6J mice: Mediated by slowing down the postsynaptic electrical signal transmission speed in the cochlear-inferior colliculus auditory signaling pathway
Source: PLoS One. 2026 Mar 11;21(3):e0342321. doi: 10.1371/journal.pone.0342321 (PMC12978441; doi:10.1371/journal.pone.0342321)
Supplement: S1 File — (ZIP) [file pone.0342321.s001.zip › 2025-6-20-30d-4.pdf]

Exam report

Patient: 2025-6-20-30d-4**,** - (-)
Date: June 20, 2025

ABR: ABR 2 CLICK
1: Cz-M1

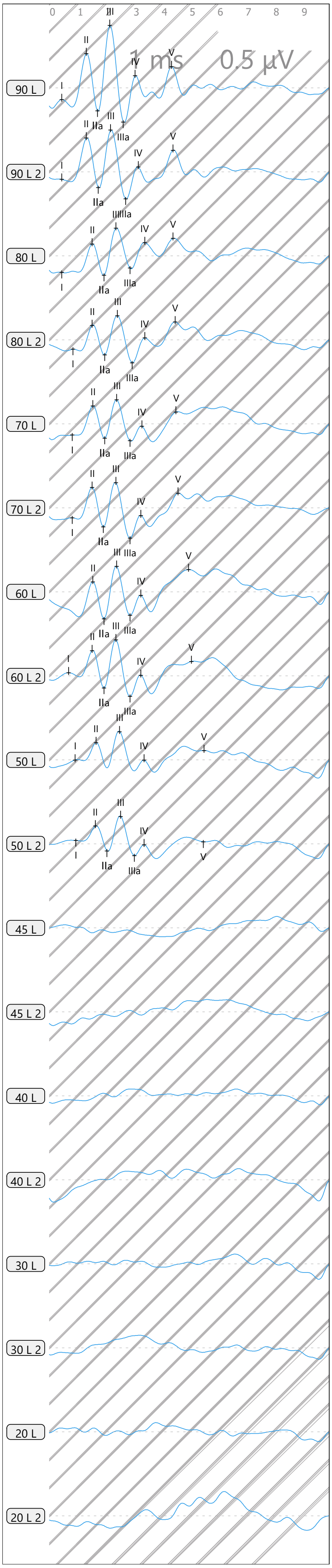

0 1 2 3 4 5 6 7 8 9
1 ms
0.5 µV
90 L
90 L 2
80 L
80 L 2
70 L
70 L 2
60 L
60 L 2
50 L
50 L 2
45 L
45 L 2
40 L
40 L 2
30 L
30 L 2
20 L
20 L 2

latency&&

N	I (ms)	II (ms)	III (ms)	IV (ms)	V (ms)
90 L	0.45	1.32	2.17	3.07	4.37
90 L 2	0.45	1.32	2.20	3.18	4.42
80 L	0.45	1.53	2.38	3.41	4.42
80 L 2	0.85	1.53	2.43	3.41	4.50
70 L	0.82	1.56	2.41	3.31	4.52
70 L 2	0.82	1.53	2.38	3.28	4.60
60 L		1.56	2.41	3.28	4.97
60 L 2	0.69	1.53	2.38	3.28	5.08
50 L	0.93	1.67	2.51	3.39	5.53
50 L 2	0.95	1.64	2.54	3.39	5.50

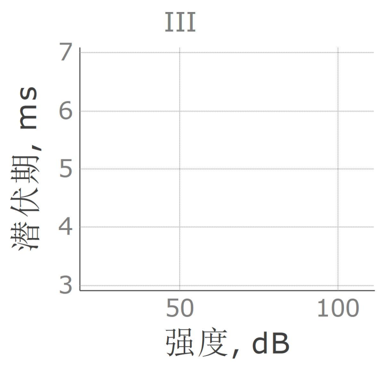

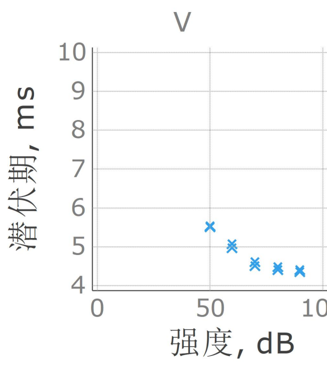

Trace parameters

N	Electr.	HPF, Hz	LPF, Hz	50 Hz	Rejection ±μV	Aver.	Reject.
90 L	Cz-M1	100	2000		10	1000	0
90 L 2	Cz-M1	100	2000		10	1000	0
80 L	Cz-M1	100	2000		10	1000	0
80 L 2	Cz-M1	100	2000		10	1000	0
70 L	Cz-M1	100	2000		10	1000	0
70 L 2	Cz-M1	100	2000		10	1000	0
60 L	Cz-M1	100	2000		10	1000	0
60 L 2	Cz-M1	100	2000		10	1000	0
50 L	Cz-M1	100	2000		10	1000	0
50 L 2	Cz-M1	100	2000		10	1000	0
45 L	Cz-M1	100	2000		10	1000	0
45 L 2	Cz-M1	100	2000		10	1000	0
40 L	Cz-M1	100	2000		10	1000	0
40 L 2	Cz-M1	100	2000		10	1000	0
30 L	Cz-M1	100	2000		10	1000	0
30 L 2	Cz-M1	100	2000		10	1000	0
20 L	Cz-M1	100	2000		10	913	0
20 L 2	Cz-M1	100	2000		10	265	0

ABR: ABR 2 4000Hz 1: Cz-M1

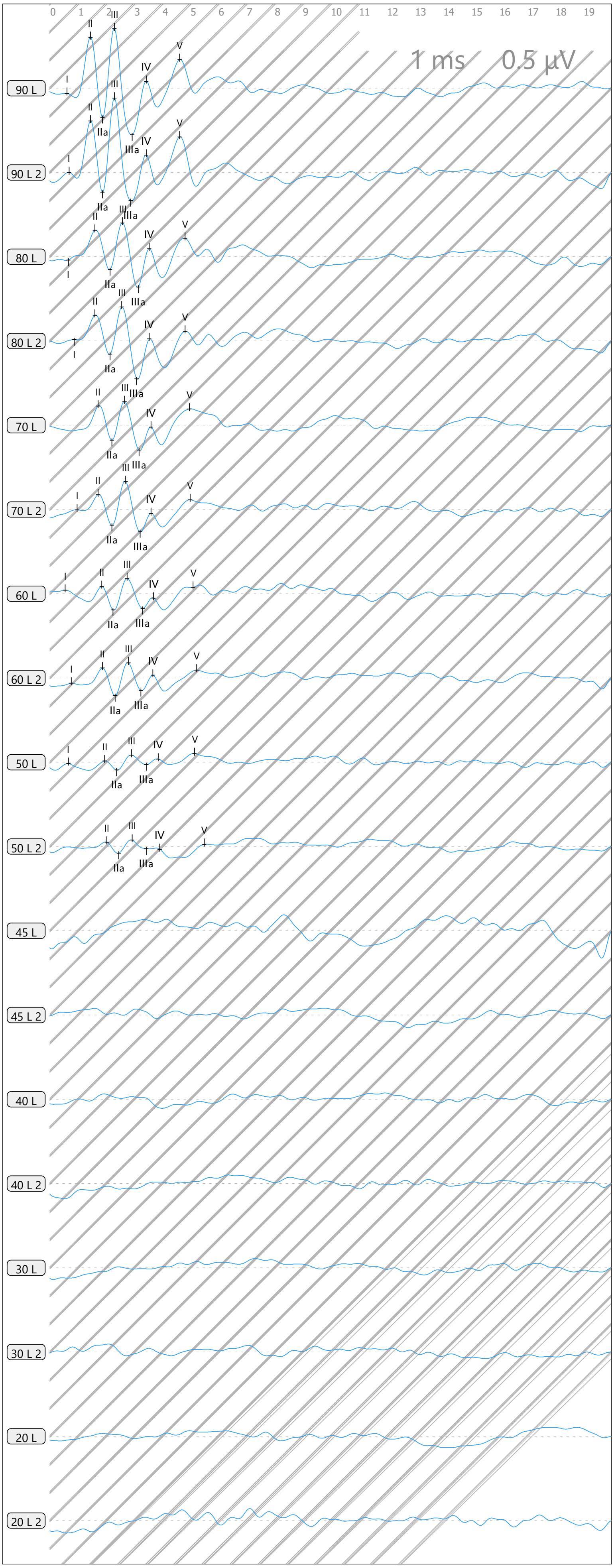
0 1 2 3 4 5 6 7 8 9 10 11 12 13 14 15 16 17 18 19
1 ms
0.5 µV
90 L
90 L 2
80 L
80 L 2
70 L
70 L 2
60 L
60 L 2
50 L
50 L 2
45 L
45 L 2
40 L
40 L 2
30 L
30 L 2
20 L
20 L 2
I
II
IIa
III
IIIa
IV
V

&&

N	I (ms)	II (ms)	III (ms)	IV (ms)	V (ms)
90 L	0.61	1.46	2.30	3.44	4.63
90 L 2	0.69	1.46	2.30	3.44	4.63
80 L	0.66	1.61	2.59	3.55	4.82
80 L 2	0.87	1.61	2.57	3.55	4.82
70 L		1.72	2.67	3.60	4.97
70 L 2	0.98	1.72	2.70	3.60	5.00
60 L	0.56	1.85	2.75	3.70	5.11
60 L 2	0.77	1.88	2.80	3.68	5.24
50 L	0.66	1.96	2.91	3.86	5.16
50 L 2		2.04	2.94	3.92	5.50

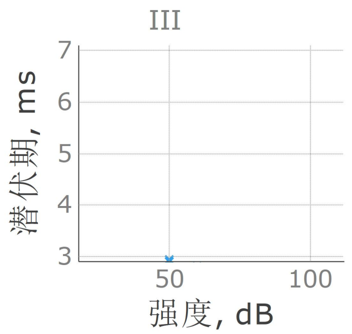

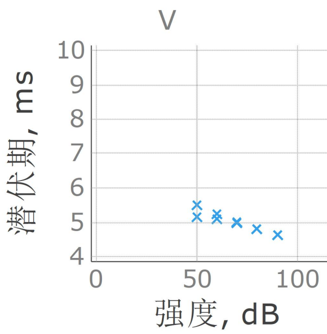

Trace parameters

N	Electr.	HPF, Hz	LPF, Hz	50 Hz	Rejection ±μV	Aver.	Reject.
90 L	Cz-M1	200	2000		10	1000	0
90 L 2	Cz-M1	200	2000		10	1000	0
80 L	Cz-M1	200	2000		10	1000	0
80 L 2	Cz-M1	200	2000		10	1000	0
70 L	Cz-M1	200	2000		10	1000	0
70 L 2	Cz-M1	200	2000		10	1000	0
60 L	Cz-M1	200	2000		10	1000	0
60 L 2	Cz-M1	200	2000		10	1000	0
50 L	Cz-M1	200	2000		10	1000	0
50 L 2	Cz-M1	200	2000		10	1000	0
45 L	Cz-M1	200	2000		10	223	0
45 L 2	Cz-M1	200	2000		10	1000	0
40 L	Cz-M1	200	2000		10	1000	0
40 L 2	Cz-M1	200	2000		10	1000	0
30 L	Cz-M1	200	2000		10	1000	0
30 L 2	Cz-M1	200	2000		10	744	0
20 L	Cz-M1	200	2000		10	1000	0
20 L 2	Cz-M1	200	2000		10	373	0

ABR: ABR 2 8000Hz 1: Cz-M1

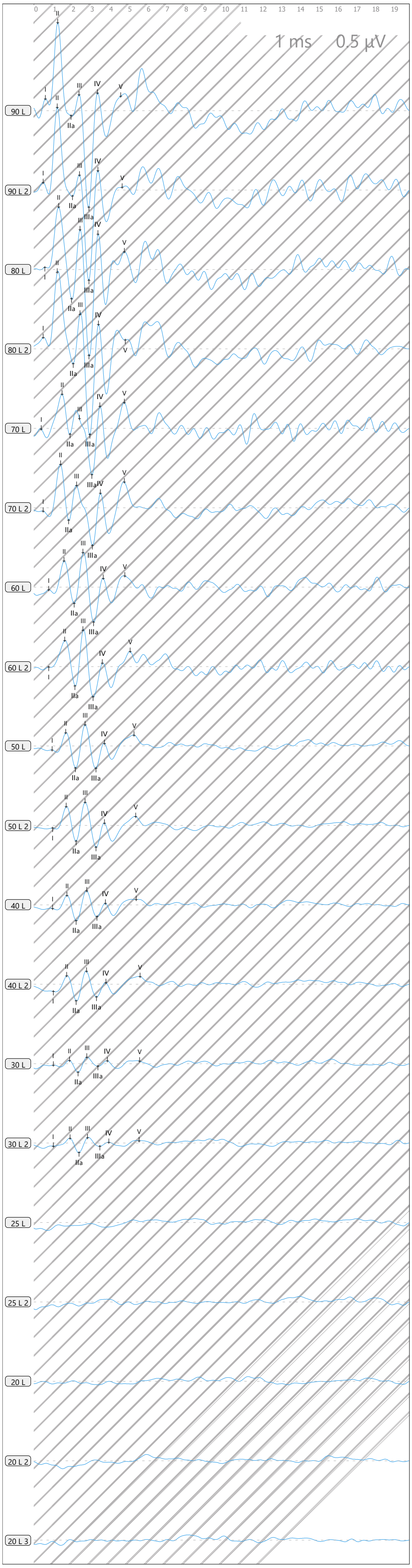
0 1 2 3 4 5 6 7 8 9 10 11 12 13 14 15 16 17 18 19
1 ms
0.5 µV
90 L
90 L 2
80 L
80 L 2
70 L
70 L 2
60 L
60 L 2
50 L
50 L 2
40 L
40 L 2
30 L
30 L 2
25 L
25 L 2
20 L
20 L 2
20 L 3
I
II
IIa
III
IIIa
IV
V

&&

N	I (ms)	II (ms)	III (ms)	IV (ms)	V (ms
90 L	0.61	1.27	2.41	3.39	4.63
90 L 2	0.50	1.24	2.43	3.41	4.71
80 L	0.58	1.32	2.46	3.41	4.82
80 L 2	0.50	1.24	2.46	3.44	4.87
70 L	0.40	1.51	2.43	3.52	4.82
70 L 2	0.50	1.43	2.28	3.55	4.82
60 L	0.79	1.61	2.62	3.70	4.84
60 L 2	0.79	1.64	2.62	3.65	5.13
50 L	0.98	1.69	2.73	3.76	5.34
50 L 2	1.01	1.72	2.73	3.76	5.42
40 L	1.01	1.77	2.83	3.81	5.45
40 L 2	1.03	1.75	2.80	3.84	5.66
30 L	1.03	1.91	2.83	3.92	5.64
30 L 2	1.03	1.93	2.86	4.00	5.61

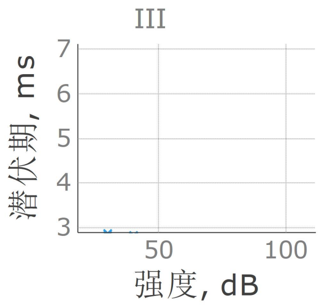

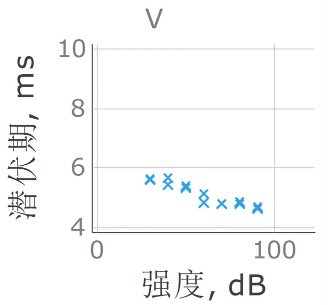

Trace parameters

N	Electr.	HPF, Hz	LPF, Hz	50 Hz	Rejection ±μV	Aver.	Reject.
90 L	Cz-M1	200	2000		10	1000	0
90 L 2	Cz-M1	200	2000		10	1000	0
80 L	Cz-M1	200	2000		10	1000	0
80 L 2	Cz-M1	200	2000		10	1000	0
70 L	Cz-M1	200	2000		10	1000	0
70 L 2	Cz-M1	200	2000		10	1000	0
60 L	Cz-M1	200	2000		10	1000	0
60 L 2	Cz-M1	200	2000		10	1000	0
50 L	Cz-M1	200	2000		10	1000	0
50 L 2	Cz-M1	200	2000		10	1000	0
40 L	Cz-M1	200	2000		10	1000	0
40 L 2	Cz-M1	200	2000		10	1000	0
30 L	Cz-M1	200	2000		10	1000	0
30 L 2	Cz-M1	200	2000		10	1000	0
25 L	Cz-M1	200	2000		10	1000	0
25 L 2	Cz-M1	200	2000		10	1000	0

20 L	Cz-M1	200	2000		10	1000	0
20 L 2	Cz-M1	200	2000		10	1000	0
20 L 3	Cz-M1	200	2000		10	1000	0

ABR: ABR CLICK 2: Cz-M2

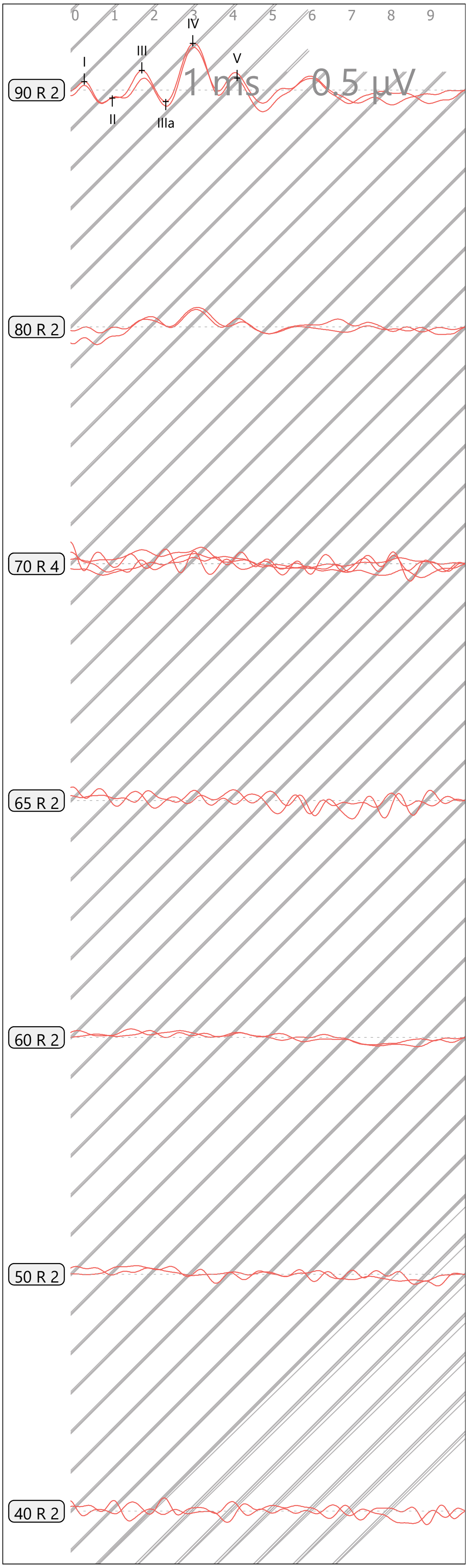
0 1 2 3 4 5 6 7 8 9
1 ms
0.5 µV
I
II
III
IIIa
IV
V
90 R 2
80 R 2
70 R 4
65 R 2
60 R 2
50 R 2
40 R 2

IV (ms)	V (ms)	I–V (ms)	I–III (ms)	III–V (ms)	V-(µ
3.10	4.21	3.86	1.46	2.41	

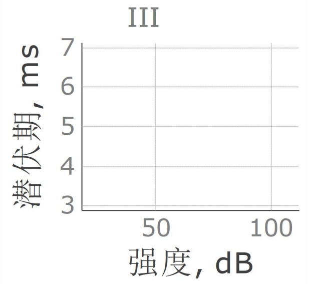

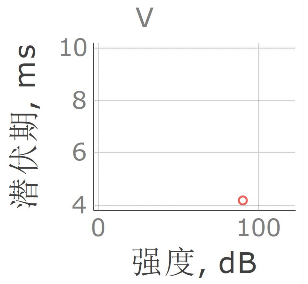

Trace parameters

N	Electr.	HPF, Hz	LPF, Hz	50 Hz	Rejection ±µV	Aver.	Reject
90 R	Cz-M2	100	2000		10	1000	0
90 R 2	Cz-M2	100	2000		10	1000	0
80 R	Cz-M2	100	2000		10	1000	0
80 R 2	Cz-M2	100	2000		10	1000	0
70 R	Cz-M2	100	2000		10	1000	0
70 R 2	Cz-M2	100	2000		10	1000	0
70 R 3	Cz-M2	100	2000		10	1000	0
70 R 4	Cz-M2	100	2000		10	1000	0
65 R	Cz-M2	100	2000		10	1000	0
65 R 2	Cz-M2	100	2000		10	1000	0
60 R	Cz-M2	100	2000		10	1000	0
60 R 2	Cz-M2	100	2000		10	1000	0
50 R	Cz-M2	100	2000		10	1000	0
50 R 2	Cz-M2	100	2000		10	1000	0
40 R	Cz-M2	100	2000		10	1000	0
40 R 2	Cz-M2	100	2000		10	1000	0

ABR: ABR 2 4000Hz 2: Cz-M2

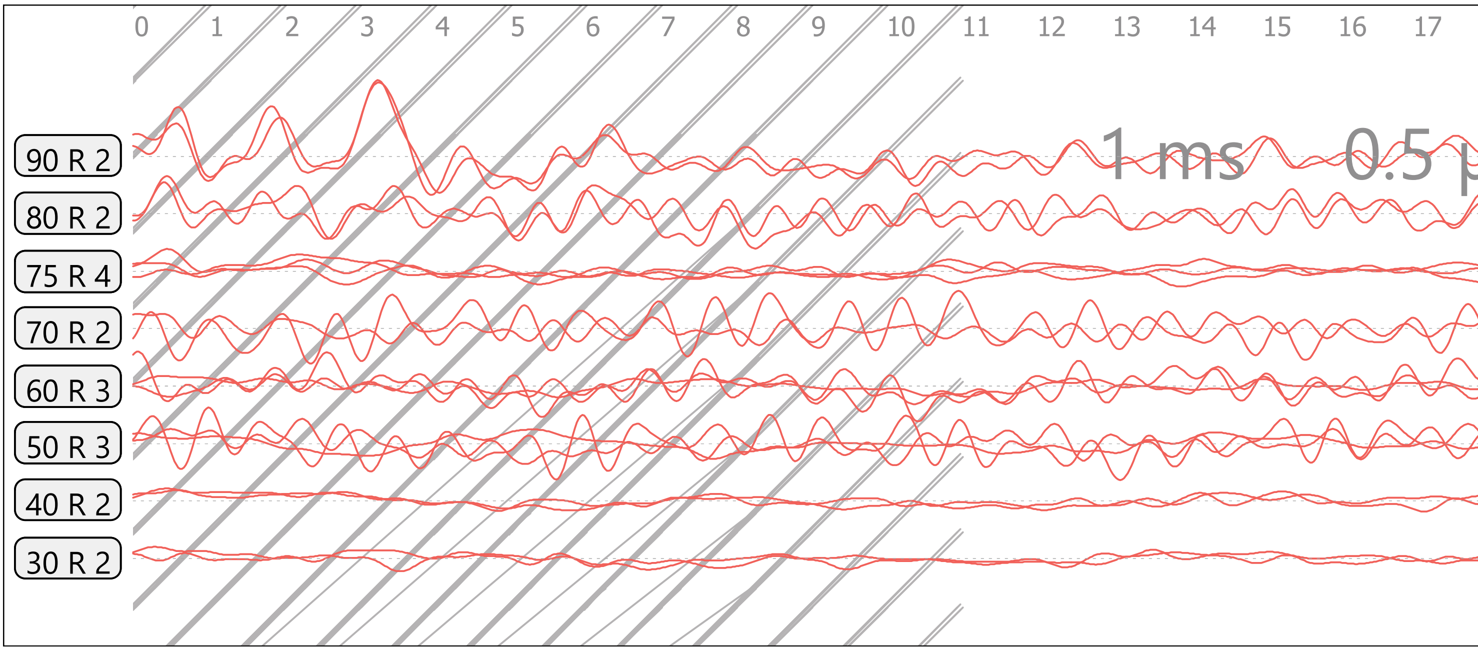

Trace parameters

N	Electr.	HPF,	LPF,	50 Hz	Rejection ±µV	Aver.	Reject

		Hz	Hz				
90 R	Cz-M2	200	2000		10	1000	0
90 R 2	Cz-M2	200	2000		10	1000	0
80 R	Cz-M2	200	2000		10	1000	0
80 R 2	Cz-M2	200	2000		10	1000	0
75 R	Cz-M2	200	2000		10	1000	0
75 R 3	Cz-M2	200	2000		10	1000	0
75 R 4	Cz-M2	200	2000		10	899	0
70 R	Cz-M2	200	2000		10	1000	0
70 R 2	Cz-M2	200	2000		10	1000	0
60 R	Cz-M2	200	2000		10	1000	0
60 R 2	Cz-M2	200	2000		10	1000	0
60 R 3	Cz-M2	200	2000		10	1000	0
50 R	Cz-M2	200	2000		10	1000	0
50 R 2	Cz-M2	200	2000		10	1000	0
50 R 3	Cz-M2	200	2000		10	1000	0
40 R	Cz-M2	200	2000		10	1000	0
40 R 2	Cz-M2	200	2000		10	1000	0
30 R	Cz-M2	200	2000		10	1000	0
30 R 2	Cz-M2	200	2000		10	1000	0

ABR: ABR 2 8000Hz 2: Cz-M2

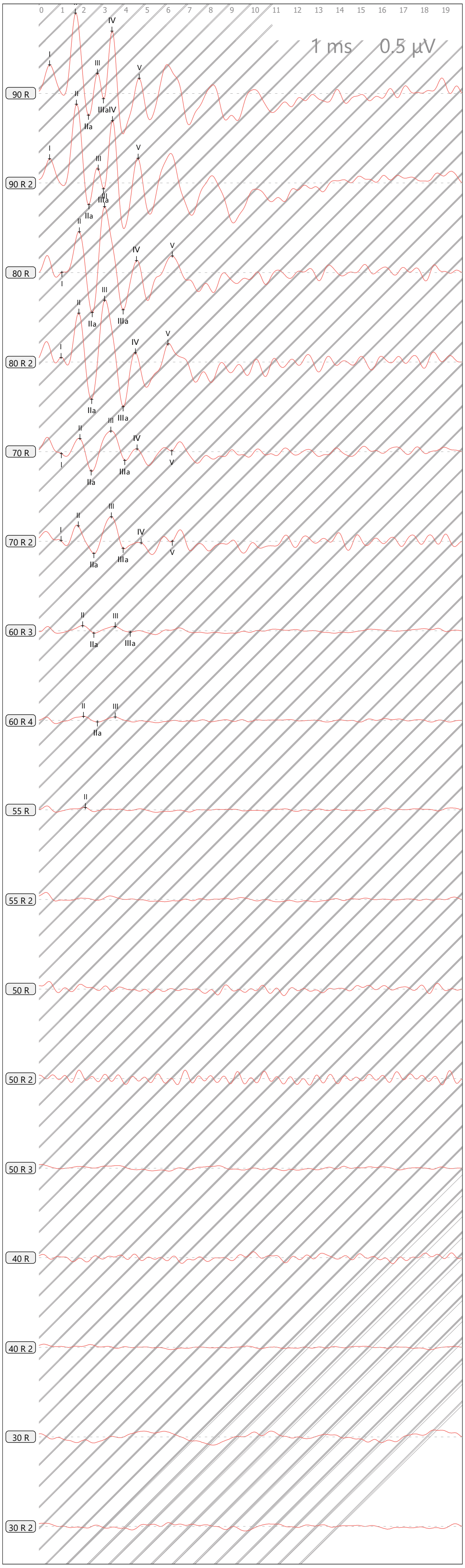

0 1 2 3 4 5 6 7 8 9 10 11 12 13 14 15 16 17 18 19
1 ms
0.5 µV
90 R
90 R 2
80 R
80 R 2
70 R
70 R 2
60 R 3
60 R 4
55 R
55 R 2
50 R
50 R 2
50 R 3
40 R
40 R 2
30 R
30 R 2

IV (ms)	V (ms)	I–III (ms)	I–V (ms)	III–V (ms)	
3.44	4.74	2.25	4.23	1.98	
3.47	4.68	2.30	4.18	1.88	
4.60	6.30	2.01	5.21	3.20	
4.55	6.09	2.06	5.05	2.99	
4.63	6.27	2.33	5.21	2.88	
4.82	6.30	2.38	5.27	2.88	

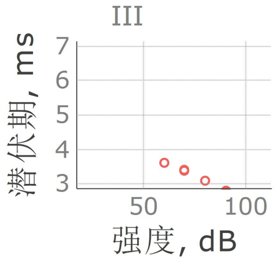

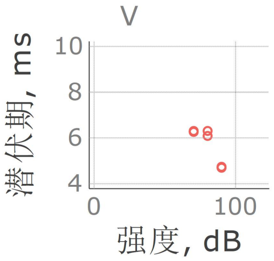

Trace parameters

N	Electr.	HPF, Hz	LPF, Hz	50 Hz	Rejection ±μV	Aver.	Reject
90 R	Cz-M2	200	2000		10	811	0
90 R 2	Cz-M2	200	2000		10	1000	0
80 R	Cz-M2	200	2000		10	1000	0
80 R 2	Cz-M2	200	2000		10	1000	0
70 R	Cz-M2	200	2000		10	1000	0
70 R 2	Cz-M2	200	2000		10	1000	0
60 R 3	Cz-M2	200	2000		10	1000	0
60 R 4	Cz-M2	200	2000		10	710	0
55 R	Cz-M2	200	2000		10	998	0
55 R 2	Cz-M2	200	2000		10	815	0
50 R	Cz-M2	200	2000		10	1000	0
50 R 2	Cz-M2	200	2000		10	1000	0
50 R 3	Cz-M2	200	2000		10	676	0
40 R	Cz-M2	200	2000		10	1000	0
40 R 2	Cz-M2	200	2000		10	1000	0
30 R	Cz-M2	200	2000		10	395	0
30 R 2	Cz-M2	200	2000		10	374	0

ECochG: ECochG 1:
Fpz-M1

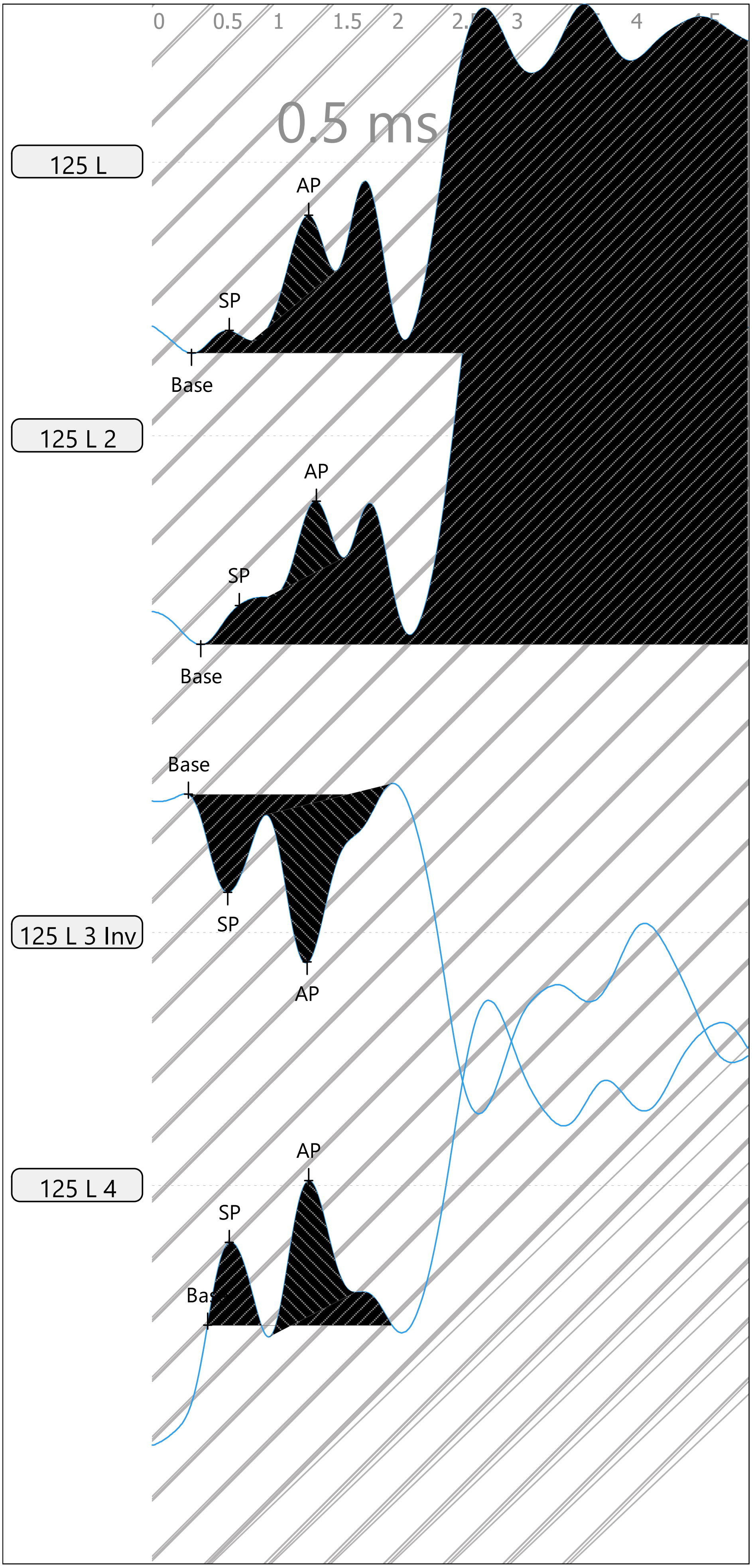

0
0.5
1
1.5
2
3
4
0.5 ms
125 L
AP
SP
Base
125 L 2
AP
SP
Base
Base
SP
AP
125 L 3 Inv
AP
SP
125 L 4

&&

N	Base (ms)	SP (ms)	AP (ms)	SP–Base (ms)	AP–Base (ms)	SP–Base (µV)	AP–Base (µV)	
125 L	0.33	0.65	1.31	0.32	0.98	0.19	1.15	0
125 L 2	0.41	0.73	1.38	0.32	0.97	0.33	1.20	0
125 L 3 Inv	0.30	0.64	1.30	0.33	0.99	0.82	1.41	0
125 L 4	0.46	0.65	1.31	0.19	0.85	0.69	1.21	0

Trace parameters

N	Electr.	HPF, Hz	LPF, Hz	50 Hz	Rejection ±µV	Aver.	R
125 L	Fpz-M1	5	2000		50	1500	
125 L 2	Fpz-M1	5	2000		50	1120	
125 L 3 Inv	Fpz-M1	5	2000		50	1430	
125 L 4	Fpz-M1	5	2000		50	881	

ECochG: ECochG 2:
Fpz-M2

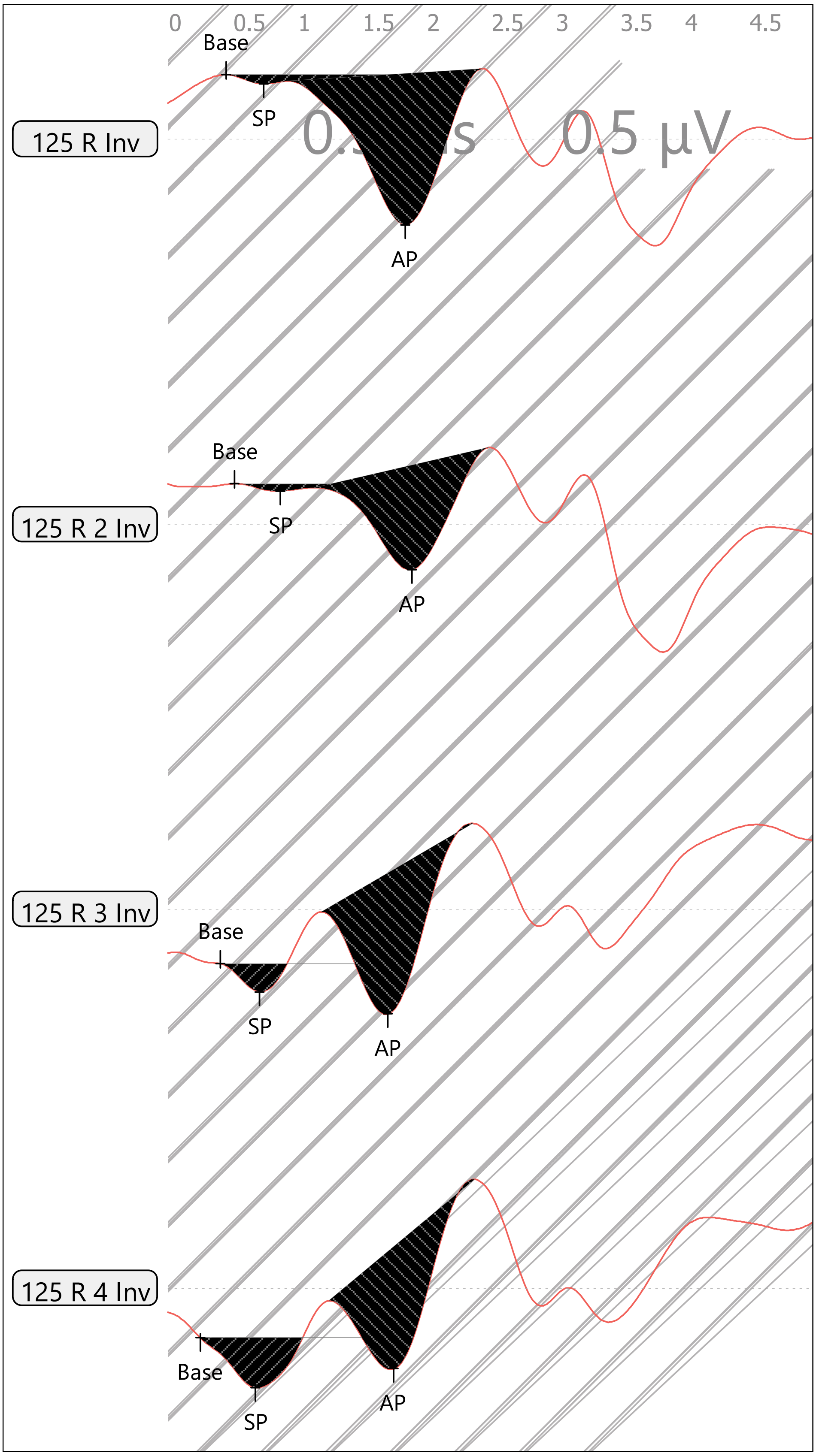

N	Base (ms)	SP (ms)	AP (ms)	SP–Base (ms)	AP–Base (ms)	SP–Base (µV)	AP–Base (µV)
125 R Inv	0.45	0.74	1.84	0.29	1.39	0.07	1.16
125 R 2 Inv	0.52	0.87	1.89	0.36	1.38	0.06	0.67
125 R 3 Inv	0.41	0.71	1.71	0.30	1.30	0.21	0.39
125 R 4 Inv	0.25	0.67	1.75	0.42	1.49	0.39	0.24

Trace parameters

N	Electr.	HPF, Hz	LPF, Hz	50 Hz	Rejection ±μV	Aver.	R[illegible]
125 R Inv	Fpz-M2	5	2000		50	1253	
125 R 2 Inv	Fpz-M2	5	2000		50	1088	
125 R 3 Inv	Fpz-M2	5	2000		50	1239	
125 R 4 Inv	Fpz-M2	5	2000		50	1175	

CONCLUSION:

Doctor:
