# Supplementary material for: Exposure to high-altitude hypobaric hypoxic environment induces low-frequency hearing loss in C57BL/6J mice: Mediated by slowing down the postsynaptic electrical signal transmission speed in the cochlear-inferior colliculus auditory signaling pathway
Source: PLoS One. 2026 Mar 11;21(3):e0342321. doi: 10.1371/journal.pone.0342321 (PMC12978441; doi:10.1371/journal.pone.0342321)
Supplement: S1 File — (ZIP) [file pone.0342321.s001.zip › 2025-6-9-3d-1.pdf]

Exam report

Patient: 2025-6-9-3d-1**,** - (-)
Date: June 10, 2025

ABR: ABR 2 CLICK 1: Cz-M1

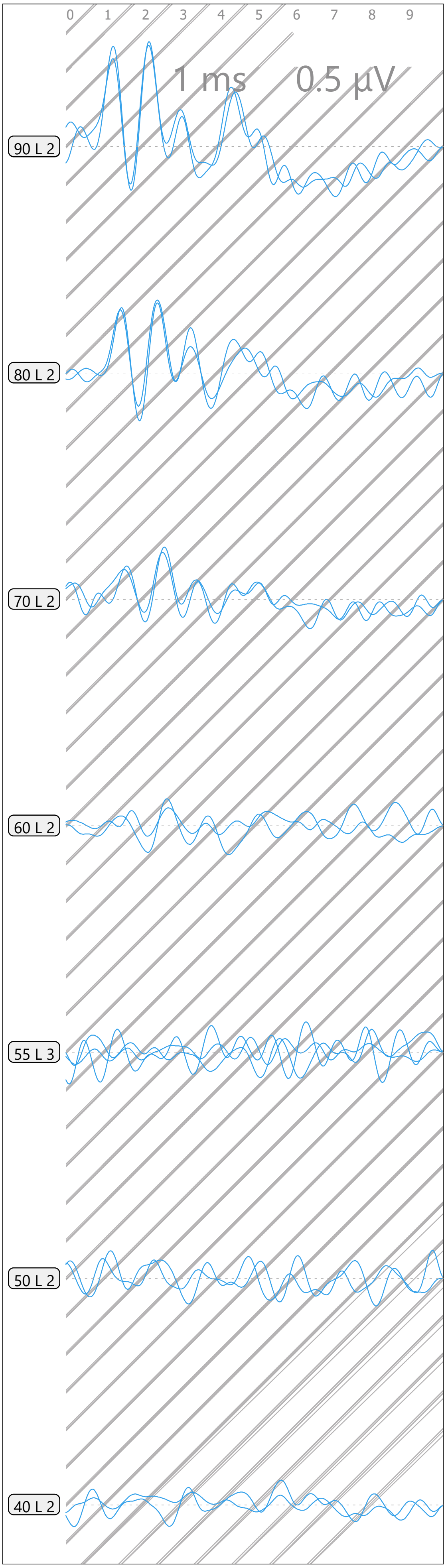
0 1 2 3 4 5 6 7 8 9
1 ms
0.5 µV
90 L 2
80 L 2
70 L 2
60 L 2
55 L 3
50 L 2
40 L 2

Trace parameters

N	Electr.	HPF, Hz	LPF, Hz	50 Hz	Rejection ±μV	Aver.	Reject.
90 L	Cz-M1	100	2000		10	1000	0
90 L 2	Cz-M1	100	2000		10	1000	0
80 L	Cz-M1	100	2000		10	1000	0
80 L 2	Cz-M1	100	2000		10	1000	0
70 L	Cz-M1	100	2000		10	1000	0
70 L 2	Cz-M1	100	2000		10	1000	0
60 L	Cz-M1	100	2000		10	1000	0
60 L 2	Cz-M1	100	2000		10	1000	0
55 L	Cz-M1	100	2000		10	1000	0
55 L 2	Cz-M1	100	2000		10	1000	0
55 L 3	Cz-M1	100	2000		10	1000	0
50 L	Cz-M1	100	2000		10	1000	0
50 L 2	Cz-M1	100	2000		10	1000	0
40 L	Cz-M1	100	2000		10	1000	0
40 L 2	Cz-M1	100	2000		10	1000	0

ABR: ABR 2 tone burst 4000Hz 1
: Cz-M1

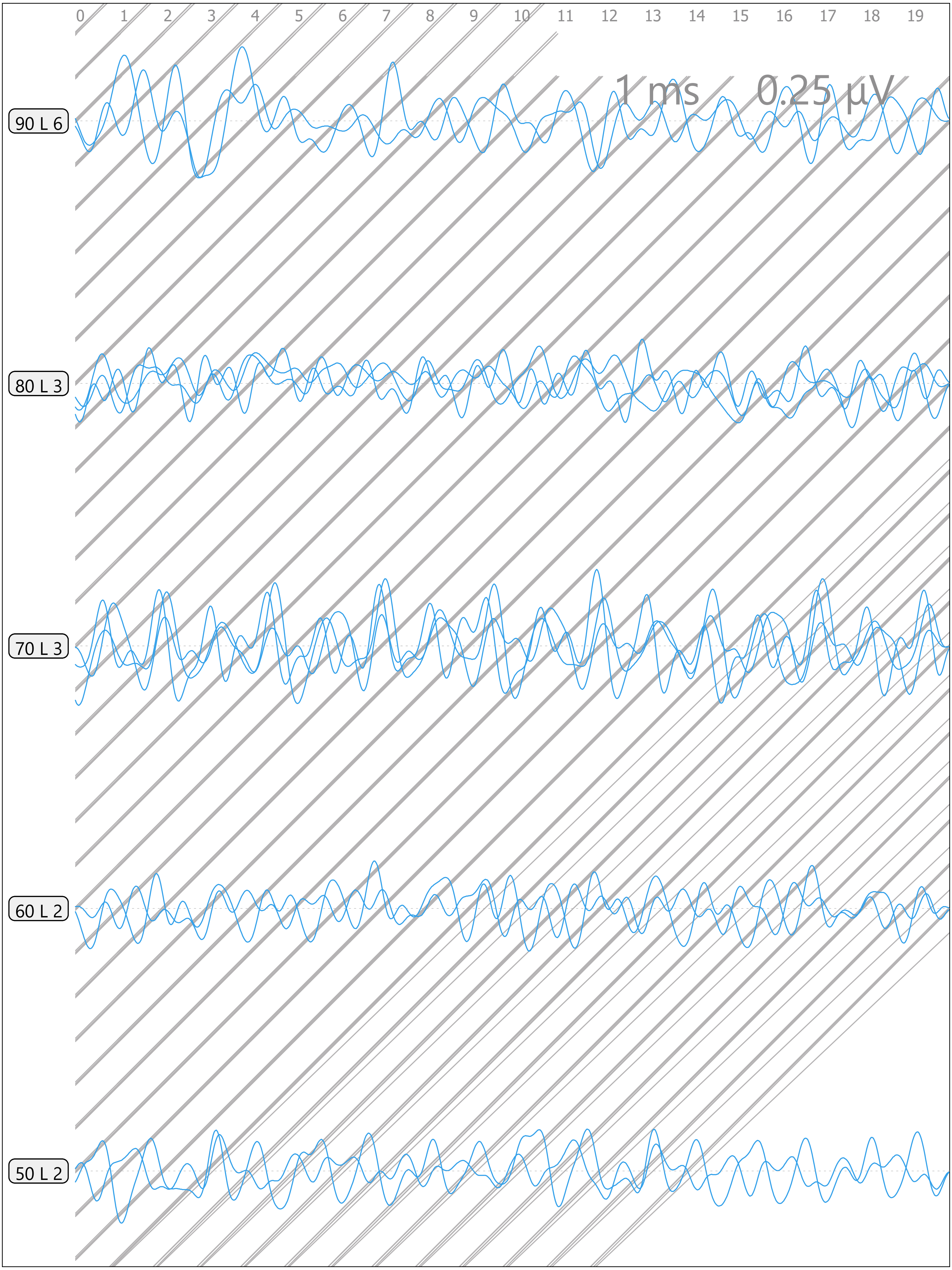

0
1
2
3
4
5
6
7
8
9
10
11
12
13
14
15
16
17
18
19
1 ms
0.25 µV
90 L 6
80 L 3
70 L 3
60 L 2
50 L 2

Trace parameters

N	Electr.	HPF, Hz	LPF, Hz	50 Hz	Rejection ±μV	Aver.	Reject.
90 L 5	Cz-M1	200	2000		10	1000	0
90 L 6	Cz-M1	200	2000		10	1000	0
80 L	Cz-M1	200	2000		10	1000	0
80 L 2	Cz-M1	200	2000		10	1000	0
80 L 3	Cz-M1	200	2000		10	1000	0
70 L	Cz-M1	200	2000		10	1000	0
70 L 2	Cz-M1	200	2000		10	1000	0
70 L 3	Cz-M1	200	2000		10	1000	0
60 L	Cz-M1	200	2000		10	1000	0
60 L 2	Cz-M1	200	2000		10	1000	0
50 L	Cz-M1	200	2000		10	1000	0
50 L 2	Cz-M1	200	2000		10	1000	0

ABR: ABR 2 8000Hz 1: Cz-M1

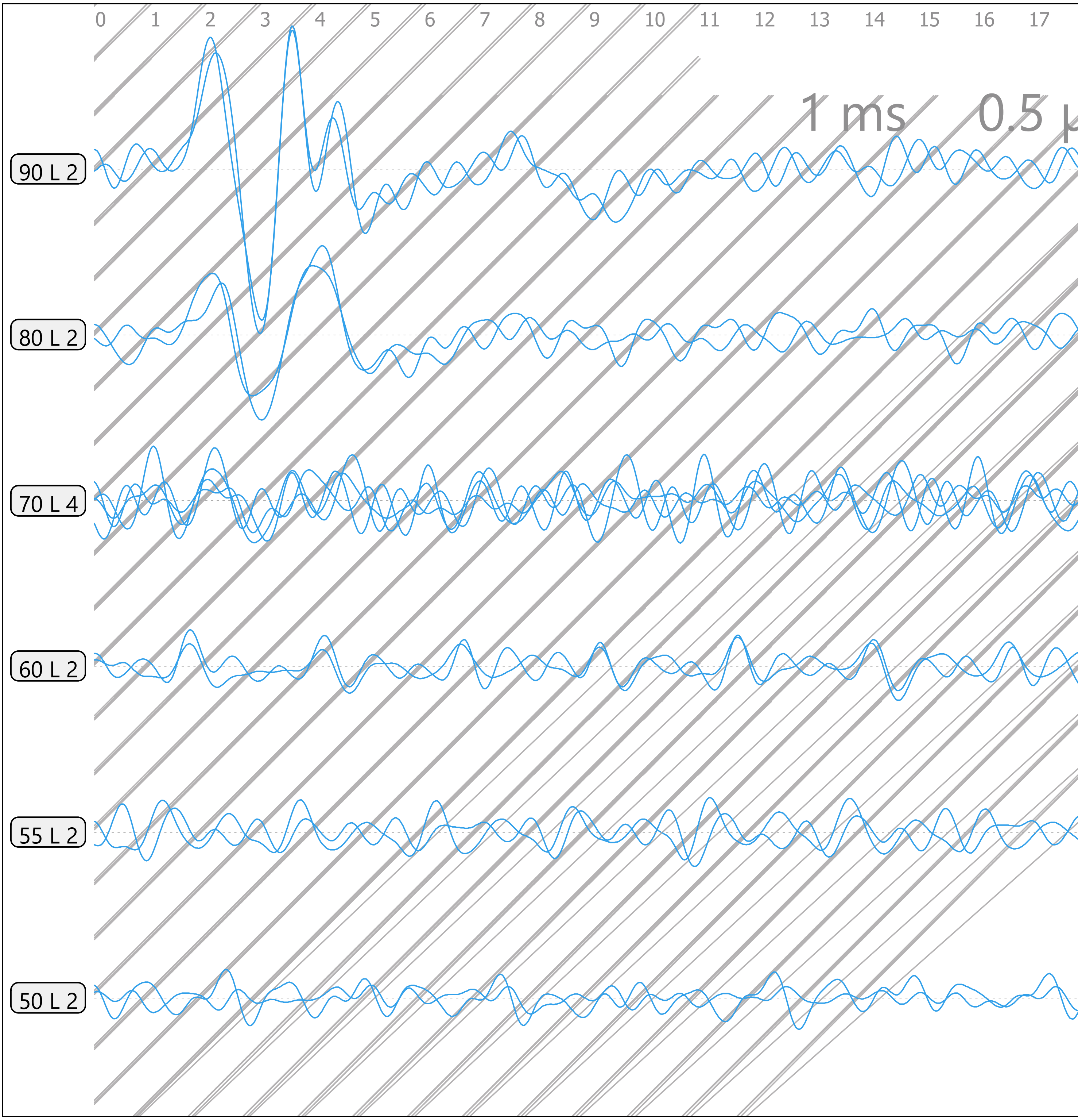

Trace parameters

N	Electr.	HPF, Hz	LPF, Hz	50 Hz	Rejection ±µV	Aver.	Reject.
90 L	Cz-M1	200	2000		10	1000	0
90 L 2	Cz-M1	200	2000		10	1000	0
80 L	Cz-M1	200	2000		10	1000	0
80 L 2	Cz-M1	200	2000		10	1000	0
70 L	Cz-M1	200	2000		10	1000	0
70 L 2	Cz-M1	200	2000		10	1000	0

70 L 3	Cz-M1	200	2000		10	1000	0
70 L 4	Cz-M1	200	2000		10	1000	0
60 L	Cz-M1	200	2000		10	1000	0
60 L 2	Cz-M1	200	2000		10	1000	0
55 L	Cz-M1	200	2000		10	1000	0
55 L 2	Cz-M1	200	2000		10	1000	0
50 L	Cz-M1	200	2000		10	1000	0
50 L 2	Cz-M1	200	2000		10	1000	0

ABR: ABR 2 CLICK
2: Cz-M2

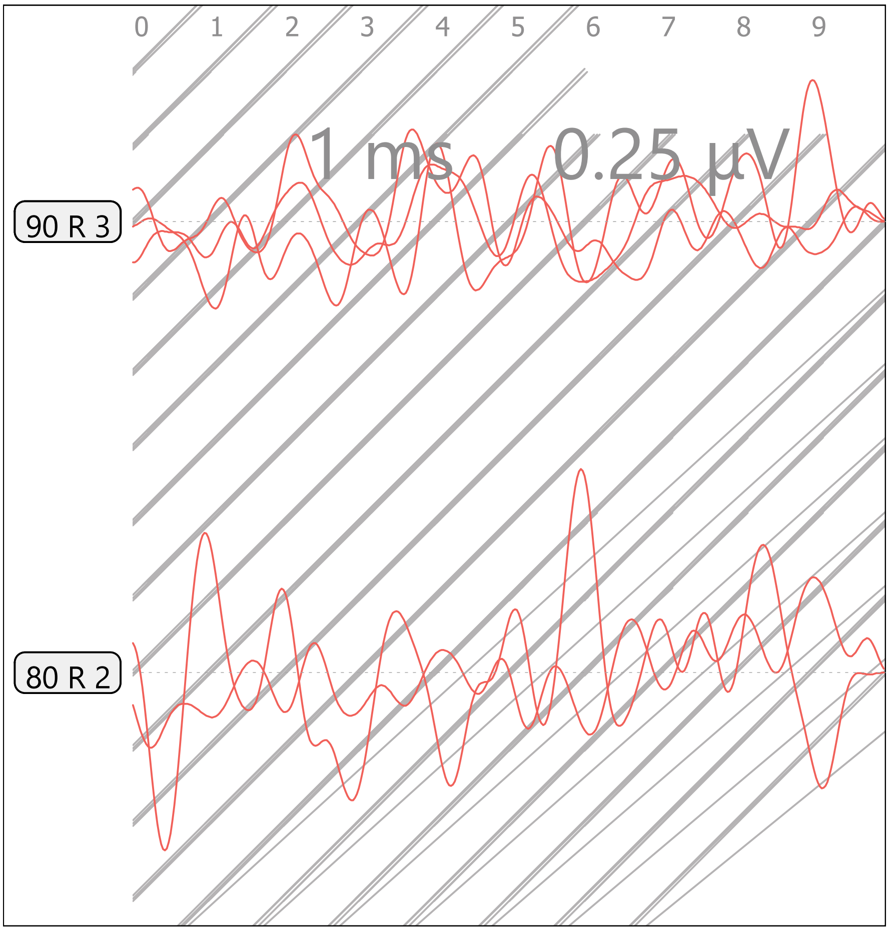

Trace parameters

N	Electr.	HPF, Hz	LPF, Hz	50 Hz	Rejection ±µV	Aver.	Reject
90 R	Cz-M2	100	2000		10	1000	0
90 R 2	Cz-M2	100	2000		10	1000	0
90 R 3	Cz-M2	100	2000		10	1000	0
80 R	Cz-M2	100	2000		10	1000	0
80 R 2	Cz-M2	100	2000		10	1000	0

DPOAE: 1-12 kHz 70/70 dB 3 points

**Test result (right ear):**	

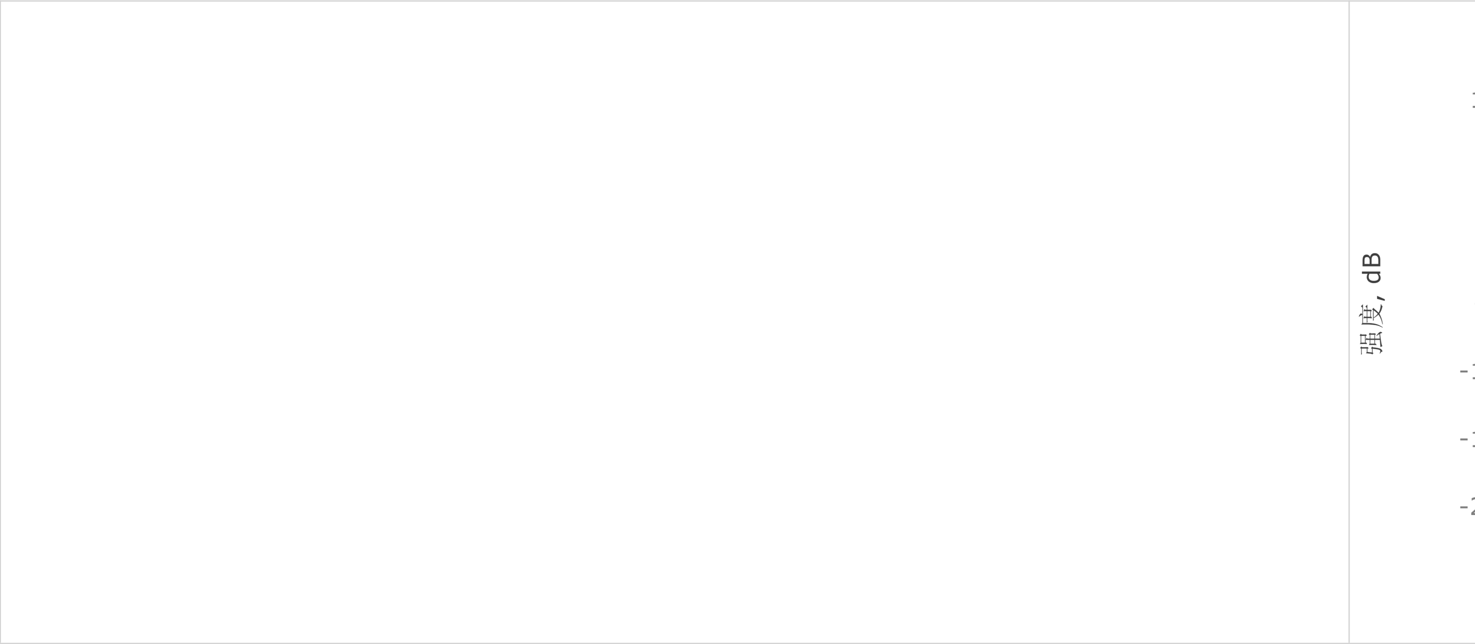

DPOAE (left ear)

F2, Hz	L1, dB	L2, dB	DP, dB	噪声, dB	SNR, dB	OAE
988	68.4	68.4	-18.88	-15.00	-3.9	✘
1270	69.1	69.0	-23.57	-15.00	-8.6	✘
1778	70.1	69.7	-10.71	-15.00	4.3	✘
2222	70.5	70.0	-14.76	-15.00	0.2	✘
2500	70.6	70.1	-16.35	-15.00	-1.4	✘
3200	70.9	70.4	-17.50	-15.00	-2.5	✘
4444	71.3	70.7	-18.20	-15.00	-3.2	✘
5000	71.3	70.5	-5.05	-13.08	8.0	✔
6154	71.1	70.7	-11.04	-15.00	4.0	✘
8000	71.3	69.2	7.54	-9.19	16.7	✔
8889	71.5	67.8	13.47	-7.87	21.3	✔
10000	70.6	61.0	10.08	-8.42	18.5	✔
11429	63.2	62.9	-5.64	-12.04	6.4	✔
(dB SPL)	:: 0.0					

ECochG: ECochG 2: Cz-M2

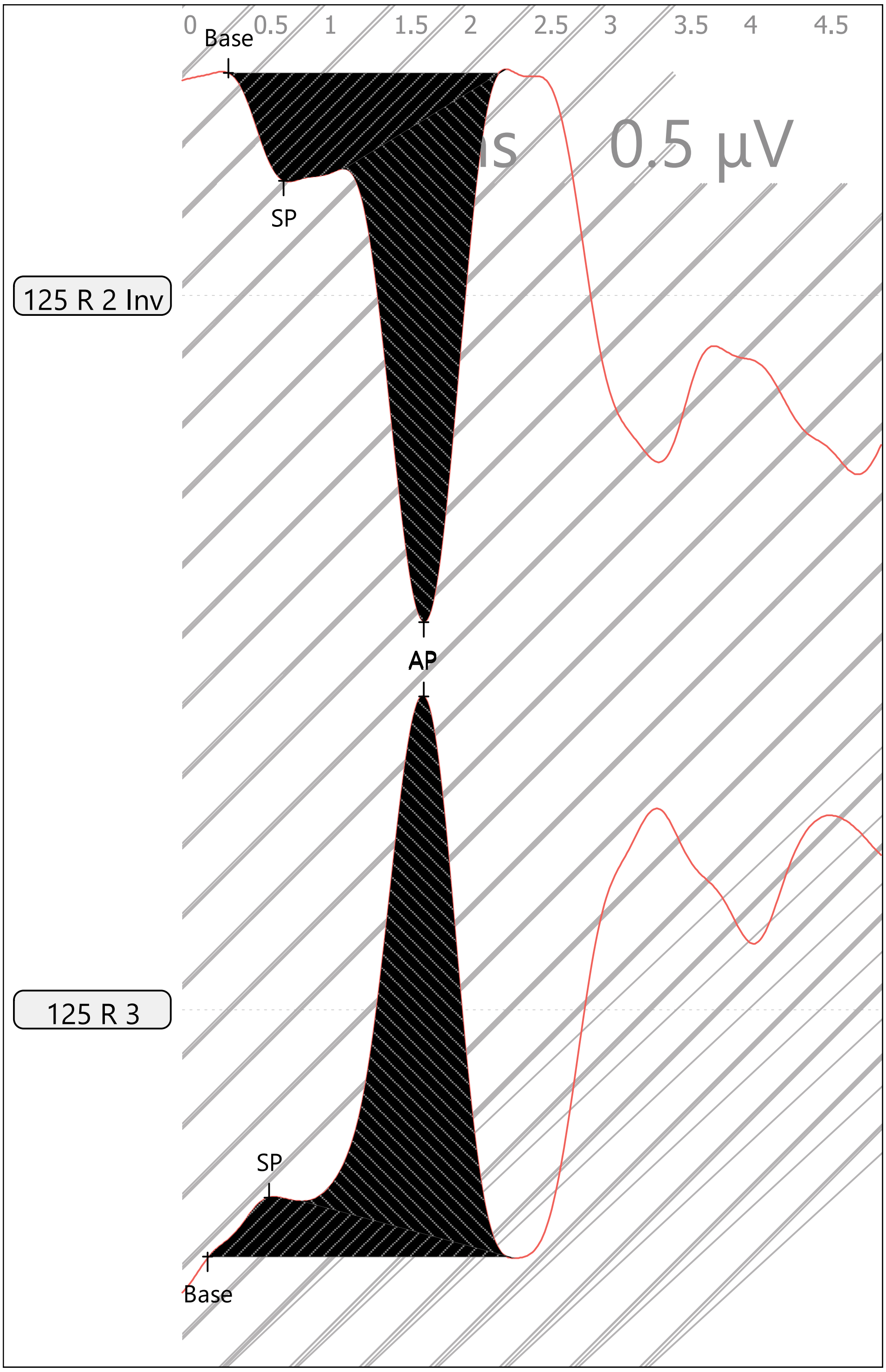

&& (right ear

N	Base (ms)	SP (ms)	AP (ms)	SP–Base (ms)	AP–Base (ms)	SP–Base (µV)	AP–Base (µV)	[illegible]
125 R 2 Inv	0.33	0.73	1.72	0.40	1.39	0.77	3.91	0[illegible]
125 R 3	0.19	0.62	1.72	0.44	1.53	0.43	4.00	0[illegible]

Trace parameters

N	Electr.	HPF, Hz	LPF, Hz	50 Hz	Rejection ±µV	Aver.	R[illegible]
125 R 2 Inv	Cz-M2	5	2000		50	1272	
125 R 3	Cz-M2	5	2000		50	1500	

EcochG: ECochG 1:
Fpz-M1

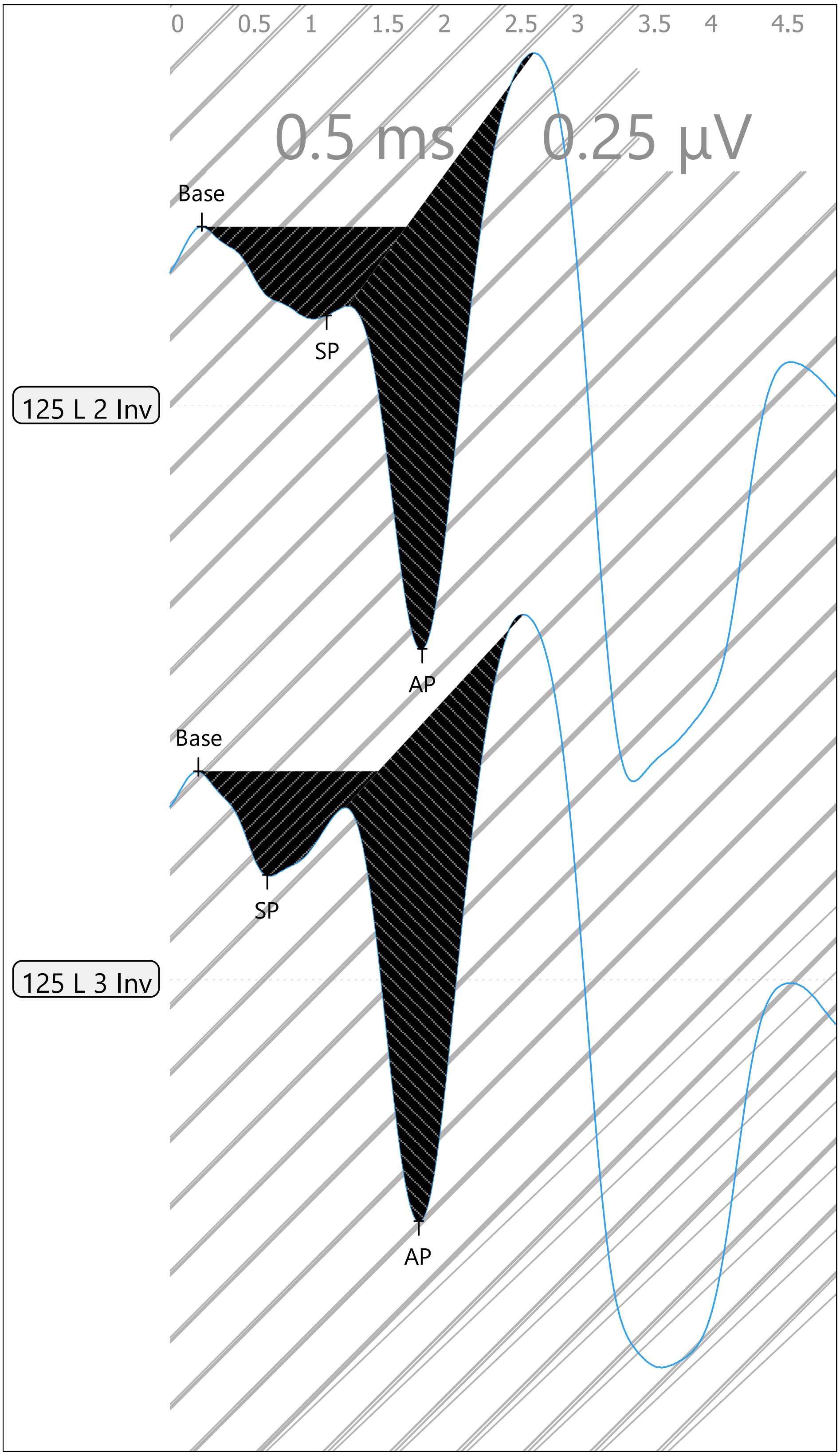

&& (left ear

N	Base (ms)	SP (ms)	AP (ms)	SP–Base (ms)	AP–Base (ms)	SP–Base (µV)	AP–Base (µV)	
125 L 2 Inv	0.24	1.18	1.89	0.94	1.65	0.33	1.58	0
125 L 3 Inv	0.21	0.73	1.87	0.52	1.65	0.39	1.68	0

Trace parameters

N	Electr.	HPF, Hz	LPF, Hz	50 Hz	Rejection ±µV	Aver.	R
125 L 2 Inv	Fpz-M1	5	2000		50	1446	
125 L 3 Inv	Fpz-M1	5	2000		50	1500	

CONCLUSION:

Doctor:
