# Supplementary material for: Exposure to high-altitude hypobaric hypoxic environment induces low-frequency hearing loss in C57BL/6J mice: Mediated by slowing down the postsynaptic electrical signal transmission speed in the cochlear-inferior colliculus auditory signaling pathway
Source: PLoS One. 2026 Mar 11;21(3):e0342321. doi: 10.1371/journal.pone.0342321 (PMC12978441; doi:10.1371/journal.pone.0342321)
Supplement: S1 File — (ZIP) [file pone.0342321.s001.zip › 2025-6-9-3d-2.pdf]

Exam report

Patient: 2025-6-9-3d-2 (-)
Date: June 9, 2025

ABR: ABR 2 tone burst 4000Hz 1
: Cz-M1

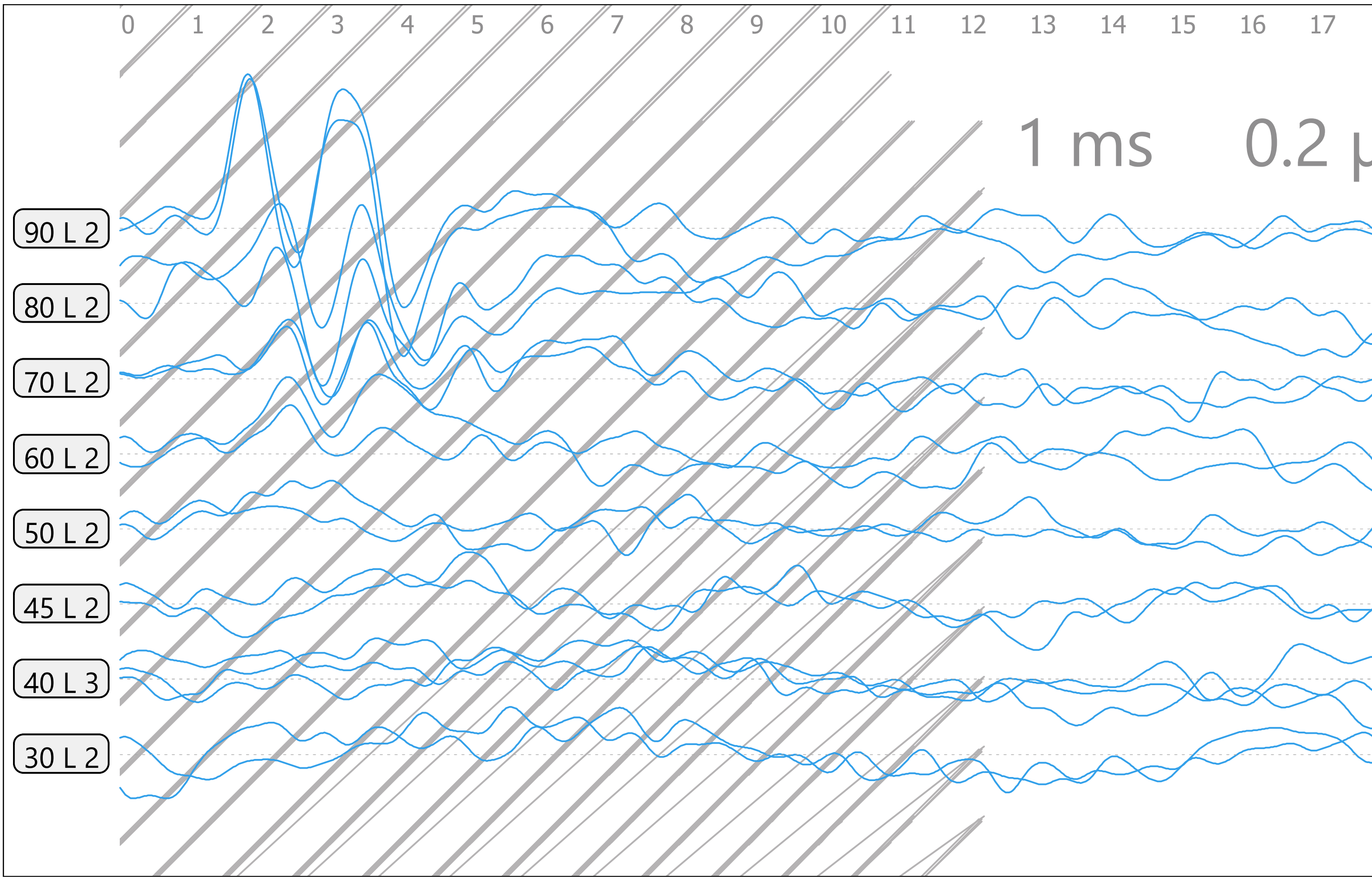

Trace parameters

N	Electr.	HPF, Hz	LPF, Hz	50 Hz	Rejection ±μV	Aver.	Reject.
90 L	Cz-M1	200	2000		10	1000	0
90 L 2	Cz-M1	200	2000		10	1000	0
80 L	Cz-M1	200	2000		10	1000	0
80 L 2	Cz-M1	200	2000		10	1000	0
70 L	Cz-M1	200	2000		10	1000	0
70 L 2	Cz-M1	200	2000		10	1000	0
60 L	Cz-M1	200	2000		10	1000	0
60 L 2	Cz-M1	200	2000		10	1000	0
50 L	Cz-M1	200	2000		10	1000	0
50 L 2	Cz-M1	200	2000		10	1000	0
45 L	Cz-M1	200	2000		10	1000	0
45 L 2	Cz-M1	200	2000		10	1000	0

40 L	Cz-M1	200	2000		10	1000	0
40 L 2	Cz-M1	200	2000		10	1000	0
40 L 3	Cz-M1	200	2000		10	1000	0
30 L	Cz-M1	200	2000		10	1000	0
30 L 2	Cz-M1	200	2000		10	1000	0

ABR: ABR 2 8000Hz 1: Cz-M1

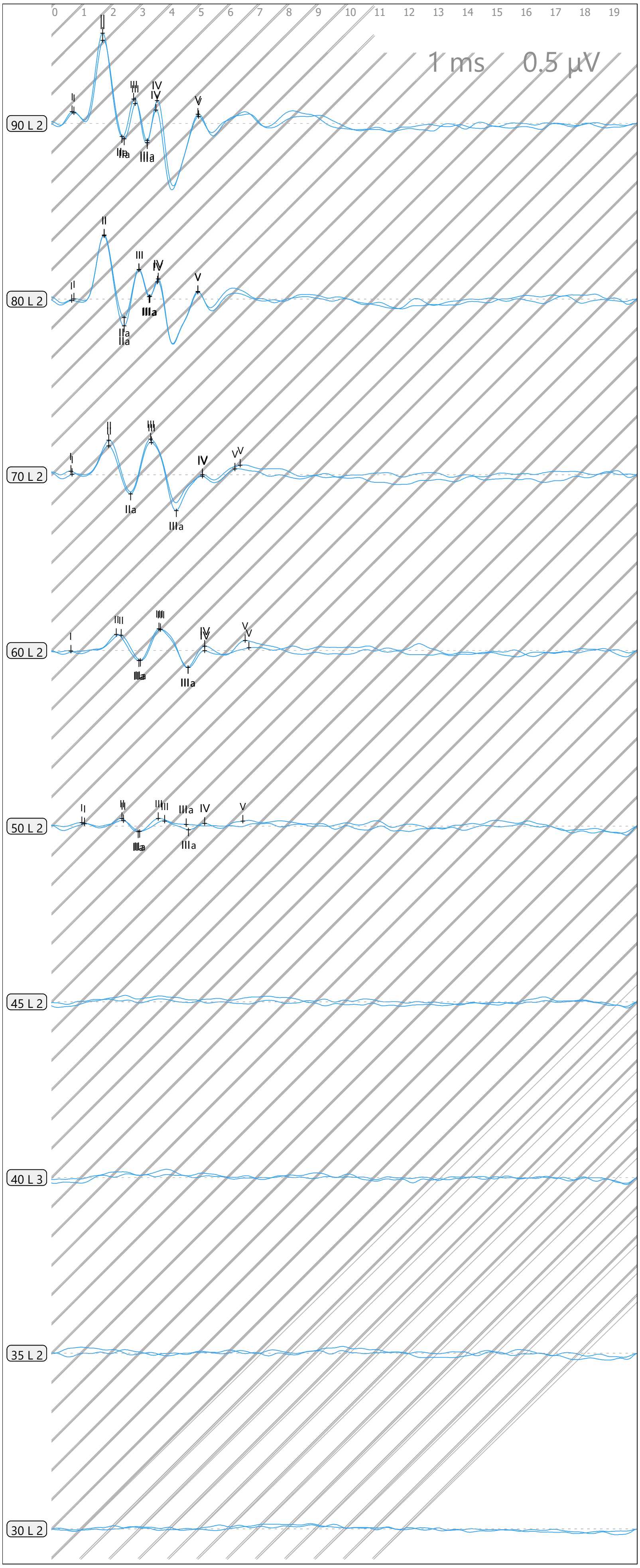

0 1 2 3 4 5 6 7 8 9 10 11 12 13 14 15 16 17 18 19
1 ms
0.5 µV
90 L 2
80 L 2
70 L 2
60 L 2
50 L 2
45 L 2
40 L 3
35 L 2
30 L 2
I
II
IIa
III
IIIa
IV
V

&& (left ear)

N	I (ms)	II (ms)	III (ms)	IV (ms)	V (ms)
90 L	0.71	1.75	2.80	3.57	5.03
90 L 2	0.77	1.75	2.86	3.60	5.00
80 L	0.77	1.80	2.99	3.65	5.00
80 L 2	0.69	1.80	2.99	3.62	5.00
70 L	0.71	1.96	3.39	5.16	6.27
70 L 2	0.66	1.96	3.41	5.16	6.46
60 L	0.66	2.22	3.68	5.24	6.75
60 L 2		2.38	3.73	5.24	6.61
50 L	1.03	2.46	3.86	5.24	6.54
50 L 2	1.14	2.41	3.65		

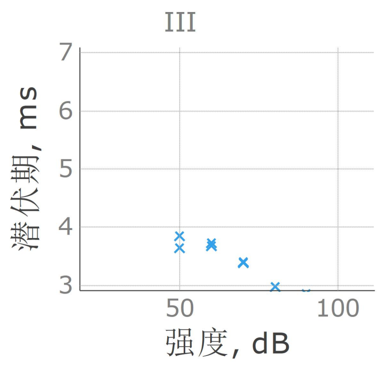

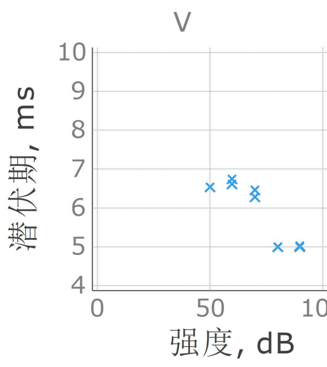

Trace parameters

N	Electr.	HPF, Hz	LPF, Hz	50 Hz	Rejection ±μV	Aver.	Reject.
90 L	Cz-M1	200	2000		10	1000	0
90 L 2	Cz-M1	200	2000		10	1000	0
80 L	Cz-M1	200	2000		10	1000	0
80 L 2	Cz-M1	200	2000		10	1000	0
70 L	Cz-M1	200	2000		10	1000	0
70 L 2	Cz-M1	200	2000		10	1000	0
60 L	Cz-M1	200	2000		10	1000	0
60 L 2	Cz-M1	200	2000		10	1000	0
50 L	Cz-M1	200	2000		10	1000	0
50 L 2	Cz-M1	200	2000		10	1000	0
45 L	Cz-M1	200	2000		10	1000	0
45 L 2	Cz-M1	200	2000		10	1000	0
40 L 2	Cz-M1	200	2000		10	1000	0
40 L 3	Cz-M1	200	2000		10	1000	0
35 L	Cz-M1	200	2000		10	1000	0
35 L 2	Cz-M1	200	2000		10	1000	0
30 L	Cz-M1	200	2000		10	1000	0

30 L 2	Cz-M1	200	2000		10	1000	0

ABR: ABR 2 CLICK 2: Cz-M2

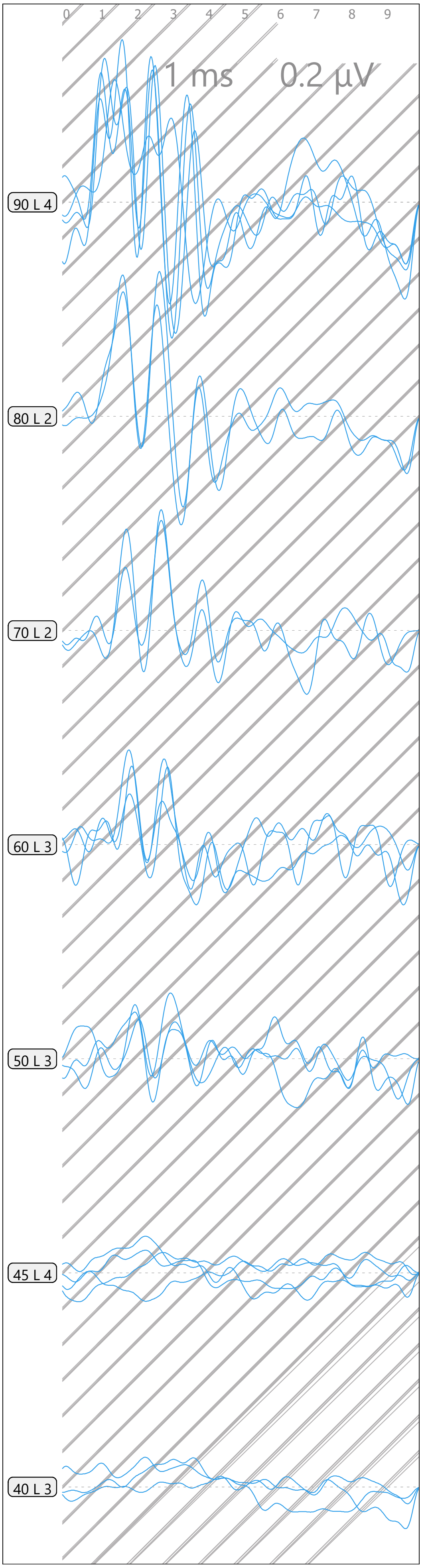
0 1 2 3 4 5 6 7 8 9
1 ms
0.2 µV
90 L 4
80 L 2
70 L 2
60 L 3
50 L 3
45 L 4
40 L 3

Trace parameters

N	Electr.	HPF, Hz	LPF, Hz	50 Hz	Rejection ±μV	Aver.	Reject.
90 L	Cz-M1	100	2000		10	1000	0
90 L 2	Cz-M1	100	2000		10	1000	0
90 L 3	Cz-M1	100	2000		10	1000	0
90 L 4	Cz-M1	100	2000		10	1000	0
80 L	Cz-M1	100	2000		10	1000	0
80 L 2	Cz-M1	100	2000		10	1000	0
70 L	Cz-M1	100	2000		10	1000	0
70 L 2	Cz-M1	100	2000		10	1000	0
60 L	Cz-M1	100	2000		10	1000	0
60 L 2	Cz-M1	100	2000		10	1000	0
60 L 3	Cz-M1	100	2000		10	1000	0
50 L	Cz-M1	100	2000		10	1000	0
50 L 2	Cz-M1	100	2000		10	1000	0
50 L 3	Cz-M1	100	2000		10	1000	0
45 L	Cz-M1	100	2000		10	1000	0
45 L 2	Cz-M1	100	2000		10	1000	0
45 L 3	Cz-M1	100	2000		10	1000	0
45 L 4	Cz-M1	100	2000		10	1000	0
40 L	Cz-M1	100	2000		10	1000	0
40 L 2	Cz-M1	100	2000		10	1000	0
40 L 3	Cz-M1	100	2000		10	1000	0

ABR: ABR 2 CLICK 2: Cz-M2

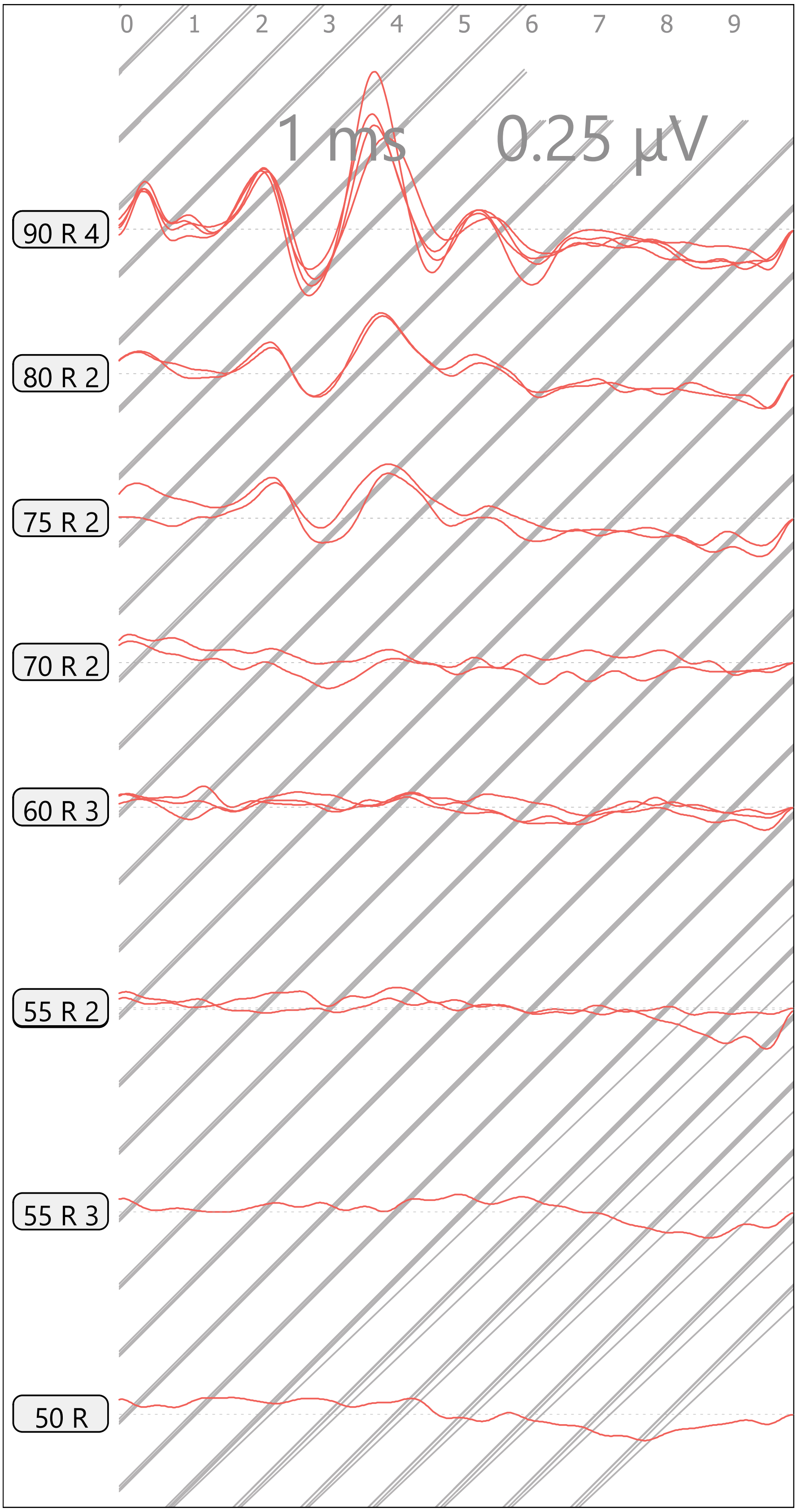

Trace parameters

N	Electr.	HPF, Hz	LPF, Hz	50 Hz	Rejection ±µV	Aver.	Reject
90 R	Cz-M2	100	2000		10	1000	0
90 R 2	Cz-M2	100	2000		10	1000	0
90 R 3	Cz-M2	100	2000		10	1000	0
90 R 4	Cz-M2	100	2000		10	1000	0
80 R	Cz-M2	100	2000		10	1000	1

80 R 2	Cz-M2	100	2000		10	1000	3
75 R	Cz-M2	100	2000		10	1000	0
75 R 2	Cz-M2	100	2000		10	1000	0
70 R	Cz-M2	100	2000		10	1000	2
70 R 2	Cz-M2	100	2000		10	1000	1
60 R	Cz-M2	100	2000		10	1000	1
60 R 2	Cz-M2	100	2000		10	1000	0
60 R 3	Cz-M2	100	2000		10	1000	1
55 R	Cz-M2	100	2000		10	1000	0
55 R 2	Cz-M2	100	2000		10	1000	0
55 R 3	Cz-M2	100	2000		10	1000	0
50 R	Cz-M2	100	2000		10	1000	0

ABR: ABR 2 tone burst 4000Hz 2
: Cz-M2

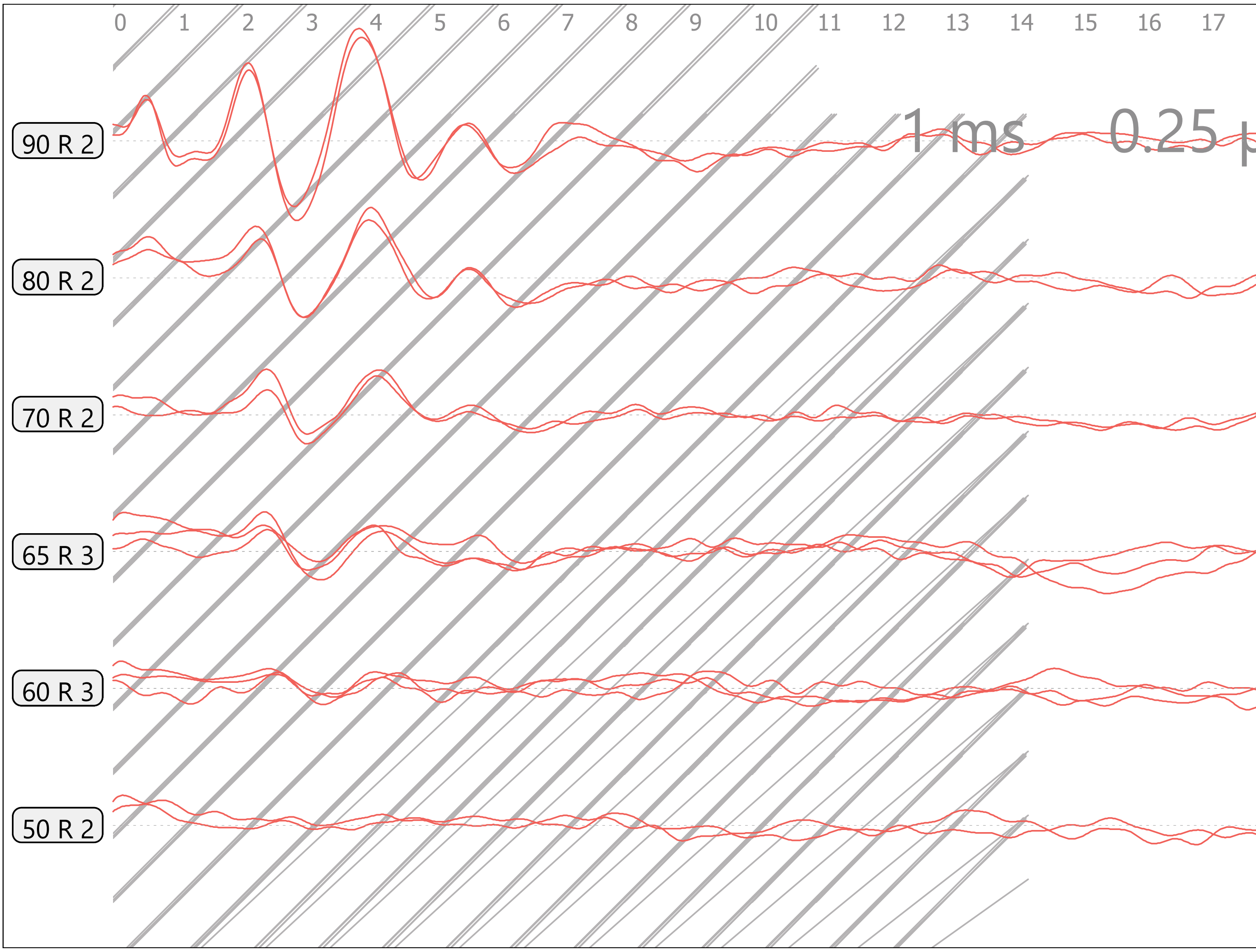

Trace parameters

N	Electr.	HPF, Hz	LPF, Hz	50 Hz	Rejection ±μV	Aver.	Reject
90 R	Cz-M2	200	2000		10	1000	0
90 R 2	Cz-M2	200	2000		10	1000	0
80 R	Cz-M2	200	2000		10	1000	0
80 R 2	Cz-M2	200	2000		10	1000	0

70 R	Cz-M2	200	2000		10	1000	0
70 R 2	Cz-M2	200	2000		10	1000	0
65 R	Cz-M2	200	2000		10	1000	0
65 R 2	Cz-M2	200	2000		10	1000	0
65 R 3	Cz-M2	200	2000		10	1000	0
60 R	Cz-M2	200	2000		10	1000	0
60 R 2	Cz-M2	200	2000		10	1000	0
60 R 3	Cz-M2	200	2000		10	1000	0
50 R	Cz-M2	200	2000		10	1000	0
50 R 2	Cz-M2	200	2000		10	1000	0

ABR: ABR 2 8000Hz 2: Cz-M2

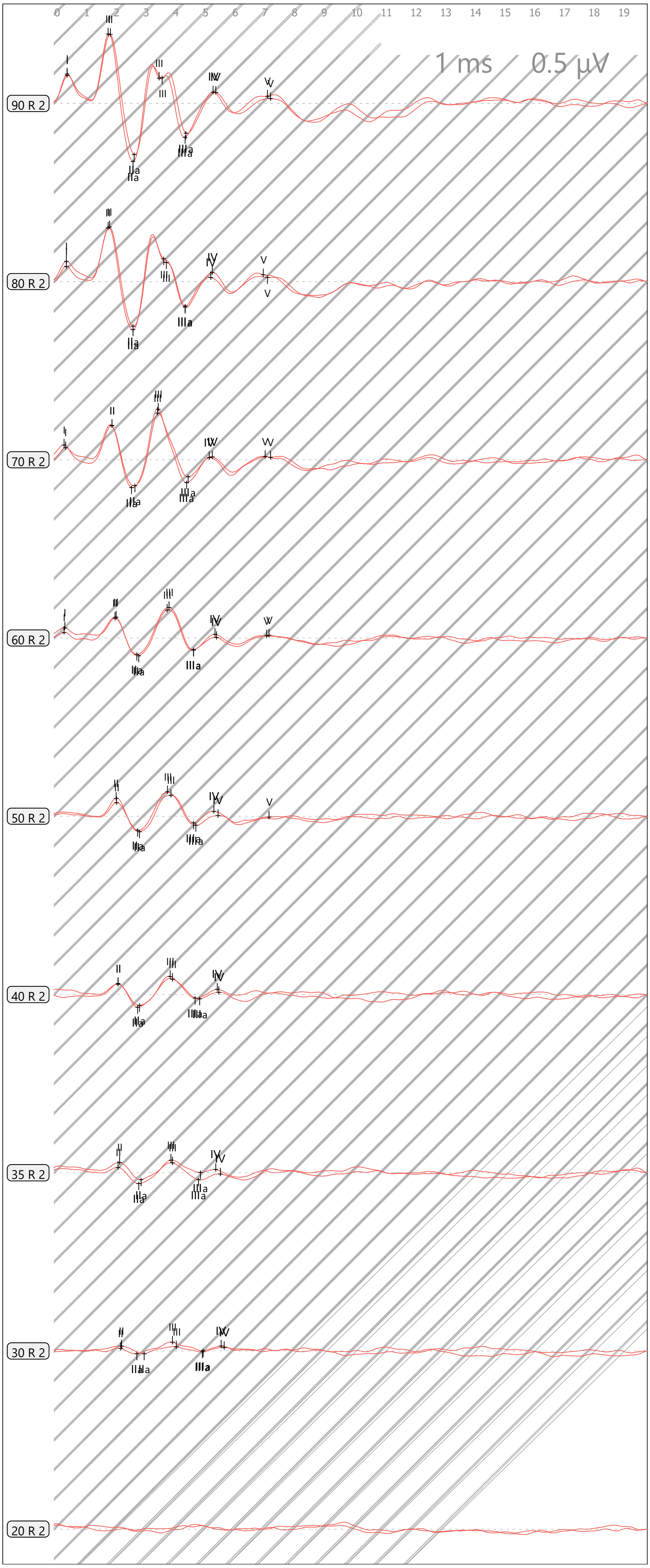
0 1 2 3 4 5 6 7 8 9 10 11 12 13 14 15 16 17 18 19
1 ms
0.5 µV
90 R 2
80 R 2
70 R 2
60 R 2
50 R 2
40 R 2
35 R 2
30 R 2
20 R 2

IV (ms)	V (ms)	I–III (ms)	I–V (ms)	III–V (ms)	
5.37	7.20	3.10	6.75	3.65	
5.45	7.30	3.20	6.85	3.65	
5.34	7.20	3.36	6.77	3.41	
5.29	7.06	3.28	6.64	3.36	
5.34	7.12	3.15	6.77	3.62	
5.24	7.30	3.12	6.91	3.78	
5.42	7.17	3.44	6.80	3.36	
5.48	7.25	3.55	6.91	3.36	
5.53	7.25			3.41	
5.40					
5.56					
5.50					
5.45					
5.61					
5.64					
5.74					

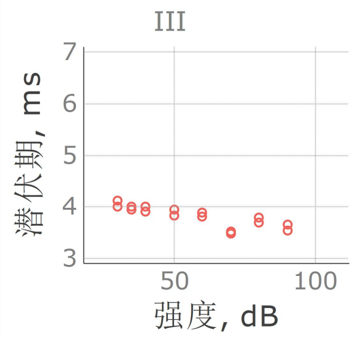

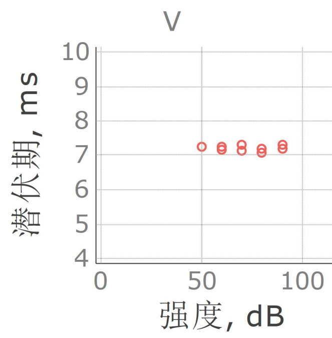

Trace parameters

N	Electr.	HPF, Hz	LPF, Hz	50 Hz	Rejection ±μV	Aver.	Reject
90 R	Cz-M2	200	2000		10	1000	0
90 R 2	Cz-M2	200	2000		10	1000	0
80 R	Cz-M2	200	2000		10	1000	0
80 R 2	Cz-M2	200	2000		10	1000	0
70 R	Cz-M2	200	2000		10	1000	0
70 R 2	Cz-M2	200	2000		10	1000	0
60 R	Cz-M2	200	2000		10	1000	0
60 R 2	Cz-M2	200	2000		10	1000	0
50 R	Cz-M2	200	2000		10	1000	0
50 R 2	Cz-M2	200	2000		10	1000	0
40 R	Cz-M2	200	2000		10	1000	0
40 R 2	Cz-M2	200	2000		10	1000	0
35 R	Cz-M2	200	2000		10	1000	0
35 R 2	Cz-M2	200	2000		10	1000	0

30 R	Cz-M2	200	2000		10	1000	0
30 R 2	Cz-M2	200	2000		10	1000	0
20 R	Cz-M2	200	2000		10	1000	0
20 R 2	Cz-M2	200	2000		10	1000	0

DPOAE: 1-12 kHz 70/70 dB 3 points

Test result (right ear):

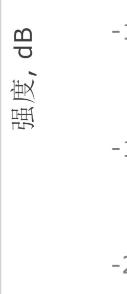

DPOAE

F2, Hz	L1, dB	L2, dB	DP, dB	噪声, dB	SNR, dB	OAE
988	67.9	68.4	-1.26	-3.53	2.3	✘
1270	68.7	69.0	-7.84	-4.11	-3.7	✘
1778	69.5	69.7	-11.23	-6.03	-5.2	✘
2222	70.0	70.0	-8.07	-8.23	0.2	✘
2500	70.1	70.1	-13.04	-13.47	0.4	✘
3200	70.3	68.9	-8.73	-14.73	6.0	✘
4444	70.5	71.0	-22.83	-15.00	-7.8	✘
5000	71.3	70.5	-5.08	-11.34	6.3	✘
6154	70.8	70.7	-16.70	-15.00	-1.7	✘
8000	71.1	70.6	-10.70	-15.00	4.3	✘
8889	70.9	67.0	-16.05	-15.00	-1.1	✘
10000	70.7	51.2	-4.06	-10.25	6.2	✓
11429	51.6	62.3	-10.79	-15.00	4.2	✘
噪音水平 (dB SPL):: 0.0						

DPOAE: 1-12 kHz 70/70 dB 3 points

Test result (right ear): REFER

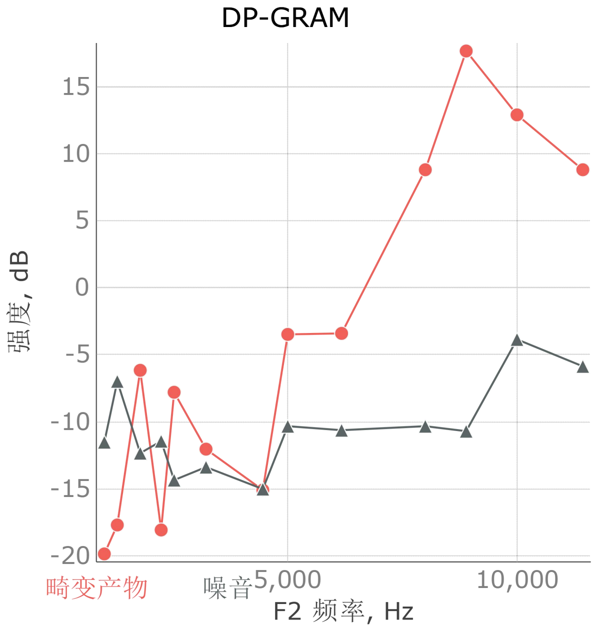

DPOAE

F2, Hz	L1, dB	L2, dB	DP, dB	dB	SNR, dB	OAE
988	68.0	68.5	-19.87	-11.53	-8.3	✗
1270	69.0	69.2	-17.71	-7.01	-10.7	✗
1778	69.7	69.8	-6.18	-12.36	6.2	✗
2222	70.1	70.1	-18.09	-11.49	-6.6	✗
2500	70.2	70.2	-7.83	-14.40	6.6	✗
3200	70.5	70.5	-12.03	-13.37	1.3	✗
4444	70.8	70.8	-15.11	-15.00	-0.1	✗
5000	70.6	70.5	-3.49	-10.32	6.8	✓
6154	70.5	71.1	-3.39	-10.61	7.2	✓
8000	71.3	69.1	8.79	-10.34	19.1	✓
8889	70.5	69.3	17.68	-10.70	28.4	✓
10000	69.2	60.4	12.91	-3.86	16.8	✓
11429	62.4	56.3	8.85	-5.87	14.7	✓
(dB SPL)	:: 0.0					

EcochG: ECochG 1: Cz-M1

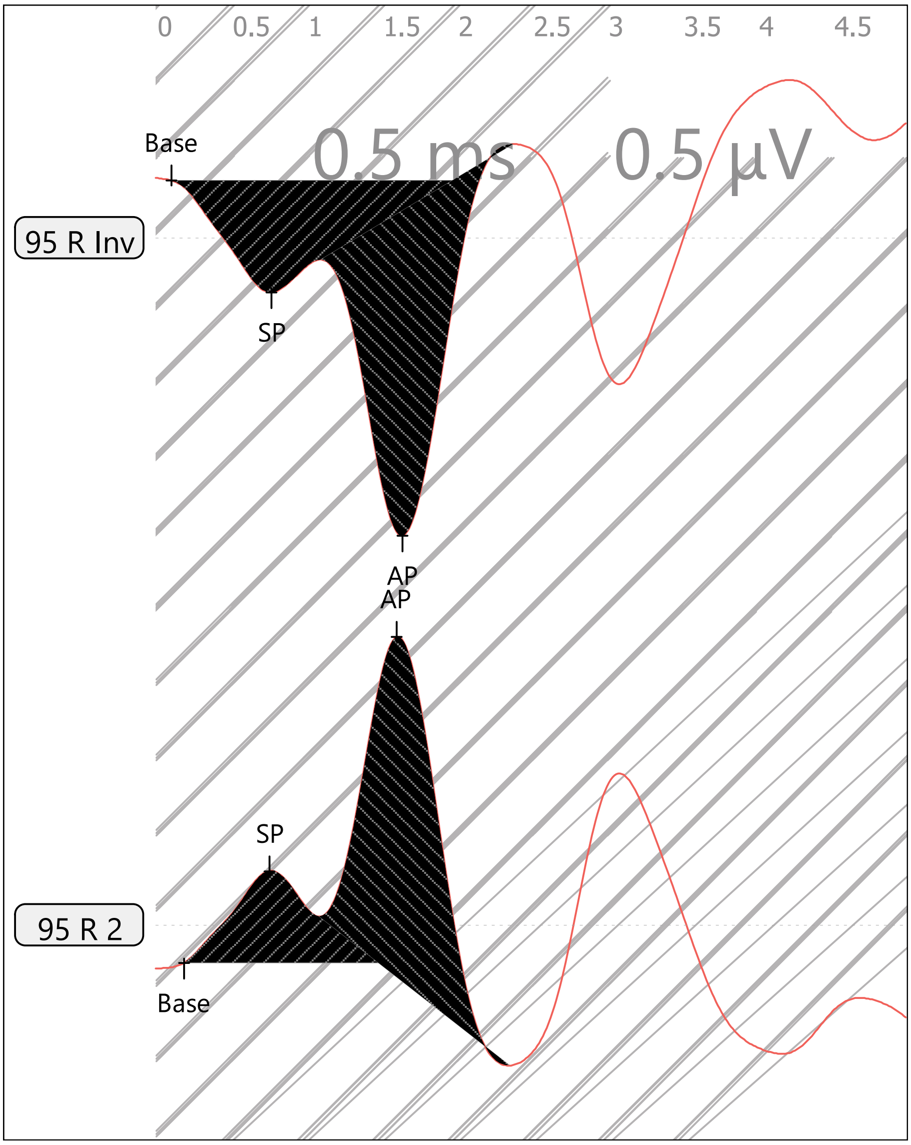

N	Base (ms)	SP (ms)	AP (ms)	SP–Base (ms)	AP–Base (ms)	SP–Base (µV)	AP–Base (µV)
95 R Inv	0.11	0.77	1.64	0.66	1.53	0.74	2.36
95 R 2	0.19	0.75	1.60	0.57	1.42	0.61	2.16

Trace parameters

N	Electr.	HPF, Hz	LPF, Hz	50 Hz	Rejection ±µV	Aver.	Reje
95 R Inv	Fpz-M2	5	2000		50	1500	24
95 R 2	Fpz-M2	5	2000		50	1500	28

EcochG: ECochG 1:
Fpz-M1

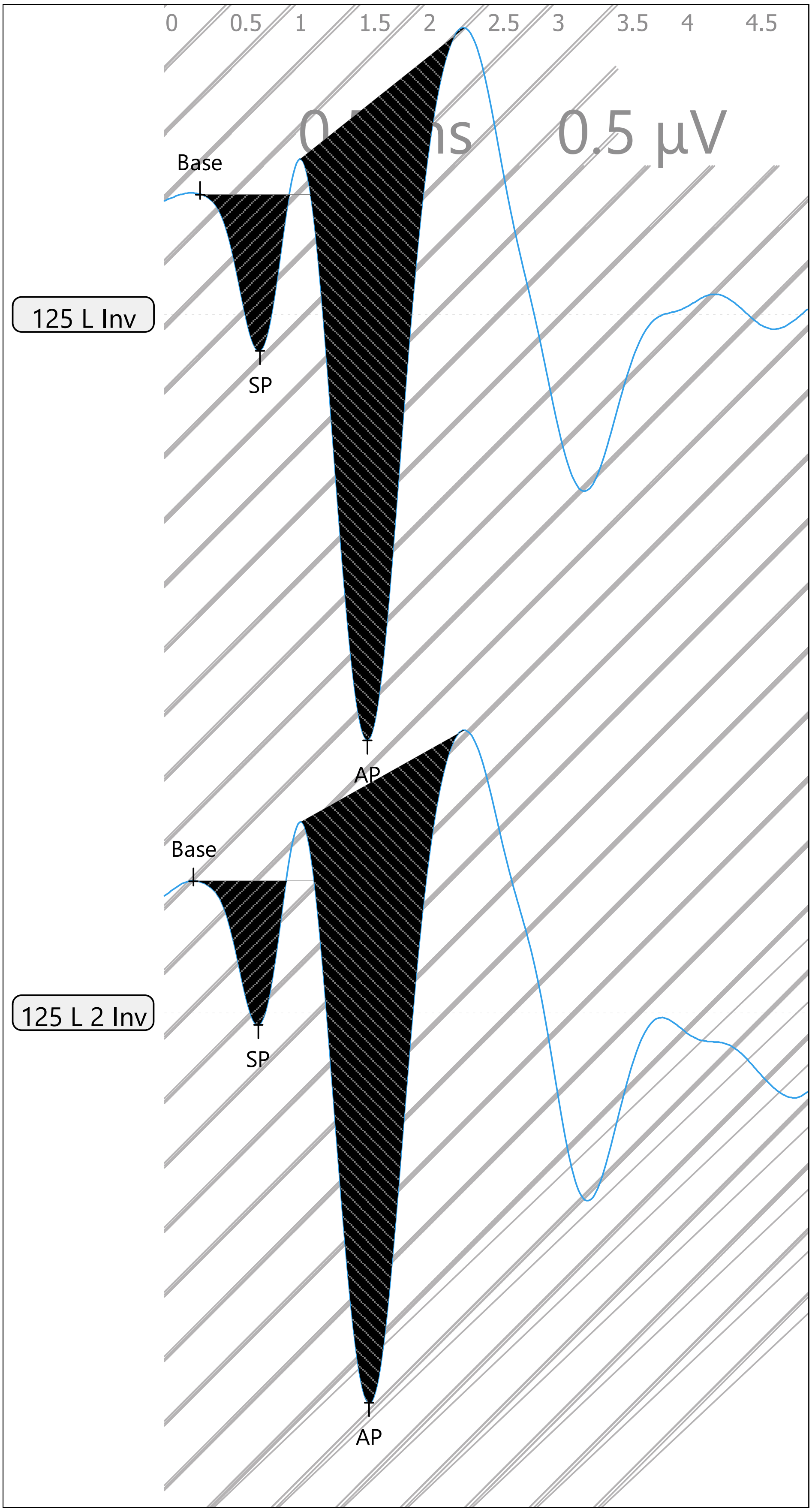

&&

N	Base (ms)	SP (ms)	AP (ms)	SP–Base (ms)	AP–Base (ms)	SP–Base (µV)	AP–Base (µV)	
125 L Inv	0.28	0.74	1.57	0.46	1.30	1.21	4.23	[illegible]
125 L 2 Inv	0.22	0.73	1.59	0.50	1.36	1.12	4.05	[illegible]

Trace parameters

N	Electr.	HPF, Hz	LPF, Hz	50 Hz	Rejection ±μV	Aver.	R[illegible]
125 L Inv	Fpz-M1	5	2000		50	1181	
125 L 2 Inv	Fpz-M1	5	2000		50	1299	

CONCLUSION:

Doctor:
