# Supplementary material for: Exposure to high-altitude hypobaric hypoxic environment induces low-frequency hearing loss in C57BL/6J mice: Mediated by slowing down the postsynaptic electrical signal transmission speed in the cochlear-inferior colliculus auditory signaling pathway
Source: PLoS One. 2026 Mar 11;21(3):e0342321. doi: 10.1371/journal.pone.0342321 (PMC12978441; doi:10.1371/journal.pone.0342321)
Supplement: S1 File — (ZIP) [file pone.0342321.s001.zip › 2025-6-9-3d-3.pdf]

Exam report

Patient: 2025-6-9-3d-3- (-)
Date: June 10, 2025

ABR: ABR 2 CLICK 1: Cz-M1

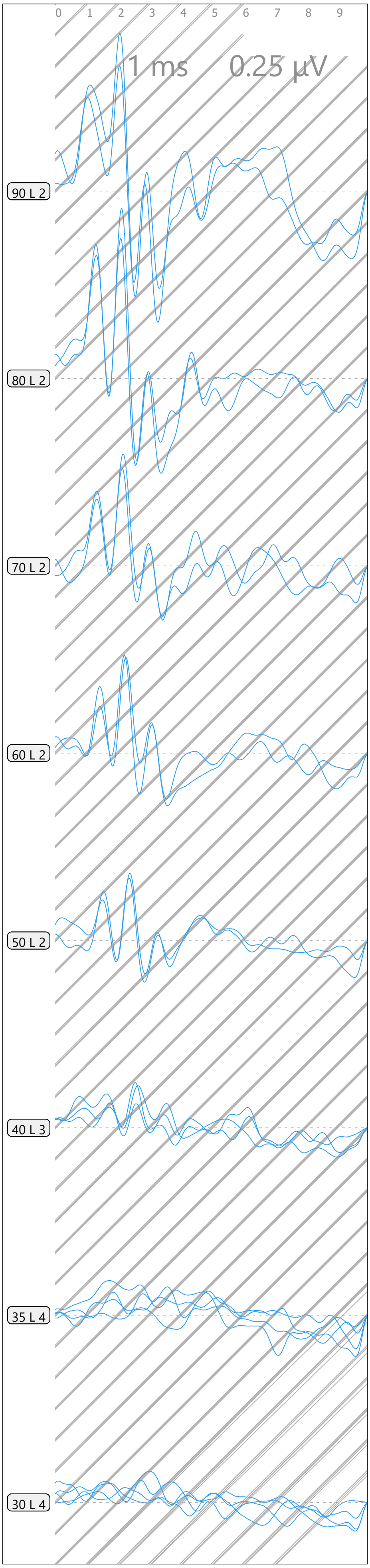
0 1 2 3 4 5 6 7 8 9
1 ms
0.25 µV
90 L 2
80 L 2
70 L 2
60 L 2
50 L 2
40 L 3
35 L 4
30 L 4

Trace parameters

N	Electr.	HPF, Hz	LPF, Hz	50 Hz	Rejection ±μV	Aver.	Reject.
90 L	Cz-M1	100	2000		10	1000	0
90 L 2	Cz-M1	100	2000		10	1000	0
80 L	Cz-M1	100	2000		10	1000	0
80 L 2	Cz-M1	100	2000		10	1000	0
70 L	Cz-M1	100	2000		10	1000	0
70 L 2	Cz-M1	100	2000		10	1000	0
60 L	Cz-M1	100	2000		10	1000	0
60 L 2	Cz-M1	100	2000		10	1000	0
50 L	Cz-M1	100	2000		10	1000	0
50 L 2	Cz-M1	100	2000		10	1000	0
40 L	Cz-M1	100	2000		10	1000	0
40 L 2	Cz-M1	100	2000		10	1000	0
40 L 3	Cz-M1	100	2000		10	1000	0
35 L	Cz-M1	100	2000		10	1000	0
35 L 2	Cz-M1	100	2000		10	1000	0
35 L 3	Cz-M1	100	2000		10	1000	0
35 L 4	Cz-M1	100	2000		10	1000	0
30 L	Cz-M1	100	2000		10	1000	0
30 L 2	Cz-M1	100	2000		10	1000	0
30 L 3	Cz-M1	100	2000		10	1000	0
30 L 4	Cz-M1	100	2000		10	1000	0

ABR: ABR 2 tone burst 4000Hz 1

: Cz-M1

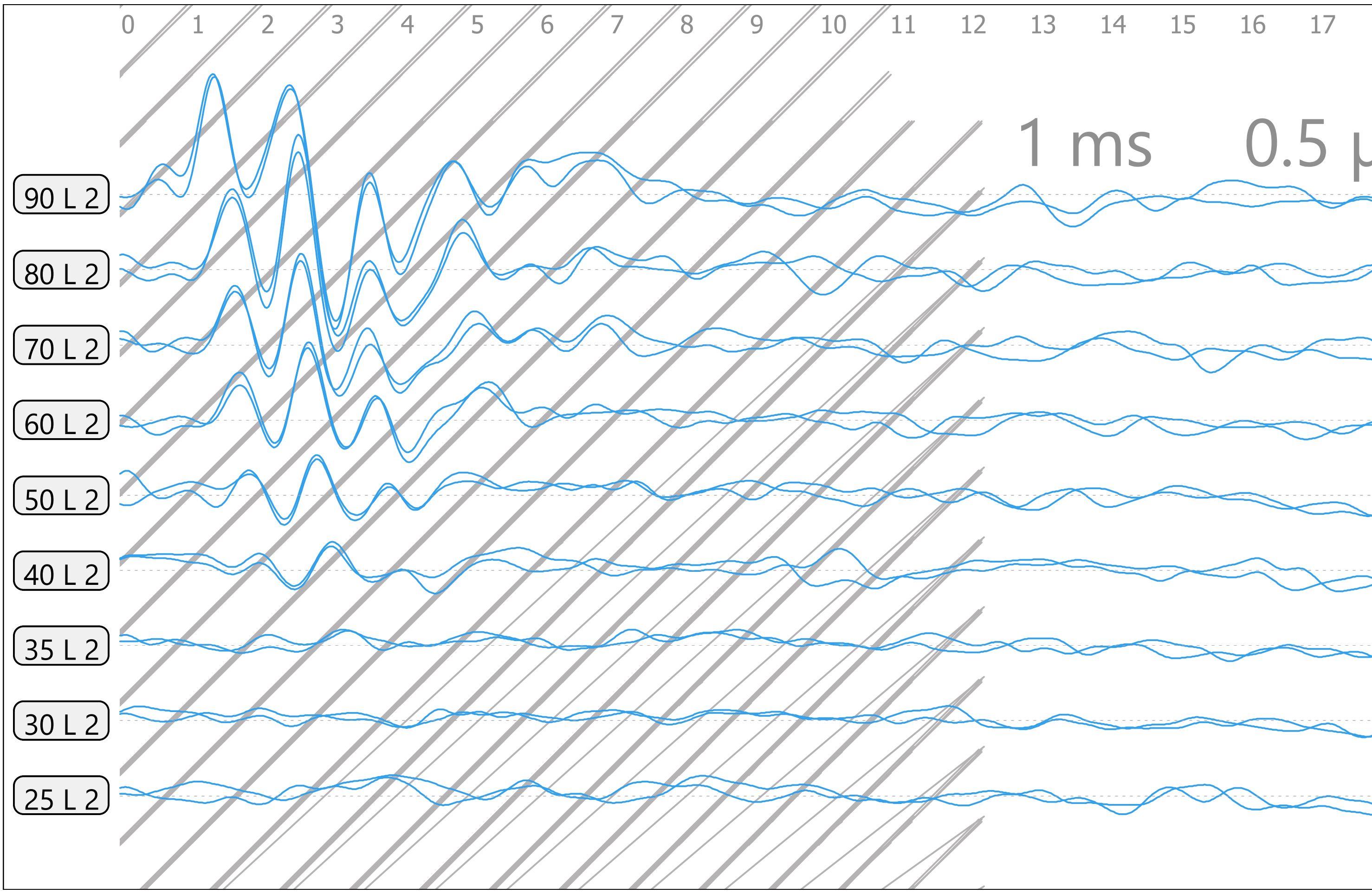

Trace parameters

N	Electr.	HPF, Hz	LPF, Hz	50 Hz	Rejection ±μV	Aver.	Reject.

90 L	Cz-M1	200	2000		10	1000	0
90 L 2	Cz-M1	200	2000		10	1000	0
80 L	Cz-M1	200	2000		10	1000	0
80 L 2	Cz-M1	200	2000		10	1000	0
70 L	Cz-M1	200	2000		10	1000	0
70 L 2	Cz-M1	200	2000		10	1000	0
60 L	Cz-M1	200	2000		10	1000	0
60 L 2	Cz-M1	200	2000		10	1000	0
50 L	Cz-M1	200	2000		10	1000	0
50 L 2	Cz-M1	200	2000		10	1000	0
40 L	Cz-M1	200	2000		10	1000	0
40 L 2	Cz-M1	200	2000		10	1000	0
35 L	Cz-M1	200	2000		10	1000	0
35 L 2	Cz-M1	200	2000		10	1000	0
30 L	Cz-M1	200	2000		10	1000	0
30 L 2	Cz-M1	200	2000		10	1000	0
25 L	Cz-M1	200	2000		10	1000	0
25 L 2	Cz-M1	200	2000		10	1000	0

ABR: ABR 2 8000Hz 1: Cz-M1

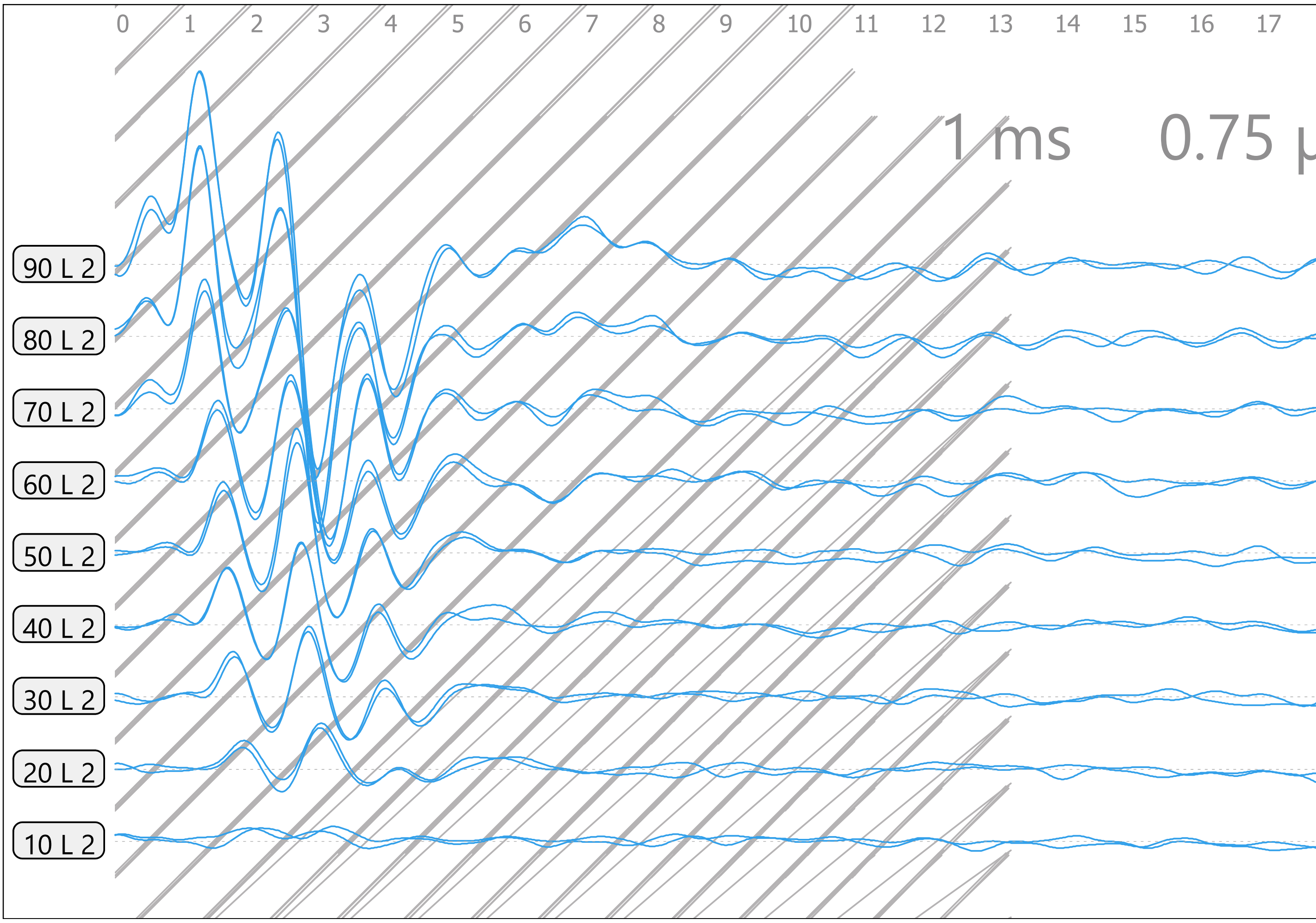

Trace parameters

N	Electr.	HPF, Hz	LPF, Hz	50 Hz	Rejection ±μV	Aver.	Reject.
90 L	Cz-M1	200	2000		10	1000	0
90 L 2	Cz-M1	200	2000		10	1000	0
80 L	Cz-M1	200	2000		10	1000	0
80 L 2	Cz-M1	200	2000		10	1000	0
70 L	Cz-M1	200	2000		10	1000	0
70 L 2	Cz-M1	200	2000		10	1000	0
60 L	Cz-M1	200	2000		10	1000	0
60 L 2	Cz-M1	200	2000		10	1000	0
50 L	Cz-M1	200	2000		10	1000	0
50 L 2	Cz-M1	200	2000		10	1000	0
40 L	Cz-M1	200	2000		10	1000	0
40 L 2	Cz-M1	200	2000		10	1000	0
30 L	Cz-M1	200	2000		10	1000	0
30 L 2	Cz-M1	200	2000		10	1000	0
20 L	Cz-M1	200	2000		10	1000	0

20 L 2	Cz-M1	200	2000		10	1000	0
10 L	Cz-M1	200	2000		10	1000	0
10 L 2	Cz-M1	200	2000		10	1000	0

ABR: ABR 2 CLICK 2: Cz-M2

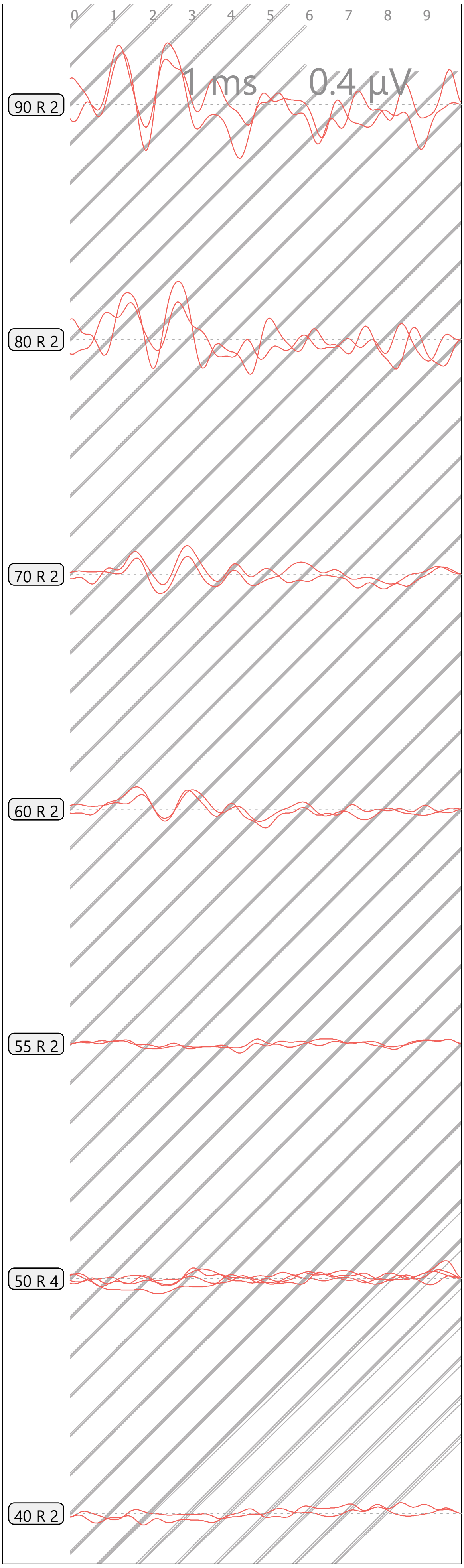

0
1
2
3
4
5
6
7
8
9
1 ms
0.4 µV
90 R 2
80 R 2
70 R 2
60 R 2
55 R 2
50 R 4
40 R 2

Trace parameters

N	Electr.	HPF, Hz	LPF, Hz	50 Hz	Rejection ±μV	Aver.	Reject
90 R	Cz-M2	100	2000		10	900	0
90 R 2	Cz-M2	100	2000		10	1000	0
80 R	Cz-M2	100	2000		10	1000	0
80 R 2	Cz-M2	100	2000		10	1000	0
70 R	Cz-M2	100	2000		10	1000	0
70 R 2	Cz-M2	100	2000		10	1000	0
60 R	Cz-M2	100	2000		10	1000	0
60 R 2	Cz-M2	100	2000		10	1000	0
55 R	Cz-M2	100	2000		10	1000	0
55 R 2	Cz-M2	100	2000		10	1000	0
50 R	Cz-M2	100	2000		10	1000	0
50 R 2	Cz-M2	100	2000		10	1000	0
50 R 3	Cz-M2	100	2000		10	1000	0
50 R 4	Cz-M2	100	2000		10	1000	0
40 R	Cz-M2	100	2000		10	1000	0
40 R 2	Cz-M2	100	2000		10	1000	0

ABR: ABR 2 tone burst 4000Hz 2
: Cz-M2

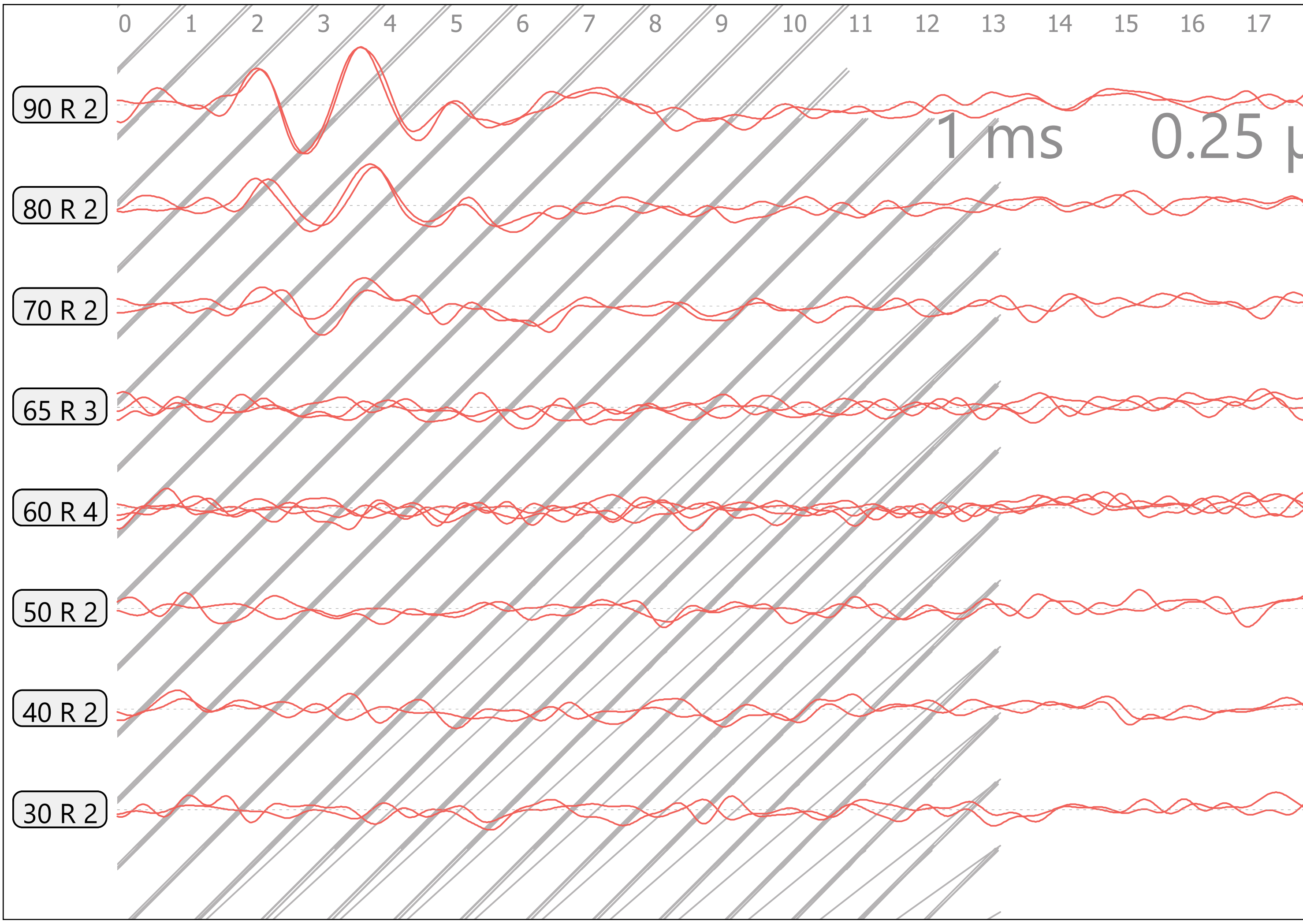

Trace parameters

N	Electr.	HPF, Hz	LPF, Hz	50 Hz	Rejection ±μV	Aver.	Reject
90 R	Cz-M2	200	2000		10	1000	0
90 R 2	Cz-M2	200	2000		10	1000	0

80 R	Cz-M2	200	2000		10	1000	0
80 R 2	Cz-M2	200	2000		10	1000	0
70 R	Cz-M2	200	2000		10	1000	0
70 R 2	Cz-M2	200	2000		10	1000	0
65 R	Cz-M2	200	2000		10	1000	0
65 R 2	Cz-M2	200	2000		10	1000	0
65 R 3	Cz-M2	200	2000		10	1000	0
60 R	Cz-M2	200	2000		10	1000	0
60 R 2	Cz-M2	200	2000		10	1000	0
60 R 3	Cz-M2	200	2000		10	1000	0
60 R 4	Cz-M2	200	2000		10	1000	0
50 R	Cz-M2	200	2000		10	1000	0
50 R 2	Cz-M2	200	2000		10	1000	0
40 R	Cz-M2	200	2000		10	1000	0
40 R 2	Cz-M2	200	2000		10	1000	0
30 R	Cz-M2	200	2000		10	1000	0
30 R 2	Cz-M2	200	2000		10	1000	0

DPOAE: 1-12 kHz 70/70 dB 3 points

Test result (right ear):

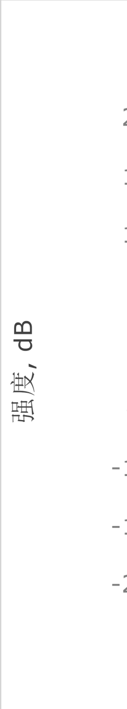

DPOAE

F2, Hz	L1, dB	L2, dB	DP, dB	dB	SNR, dB	OAE
988	68.1	68.4	-14.30	-8.15	-6.2	✘

1270	68.8	69.1	-23.30	-8.02	-15.3	✘
1778	69.6	69.7	-3.61	-11.63	8.0	✔
2222	70.0	70.0	-10.28	-15.00	4.7	✘
2500	70.1	70.1	-14.76	-15.00	0.2	✘
3200	70.4	70.4	-14.21	-15.00	0.8	✘
4444	70.9	70.7	-20.33	-15.00	-5.3	✘
5000	70.8	70.2	0.22	-10.10	10.3	✔
6154	70.5	70.4	6.64	-7.83	14.5	✔
8000	70.3	70.3	2.99	-9.06	12.1	✔
8889	69.9	69.4	12.93	-8.76	21.7	✔
10000	70.8	60.4	19.74	-2.06	21.8	✔
11429	62.7	56.6	18.77	-4.91	23.7	✔
(dB SPL)	:: 0.0					

ECochG: ECochG
1: Fpz-M1

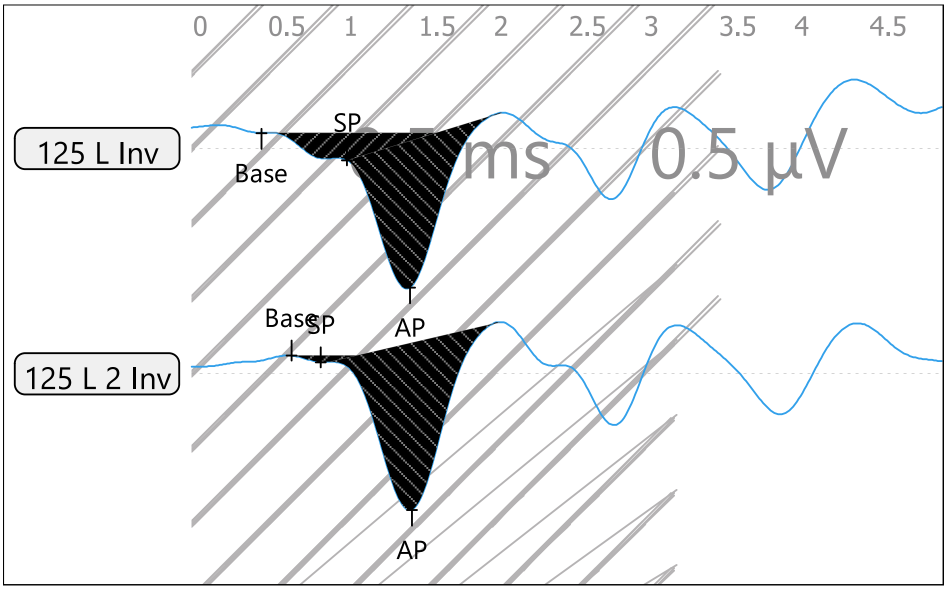

&&

N	Base (ms)	SP (ms)	AP (ms)	SP–Base (ms)	AP–Base (ms)	SP–Base (µV)	AP–Base (µV)	
125 L Inv	0.46	1.03	1.46	0.57	0.99	0.18	1.03	0
125 L 2 Inv	0.66	0.86	1.47	0.20	0.81	0.04	1.03	0

Trace parameters

N	Electr.	HPF, Hz	LPF, Hz	50 Hz	Rejection ±µV	Aver.	R
125 L Inv	Fpz-M1	5	2000		50	1500	
125 L 2 Inv	Fpz-M1	5	2000		50	1116	

CONCLUSION:

Doctor:
