# Supplementary material for: Exposure to high-altitude hypobaric hypoxic environment induces low-frequency hearing loss in C57BL/6J mice: Mediated by slowing down the postsynaptic electrical signal transmission speed in the cochlear-inferior colliculus auditory signaling pathway
Source: PLoS One. 2026 Mar 11;21(3):e0342321. doi: 10.1371/journal.pone.0342321 (PMC12978441; doi:10.1371/journal.pone.0342321)
Supplement: S1 File — (ZIP) [file pone.0342321.s001.zip › 2025.05.22-04, norma.pdf]

Exam report

Patient: 2025.05.22-04**,** normal- (-)
Date: May 23, 2025

ABR: ABR 2 channel ion channel CLICK
1: Cz-M1

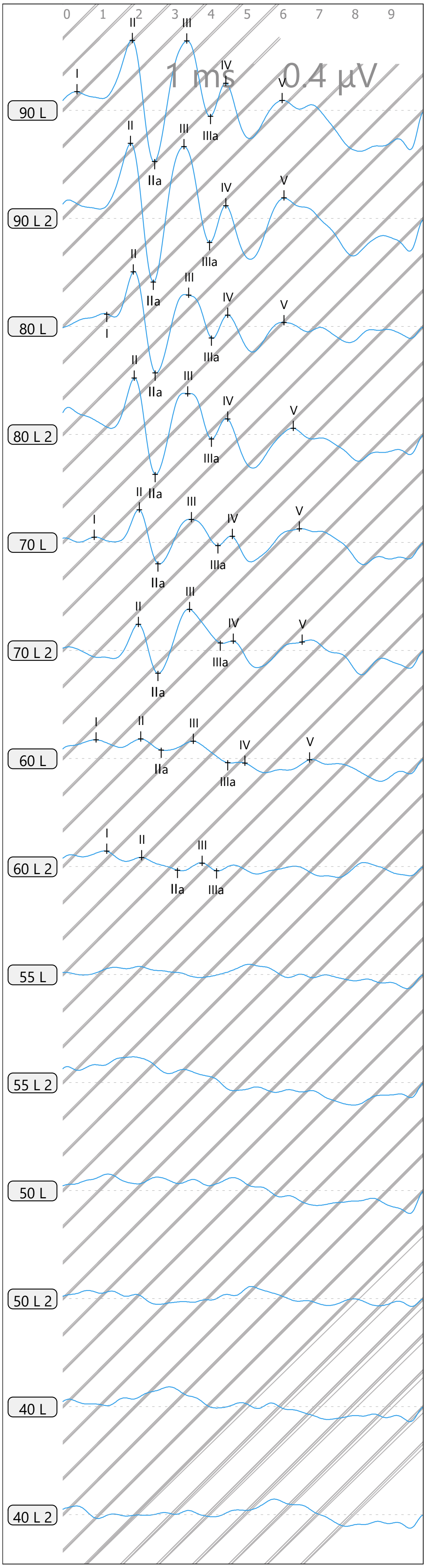
0 1 2 3 4 5 6 7 8 9
1 ms
0.4 µV
90 L
90 L 2
80 L
80 L 2
70 L
70 L 2
60 L
60 L 2
55 L
55 L 2
50 L
50 L 2
40 L
40 L 2
I
II
IIa
III
IIIa
IV
V

latency && aplitude (left ear

N	I (ms)	II (ms)	III (ms)	IV (ms)
90 L	0.40	1.93	3.44	4.52
90 L 2		1.88	3.36	4.52
80 L	1.22	1.96	3.49	4.58
80 L 2		1.98	3.47	4.58
70 L	0.87	2.12	3.57	4.71
70 L 2		2.09	3.52	4.74
60 L	0.93	2.17	3.62	5.05
60 L 2	1.22	2.20	3.86	

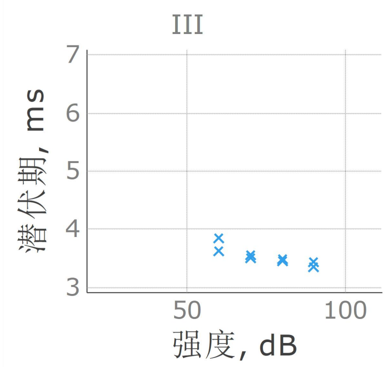

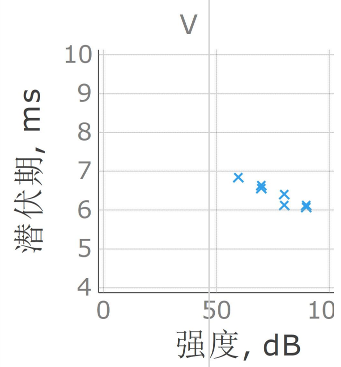

Trace parameters

N	Electr.	HPF, Hz	LPF, Hz	50 Hz	Rejection ±μV	Aver.	Reject.
90 L	Cz-M1	100	2000		10	1024	0
90 L 2	Cz-M1	100	2000		10	1024	0
80 L	Cz-M1	100	2000		10	1024	0
80 L 2	Cz-M1	100	2000		10	1024	0
70 L	Cz-M1	100	2000		10	1024	0
70 L 2	Cz-M1	100	2000		10	1024	0
60 L	Cz-M1	100	2000		10	1024	0
60 L 2	Cz-M1	100	2000		10	1024	0
55 L	Cz-M1	100	2000		10	1024	0
55 L 2	Cz-M1	100	2000		10	1024	0
50 L	Cz-M1	100	2000		10	1024	0
50 L 2	Cz-M1	100	2000		10	1024	0
40 L	Cz-M1	100	2000		10	1024	0
40 L 2	Cz-M1	100	2000		10	1024	0

ABR: ABR 2 4000Hz 1: Cz-M1

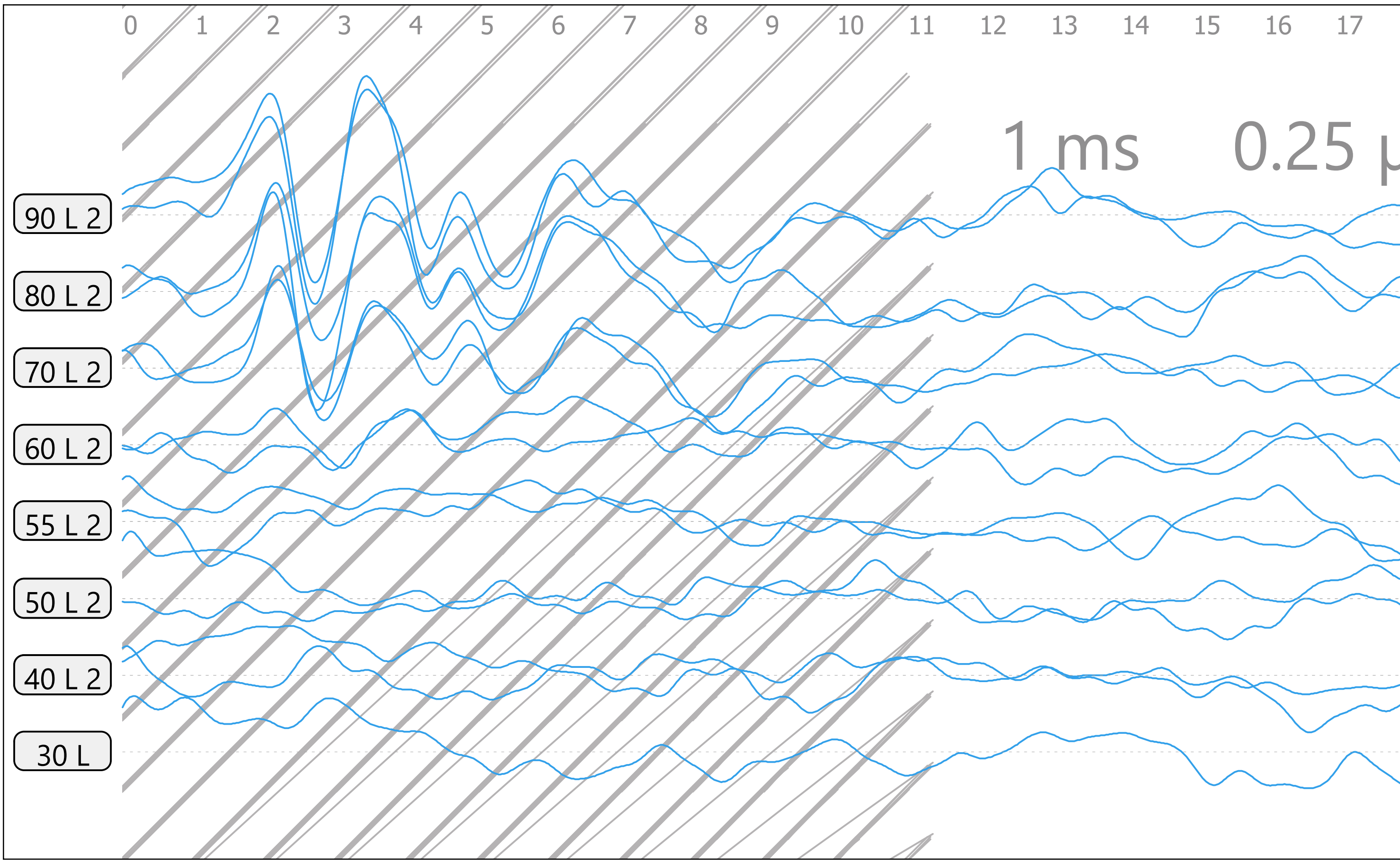

Trace parameters

N	Electr.	HPF, Hz	LPF, Hz	50 Hz	Rejection ±μV	Aver.	Reject.
90 L	Cz-M1	200	2000		10	1000	0
90 L 2	Cz-M1	200	2000		10	1000	0
80 L	Cz-M1	200	2000		10	1000	0
80 L 2	Cz-M1	200	2000		10	1000	0
70 L	Cz-M1	200	2000		10	1000	0
70 L 2	Cz-M1	200	2000		10	1000	0
60 L	Cz-M1	200	2000		10	1000	0
60 L 2	Cz-M1	200	2000		10	1003	0
55 L	Cz-M1	200	2000		10	1000	0
55 L 2	Cz-M1	200	2000		10	1000	0
50 L	Cz-M1	200	2000		10	1000	0
50 L 2	Cz-M1	200	2000		10	1000	0
40 L	Cz-M1	200	2000		10	1000	0
40 L 2	Cz-M1	200	2000		10	1000	0
30 L	Cz-M1	200	2000		10	1000	0

ABR: ABR 2 6000Hz 1: Cz-M1

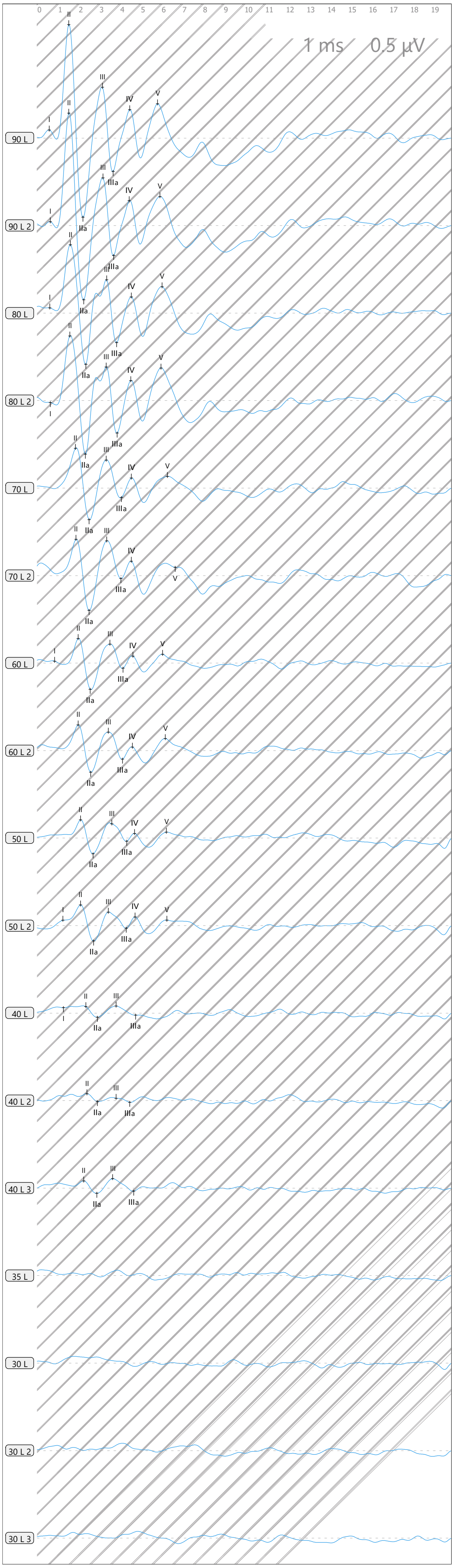
0 1 2 3 4 5 6 7 8 9 10 11 12 13 14 15 16 17 18 19
1 ms
0.5 µV
90 L
90 L 2
80 L
80 L 2
70 L
70 L 2
60 L
60 L 2
50 L
50 L 2
40 L
40 L 2
40 L 3
35 L
30 L
30 L 2
30 L 3

&&

N	I (ms)	II (ms)	III (ms)	IV (ms)	V (ms)
90 L	0.58	1.53	3.15	4.47	5.82
90 L 2	0.64	1.53	3.18	4.45	5.93
80 L	0.61	1.61	3.36	4.55	6.03
80 L 2	0.64	1.59	3.33	4.52	5.98
70 L		1.85	3.33	4.55	6.30
70 L 2		1.88	3.36	4.55	6.67
60 L	0.85	1.98	3.52	4.63	6.06
60 L 2		1.98	3.44	4.60	6.19
50 L		2.09	3.60	4.71	6.24
50 L 2	1.24	2.09	3.44	4.74	6.27
40 L	1.27	2.35	3.81		
40 L 2		2.41	3.81		
40 L 3		2.25	3.65		

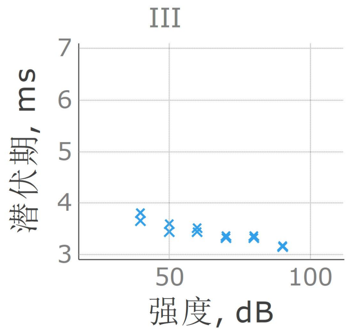

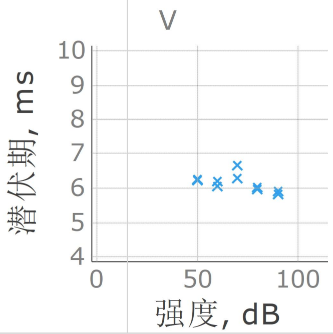

Trace parameters

N	Electr.	HPF, Hz	LPF, Hz	50 Hz	Rejection ±μV	Aver.	Reject.
90 L	Cz-M1	200	2000		10	788	0
90 L 2	Cz-M1	200	2000		10	759	0
80 L	Cz-M1	200	2000		10	1000	0
80 L 2	Cz-M1	200	2000		10	730	0
70 L	Cz-M1	200	2000		10	721	0
70 L 2	Cz-M1	200	2000		10	717	0
60 L	Cz-M1	200	2000		10	727	0
60 L 2	Cz-M1	200	2000		10	815	0
50 L	Cz-M1	200	2000		10	1000	0
50 L 2	Cz-M1	200	2000		10	662	0
40 L	Cz-M1	200	2000		10	1000	0
40 L 2	Cz-M1	200	2000		10	1000	0
40 L 3	Cz-M1	200	2000		10	1000	0
35 L	Cz-M1	200	2000		10	1000	0
30 L	Cz-M1	200	2000		10	1000	0
30 L 2	Cz-M1	200	2000		10	1000	0

30 L 3	Cz-M1	200	2000		10	1000	0

ABR: ABR 2 tone burst 8000Hz 1: Cz-M1

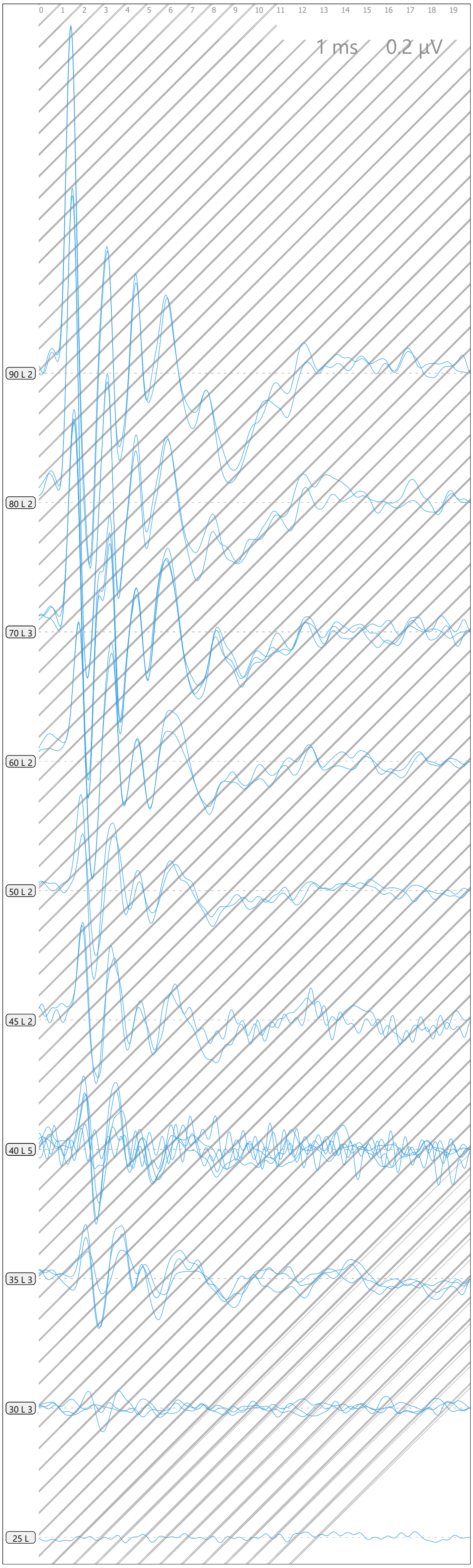
0 1 2 3 4 5 6 7 8 9 10 11 12 13 14 15 16 17 18 19
1 ms
0.2 µV
90 L 2
80 L 2
70 L 3
60 L 2
50 L 2
45 L 2
40 L 5
35 L 3
30 L 3
25 L

Trace parameters

N	Electr.	HPF, Hz	LPF, Hz	50 Hz	Rejection ±µV	Aver.	Reject.
90 L	Cz-M1	200	2000		10	1000	0
90 L 2	Cz-M1	200	2000		10	721	0
80 L	Cz-M1	200	2000		10	975	0
80 L 2	Cz-M1	200	2000		10	693	0
70 L	Cz-M1	200	2000		10	1000	0
70 L 2	Cz-M1	200	2000		10	1000	0
70 L 3	Cz-M1	200	2000		10	740	0
60 L	Cz-M1	200	2000		10	1000	0
60 L 2	Cz-M1	200	2000		10	945	0
50 L	Cz-M1	200	2000		10	1000	0
50 L 2	Cz-M1	200	2000		10	1000	0
45 L	Cz-M1	200	2000		10	1000	0
45 L 2	Cz-M1	200	2000		10	968	0
40 L	Cz-M1	200	2000		10	1000	0
40 L 2	Cz-M1	200	2000		10	872	0
40 L 3	Cz-M1	200	2000		10	1000	0
40 L 4	Cz-M1	200	2000		10	1000	0
40 L 5	Cz-M1	200	2000		10	1000	0
35 L	Cz-M1	200	2000		10	1000	0
35 L 2	Cz-M1	200	2000		10	1000	0
35 L 3	Cz-M1	200	2000		10	1000	0
30 L	Cz-M1	200	2000		10	1000	0
30 L 2	Cz-M1	200	2000		10	1000	0
30 L 3	Cz-M1	200	2000		10	1000	0
25 L	Cz-M1	200	2000		10	1000	0

DPOAE: 1-12 kHz 70/70 dB 3 points

**Test result (right ear):**	

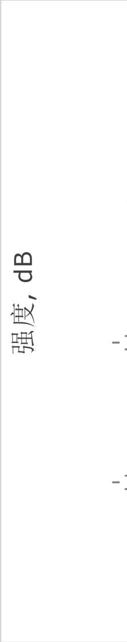

DPOAE (left ear)

F2, Hz	L1, dB	L2, dB	DP, dB	dB	SNR, dB	OAE
988	67.9	68.3	-9.58	-11.61	2.0	✘
1270	68.8	69.1	-2.06	-0.13	-1.9	✘
1778	69.6	69.7	-5.86	0.33	-6.2	✘
2222	70.0	70.0	-13.63	-11.00	-2.6	✘
2500	70.1	70.1	-8.79	-15.00	6.2	✓
3200	70.4	70.2	0.33	-0.14	0.5	✘
4444	70.7	70.6	-7.72	-1.03	-6.7	✘
5000	70.8	70.4	-1.74	-8.49	6.8	✓
6154	70.6	70.8	-17.58	-15.00	-2.6	✘
8000	70.8	71.4	-11.38	-15.00	3.6	✘
8889	70.7	69.5	-7.43	-13.44	6.0	✓
10000	71.9	53.0	-3.71	-12.04	8.3	✓
11429	54.2	56.2	-15.24	-15.00	-0.2	✘
(dB SPL)	:: 0.0					

ECochG: ECochG 2: Cz-M2

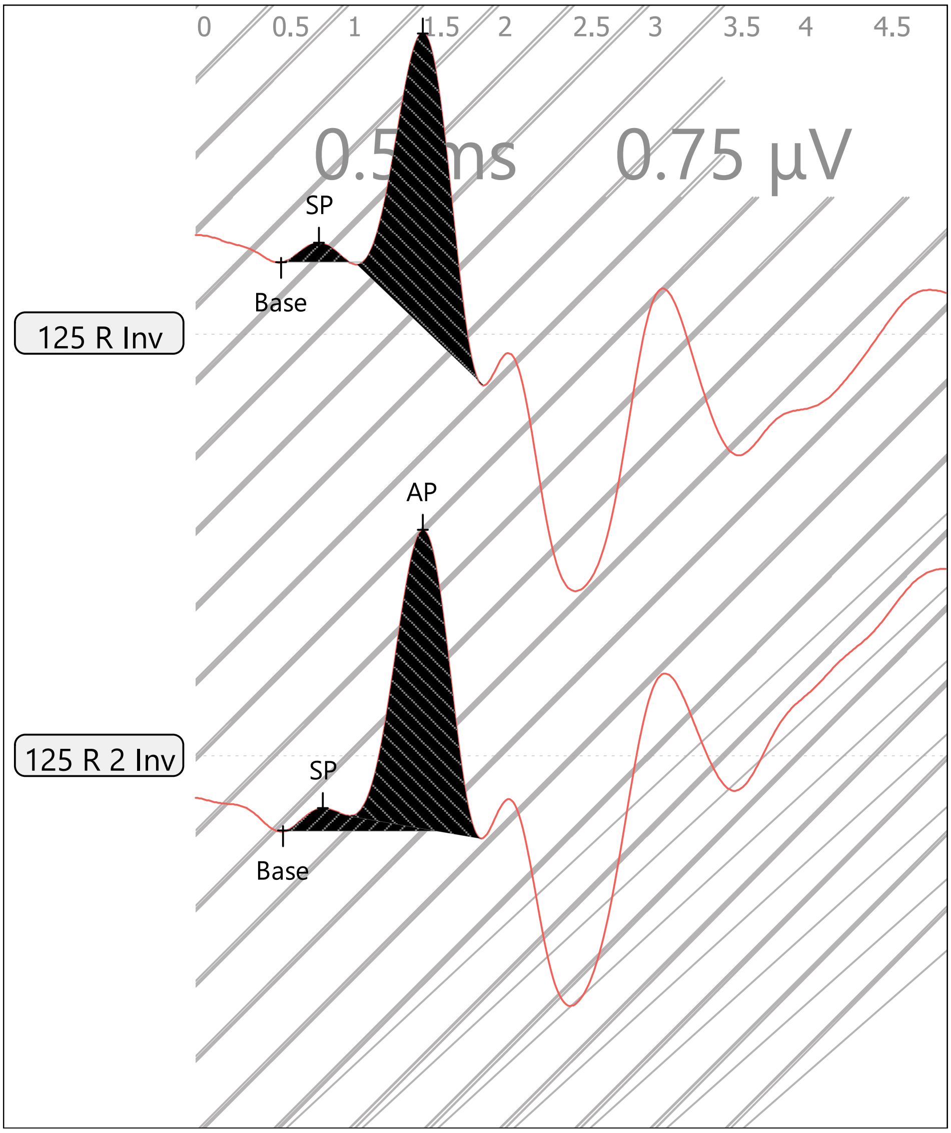

&&

N	Base (ms)	SP (ms)	AP (ms)	SP–Base (ms)	AP–Base (ms)	SP–Base (µV)	AP–Base (µV)	
125 R Inv	0.57	0.82	1.51	0.25	0.94	0.19	2.28	0
125 R 2 Inv	0.58	0.85	1.51	0.26	0.93	0.23	3.00	0

Trace parameters

N	Electr.	HPF, Hz	LPF, Hz	50 Hz	Rejection ±µV	Aver.	R
125 R Inv	Cz-M2	5	2000		50	1500	
125 R 2 Inv	Cz-M2	5	2000		50	1500	

CONCLUSION:

Doctor:
