# Supplementary material for: Exposure to high-altitude hypobaric hypoxic environment induces low-frequency hearing loss in C57BL/6J mice: Mediated by slowing down the postsynaptic electrical signal transmission speed in the cochlear-inferior colliculus auditory signaling pathway
Source: PLoS One. 2026 Mar 11;21(3):e0342321. doi: 10.1371/journal.pone.0342321 (PMC12978441; doi:10.1371/journal.pone.0342321)
Supplement: S1 File — (ZIP) [file pone.0342321.s001.zip › 2025.06.09-3d-4.pdf]

Exam report

Patient: 2025.06.09-3d-4, - (-)
Date: June 10, 2025

ABR: ABR 2 tone burst 8000Hz 2
: Cz-M2

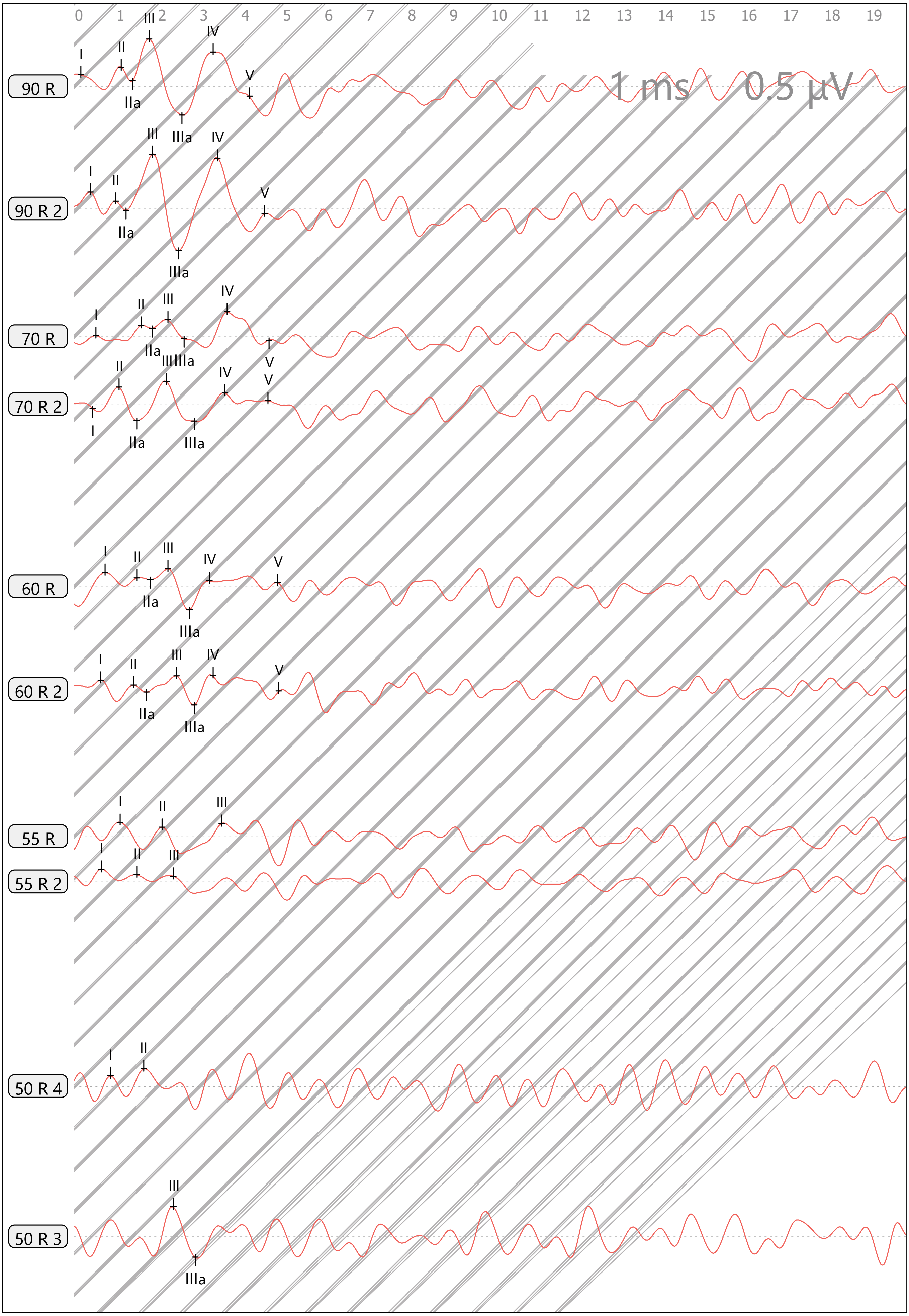

0 1 2 3 4 5 6 7 8 9 10 11 12 13 14 15 16 17 18 19
1 ms
0.5 µV
90 R
90 R 2
70 R
70 R 2
60 R
60 R 2
55 R
55 R 2
50 R 4
50 R 3

IV (ms)	V (ms)	I–V (ms)	I–III (ms)	III–V (ms)
3.33	4.21	4.05	1.64	2.41
3.44	4.58	4.18	1.48	2.70
3.68	4.68	4.15	1.72	2.43
3.62	4.66	4.21	1.77	2.43
3.25	4.89	4.15	1.51	2.65
3.33	4.92	4.29	1.83	2.46
			2.43	
			1.72	

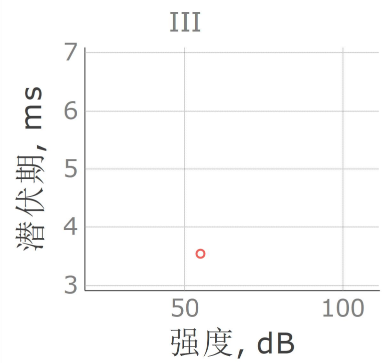

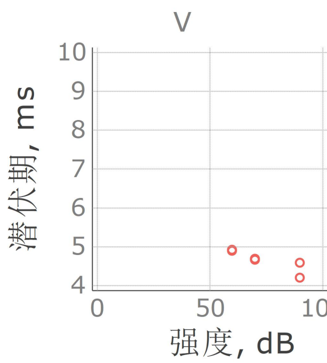

Trace parameters

N	Electr.	HPF, Hz	LPF, Hz	50 Hz	Rejection ±μV	Aver.	Reject
90 R	Cz-M2	200	2000		10	1000	0
90 R 2	Cz-M2	200	2000		10	1000	0
70 R	Cz-M2	200	2000		10	1000	0
70 R 2	Cz-M2	200	2000		10	1000	0
60 R	Cz-M2	200	2000		10	1000	0
60 R 2	Cz-M2	200	2000		10	1000	0
55 R	Cz-M2	200	2000		10	1000	0
55 R 2	Cz-M2	200	2000		10	1000	0
50 R 3	Cz-M2	200	2000		10	1000	0
50 R 4	Cz-M2	200	2000		10	1000	0

ABR: ABR 2 8000Hz 2: Cz-M2

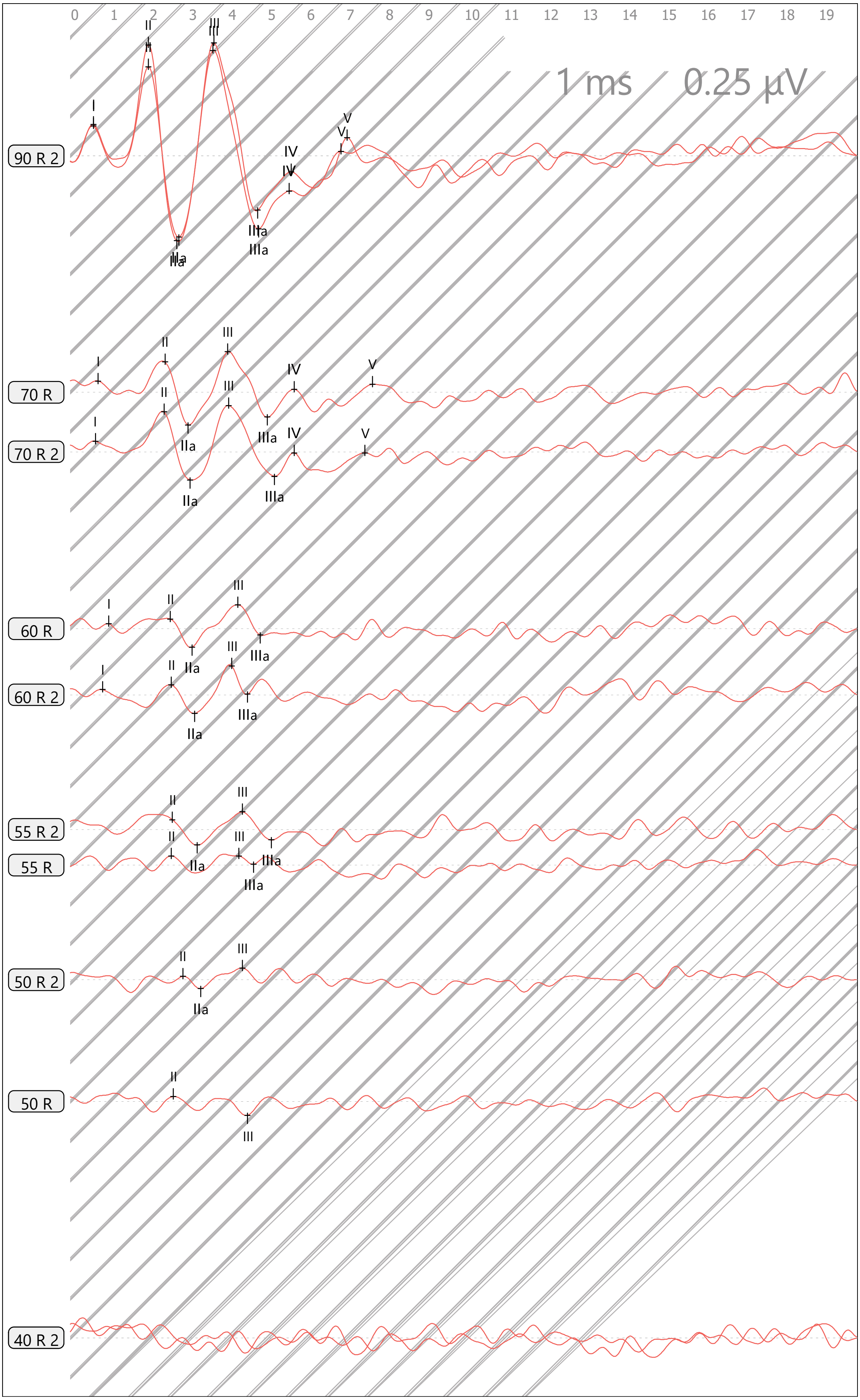
0 1 2 3 4 5 6 7 8 9 10 11 12 13 14 15 16 17 18 19
1 ms
0.25 µV
90 R 2
70 R
70 R 2
60 R
60 R 2
55 R 2
55 R
50 R 2
50 R
40 R 2

IV (ms)	V (ms)	I–V (ms)	I–III (ms)	III–V (ms)	
5.56	7.04	6.46	3.04	3.41	
5.61	6.88	6.30	3.07	3.23	
5.69	7.67	6.96	3.28	3.68	
5.69	7.49	6.85	3.39	3.47	
			3.28		
			3.28		

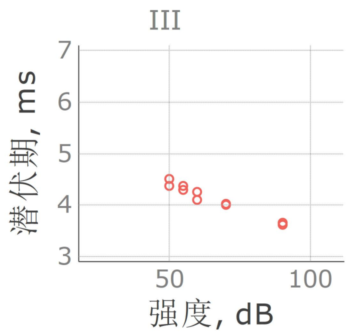

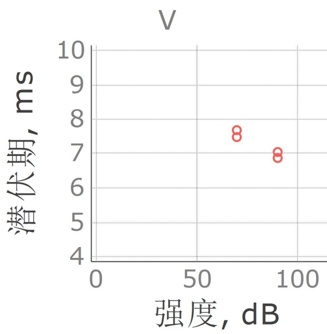

Trace parameters

N	Electr.	HPF, Hz	LPF, Hz	50 Hz	Rejection ±μV	Aver.	Reject
90 R	Cz-M2	200	2000		10	1000	0
90 R 2	Cz-M2	200	2000		10	1000	0
70 R	Cz-M2	200	2000		10	1000	0
70 R 2	Cz-M2	200	2000		10	1000	0
60 R	Cz-M2	200	2000		10	1000	0
60 R 2	Cz-M2	200	2000		10	1000	0
55 R	Cz-M2	200	2000		10	1000	0
55 R 2	Cz-M2	200	2000		10	1000	0
50 R	Cz-M2	200	2000		10	1000	0
50 R 2	Cz-M2	200	2000		10	1000	0
40 R	Cz-M2	200	2000		10	1000	0
40 R 2	Cz-M2	200	2000		10	1000	0

DPOAE: 1-12 kHz 70/70 dB 3 points

Test result (right ear): REFER

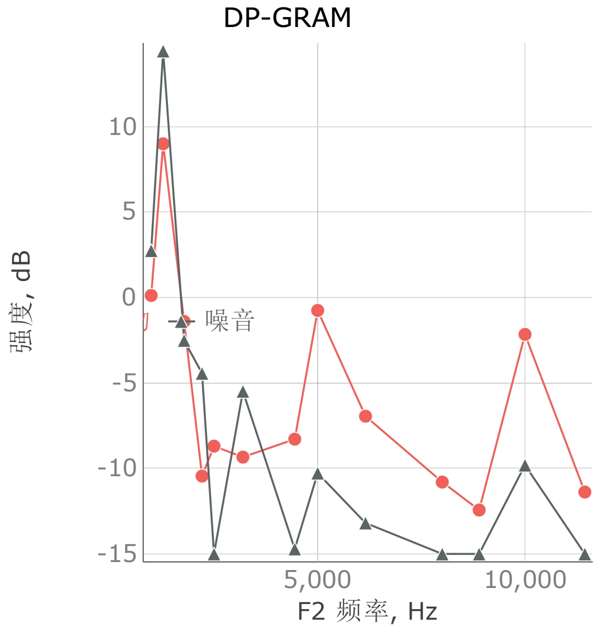

DPOAE

F2, Hz	L1, dB	L2, dB	DP, dB	噪声, dB	SNR, dB	OAE
988	68.2	68.4	0.11	2.77	-2.7	✗
1270	68.8	69.0	8.98	14.42	-5.4	✗
1778	69.6	69.7	-1.42	-2.53	1.1	✗
2222	70.0	70.0	-10.46	-4.45	-6.0	✗
2500	70.2	70.1	-8.69	-15.00	6.3	✓
3200	70.4	70.3	-9.34	-5.50	-3.8	✗
4444	70.8	70.4	-8.31	-14.73	6.4	✓
5000	70.8	70.7	-0.74	-10.28	9.5	✓
6154	71.0	70.2	-6.95	-13.17	6.2	✓
8000	69.9	71.6	-10.81	-15.00	4.2	✗
8889	69.3	72.0	-12.45	-15.00	2.6	✗
10000	71.9	59.9	-2.13	-9.82	7.7	✓
11429	61.3	53.3	-11.37	-15.00	3.6	✗
(dB SPL)	:: 0.0					

EcochG: ECochG 2: Cz-M2

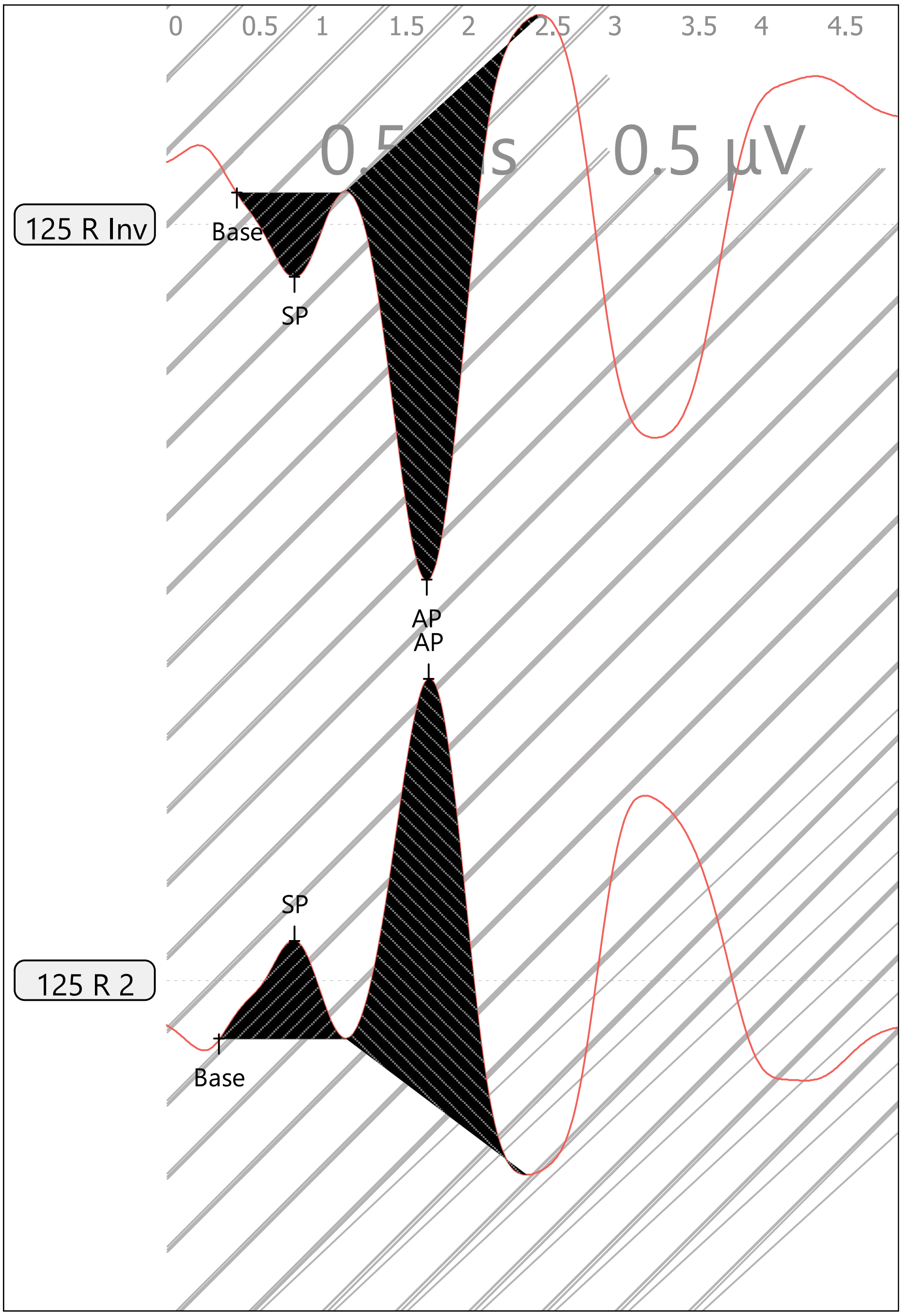

latency&& amplitude

N	Base (ms)	SP (ms)	AP (ms)	SP–Base (ms)	AP–Base (ms)	SP–Base (µV)	AP–Base (µV)	
125 R Inv	0.48	0.87	1.77	0.40	1.30	0.58	2.65	0.2
125 R 2	0.36	0.87	1.79	0.52	1.43	0.67	2.46	0.2

Trace parameters

N	Electr.	HPF, Hz	LPF, Hz	50 Hz	Rejection ±µV	Aver.	Rej
125 R Inv	Cz-M2	5	2000		50	1314	2
125 R 2	Cz-M2	5	2000		50	1094	4

CONCLUSION:

Doctor:
