# Supplementary material for: Exposure to high-altitude hypobaric hypoxic environment induces low-frequency hearing loss in C57BL/6J mice: Mediated by slowing down the postsynaptic electrical signal transmission speed in the cochlear-inferior colliculus auditory signaling pathway
Source: PLoS One. 2026 Mar 11;21(3):e0342321. doi: 10.1371/journal.pone.0342321 (PMC12978441; doi:10.1371/journal.pone.0342321)
Supplement: S1 File — (ZIP) [file pone.0342321.s001.zip › 2025.06.10-5d-2.pdf]

Exam report

Patient: 2025.06.10-5d-2, - (-)
Date: June 10, 2025

ABR: ABR 2 CLICK
1: Cz-M1

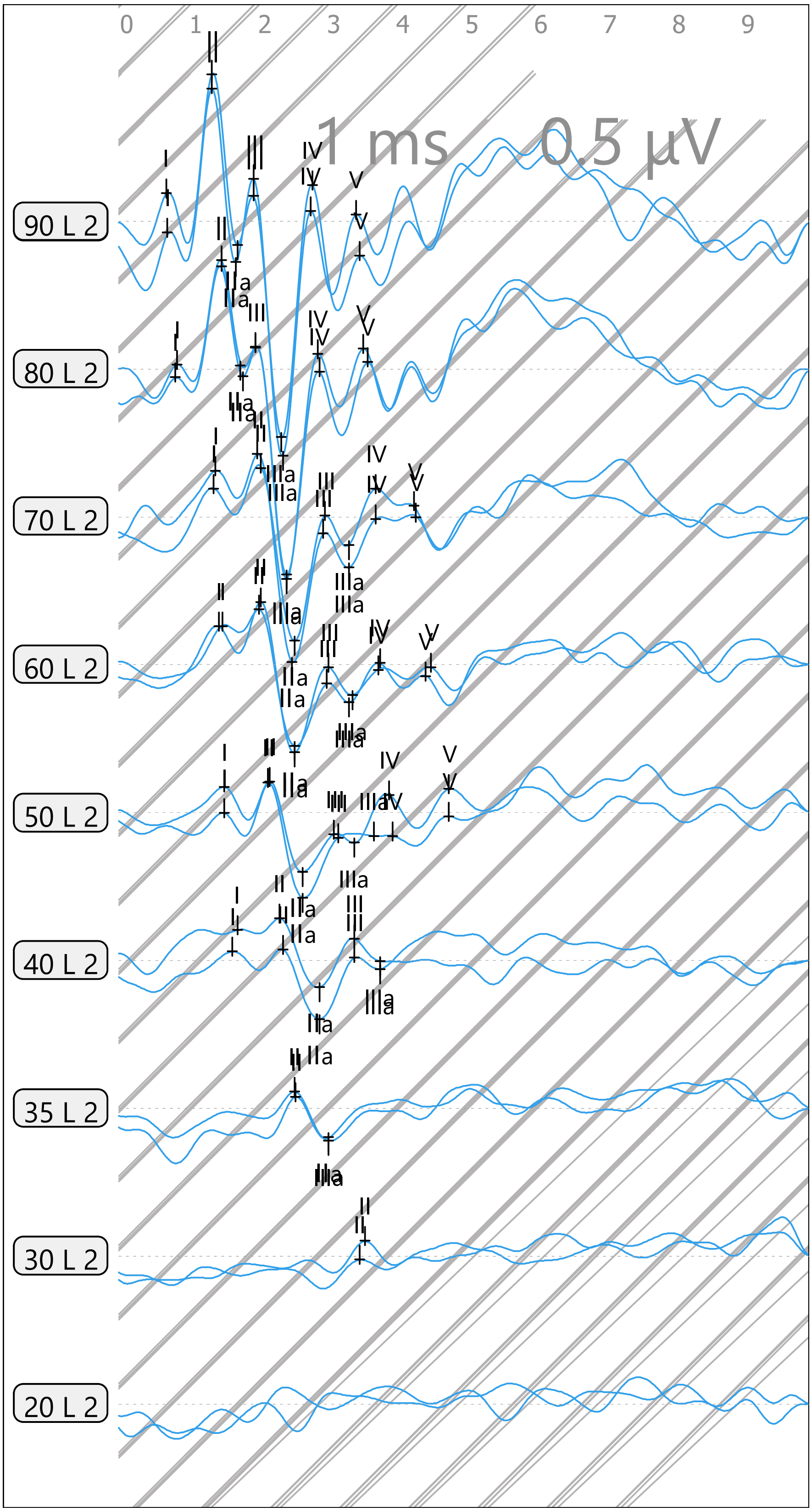

latency && amplitude) (left ear

N	I (ms)	II (ms)	III (ms)	IV (ms)	V (ms)
90 L	0.71	1.35	1.96	2.78	3.44
90 L 2	0.69	1.35	1.96	2.80	3.49
80 L	0.82	1.48	1.98	2.88	3.55
80 L 2	0.85	1.48	1.98	2.91	3.60
70 L	1.38	2.06	2.99	3.73	4.29
70 L 2	1.40	2.01	2.96	3.73	4.31
60 L	1.46	2.06	3.04	3.76	4.52
60 L 2	1.51	2.04	3.02	3.78	4.45
50 L	1.53	2.17	3.18	3.97	4.79
50 L 2	1.53	2.20	3.12	3.92	4.79
40 L	1.72	2.33	3.41		
40 L 2	1.64	2.38	3.41		
35 L		2.57			
35 L 2		2.54			
30 L		3.49			
30 L 2		3.57			

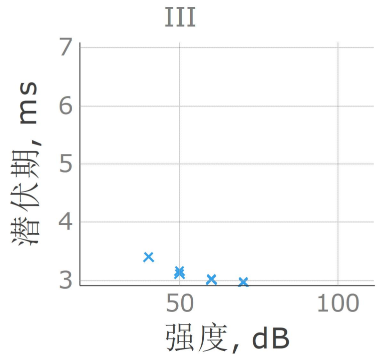

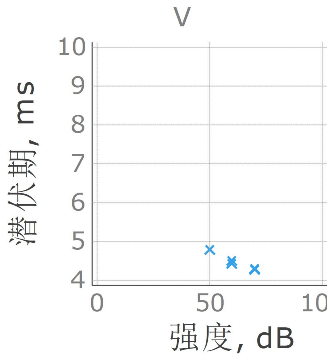

Trace parameters

N	Electr.	HPF, Hz	LPF, Hz	50 Hz	Rejection ±μV	Aver.	Reject.
90 L	Cz-M1	100	2000		10	1000	0
90 L 2	Cz-M1	100	2000		10	1000	0
80 L	Cz-M1	100	2000		10	1000	0
80 L 2	Cz-M1	100	2000		10	1000	0
70 L	Cz-M1	100	2000		10	1000	0
70 L 2	Cz-M1	100	2000		10	1000	0
60 L	Cz-M1	100	2000		10	1000	0
60 L 2	Cz-M1	100	2000		10	1000	0
50 L	Cz-M1	100	2000		10	1000	0
50 L 2	Cz-M1	100	2000		10	1000	0
40 L	Cz-M1	100	2000		10	1000	0
40 L 2	Cz-M1	100	2000		10	1000	0
35 L	Cz-M1	100	2000		10	1000	0
35 L 2	Cz-M1	100	2000		10	1000	0
30 L	Cz-M1	100	2000		10	1000	0
30 L 2	Cz-M1	100	2000		10	1000	0
20 L	Cz-M1	100	2000		10	1000	0
20 L 2	Cz-M1	100	2000		10	1000	0

ABR: ABR 2 tone burst 4000Hz 1
: Cz-M1

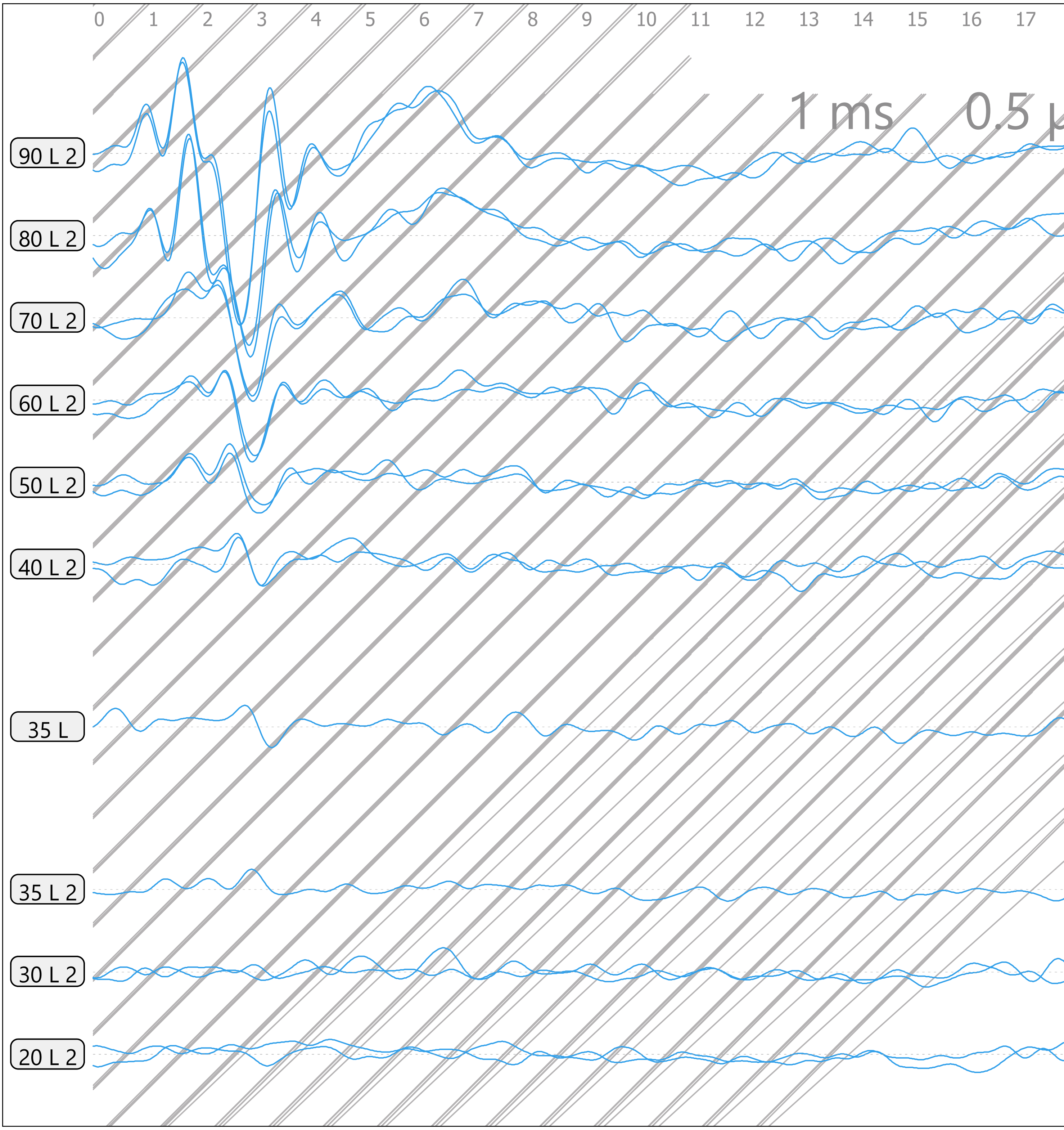

Trace parameters

N	Electr.	HPF, Hz	LPF, Hz		Rejection ±μV	Aver.	Reject.
90 L	Cz-M1	200	2000		10	1000	0
90 L 2	Cz-M1	200	2000		10	1000	0
80 L	Cz-M1	200	2000		10	1000	0
80 L 2	Cz-M1	200	2000		10	1000	0
70 L	Cz-M1	200	2000		10	1000	0
70 L 2	Cz-M1	200	2000		10	1000	0

60 L	Cz-M1	200	2000		10	1000	0
60 L 2	Cz-M1	200	2000		10	1000	0
50 L	Cz-M1	200	2000		10	1000	0
50 L 2	Cz-M1	200	2000		10	1000	0
40 L	Cz-M1	200	2000		10	1000	0
40 L 2	Cz-M1	200	2000		10	1000	0
35 L	Cz-M1	200	2000		10	1000	0
35 L 2	Cz-M1	200	2000		10	1000	0
30 L	Cz-M1	200	2000		10	1000	0
30 L 2	Cz-M1	200	2000		10	1000	0
20 L	Cz-M1	200	2000		10	1000	0
20 L 2	Cz-M1	200	2000		10	1000	0

ABR: ABR 2 8000Hz 1: Cz-M1

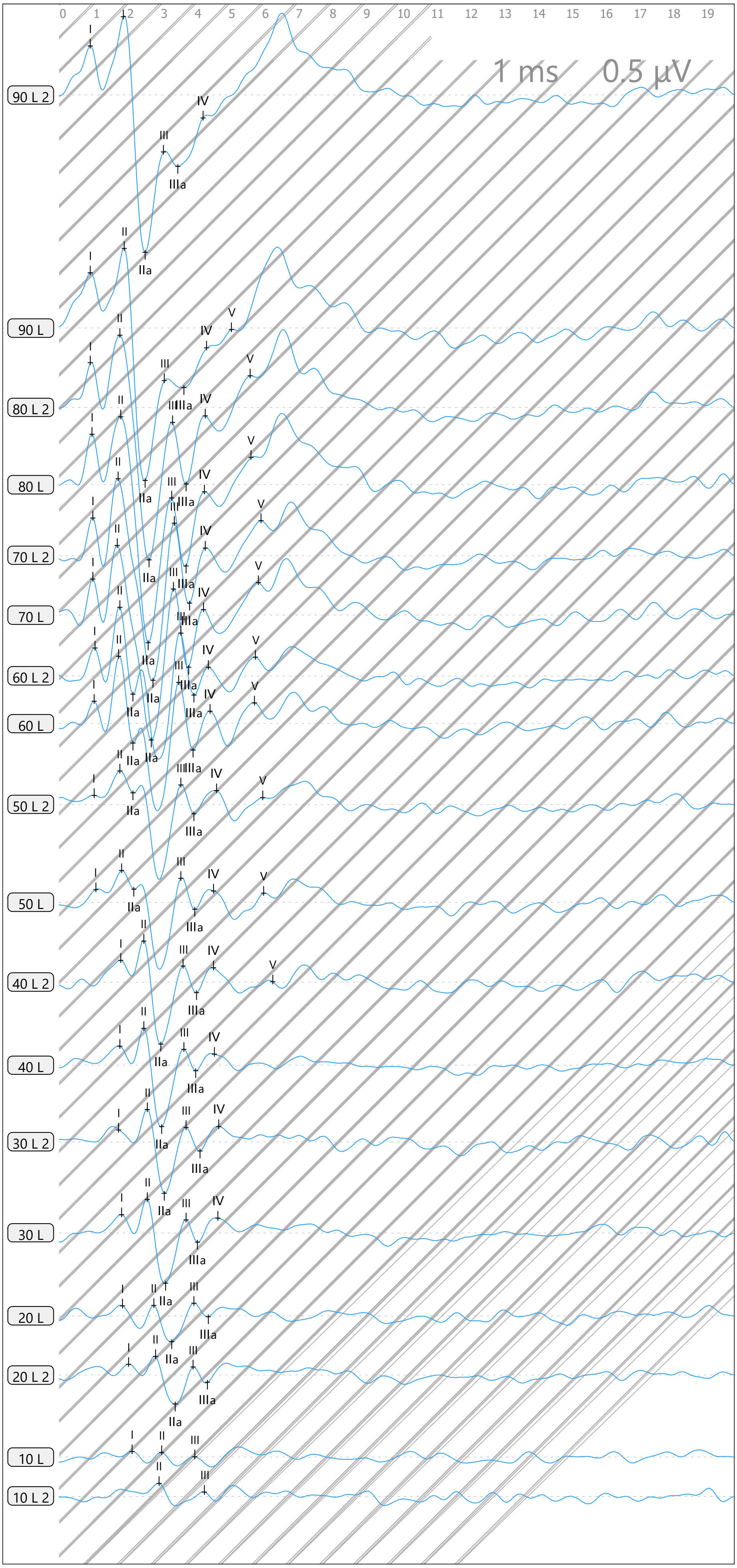
0
1
2
3
4
5
6
7
8
9
10
11
12
13
14
15
16
17
18
19
1 ms
0.5 µV
90 L 2
90 L
80 L 2
80 L
70 L 2
70 L
60 L 2
60 L
50 L 2
50 L
40 L 2
40 L
30 L 2
30 L
20 L
20 L 2
10 L
10 L 2
I
II
IIa
III
IIIa
IV
V

&& (left ear

N	I (ms)	II (ms)	III (ms)	IV (ms)	V (ms)
90 L	0.93	1.93	3.12	4.37	5.11
90 L 2	0.93	1.91	3.10	4.26	
80 L	0.98	1.83	3.33	4.31	5.69
80 L 2	0.93	1.80	3.36	4.34	5.66
70 L	1.01	1.72	3.39	4.29	5.90
70 L 2	1.01	1.75	3.41	4.34	5.98
60 L	1.03	1.77	3.55	4.47	5.79
60 L 2	1.06	1.80	3.60	4.42	5.82
50 L	1.08	1.85	3.60	4.58	6.06
50 L 2	1.03	1.80	3.60	4.66	6.03
40 L	1.80	2.51	3.70	4.60	
40 L 2	1.83	2.51	3.68	4.58	6.32
30 L	1.85	2.62	3.76	4.71	
30 L 2	1.77	2.62	3.76	4.74	
20 L	1.88	2.80	4.00		
20 L 2	2.06	2.86	3.97		
10 L	2.17	3.04	4.02		
10 L 2		2.96	4.31		

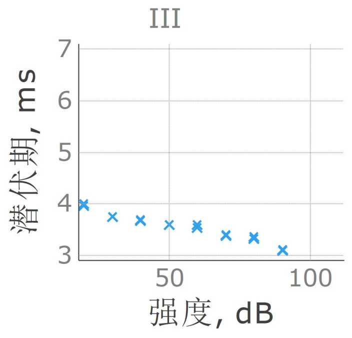

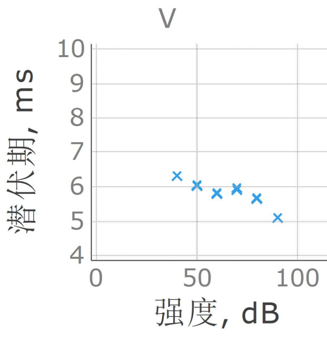

Trace parameters

N	Electr.	HPF, Hz	LPF, Hz	50 Hz	Rejection ±μV	Aver.	Reject.
90 L	Cz-M1	200	2000		10	1000	0
90 L 2	Cz-M1	200	2000		10	1000	0
80 L	Cz-M1	200	2000		10	1000	0
80 L 2	Cz-M1	200	2000		10	1000	0
70 L	Cz-M1	200	2000		10	1000	0
70 L 2	Cz-M1	200	2000		10	1000	0
60 L	Cz-M1	200	2000		10	1000	0
60 L 2	Cz-M1	200	2000		10	1000	0
50 L	Cz-M1	200	2000		10	1000	0
50 L 2	Cz-M1	200	2000		10	1000	0
40 L	Cz-M1	200	2000		10	1000	0
40 L 2	Cz-M1	200	2000		10	1000	0
30 L	Cz-M1	200	2000		10	1000	0

30 L 2	Cz-M1	200	2000		10	1000	0
20 L	Cz-M1	200	2000		10	1000	0
20 L 2	Cz-M1	200	2000		10	1000	0
10 L	Cz-M1	200	2000		10	1000	0
10 L 2	Cz-M1	200	2000		10	1000	0

ABR: ABR 2 CLICK 2: Cz-M2

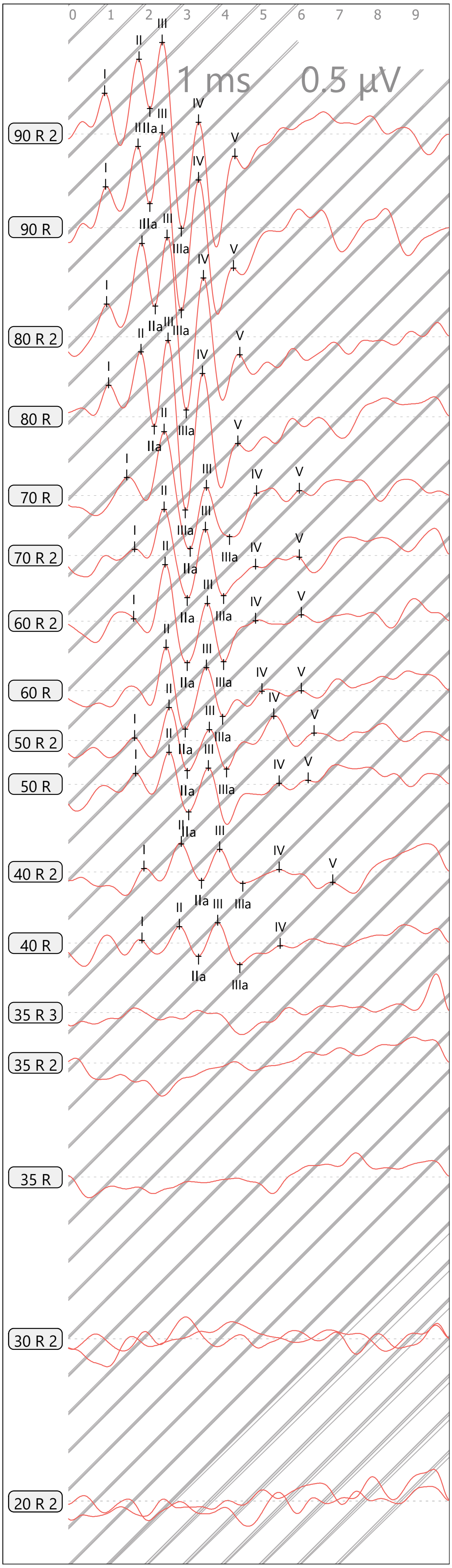
0 1 2 3 4 5 6 7 8 9
1 ms
0.5 µV
90 R 2
90 R
80 R 2
80 R
70 R
70 R 2
60 R 2
60 R
50 R 2
50 R
40 R 2
40 R
35 R 3
35 R 2
35 R
30 R 2
20 R 2

II ms)	IV (ms)	V (ms)	I–III (ms)	I–V (ms)	II (r
	3.41	4.34	1.48	3.36	1.88
	3.41	4.37	1.51	3.41	1.91
	3.52	4.45	1.56	3.39	1.83
	3.55	4.50	1.59	3.49	1.91
	4.95	6.06	2.09	4.52	2.43
	4.92	6.06	1.85	4.31	2.46
	5.08	6.11			2.49
	4.92	6.11	1.93	4.39	2.46
	5.53	6.30	1.91	4.52	2.62
	5.40	6.46	1.96	4.71	2.75
	5.56		1.98		
	5.53	6.93	1.98	4.95	2.96

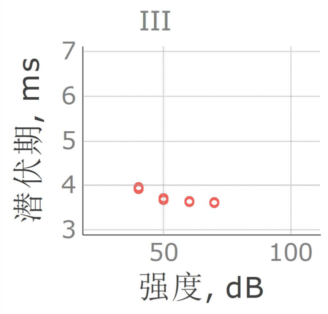

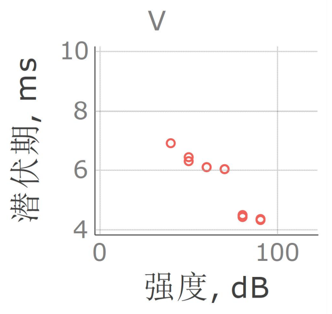

Trace parameters

N	Electr.	HPF, Hz	LPF, Hz	50 Hz	Rejection ±μV	Aver.	Reject
90 R	Cz-M2	100	2000		10	1000	0
90 R 2	Cz-M2	100	2000		10	1000	0
80 R	Cz-M2	100	2000		10	1000	0
80 R 2	Cz-M2	100	2000		10	1000	0
70 R	Cz-M2	100	2000		10	1000	0
70 R 2	Cz-M2	100	2000		10	1000	0
60 R	Cz-M2	100	2000		10	1000	0
60 R 2	Cz-M2	100	2000		10	1000	0
50 R	Cz-M2	100	2000		10	1000	0
50 R 2	Cz-M2	100	2000		10	1000	0
40 R	Cz-M2	100	2000		10	1000	0
40 R 2	Cz-M2	100	2000		10	1000	0
35 R	Cz-M2	100	2000		10	1000	0
35 R 2	Cz-M2	100	2000		10	1000	0
35 R 3	Cz-M2	100	2000		10	1000	0
30 R	Cz-M2	100	2000		10	1000	0
30 R 2	Cz-M2	100	2000		10	1000	0
20 R	Cz-M2	100	2000		10	1000	0
20 R 2	Cz-M2	100	2000		10	1000	0

ABR: ABR 2 4000Hz 2: Cz-M2

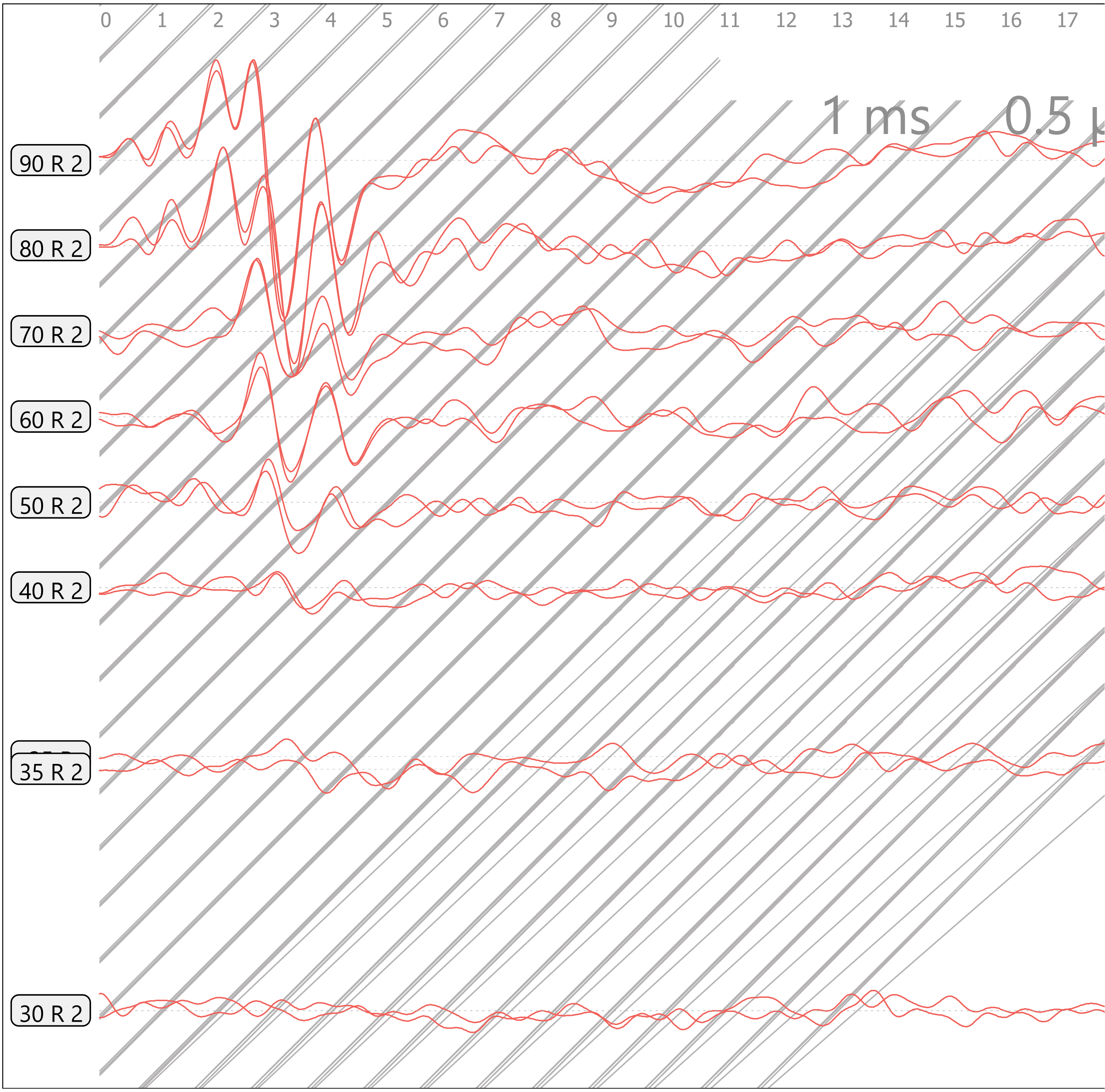

Trace parameters

N	Electr.	HPF, Hz	LPF, Hz	50 Hz	Rejection ±μV	Aver.	Reject
90 R	Cz-M2	200	2000		10	1000	0
90 R 2	Cz-M2	200	2000		10	1000	0
80 R	Cz-M2	200	2000		10	1000	0
80 R 2	Cz-M2	200	2000		10	1000	0
70 R	Cz-M2	200	2000		10	1000	0
70 R 2	Cz-M2	200	2000		10	1000	0
60 R	Cz-M2	200	2000		10	1000	0

60 R 2	Cz-M2	200	2000		10	1000	0
50 R	Cz-M2	200	2000		10	1000	0
50 R 2	Cz-M2	200	2000		10	1000	0
40 R	Cz-M2	200	2000		10	1000	0
40 R 2	Cz-M2	200	2000		10	1000	0
35 R	Cz-M2	200	2000		10	1000	0
35 R 2	Cz-M2	200	2000		10	1000	0
30 R	Cz-M2	200	2000		10	1000	0
30 R 2	Cz-M2	200	2000		10	1000	0

ABR: ABR 2 8000Hz 2: Cz-M2

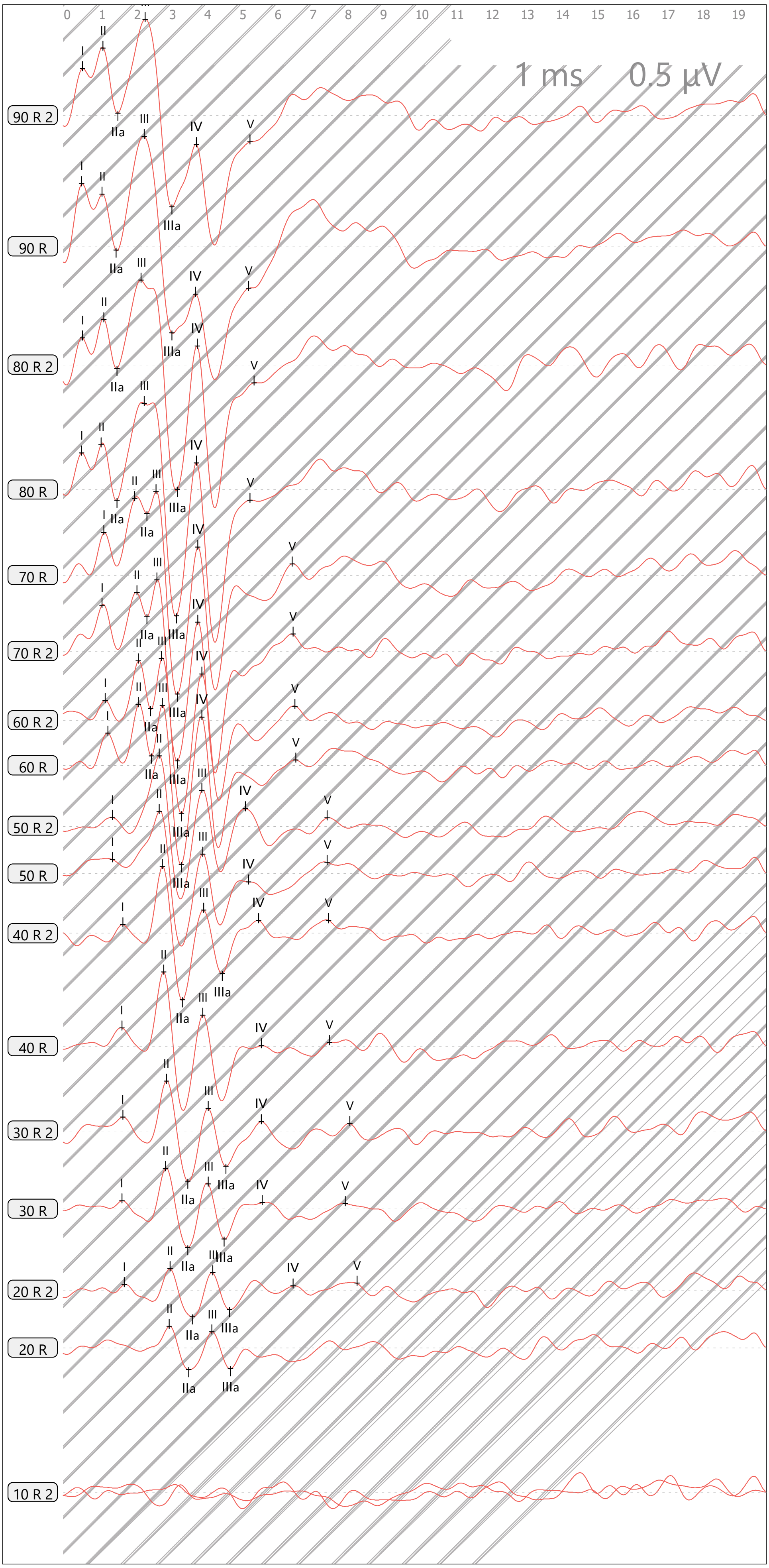
0 1 2 3 4 5 6 7 8 9 10 11 12 13 14 15 16 17 18 19
1 ms
0.5 µV
90 R 2
90 R
80 R 2
80 R
70 R
70 R 2
60 R 2
60 R
50 R 2
50 R
40 R 2
40 R
30 R 2
30 R
20 R 2
20 R
10 R 2
I
II
IIa
III
IIIa
IV
V

IV (ms)	V (ms)	I–III (ms)	I–V (ms)	III–V (ms)	
3.76	5.27	1.77	4.74	2.96	
3.78	5.32	1.77	4.76	2.99	
3.78	5.32	1.77	4.79	3.02	
3.81	5.42	1.67	4.87	3.20	
3.84	6.51	1.48	5.34	3.86	
3.84	6.54	1.56	5.42	3.86	
3.94	6.61	1.56	5.34	3.78	
3.94	6.59	1.61	5.40	3.78	
5.27	7.51	2.57	6.11	3.55	
5.19	7.51	2.54	6.11	3.57	
5.64	7.57	2.30	5.90	3.60	
5.56	7.54	2.30	5.85	3.55	
5.66	8.02	2.46	6.35	3.89	
5.64	8.15	2.43	6.46	4.02	
6.54	8.36	2.51	6.61	4.10	

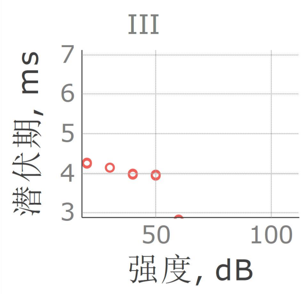

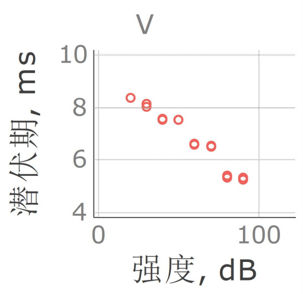

Trace parameters

N	Electr.	HPF, Hz	LPF, Hz	50 Hz	Rejection ±μV	Aver.	Reject
90 R	Cz-M2	200	2000		10	1000	0
90 R 2	Cz-M2	200	2000		10	1000	0
80 R	Cz-M2	200	2000		10	1000	0
80 R 2	Cz-M2	200	2000		10	1000	0
70 R	Cz-M2	200	2000		10	1000	0
70 R 2	Cz-M2	200	2000		10	1000	0
60 R	Cz-M2	200	2000		10	1000	0
60 R 2	Cz-M2	200	2000		10	1000	0
50 R	Cz-M2	200	2000		10	1000	0
50 R 2	Cz-M2	200	2000		10	1000	0
40 R	Cz-M2	200	2000		10	1000	0
40 R 2	Cz-M2	200	2000		10	1000	0
30 R	Cz-M2	200	2000		10	1000	0
30 R 2	Cz-M2	200	2000		10	1000	0
20 R	Cz-M2	200	2000		10	1000	0

20 R 2	Cz-M2	200	2000		10	1000	0
10 R	Cz-M2	200	2000		10	1000	0
10 R 2	Cz-M2	200	2000		10	1000	0

ECochG: ECochG 2: Cz-M2

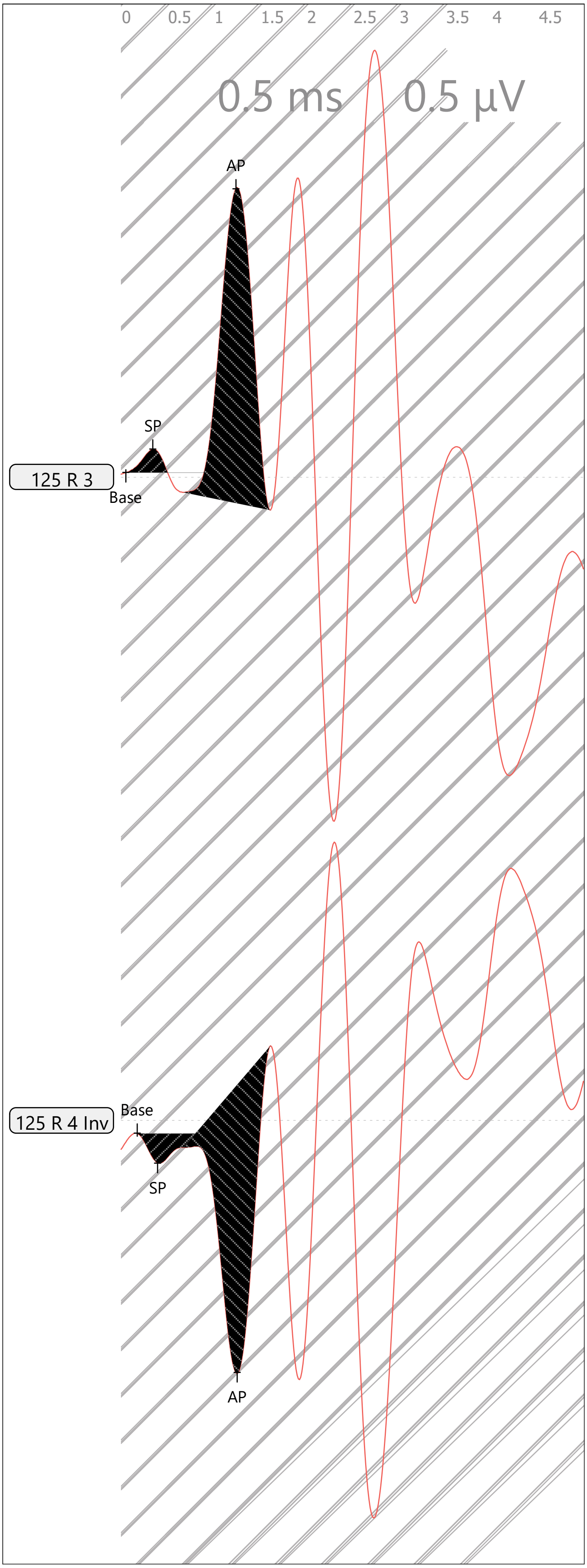

0
0.5
1
1.5
2
2.5
3
3.5
4
4.5
0.5 ms
0.5 µV
AP
SP
125 R 3
Base
Base
125 R 4 Inv
SP
AP

&&

N	Base (ms)	SP (ms)	AP (ms)	SP–Base (ms)	AP–Base (ms)	SP–Base (µV)	AP–Base (µV)	
125 R 3	0.05	0.34	1.24	0.29	1.19	0.25	3.06	0
125 R 4 Inv	0.17	0.40	1.26	0.22	1.08	0.32	2.58	0

Trace parameters

N	Electr.	HPF, Hz	LPF, Hz	50 Hz	Rejection ±µV	Aver.	R
125 R 3	Cz-M2	5	2000		50	1500	
125 R 4 Inv	Cz-M2	5	2000		50	1500	

ECochG: ECochG 1:
Fpz-M1

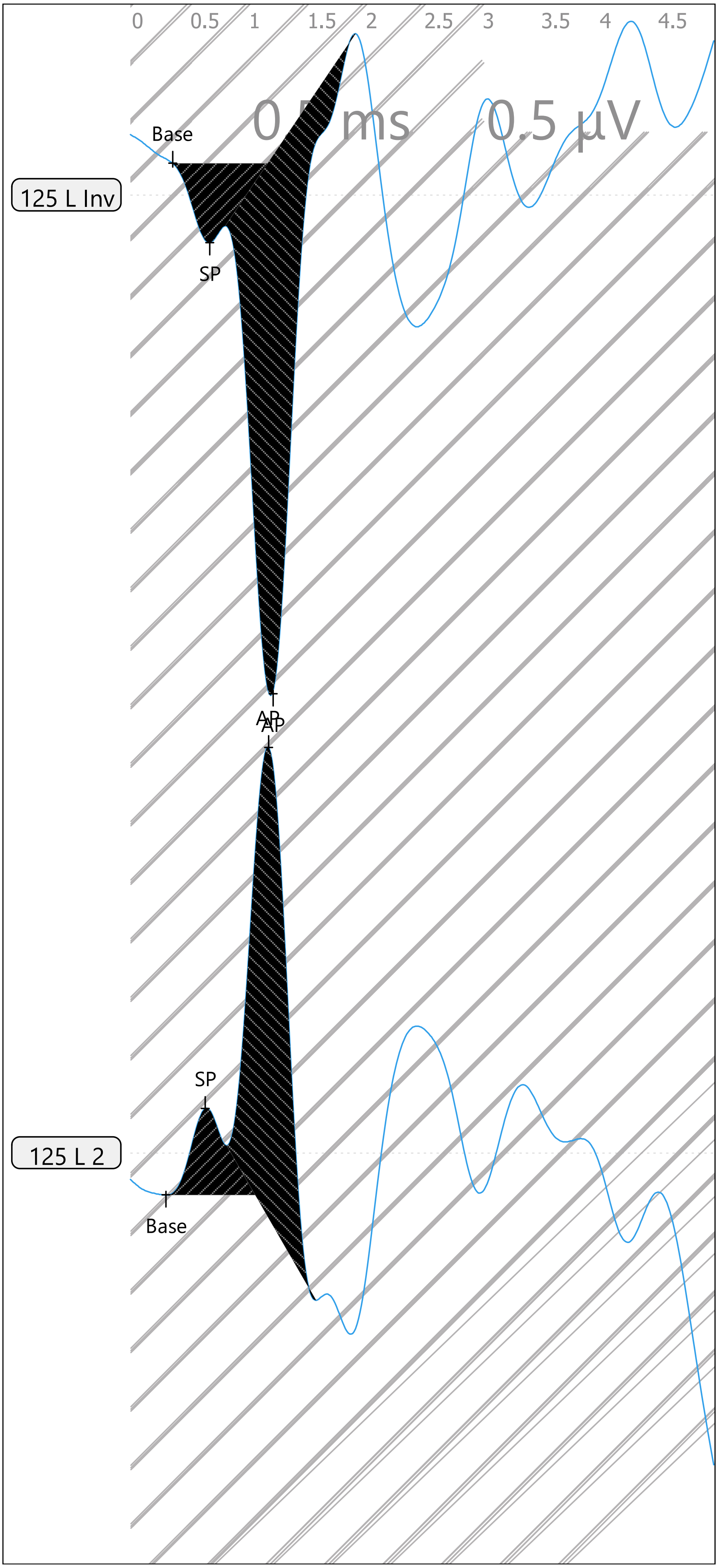
0
0.5
1
1.5
2
2.5
3
3.5
4
4.5
0.5 ms
0.5 µV
Base
125 L Inv
SP
AP
AP
SP
125 L 2
Base

&&

N	Base (ms)	SP (ms)	AP (ms)	SP–Base (ms)	AP–Base (ms)	SP–Base (µV)	AP–Base (µV)	
125 L Inv	0.36	0.67	1.22	0.32	0.86	0.68	4.53	0.15
125 L 2	0.30	0.64	1.18	0.33	0.87	0.74	3.83	0.19

Trace parameters

N	Electr.	HPF, Hz	LPF, Hz	50 Hz	Rejection ±µV	Aver.	Rej[illegible]
125 L Inv	Fpz-M1	5	2000		50	1500	4[illegible]
125 L 2	Fpz-M1	5	2000		50	1500	1[illegible]

CONCLUSION:

Doctor:
