# Supplementary material for: Exposure to high-altitude hypobaric hypoxic environment induces low-frequency hearing loss in C57BL/6J mice: Mediated by slowing down the postsynaptic electrical signal transmission speed in the cochlear-inferior colliculus auditory signaling pathway
Source: PLoS One. 2026 Mar 11;21(3):e0342321. doi: 10.1371/journal.pone.0342321 (PMC12978441; doi:10.1371/journal.pone.0342321)
Supplement: S1 File — (ZIP) [file pone.0342321.s001.zip › 2025.06.10-5d-3.pdf]

# Exam report

**Patient:** 2025.06.10-5d-3**,** - ( - )
**Date:** June 10, 2025

**ABR**: ABR 2 CLICK
1: Cz-M1

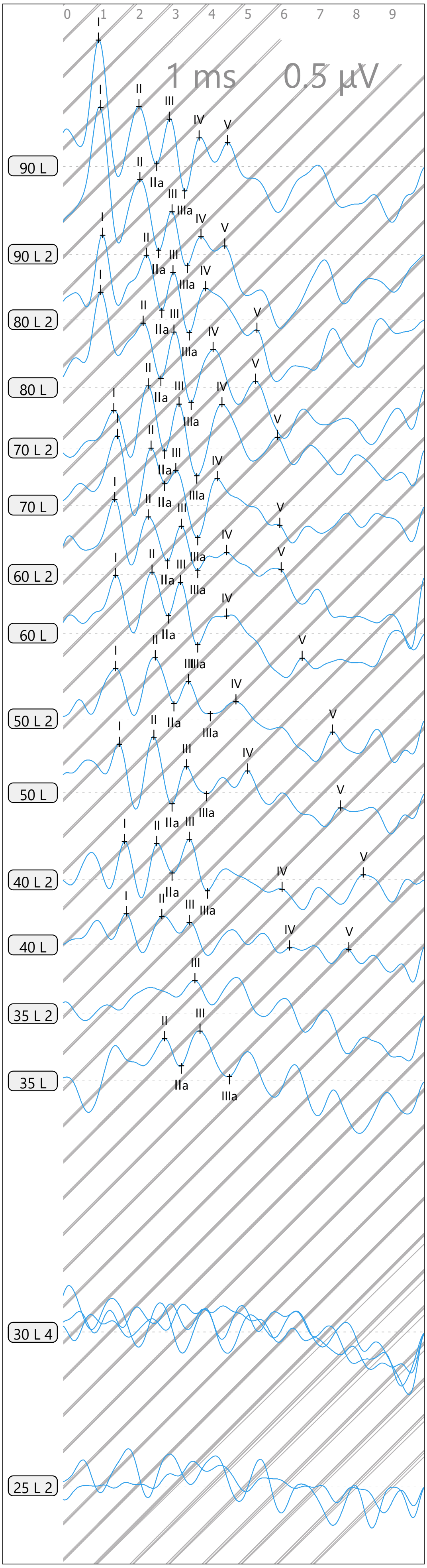

0 1 2 3 4 5 6 7 8 9
1 ms
0.5 µV
90 L
90 L 2
80 L 2
80 L
70 L 2
70 L
60 L 2
60 L
50 L 2
50 L
40 L 2
40 L
35 L 2
35 L
30 L 4
25 L 2

latency&& amplitude (left ear

| N | I (ms) | II (ms) | III (ms) | IV (ms) | V (ms) |
|---|---|---|---|---|---|
| 90 L | 0.98 | 2.09 | 2.94 | 3.76 | 4.55 |
| 90 L 2 | 1.03 | 2.12 | 3.02 | 3.81 | 4.47 |
| 80 L | 1.03 | 2.22 | 3.07 | 4.15 | 5.32 |
| 80 L 2 | 1.08 | 2.30 | 3.04 | 3.94 | 5.37 |
| 70 L | 1.51 | 2.43 | 3.12 | 4.26 | 6.01 |
| 70 L 2 | 1.40 | 2.35 | 3.20 | 4.39 | 5.93 |
| 60 L | 1.46 | 2.46 | 3.25 | 4.52 | 6.61 |
| 60 L 2 | 1.43 | 2.35 | 3.28 | 4.52 | 6.03 |
| 50 L | 1.56 | 2.51 | 3.41 | 5.11 | 7.67 |
| 50 L 2 | 1.46 | 2.54 | 3.47 | 4.79 | 7.46 |
| 40 L | 1.75 | 2.73 | 3.49 | 6.27 | 7.91 |
| 40 L 2 | 1.69 | 2.59 | 3.49 | 6.06 | 8.31 |
| 35 L | | 2.80 | 3.78 | | |
| 35 L 2 | | | 3.65 | | |

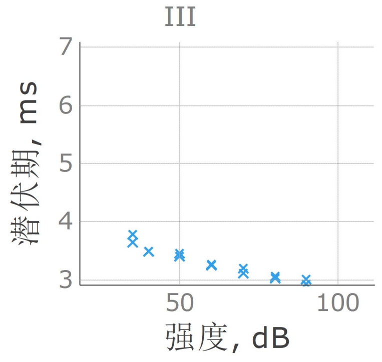


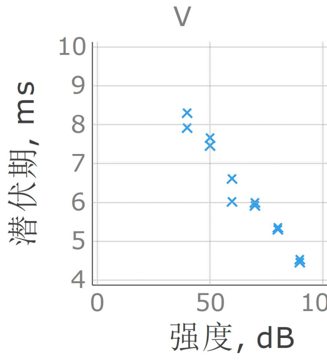


Trace parameters

| N | Electr. | HPF, Hz | LPF, Hz | 50 Hz | Rejection ±μV | Aver. | Reject. |
|---|---|---|---|---|---|---|---|
| 90 L | Cz-M1 | 100 | 2000 | turn on | 10 | 1000 | 0 |
| 90 L 2 | Cz-M1 | 100 | 2000 | | 10 | 1000 | 0 |
| 80 L | Cz-M1 | 100 | 2000 | | 10 | 1000 | 0 |
| 80 L 2 | Cz-M1 | 100 | 2000 | | 10 | 1000 | 0 |
| 70 L | Cz-M1 | 100 | 2000 | | 10 | 1000 | 0 |
| 70 L 2 | Cz-M1 | 100 | 2000 | | 10 | 1000 | 0 |
| 60 L | Cz-M1 | 100 | 2000 | | 10 | 1000 | 0 |
| 60 L 2 | Cz-M1 | 100 | 2000 | | 10 | 1000 | 0 |
| 50 L | Cz-M1 | 100 | 2000 | | 10 | 1000 | 0 |
| 50 L 2 | Cz-M1 | 100 | 2000 | | 10 | 1000 | 0 |
| 40 L | Cz-M1 | 100 | 2000 | | 10 | 1000 | 0 |
| 40 L 2 | Cz-M1 | 100 | 2000 | | 10 | 1000 | 0 |
| 35 L | Cz-M1 | 100 | 2000 | | 10 | 1000 | 0 |
| 35 L 2 | Cz-M1 | 100 | 2000 | | 10 | 1000 | 0 |
| 30 L 2 | Cz-M1 | 100 | 2000 | | 10 | 1000 | 0 |
| 30 L 3 | Cz-M1 | 100 | 2000 | | 10 | 1000 | 0 |
| 30 L 4 | Cz-M1 | 100 | 2000 | | 10 | 1000 | 0 |
| 25 L | Cz-M1 | 100 | 2000 | | 10 | 1000 | 0 |
| 25 L 2 | Cz-M1 | 100 | 2000 | | 10 | 1000 | 0 |

**ABR**: ABR 2 2000Hz 1: Cz-M1

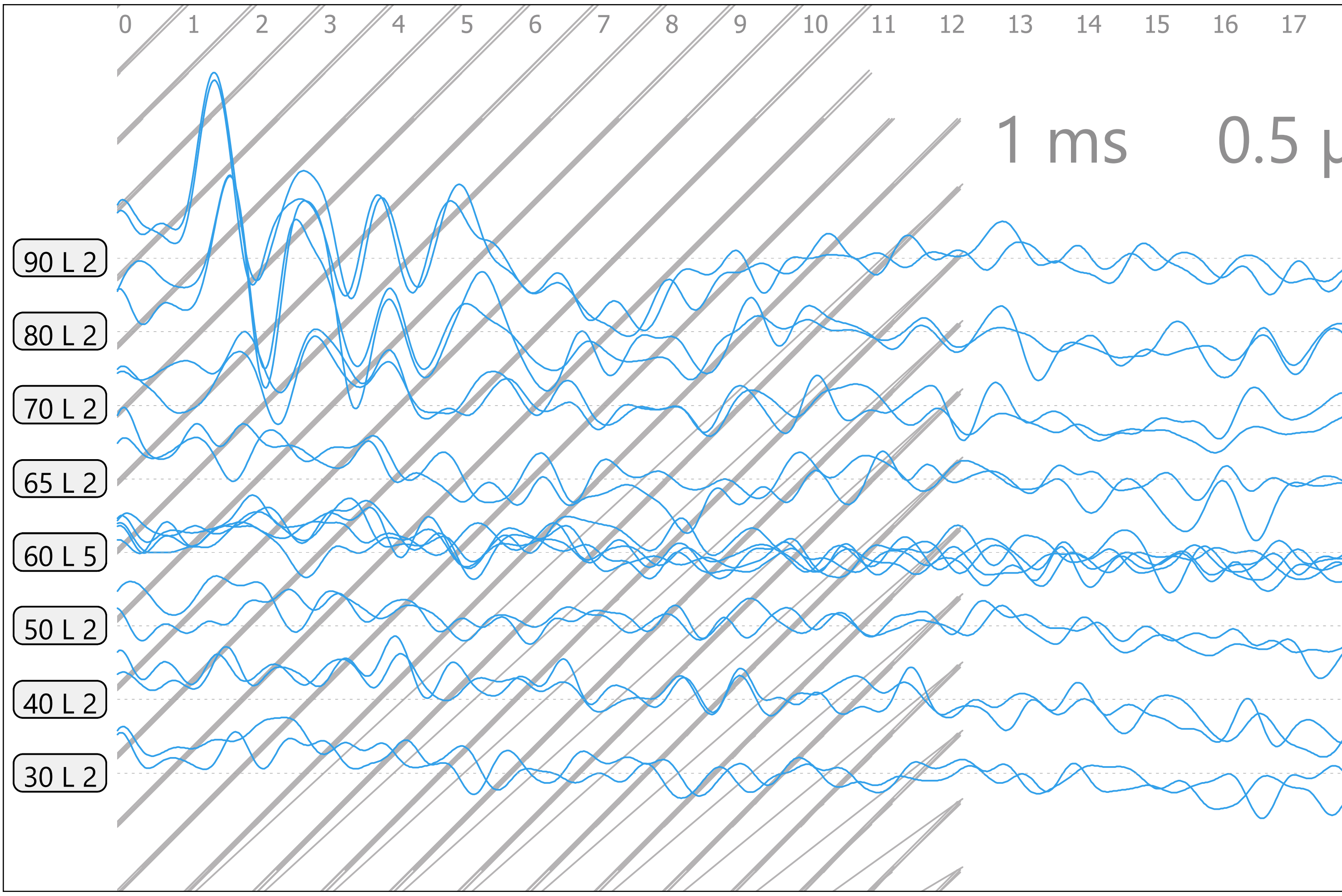


Trace parameters

| N | Electr. | HPF, Hz | LPF, Hz | 50 Hz | Rejection ±μV | Aver. | Reject. |
|---|---|---|---|---|---|---|---|
| 90 L | Cz-M1 | 200 | 2000 | | 10 | 1000 | 0 |
| 90 L 2 | Cz-M1 | 200 | 2000 | | 10 | 1000 | 0 |
| 80 L | Cz-M1 | 200 | 2000 | | 10 | 1000 | 0 |
| 80 L 2 | Cz-M1 | 200 | 2000 | | 10 | 1000 | 0 |
| 70 L | Cz-M1 | 200 | 2000 | | 10 | 1000 | 0 |
| 70 L 2 | Cz-M1 | 200 | 2000 | | 10 | 1000 | 0 |
| 65 L | Cz-M1 | 200 | 2000 | | 10 | 1000 | 0 |
| 65 L 2 | Cz-M1 | 200 | 2000 | | 10 | 1000 | 0 |
| 60 L | Cz-M1 | 200 | 2000 | | 10 | 1000 | 0 |
| 60 L 2 | Cz-M1 | 200 | 2000 | | 10 | 1000 | 0 |
| 60 L 3 | Cz-M1 | 200 | 2000 | | 10 | 1000 | 0 |
| 60 L 4 | Cz-M1 | 200 | 2000 | | 10 | 1000 | 0 |
| 60 L 5 | Cz-M1 | 200 | 2000 | | 10 | 1000 | 0 |
| 50 L | Cz-M1 | 200 | 2000 | | 10 | 1000 | 0 |
| 50 L 2 | Cz-M1 | 200 | 2000 | | 10 | 1000 | 0 |
| 40 L | Cz-M1 | 200 | 2000 | | 10 | 1000 | 0 |

| | | | | | | | |
|---|---|---|---|---|---|---|---|
| 40 L 2 | Cz-M1 | 200 | 2000 | | 10 | 1000 | 0 |
| 30 L | Cz-M1 | 200 | 2000 | | 10 | 1000 | 0 |
| 30 L 2 | Cz-M1 | 200 | 2000 | | 10 | 1000 | 0 |

**ABR**: ABR 2 tone burst 4000Hz 1
: Cz-M1

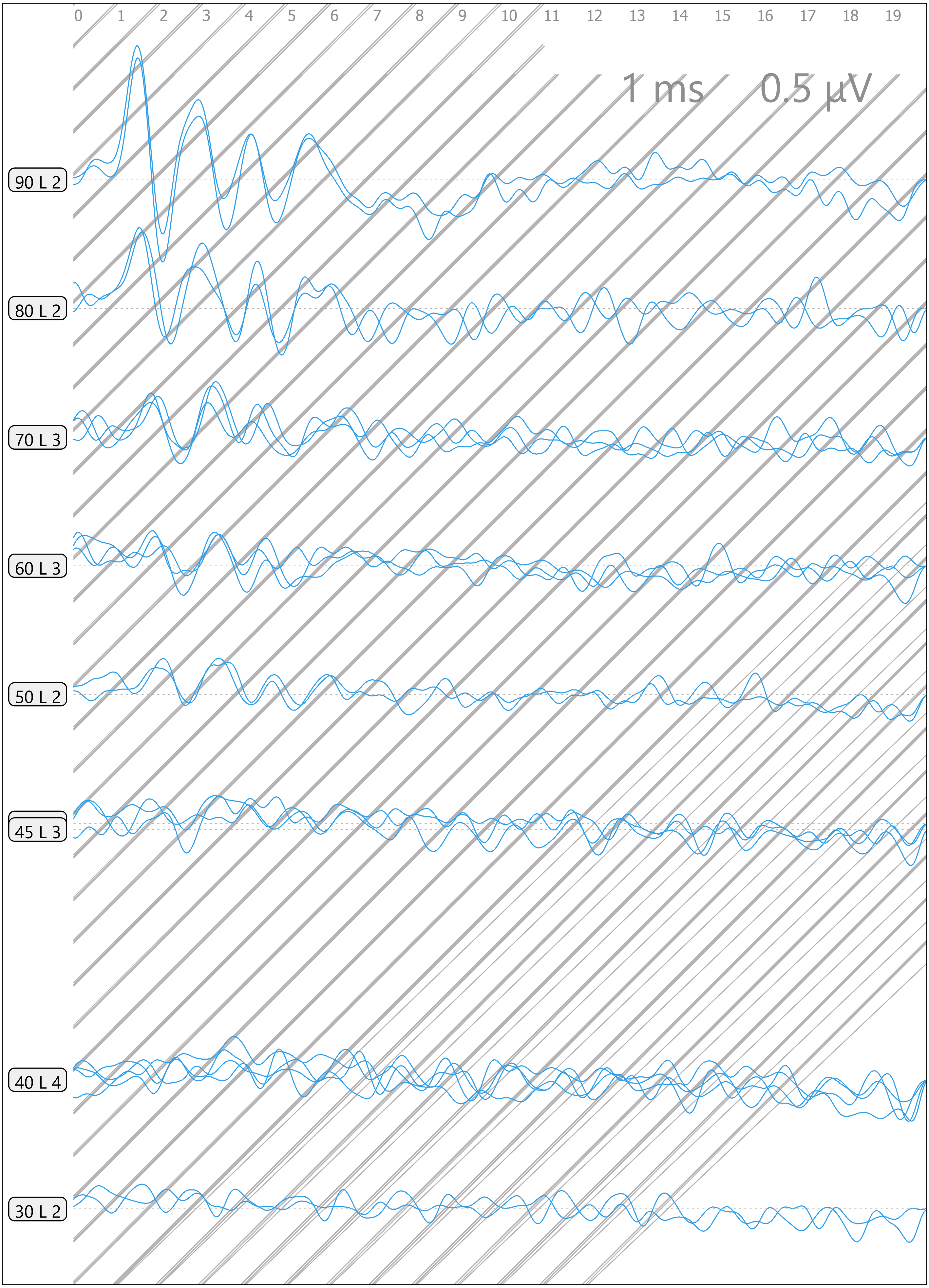
0 1 2 3 4 5 6 7 8 9 10 11 12 13 14 15 16 17 18 19
1 ms
0.5 µV
90 L 2
80 L 2
70 L 3
60 L 3
50 L 2
45 L 3
40 L 4
30 L 2

Trace parameters

| N | Electr. | HPF, Hz | LPF, Hz | 50 Hz | Rejection ±μV | Aver. | Reject. |
|---|---|---|---|---|---|---|---|
| 90 L | Cz-M1 | 200 | 2000 | | 10 | 1000 | 0 |
| 90 L 2 | Cz-M1 | 200 | 2000 | | 10 | 1000 | 0 |
| 80 L | Cz-M1 | 200 | 2000 | | 10 | 1000 | 0 |
| 80 L 2 | Cz-M1 | 200 | 2000 | | 10 | 1000 | 0 |
| 70 L | Cz-M1 | 200 | 2000 | | 10 | 1000 | 0 |
| 70 L 2 | Cz-M1 | 200 | 2000 | | 10 | 1000 | 0 |
| 70 L 3 | Cz-M1 | 200 | 2000 | | 10 | 1000 | 0 |
| 60 L | Cz-M1 | 200 | 2000 | | 10 | 1000 | 0 |
| 60 L 2 | Cz-M1 | 200 | 2000 | | 10 | 1000 | 0 |
| 60 L 3 | Cz-M1 | 200 | 2000 | | 10 | 1000 | 0 |
| 50 L | Cz-M1 | 200 | 2000 | | 10 | 1000 | 0 |
| 50 L 2 | Cz-M1 | 200 | 2000 | | 10 | 1000 | 0 |
| 45 L | Cz-M1 | 200 | 2000 | | 10 | 1000 | 0 |
| 45 L 2 | Cz-M1 | 200 | 2000 | | 10 | 1000 | 0 |
| 45 L 3 | Cz-M1 | 200 | 2000 | | 10 | 1000 | 0 |
| 40 L | Cz-M1 | 200 | 2000 | | 10 | 1000 | 0 |
| 40 L 2 | Cz-M1 | 200 | 2000 | | 10 | 1000 | 0 |
| 40 L 3 | Cz-M1 | 200 | 2000 | | 10 | 1000 | 0 |
| 40 L 4 | Cz-M1 | 200 | 2000 | | 10 | 1000 | 0 |
| 30 L | Cz-M1 | 200 | 2000 | | 10 | 1000 | 0 |
| 30 L 2 | Cz-M1 | 200 | 2000 | | 10 | 1000 | 0 |

**ABR**: ABR 2 8000Hz 1: Cz-M1

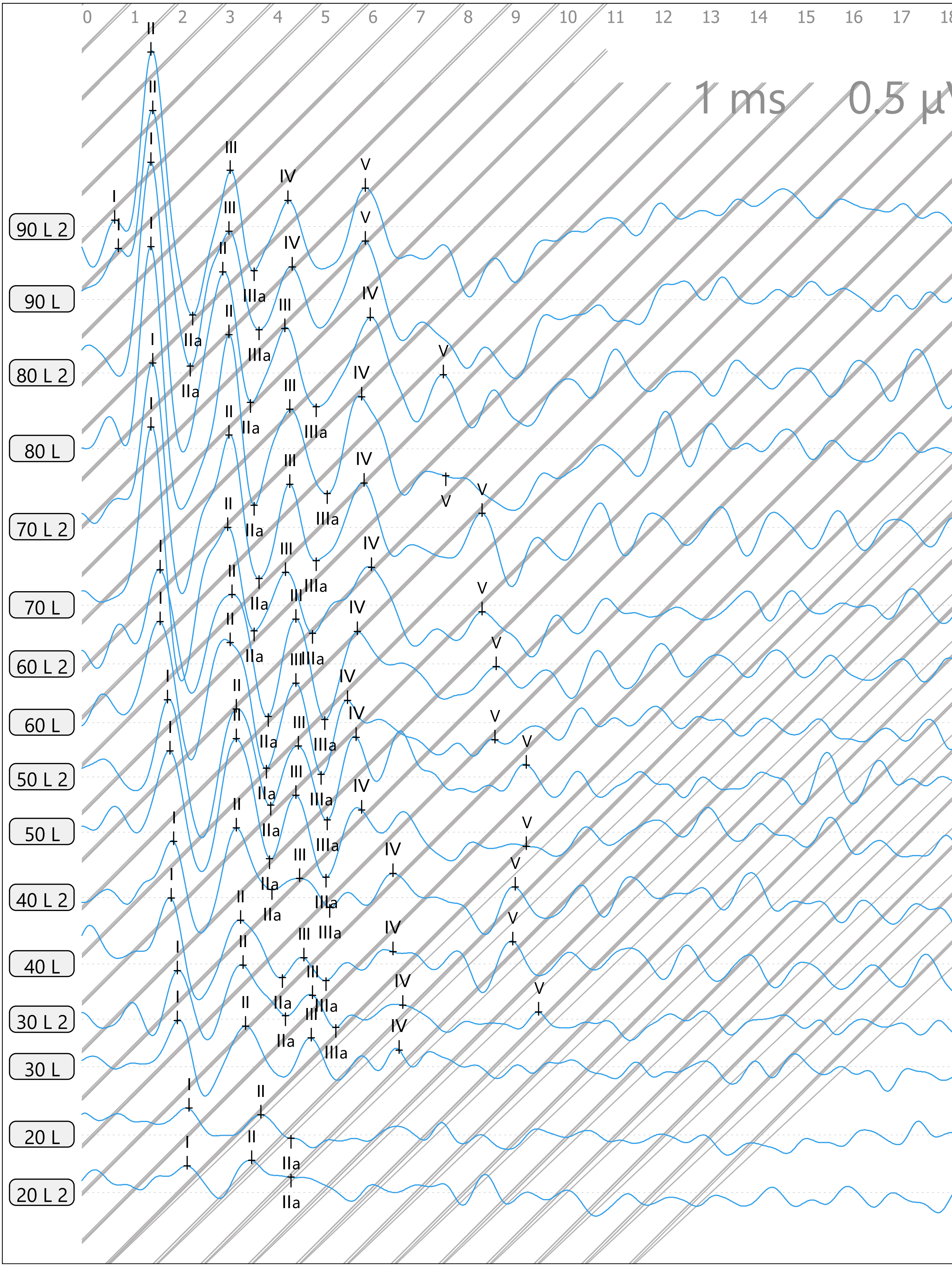
0
1
2
3
4
5
6
7
8
9
10
11
12
13
14
15
16
17
1 ms
90 L 2
90 L
80 L 2
80 L
70 L 2
70 L
60 L 2
60 L
50 L 2
50 L
40 L 2
40 L
30 L 2
30 L
20 L
20 L 2
I
II
IIa
III
IIIa
IV
V

&& (left ear

| N | I (ms) | II (ms) | III (ms) | IV (ms) | V (ms) |
|---|---|---|---|---|---|
| 90 L | 0.77 | 1.48 | 3.10 | 4.42 | 5.95 |
| 90 L 2 | 0.69 | 1.46 | 3.12 | 4.34 | 5.95 |
| 80 L | 1.46 | 3.10 | 4.37 | 5.87 | 7.65 |
| 80 L 2 | 1.46 | 2.96 | 4.26 | 6.06 | 7.59 |
| 70 L | 1.46 | 3.07 | 4.29 | 6.09 | 8.41 |
| 70 L 2 | 1.48 | 3.10 | 4.37 | 5.93 | 8.41 |
| 60 L | 1.64 | 3.12 | 4.50 | 5.58 | 8.68 |
| 60 L 2 | 1.64 | 3.15 | 4.50 | 5.79 | 8.70 |
| 50 L | 1.85 | 3.25 | 4.50 | 5.87 | 9.34 |
| 50 L 2 | 1.80 | 3.25 | 4.55 | 5.77 | 9.34 |
| 40 L | 1.88 | 3.33 | 4.66 | 6.54 | 9.05 |
| 40 L 2 | 1.93 | 3.25 | 4.58 | 6.54 | 9.10 |
| 30 L | 2.01 | 3.44 | 4.82 | 6.67 | |
| 30 L 2 | 2.01 | 3.39 | 4.84 | 6.75 | 9.60 |
| 20 L | 2.25 | 3.76 | | | |
| 20 L 2 | 2.22 | 3.57 | | | |

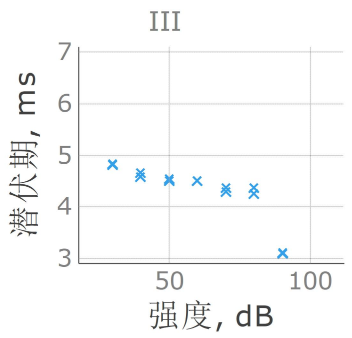


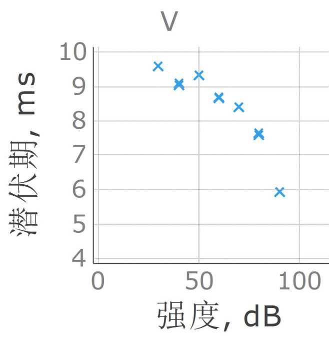


Trace parameters

| N | Electr. | HPF, Hz | LPF, Hz | 50 Hz | Rejection ±μV | Aver. | Reject. |
|---|---|---|---|---|---|---|---|
| 90 L | Cz-M1 | 200 | 2000 | | 10 | 1000 | 0 |
| 90 L 2 | Cz-M1 | 200 | 2000 | | 10 | 1000 | 0 |
| 80 L | Cz-M1 | 200 | 2000 | | 10 | 1000 | 0 |
| 80 L 2 | Cz-M1 | 200 | 2000 | | 10 | 1000 | 0 |
| 70 L | Cz-M1 | 200 | 2000 | | 10 | 1000 | 0 |
| 70 L 2 | Cz-M1 | 200 | 2000 | | 10 | 1000 | 0 |
| 60 L | Cz-M1 | 200 | 2000 | | 10 | 1000 | 0 |
| 60 L 2 | Cz-M1 | 200 | 2000 | | 10 | 1000 | 0 |
| 50 L | Cz-M1 | 200 | 2000 | | 10 | 1000 | 0 |
| 50 L 2 | Cz-M1 | 200 | 2000 | | 10 | 1000 | 0 |
| 40 L | Cz-M1 | 200 | 2000 | | 10 | 1000 | 0 |
| 40 L 2 | Cz-M1 | 200 | 2000 | | 10 | 1000 | 0 |
| 30 L | Cz-M1 | 200 | 2000 | | 10 | 1000 | 0 |
| 30 L 2 | Cz-M1 | 200 | 2000 | | 10 | 1000 | 0 |

| | | | | | | | |
|---|---|---|---|---|---|---|---|
| | | | | | | | |
| 20 L | Cz-M1 | 200 | 2000 | | 10 | 1000 | 0 |
| 20 L 2 | Cz-M1 | 200 | 2000 | | 10 | 1000 | 0 |

**ABR**: ABR 2 CLICK 2: Cz-M2

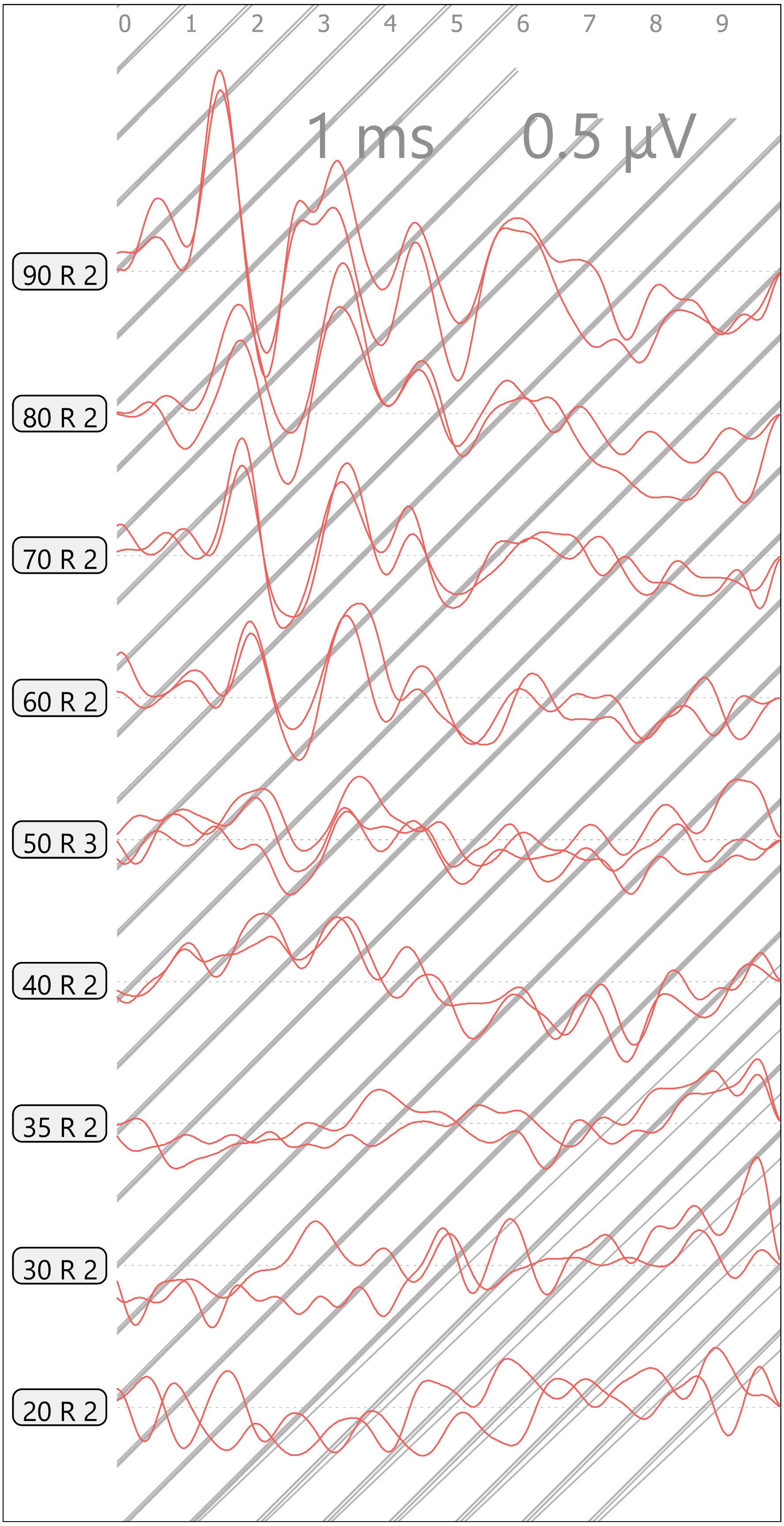


Trace parameters

| N | Electr. | HPF, Hz | LPF, Hz | 50 Hz | Rejection ±µV | Aver. | Reject |
|---|---|---|---|---|---|---|---|
| 90 R | Cz-M2 | 100 | 2000 | | 10 | 1000 | 0 |
| 90 R 2 | Cz-M2 | 100 | 2000 | | 10 | 1000 | 0 |
| 80 R | Cz-M2 | 100 | 2000 | | 10 | 1000 | 0 |
| 80 R 2 | Cz-M2 | 100 | 2000 | | 10 | 1000 | 0 |

| | | | | | | | |
|---|---|---|---|---|---|---|---|
| 70 R | Cz-M2 | 100 | 2000 | | 10 | 1000 | 0 |
| 70 R 2 | Cz-M2 | 100 | 2000 | | 10 | 1000 | 0 |
| 60 R | Cz-M2 | 100 | 2000 | | 10 | 1000 | 0 |
| 60 R 2 | Cz-M2 | 100 | 2000 | | 10 | 1000 | 0 |
| 50 R | Cz-M2 | 100 | 2000 | | 10 | 1000 | 0 |
| 50 R 2 | Cz-M2 | 100 | 2000 | | 10 | 1000 | 0 |
| 50 R 3 | Cz-M2 | 100 | 2000 | | 10 | 1000 | 0 |
| 40 R | Cz-M2 | 100 | 2000 | | 10 | 1000 | 0 |
| 40 R 2 | Cz-M2 | 100 | 2000 | | 10 | 1000 | 0 |
| 35 R | Cz-M2 | 100 | 2000 | | 10 | 1000 | 0 |
| 35 R 2 | Cz-M2 | 100 | 2000 | | 10 | 1000 | 0 |
| 30 R | Cz-M2 | 100 | 2000 | | 10 | 1000 | 0 |
| 30 R 2 | Cz-M2 | 100 | 2000 | | 10 | 1000 | 0 |
| 20 R | Cz-M2 | 100 | 2000 | | 10 | 1000 | 0 |
| 20 R 2 | Cz-M2 | 100 | 2000 | | 10 | 1000 | 0 |

**ABR**: ABR 2 4000Hz 2: Cz-M2

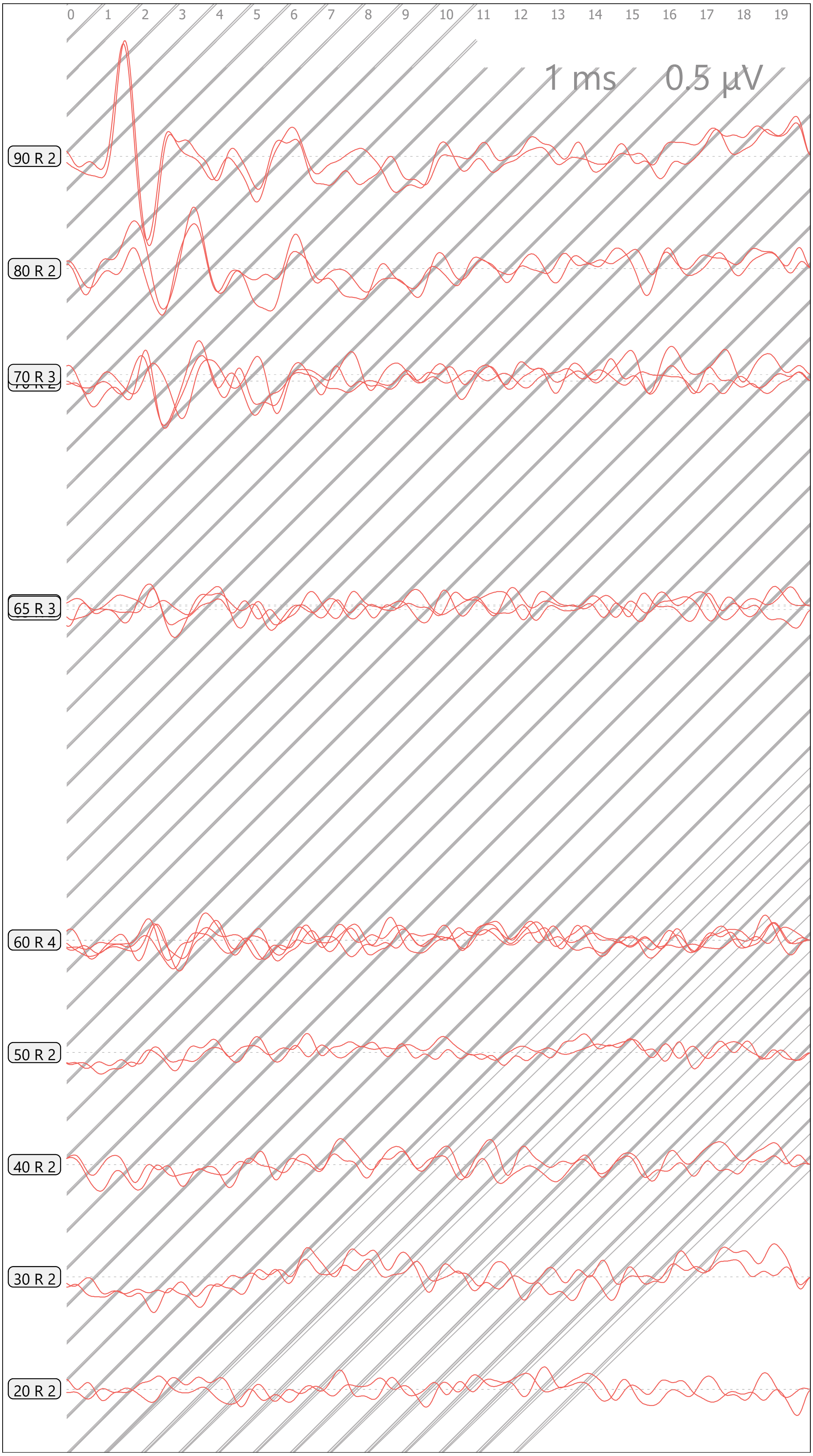
0 1 2 3 4 5 6 7 8 9 10 11 12 13 14 15 16 17 18 19
1 ms
0.5 µV
90 R 2
80 R 2
70 R 3
65 R 3
60 R 4
50 R 2
40 R 2
30 R 2
20 R 2

Trace parameters

| N | Electr. | HPF, Hz | LPF, Hz | 50 Hz | Rejection ±μV | Aver. | Reject |
|---|---|---|---|---|---|---|---|
| 90 R | Cz-M2 | 200 | 2000 | | 10 | 1000 | 0 |
| 90 R 2 | Cz-M2 | 200 | 2000 | | 10 | 1000 | 0 |
| 80 R | Cz-M2 | 200 | 2000 | | 10 | 1000 | 0 |
| 80 R 2 | Cz-M2 | 200 | 2000 | | 10 | 1000 | 0 |
| 70 R | Cz-M2 | 200 | 2000 | | 10 | 1000 | 0 |
| 70 R 2 | Cz-M2 | 200 | 2000 | | 10 | 1000 | 0 |
| 70 R 3 | Cz-M2 | 200 | 2000 | | 10 | 1000 | 0 |
| 65 R | Cz-M2 | 200 | 2000 | | 10 | 1000 | 0 |
| 65 R 2 | Cz-M2 | 200 | 2000 | | 10 | 1000 | 0 |
| 65 R 3 | Cz-M2 | 200 | 2000 | | 10 | 1000 | 0 |
| 60 R | Cz-M2 | 200 | 2000 | | 10 | 1000 | 0 |
| 60 R 2 | Cz-M2 | 200 | 2000 | | 10 | 1000 | 0 |
| 60 R 3 | Cz-M2 | 200 | 2000 | | 10 | 1000 | 0 |
| 60 R 4 | Cz-M2 | 200 | 2000 | | 10 | 1000 | 0 |
| 50 R | Cz-M2 | 200 | 2000 | | 10 | 1000 | 0 |
| 50 R 2 | Cz-M2 | 200 | 2000 | | 10 | 1000 | 0 |
| 40 R | Cz-M2 | 200 | 2000 | | 10 | 1000 | 0 |
| 40 R 2 | Cz-M2 | 200 | 2000 | | 10 | 1000 | 0 |
| 30 R | Cz-M2 | 200 | 2000 | | 10 | 1000 | 0 |
| 30 R 2 | Cz-M2 | 200 | 2000 | | 10 | 1000 | 0 |
| 20 R | Cz-M2 | 200 | 2000 | | 10 | 1000 | 0 |
| 20 R 2 | Cz-M2 | 200 | 2000 | | 10 | 1000 | 0 |

**ABR**: ABR 2 8000Hz 2: Cz-M2

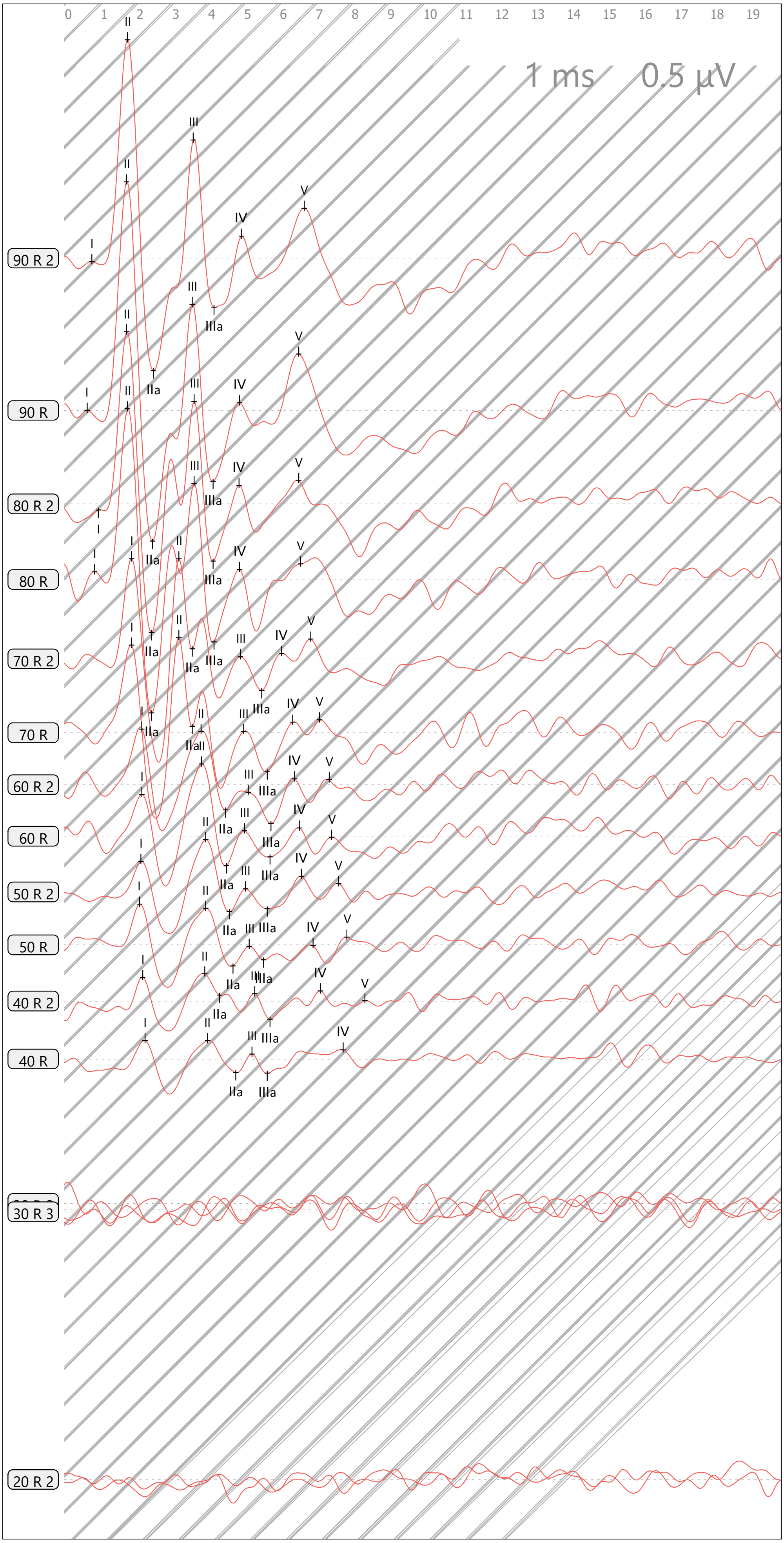
0 1 2 3 4 5 6 7 8 9 10 11 12 13 14 15 16 17 18 19
1 ms
0.5 µV
90 R 2
90 R
80 R 2
80 R
70 R 2
70 R
60 R 2
60 R
50 R 2
50 R
40 R 2
40 R
30 R 3
20 R 2

| IV (ms) | V (ms) | I–III (ms) | I–V (ms) | III–V (ms) | |
|---|---|---|---|---|---|
| 4.89 | 6.54 | 2.94 | 5.90 | 2.96 | |
| 4.95 | 6.69 | 2.83 | 5.93 | 3.10 | |
| 4.89 | 6.59 | 2.78 | 5.74 | 2.96 | |
| 4.87 | 6.54 | 2.67 | 5.58 | 2.91 | |
| 6.38 | 7.12 | 3.12 | 5.24 | 2.12 | |
| 6.06 | 6.88 | 3.04 | 5.00 | 1.96 | |
| 6.56 | 7.46 | 2.86 | 5.29 | 2.43 | |
| 6.43 | 7.38 | 2.96 | 5.21 | 2.25 | |
| 6.93 | 7.88 | 3.07 | 5.79 | 2.73 | |
| 6.61 | 7.65 | 2.91 | 5.50 | 2.59 | |
| 7.78 | | 2.99 | | | |
| 7.14 | 8.39 | 3.12 | 6.19 | 3.07 | |

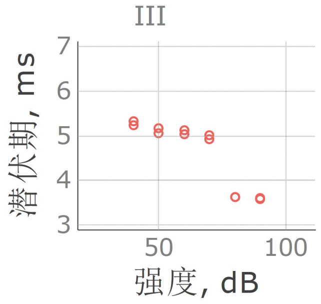


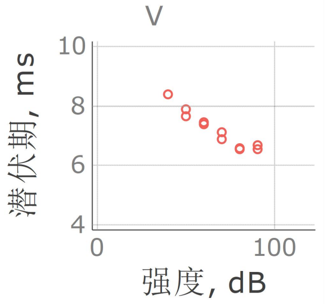


Trace parameters

| N | Electr. | HPF, Hz | LPF, Hz | 50 Hz | Rejection ±μV | Aver. | Reject |
|---|---|---|---|---|---|---|---|
| 90 R | Cz-M2 | 200 | 2000 | | 10 | 1000 | 0 |
| 90 R 2 | Cz-M2 | 200 | 2000 | | 10 | 1000 | 0 |
| 80 R | Cz-M2 | 200 | 2000 | | 10 | 1000 | 0 |
| 80 R 2 | Cz-M2 | 200 | 2000 | | 10 | 1000 | 0 |
| 70 R | Cz-M2 | 200 | 2000 | | 10 | 1000 | 0 |
| 70 R 2 | Cz-M2 | 200 | 2000 | | 10 | 1000 | 0 |
| 60 R | Cz-M2 | 200 | 2000 | | 10 | 1000 | 0 |
| 60 R 2 | Cz-M2 | 200 | 2000 | | 10 | 1000 | 0 |
| 50 R | Cz-M2 | 200 | 2000 | | 10 | 1000 | 0 |
| 50 R 2 | Cz-M2 | 200 | 2000 | | 10 | 1000 | 0 |
| 40 R | Cz-M2 | 200 | 2000 | | 10 | 1000 | 0 |
| 40 R 2 | Cz-M2 | 200 | 2000 | | 10 | 1000 | 0 |
| 30 R | Cz-M2 | 200 | 2000 | | 10 | 1000 | 0 |
| 30 R 2 | Cz-M2 | 200 | 2000 | | 10 | 1000 | 0 |
| 30 R 3 | Cz-M2 | 200 | 2000 | | 10 | 1000 | 0 |
| 20 R | Cz-M2 | 200 | 2000 | | 10 | 1000 | 0 |
| 20 R 2 | Cz-M2 | 200 | 2000 | | 10 | 1000 | 0 |

| | | | | | | | |
|---|---|---|---|---|---|---|---|

**DPOAE**: 1-12 kHz 70/70 dB 3 points

**Test result (right ear):**

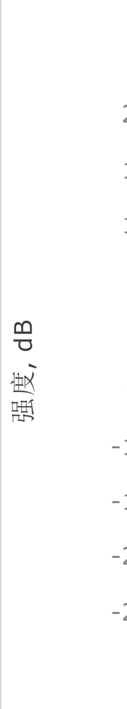


DPOAE (left ear)

| F2, Hz | L1, dB | L2, dB | DP, dB | 噪声, dB | SNR, dB | OAE |
|---|---|---|---|---|---|---|
| 988 | 68.1 | 68.4 | -9.57 | -9.52 | -0.1 | ✘ |
| 1270 | 68.8 | 69.1 | -19.67 | -8.24 | -11.4 | ✘ |
| 1778 | 69.6 | 69.7 | -13.09 | -14.88 | 1.8 | ✘ |
| 2222 | 70.0 | 70.0 | -15.24 | -15.00 | -0.2 | ✘ |
| 2500 | 70.2 | 70.1 | -18.31 | -15.00 | -3.3 | ✘ |
| 3200 | 70.4 | 70.4 | -26.03 | -15.00 | -11.0 | ✘ |
| 4444 | 70.9 | 70.7 | -13.42 | -15.00 | 1.6 | ✘ |
| 5000 | 70.9 | 70.5 | 2.73 | -11.78 | 14.5 | ✔ |
| 6154 | 70.7 | 70.6 | 2.97 | -8.45 | 11.4 | ✔ |
| 8000 | 70.5 | 69.8 | 4.01 | -6.53 | 10.5 | ✔ |
| 8889 | 70.5 | 70.3 | 14.91 | -4.39 | 19.3 | ✔ |
| 10000 | 69.8 | 62.1 | 19.50 | -10.02 | 29.5 | ✔ |
| 11429 | 64.5 | 58.3 | 11.27 | -5.59 | 16.9 | ✔ |
| (dB SPL) | :: 0.0 | | | | | |

**EcochG**: ECochG 1:
Fpz-M1

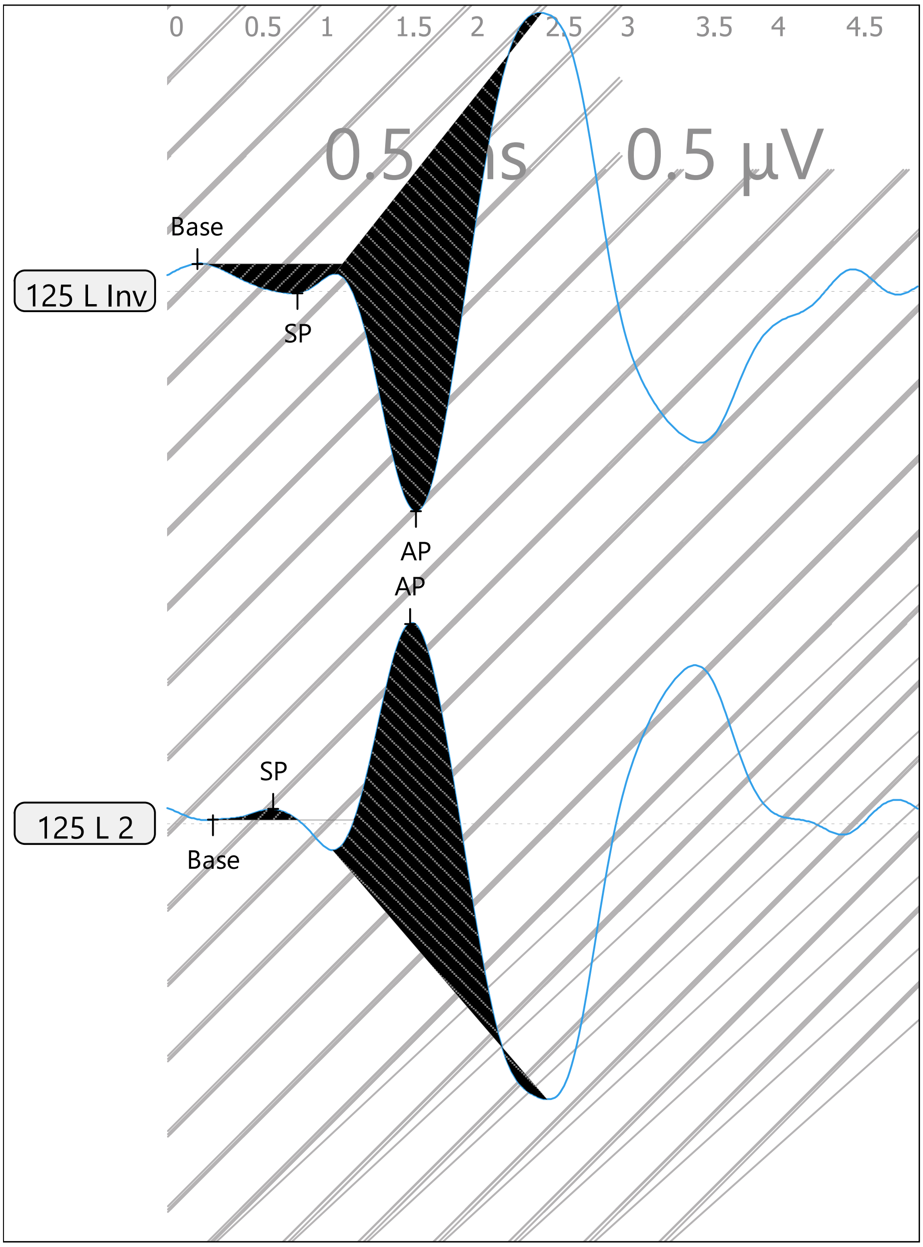


&&

| N | Base (ms) | SP (ms) | AP (ms) | SP–Base (ms) | AP–Base (ms) | SP–Base (µV) | AP–Base (µV) | [illegible] |
|---|---|---|---|---|---|---|---|---|
| 125 L Inv | 0.20 | 0.86 | 1.65 | 0.66 | 1.46 | 0.20 | 1.64 | 0.1[illegible] |
| 125 L 2 | 0.30 | 0.70 | 1.61 | 0.40 | 1.31 | 0.07 | 1.30 | 0.0[illegible] |

Trace parameters

| N | Electr. | HPF, Hz | LPF, Hz | 50 Hz | Rejection ±µV | Aver. | Rej[illegible] |
|---|---|---|---|---|---|---|---|
| 125 L Inv | Fpz-M1 | 5 | 2000 | | 50 | 1500 | 1[illegible] |
| 125 L 2 | Fpz-M1 | 5 | 2000 | | 50 | 1500 | 2[illegible] |

**ECochG**: ECochG 2:
Fpz-M2

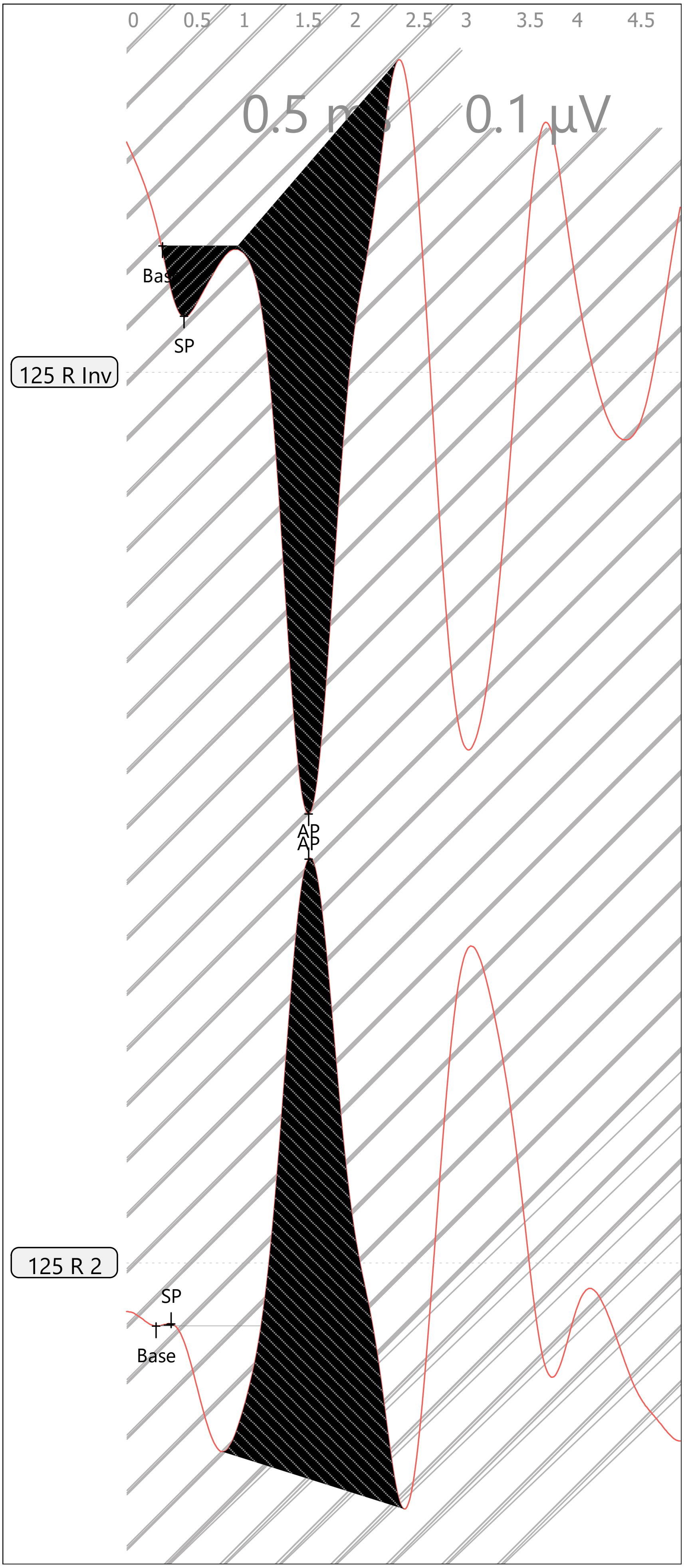

0
0.5
1
1.5
2
2.5
3
3.5
4
4.5
0.5 ms
0.1 µV
Base
SP
125 R Inv
AP
AP
125 R 2
SP
Base

&& (right ear

| N | Base (ms) | SP (ms) | AP (ms) | SP–Base (ms) | AP–Base (ms) | SP–Base (μV) | AP–Base (μV) | |
|---|---|---|---|---|---|---|---|---|
| 125 R Inv | 0.32 | 0.52 | 1.64 | 0.20 | 1.32 | 0.13 | 1.02 | 0.1 |
| 125 R 2 | 0.26 | 0.40 | 1.64 | 0.13 | 1.38 | 0.00 | 0.84 | 0.0 |

Trace parameters

| N | Electr. | HPF, Hz | LPF, Hz | 50 Hz | Rejection ±μV | Aver. | Rej |
|---|---|---|---|---|---|---|---|
| 125 R Inv | Fpz-M2 | 5 | 2000 | | 50 | 1500 | 14 |
| 125 R 2 | Fpz-M2 | 5 | 2000 | | 50 | 1500 | 11 |

**CONCLUSION:**

**Doctor:**
