# Supplementary material for: Exposure to high-altitude hypobaric hypoxic environment induces low-frequency hearing loss in C57BL/6J mice: Mediated by slowing down the postsynaptic electrical signal transmission speed in the cochlear-inferior colliculus auditory signaling pathway
Source: PLoS One. 2026 Mar 11;21(3):e0342321. doi: 10.1371/journal.pone.0342321 (PMC12978441; doi:10.1371/journal.pone.0342321)
Supplement: S1 File — (ZIP) [file pone.0342321.s001.zip › 2025.06.10-5d-4.pdf]

Auditory Evoked Potential Test Report

2025.06.10-5d-4(-)

June 10, 2025

ABR: ABR 2 CLICK
1: Cz-M1

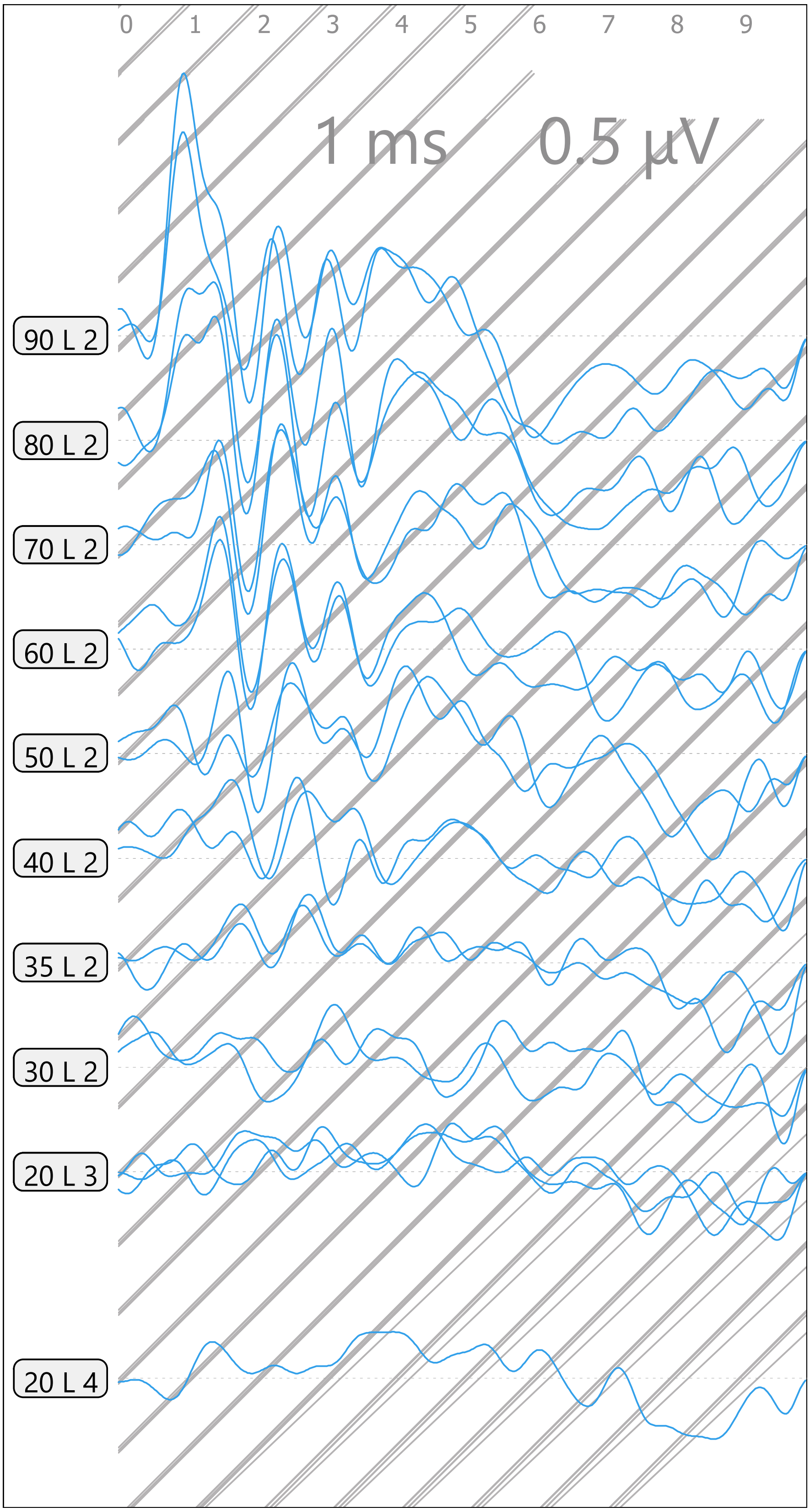

ABR: ABR 2 tone burst 4000Hz 1
: Cz-M1

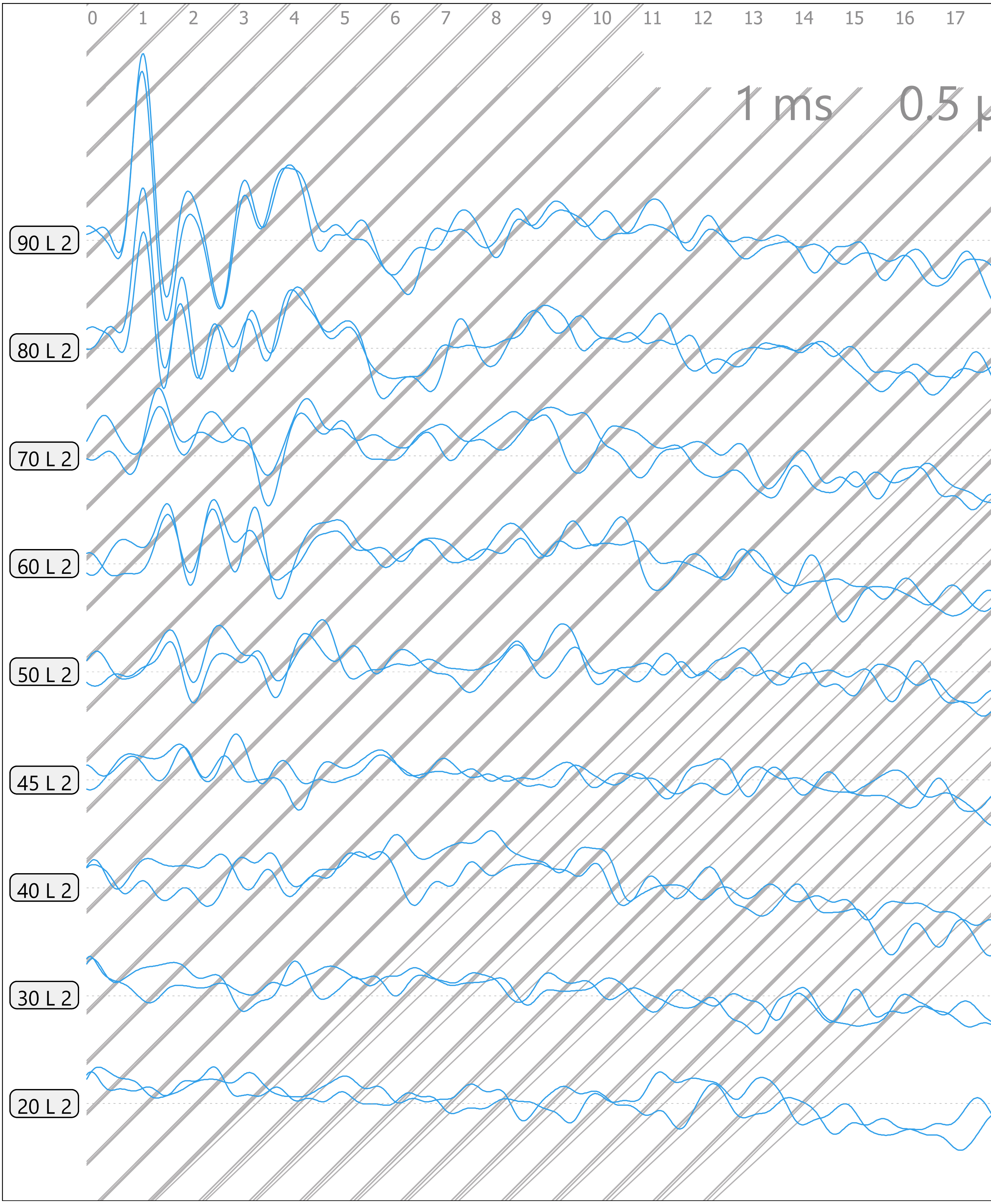

ABR: ABR 2 8000Hz 1: Cz-M1

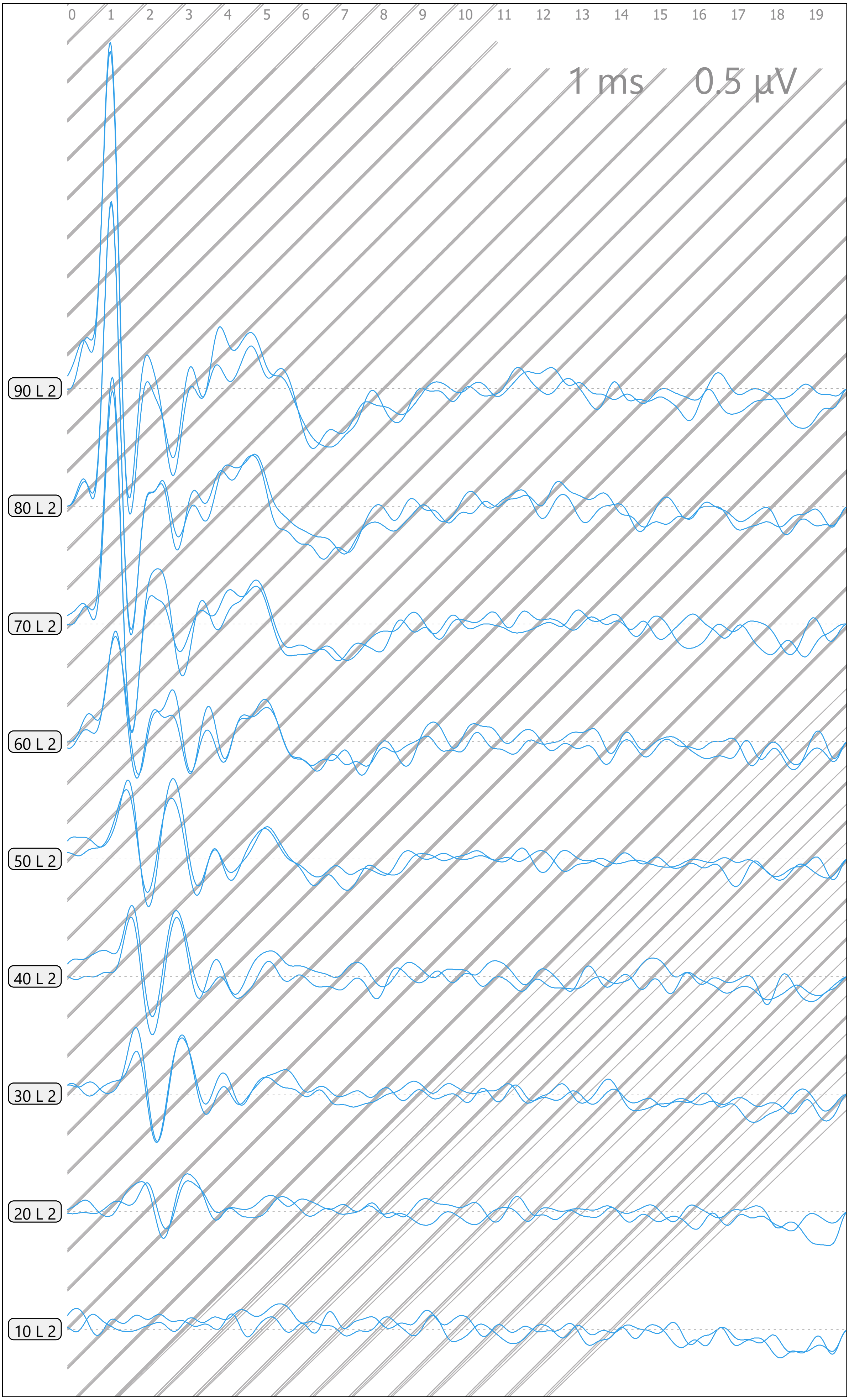
0 1 2 3 4 5 6 7 8 9 10 11 12 13 14 15 16 17 18 19
1 ms
0.5 µV
90 L 2
80 L 2
70 L 2
60 L 2
50 L 2
40 L 2
30 L 2
20 L 2
10 L 2

ABR: ABR 2 CLICK
2: Cz-M2

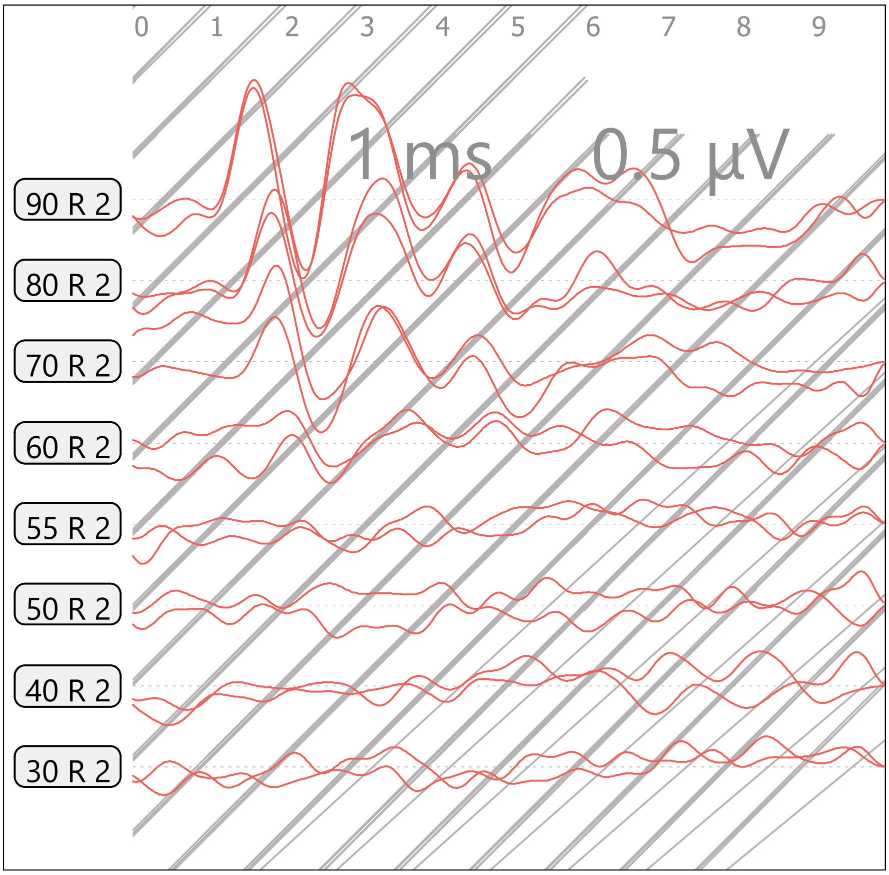

ABR: ABR 2 4000Hz 2: Cz-M2

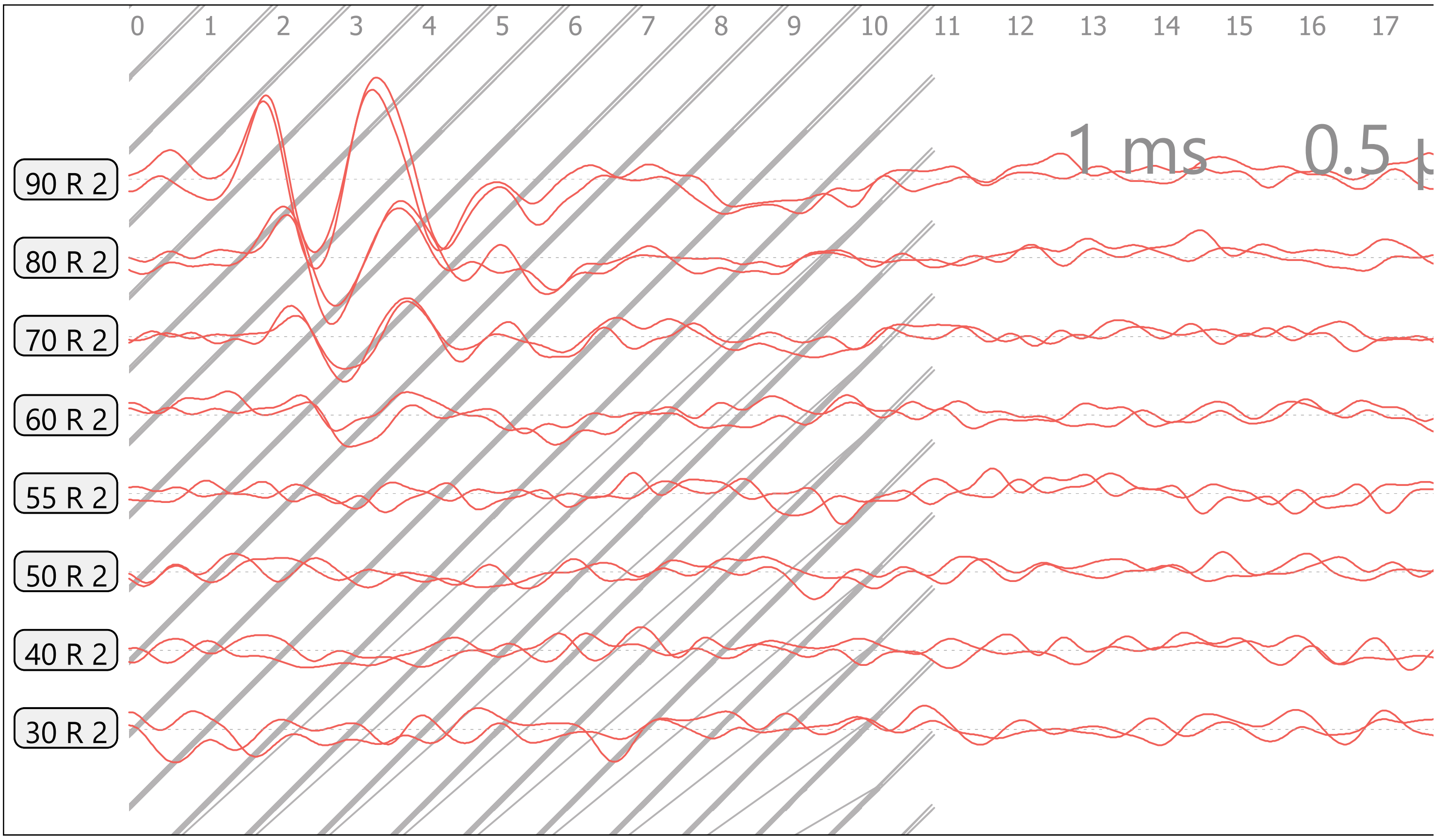

ABR: ABR 2 tone burst 8000Hz 2

: Cz-M2

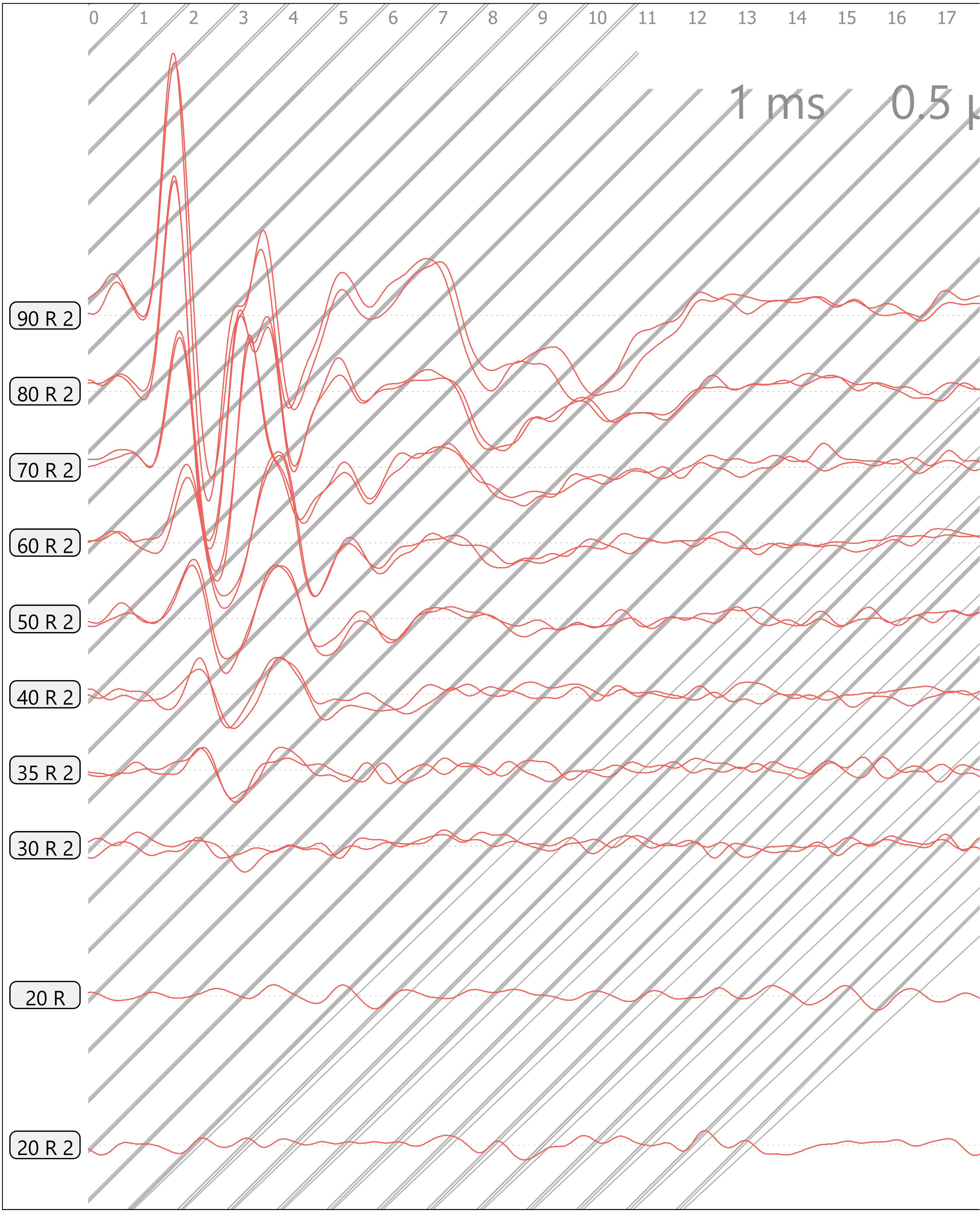

ECochG: ECochG

2: Cz-M2

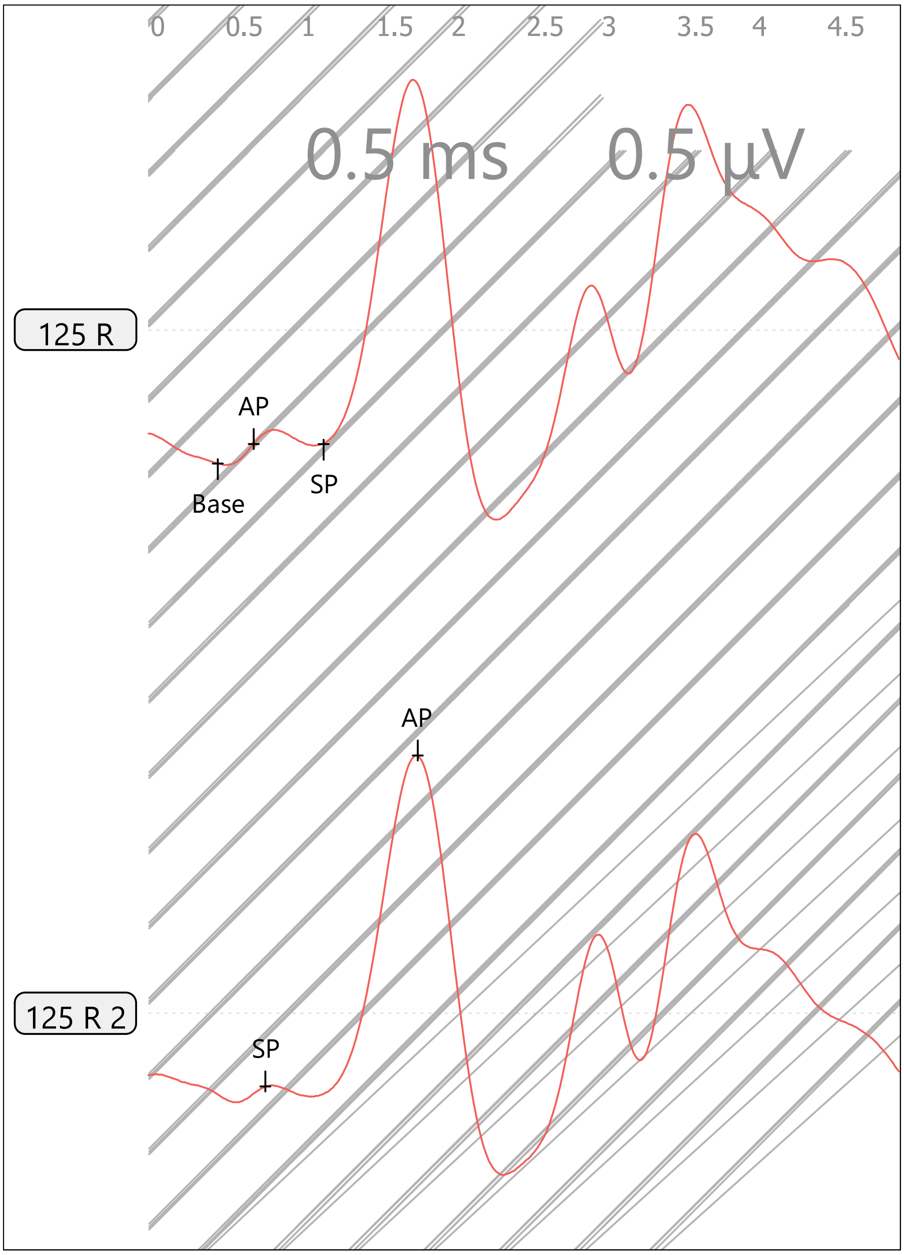

&& (right ear)

	SP–Base (ms)	AP–Base (ms)	SP–Base (µV)	AP–Base (µV)	SP/AP amplitude ratio	SP/AP area ratio
	0.70	0.24	0.13	0.13	1.01	0.00

ECochG: ECochG 1:
Fpz-M1

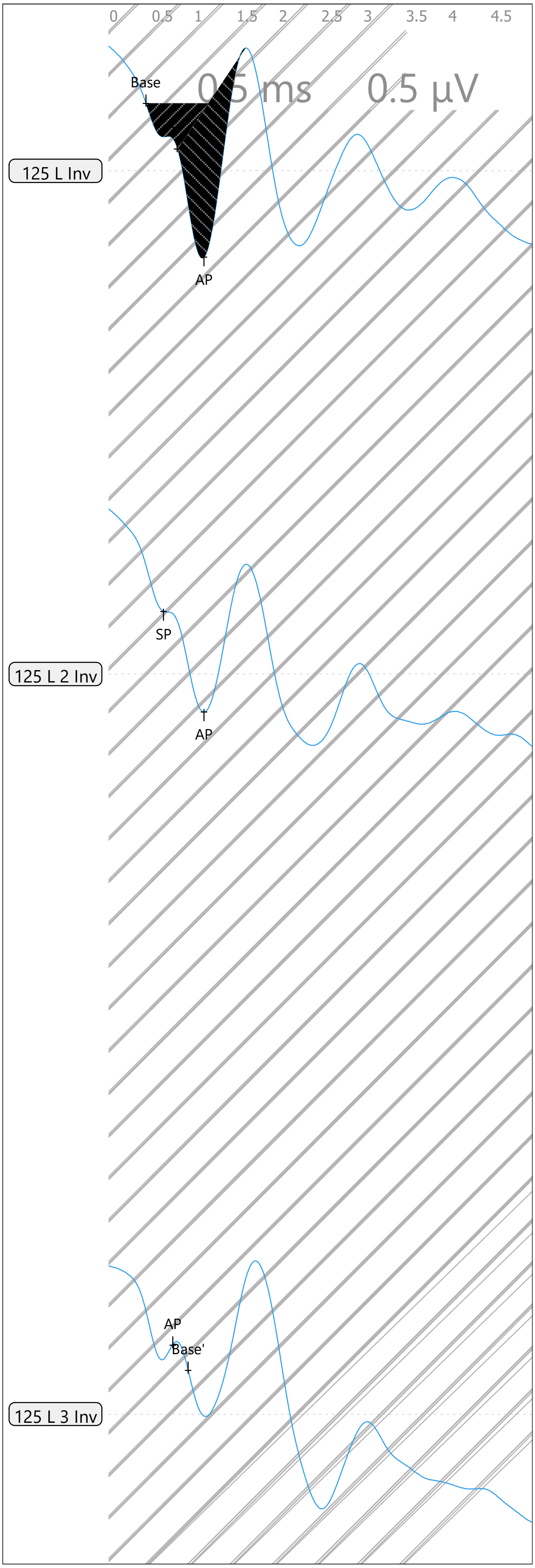
0
0.5
1
1.5
2
2.5
3
3.5
4
4.5
0.5 ms
0.5 µV
Base
AP
125 L Inv
SP
AP
125 L 2 Inv
AP
Base'
125 L 3 Inv

&& (left ear

AP (ms)	SP–Base (ms)	AP–Base (ms)	SP–Base (µV)	AP–Base (µV)	SP/AP amplitude ratio	SP/AP area ratio
	0.37	0.69	0.54	1.81	0.30	1.20
