# Supplementary material for: Exposure to high-altitude hypobaric hypoxic environment induces low-frequency hearing loss in C57BL/6J mice: Mediated by slowing down the postsynaptic electrical signal transmission speed in the cochlear-inferior colliculus auditory signaling pathway
Source: PLoS One. 2026 Mar 11;21(3):e0342321. doi: 10.1371/journal.pone.0342321 (PMC12978441; doi:10.1371/journal.pone.0342321)
Supplement: S1 File — (ZIP) [file pone.0342321.s001.zip › 2025.06.11-10d-04.pdf]

Exam report

Patient: 2025.06.11-5d-04(-)
Date: June 12, 2025

ABR: ABR 2 CLICK
1: Cz-M1

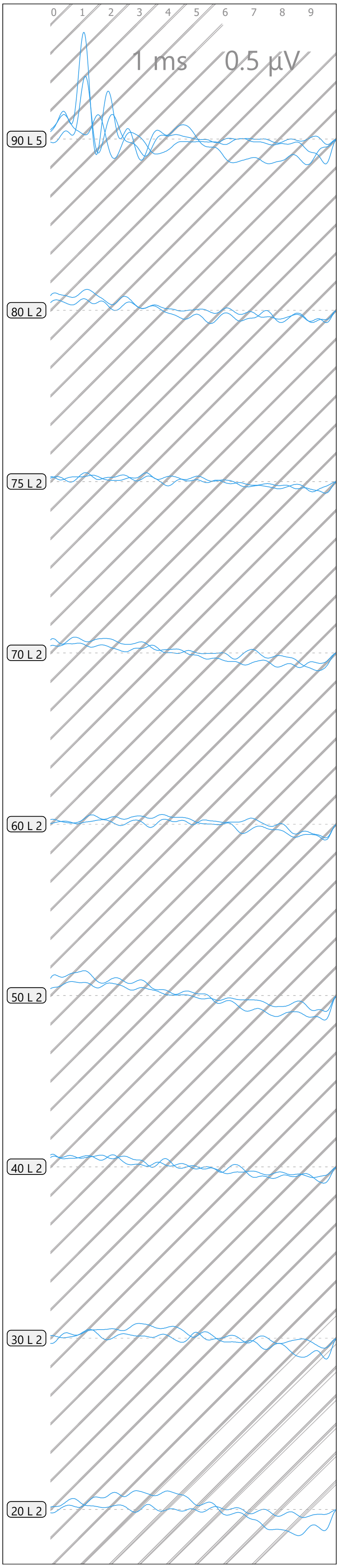
0 1 2 3 4 5 6 7 8 9
1 ms
0.5 µV
90 L 5
80 L 2
75 L 2
70 L 2
60 L 2
50 L 2
40 L 2
30 L 2
20 L 2

Trace parameters

N	Electr.	HPF, Hz	LPF, Hz	50 Hz	Rejection ±μV	Aver.	Reject.
90 L 3	Cz-M1	100	2000		10	1000	0
90 L 4	Cz-M1	100	2000		10	1000	0
90 L 5	Cz-M1	100	2000		10	1000	0
80 L	Cz-M1	100	2000		10	1000	0
80 L 2	Cz-M1	100	2000		10	1000	0
75 L	Cz-M1	100	2000		10	1000	0
75 L 2	Cz-M1	100	2000		10	1000	0
70 L	Cz-M1	100	2000		10	1000	0
70 L 2	Cz-M1	100	2000		10	1000	0
60 L	Cz-M1	100	2000		10	1000	0
60 L 2	Cz-M1	100	2000		10	1000	0
50 L	Cz-M1	100	2000		10	1000	0
50 L 2	Cz-M1	100	2000		10	1000	0
40 L	Cz-M1	100	2000		10	1000	0
40 L 2	Cz-M1	100	2000		10	1000	0
30 L	Cz-M1	100	2000		10	1000	0
30 L 2	Cz-M1	100	2000		10	1000	0
20 L	Cz-M1	100	2000		10	1000	0
20 L 2	Cz-M1	100	2000		10	1000	0

ABR: ABR 2 tone burst 4000Hz 1
: Cz-M1

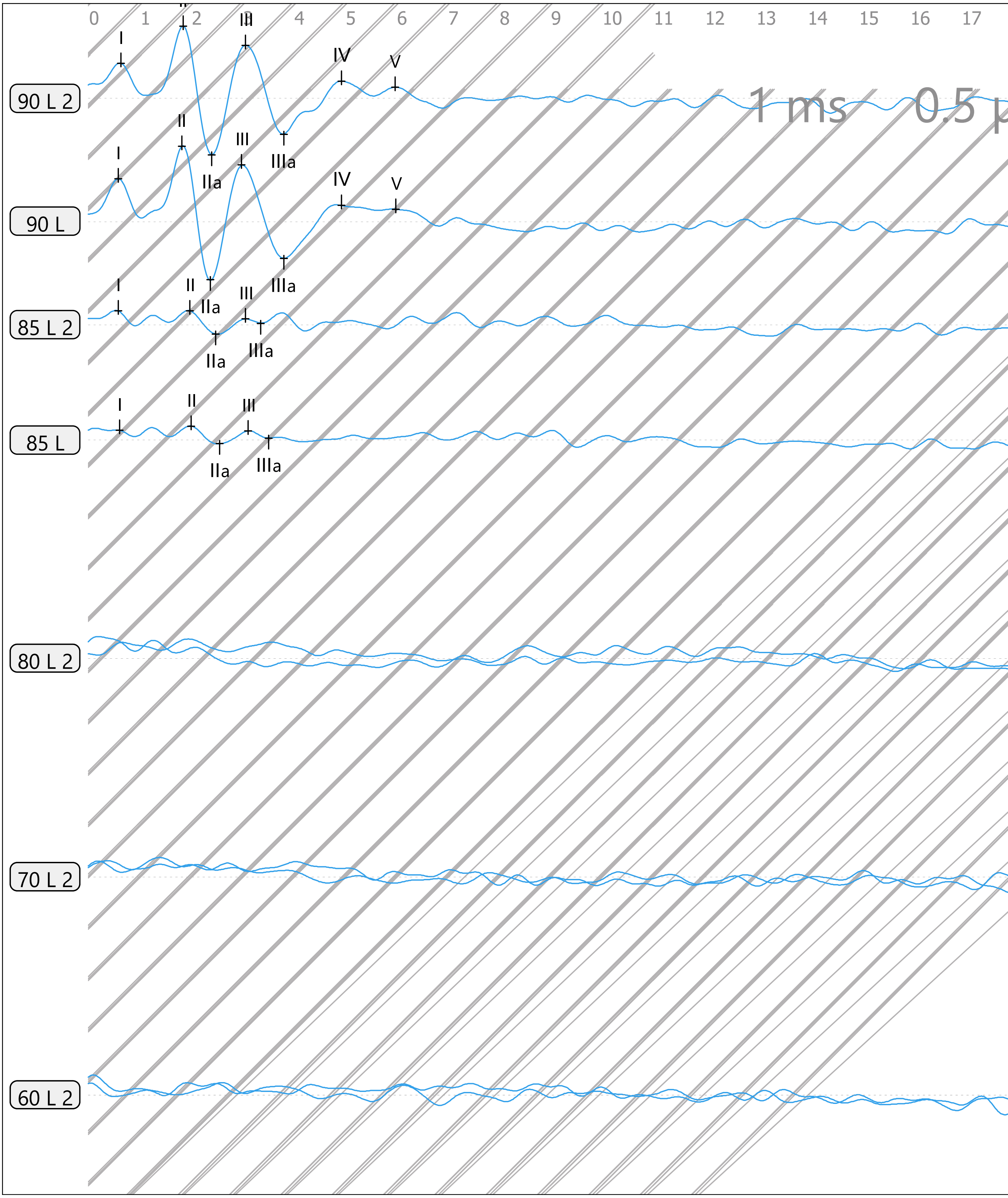

latency&& amplitude (left ear)

N	I (ms)	II (ms)	III (ms)	IV (ms)	V (ms)
90 L	0.58	1.83	2.99	4.95	6.01
90 L 2	0.64	1.85	3.07	4.95	5.98

85 L	0.61	2.01	3.12		
85 L 2	0.58	1.98	3.07		

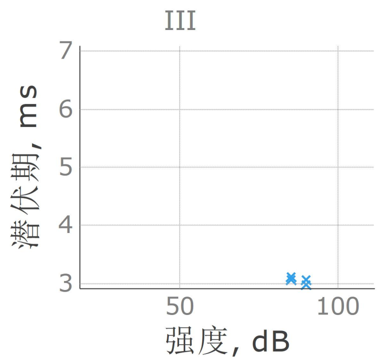

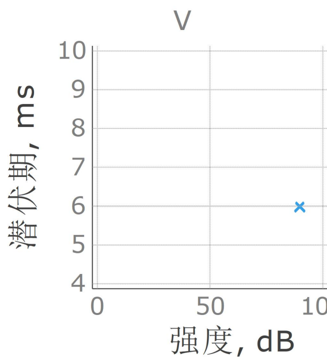

Trace parameters

N	Electr.	HPF, Hz	LPF, Hz	50 Hz	Rejection ±μV	Aver.	Reject.
90 L	Cz-M1	200	2000		10	1000	0
90 L 2	Cz-M1	200	2000		10	1000	0
85 L	Cz-M1	200	2000		10	1000	0
85 L 2	Cz-M1	200	2000		10	1000	0
80 L	Cz-M1	200	2000		10	1000	0
80 L 2	Cz-M1	200	2000		10	1000	0
70 L	Cz-M1	200	2000		10	1000	0
70 L 2	Cz-M1	200	2000		10	1000	0
60 L	Cz-M1	200	2000		10	1000	0
60 L 2	Cz-M1	200	2000		10	1000	0

ABR: ABR 2 tone burst 8000Hz 1
: Cz-M1

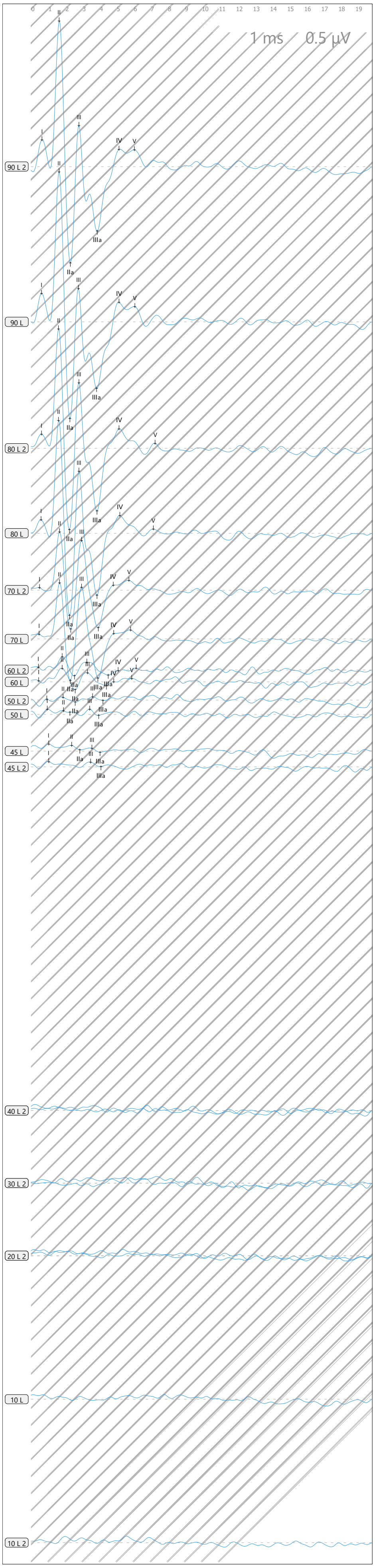
0 1 2 3 4 5 6 7 8 9 10 11 12 13 14 15 16 17 18 19
1 ms
0.5 µV
90 L 2
90 L
80 L 2
80 L
70 L 2
70 L
60 L 2
60 L
50 L 2
50 L
45 L
45 L 2
40 L 2
30 L 2
20 L 2
10 L
10 L 2
I
II
IIa
III
IIIa
IV
V

&& (left ear

N	I (ms)	II (ms)	III (ms)	IV (ms)	V (ms)
90 L	0.61	1.64	2.78	5.16	6.09
90 L 2	0.64	1.64	2.80	5.16	6.06
80 L	0.58	1.61	2.80	5.21	7.17
80 L 2	0.61	1.61	2.80	5.16	7.28
70 L	0.48	1.67	2.96	4.84	5.85
70 L 2	0.50	1.67	2.96	4.82	5.74
60 L	0.45	1.83	3.31	4.84	5.90
60 L 2	0.42	1.83	3.28	5.11	6.16
50 L	0.95	1.91	3.44		
50 L 2	0.93	1.88	3.60		
45 L	1.03	2.38	3.57		
45 L 2	1.03		3.49		

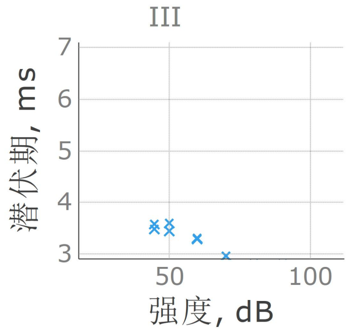

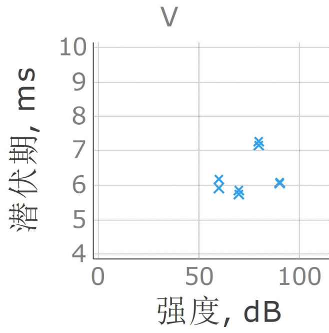

Trace parameters

N	Electr.	HPF, Hz	LPF, Hz	50 Hz	Rejection ±μV	Aver.	Reject.
90 L	Cz-M1	200	2000		10	1000	0
90 L 2	Cz-M1	200	2000		10	1000	0
80 L	Cz-M1	200	2000		10	1000	0
80 L 2	Cz-M1	200	2000		10	1000	0
70 L	Cz-M1	200	2000		10	1000	0
70 L 2	Cz-M1	200	2000		10	1000	0
60 L	Cz-M1	200	2000		10	1000	0
60 L 2	Cz-M1	200	2000		10	1000	0
50 L	Cz-M1	200	2000		10	1000	0
50 L 2	Cz-M1	200	2000		10	1000	0
45 L	Cz-M1	200	2000		10	1000	0
45 L 2	Cz-M1	200	2000		10	1000	0
40 L	Cz-M1	200	2000		10	1000	0
40 L 2	Cz-M1	200	2000		10	1000	0
30 L	Cz-M1	200	2000		10	1000	0
30 L 2	Cz-M1	200	2000		10	1000	0

20 L	Cz-M1	200	2000		10	1000	0
20 L 2	Cz-M1	200	2000		10	1000	0
10 L	Cz-M1	200	2000		10	1000	0
10 L 2	Cz-M1	200	2000		10	1000	0

EcochG: ECochG 1:
Fpz-M1

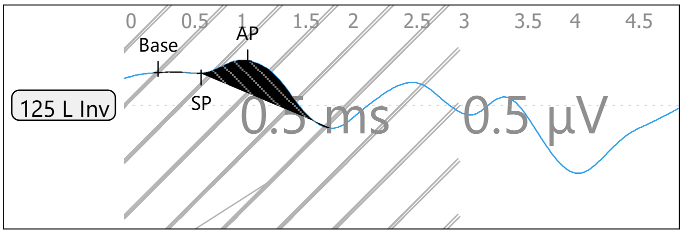

&& (left ear

N	Base (ms)	SP (ms)	AP (ms)	SP–Base (ms)	AP–Base (ms)	SP–Base (µV)	AP–Base (µV)	
125 L Inv	0.30	0.69	1.11	0.38	0.81	0.01	0.11	0.0[illegible]

Trace parameters

N	Electr.	HPF, Hz	LPF, Hz	50 Hz	Rejection ±µV	Aver.	Rej
125 L Inv	Fpz-M1	5	2000		50	822	[illegible]

CONCLUSION:

Doctor:
