# Supplementary material for: Exposure to high-altitude hypobaric hypoxic environment induces low-frequency hearing loss in C57BL/6J mice: Mediated by slowing down the postsynaptic electrical signal transmission speed in the cochlear-inferior colliculus auditory signaling pathway
Source: PLoS One. 2026 Mar 11;21(3):e0342321. doi: 10.1371/journal.pone.0342321 (PMC12978441; doi:10.1371/journal.pone.0342321)
Supplement: S1 File — (ZIP) [file pone.0342321.s001.zip › 2025.06.15-35d-02.pdf]

# Exam report

**Patient:** 2025.06.15-35d-02**,** - ( - )
**Date:** June 15, 2025

**ABR**: ABR 2 CLICK
1: Cz-M1

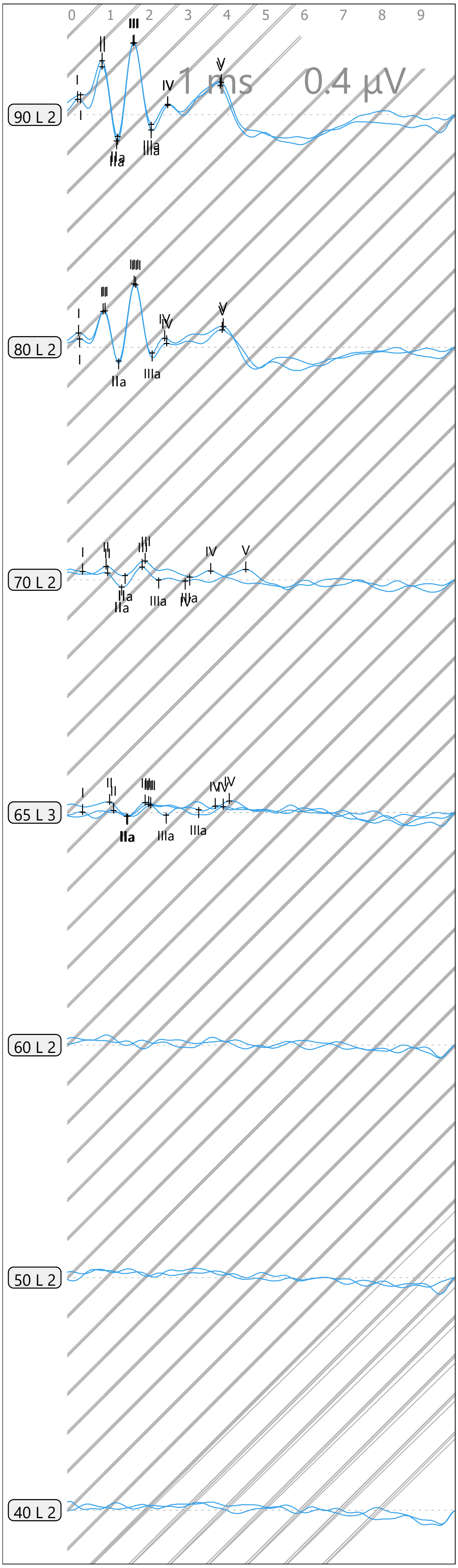

0 1 2 3 4 5 6 7 8 9
1 ms
0.4 µV
90 L 2
80 L 2
70 L 2
65 L 3
60 L 2
50 L 2
40 L 2

latency&& amplitude (left ear

| N | I (ms) | II (ms) | III (ms) | IV (ms) | V (ms) |
|---|---|---|---|---|---|
| 90 L | 0.34 | 0.90 | 1.69 | 2.59 | 3.97 |
| 90 L 2 | 0.26 | 0.90 | 1.72 | 2.59 | 3.94 |
| 80 L | 0.29 | 0.93 | 1.72 | 2.51 | 4.02 |
| 80 L 2 | 0.32 | 0.98 | 1.77 | 2.57 | 4.00 |
| 70 L | 0.40 | 1.03 | 1.93 | 3.04 | 4.60 |
| 70 L 2 | | 1.01 | 2.01 | 3.70 | |
| 65 L | | 1.08 | 2.01 | 3.81 | |
| 65 L 2 | 0.40 | 1.19 | 2.09 | 4.02 | |
| 65 L 3 | | | 2.14 | 4.18 | |

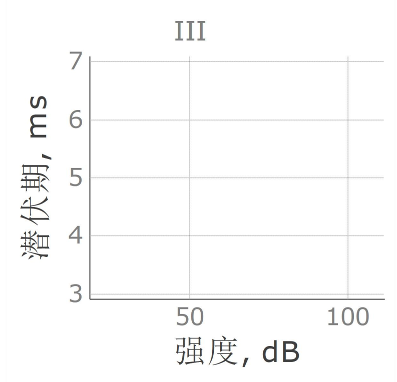


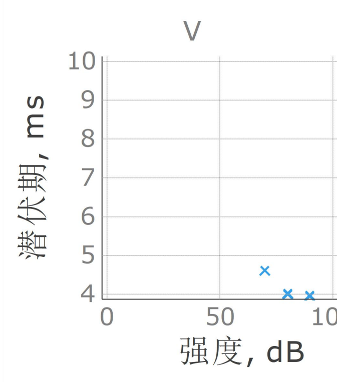


Trace parameters

| N | Electr. | HPF, Hz | LPF, Hz | 50 Hz | Rejection ±μV | Aver. | Reject. |
|---|---|---|---|---|---|---|---|
| 90 L | Cz-M1 | 100 | 2000 | | 10 | 1000 | 0 |
| 90 L 2 | Cz-M1 | 100 | 2000 | | 10 | 1000 | 0 |
| 80 L | Cz-M1 | 100 | 2000 | | 10 | 1000 | 0 |
| 80 L 2 | Cz-M1 | 100 | 2000 | | 10 | 1000 | 0 |
| 70 L | Cz-M1 | 100 | 2000 | | 10 | 1000 | 0 |
| 70 L 2 | Cz-M1 | 100 | 2000 | | 10 | 1000 | 0 |
| 65 L | Cz-M1 | 100 | 2000 | | 10 | 1000 | 0 |
| 65 L 2 | Cz-M1 | 100 | 2000 | | 10 | 1000 | 0 |
| 65 L 3 | Cz-M1 | 100 | 2000 | | 10 | 1000 | 0 |
| 60 L | Cz-M1 | 100 | 2000 | | 10 | 1000 | 0 |
| 60 L 2 | Cz-M1 | 100 | 2000 | | 10 | 1000 | 0 |
| 50 L | Cz-M1 | 100 | 2000 | | 10 | 1000 | 0 |
| 50 L 2 | Cz-M1 | 100 | 2000 | | 10 | 1000 | 0 |
| 40 L | Cz-M1 | 100 | 2000 | | 10 | 1000 | 0 |
| 40 L 2 | Cz-M1 | 100 | 2000 | | 10 | 1000 | 0 |

**ABR**: ABR 2 tone burst 4000Hz 1
: Cz-M1

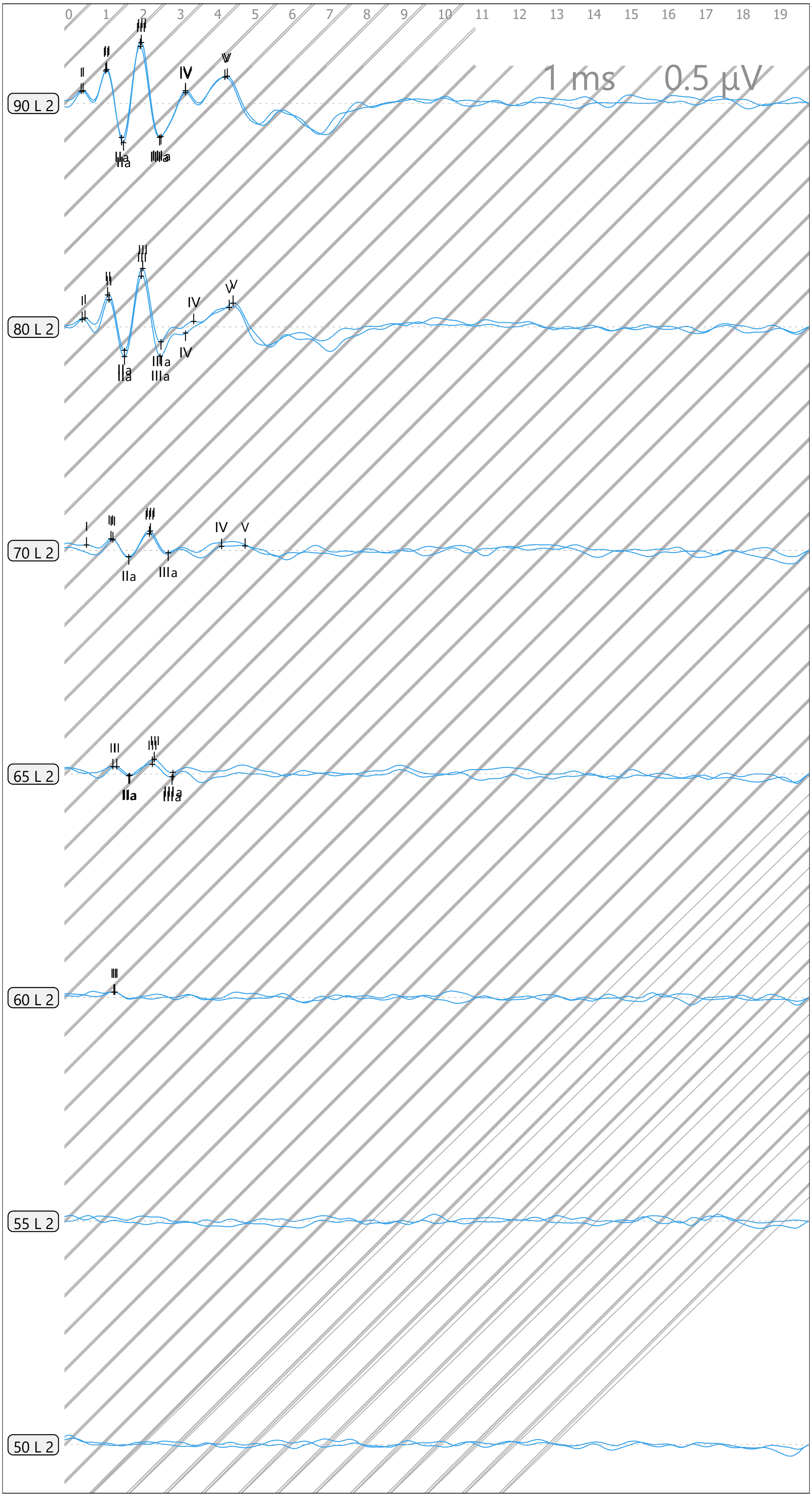

0 1 2 3 4 5 6 7 8 9 10 11 12 13 14 15 16 17 18 19
1 ms
0.5 µV
90 L 2
80 L 2
70 L 2
65 L 2
60 L 2
55 L 2
50 L 2

&& (left ear

| N | I (ms) | II (ms) | III (ms) | IV (ms) | V (ms) |
|---|---|---|---|---|---|
| 90 L | 0.45 | 1.11 | 2.04 | 3.25 | 4.31 |
| 90 L 2 | 0.50 | 1.14 | 2.06 | 3.25 | 4.37 |
| 80 L | 0.48 | 1.19 | 2.06 | 3.25 | 4.42 |
| 80 L 2 | 0.56 | 1.16 | 2.09 | 3.47 | 4.52 |
| 70 L | 0.58 | 1.24 | 2.28 | 4.21 | 4.84 |
| 70 L 2 | | 1.30 | 2.30 | | |
| 65 L | | 1.40 | 2.41 | | |
| 65 L 2 | | 1.30 | 2.35 | | |
| 60 L | | 1.35 | | | |
| 60 L 2 | | 1.32 | | | |

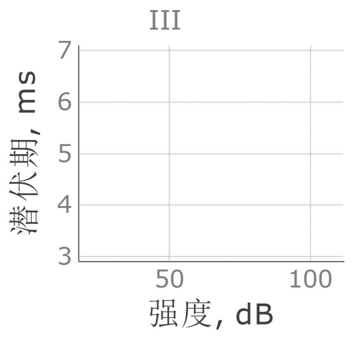


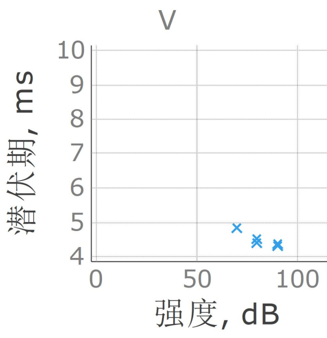


Trace parameters

| N | Electr. | HPF, Hz | LPF, Hz | 50 Hz | Rejection ±μV | Aver. | Reject. |
|---|---|---|---|---|---|---|---|
| 90 L | Cz-M1 | 200 | 2000 | | 10 | 1000 | 0 |
| 90 L 2 | Cz-M1 | 200 | 2000 | | 10 | 1000 | 0 |
| 80 L | Cz-M1 | 200 | 2000 | | 10 | 1000 | 0 |
| 80 L 2 | Cz-M1 | 200 | 2000 | | 10 | 1000 | 0 |
| 70 L | Cz-M1 | 200 | 2000 | | 10 | 1000 | 0 |
| 70 L 2 | Cz-M1 | 200 | 2000 | | 10 | 1000 | 0 |
| 65 L | Cz-M1 | 200 | 2000 | | 10 | 1000 | 0 |
| 65 L 2 | Cz-M1 | 200 | 2000 | | 10 | 1000 | 0 |
| 60 L | Cz-M1 | 200 | 2000 | | 10 | 1000 | 0 |
| 60 L 2 | Cz-M1 | 200 | 2000 | | 10 | 1000 | 0 |
| 55 L | Cz-M1 | 200 | 2000 | | 10 | 1000 | 0 |
| 55 L 2 | Cz-M1 | 200 | 2000 | | 10 | 1000 | 0 |
| 50 L | Cz-M1 | 200 | 2000 | | 10 | 1000 | 0 |
| 50 L 2 | Cz-M1 | 200 | 2000 | | 10 | 1000 | 0 |

**ABR**: ABR 2 8000Hz 1: Cz-M1

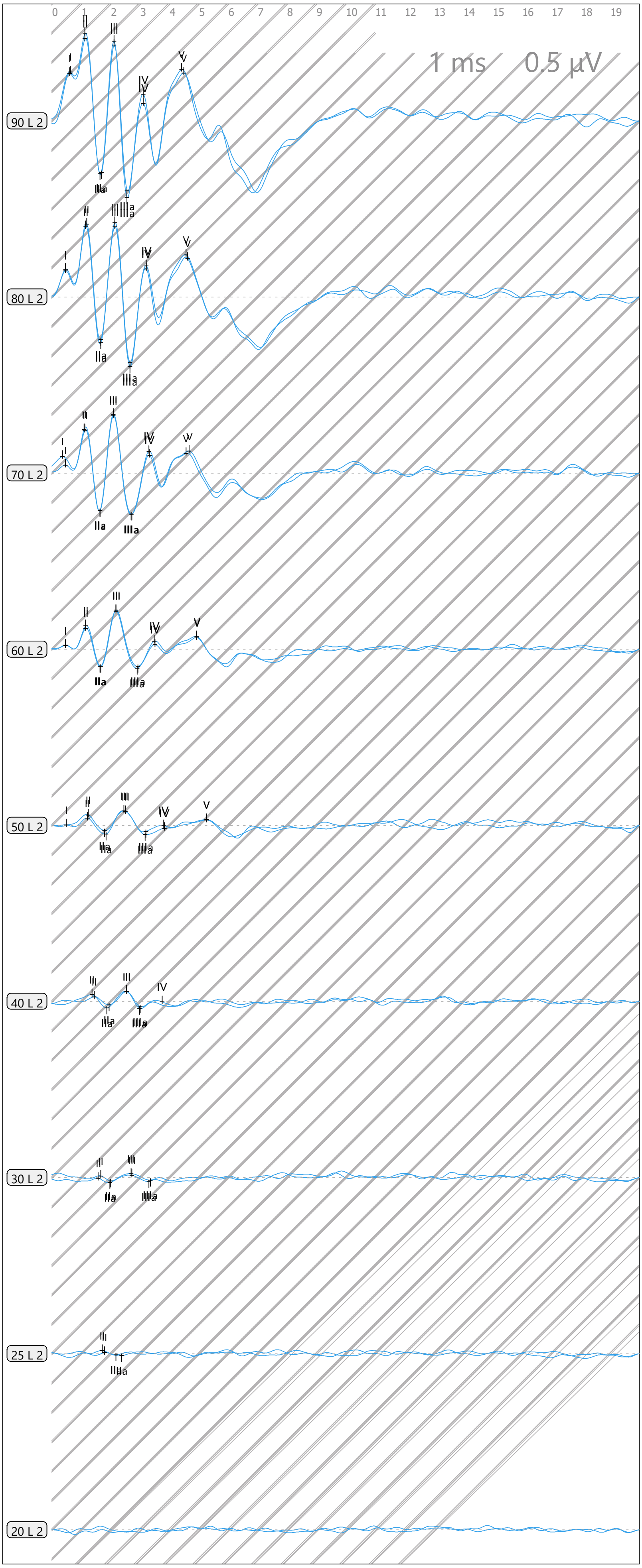

0
1
2
3
4
5
6
7
8
9
10
11
12
13
14
15
16
17
18
19
1 ms
0.5 µV
90 L 2
80 L 2
70 L 2
60 L 2
50 L 2
40 L 2
30 L 2
25 L 2
20 L 2

&& (left ear

| N | I (ms) | II (ms) | III (ms) | IV (ms) | V (ms) |
|---|---|---|---|---|---|
| 90 L | 0.61 | 1.14 | 2.12 | 3.12 | 4.42 |
| 90 L 2 | 0.64 | 1.14 | 2.12 | 3.12 | 4.50 |
| 80 L | 0.48 | 1.16 | 2.14 | 3.23 | 4.63 |
| 80 L 2 | 0.48 | 1.19 | 2.14 | 3.23 | 4.58 |
| 70 L | 0.37 | 1.14 | 2.09 | 3.33 | 4.58 |
| 70 L 2 | 0.48 | 1.11 | 2.09 | 3.31 | 4.68 |
| 60 L | 0.48 | 1.16 | 2.20 | 3.49 | 4.95 |
| 60 L 2 | 0.48 | 1.16 | 2.20 | 3.52 | 4.95 |
| 50 L | 0.50 | 1.22 | 2.46 | 3.84 | 5.27 |
| 50 L 2 | | 1.24 | 2.51 | 3.81 | 5.27 |
| 40 L | | 1.38 | 2.54 | 3.76 | |
| 40 L 2 | | 1.46 | 2.54 | | |
| 30 L | | 1.67 | 2.70 | | |
| 30 L 2 | | 1.59 | 2.73 | | |
| 25 L | | 1.72 | | | |
| 25 L 2 | | 1.80 | | | |

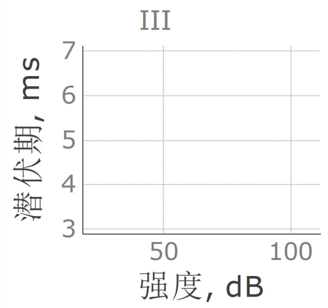


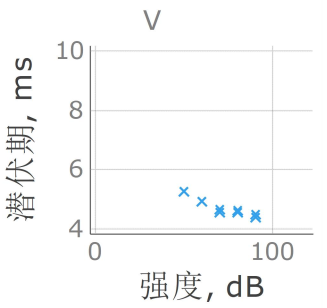


Trace parameters

| N | Electr. | HPF, Hz | LPF, Hz | 50 Hz | Rejection ±µV | Aver. | Reject. |
|---|---|---|---|---|---|---|---|
| 90 L | Cz-M1 | 200 | 2000 | | 10 | 1000 | 0 |
| 90 L 2 | Cz-M1 | 200 | 2000 | | 10 | 1000 | 0 |
| 80 L | Cz-M1 | 200 | 2000 | | 10 | 1000 | 0 |
| 80 L 2 | Cz-M1 | 200 | 2000 | | 10 | 1000 | 0 |
| 70 L | Cz-M1 | 200 | 2000 | | 10 | 1000 | 0 |
| 70 L 2 | Cz-M1 | 200 | 2000 | | 10 | 1000 | 0 |
| 60 L | Cz-M1 | 200 | 2000 | | 10 | 1000 | 0 |
| 60 L 2 | Cz-M1 | 200 | 2000 | | 10 | 1000 | 0 |
| 50 L | Cz-M1 | 200 | 2000 | | 10 | 1000 | 0 |
| 50 L 2 | Cz-M1 | 200 | 2000 | | 10 | 1000 | 0 |
| 40 L | Cz-M1 | 200 | 2000 | | 10 | 1000 | 0 |
| 40 L 2 | Cz-M1 | 200 | 2000 | | 10 | 1000 | 0 |
| 30 L | Cz-M1 | 200 | 2000 | | 10 | 1000 | 0 |
| 30 L 2 | Cz-M1 | 200 | 2000 | | 10 | 1000 | 0 |

| | | | | | | | |
|---|---|---|---|---|---|---|---|
| 25 L | Cz-M1 | 200 | 2000 | | 10 | 1000 | 0 |
| 25 L 2 | Cz-M1 | 200 | 2000 | | 10 | 1000 | 0 |
| 20 L | Cz-M1 | 200 | 2000 | | 10 | 1000 | 0 |
| 20 L 2 | Cz-M1 | 200 | 2000 | | 10 | 1000 | 0 |

**ABR**: ABR 2 CLICK 2: Cz-M2

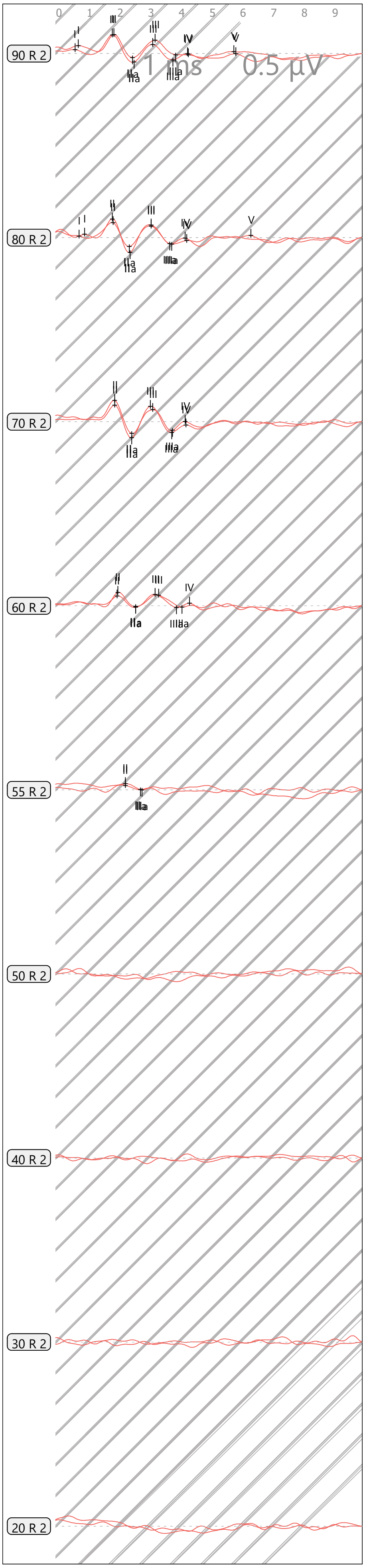
0 1 2 3 4 5 6 7 8 9
1 ms
0.5 µV
90 R 2
80 R 2
70 R 2
60 R 2
55 R 2
50 R 2
40 R 2
30 R 2
20 R 2

| IV (ms) | V (ms) | I–III (ms) | I–V (ms) | III–V (ms) | |
|---|---|---|---|---|---|
| 4.31 | 5.82 | 2.54 | 5.19 | 2.65 | |
| 4.34 | 5.87 | 2.51 | 5.13 | 2.62 | |
| 4.29 | 6.38 | 2.35 | 5.61 | 3.25 | |
| 4.23 | | 2.17 | | | |
| 4.23 | | | | | |
| 4.26 | | | | | |
| 4.37 | | | | | |
| | | | | | |
| | | | | | |
| | | | | | |

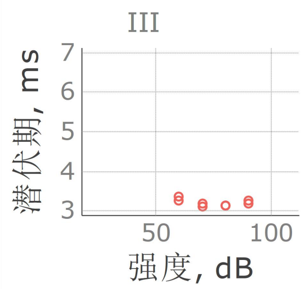


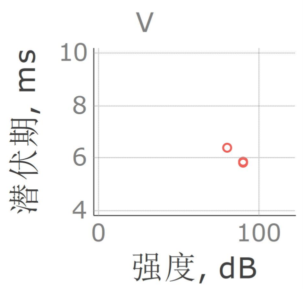


Trace parameters

| N | Electr. | HPF, Hz | LPF, Hz | 50 Hz | Rejection ±µV | Aver. | Reject |
|---|---|---|---|---|---|---|---|
| 90 R | Cz-M2 | 100 | 2000 | | 10 | 1000 | 0 |
| 90 R 2 | Cz-M2 | 100 | 2000 | | 10 | 1000 | 0 |
| 80 R | Cz-M2 | 100 | 2000 | | 10 | 1000 | 0 |
| 80 R 2 | Cz-M2 | 100 | 2000 | | 10 | 1000 | 0 |
| 70 R | Cz-M2 | 100 | 2000 | | 10 | 1000 | 0 |
| 70 R 2 | Cz-M2 | 100 | 2000 | | 10 | 1000 | 0 |
| 60 R | Cz-M2 | 100 | 2000 | | 10 | 1000 | 0 |
| 60 R 2 | Cz-M2 | 100 | 2000 | | 10 | 1000 | 0 |
| 55 R | Cz-M2 | 100 | 2000 | | 10 | 1000 | 0 |
| 55 R 2 | Cz-M2 | 100 | 2000 | | 10 | 1000 | 0 |
| 50 R | Cz-M2 | 100 | 2000 | | 10 | 1000 | 0 |
| 50 R 2 | Cz-M2 | 100 | 2000 | | 10 | 1000 | 0 |
| 40 R | Cz-M2 | 100 | 2000 | | 10 | 1000 | 0 |
| 40 R 2 | Cz-M2 | 100 | 2000 | | 10 | 1000 | 0 |
| 30 R | Cz-M2 | 100 | 2000 | | 10 | 1000 | 0 |
| 30 R 2 | Cz-M2 | 100 | 2000 | | 10 | 1000 | 0 |
| 20 R | Cz-M2 | 100 | 2000 | | 10 | 1000 | 0 |
| 20 R 2 | Cz-M2 | 100 | 2000 | | 10 | 1000 | 0 |

**ABR**: ABR 2 4000Hz 2: Cz-M2

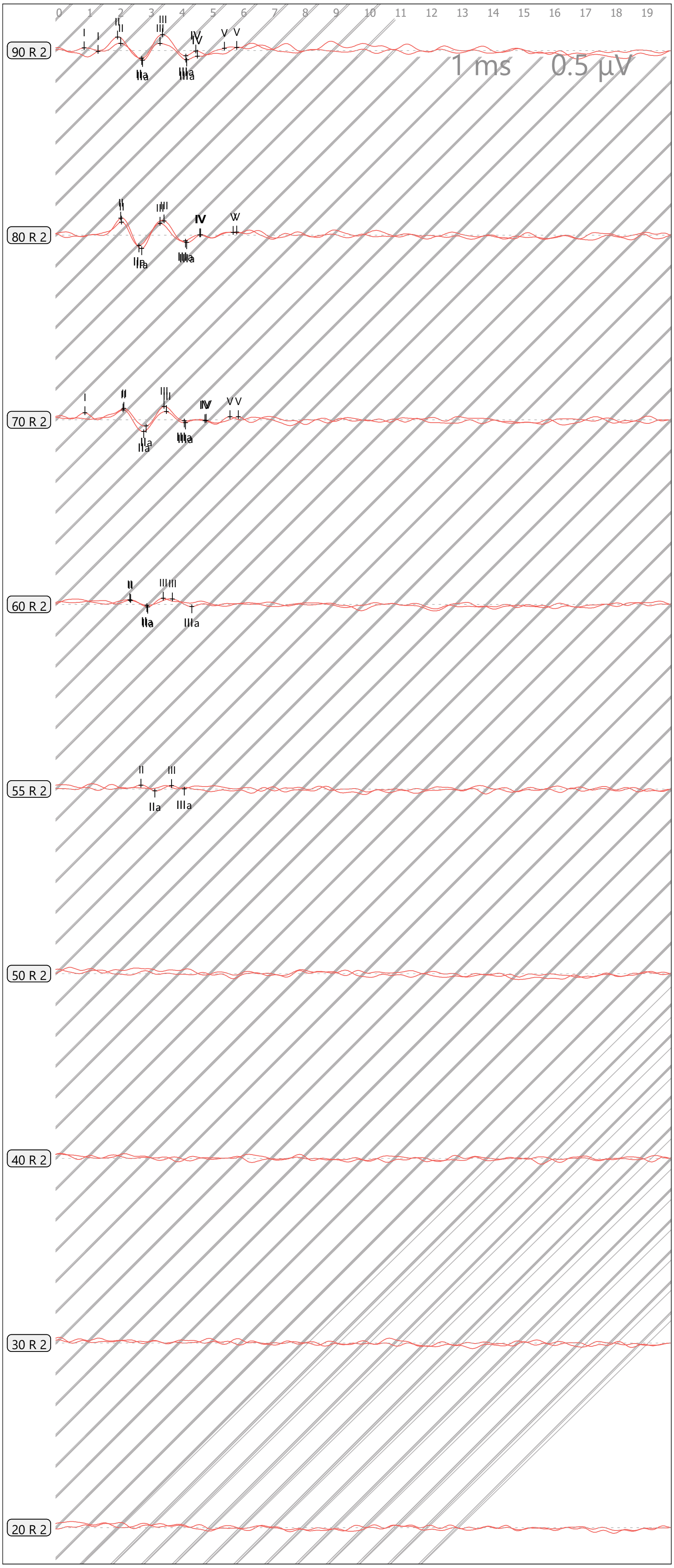

0 1 2 3 4 5 6 7 8 9 10 11 12 13 14 15 16 17 18 19
1 ms
0.5 µV
90 R 2
80 R 2
70 R 2
60 R 2
55 R 2
50 R 2
40 R 2
30 R 2
20 R 2
I
II
IIa
III
IIIa
IV
V

| IV (ms) | V (ms) | I–III (ms) | I–V (ms) | III–V (ms) | |
|---|---|---|---|---|---|
| 4.60 | 5.48 | 2.01 | 4.10 | 2.09 | |
| 4.55 | 5.87 | 2.54 | 4.95 | 2.41 | |
| 4.71 | 5.77 | | | 2.25 | |
| 4.68 | 5.87 | | | 2.49 | |
| 4.89 | 5.93 | 2.57 | 4.97 | 2.41 | |
| 4.84 | 5.66 | | | 2.06 | |
| | | | | | |
| | | | | | |
| | | | | | |

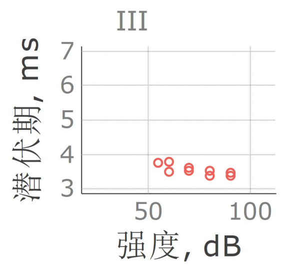


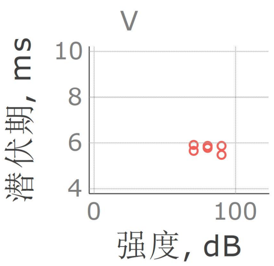


Trace parameters

| N | Electr. | HPF, Hz | LPF, Hz | 50 Hz | Rejection ±µV | Aver. | Reject |
|---|---|---|---|---|---|---|---|
| 90 R | Cz-M2 | 200 | 2000 | | 10 | 1000 | 0 |
| 90 R 2 | Cz-M2 | 200 | 2000 | | 10 | 1000 | 0 |
| 80 R | Cz-M2 | 200 | 2000 | | 10 | 1000 | 0 |
| 80 R 2 | Cz-M2 | 200 | 2000 | | 10 | 1000 | 0 |
| 70 R | Cz-M2 | 200 | 2000 | | 10 | 1000 | 0 |
| 70 R 2 | Cz-M2 | 200 | 2000 | | 10 | 1000 | 0 |
| 60 R | Cz-M2 | 200 | 2000 | | 10 | 1000 | 0 |
| 60 R 2 | Cz-M2 | 200 | 2000 | | 10 | 1000 | 0 |
| 55 R | Cz-M2 | 200 | 2000 | | 10 | 1000 | 0 |
| 55 R 2 | Cz-M2 | 200 | 2000 | | 10 | 1000 | 0 |
| 50 R | Cz-M2 | 200 | 2000 | | 10 | 1000 | 0 |
| 50 R 2 | Cz-M2 | 200 | 2000 | | 10 | 1000 | 0 |
| 40 R | Cz-M2 | 200 | 2000 | | 10 | 1000 | 0 |
| 40 R 2 | Cz-M2 | 200 | 2000 | | 10 | 1000 | 0 |
| 30 R | Cz-M2 | 200 | 2000 | | 10 | 1000 | 0 |
| 30 R 2 | Cz-M2 | 200 | 2000 | | 10 | 1000 | 0 |
| 20 R | Cz-M2 | 200 | 2000 | | 10 | 1000 | 0 |
| 20 R 2 | Cz-M2 | 200 | 2000 | | 10 | 1000 | 0 |

**ABR**: ABR 2 8000Hz

2: Cz-M2

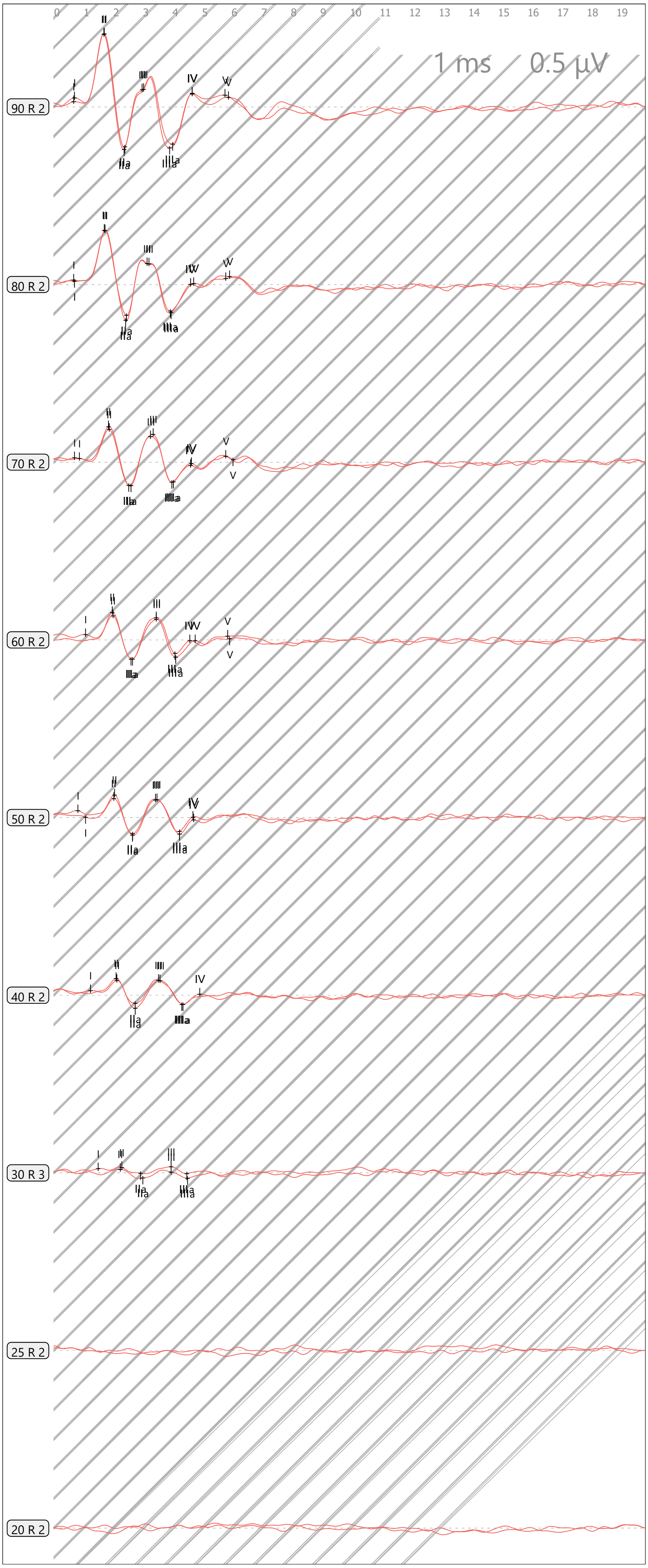
0 1 2 3 4 5 6 7 8 9 10 11 12 13 14 15 16 17 18 19
1 ms
0.5 µV
90 R 2
80 R 2
70 R 2
60 R 2
50 R 2
40 R 2
30 R 3
25 R 2
20 R 2

| | IV (ms) | V (ms) | I–III (ms) | I–V (ms) | III–V (ms) | |
|---|---|---|---|---|---|---|
| | 4.68 | 5.79 | 2.28 | 5.08 | 2.80 | |
| | 4.68 | 5.90 | 2.35 | 5.21 | 2.86 | |
| | 4.74 | 5.95 | 2.54 | 5.27 | 2.73 | |
| | 4.63 | 5.82 | 2.43 | 5.11 | 2.67 | |
| | 4.63 | 6.06 | 2.57 | 5.34 | 2.78 | |
| | 4.66 | 5.82 | 2.49 | 4.95 | 2.46 | |
| | 4.60 | 5.87 | | | 2.41 | |
| | 4.79 | 5.95 | 2.38 | 4.87 | 2.49 | |
| | 4.71 | | 2.35 | | | |
| | 4.74 | | 2.67 | | | |
| | 4.95 | | 2.35 | | | |
| | | | | | | |
| | | | 2.46 | | | |
| | | | | | | |

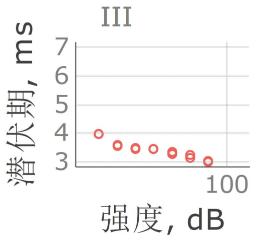


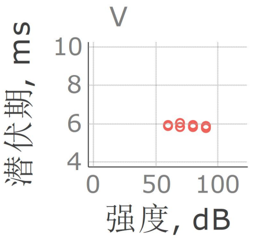


Trace parameters

| N | Electr. | HPF, Hz | LPF, Hz | 50 Hz | Rejection ±μV | Aver. | Reject |
|---|---|---|---|---|---|---|---|
| 90 R | Cz-M2 | 200 | 2000 | | 10 | 1000 | 0 |
| 90 R 2 | Cz-M2 | 200 | 2000 | | 10 | 1000 | 0 |
| 80 R | Cz-M2 | 200 | 2000 | | 10 | 1000 | 0 |
| 80 R 2 | Cz-M2 | 200 | 2000 | | 10 | 1000 | 0 |
| 70 R | Cz-M2 | 200 | 2000 | | 10 | 1000 | 0 |
| 70 R 2 | Cz-M2 | 200 | 2000 | | 10 | 1000 | 0 |
| 60 R | Cz-M2 | 200 | 2000 | | 10 | 1000 | 0 |
| 60 R 2 | Cz-M2 | 200 | 2000 | | 10 | 1000 | 0 |
| 50 R | Cz-M2 | 200 | 2000 | | 10 | 1000 | 0 |
| 50 R 2 | Cz-M2 | 200 | 2000 | | 10 | 1000 | 0 |
| 40 R | Cz-M2 | 200 | 2000 | | 10 | 1000 | 0 |
| 40 R 2 | Cz-M2 | 200 | 2000 | | 10 | 1000 | 0 |
| 30 R 2 | Cz-M2 | 200 | 2000 | | 10 | 1000 | 0 |
| 30 R 3 | Cz-M2 | 200 | 2000 | | 10 | 1000 | 0 |
| 25 R | Cz-M2 | 200 | 2000 | | 10 | 1000 | 0 |
| 25 R 2 | Cz-M2 | 200 | 2000 | | 10 | 1000 | 0 |
| 20 R | Cz-M2 | 200 | 2000 | | 10 | 1000 | 0 |

| | | | | | | | |
|---|---|---|---|---|---|---|---|
| | | | | | | | |
| 20 R 2 | Cz-M2 | 200 | 2000 | | 10 | 1000 | 0 |

**ECochG**: ECochG 1: Cz-M1

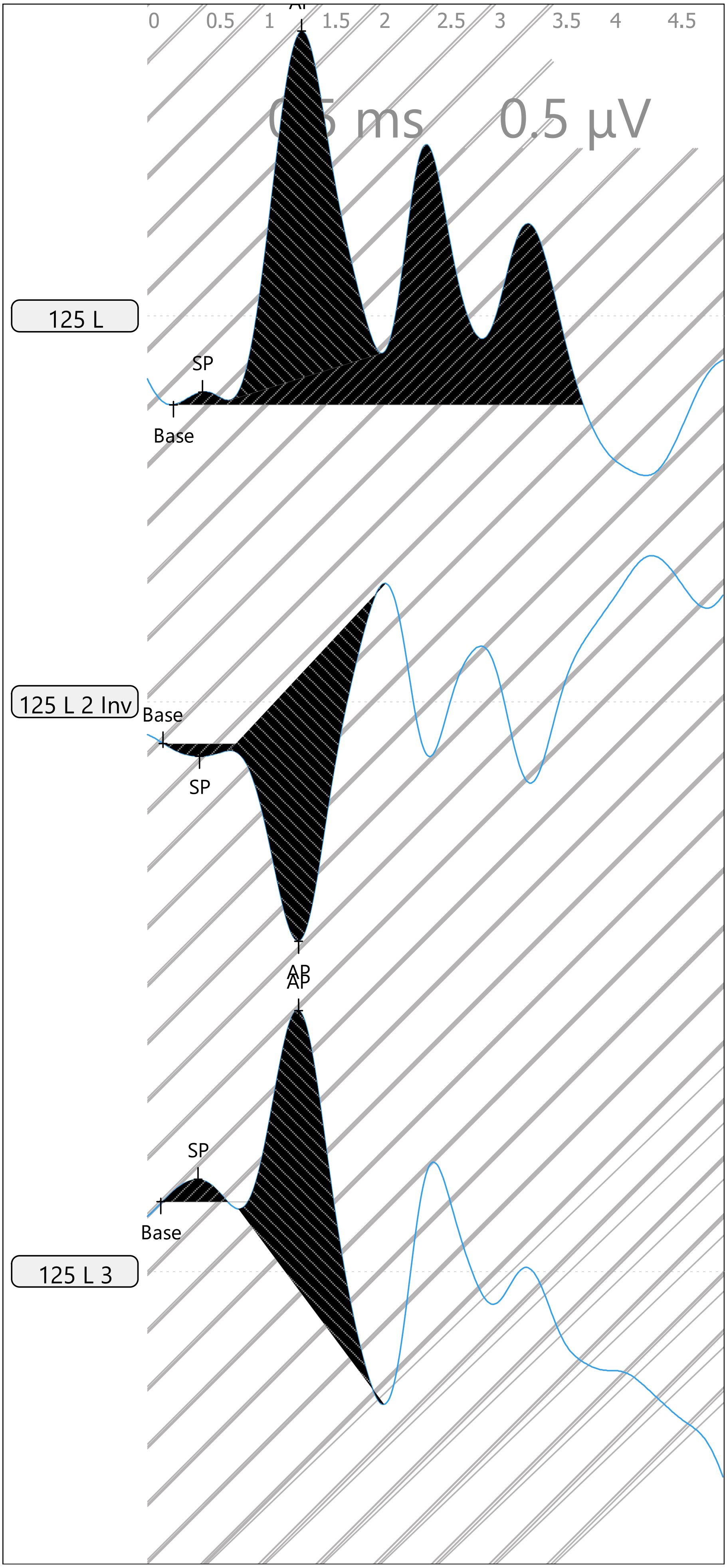
0
0.5
1
1.5
2
2.5
3
3.5
4
4.5
0.5 µV
125 L
SP
Base
125 L 2 Inv
Base
SP
AP
AP
SP
Base
125 L 3

&& (left ear

| N | Base (ms) | SP (ms) | AP (ms) | SP–Base (ms) | AP–Base (ms) | SP–Base (µV) | AP–Base (µV) | |
|---|---|---|---|---|---|---|---|---|
| 125 L | 0.22 | 0.48 | 1.34 | 0.25 | 1.11 | 0.11 | 3.24 | 0 |
| 125 L 2 Inv | 0.13 | 0.45 | 1.31 | 0.32 | 1.18 | 0.11 | 1.71 | 0 |
| 125 L 3 | 0.12 | 0.44 | 1.31 | 0.32 | 1.19 | 0.20 | 1.66 | 0 |

Trace parameters

| N | Electr. | HPF, Hz | LPF, Hz | 50 Hz | Rejection ±µV | Aver. | R |
|---|---|---|---|---|---|---|---|
| 125 L | Cz-M1 | 5 | 2000 | | 50 | 1500 | |
| 125 L 2 Inv | Cz-M1 | 5 | 2000 | | 50 | 1500 | |
| 125 L 3 | Cz-M1 | 5 | 2000 | | 50 | 1500 | |

**ECochG**: ECochG

2: Fpz-M2

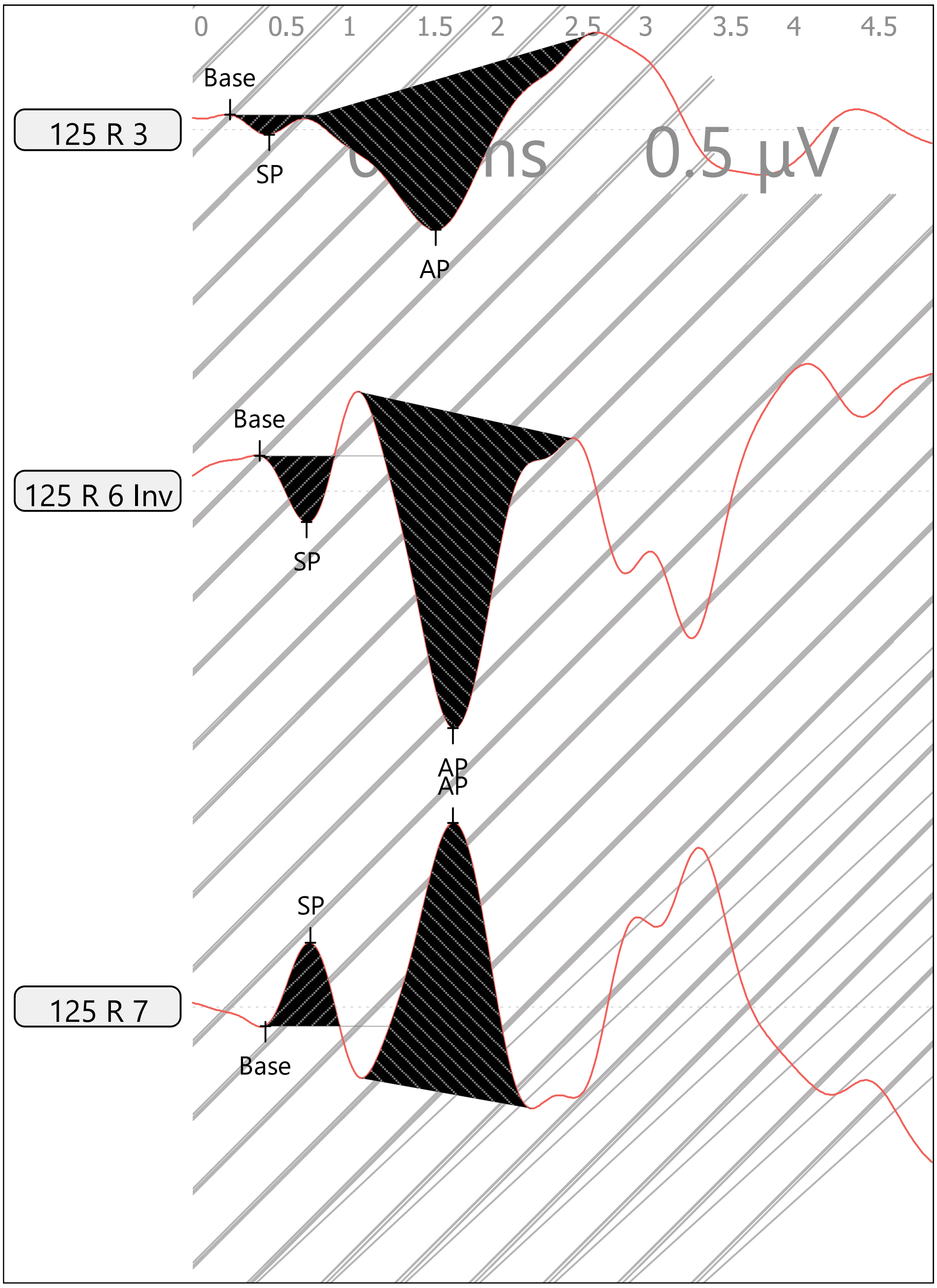


&& (right) ear

| N | Base (ms) | SP (ms) | AP (ms) | SP–Base (ms) | AP–Base (ms) | SP–Base (µV) | AP–Base (µV) | |
|---|---|---|---|---|---|---|---|---|
| 125 R 3 | 0.25 | 0.52 | 1.64 | 0.26 | 1.39 | 0.13 | 0.78 | 0 |
| 125 R 6 Inv | 0.45 | 0.77 | 1.76 | 0.32 | 1.31 | 0.45 | 1.84 | 0 |

| | | | | | | | | |
|---|---|---|---|---|---|---|---|---|
| 125 R 7 | 0.49 | 0.79 | 1.76 | 0.30 | 1.27 | 0.56 | 1.37 | 0[illegible] |

Trace parameters

| N | Electr. | HPF, Hz | LPF, Hz | 50 Hz | Rejection ±μV | Aver. | R[illegible] |
|---|---|---|---|---|---|---|---|
| 125 R 3 | Fpz-M2 | 5 | 2000 | | 50 | 1500 | |
| 125 R 6 Inv | Fpz-M2 | 5 | 2000 | | 50 | 1459 | |
| 125 R 7 | Fpz-M2 | 5 | 2000 | | 50 | 1500 | |

**CONCLUSION:**

**Doctor:**
