# Supplementary material for: Exposure to high-altitude hypobaric hypoxic environment induces low-frequency hearing loss in C57BL/6J mice: Mediated by slowing down the postsynaptic electrical signal transmission speed in the cochlear-inferior colliculus auditory signaling pathway
Source: PLoS One. 2026 Mar 11;21(3):e0342321. doi: 10.1371/journal.pone.0342321 (PMC12978441; doi:10.1371/journal.pone.0342321)
Supplement: S1 File — (ZIP) [file pone.0342321.s001.zip › 2025.06.15-7d-01.pdf]

Exam report

Patient: 2025.06.15-7d-01- (-)
Date: June 15, 2025

ABR: ABR 2 CLICK
1: Cz-M1

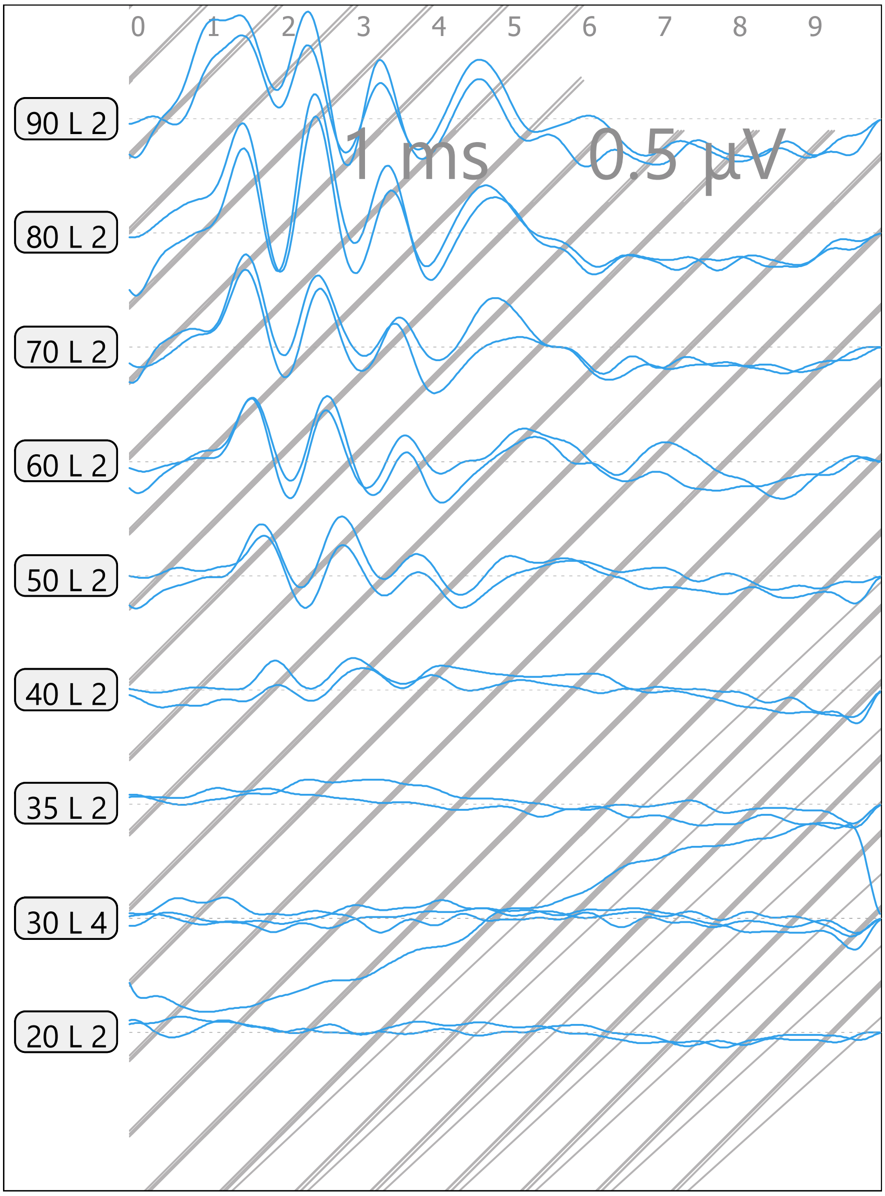

Trace parameters

N	Electr.	HPF, Hz	LPF, Hz	50 Hz	Rejection ±µV	Aver.	Reject.
90 L	Cz-M1	100	2000		10	1000	0
90 L 2	Cz-M1	100	2000		10	1000	0
80 L	Cz-M1	100	2000		10	1000	0
80 L 2	Cz-M1	100	2000		10	1000	0
70 L	Cz-M1	100	2000		10	1000	0
70 L 2	Cz-M1	100	2000		10	1000	0
60 L	Cz-M1	100	2000		10	1000	0
60 L 2	Cz-M1	100	2000		10	1000	0
50 L	Cz-M1	100	2000		10	1000	0
50 L 2	Cz-M1	100	2000		10	1000	0
40 L	Cz-M1	100	2000		10	1000	0
40 L 2	Cz-M1	100	2000		10	1000	0

35 L	Cz-M1	100	2000		10	1000	0
35 L 2	Cz-M1	100	2000		10	1000	0
30 L	Cz-M1	100	2000		10	1000	0
30 L 2	Cz-M1	100	2000		10	1000	0
30 L 3	Cz-M1	100	2000		10	1000	0
30 L 4	Cz-M1	100	2000		10	1000	0
20 L	Cz-M1	100	2000		10	1000	0
20 L 2	Cz-M1	100	2000		10	1000	0

ABR: ABR 2 tone burst 4000Hz 1
: Cz-M1

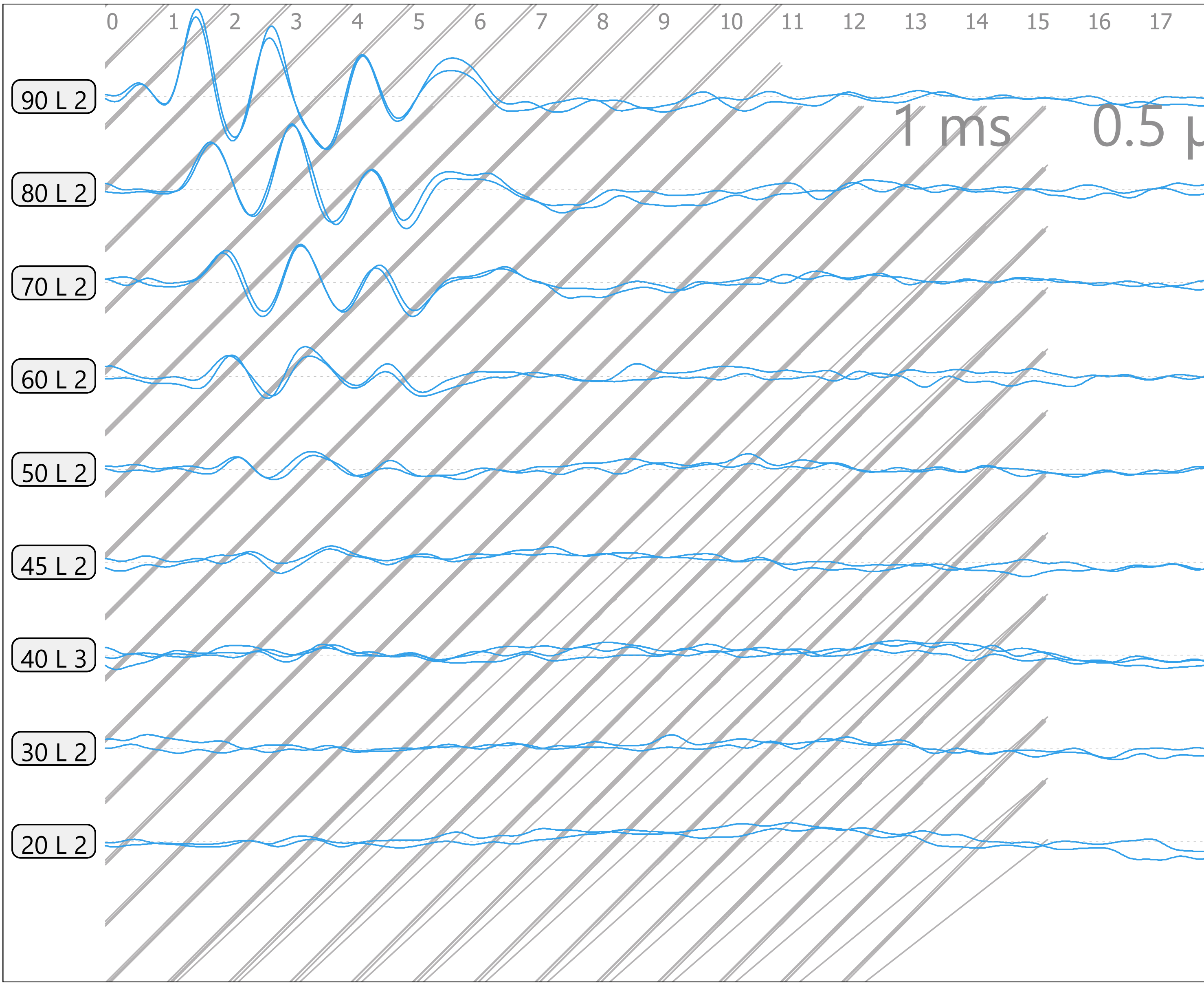

Trace parameters

N	Electr.	HPF, Hz	LPF, Hz	50 Hz	Rejection ±µV	Aver.	Reject.
90 L	Cz-M1	200	2000		10	1000	0
90 L 2	Cz-M1	200	2000		10	1000	0
80 L	Cz-M1	200	2000		10	1000	0
80 L 2	Cz-M1	200	2000		10	1000	0
70 L	Cz-M1	200	2000		10	1000	0

70 L 2	Cz-M1	200	2000		10	1000	0
60 L	Cz-M1	200	2000		10	1000	0
60 L 2	Cz-M1	200	2000		10	1000	0
50 L	Cz-M1	200	2000		10	1000	0
50 L 2	Cz-M1	200	2000		10	1000	0
45 L	Cz-M1	200	2000		10	1000	0
45 L 2	Cz-M1	200	2000		10	1000	0
40 L	Cz-M1	200	2000		10	1000	0
40 L 2	Cz-M1	200	2000		10	1000	0
40 L 3	Cz-M1	200	2000		10	1000	0
30 L	Cz-M1	200	2000		10	1000	0
30 L 2	Cz-M1	200	2000		10	1000	0
20 L	Cz-M1	200	2000		10	1000	0
20 L 2	Cz-M1	200	2000		10	1000	0

ABR: ABR 2 8000Hz 1: Cz-M1

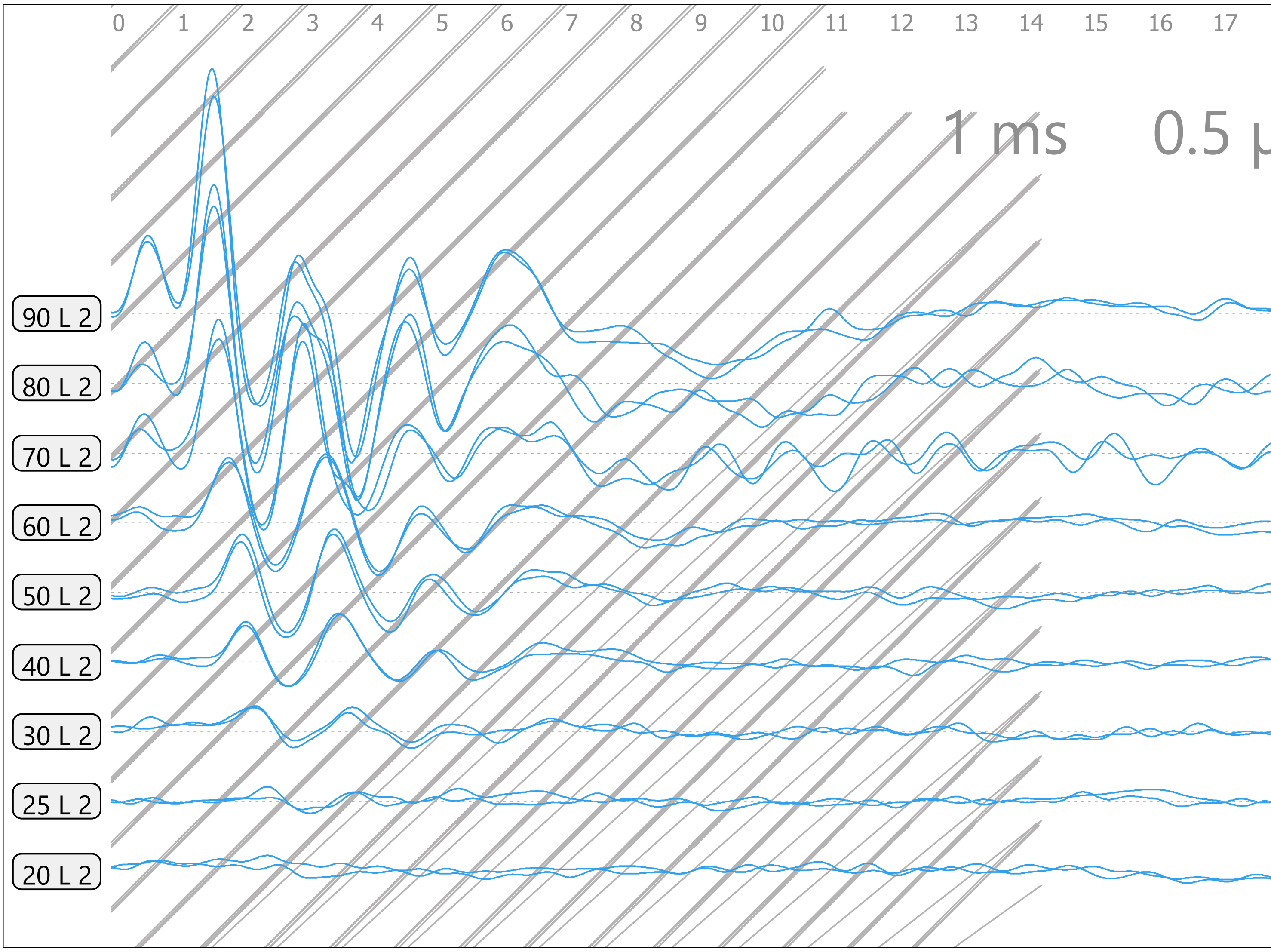

Trace parameters

N	Electr.	HPF, Hz	LPF, Hz	50 Hz	Rejection ±μV	Aver.	Reject.
90 L	Cz-M1	200	2000		10	1000	0
90 L 2	Cz-M1	200	2000		10	1000	0
80 L	Cz-M1	200	2000		10	1000	0
80 L 2	Cz-M1	200	2000		10	1000	0
70 L	Cz-M1	200	2000		10	1000	0
70 L 2	Cz-M1	200	2000		10	1000	0
60 L	Cz-M1	200	2000		10	1000	0
60 L 2	Cz-M1	200	2000		10	1000	0
50 L	Cz-M1	200	2000		10	1000	0
50 L 2	Cz-M1	200	2000		10	1000	0
40 L	Cz-M1	200	2000		10	1000	0
40 L 2	Cz-M1	200	2000		10	1000	0
30 L	Cz-M1	200	2000		10	1000	0
30 L 2	Cz-M1	200	2000		10	1000	0

25 L	Cz-M1	200	2000		10	1000	0
25 L 2	Cz-M1	200	2000		10	1000	0
20 L	Cz-M1	200	2000		10	1000	0
20 L 2	Cz-M1	200	2000		10	1000	0

ABR: ABR 2 CLICK
2: Fpz-M2

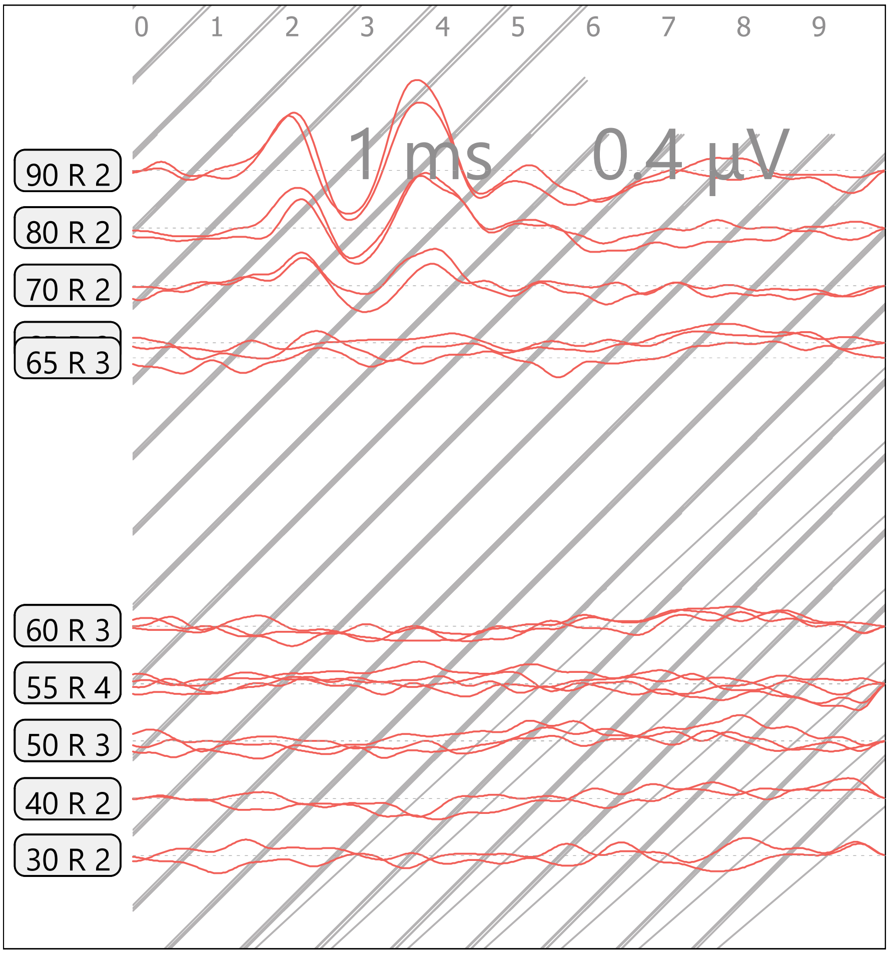

Trace parameters

N	Electr.	HPF, Hz	LPF, Hz	50 Hz	Rejection ±µV	Aver.	Reject
90 R	Fpz-M2	100	2000		10	1000	0
90 R 2	Fpz-M2	100	2000		10	1000	0
80 R	Fpz-M2	100	2000		10	1000	0
80 R 2	Fpz-M2	100	2000		10	1000	0
70 R	Fpz-M2	100	2000		10	1000	0
70 R 2	Fpz-M2	100	2000		10	1000	0
65 R	Fpz-M2	100	2000		10	1000	0
65 R 2	Fpz-M2	100	2000		10	1000	0
65 R 3	Fpz-M2	100	2000		10	1000	0
60 R	Fpz-M2	100	2000		10	1000	0
60 R 2	Fpz-M2	100	2000		10	1000	0
60 R 3	Fpz-M2	100	2000		10	1000	0
55 R	Fpz-M2	100	2000		10	1000	0
55 R 2	Fpz-M2	100	2000		10	1000	0
55 R 3	Fpz-M2	100	2000		10	1000	0
55 R 4	Fpz-M2	100	2000		10	1000	0

50 R	Fpz-M2	100	2000		10	1000	0
50 R 2	Fpz-M2	100	2000		10	1000	0
50 R 3	Fpz-M2	100	2000		10	1000	0
40 R	Fpz-M2	100	2000		10	1000	0
40 R 2	Fpz-M2	100	2000		10	1000	0
30 R	Fpz-M2	100	2000		10	1000	0
30 R 2	Fpz-M2	100	2000		10	1000	0

ABR: ABR 2 4000Hz 2: Fpz-M2

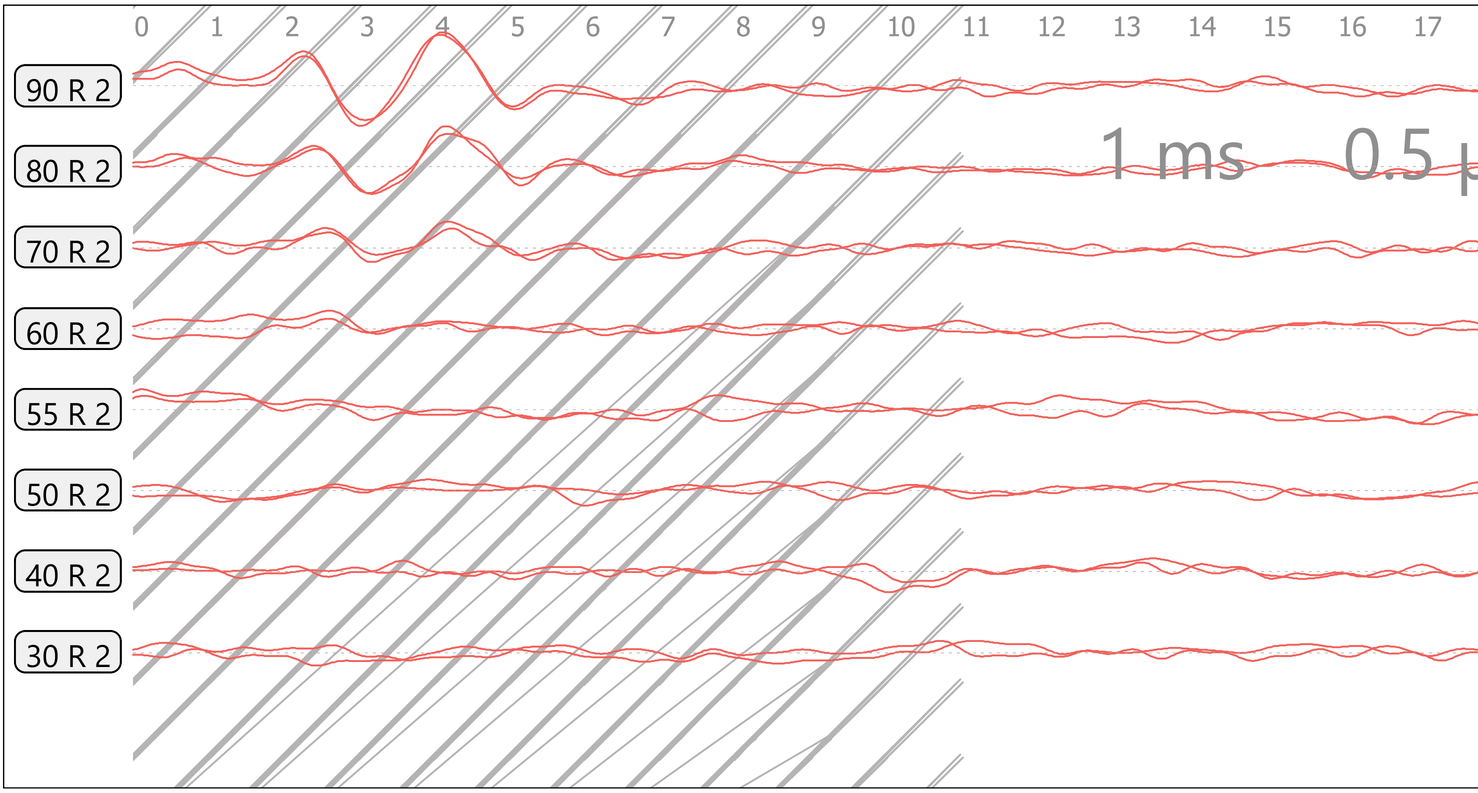

Trace parameters

N	Electr.	HPF, Hz	LPF, Hz	50 Hz	Rejection ±μV	Aver.	Reject
90 R	Fpz-M2	200	2000		10	1000	0
90 R 2	Fpz-M2	200	2000		10	1000	0
80 R	Fpz-M2	200	2000		10	1000	0
80 R 2	Fpz-M2	200	2000		10	1000	0
70 R	Fpz-M2	200	2000		10	1000	0
70 R 2	Fpz-M2	200	2000		10	1000	0
60 R	Fpz-M2	200	2000		10	1000	0
60 R 2	Fpz-M2	200	2000		10	1000	0
55 R	Fpz-M2	200	2000		10	1000	0
55 R 2	Fpz-M2	200	2000		10	1000	0
50 R	Fpz-M2	200	2000		10	1000	0
50 R 2	Fpz-M2	200	2000		10	1000	0
40 R	Fpz-M2	200	2000		10	1000	0

40 R 2	Fpz-M2	200	2000		10	1000	0
30 R	Fpz-M2	200	2000		10	1000	0
30 R 2	Fpz-M2	200	2000		10	1000	0

ABR: ABR 2 8000Hz 2: Fpz-M2

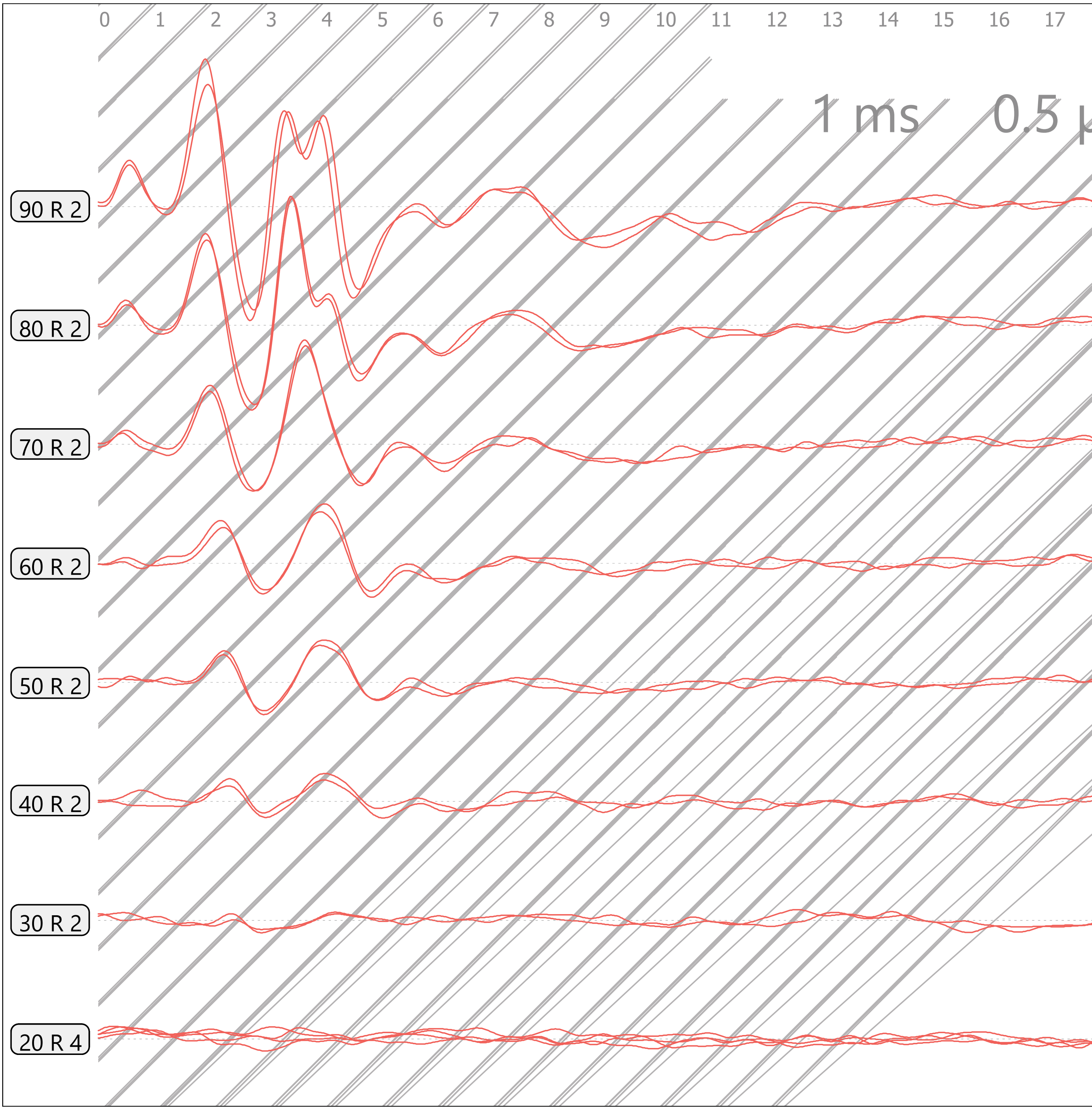

Trace parameters

N	Electr.	HPF, Hz	LPF, Hz	50 Hz	Rejection ±µV	Aver.	Reject
90 R	Fpz-M2	200	2000		10	1000	0
90 R 2	Fpz-M2	200	2000		10	1000	0

80 R	Fpz-M2	200	2000		10	1000	0
80 R 2	Fpz-M2	200	2000		10	1000	0
70 R	Fpz-M2	200	2000		10	1000	0
70 R 2	Fpz-M2	200	2000		10	1000	0
60 R	Fpz-M2	200	2000		10	1000	0
60 R 2	Fpz-M2	200	2000		10	1000	0
50 R	Fpz-M2	200	2000		10	1000	0
50 R 2	Fpz-M2	200	2000		10	1000	0
40 R	Fpz-M2	200	2000		10	1000	0
40 R 2	Fpz-M2	200	2000		10	1000	0
30 R	Fpz-M2	200	2000		10	1000	0
30 R 2	Fpz-M2	200	2000		10	1000	0
20 R	Fpz-M2	200	2000		10	1000	0
20 R 2	Fpz-M2	200	2000		10	1000	0
20 R 3	Fpz-M2	200	2000		10	1000	0
20 R 4	Fpz-M2	200	2000		10	1000	0

ECochG: ECochG 1: Cz-M1

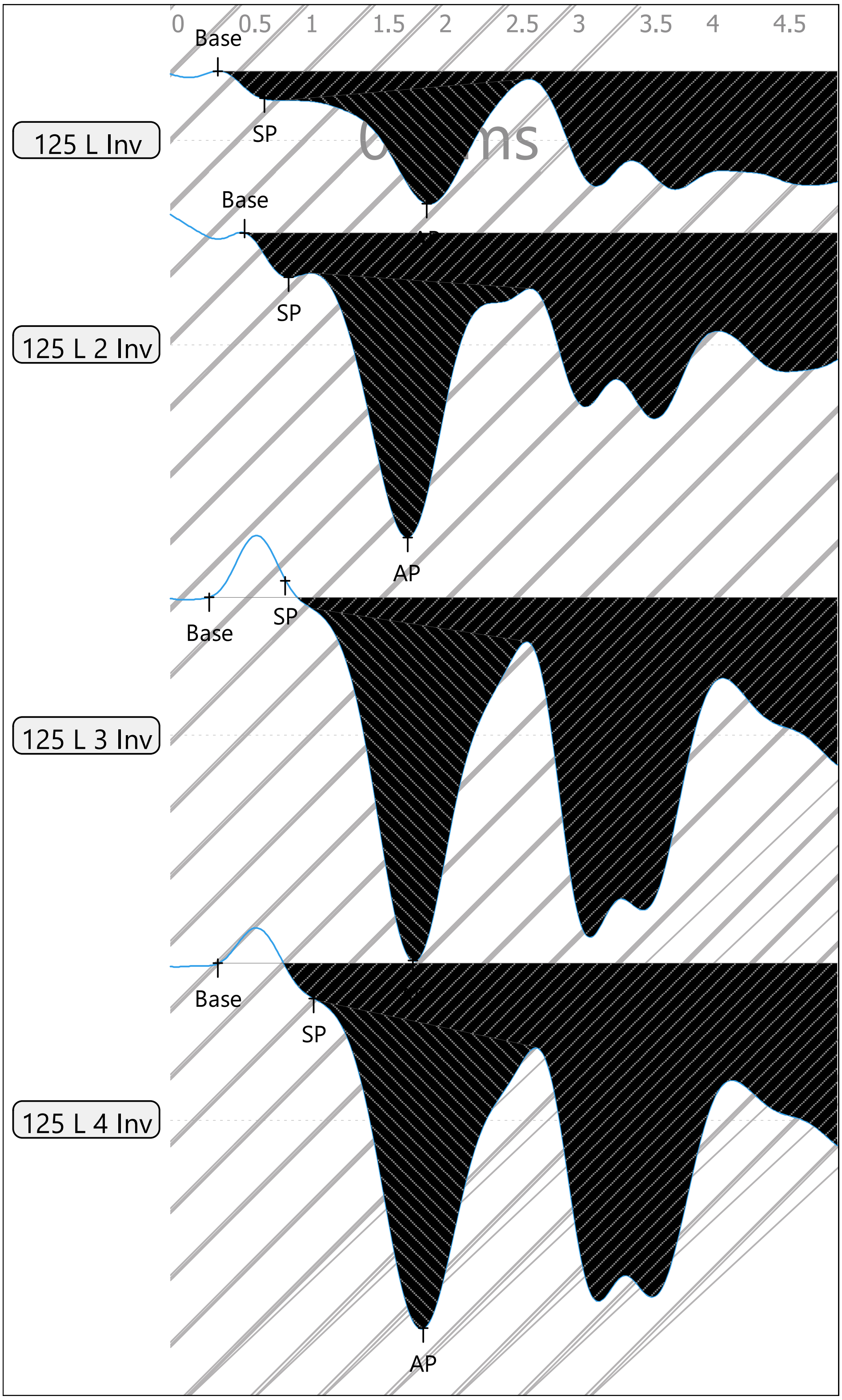

&& (tone burst(left ear)

N	Base (ms)	SP (ms)	AP (ms)	SP–Base (ms)	AP–Base (ms)	SP–Base (µV)	AP–Base (µV)	
125 L Inv	0.36	0.70	1.92	0.34	1.56	0.42	1.98	0
125 L 2 Inv	0.56	0.89	1.77	0.33	1.22	0.67	4.55	0
125 L 3 Inv	0.29	0.86	1.81	0.57	1.52	0.25	5.43	0
125 L 4 Inv	0.36	1.07	1.89	0.71	1.53	0.53	5.46	0

Trace parameters

N	Electr.	HPF, Hz	LPF, Hz	50 Hz	Rejection ±µV	Aver.	R
125 L Inv	Cz-M1	5	2000		50	1500	
125 L 2 Inv	Cz-M1	5	2000		50	700	

125 L 3 Inv	Cz-M1	5	2000		50	1500	
125 L 4 Inv	Cz-M1	5	2000		50	1500	

ECochG: ECochG
2: Fpz-M2

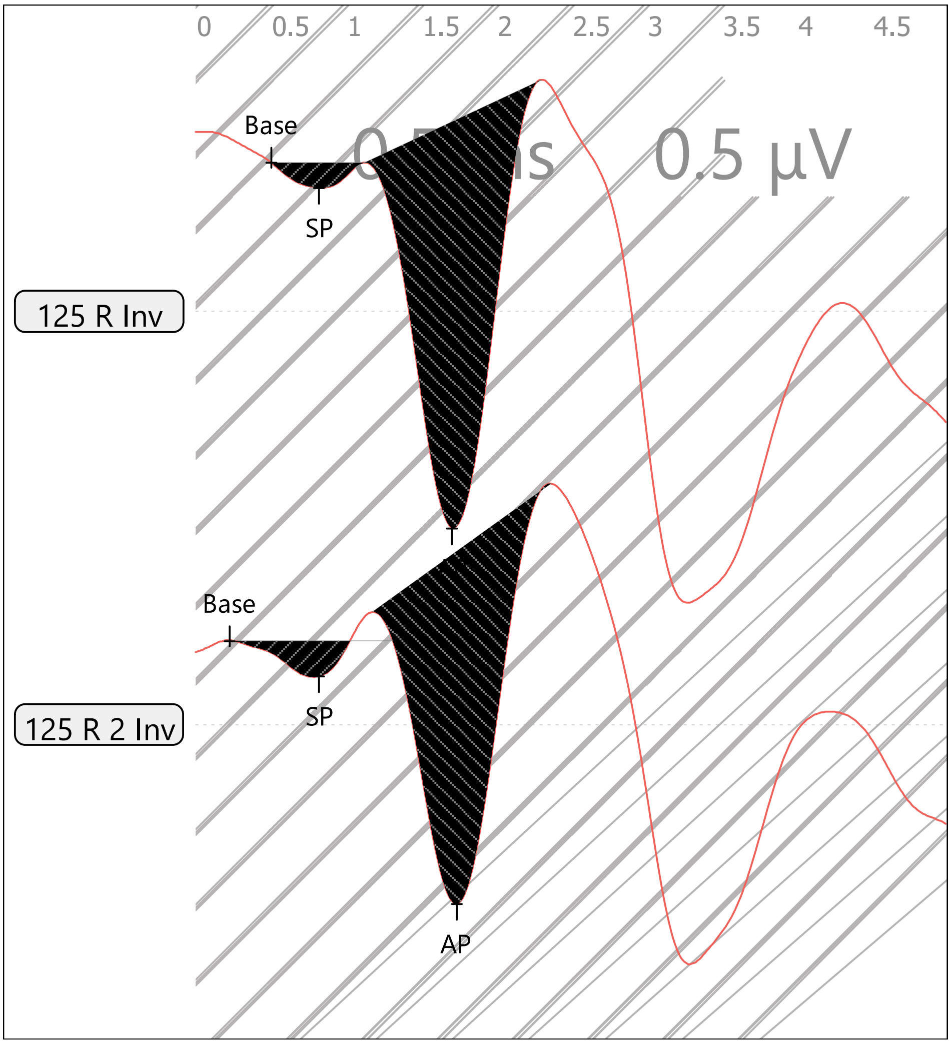

&& (left ear)

N	Base (ms)	SP (ms)	AP (ms)	SP–Base (ms)	AP–Base (ms)	SP–Base (µV)	AP–Base (µV)	
125 R Inv	0.50	0.82	1.71	0.32	1.20	0.17	2.43	0
125 R 2 Inv	0.22	0.82	1.73	0.60	1.51	0.24	1.75	0

Trace parameters

N	Electr.	HPF, Hz	LPF, Hz	50 Hz	Rejection ±µV	Aver.	R
125 R Inv	Fpz-M2	5	2000		50	1500	
125 R 2 Inv	Fpz-M2	5	2000		50	1500	

CONCLUSION:

Doctor:
