# Supplementary material for: Exposure to high-altitude hypobaric hypoxic environment induces low-frequency hearing loss in C57BL/6J mice: Mediated by slowing down the postsynaptic electrical signal transmission speed in the cochlear-inferior colliculus auditory signaling pathway
Source: PLoS One. 2026 Mar 11;21(3):e0342321. doi: 10.1371/journal.pone.0342321 (PMC12978441; doi:10.1371/journal.pone.0342321)
Supplement: S1 File — (ZIP) [file pone.0342321.s001.zip › 2025.06.16-20d-01.pdf]

Exam report

Patient: 2025.06.16-20d-01, - (-)
Date: June 16, 2025

ABR: ABR 2 CLICK
1: Cz-M1

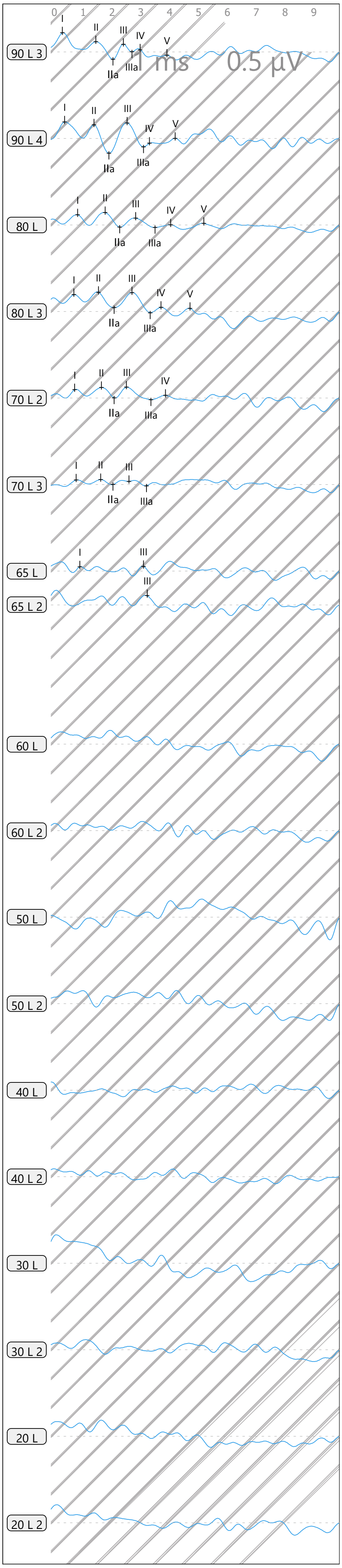
0 1 2 3 4 5 6 7 8 9
1 ms
0.5 µV
90 L 3
90 L 4
80 L
80 L 3
70 L 2
70 L 3
65 L
65 L 2
60 L
60 L 2
50 L
50 L 2
40 L
40 L 2
30 L
30 L 2
20 L
20 L 2
I
II
IIa
III
IIIa
IV
V

latency && amplitude (left ear

N	I (ms)	II (ms)	III (ms)	IV (ms)	V (ms)
90 L 3	0.40	1.56	2.51	3.10	4.02
90 L 4	0.48	1.48	2.65	3.41	4.31
80 L	0.93	1.88	2.94	4.15	5.29
80 L 3	0.79	1.64	2.80	3.81	4.82
70 L 2	0.82	1.75	2.62	3.97	
70 L 3	0.87	1.72	2.70		
65 L	1.01		3.20		
65 L 2			3.33		

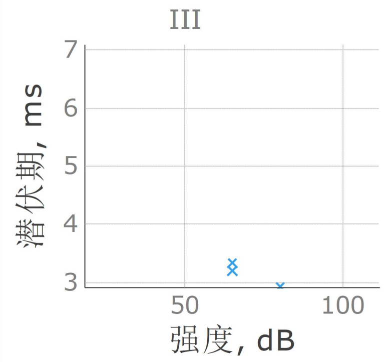

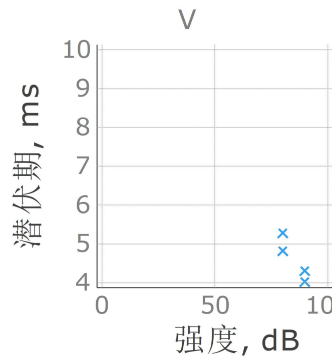

Trace parameters

N	Electr.	HPF, Hz	LPF, Hz	50 Hz	Rejection ±µV	Aver.	Reject.
90 L 3	Cz-M1	100	2000		10	1000	0
90 L 4	Cz-M1	100	2000		10	1000	0
80 L	Cz-M1	100	2000		10	1000	0
80 L 3	Cz-M1	100	2000		10	1000	0
70 L 2	Cz-M1	100	2000		10	1000	0
70 L 3	Cz-M1	100	2000		10	1000	0
65 L	Cz-M1	100	2000		10	1000	0
65 L 2	Cz-M1	100	2000		10	1000	0
60 L	Cz-M1	100	2000		10	1000	0
60 L 2	Cz-M1	100	2000		10	1000	0
50 L	Cz-M1	100	2000		10	1000	0
50 L 2	Cz-M1	100	2000		10	1000	0
40 L	Cz-M1	100	2000		10	1000	0
40 L 2	Cz-M1	100	2000		10	1000	0
30 L	Cz-M1	100	2000		10	1000	0
30 L 2	Cz-M1	100	2000		10	1000	0
20 L	Cz-M1	100	2000		10	1000	0
20 L 2	Cz-M1	100	2000		10	1000	0

ABR: ABR 2 tone burst 4000Hz 1
: Cz-M1

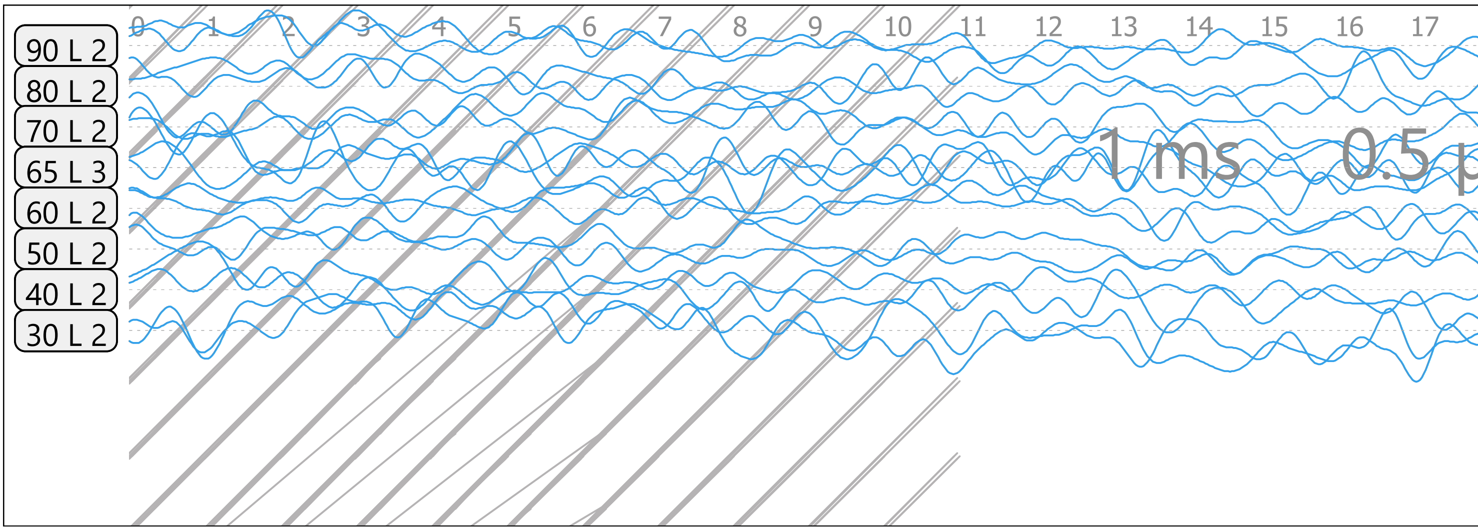

Trace parameters

N	Electr.	HPF, Hz	LPF, Hz	50 Hz	Rejection ±μV	Aver.	Reject.
90 L	Cz-M1	200	2000		10	1000	0
90 L 2	Cz-M1	200	2000		10	1000	0
80 L	Cz-M1	200	2000		10	1000	0
80 L 2	Cz-M1	200	2000		10	1000	0
70 L	Cz-M1	200	2000		10	1000	0
70 L 2	Cz-M1	200	2000		10	1000	0
65 L	Cz-M1	200	2000		10	1000	0
65 L 2	Cz-M1	200	2000		10	1000	0
65 L 3	Cz-M1	200	2000		10	1000	0
60 L	Cz-M1	200	2000		10	1000	0
60 L 2	Cz-M1	200	2000		10	1000	0
50 L	Cz-M1	200	2000		10	1000	0
50 L 2	Cz-M1	200	2000		10	1000	0
40 L	Cz-M1	200	2000		10	1000	0
40 L 2	Cz-M1	200	2000		10	1000	0
30 L	Cz-M1	200	2000		10	1000	0
30 L 2	Cz-M1	200	2000		10	1000	0

ABR: ABR 2 8000Hz 1: Cz-M1

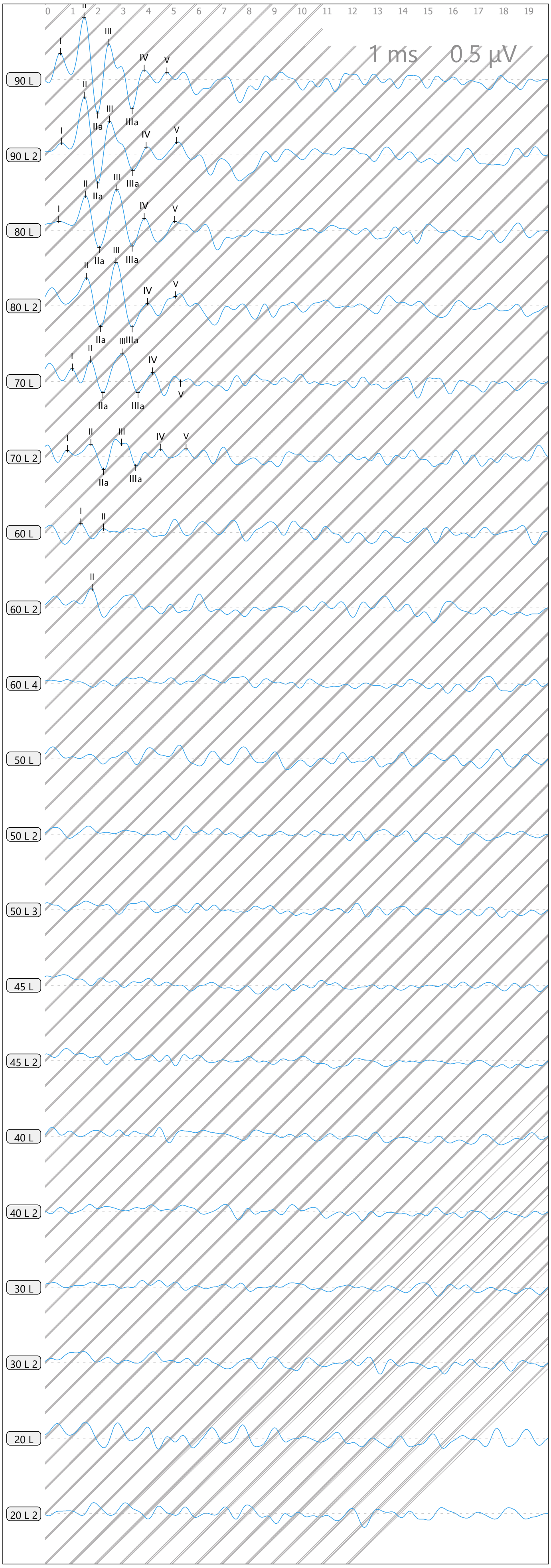

0 1 2 3 4 5 6 7 8 9 10 11 12 13 14 15 16 17 18 19
1 ms
0.5 µV
90 L
90 L 2
80 L
80 L 2
70 L
70 L 2
60 L
60 L 2
60 L 4
50 L
50 L 2
50 L 3
45 L
45 L 2
40 L
40 L 2
30 L
30 L 2
20 L
20 L 2

&& (left ear

N	I (ms)	II (ms)	III (ms)	IV (ms)	V (ms)
90 L	0.61	1.56	2.51	3.94	4.84
90 L 2	0.66	1.59	2.57	4.02	5.24
80 L	0.56	1.61	2.86	3.94	5.16
80 L 2		1.64	2.83	4.07	5.19
70 L	1.08	1.80	3.07	4.29	5.40
70 L 2	0.90	1.83	3.04	4.60	5.61
60 L	1.43	2.33			
60 L 2		1.88			

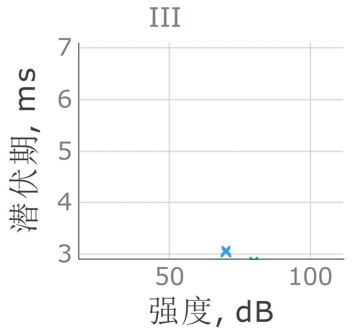

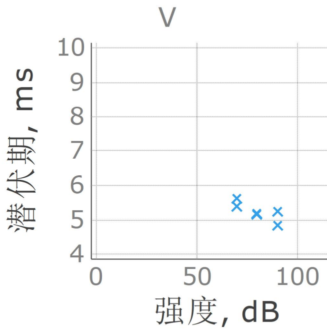

Trace parameters

N	Electr.	HPF, Hz	LPF, Hz	50 Hz	Rejection ±µV	Aver.	Reject.
90 L	Cz-M1	200	2000		10	1000	0
90 L 2	Cz-M1	200	2000		10	1000	0
80 L	Cz-M1	200	2000		10	1000	0
80 L 2	Cz-M1	200	2000		10	1000	0
70 L	Cz-M1	200	2000		10	1000	0
70 L 2	Cz-M1	200	2000		10	1000	0
60 L	Cz-M1	200	2000		10	1000	0
60 L 2	Cz-M1	200	2000		10	1000	0
60 L 4	Cz-M1	200	2000		10	1000	0
50 L	Cz-M1	200	2000		10	1000	0
50 L 2	Cz-M1	200	2000		10	1000	0
50 L 3	Cz-M1	200	2000		10	1000	0
45 L	Cz-M1	200	2000		10	1000	0
45 L 2	Cz-M1	200	2000		10	1000	0
40 L	Cz-M1	200	2000		10	1000	0
40 L 2	Cz-M1	200	2000		10	1000	0
30 L	Cz-M1	200	2000		10	1000	0
30 L 2	Cz-M1	200	2000		10	1000	0
20 L	Cz-M1	200	2000		10	1000	0

20 L 2	Cz-M1	200	2000		10	1000	0

ECochG: ECochG

1: Fpz-M1

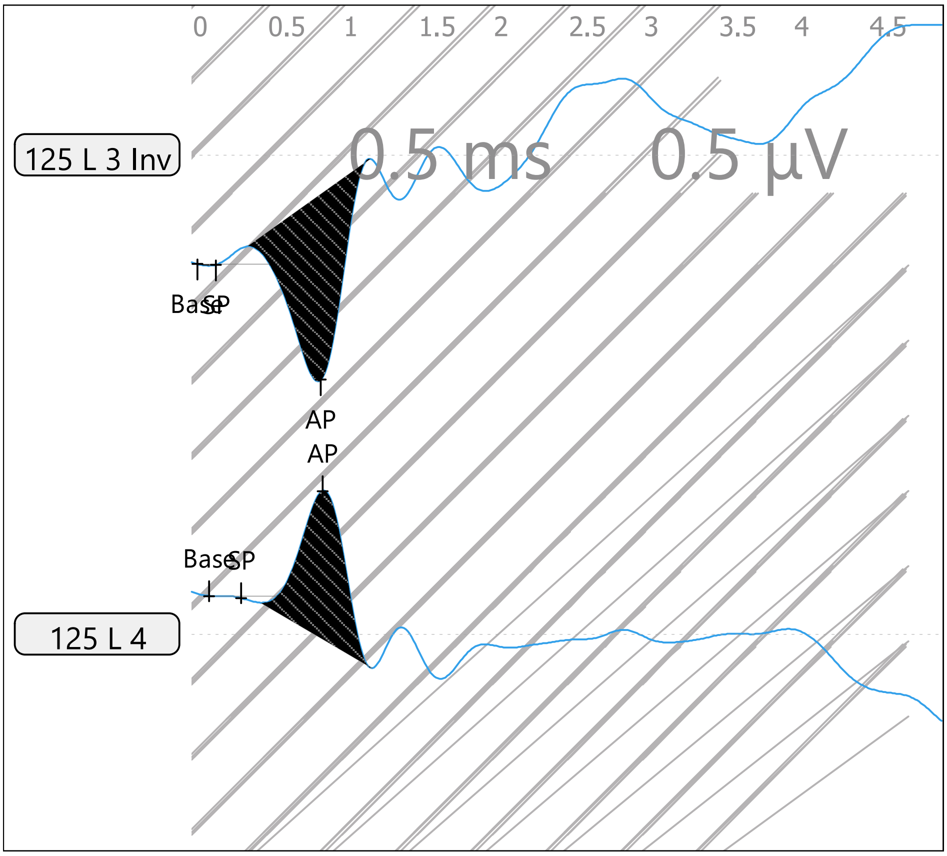

&& (left ear

N	Base (ms)	SP (ms)	AP (ms)	SP–Base (ms)	AP–Base (ms)	SP–Base (µV)	AP–Base (µV)	
125 L 3 Inv	0.04	0.16	0.86	0.12	0.82	0.00	0.77	0
125 L 4	0.12	0.33	0.87	0.21	0.75	0.01	0.70	0

Trace parameters

N	Electr.	HPF, Hz	LPF, Hz	50 Hz	Rejection ±µV	Aver.	R
125 L 3 Inv	Fpz-M1	5	2000		50	1500	
125 L 4	Fpz-M1	5	2000		50	1500	

CONCLUSION:

Doctor:
