# Supplementary material for: Exposure to high-altitude hypobaric hypoxic environment induces low-frequency hearing loss in C57BL/6J mice: Mediated by slowing down the postsynaptic electrical signal transmission speed in the cochlear-inferior colliculus auditory signaling pathway
Source: PLoS One. 2026 Mar 11;21(3):e0342321. doi: 10.1371/journal.pone.0342321 (PMC12978441; doi:10.1371/journal.pone.0342321)
Supplement: S1 File — (ZIP) [file pone.0342321.s001.zip › 2025.06.16-20d-03.pdf]

Exam report

Patient: 2025.06.16-20d-03**,** - (-)
Date: June 18, 2025

ABR: ABR 2 CLICK
1: Cz-M1

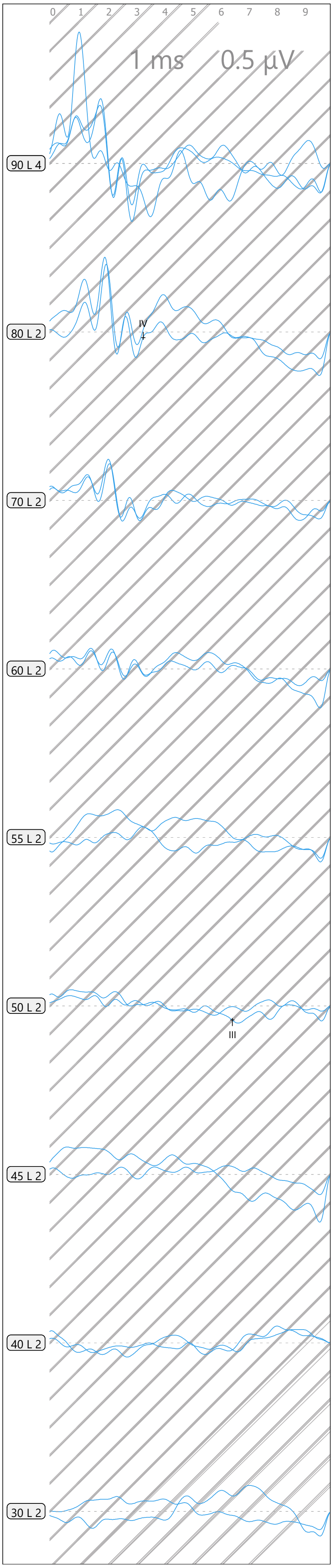
0 1 2 3 4 5 6 7 8 9
1 ms
0.5 µV
90 L 4
80 L 2
IV
70 L 2
60 L 2
55 L 2
50 L 2
III
45 L 2
40 L 2
30 L 2

latency&& amplitude (left ear

N	I (ms)	II (ms)	III (ms)	IV (ms)	(
80 L				3.33	
50 L			6.51		

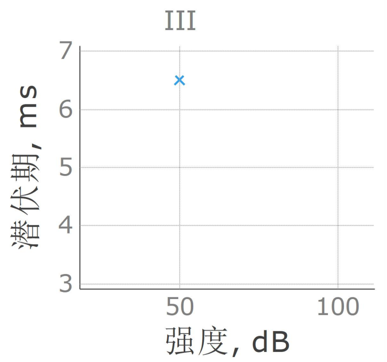

Trace parameters

N	Electr.	HPF, Hz	LPF, Hz	50 Hz	Rejection ±μV	Aver.	Reject.
90 L 2	Cz-M1	100	2000		10	1000	0
90 L 3	Cz-M1	100	2000		10	1000	0
90 L 4	Cz-M1	100	2000		10	1000	0
80 L	Cz-M1	100	2000		10	1000	0
80 L 2	Cz-M1	100	2000		10	1000	0
70 L	Cz-M1	100	2000		10	1000	0
70 L 2	Cz-M1	100	2000		10	1000	0
60 L	Cz-M1	100	2000		10	1000	0
60 L 2	Cz-M1	100	2000		10	1000	0
55 L	Cz-M1	100	2000		10	1000	0
55 L 2	Cz-M1	100	2000		10	1000	1
50 L	Cz-M1	100	2000		10	1000	0
50 L 2	Cz-M1	100	2000		10	1000	0
45 L	Cz-M1	100	2000		10	1000	0
45 L 2	Cz-M1	100	2000		10	1000	0
40 L	Cz-M1	100	2000		10	1000	0
40 L 2	Cz-M1	100	2000		10	1000	0
30 L	Cz-M1	100	2000		10	1000	0
30 L 2	Cz-M1	100	2000		10	1000	0

ABR: ABR 2 tone burst 4000Hz 1
: Cz-M1

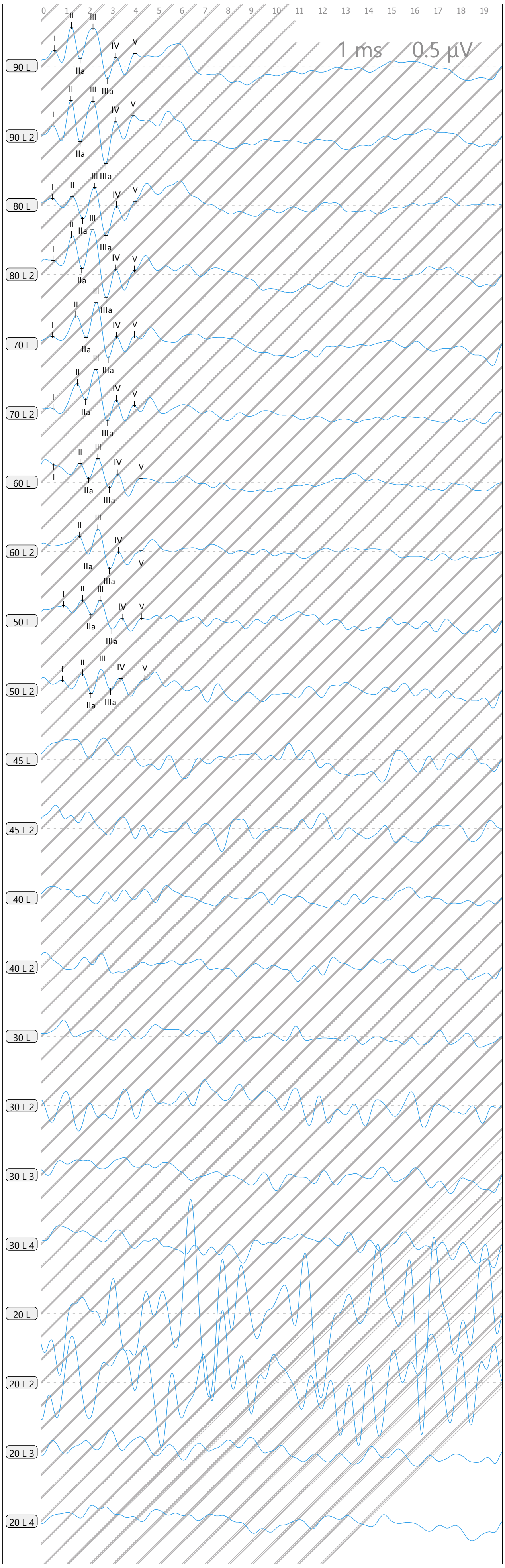
0 1 2 3 4 5 6 7 8 9 10 11 12 13 14 15 16 17 18 19
1 ms
0.5 µV
90 L
90 L 2
80 L
80 L 2
70 L
70 L 2
60 L
60 L 2
50 L
50 L 2
45 L
45 L 2
40 L
40 L 2
30 L
30 L 2
30 L 3
30 L 4
20 L
20 L 2
20 L 3
20 L 4
I
II
IIa
III
IIIa
IV
V

&&

N	I (ms)	II (ms)	III (ms)	IV (ms)	V (ms)
90 L	0.58	1.32	2.25	3.23	4.07
90 L 2	0.53	1.30	2.25	3.23	4.00
80 L	0.50	1.35	2.33	3.28	4.07
80 L 2	0.53	1.32	2.22	3.25	4.05
70 L	0.50	1.51	2.38	3.28	4.05
70 L 2	0.53	1.59	2.38	3.28	4.05
60 L	0.56	1.69	2.46	3.33	4.34
60 L 2		1.67	2.46	3.36	4.34
50 L	0.98	1.80	2.57	3.52	4.37
50 L 2	0.93	1.80	2.65	3.47	4.50

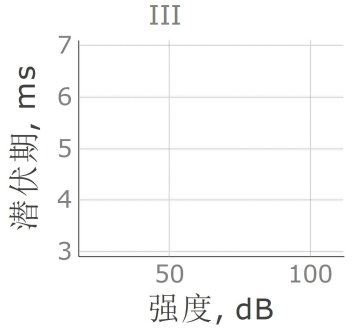

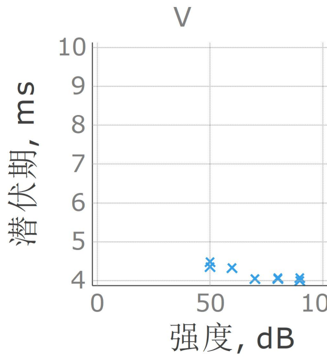

Trace parameters

N	Electr.	HPF, Hz	LPF, Hz	50 Hz	Rejection ±μV	Aver.	Reject.
90 L	Cz-M1	200	2000		10	1000	0
90 L 2	Cz-M1	200	2000		10	1000	0
80 L	Cz-M1	200	2000		10	1000	0
80 L 2	Cz-M1	200	2000		10	1000	0
70 L	Cz-M1	200	2000		10	1000	0
70 L 2	Cz-M1	200	2000		10	1000	0
60 L	Cz-M1	200	2000		10	1000	0
60 L 2	Cz-M1	200	2000		10	1001	0
50 L	Cz-M1	200	2000		10	1000	0
50 L 2	Cz-M1	200	2000		10	1000	0
45 L	Cz-M1	200	2000		10	1000	0
45 L 2	Cz-M1	200	2000		10	1000	0
40 L	Cz-M1	200	2000		10	1000	0
40 L 2	Cz-M1	200	2000		10	1000	0
30 L	Cz-M1	200	2000		10	1000	0
30 L 2	Cz-M1	200	2000		10	1000	0
30 L 3	Cz-M1	200	2000		10	1000	0

30 L 4	Cz-M1	200	2000		10	1000	0
20 L	Cz-M1	200	2000		10	1000	0
20 L 2	Cz-M1	200	2000		10	1000	0
20 L 3	Cz-M1	200	2000		10	1000	0
20 L 4	Cz-M1	200	2000		10	1000	0

ABR: ABR 2 8000Hz 1: Cz-M1

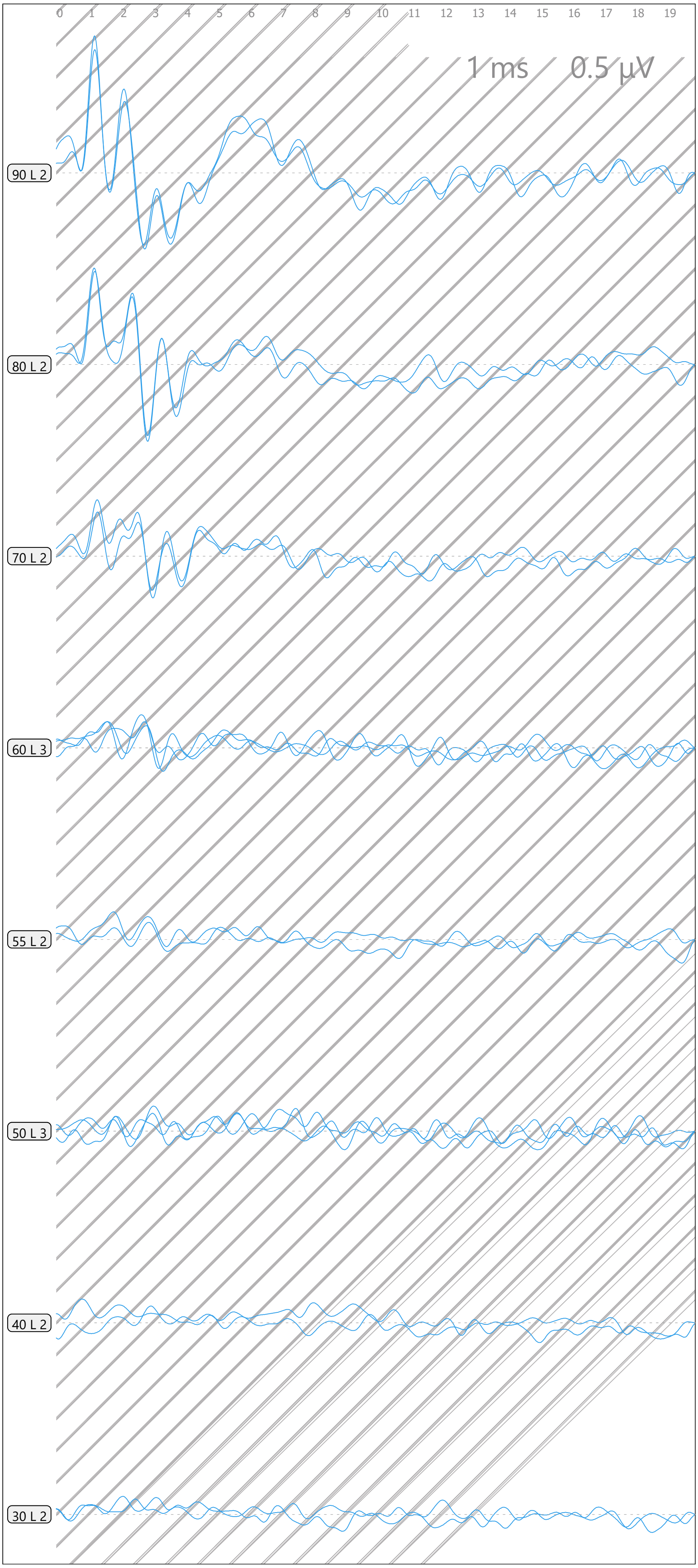
0 1 2 3 4 5 6 7 8 9 10 11 12 13 14 15 16 17 18 19
1 ms
0.5 µV
90 L 2
80 L 2
70 L 2
60 L 3
55 L 2
50 L 3
40 L 2
30 L 2

Trace parameters

N	Electr.	HPF, Hz	LPF, Hz	50 Hz	Rejection ±µV	Aver.	Reject.
90 L	Cz-M1	200	2000		10	1000	0
90 L 2	Cz-M1	200	2000		10	1000	0
80 L	Cz-M1	200	2000		10	1000	0
80 L 2	Cz-M1	200	2000		10	1000	0
70 L	Cz-M1	200	2000		10	1000	0
70 L 2	Cz-M1	200	2000		10	1000	0
60 L	Cz-M1	200	2000		10	1000	0
60 L 2	Cz-M1	200	2000		10	1000	0
60 L 3	Cz-M1	200	2000		10	1000	0
55 L	Cz-M1	200	2000		10	1000	0
55 L 2	Cz-M1	200	2000		10	1000	0
50 L	Cz-M1	200	2000		10	1000	0
50 L 2	Cz-M1	200	2000		10	1000	0
50 L 3	Cz-M1	200	2000		10	1000	0
40 L	Cz-M1	200	2000		10	1000	0
40 L 2	Cz-M1	200	2000		10	1000	0
30 L	Cz-M1	200	2000		10	1000	0
30 L 2	Cz-M1	200	2000		10	1000	0

ABR: ABR 2 CLICK
2: Cz-M2

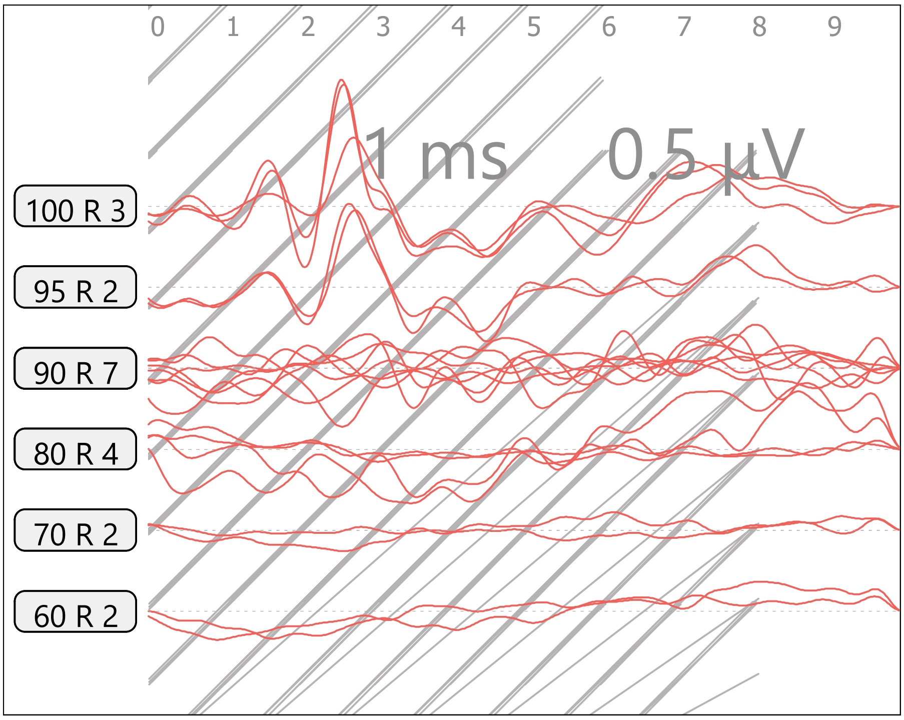

Trace parameters

N	Electr.	HPF, Hz	LPF, Hz	50 Hz	Rejection ±µV	Aver.	Rejec
100 R	Cz-M2	100	2000		10	1000	0
100 R 2	Cz-M2	100	2000		10	1000	0
100 R 3	Cz-M2	100	2000		10	1000	0
95 R	Cz-M2	100	2000		10	1000	0
95 R 2	Cz-M2	100	2000		10	1000	0
90 R	Cz-M2	100	2000		10	1000	0
90 R 2	Cz-M2	100	2000		10	1000	0
90 R 3	Cz-M2	100	2000		10	1000	0
90 R 4	Cz-M2	100	2000		10	1000	0
90 R 5	Cz-M2	100	2000		10	1000	0
90 R 6	Cz-M2	100	2000		10	1000	0
90 R 7	Cz-M2	100	2000		10	1000	0
80 R	Cz-M2	100	2000		10	267	0
80 R 2	Cz-M2	100	2000		10	1000	0
80 R 3	Cz-M2	100	2000		10	1000	0
80 R 4	Cz-M2	100	2000		10	1000	0
70 R	Cz-M2	100	2000		10	1000	0
70 R 2	Cz-M2	100	2000		10	1000	0
60 R	Cz-M2	100	2000		10	1000	0
60 R 2	Cz-M2	100	2000		10	1000	0

ABR: ABR 2 4000Hz 2: Cz-M2

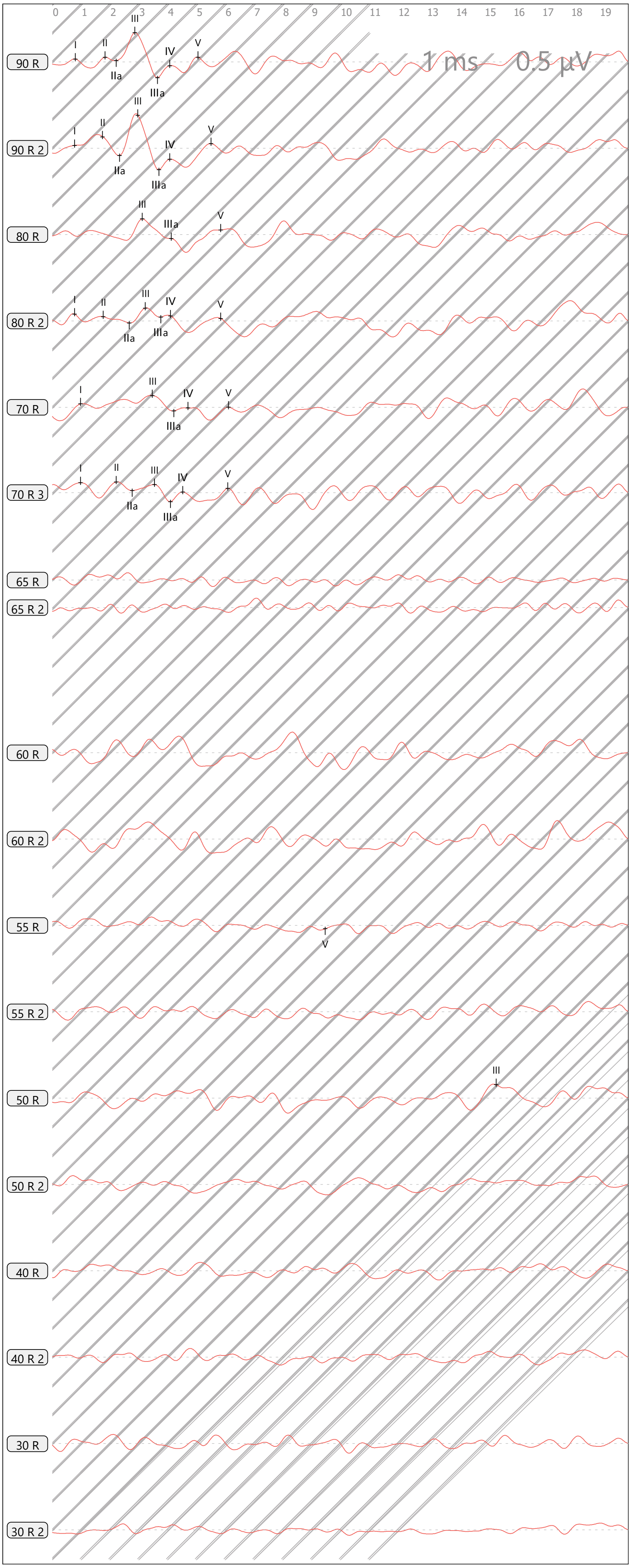

0 1 2 3 4 5 6 7 8 9 10 11 12 13 14 15 16 17 18 19
1 ms
0.5 µV
90 R
90 R 2
80 R
80 R 2
70 R
70 R 3
65 R
65 R 2
60 R
60 R 2
55 R
55 R 2
50 R
50 R 2
40 R
40 R 2
30 R
30 R 2

IV (ms)	V (ms)	I–III (ms)	I–V (ms)	III–V (ms)	
4.07	5.05	2.06	4.26	2.20	
4.07	5.50	2.20	4.74	2.54	
	5.85			2.73	
4.10	5.85	2.46	5.08	2.62	
4.71	6.11	2.49	5.13	2.65	
4.52	6.09	2.57	5.11	2.54	
	9.47				

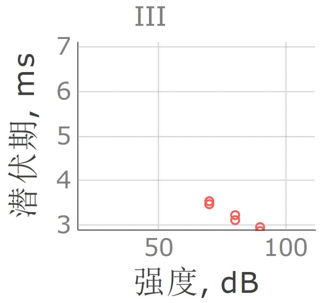

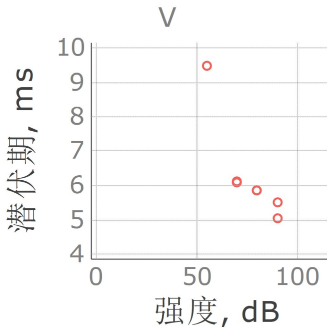

Trace parameters

N	Electr.	HPF, Hz	LPF, Hz	50 Hz	Rejection ±μV	Aver.	Reject
90 R	Cz-M2	200	2000		10	1000	0
90 R 2	Cz-M2	200	2000		10	1000	0
80 R	Cz-M2	200	2000		10	1000	0
80 R 2	Cz-M2	200	2000		10	1000	0
70 R	Cz-M2	200	2000		10	1000	0
70 R 3	Cz-M2	200	2000		10	1000	0
65 R	Cz-M2	200	2000		10	1000	0
65 R 2	Cz-M2	200	2000		10	1000	0
60 R	Cz-M2	200	2000		10	1000	0
60 R 2	Cz-M2	200	2000		10	1000	0
55 R	Cz-M2	200	2000		10	1000	0
55 R 2	Cz-M2	200	2000		10	1000	0
50 R	Cz-M2	200	2000		10	1000	0
50 R 2	Cz-M2	200	2000		10	1000	0
40 R	Cz-M2	200	2000		10	1000	0
40 R 2	Cz-M2	200	2000		10	1000	0
30 R	Cz-M2	200	2000		10	1000	0
30 R 2	Cz-M2	200	2000		10	1000	0

ABR: ABR 2 8000Hz 2: Cz-M2

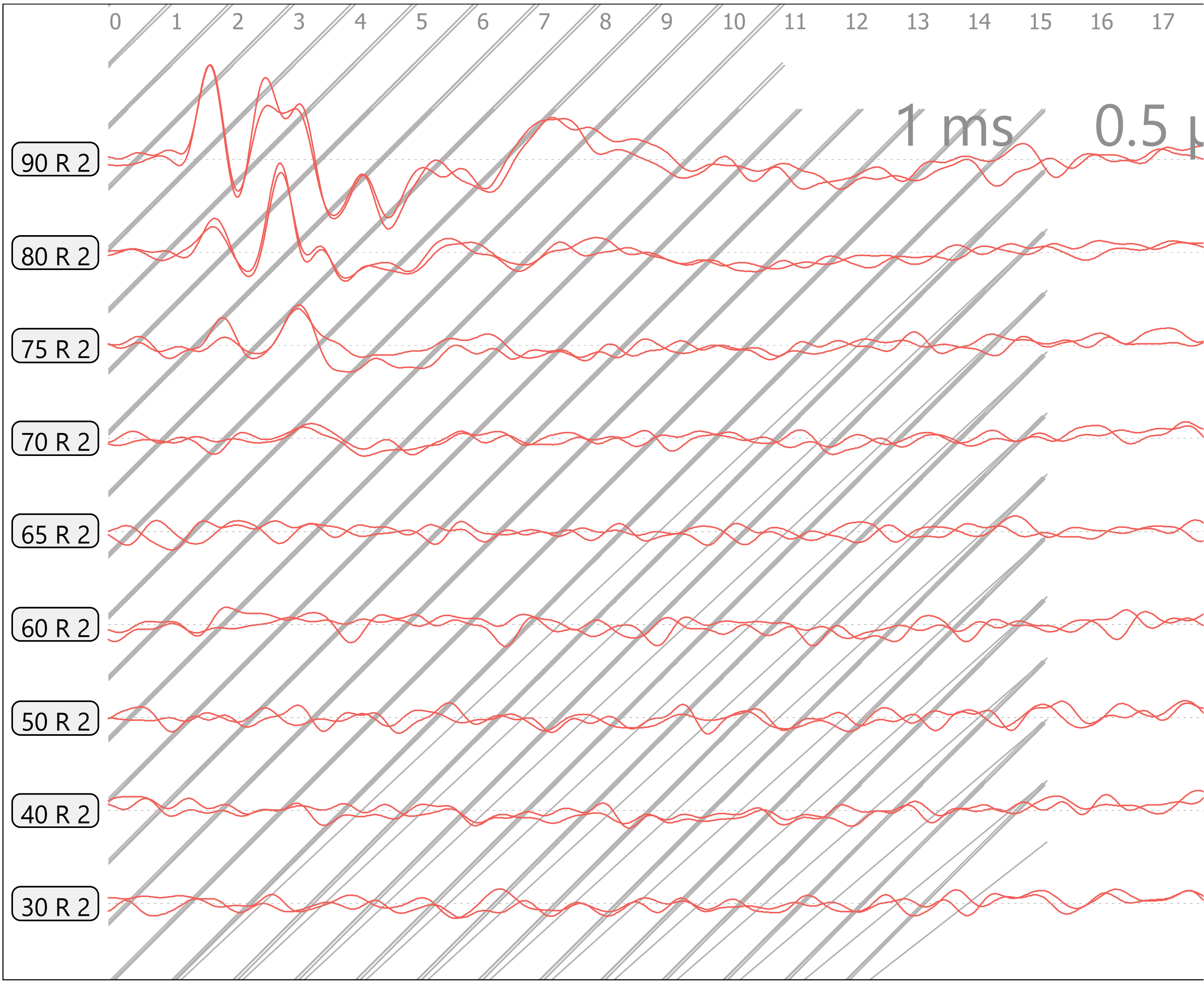

Trace parameters

N	Electr.	HPF, Hz	LPF, Hz	50 Hz	Rejection ±μV	Aver.	Reject
90 R	Cz-M2	200	2000		10	1000	0
90 R 2	Cz-M2	200	2000		10	1000	0
80 R	Cz-M2	200	2000		10	1000	0
80 R 2	Cz-M2	200	2000		10	1000	0
75 R	Cz-M2	200	2000		10	1000	0
75 R 2	Cz-M2	200	2000		10	1000	0
70 R	Cz-M2	200	2000		10	1000	0
70 R 2	Cz-M2	200	2000		10	1000	0
65 R	Cz-M2	200	2000		10	1000	0
65 R 2	Cz-M2	200	2000		10	1000	0
60 R	Cz-M2	200	2000		10	1000	0

60 R 2	Cz-M2	200	2000		10	1000	0
50 R	Cz-M2	200	2000		10	1000	0
50 R 2	Cz-M2	200	2000		10	1000	0
40 R	Cz-M2	200	2000		10	1000	0
40 R 2	Cz-M2	200	2000		10	1000	0
30 R	Cz-M2	200	2000		10	1000	0
30 R 2	Cz-M2	200	2000		10	1000	0

DPOAE: 1-12 kHz 70/70 dB 3 points

Test result (right ear):

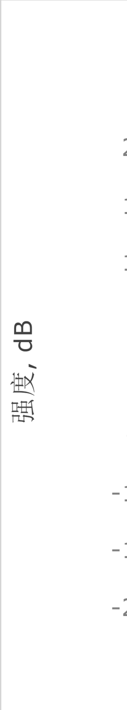

DPOAE

F2, Hz	L1, dB	L2, dB	DP, dB	噪声, dB	SNR, dB	OAE
988	68.1	68.6	-20.51	-15.00	-5.5	✘
1270	69.0	69.2	-17.14	-15.00	-2.1	✘
1778	69.9	69.9	-11.85	-15.00	3.2	✘
2222	70.2	70.2	-13.13	-15.00	1.9	✘
2500	70.4	70.4	-21.41	-15.00	-6.4	✘
3200	70.7	70.5	-17.60	-14.95	-2.7	✘
4444	71.1	70.9	-7.66	-14.94	7.3	✘
5000	71.1	70.6	-1.02	-13.05	12.0	✓
6154	70.9	70.7	4.46	-11.09	15.5	✓
8000	71.0	70.6	21.40	-6.29	27.7	✓
8889	71.1	70.3	22.37	-7.83	30.2	✓
10000	71.3	55.6	17.27	-10.78	28.1	✓
11429	56.2	60.5	13.03	-4.04	17.1	✓

(dB SPL):: 0.0

ECochG: ECochG 1:
Fpz-M1

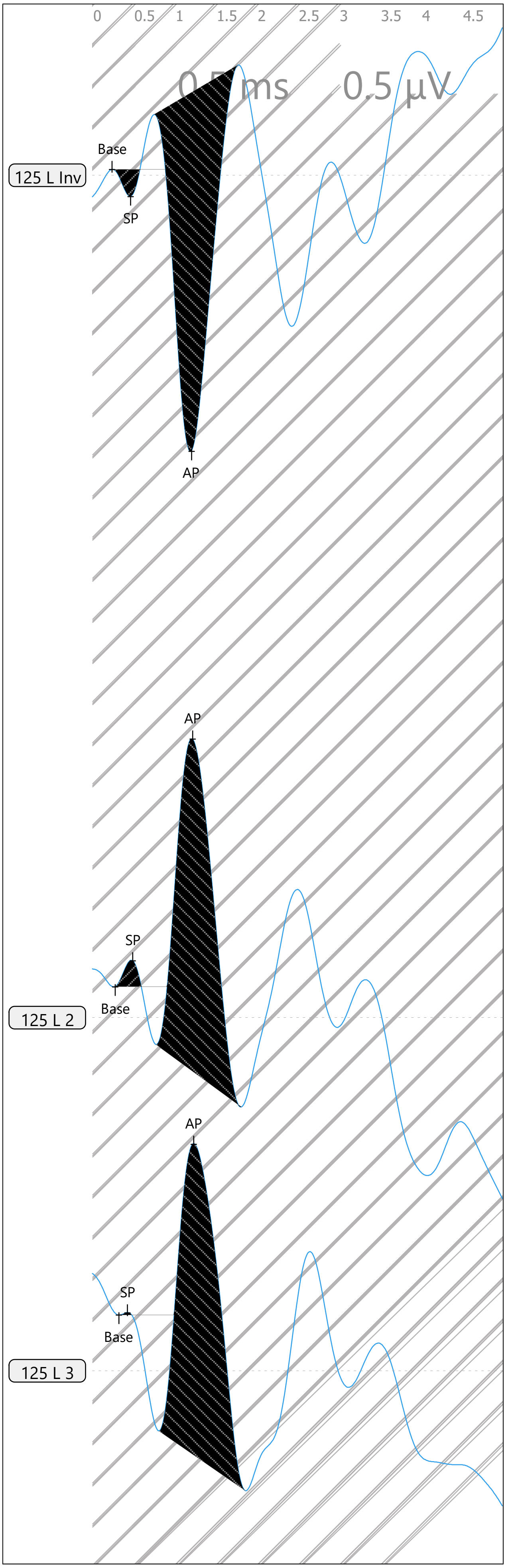

0
0.5
1
1.5
2
2.5
3
3.5
4
4.5
0.5 ms
0.5 µV
125 L Inv
Base
SP
AP
125 L 2
AP
SP
Base
125 L 3
AP
SP
Base

&&   (   )

N	Base (ms)	SP (ms)	AP (ms)	SP–Base (ms)	AP–Base (ms)	SP–Base (µV)	AP–Base (µV)	
125 L Inv	0.24	0.46	1.20	0.22	0.97	0.33	3.43	0.1[illegible]
125 L 2	0.28	0.49	1.22	0.21	0.94	0.32	3.01	0.1[illegible]
125 L 3	0.32	0.42	1.23	0.11	0.91	0.03	2.08	0.0[illegible]

Trace parameters

N	Electr.	HPF, Hz	LPF, Hz	50 Hz	Rejection ±µV	Aver.	Rej
125 L Inv	Fpz-M1	5	2000		50	1500	1[illegible]
125 L 2	Fpz-M1	5	2000		50	1500	9[illegible]
125 L 3	Fpz-M1	5	2000		50	1500	1[illegible]

ECochG: ECochG
2: Fpz-M2

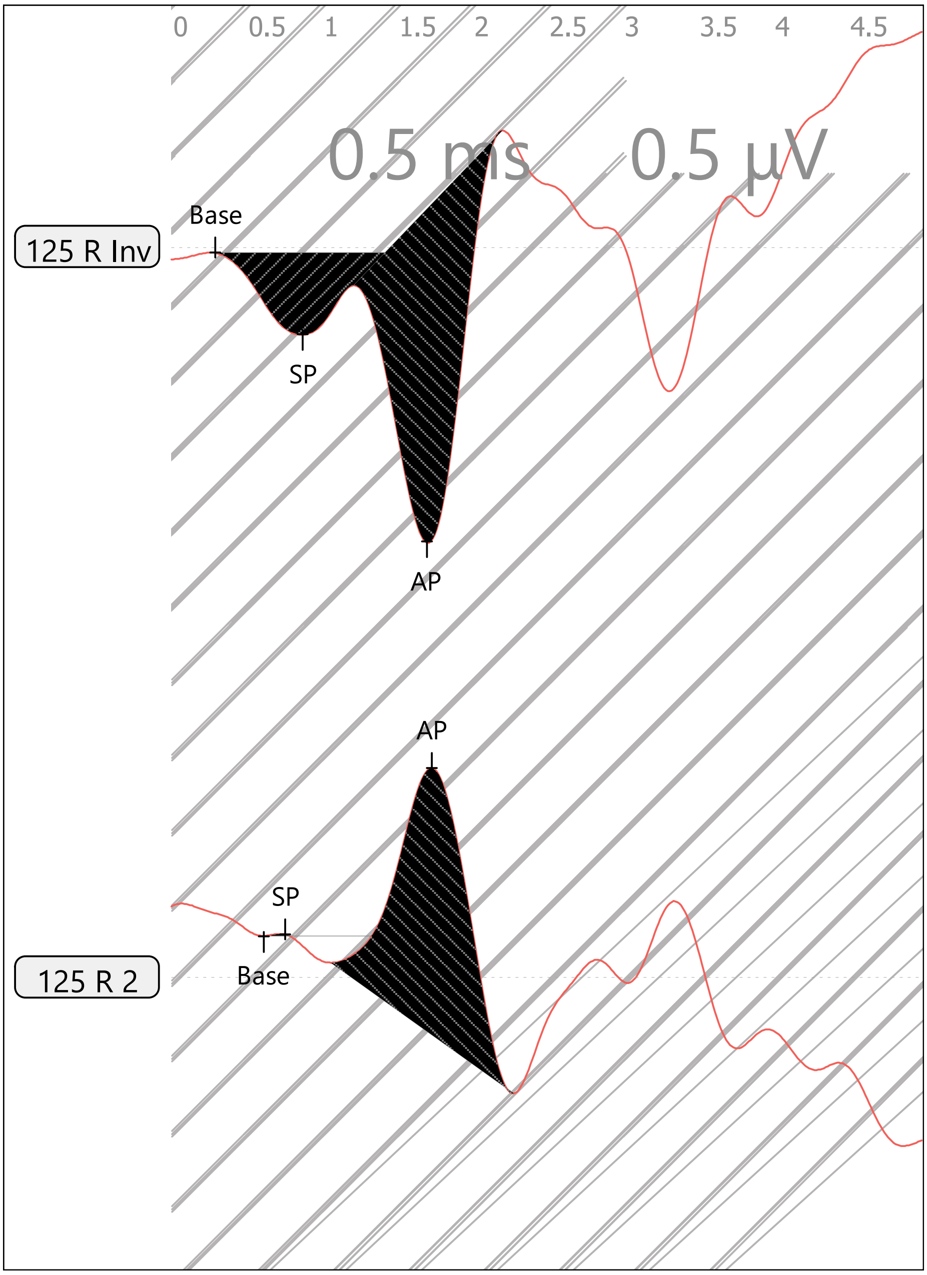

&&

N	Base (ms)	SP (ms)	AP (ms)	SP–Base (ms)	AP–Base (ms)	SP–Base (µV)	AP–Base (µV)	
125 R Inv	0.29	0.87	1.69	0.58	1.40	0.54	1.92	0.2[illegible]
125 R 2	0.61	0.75	1.73	0.15	1.12	0.01	1.11	0.0[illegible]

Trace parameters

N	Electr.	HPF, Hz	LPF, Hz	50 Hz	Rejection ±μV	Aver.	Rej[illegible]
125 R Inv	Fpz-M2	5	2000		50	946	6[illegible]
125 R 2	Fpz-M2	5	2000		50	1500	9[illegible]

CONCLUSION:

Doctor:
