# Supplementary material for: Exposure to high-altitude hypobaric hypoxic environment induces low-frequency hearing loss in C57BL/6J mice: Mediated by slowing down the postsynaptic electrical signal transmission speed in the cochlear-inferior colliculus auditory signaling pathway
Source: PLoS One. 2026 Mar 11;21(3):e0342321. doi: 10.1371/journal.pone.0342321 (PMC12978441; doi:10.1371/journal.pone.0342321)
Supplement: S1 File — (ZIP) [file pone.0342321.s001.zip › 2025.06.16-35d-01.pdf]

# Exam report

**Patient:** 2025.06.16-35d-01, - ( - )
**Date:** June 16, 2025

**ABR**: ABR 2 CLICK
1: Cz-M1

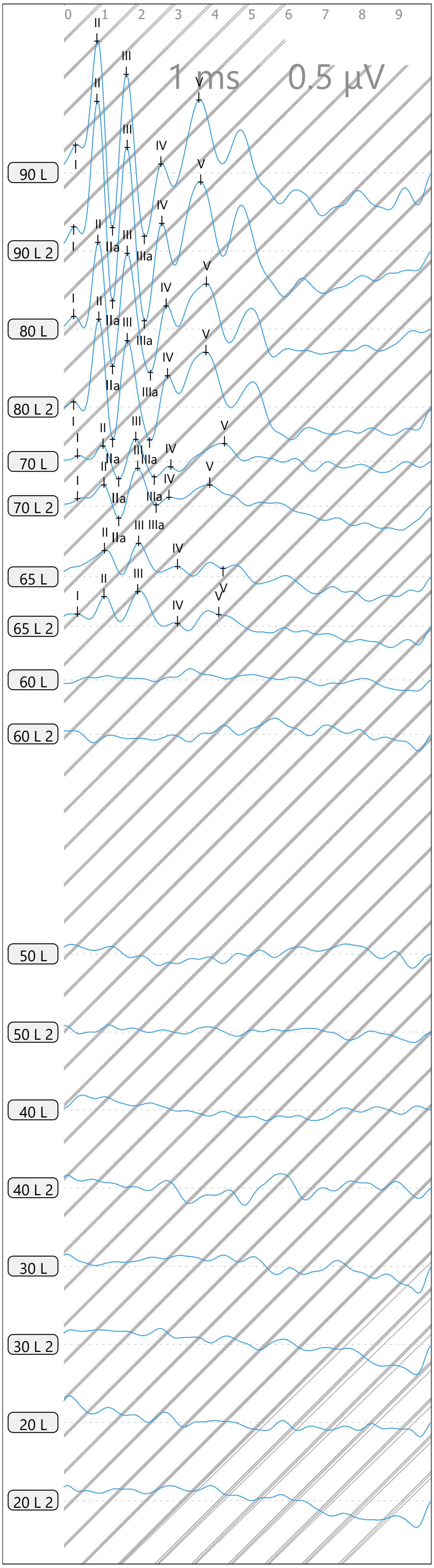
0 1 2 3 4 5 6 7 8 9
1 ms
0.5 µV
90 L
90 L 2
80 L
80 L 2
70 L
70 L 2
65 L
65 L 2
60 L
60 L 2
50 L
50 L 2
40 L
40 L 2
30 L
30 L 2
20 L
20 L 2

&&

| N | I (ms) | II (ms) | III (ms) | IV (ms) | V (ms) |
|---|---|---|---|---|---|
| 90 L | 0.32 | 0.90 | 1.69 | 2.65 | 3.68 |
| 90 L 2 | 0.26 | 0.90 | 1.72 | 2.67 | 3.73 |
| 80 L | 0.26 | 0.93 | 1.72 | 2.78 | 3.89 |
| 80 L 2 | 0.26 | 0.95 | 1.72 | 2.83 | 3.86 |
| 70 L | 0.37 | 1.06 | 1.96 | 2.91 | 4.37 |
| 70 L 2 | 0.37 | 1.08 | 2.01 | 2.86 | 3.97 |
| 65 L | | 1.11 | 2.04 | 3.10 | 4.34 |
| 65 L 2 | 0.37 | 1.08 | 2.01 | 3.10 | 4.21 |

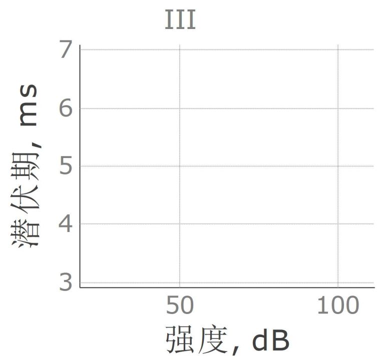


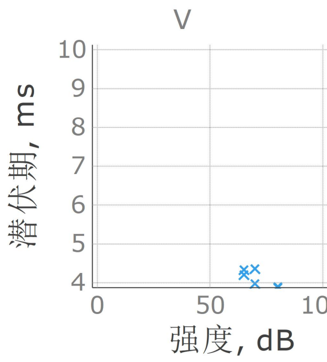


Trace parameters

| N | Electr. | HPF, Hz | LPF, Hz | 50 Hz | Rejection ±μV | Aver. | Reject. |
|---|---|---|---|---|---|---|---|
| 90 L | Cz-M1 | 100 | 2000 | | 10 | 1000 | 0 |
| 90 L 2 | Cz-M1 | 100 | 2000 | | 10 | 1000 | 0 |
| 80 L | Cz-M1 | 100 | 2000 | | 10 | 1000 | 0 |
| 80 L 2 | Cz-M1 | 100 | 2000 | | 10 | 1000 | 0 |
| 70 L | Cz-M1 | 100 | 2000 | | 10 | 1000 | 0 |
| 70 L 2 | Cz-M1 | 100 | 2000 | | 10 | 1000 | 0 |
| 65 L | Cz-M1 | 100 | 2000 | | 10 | 1000 | 0 |
| 65 L 2 | Cz-M1 | 100 | 2000 | | 10 | 1000 | 0 |
| 60 L | Cz-M1 | 100 | 2000 | | 10 | 1000 | 0 |
| 60 L 2 | Cz-M1 | 100 | 2000 | | 10 | 1000 | 0 |
| 50 L | Cz-M1 | 100 | 2000 | | 10 | 1000 | 0 |
| 50 L 2 | Cz-M1 | 100 | 2000 | | 10 | 1000 | 0 |
| 40 L | Cz-M1 | 100 | 2000 | | 10 | 1000 | 0 |
| 40 L 2 | Cz-M1 | 100 | 2000 | | 10 | 1000 | 0 |
| 30 L | Cz-M1 | 100 | 2000 | | 10 | 1000 | 0 |
| 30 L 2 | Cz-M1 | 100 | 2000 | | 10 | 1000 | 0 |
| 20 L | Cz-M1 | 100 | 2000 | | 10 | 1000 | 0 |
| 20 L 2 | Cz-M1 | 100 | 2000 | | 10 | 1000 | 0 |

**ABR**: ABR 2 tone burst 4000Hz 1
: Cz-M1

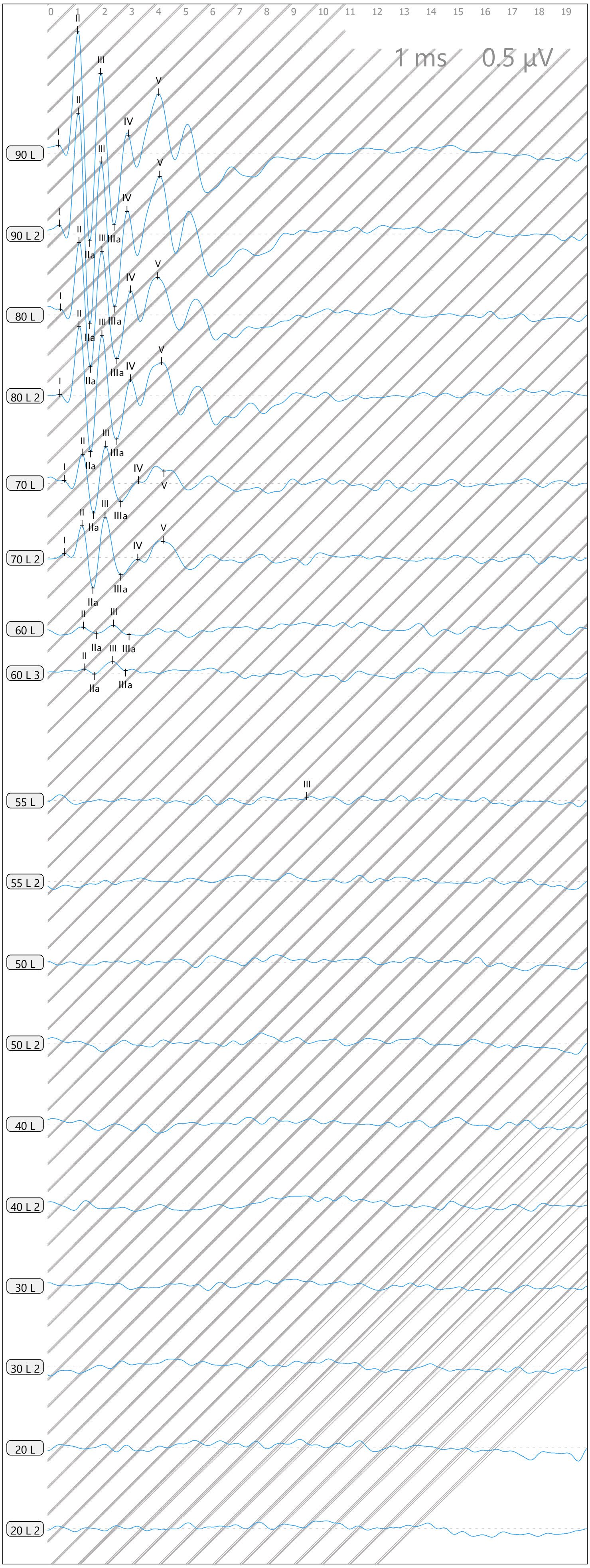

0 1 2 3 4 5 6 7 8 9 10 11 12 13 14 15 16 17 18 19
1 ms
0.5 µV
90 L
90 L 2
80 L
80 L 2
70 L
70 L 2
60 L
60 L 3
55 L
55 L 2
50 L
50 L 2
40 L
40 L 2
30 L
30 L 2
20 L
20 L 2

&&

| N | I (ms) | II (ms) | III (ms) | Ⅳ (ms) | V (ms) |
|---|---|---|---|---|---|
| 90 L | 0.40 | 1.11 | 1.96 | 2.99 | 4.10 |
| 90 L 2 | 0.42 | 1.14 | 1.98 | 2.94 | 4.15 |
| 80 L | 0.48 | 1.16 | 2.01 | 3.07 | 4.07 |
| 80 L 2 | 0.45 | 1.16 | 2.01 | 3.07 | 4.21 |
| 70 L | 0.61 | 1.30 | 2.14 | 3.36 | 4.31 |
| 70 L 2 | 0.61 | 1.27 | 2.12 | 3.33 | 4.29 |
| 60 L | | 1.32 | 2.43 | | |
| 60 L 3 | | 1.35 | 2.41 | | |
| 55 L | | | 9.60 | | |

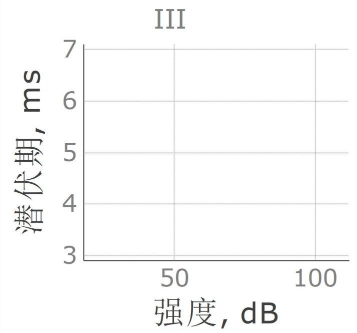


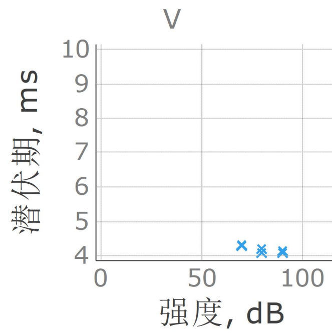


Trace parameters

| N | Electr. | HPF, Hz | LPF, Hz | 50 Hz | Rejection ±μV | Aver. | Reject. |
|---|---|---|---|---|---|---|---|
| 90 L | Cz-M1 | 200 | 2000 | | 10 | 1000 | 0 |
| 90 L 2 | Cz-M1 | 200 | 2000 | | 10 | 1000 | 0 |
| 80 L | Cz-M1 | 200 | 2000 | | 10 | 1000 | 0 |
| 80 L 2 | Cz-M1 | 200 | 2000 | | 10 | 1000 | 0 |
| 70 L | Cz-M1 | 200 | 2000 | | 10 | 1000 | 0 |
| 70 L 2 | Cz-M1 | 200 | 2000 | | 10 | 1000 | 0 |
| 60 L | Cz-M1 | 200 | 2000 | | 10 | 1000 | 0 |
| 60 L 3 | Cz-M1 | 200 | 2000 | | 10 | 1000 | 0 |
| 55 L | Cz-M1 | 200 | 2000 | | 10 | 1000 | 0 |
| 55 L 2 | Cz-M1 | 200 | 2000 | | 10 | 1000 | 0 |
| 50 L | Cz-M1 | 200 | 2000 | | 10 | 1000 | 0 |
| 50 L 2 | Cz-M1 | 200 | 2000 | | 10 | 1000 | 0 |
| 40 L | Cz-M1 | 200 | 2000 | | 10 | 1000 | 0 |
| 40 L 2 | Cz-M1 | 200 | 2000 | | 10 | 1000 | 0 |
| 30 L | Cz-M1 | 200 | 2000 | | 10 | 1000 | 0 |
| 30 L 2 | Cz-M1 | 200 | 2000 | | 10 | 1000 | 0 |
| 20 L | Cz-M1 | 200 | 2000 | | 10 | 1000 | 0 |
| 20 L 2 | Cz-M1 | 200 | 2000 | | 10 | 1000 | 0 |

**ABR**: ABR 2 8000Hz 1: Cz-M1

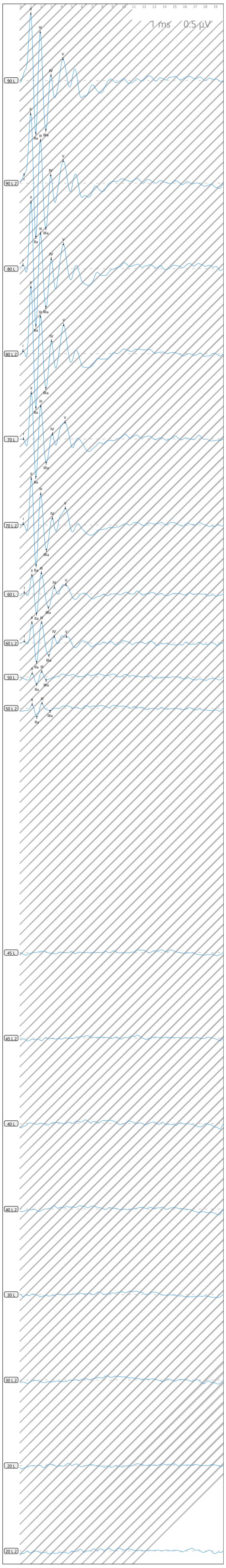
0 1 2 3 4 5 6 7 8 9 10 11 12 13 14 15 16 17 18 19
1 ms
0.5 µV
90 L
90 L 2
80 L
80 L 2
70 L
70 L 2
60 L
60 L 2
50 L
50 L 2
45 L
45 L 2
40 L
40 L 2
30 L
30 L 2
20 L
20 L 2

&&

| N | I (ms) | II (ms) | III (ms) | IV (ms) | V (ms) |
|---|---|---|---|---|---|
| 90 L | | 1.08 | 2.01 | 3.07 | 4.23 |
| 90 L 2 | 0.42 | 1.06 | 2.04 | 3.04 | 4.26 |
| 80 L | 0.32 | 1.11 | 2.04 | 3.10 | 4.29 |
| 80 L 2 | 0.32 | 1.08 | 2.01 | 3.12 | 4.31 |
| 70 L | 0.37 | 1.14 | 2.06 | 3.20 | 4.45 |
| 70 L 2 | 0.34 | 1.14 | 2.06 | 3.20 | 4.47 |
| 60 L | 0.45 | 1.19 | 2.12 | 3.41 | 4.55 |
| 60 L 2 | 0.45 | 1.19 | 2.14 | 3.39 | 4.58 |
| 50 L | | 1.22 | 2.17 | | |
| 50 L 2 | | 1.22 | 2.20 | | |

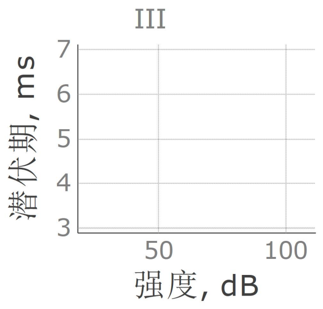


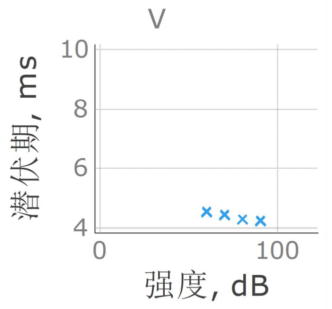


Trace parameters

| N | Electr. | HPF, Hz | LPF, Hz | 50 Hz | Rejection ±μV | Aver. | Reject. |
|---|---|---|---|---|---|---|---|
| 90 L | Cz-M1 | 200 | 2000 | | 10 | 1000 | 0 |
| 90 L 2 | Cz-M1 | 200 | 2000 | | 10 | 1000 | 0 |
| 80 L | Cz-M1 | 200 | 2000 | | 10 | 1000 | 0 |
| 80 L 2 | Cz-M1 | 200 | 2000 | | 10 | 1000 | 0 |
| 70 L | Cz-M1 | 200 | 2000 | | 10 | 1000 | 0 |
| 70 L 2 | Cz-M1 | 200 | 2000 | | 10 | 1000 | 0 |
| 60 L | Cz-M1 | 200 | 2000 | | 10 | 1000 | 0 |
| 60 L 2 | Cz-M1 | 200 | 2000 | | 10 | 1000 | 0 |
| 50 L | Cz-M1 | 200 | 2000 | | 10 | 1000 | 0 |
| 50 L 2 | Cz-M1 | 200 | 2000 | | 10 | 1000 | 0 |
| 45 L | Cz-M1 | 200 | 2000 | | 10 | 1000 | 0 |
| 45 L 2 | Cz-M1 | 200 | 2000 | | 10 | 1000 | 0 |
| 40 L | Cz-M1 | 200 | 2000 | | 10 | 1000 | 0 |
| 40 L 2 | Cz-M1 | 200 | 2000 | | 10 | 1000 | 0 |
| 30 L | Cz-M1 | 200 | 2000 | | 10 | 1000 | 0 |
| 30 L 2 | Cz-M1 | 200 | 2000 | | 10 | 1000 | 0 |
| 20 L | Cz-M1 | 200 | 2000 | | 10 | 1000 | 0 |
| 20 L 2 | Cz-M1 | 200 | 2000 | | 10 | 1000 | 0 |

**ABR**: ABR 2 CLICK
2: Cz-M2

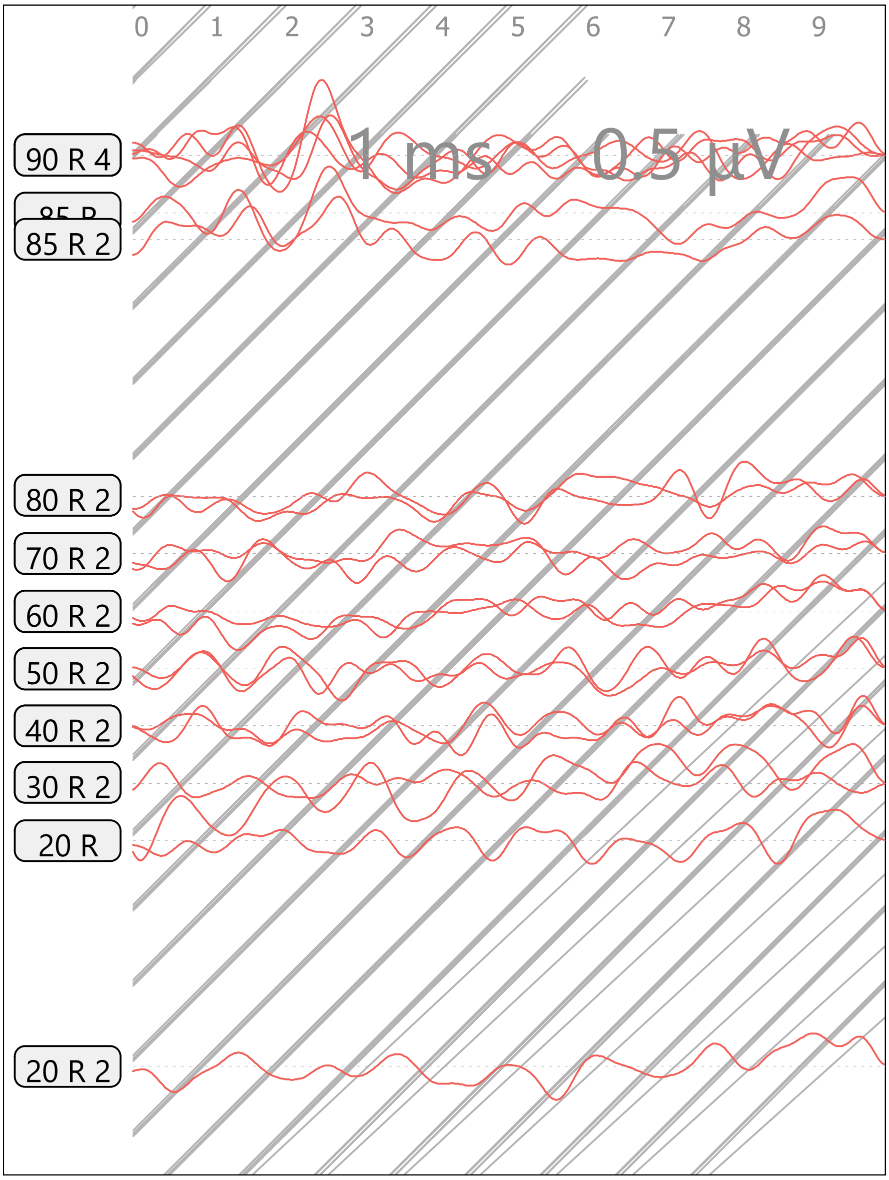


Trace parameters

| N | Electr. | HPF, Hz | LPF, Hz | 50 Hz | Rejection ±µV | Aver. | Reject |
|---|---|---|---|---|---|---|---|
| 90 R | Cz-M2 | 100 | 2000 | | 10 | 1000 | 0 |
| 90 R 2 | Cz-M2 | 100 | 2000 | | 10 | 1000 | 0 |
| 90 R 3 | Cz-M2 | 100 | 2000 | | 10 | 1000 | 0 |
| 90 R 4 | Cz-M2 | 100 | 2000 | | 10 | 1000 | 0 |
| 85 R | Cz-M2 | 100 | 2000 | | 10 | 1000 | 0 |
| 85 R 2 | Cz-M2 | 100 | 2000 | | 10 | 1000 | 0 |
| 80 R | Cz-M2 | 100 | 2000 | | 10 | 1000 | 0 |
| 80 R 2 | Cz-M2 | 100 | 2000 | | 10 | 1000 | 0 |
| 70 R | Cz-M2 | 100 | 2000 | | 10 | 1000 | 0 |
| 70 R 2 | Cz-M2 | 100 | 2000 | | 10 | 1000 | 0 |
| 60 R | Cz-M2 | 100 | 2000 | | 10 | 1000 | 0 |
| 60 R 2 | Cz-M2 | 100 | 2000 | | 10 | 1000 | 0 |
| 50 R | Cz-M2 | 100 | 2000 | | 10 | 1000 | 0 |
| 50 R 2 | Cz-M2 | 100 | 2000 | | 10 | 1000 | 0 |
| 40 R | Cz-M2 | 100 | 2000 | | 10 | 1000 | 0 |
| 40 R 2 | Cz-M2 | 100 | 2000 | | 10 | 1000 | 0 |
| 30 R | Cz-M2 | 100 | 2000 | | 10 | 1000 | 0 |

| | | | | | | | |
|---|---|---|---|---|---|---|---|
| 30 R 2 | Cz-M2 | 100 | 2000 | | 10 | 1000 | 0 |
| 20 R | Cz-M2 | 100 | 2000 | | 10 | 1000 | 0 |
| 20 R 2 | Cz-M2 | 100 | 2000 | | 10 | 1000 | 0 |

**ABR**: ABR 2 8000Hz 2: Cz-M2

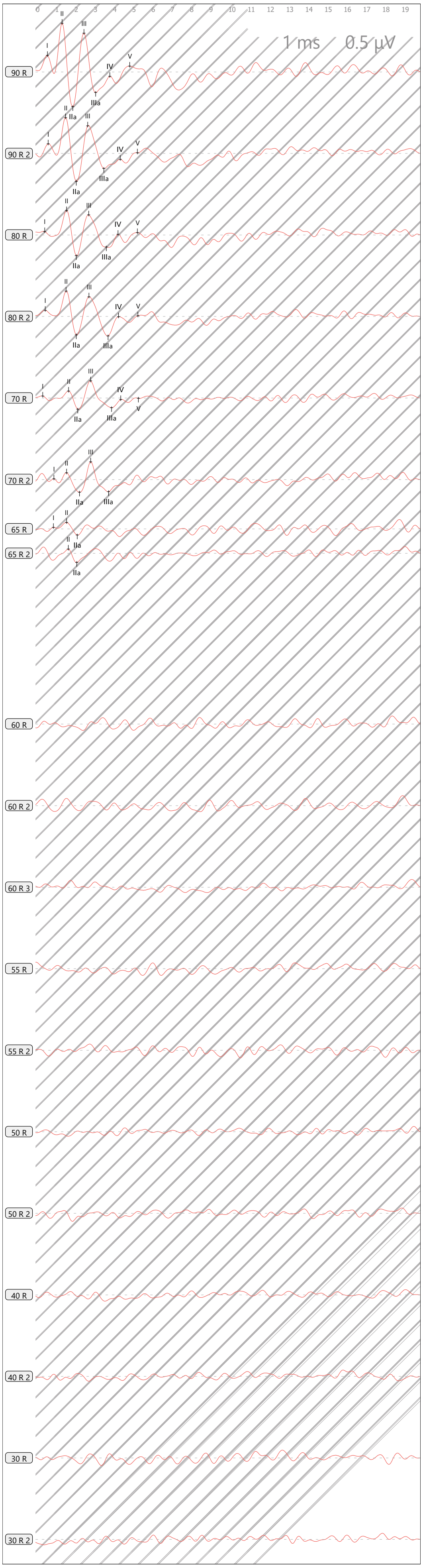

0 1 2 3 4 5 6 7 8 9 10 11 12 13 14 15 16 17 18 19
1 ms
0.5 µV
90 R
90 R 2
80 R
80 R 2
70 R
70 R 2
65 R
65 R 2
60 R
60 R 2
60 R 3
55 R
55 R 2
50 R
50 R 2
40 R
40 R 2
30 R
30 R 2

| IV (ms) | V (ms) | I–III (ms) | I–V (ms) | III–V (ms) | |
|---|---|---|---|---|---|
| 3.86 | 4.89 | 1.91 | 4.29 | 2.38 | |
| 4.39 | 5.29 | 2.04 | 4.63 | 2.59 | |
| 4.29 | 5.29 | 2.28 | 4.82 | 2.54 | |
| 4.31 | 5.32 | 2.28 | 4.82 | 2.54 | |
| 4.42 | 5.34 | 2.49 | 4.97 | 2.49 | |
| | | 1.91 | | | |
| | | | | | |
| | | | | | |

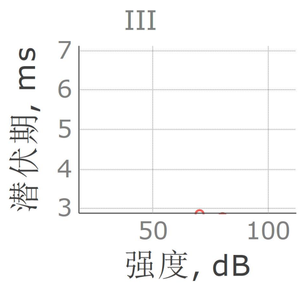


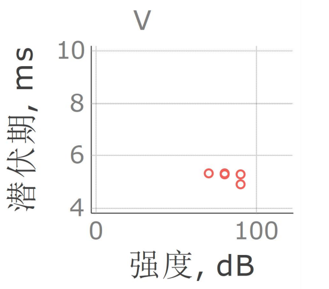


Trace parameters

| N | Electr. | HPF, Hz | LPF, Hz | 50 Hz | Rejection ±μV | Aver. | Reject |
|---|---|---|---|---|---|---|---|
| 90 R | Cz-M2 | 200 | 2000 | | 10 | 1000 | 0 |
| 90 R 2 | Cz-M2 | 200 | 2000 | | 10 | 1000 | 0 |
| 80 R | Cz-M2 | 200 | 2000 | | 10 | 1000 | 0 |
| 80 R 2 | Cz-M2 | 200 | 2000 | | 10 | 1000 | 0 |
| 70 R | Cz-M2 | 200 | 2000 | | 10 | 1000 | 0 |
| 70 R 2 | Cz-M2 | 200 | 2000 | | 10 | 1000 | 0 |
| 65 R | Cz-M2 | 200 | 2000 | | 10 | 1000 | 0 |
| 65 R 2 | Cz-M2 | 200 | 2000 | | 10 | 1000 | 0 |
| 60 R | Cz-M2 | 200 | 2000 | | 10 | 1000 | 0 |
| 60 R 2 | Cz-M2 | 200 | 2000 | | 10 | 1000 | 0 |
| 60 R 3 | Cz-M2 | 200 | 2000 | | 10 | 1000 | 0 |
| 55 R | Cz-M2 | 200 | 2000 | | 10 | 1000 | 0 |
| 55 R 2 | Cz-M2 | 200 | 2000 | | 10 | 1000 | 0 |
| 50 R | Cz-M2 | 200 | 2000 | | 10 | 1000 | 0 |
| 50 R 2 | Cz-M2 | 200 | 2000 | | 10 | 1000 | 0 |
| 40 R | Cz-M2 | 200 | 2000 | | 10 | 1000 | 0 |
| 40 R 2 | Cz-M2 | 200 | 2000 | | 10 | 1000 | 0 |
| 30 R | Cz-M2 | 200 | 2000 | | 10 | 1000 | 0 |
| 30 R 2 | Cz-M2 | 200 | 2000 | | 10 | 1000 | 0 |

**ABR**: ABR 2 CLICK 2: Cz-M2

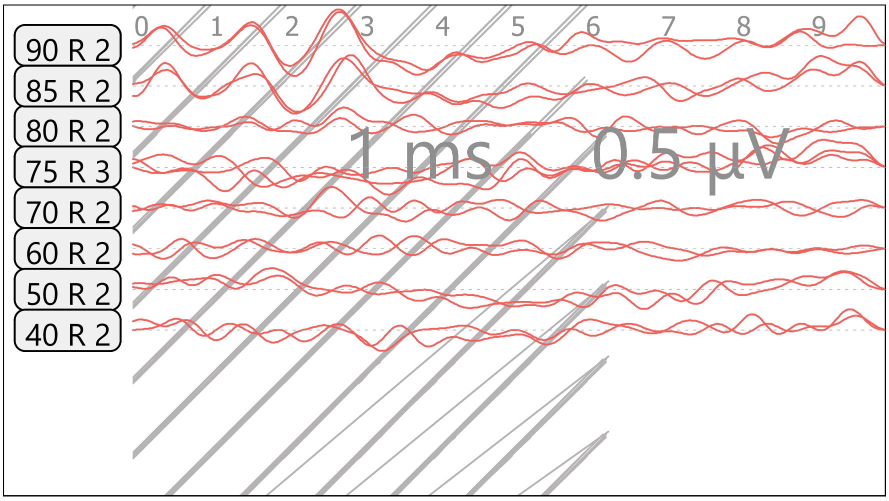


Trace parameters

| N | Electr. | HPF, Hz | LPF, Hz | 50 Hz | Rejection ±µV | Aver. | Reject |
|---|---|---|---|---|---|---|---|
| 90 R | Cz-M2 | 100 | 2000 | | 10 | 1000 | 0 |
| 90 R 2 | Cz-M2 | 100 | 2000 | | 10 | 1000 | 0 |
| 85 R | Cz-M2 | 100 | 2000 | | 10 | 1000 | 0 |
| 85 R 2 | Cz-M2 | 100 | 2000 | | 10 | 1000 | 0 |
| 80 R | Cz-M2 | 100 | 2000 | | 10 | 1000 | 0 |
| 80 R 2 | Cz-M2 | 100 | 2000 | | 10 | 1000 | 0 |
| 75 R | Cz-M2 | 100 | 2000 | | 10 | 1000 | 0 |
| 75 R 2 | Cz-M2 | 100 | 2000 | | 10 | 1000 | 0 |
| 75 R 3 | Cz-M2 | 100 | 2000 | | 10 | 1000 | 0 |
| 70 R | Cz-M2 | 100 | 2000 | | 10 | 1000 | 0 |
| 70 R 2 | Cz-M2 | 100 | 2000 | | 10 | 1000 | 0 |
| 60 R | Cz-M2 | 100 | 2000 | | 10 | 1000 | 0 |
| 60 R 2 | Cz-M2 | 100 | 2000 | | 10 | 1000 | 0 |
| 50 R | Cz-M2 | 100 | 2000 | | 10 | 1000 | 0 |
| 50 R 2 | Cz-M2 | 100 | 2000 | | 10 | 1000 | 0 |
| 40 R | Cz-M2 | 100 | 2000 | | 10 | 1000 | 0 |
| 40 R 2 | Cz-M2 | 100 | 2000 | | 10 | 1000 | 0 |

**ABR**: ABR 2 4000Hz 2: Cz-M2

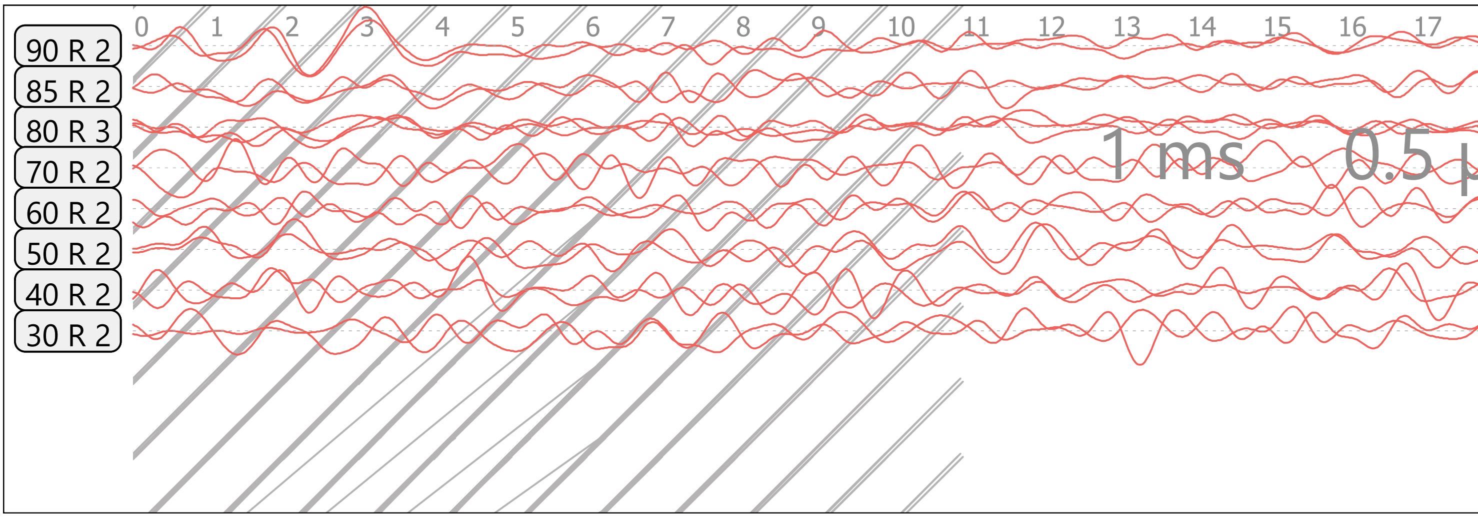


Trace parameters

| N | Electr. | HPF, Hz | LPF, Hz | 50 Hz | Rejection ±µV | Aver. | Reject |
|---|---|---|---|---|---|---|---|

| | | | | | | | |
|---|---|---|---|---|---|---|---|
| 90 R | Cz-M2 | 200 | 2000 | | 10 | 1000 | 0 |
| 90 R 2 | Cz-M2 | 200 | 2000 | | 10 | 1000 | 0 |
| 85 R | Cz-M2 | 200 | 2000 | | 10 | 1000 | 0 |
| 85 R 2 | Cz-M2 | 200 | 2000 | | 10 | 1000 | 0 |
| 80 R | Cz-M2 | 200 | 2000 | | 10 | 1000 | 0 |
| 80 R 2 | Cz-M2 | 200 | 2000 | | 10 | 1000 | 0 |
| 80 R 3 | Cz-M2 | 200 | 2000 | | 10 | 1000 | 0 |
| 70 R | Cz-M2 | 200 | 2000 | | 10 | 1000 | 0 |
| 70 R 2 | Cz-M2 | 200 | 2000 | | 10 | 1000 | 0 |
| 60 R | Cz-M2 | 200 | 2000 | | 10 | 1000 | 0 |
| 60 R 2 | Cz-M2 | 200 | 2000 | | 10 | 1000 | 0 |
| 50 R | Cz-M2 | 200 | 2000 | | 10 | 1000 | 0 |
| 50 R 2 | Cz-M2 | 200 | 2000 | | 10 | 1000 | 0 |
| 40 R | Cz-M2 | 200 | 2000 | | 10 | 1000 | 0 |
| 40 R 2 | Cz-M2 | 200 | 2000 | | 10 | 1000 | 0 |
| 30 R | Cz-M2 | 200 | 2000 | | 10 | 1000 | 0 |
| 30 R 2 | Cz-M2 | 200 | 2000 | | 10 | 1000 | 0 |

**ECochG**: ECochG 1:
Fpz-M1

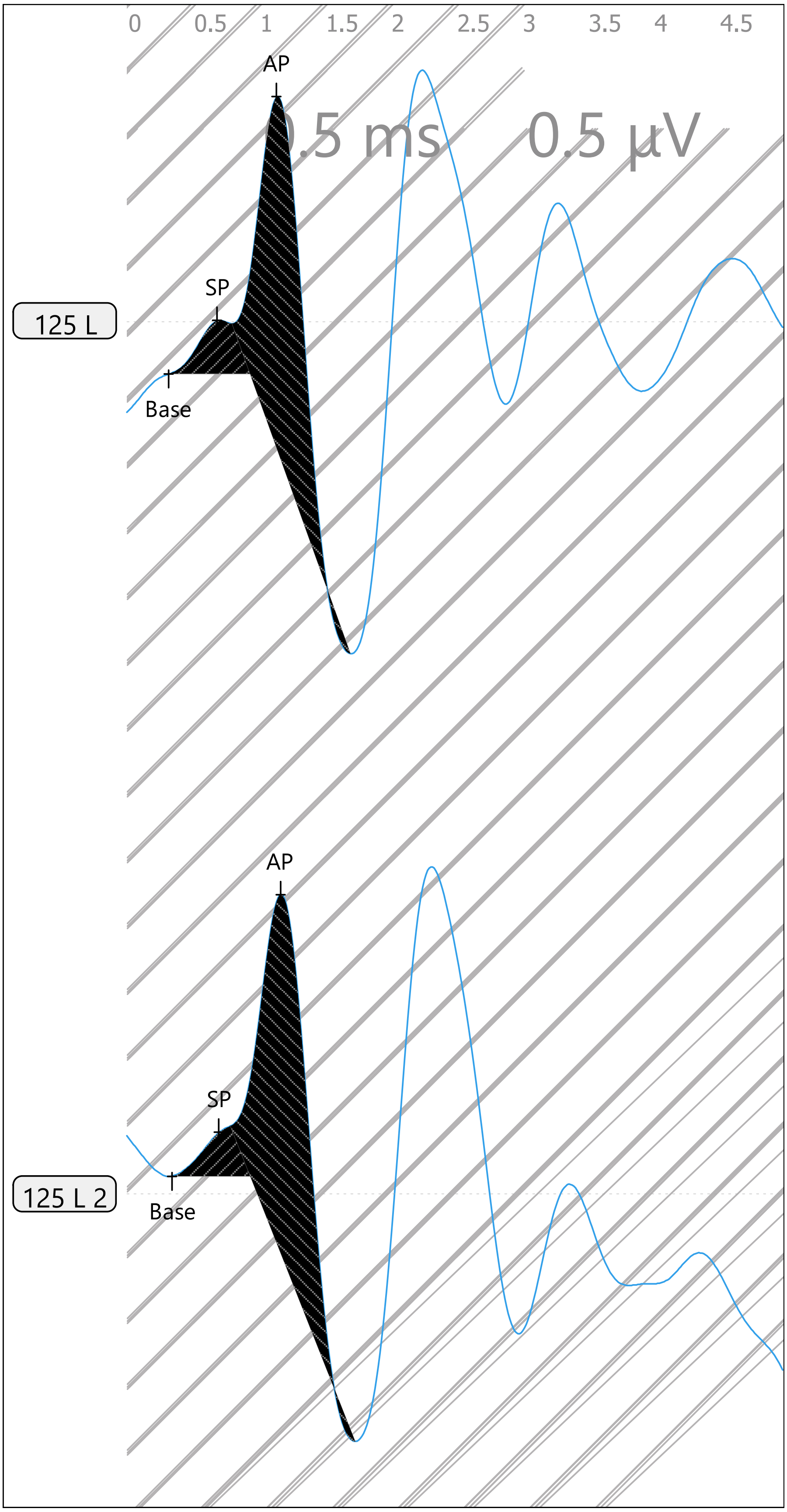


&&

| N | Base (ms) | SP (ms) | AP (ms) | SP–Base (ms) | AP–Base (ms) | SP–Base (µV) | AP–Base (µV) | |
|---|---|---|---|---|---|---|---|---|
| 125 L | 0.32 | 0.69 | 1.14 | 0.37 | 0.82 | 0.41 | 2.10 | 0.19 |
| 125 L 2 | 0.34 | 0.70 | 1.16 | 0.36 | 0.82 | 0.33 | 2.13 | 0.15 |

Trace parameters

| N | Electr. | HPF, Hz | LPF, Hz | 50 Hz | Rejection ±µV | Aver. | Rejec |
|---|---|---|---|---|---|---|---|

| | | | | | | | |
|---|---|---|---|---|---|---|---|
| 125 L | Fpz-M1 | 5 | 2000 | | 50 | 1500 | 263 |
| 125 L 2 | Fpz-M1 | 5 | 2000 | | 50 | 1500 | 306 |

**ECochG**: ECochG 2:
Fpz-M2

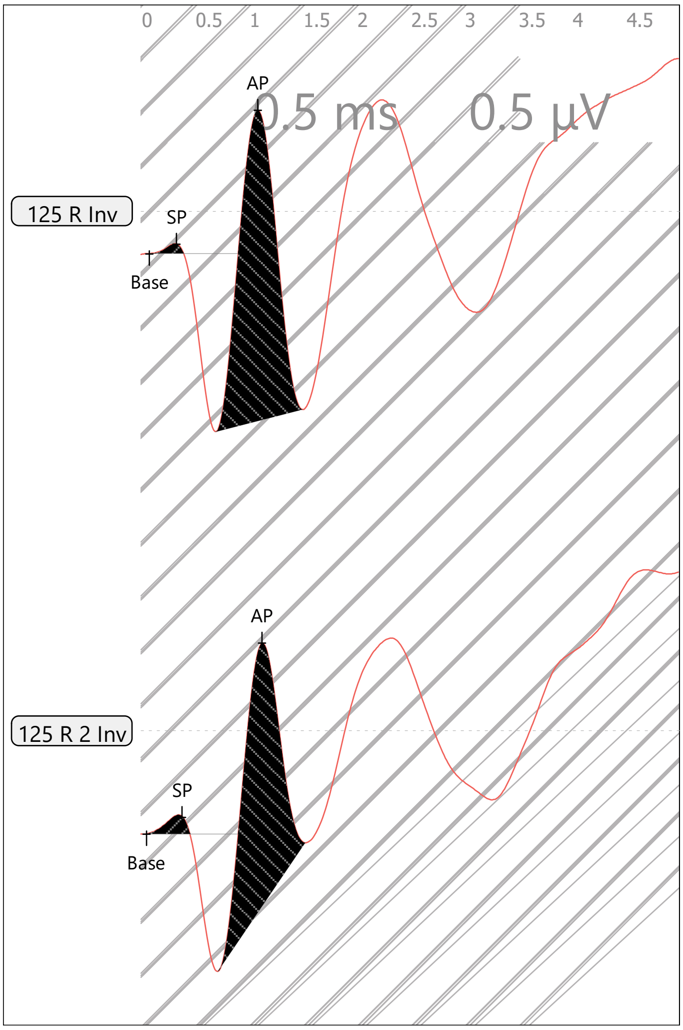


&&

| N | Base (ms) | SP (ms) | AP (ms) | SP–Base (ms) | AP–Base (ms) | SP–Base (µV) | AP–Base (µV) | |
|---|---|---|---|---|---|---|---|---|
| 125 R Inv | 0.08 | 0.33 | 1.08 | 0.25 | 1.01 | 0.09 | 1.33 | 0 |
| 125 R 2 Inv | 0.05 | 0.38 | 1.12 | 0.33 | 1.07 | 0.16 | 1.77 | 0 |

Trace parameters

| N | Electr. | HPF, Hz | LPF, Hz | 50 Hz | Rejection ±µV | Aver. | R |
|---|---|---|---|---|---|---|---|
| 125 R Inv | Fpz-M2 | 5 | 2000 | | 50 | 1500 | |
| 125 R 2 Inv | Fpz-M2 | 5 | 2000 | | 50 | 1500 | |

**CONCLUSION:**

**Doctor:**
