# Supplementary material for: Exposure to high-altitude hypobaric hypoxic environment induces low-frequency hearing loss in C57BL/6J mice: Mediated by slowing down the postsynaptic electrical signal transmission speed in the cochlear-inferior colliculus auditory signaling pathway
Source: PLoS One. 2026 Mar 11;21(3):e0342321. doi: 10.1371/journal.pone.0342321 (PMC12978441; doi:10.1371/journal.pone.0342321)
Supplement: S1 File — (ZIP) [file pone.0342321.s001.zip › 2025.06.16-35d-04.pdf]

Exam report

Patient: 2025.06.16-35d-04**,** - (-)
Date: June 16, 2025

ABR: ABR 2 CLICK
1: Cz-M1

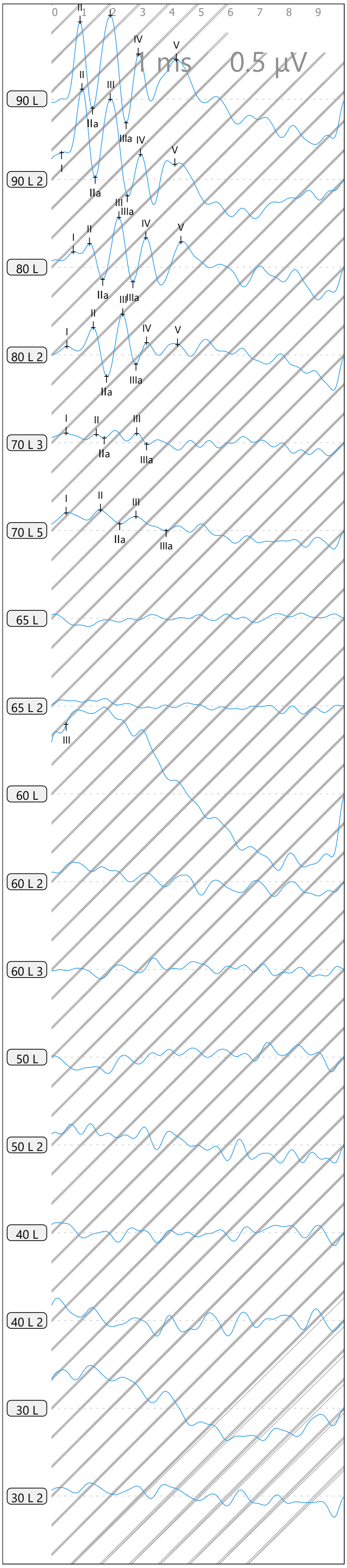
0
1
2
3
4
5
6
7
8
9
1 ms
0.5 µV
90 L
90 L 2
80 L
80 L 2
70 L 3
70 L 5
65 L
65 L 2
60 L
60 L 2
60 L 3
50 L
50 L 2
40 L
40 L 2
30 L
30 L 2

latency&& amplitude (左耳

N	I (ms)	II (ms)	III (ms)	IV (ms)	V (ms)
90 L		0.98	2.01	2.96	4.26
90 L 2	0.34	1.03	2.01	3.04	4.21
80 L	0.74	1.30	2.30	3.23	4.42
80 L 2	0.53	1.43	2.43	3.25	4.31
70 L 3	0.50	1.53	2.91		
70 L 5	0.50	1.67	2.88		
60 L			0.50		

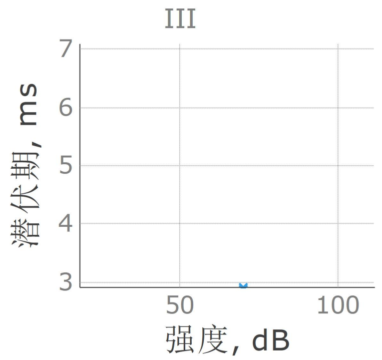

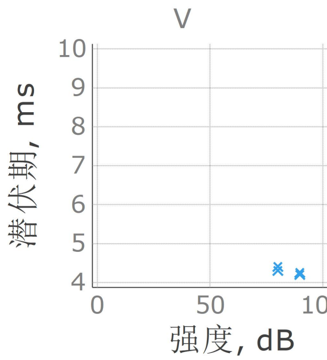

Trace parameters

N	Electr.	HPF, Hz	LPF, Hz	50 Hz	Rejection ±μV	Aver.	Reject.
90 L	Cz-M1	100	2000		10	1000	0
90 L 2	Cz-M1	100	2000		10	1000	0
80 L	Cz-M1	100	2000		10	1000	0
80 L 2	Cz-M1	100	2000		10	1000	0
70 L 3	Cz-M1	100	2000		10	1000	0
70 L 5	Cz-M1	100	2000		10	1000	0
65 L	Cz-M1	100	2000		10	1000	0
65 L 2	Cz-M1	100	2000		10	1000	0
60 L	Cz-M1	100	2000		10	1000	0
60 L 2	Cz-M1	100	2000		10	1000	0
60 L 3	Cz-M1	100	2000		10	1000	0
50 L	Cz-M1	100	2000		10	1000	0
50 L 2	Cz-M1	100	2000		10	1000	0
40 L	Cz-M1	100	2000		10	1000	0
40 L 2	Cz-M1	100	2000		10	1000	0
30 L	Cz-M1	100	2000		10	1000	0
30 L 2	Cz-M1	100	2000		10	1000	0

ABR: ABR 2 tone burst 4000Hz 1
: Cz-M1

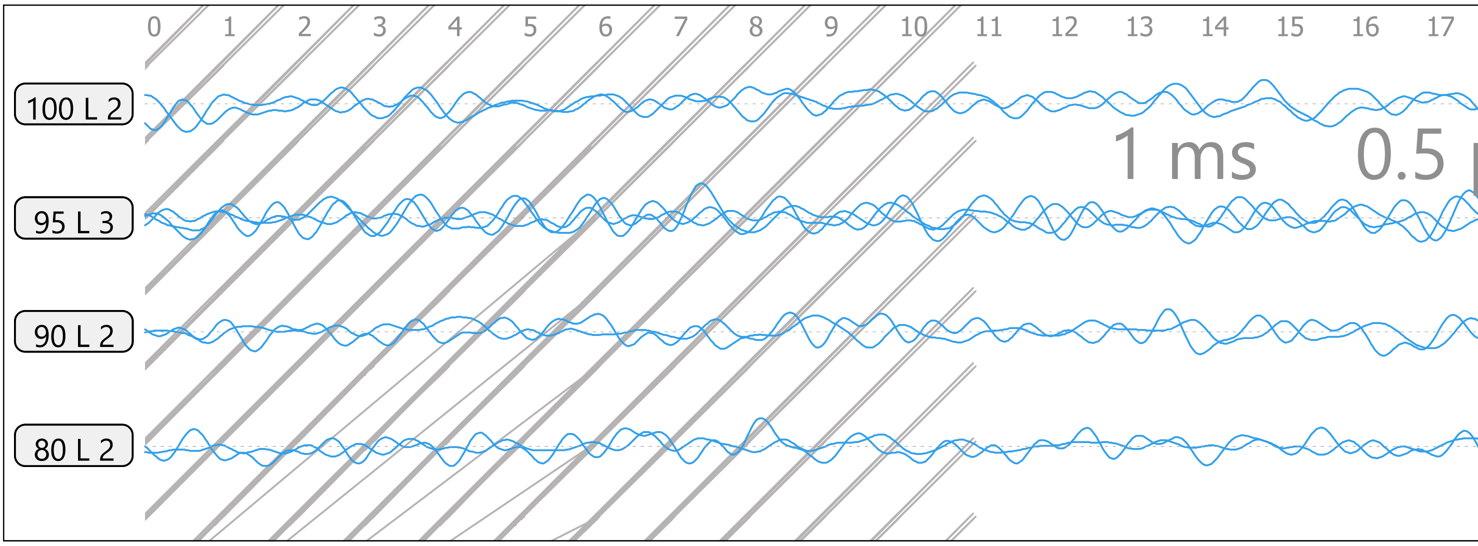

Trace parameters

N	Electr.	HPF, Hz	LPF, Hz	50 Hz	Rejection ±μV	Aver.	Rejec
100 L	Cz-M1	200	2000		10	1000	0
100 L 2	Cz-M1	200	2000		10	1000	0
95 L	Cz-M1	200	2000		10	1000	0
95 L 2	Cz-M1	200	2000		10	1000	0
95 L 3	Cz-M1	200	2000		10	1000	0
90 L	Cz-M1	200	2000		10	1000	0
90 L 2	Cz-M1	200	2000		10	1000	0
80 L	Cz-M1	200	2000		10	1000	0
80 L 2	Cz-M1	200	2000		10	1000	0

ABR: ABR 2 tone burst 8000Hz
1: Cz-M1

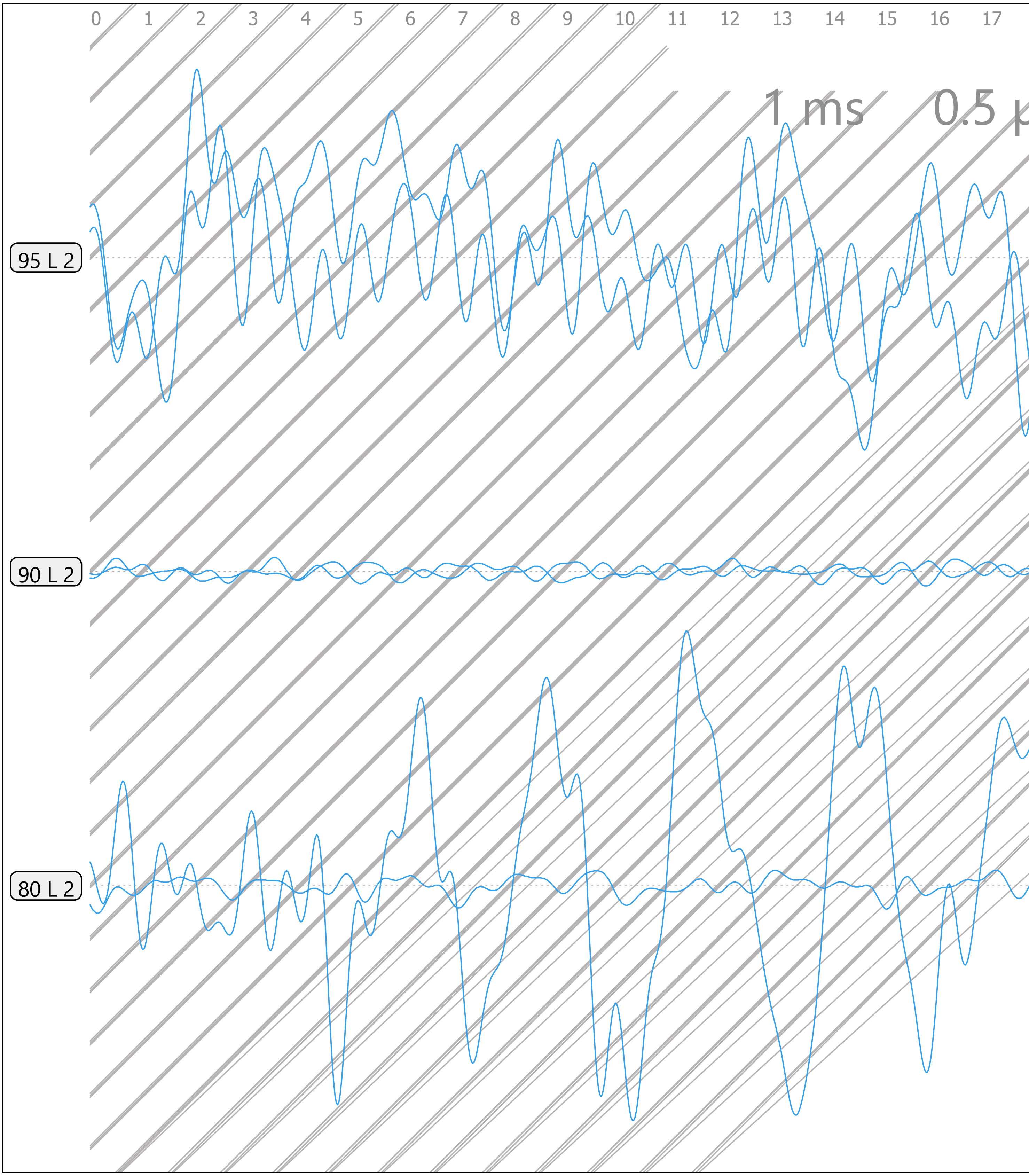

Trace parameters

N	Electr.	HPF, Hz	LPF, Hz	50 Hz	Rejection ±µV	Aver.	Reject.
95 L	Cz-M1	200	2000		10	1000	0
95 L 2	Cz-M1	200	2000		10	1000	0
90 L	Cz-M1	200	2000		10	1000	0

90 L 2	Cz-M1	200	2000		10	1000	0
80 L	Cz-M1	200	2000		10	1000	0
80 L 2	Cz-M1	200	2000		10	1000	0

CONCLUSION:

Doctor:
