# Supplementary material for: Exposure to high-altitude hypobaric hypoxic environment induces low-frequency hearing loss in C57BL/6J mice: Mediated by slowing down the postsynaptic electrical signal transmission speed in the cochlear-inferior colliculus auditory signaling pathway
Source: PLoS One. 2026 Mar 11;21(3):e0342321. doi: 10.1371/journal.pone.0342321 (PMC12978441; doi:10.1371/journal.pone.0342321)
Supplement: S1 File — (ZIP) [file pone.0342321.s001.zip › 2025.06.17-15d-04.pdf]

Exam report

Patient: 2025.06.17-15d-04- (-)
Date: June 18, 2025

ABR: ABR 2 CLICK
1: Cz-M1

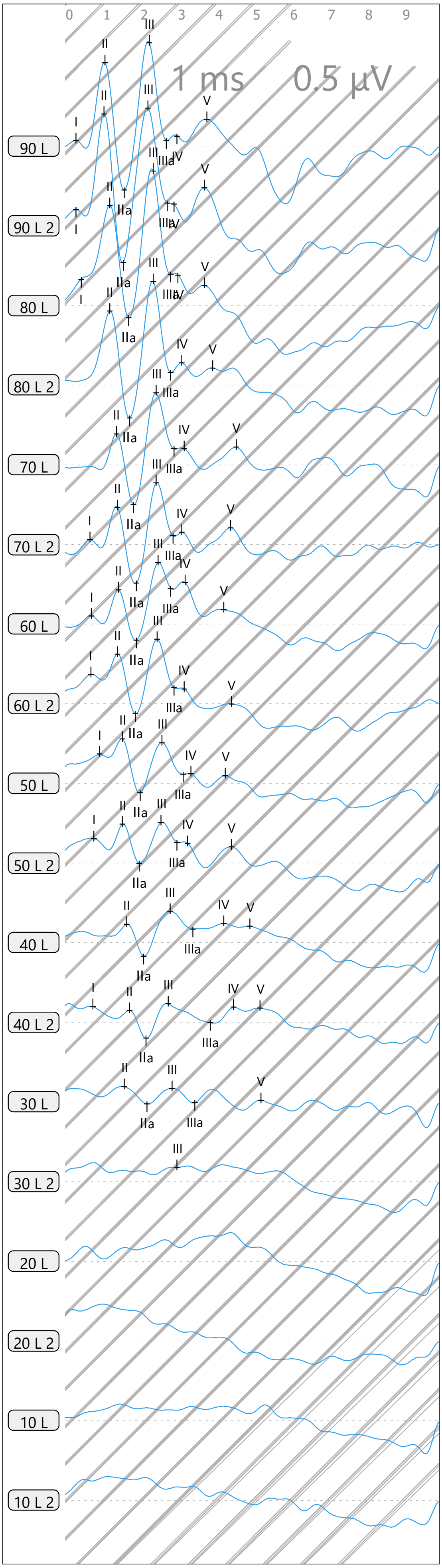
0 1 2 3 4 5 6 7 8 9
1 ms
0.5 µV
90 L
90 L 2
80 L
80 L 2
70 L
70 L 2
60 L
60 L 2
50 L
50 L 2
40 L
40 L 2
30 L
30 L 2
20 L
20 L 2
10 L
10 L 2

latency&& amplitude (左耳

N	I (ms)	II (ms)	III (ms)	IV (ms)	V (ms)
90 L	0.29	1.06	2.25	2.99	3.78
90 L 2	0.29	1.03	2.22	2.91	3.73
80 L	0.42	1.19	2.35	3.02	3.73
80 L 2		1.19	2.35	3.12	3.94
70 L		1.38	2.43	3.18	4.58
70 L 2	0.66	1.40	2.43	3.12	4.42
60 L	0.71	1.43	2.49	3.20	4.23
60 L 2	0.69	1.40	2.46	3.18	4.45
50 L	0.93	1.53	2.59	3.36	4.29
50 L 2	0.77	1.53	2.57	3.28	4.45
40 L		1.64	2.80	4.23	4.95
40 L 2	0.74	1.72	2.75	4.50	5.21
30 L		1.59	2.86		5.24
30 L 2			2.99		

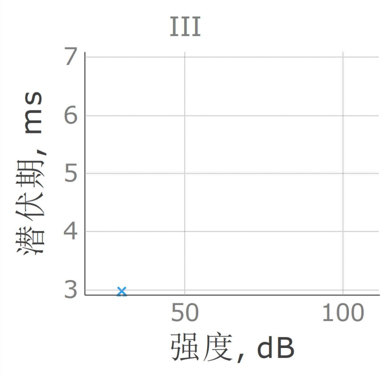

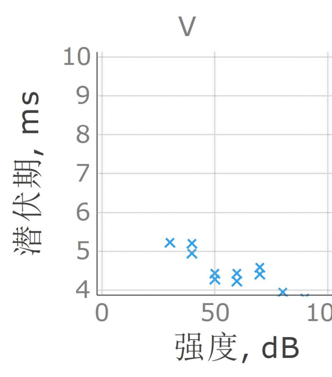

Trace parameters

N	Electr.	HPF, Hz	LPF, Hz	50 Hz	Rejection ±μV	Aver.	Reject.
90 L	Cz-M1	100	2000		10	1000	0
90 L 2	Cz-M1	100	2000		10	1000	0
80 L	Cz-M1	100	2000		10	1000	0
80 L 2	Cz-M1	100	2000		10	1000	0
70 L	Cz-M1	100	2000		10	1000	0
70 L 2	Cz-M1	100	2000		10	1000	0
60 L	Cz-M1	100	2000		10	1000	0
60 L 2	Cz-M1	100	2000		10	1000	0
50 L	Cz-M1	100	2000		10	1000	0
50 L 2	Cz-M1	100	2000		10	1000	0
40 L	Cz-M1	100	2000		10	1000	0
40 L 2	Cz-M1	100	2000		10	1000	0
30 L	Cz-M1	100	2000		10	1000	0
30 L 2	Cz-M1	100	2000		10	1000	0
20 L	Cz-M1	100	2000		10	1000	0
20 L 2	Cz-M1	100	2000		10	1000	0
10 L	Cz-M1	100	2000		10	1000	0
10 L 2	Cz-M1	100	2000		10	1000	0

ABR: ABR 2 tone burst 4000Hz 1
: Cz-M1

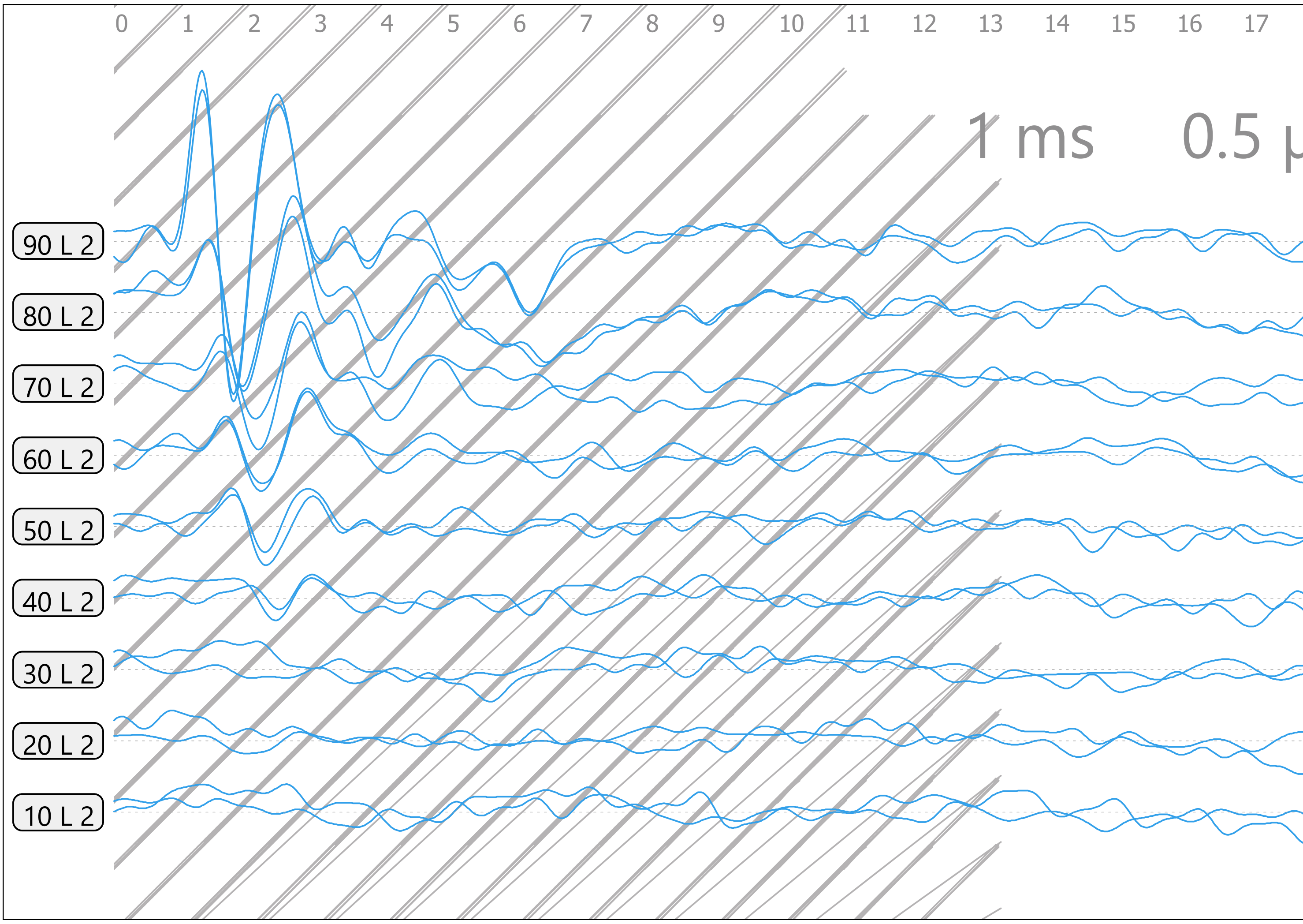

Trace parameters

N	Electr.	HPF, Hz	LPF, Hz	50 Hz	Rejection ±µV	Aver.	Reject.
90 L	Cz-M1	200	2000		10	1000	0
90 L 2	Cz-M1	200	2000		10	1000	0
80 L	Cz-M1	200	2000		10	1000	0
80 L 2	Cz-M1	200	2000		10	1000	0
70 L	Cz-M1	200	2000		10	1000	0
70 L 2	Cz-M1	200	2000		10	1000	0
60 L	Cz-M1	200	2000		10	1000	0
60 L 2	Cz-M1	200	2000		10	1000	0
50 L	Cz-M1	200	2000		10	1000	0
50 L 2	Cz-M1	200	2000		10	1000	0
40 L	Cz-M1	200	2000		10	1000	0
40 L 2	Cz-M1	200	2000		10	1000	0
30 L	Cz-M1	200	2000		10	1000	0
30 L 2	Cz-M1	200	2000		10	1000	0
20 L	Cz-M1	200	2000		10	1000	0

20 L 2	Cz-M1	200	2000		10	1000	0
10 L	Cz-M1	200	2000		10	1000	0
10 L 2	Cz-M1	200	2000		10	1000	0

ABR: ABR 2 tone burst 8000Hz 1
: Cz-M1

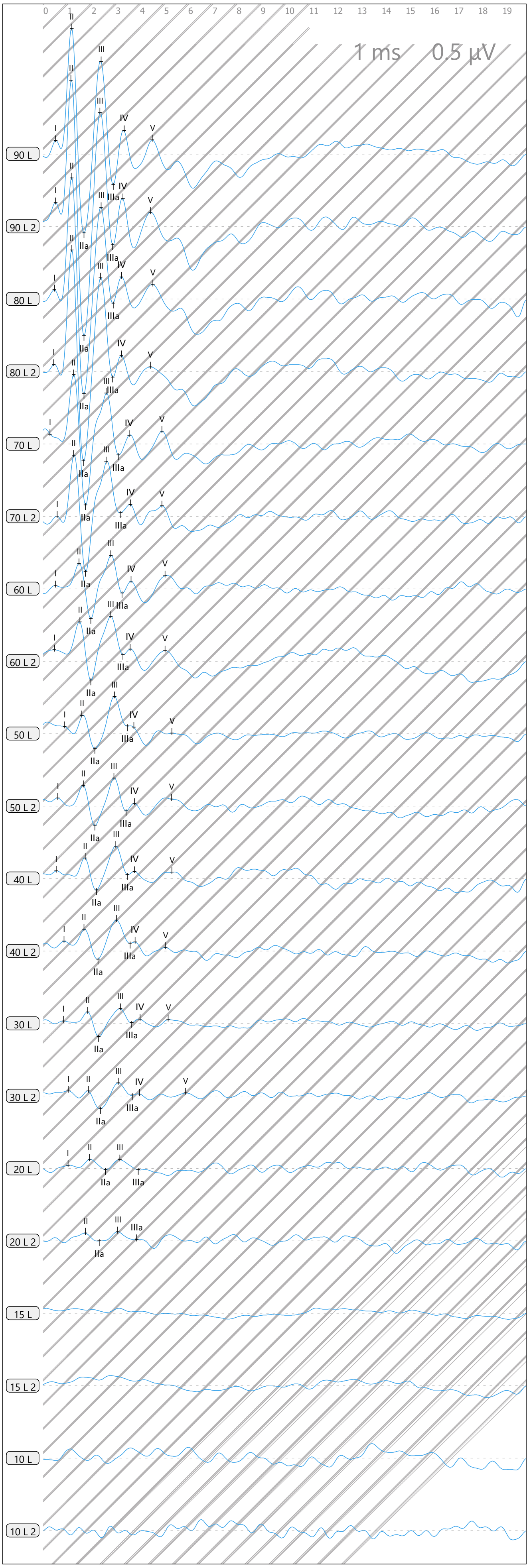
0 1 2 3 4 5 6 7 8 9 10 11 12 13 14 15 16 17 18 19
1 ms
0.5 µV
90 L
90 L 2
80 L
80 L 2
70 L
70 L 2
60 L
60 L 2
50 L
50 L 2
40 L
40 L 2
30 L
30 L 2
20 L
20 L 2
15 L
15 L 2
10 L
10 L 2

latency&& amplitude (left ear

N	I (ms)	II (ms)	III (ms)	IV (ms)	V (ms)
90 L	0.53	1.19	2.41	3.36	4.52
90 L 2	0.53	1.16	2.35	3.31	4.45
80 L	0.48	1.19	2.41	3.25	4.55
80 L 2	0.45	1.19	2.38	3.25	4.45
70 L	0.29	1.27	2.62	3.57	4.92
70 L 2	0.58	1.27	2.62	3.62	4.92
60 L	0.53	1.48	2.80	3.65	5.05
60 L 2	0.48	1.53	2.80	3.60	5.05
50 L	0.90	1.61	2.96	3.76	5.34
50 L 2	0.61	1.67	2.94	3.78	5.32
40 L	0.56	1.75	3.02	3.78	5.34
40 L 2	0.87	1.69	3.04	3.81	5.08
30 L	0.85	1.85	3.20	4.02	5.19
30 L 2	1.06	1.88	3.12	4.00	5.90
20 L	1.03	1.93	3.18		
20 L 2		1.77	3.10		

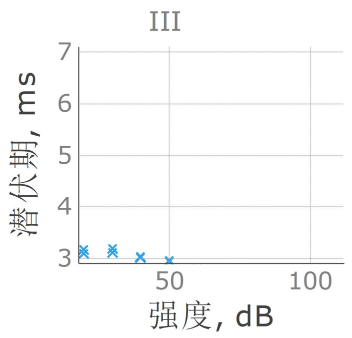

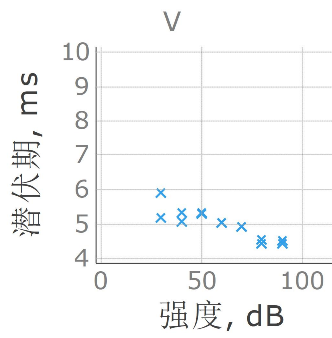

Trace parameters

N	Electr.	HPF, Hz	LPF, Hz	50 Hz	Rejection ±µV	Aver.	Reject.
90 L	Cz-M1	200	2000		10	1000	0
90 L 2	Cz-M1	200	2000		10	1000	0
80 L	Cz-M1	200	2000		10	1000	0
80 L 2	Cz-M1	200	2000		10	1000	0
70 L	Cz-M1	200	2000		10	1000	0
70 L 2	Cz-M1	200	2000		10	1000	0
60 L	Cz-M1	200	2000		10	1000	0
60 L 2	Cz-M1	200	2000		10	1000	0
50 L	Cz-M1	200	2000		10	1000	0
50 L 2	Cz-M1	200	2000		10	1000	0
40 L	Cz-M1	200	2000		10	1000	0
40 L 2	Cz-M1	200	2000		10	1000	0
30 L	Cz-M1	200	2000		10	1000	0
30 L 2	Cz-M1	200	2000		10	1000	0

20 L	Cz-M1	200	2000		10	1000	0
20 L 2	Cz-M1	200	2000		10	1000	0
15 L	Cz-M1	200	2000		10	1000	0
15 L 2	Cz-M1	200	2000		10	1000	0
10 L	Cz-M1	200	2000		10	1000	0
10 L 2	Cz-M1	200	2000		10	1000	0

ABR: ABR 2 CLICK 2: Cz-M2

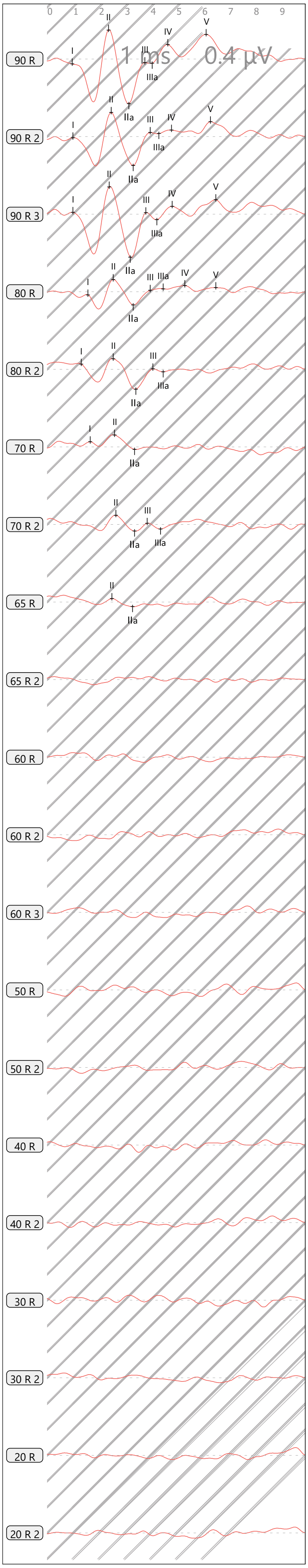
0 1 2 3 4 5 6 7 8 9
1 ms
0.4 µV
90 R
90 R 2
90 R 3
80 R
80 R 2
70 R
70 R 2
65 R
65 R 2
60 R
60 R 2
60 R 3
50 R
50 R 2
40 R
40 R 2
30 R
30 R 2
20 R
20 R 2
I
II
IIa
III
IIIa
IV
V

IV (ms)	V (ms)	I–III (ms)	I–V (ms)	III–V (ms)	
4.68	6.16	2.80	5.19	2.38	
4.82	6.32	2.99	5.32	2.33	
4.84	6.54	2.83	5.53	2.70	
5.34	6.54	2.41	4.95	2.54	
		2.78			

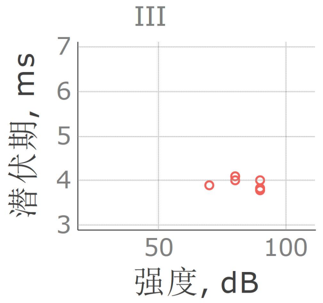

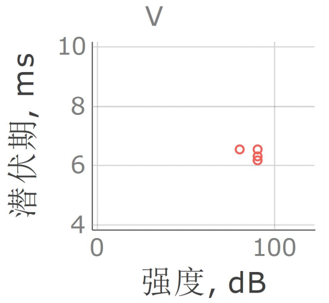

Trace parameters

N	Electr.	HPF, Hz	LPF, Hz	50 Hz	Rejection ±μV	Aver.	Reject
90 R	Cz-M2	100	2000		10	1000	0
90 R 2	Cz-M2	100	2000		10	1000	0
90 R 3	Cz-M2	100	2000		10	1000	0
80 R	Cz-M2	100	2000		10	1000	0
80 R 2	Cz-M2	100	2000		10	1000	0
70 R	Cz-M2	100	2000		10	1000	0
70 R 2	Cz-M2	100	2000		10	1000	0
65 R	Cz-M2	100	2000		10	1000	0
65 R 2	Cz-M2	100	2000		10	1000	0
60 R	Cz-M2	100	2000		10	1000	0
60 R 2	Cz-M2	100	2000		10	1000	0
60 R 3	Cz-M2	100	2000		10	1000	0
50 R	Cz-M2	100	2000		10	1000	0
50 R 2	Cz-M2	100	2000		10	1000	0
40 R	Cz-M2	100	2000		10	1000	0
40 R 2	Cz-M2	100	2000		10	1000	0
30 R	Cz-M2	100	2000		10	1000	0
30 R 2	Cz-M2	100	2000		10	1000	0
20 R	Cz-M2	100	2000		10	1000	0
20 R 2	Cz-M2	100	2000		10	1000	0

ABR: ABR 2 tone burst 4000Hz 2
: Cz-M2

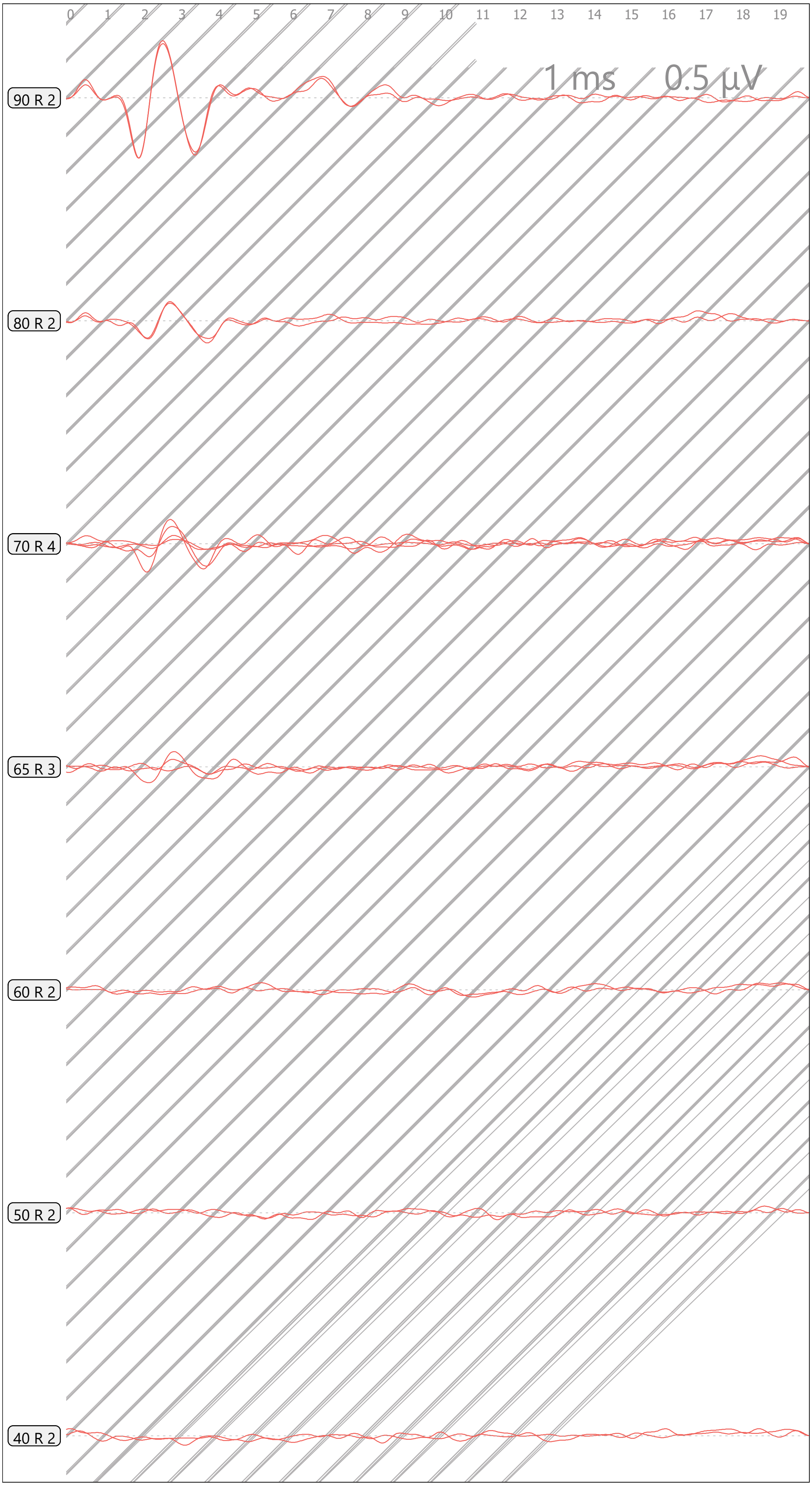
0 1 2 3 4 5 6 7 8 9 10 11 12 13 14 15 16 17 18 19
1 ms
0.5 µV
90 R 2
80 R 2
70 R 4
65 R 3
60 R 2
50 R 2
40 R 2

Trace parameters

N	Electr.	HPF, Hz	LPF, Hz	50 Hz	Rejection ±μV	Aver.	Reject
90 R	Cz-M2	200	2000		10	1000	0
90 R 2	Cz-M2	200	2000		10	1000	0
80 R	Cz-M2	200	2000		10	1000	0
80 R 2	Cz-M2	200	2000		10	1000	0
70 R	Cz-M2	200	2000		10	1000	0
70 R 2	Cz-M2	200	2000		10	1000	0
70 R 3	Cz-M2	200	2000		10	1000	0
70 R 4	Cz-M2	200	2000		10	1000	0
65 R	Cz-M2	200	2000		10	1000	0
65 R 2	Cz-M2	200	2000		10	1000	0
65 R 3	Cz-M2	200	2000		10	1000	0
60 R	Cz-M2	200	2000		10	1000	0
60 R 2	Cz-M2	200	2000		10	1000	0
50 R	Cz-M2	200	2000		10	1000	0
50 R 2	Cz-M2	200	2000		10	1000	0
40 R	Cz-M2	200	2000		10	1000	0
40 R 2	Cz-M2	200	2000		10	1000	0

ABR: ABR 2 tone burst 8000Hz 2
: Cz-M2

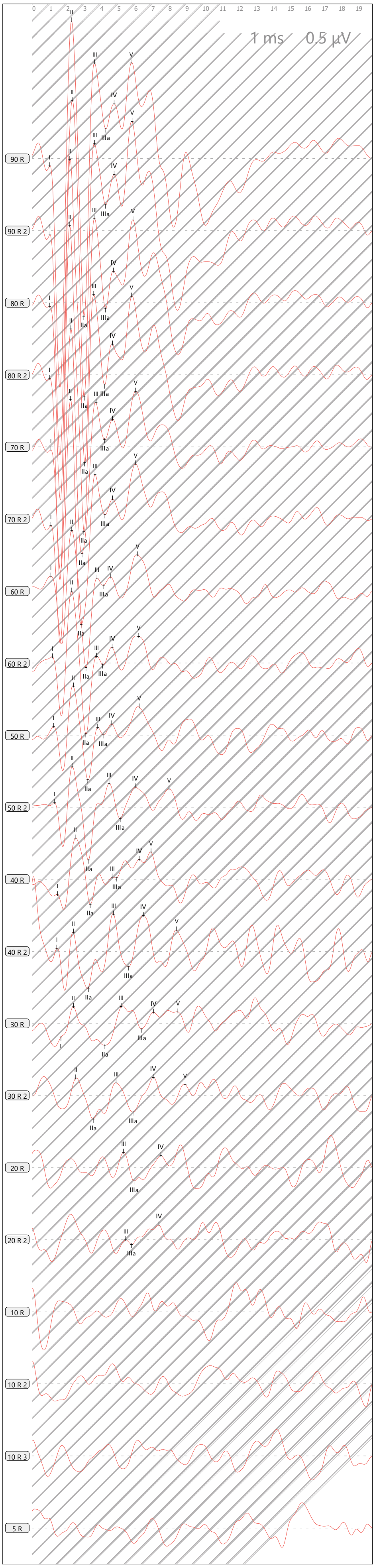
0 1 2 3 4 5 6 7 8 9 10 11 12 13 14 15 16 17 18 19
1 ms
0.5 µV
90 R
90 R 2
80 R
80 R 2
70 R
70 R 2
60 R
60 R 2
50 R
50 R 2
40 R
40 R 2
30 R
30 R 2
20 R
20 R 2
10 R
10 R 2
10 R 3
5 R

Ⅳ (ms)	V (ms)	I–III (ms)	I–V (ms)	III–V (ms)	
4.82	5.82	2.65	4.79	2.14	
4.82	5.87	2.62	4.82	2.20	
4.79	5.93	2.62	4.89	2.28	
4.74	5.85	2.59	4.82	2.22	
4.74	6.09	2.65	4.97	2.33	
4.74	6.09	2.59	4.97	2.38	
4.60	6.19	2.70	5.08	2.38	
4.71	6.27	2.59	5.08	2.49	
4.68	6.30	2.59	5.03	2.43	
6.06	8.04	3.20	6.72	3.52	
6.30	6.99	3.20	5.48	2.28	
6.54	8.49	3.33	7.04	3.70	
7.14	8.57	3.55	6.88	3.33	
7.12	9.00			4.05	
7.57					
7.46					

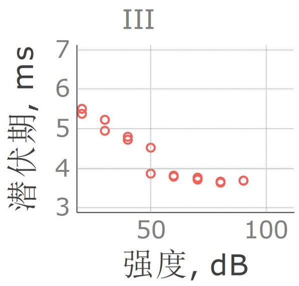

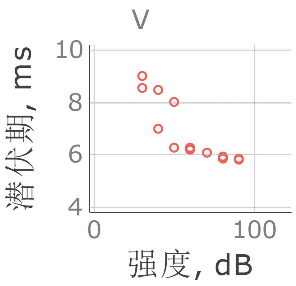

Trace parameters

N	Electr.	HPF, Hz	LPF, Hz	50 Hz	Rejection ±μV	Aver.	Reject.
90 R	Cz-M2	200	2000		10	1000	0
90 R 2	Cz-M2	200	2000		10	1000	0
80 R	Cz-M2	200	2000		10	1000	0
80 R 2	Cz-M2	200	2000		10	1000	0
70 R	Cz-M2	200	2000		10	1000	0
70 R 2	Cz-M2	200	2000		10	1000	0
60 R	Cz-M2	200	2000		10	1000	0
60 R 2	Cz-M2	200	2000		10	1000	0
50 R	Cz-M2	200	2000		10	1000	0
50 R 2	Cz-M2	200	2000		10	1000	0
40 R	Cz-M2	200	2000		10	1000	0
40 R 2	Cz-M2	200	2000		10	1000	0
30 R	Cz-M2	200	2000		10	1000	0
30 R 2	Cz-M2	200	2000		10	1000	0
20 R	Cz-M2	200	2000		10	1000	0

20 R 2	Cz-M2	200	2000		10	1000	0
10 R	Cz-M2	200	2000		10	1000	0
10 R 2	Cz-M2	200	2000		10	1000	0
10 R 3	Cz-M2	200	2000		10	1000	0
5 R	Cz-M2	200	2000		10	1000	0

ECochG: ECochG 1: Cz-M1

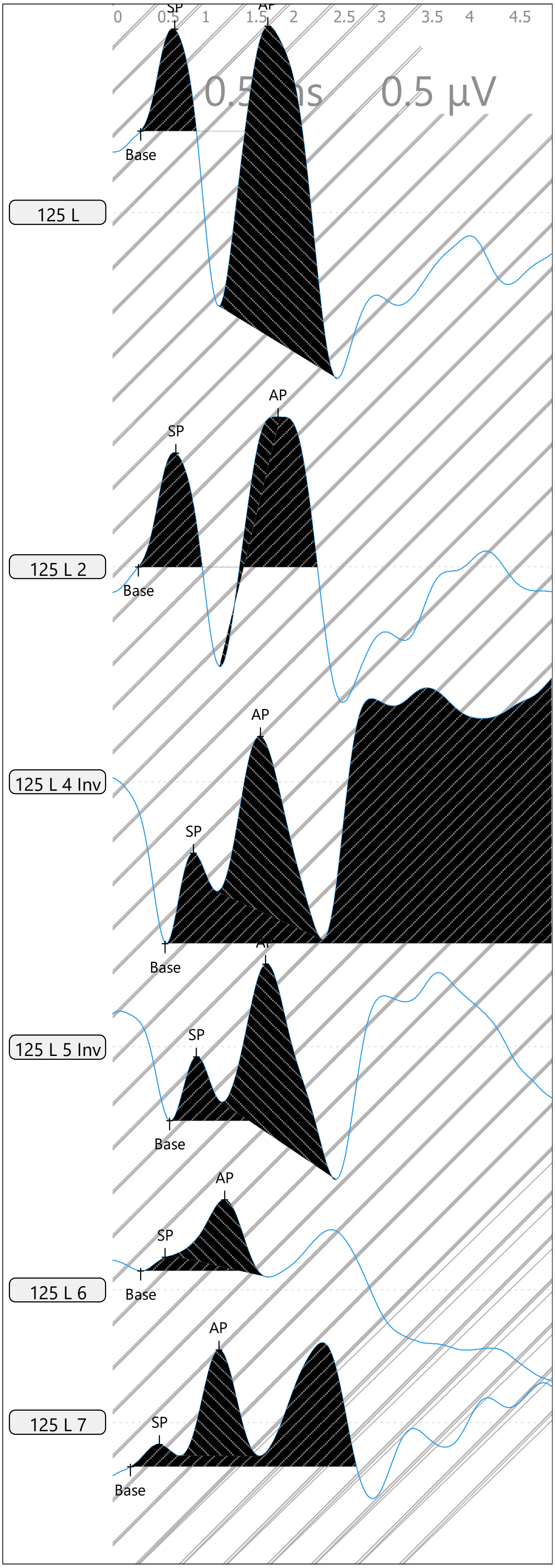

0
0.5
1
1.5
2
2.5
3
3.5
4
4.5
0.5 ms
0.5 µV
125 L
125 L 2
125 L 4 Inv
125 L 5 Inv
125 L 6
125 L 7
SP
AP
Base

&& (left ear

N	Base (ms)	SP (ms)	AP (ms)	SP–Base (ms)	AP–Base (ms)	SP–Base (µV)	AP–Base (µV)	[illegible]
125 L	0.32	0.70	1.76	0.38	1.44	1.16	1.19	[illegible]
125 L 2	0.29	0.71	1.88	0.42	1.59	1.29	1.71	[illegible]
125 L 4 Inv	0.60	0.91	1.68	0.32	1.08	1.02	2.35	[illegible]
125 L 5 Inv	0.65	0.95	1.73	0.30	1.08	0.73	1.78	[illegible]
125 L 6	0.32	0.60	1.27	0.28	0.95	0.15	0.81	[illegible]
125 L 7	0.20	0.53	1.20	0.33	1.01	0.26	1.33	[illegible]

Trace parameters

N	Electr.	HPF, Hz	LPF, Hz	50 Hz	Rejection ±µV	Aver.	[illegible]
125 L	Cz-M1	5	2000		50	1500	
125 L 2	Cz-M1	5	2000		50	1195	
125 L 4 Inv	Cz-M1	5	2000		50	1123	
125 L 5 Inv	Cz-M1	5	2000		50	296	
125 L 6	Cz-M1	5	2000		50	1182	
125 L 7	Cz-M1	5	2000		50	1047	

ECochG: ECochG 2:
Fpz-M2

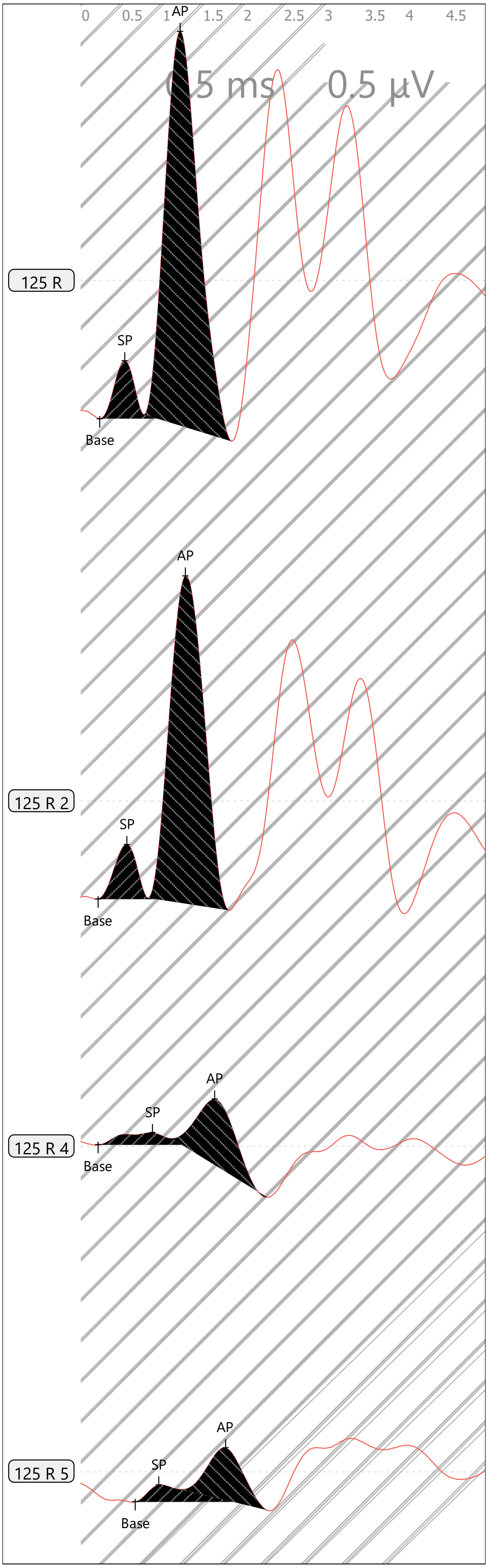

0
0.5
1
1.5
2
2.5
3
3.5
4
4.5
0.5 ms
0.5 µV
125 R
AP
SP
Base
125 R 2
AP
SP
Base
125 R 4
AP
SP
Base
125 R 5
AP
SP
Base

&& (right ear

N	Base (ms)	SP (ms)	AP (ms)	SP–Base (ms)	AP–Base (ms)	SP–Base (µV)	AP–Base (µV)	
125 R	0.24	0.54	1.23	0.30	0.99	0.71	4.78	0.15
125 R 2	0.21	0.57	1.30	0.36	1.08	0.67	3.99	0.17
125 R 4	0.21	0.89	1.65	0.67	1.44	0.15	0.56	0.27
125 R 5	0.67	0.97	1.79	0.29	1.11	0.21	0.66	0.31

Trace parameters

N	Electr.	HPF, Hz	LPF, Hz	50 Hz	Rejection ±µV	Aver.	Rejec
125 R	Fpz-M2	5	2000		50	1500	172
125 R 2	Fpz-M2	5	2000		50	1500	52
125 R 4	Fpz-M2	5	2000		50	1061	41
125 R 5	Fpz-M2	5	2000		50	1045	38

CONCLUSION:

Doctor:
