# Supplementary material for: Exposure to high-altitude hypobaric hypoxic environment induces low-frequency hearing loss in C57BL/6J mice: Mediated by slowing down the postsynaptic electrical signal transmission speed in the cochlear-inferior colliculus auditory signaling pathway
Source: PLoS One. 2026 Mar 11;21(3):e0342321. doi: 10.1371/journal.pone.0342321 (PMC12978441; doi:10.1371/journal.pone.0342321)
Supplement: S1 File — (ZIP) [file pone.0342321.s001.zip › 2025.5.22-01-normal.pdf]

Exam report

Patient: 2025.5.22-01-normal(-)
Date: May 22,2025

ABR: ABR 2 CLICK
1: Cz-M1

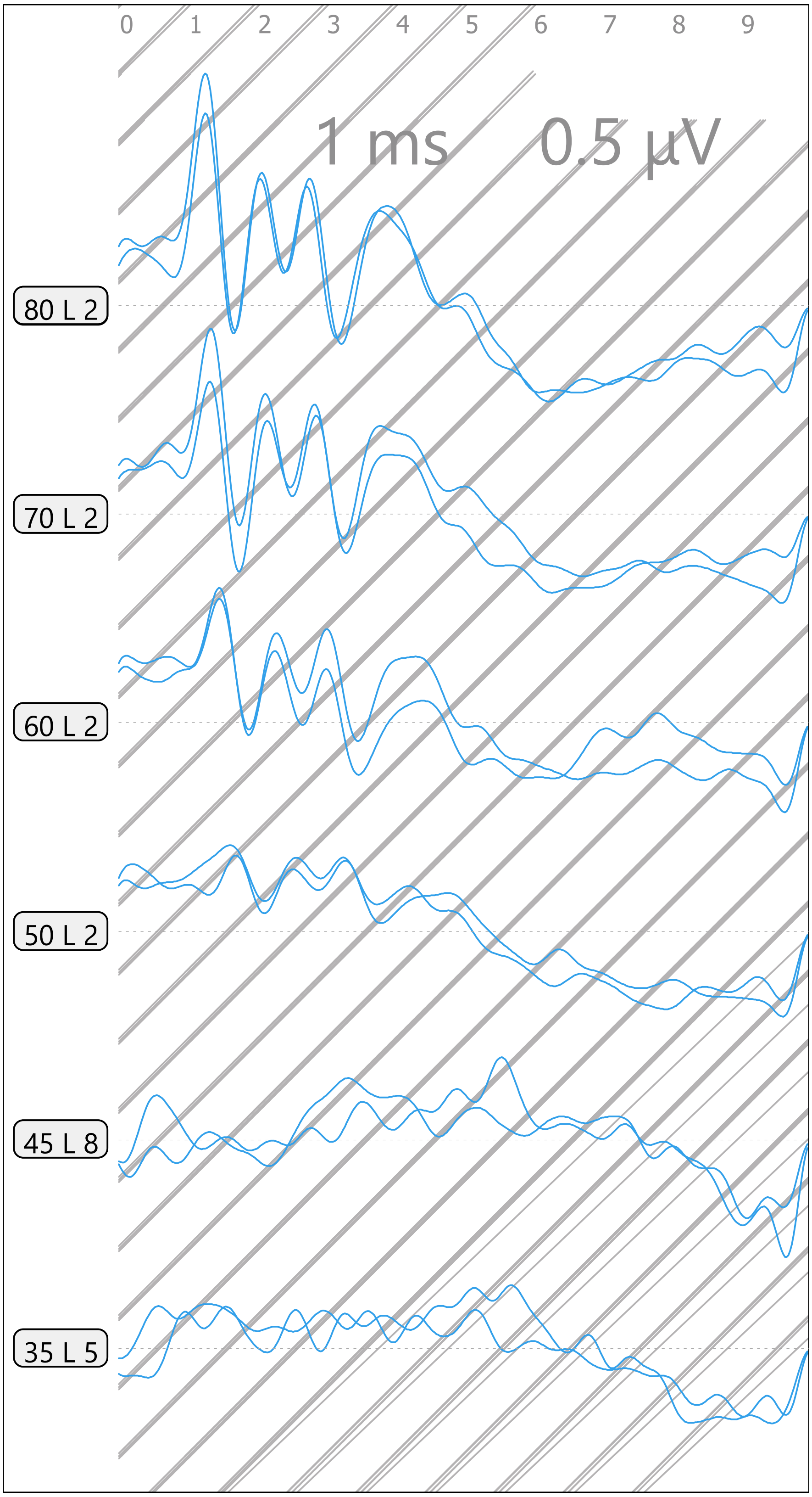

Trace parameters

N	Electr.	HPF, Hz	LPF, Hz	50 Hz	Rejection ±µV	Aver.	Reject.

80 L	Cz-M1	100	2000		10	1000	0
80 L 2	Cz-M1	100	2000		10	1000	0
70 L	Cz-M1	100	2000		10	1000	0
70 L 2	Cz-M1	100	2000		10	1000	0
60 L	Cz-M1	100	2000		10	1000	0
60 L 2	Cz-M1	100	2000		10	1000	0
50 L	Cz-M1	100	2000		10	1000	0
50 L 2	Cz-M1	100	2000		10	1000	0
45 L 7	Cz-M1	100	2000		80	1000	0
45 L 8	Cz-M1	100	2000		80	1000	0
35 L 4	Cz-M1	100	2000		80	846	0
35 L 5	Cz-M1	100	2000		80	1000	0

ABR: ABR 2 2000Hz

1: Cz-M1

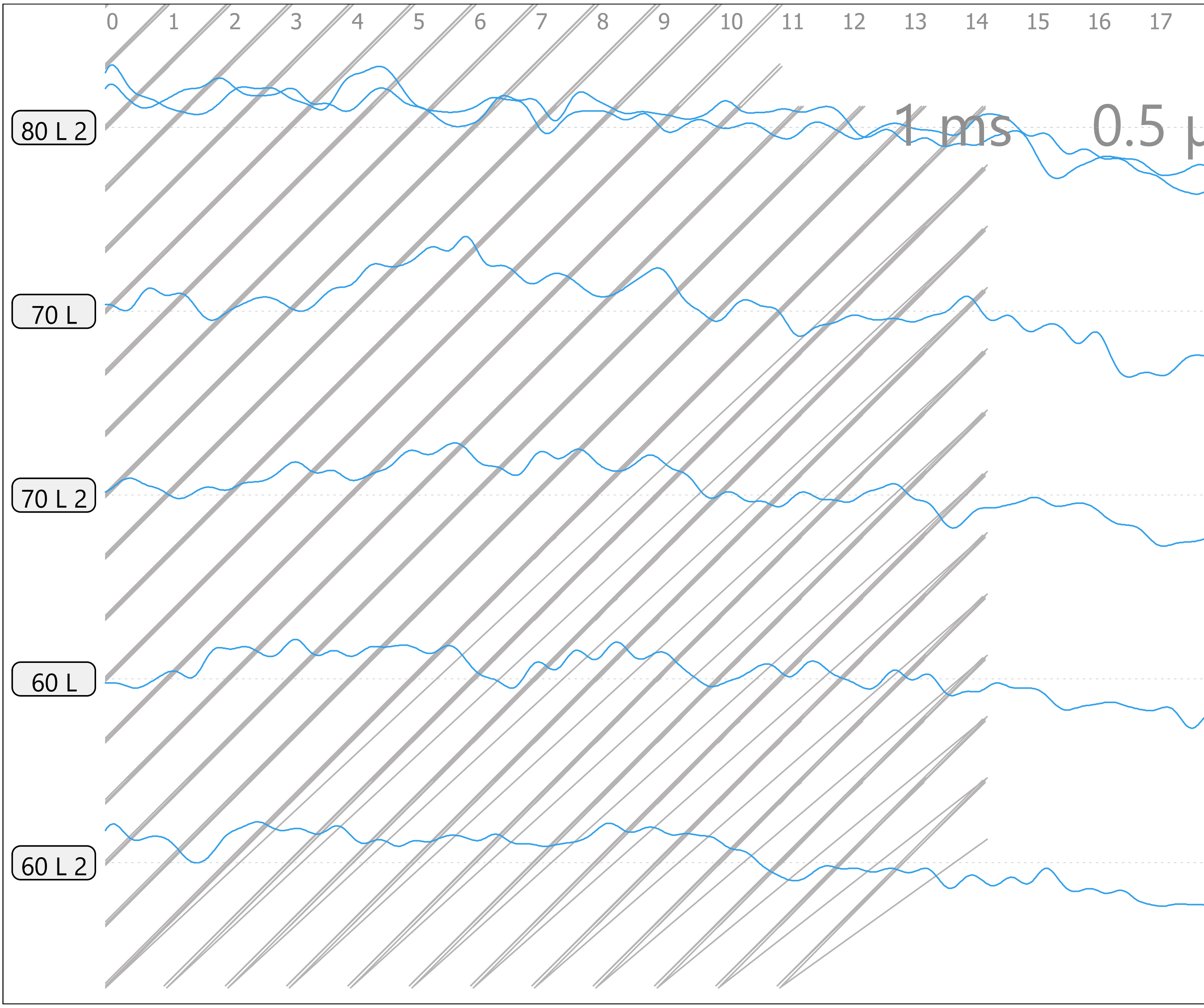

Trace parameters

N	Electr.	HPF, Hz	LPF, Hz	50 Hz	Rejection ±μV	Aver.	Reject.
80 L	Cz-M1	200	2000		10	1000	0
80 L 2	Cz-M1	200	2000		10	1000	0

70 L	Cz-M1	200	2000		10	1000	0
70 L 2	Cz-M1	200	2000		10	1000	0
60 L	Cz-M1	200	2000		10	1000	0
60 L 2	Cz-M1	200	2000		10	1000	0

ABR: ABR 2 8000Hz 1: Cz-M1

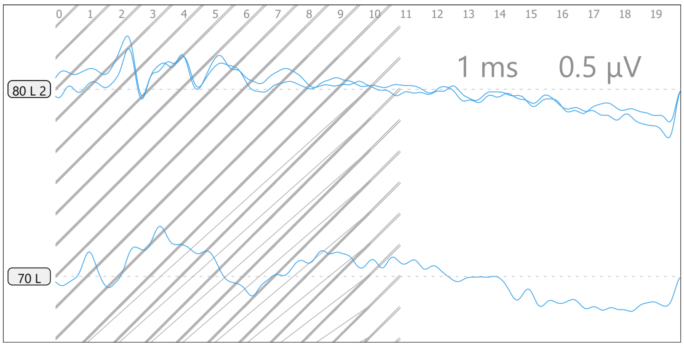

Trace parameters

N	Electr.	HPF, Hz	LPF, Hz	50 Hz	Rejection ±µV	Aver.	Reject.
80 L	Cz-M1	200	2000		10	1000	0
80 L 2	Cz-M1	200	2000		10	1000	0
70 L	Cz-M1	200	2000		10	1000	0

ABR: ABR 2 2000Hz 1: Cz-M1

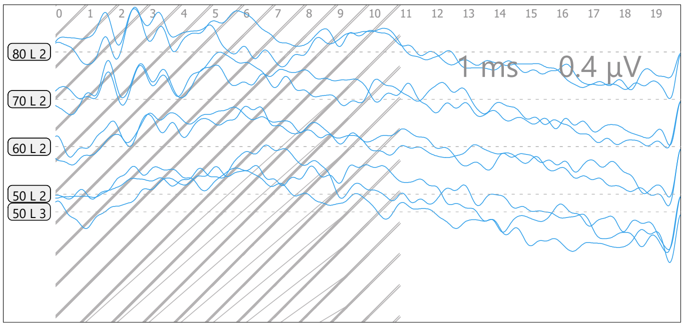

Trace parameters

N	Electr.	HPF, Hz	LPF, Hz	50 Hz	Rejection ±µV	Aver.	Reject.
80 L	Cz-M1	200	2000		10	1000	0
80 L 2	Cz-M1	200	2000		10	1000	0
70 L	Cz-M1	200	2000		10	1000	0
70 L 2	Cz-M1	200	2000		10	1000	0
60 L	Cz-M1	200	2000		10	1000	0
60 L 2	Cz-M1	200	2000		10	1000	0
50 L	Cz-M1	200	2000		10	1000	0
50 L 2	Cz-M1	200	2000		10	1000	0
50 L 3	Cz-M1	200	2000		10	1000	0

ABR: ABR 2 4000Hz 1: Cz-M1

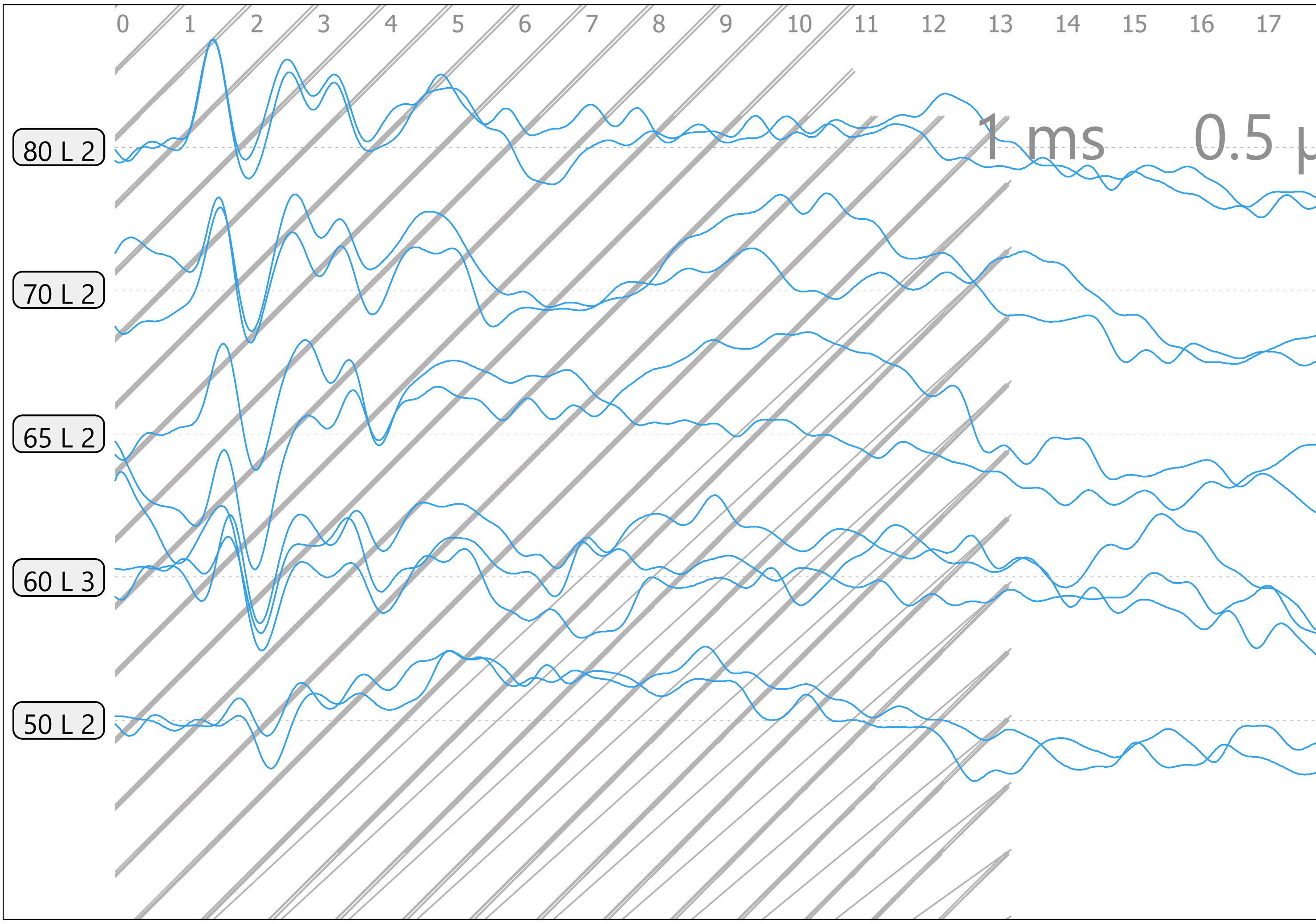

Trace parameters

N	Electr.	HPF, Hz	LPF, Hz	50 Hz	Rejection ±μV	Aver.	Reject.
80 L	Cz-M1	200	2000		10	1000	0
80 L 2	Cz-M1	200	2000		10	1000	0
70 L	Cz-M1	200	2000		10	1000	0
70 L 2	Cz-M1	200	2000		10	1000	0
65 L	Cz-M1	200	2000		10	1000	0
65 L 2	Cz-M1	200	2000		10	1000	0
60 L	Cz-M1	200	2000		10	1000	0
60 L 2	Cz-M1	200	2000		10	1000	0
60 L 3	Cz-M1	200	2000		10	1000	0
50 L	Cz-M1	200	2000		10	1000	0
50 L 2	Cz-M1	200	2000		10	1000	0

ABR: ABR 2 8000Hz 1: Cz-M1

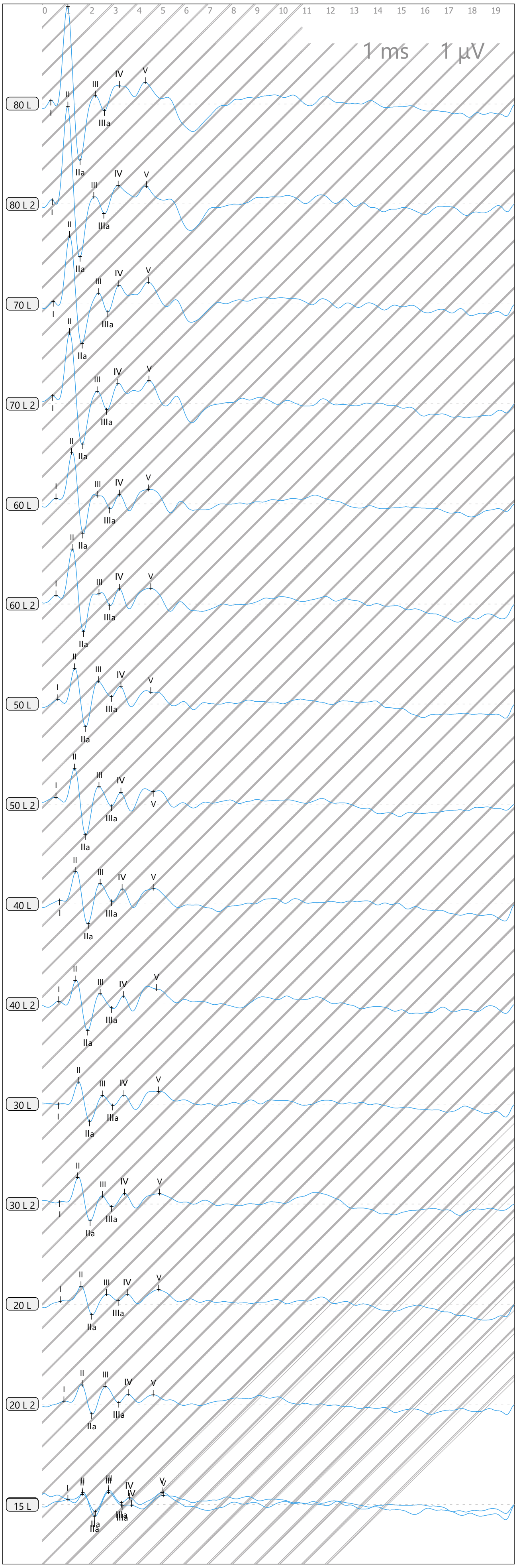

0 1 2 3 4 5 6 7 8 9 10 11 12 13 14 15 16 17 18 19
1 ms
1 µV
80 L
80 L 2
70 L
70 L 2
60 L
60 L 2
50 L
50 L 2
40 L
40 L 2
30 L
30 L 2
20 L
20 L 2
15 L

latency&& (left ear)

N	I (ms)	II (ms)	III (ms)	Ⅳ (ms)	V (ms)
80 L	0.37	1.08	2.25	3.28	4.37
80 L 2	0.42	1.08	2.20	3.23	4.42
70 L	0.48	1.16	2.38	3.25	4.50
70 L 2	0.45	1.16	2.33	3.20	4.52
60 L	0.58	1.24	2.35	3.28	4.50
60 L 2	0.58	1.27	2.41	3.28	4.60
50 L	0.66	1.38	2.38	3.33	4.60
50 L 2	0.58	1.38	2.41	3.33	4.71
40 L	0.74	1.40	2.46	3.39	4.71
40 L 2	0.71	1.40	2.46	3.44	4.84
30 L	0.69	1.53	2.54	3.47	4.92
30 L 2	0.74	1.51	2.57	3.49	4.97
20 L	0.77	1.64	2.73	3.60	4.95
20 L 2	0.93	1.69	2.67	3.65	4.71
20 L 3		1.69	2.83	3.70	5.08
15 L	1.08	1.72	2.80	3.78	5.13

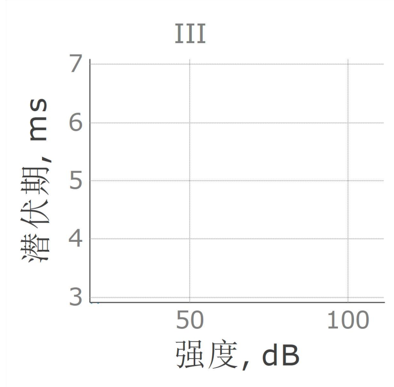

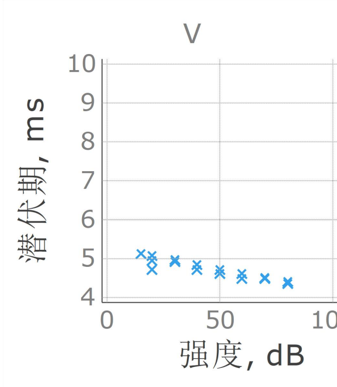

Trace parameters

N	Electr.	HPF, Hz	LPF, Hz	50 Hz	Rejection ±μV	Aver.	Reject.
80 L	Cz-M1	200	2000		10	1000	0
80 L 2	Cz-M1	200	2000		10	1000	0
70 L	Cz-M1	200	2000		10	1000	0
70 L 2	Cz-M1	200	2000		10	1000	0
60 L	Cz-M1	200	2000		10	1000	0
60 L 2	Cz-M1	200	2000		10	1000	0
50 L	Cz-M1	200	2000		10	1000	0
50 L 2	Cz-M1	200	2000		10	1000	0
40 L	Cz-M1	200	2000		10	1000	0
40 L 2	Cz-M1	200	2000		10	1000	0
30 L	Cz-M1	200	2000		10	1000	0
30 L 2	Cz-M1	200	2000		10	1000	0
20 L	Cz-M1	200	2000		10	1000	0

20 L 2	Cz-M1	200	2000		10	1000	0
20 L 3	Cz-M1	200	2000		10	1000	0
15 L	Cz-M1	200	2000		10	1000	0

DPOAE: 1-12 kHz 70/70 dB 3 points

Test result (right ear):

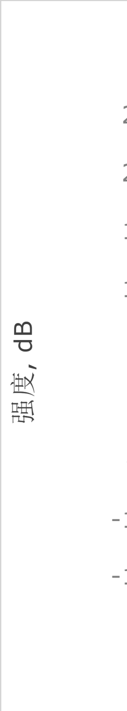

DPOAE (left ear)

F2, Hz	L1, dB	L2, dB	DP, dB	dB	SNR, dB	OAE
988	67.9	68.2	-15.08	-12.81	-2.3	✘
1270	68.6	69.0	-3.88	-1.45	-2.4	✘
1778	69.6	69.7	-12.81	-15.00	2.2	✘
2222	70.0	70.0	-12.41	-15.00	2.6	✘
2500	70.1	70.1	-12.68	-15.00	2.3	✘
3200	70.4	70.4	-19.11	-15.00	-4.1	✘
4444	70.9	70.7	-6.75	-14.35	7.6	✔
5000	70.9	70.4	-3.94	-10.74	6.8	✔
6154	70.7	70.6	-2.92	-9.49	6.6	✔
8000	70.4	70.3	8.40	-7.42	15.8	✔
8889	70.9	70.9	22.89	-7.72	30.6	✔
10000	70.5	63.8	24.50	-5.84	30.3	✔
11429	65.8	55.6	18.27	-2.74	21.0	✔
(dB SPL)	:: 0.0					

EcochG: ECochG 1:
Fpz-M1

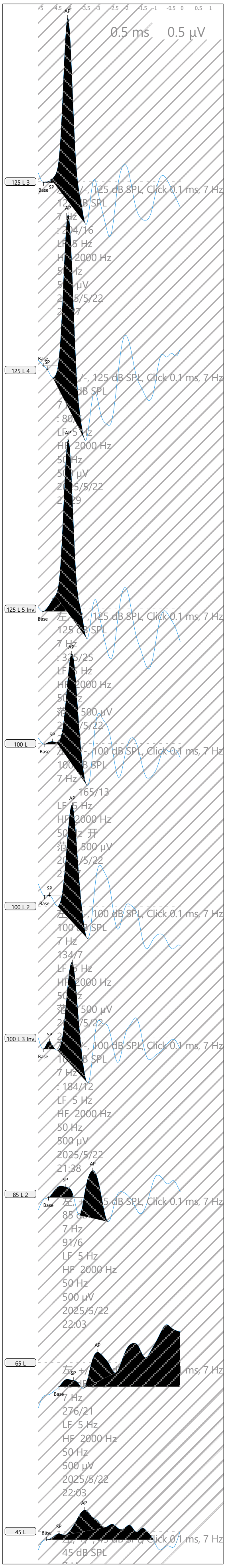
0.5 ms
0.5 µV
125 L 3
125 L 4
125 L 5 Inv
100 L
100 L 2
100 L 3 Inv
85 L 2
65 L
45 L
LF 5 Hz
HF 2000 Hz
50 Hz
500 µV
2025/5/22
21:38
22:03
45 dB SPL

latency && (left ear

N	Base (ms)	SP (ms)	AP (ms)	SP–Base (ms)	AP–Base (ms)	SP–Base (µV)	AP–Base (µV)	
125 L 3	0.19	0.46	1.03	0.28	0.85	0.10	5.78	0
125 L 4	0.17	0.32	1.05	0.15	0.87	0.12	5.29	0
125 L 5 Inv	0.16	0.52	1.05	0.36	0.89	0.44	6.03	0
100 L	0.22	0.49	1.16	0.26	0.94	0.09	3.17	0
100 L 2	0.21	0.40	1.19	0.19	0.98	0.01	3.17	0
100 L 3 Inv	0.17	0.40	1.19	0.22	1.02	0.26	3.04	0
85 L 2	0.36	0.95	1.92	0.60	1.56	0.37	0.94	0
65 L	0.73	1.23	2.09	0.50	1.36	0.22	1.21	0
45 L	0.29	0.73	1.61	0.44	1.32	0.21	1.04	0

Trace parameters

N	Electr.	HPF, Hz	LPF, Hz	50 Hz	Rejection ±µV	Aver.	R
125 L 3	Fpz-M1	5	2000		50	204	
125 L 4	Fpz-M1	5	2000		50	80	
125 L 5 Inv	Fpz-M1	5	2000		50	325	
100 L	Fpz-M1	5	2000		50	165	
100 L 2	Fpz-M1	5	2000		50	134	
100 L 3 Inv	Fpz-M1	5	2000		50	184	
85 L 2	Fpz-M1	5	2000		50	91	
65 L	Fpz-M1	5	2000		50	276	
45 L	Fpz-M1	5	2000		50	117	

CONCLUSION:

Doctor:
