# Supplementary material for: Exposure to high-altitude hypobaric hypoxic environment induces low-frequency hearing loss in C57BL/6J mice: Mediated by slowing down the postsynaptic electrical signal transmission speed in the cochlear-inferior colliculus auditory signaling pathway
Source: PLoS One. 2026 Mar 11;21(3):e0342321. doi: 10.1371/journal.pone.0342321 (PMC12978441; doi:10.1371/journal.pone.0342321)
Supplement: S1 File — (ZIP) [file pone.0342321.s001.zip › 2025.5.22-02-normal.pdf]

Exam report

Patient: 2025.5.22-02, - (-)
Date: May 22, 2025

ABR: ABR 2 CLICK 1: Cz-M1

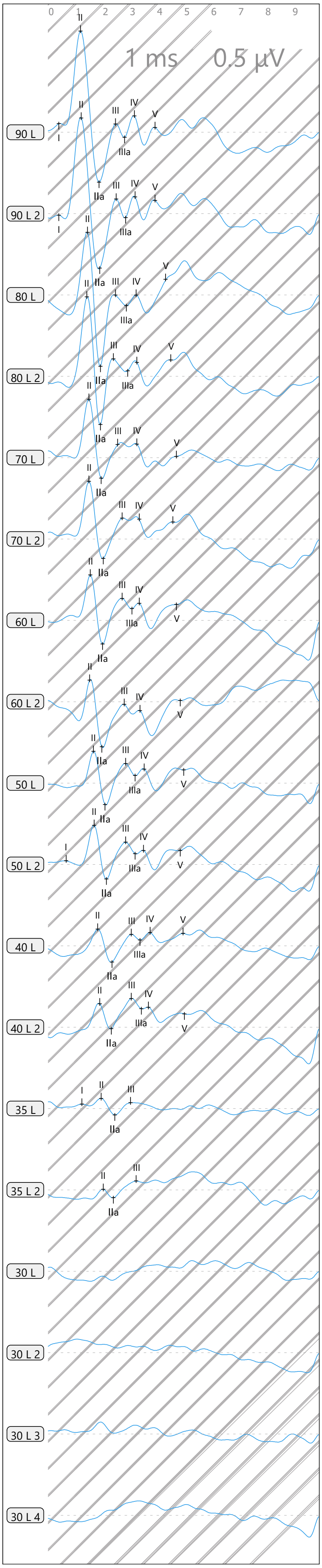

1 ms
0.5 µV
90 L
90 L 2
80 L
80 L 2
70 L
70 L 2
60 L
60 L 2
50 L
50 L 2
40 L
40 L 2
35 L
35 L 2
30 L
30 L 2
30 L 3
30 L 4

latency && (left ear

N	I (ms)	II (ms)	III (ms)	IV (ms)	V (ms)
90 L	0.40	1.19	2.49	3.18	3.94
90 L 2	0.40	1.22	2.51	3.20	3.94
80 L		1.46	2.49	3.25	4.34
80 L 2		1.43	2.41	3.28	4.52
70 L		1.51	2.57	3.28	4.74
70 L 2		1.51	2.73	3.36	4.60
60 L		1.56	2.73	3.36	4.74
60 L 2		1.53	2.80	3.39	4.87
50 L		1.67	2.86	3.55	5.00
50 L 2	0.66	1.69	2.86	3.52	4.87
40 L		1.83	3.07	3.76	4.97
40 L 2		1.91	3.07	3.70	5.03
35 L	1.24	1.96	3.04		
35 L 2		2.04	3.25		

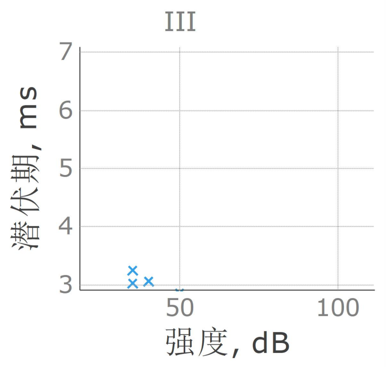

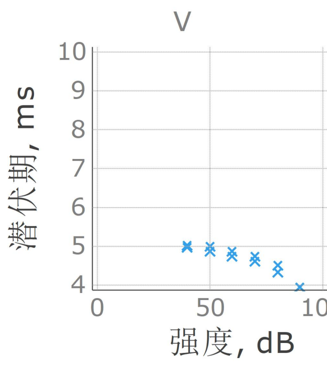

Trace parameters

N	Electr.	HPF, Hz	LPF, Hz	50 Hz	Rejection ±μV	Aver.	Reject.
90 L	Cz-M1	100	2000		10	1024	0
90 L 2	Cz-M1	100	2000		10	1024	0
80 L	Cz-M1	100	2000		10	1024	0
80 L 2	Cz-M1	100	2000		10	1024	0
70 L	Cz-M1	100	2000		10	1024	0
70 L 2	Cz-M1	100	2000		10	1024	0
60 L	Cz-M1	100	2000		10	1024	0
60 L 2	Cz-M1	100	2000		10	1024	0
50 L	Cz-M1	100	2000		10	1024	0
50 L 2	Cz-M1	100	2000		10	1024	0
40 L	Cz-M1	100	2000		10	1024	0
40 L 2	Cz-M1	100	2000		10	1024	0
35 L	Cz-M1	100	2000		10	1024	0
35 L 2	Cz-M1	100	2000		10	1024	0
30 L	Cz-M1	100	2000		10	1024	0
30 L 2	Cz-M1	100	2000		10	893	0
30 L 3	Cz-M1	100	2000		10	1024	0
30 L 4	Cz-M1	100	2000		10	1024	0

ABR: ABR 2 4000Hz 1: Cz-M1

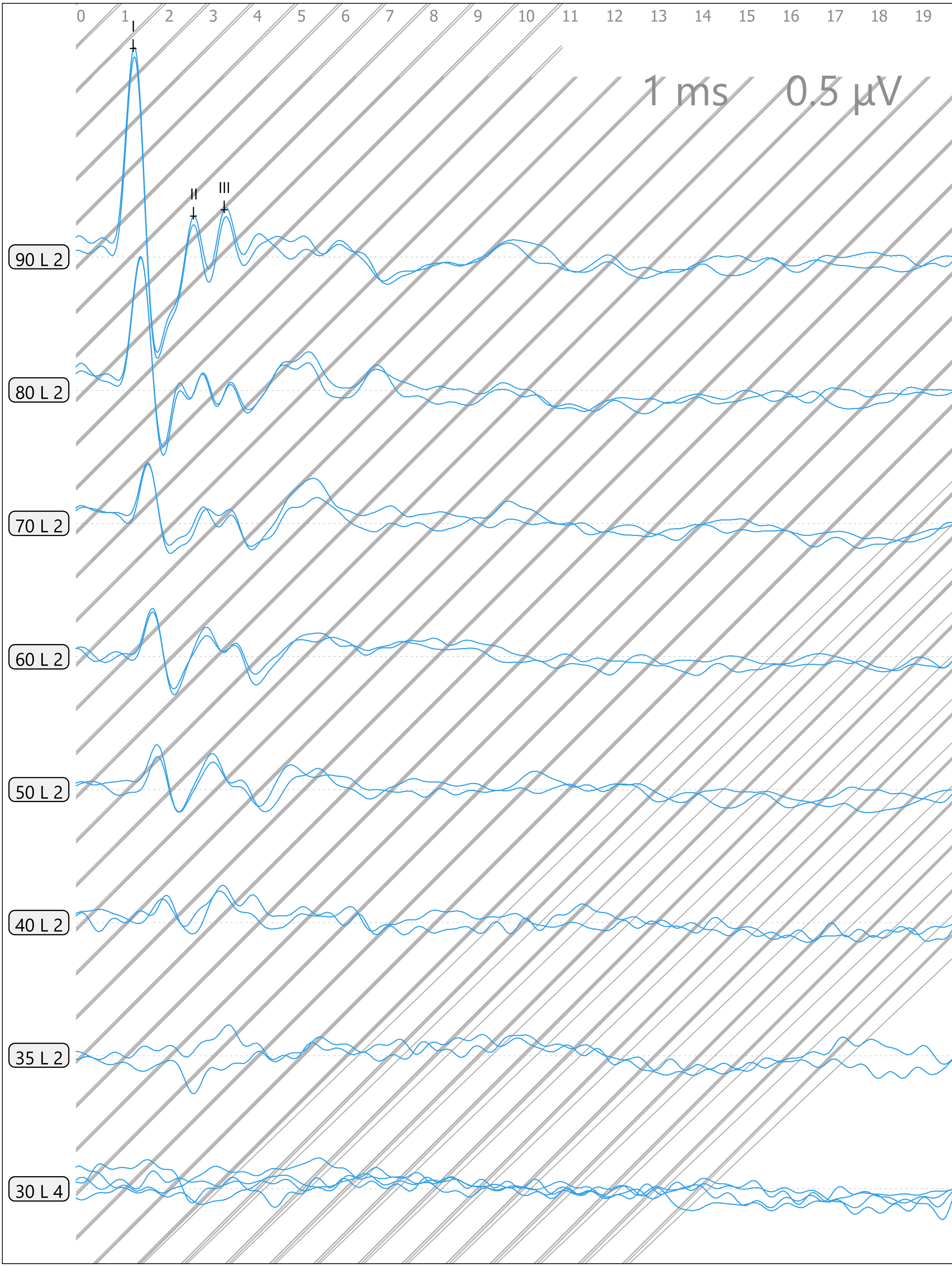
0 1 2 3 4 5 6 7 8 9 10 11 12 13 14 15 16 17 18 19
1 ms
0.5 µV
I
II
III
90 L 2
80 L 2
70 L 2
60 L 2
50 L 2
40 L 2
35 L 2
30 L 4

&&　　　(left ear

N	II (ms)	I (ms)	III (ms)	V (ms)
90 L 2	2.67	1.30	3.36	

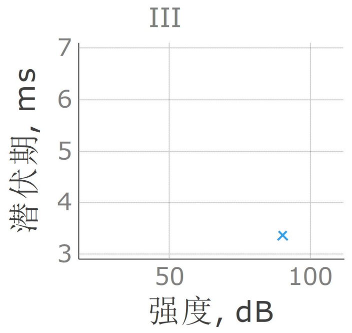

Trace parameters

N	Electr.	HPF, Hz	LPF, Hz	50 Hz	Rejection ±µV	Aver.	Reject.
90 L	Cz-M1	200	2000		10	1000	0
90 L 2	Cz-M1	200	2000		10	1000	0
80 L	Cz-M1	200	2000		10	1000	0
80 L 2	Cz-M1	200	2000		10	1000	0
70 L	Cz-M1	200	2000		10	1000	0
70 L 2	Cz-M1	200	2000		10	1000	0
60 L	Cz-M1	200	2000		10	1000	0
60 L 2	Cz-M1	200	2000		10	1000	0
50 L	Cz-M1	200	2000		10	1000	0
50 L 2	Cz-M1	200	2000		10	1000	0
40 L	Cz-M1	200	2000		10	1000	0
40 L 2	Cz-M1	200	2000		10	1000	0
35 L	Cz-M1	200	2000		10	1000	0
35 L 2	Cz-M1	200	2000		10	1000	0
30 L	Cz-M1	200	2000		10	1000	0
30 L 2	Cz-M1	200	2000		10	1000	0
30 L 3	Cz-M1	200	2000		10	1000	0
30 L 4	Cz-M1	200	2000		10	1000	0

ABR: ABR 2 6000Hz 1: Cz-M1

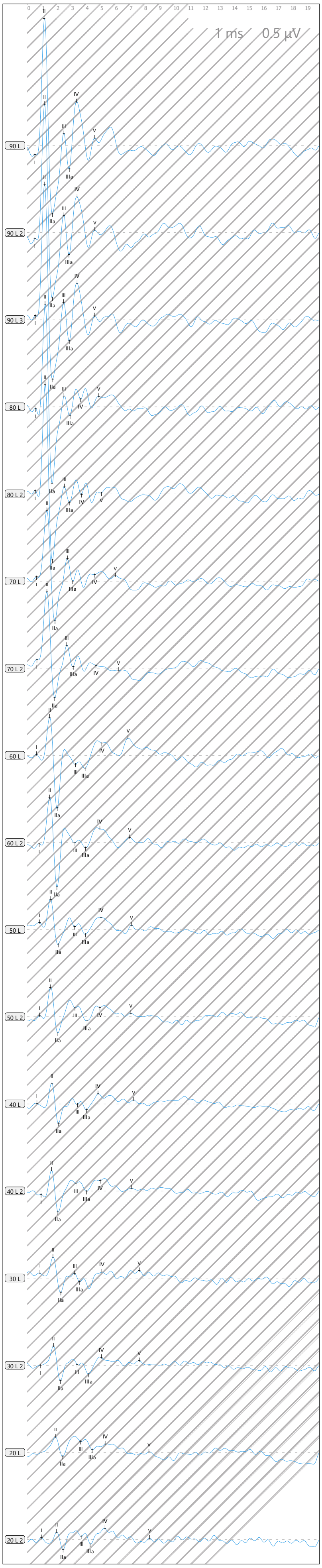
0 1 2 3 4 5 6 7 8 9 10 11 12 13 14 15 16 17 18 19
1 ms
0.5 µV
90 L
90 L 2
90 L 3
80 L
80 L 2
70 L
70 L 2
60 L
60 L 2
50 L
50 L 2
40 L
40 L 2
30 L
30 L 2
20 L
20 L 2

latency && amplitude) (left ear

N	I (ms)	II (ms)	III (ms)	IV (ms)	V (ms
90 L	0.53	1.16	2.51	3.36	4.60
90 L 2	0.53	1.19	2.51	3.41	4.63
90 L 3	0.56	1.19	2.49	3.41	4.60
80 L	0.58	1.22	2.51	3.65	4.89
80 L 2	0.56	1.22	2.54	3.73	5.08
70 L	0.64	1.35	2.75	4.63	6.03
70 L 2	0.66	1.35	2.70	4.71	6.24
60 L	0.64	1.53	3.31	5.11	6.91
60 L 2	0.82	1.53	3.28	4.97	7.01
50 L	0.85	1.61	3.25	5.05	7.14
50 L 2	0.85	1.59	3.28	4.97	7.09
40 L	0.66	1.69	3.44	4.84	7.28
40 L 2	0.95	1.67	3.33	5.00	7.14
30 L	0.87	1.77	3.25	5.11	7.67
30 L 2	0.90	1.80	3.41	5.08	7.67
20 L		1.93	3.65	5.34	8.33
20 L 2	0.98	2.01	3.70	5.32	8.39

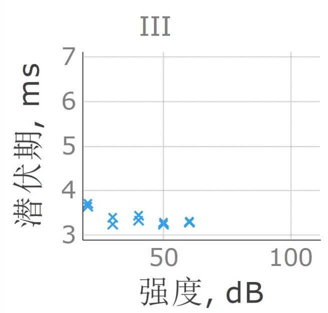

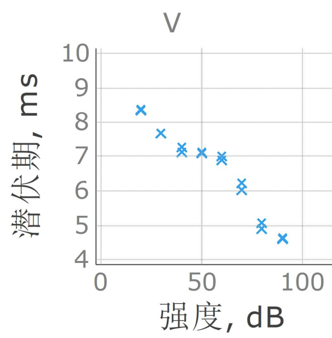

Trace parameters

N	Electr.	HPF, Hz	LPF, Hz	50 Hz	Rejection ±μV	Aver.	Reject.
90 L	Cz-M1	200	2000		10	1000	0
90 L 2	Cz-M1	200	2000		10	1000	0
90 L 3	Cz-M1	200	2000		10	1000	0
80 L	Cz-M1	200	2000		10	1000	0
80 L 2	Cz-M1	200	2000		10	1000	0
70 L	Cz-M1	200	2000		10	1000	0
70 L 2	Cz-M1	200	2000		10	1000	0
60 L	Cz-M1	200	2000		10	1000	0
60 L 2	Cz-M1	200	2000		10	961	0
50 L	Cz-M1	200	2000		10	1000	0
50 L 2	Cz-M1	200	2000		10	1000	0
40 L	Cz-M1	200	2000		10	1000	0
40 L 2	Cz-M1	200	2000		10	1000	0

30 L	Cz-M1	200	2000		10	1000	0
30 L 2	Cz-M1	200	2000		10	1000	0
20 L	Cz-M1	200	2000		10	1000	0
20 L 2	Cz-M1	200	2000		10	1000	0

ABR: ABR 2 tone burst 8000Hz 1
: Cz-M1

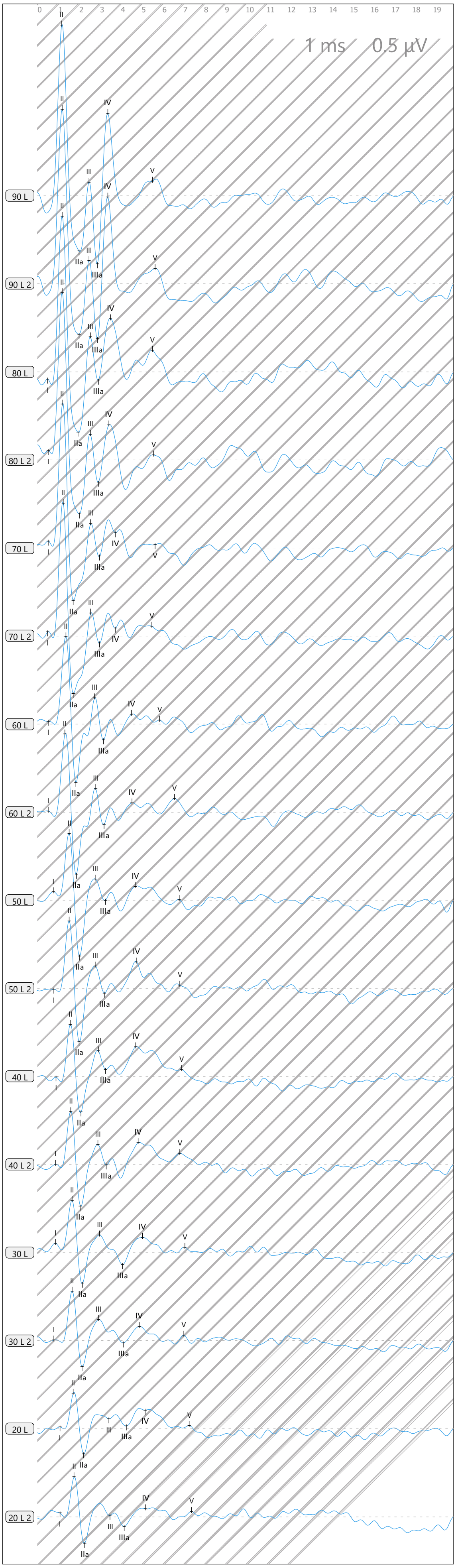
0 1 2 3 4 5 6 7 8 9 10 11 12 13 14 15 16 17 18 19
1 ms
0.5 µV
90 L
90 L 2
80 L
80 L 2
70 L
70 L 2
60 L
60 L 2
50 L
50 L 2
40 L
40 L 2
30 L
30 L 2
20 L
20 L 2

&& (left ear

N	I (ms)	II (ms)	III (ms)	IV (ms)	V (ms)
90 L		1.16	2.49	3.39	5.53
90 L 2		1.19	2.49	3.39	5.66
80 L	0.50	1.19	2.54	3.52	5.53
80 L 2	0.53	1.19	2.54	3.44	5.58
70 L	0.53	1.19	2.57	3.76	5.66
70 L 2	0.50	1.24	2.57	3.76	5.50
60 L	0.53	1.38	2.75	4.52	5.87
60 L 2	0.53	1.32	2.80	4.55	6.59
50 L	0.77	1.53	2.78	4.71	6.83
50 L 2	0.79	1.53	2.78	4.76	6.85
40 L	0.90	1.59	2.94	4.74	6.93
40 L 2	0.87	1.61	2.91	4.84	6.85
30 L	0.87	1.67	2.99	5.05	7.09
30 L 2	0.79	1.67	2.94	4.89	7.04
20 L	1.08	1.72	3.44	5.19	7.30
20 L 2	1.08	1.77	3.49	5.21	7.41

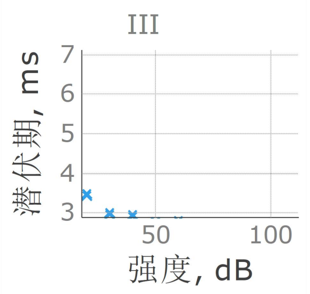

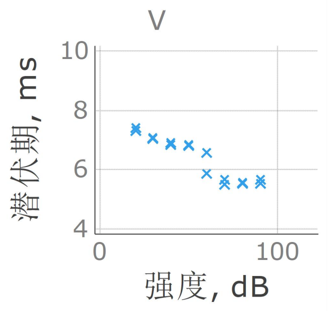

Trace parameters

N	Electr.	HPF, Hz	LPF, Hz	50 Hz	Rejection ±μV	Aver.	Reject.
90 L	Cz-M1	200	2000		10	1000	0
90 L 2	Cz-M1	200	2000		10	1000	0
80 L	Cz-M1	200	2000		10	1000	0
80 L 2	Cz-M1	200	2000		10	1000	0
70 L	Cz-M1	200	2000		10	1000	0
70 L 2	Cz-M1	200	2000		10	1000	0
60 L	Cz-M1	200	2000		10	1000	0
60 L 2	Cz-M1	200	2000		10	1000	0
50 L	Cz-M1	200	2000		10	1000	0
50 L 2	Cz-M1	200	2000		10	1000	0
40 L	Cz-M1	200	2000		10	1000	0
40 L 2	Cz-M1	200	2000		10	1000	0
30 L	Cz-M1	200	2000		10	1000	0
30 L 2	Cz-M1	200	2000		10	1000	0

20 L	Cz-M1	200	2000		10	1000	0
20 L 2	Cz-M1	200	2000		10	1000	0

DPOAE: 1-12 kHz 70/70 dB 3 points

Test result (right ear):

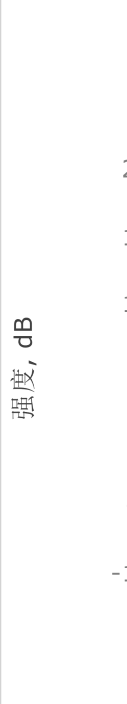

DPOAE

F2, Hz	L1, dB	L2, dB	DP, dB	噪声, dB	SNR, dB	OAE
988	68.1	68.4	-0.37	15.14	-15.5	✘
1270	68.8	69.1	3.47	11.98	-8.5	✘
1778	69.6	69.7	1.64	2.43	-0.8	✘
2222	70.0	70.0	-6.06	-12.11	6.1	✔
2500	70.1	70.1	-5.06	-11.31	6.3	✔
3200	70.4	70.4	-1.95	-2.33	0.4	✘
4444	70.8	70.6	-5.93	-0.34	-5.6	✘
5000	70.9	70.5	-0.28	-7.75	7.5	✔
6154	70.6	70.7	4.43	-1.63	6.1	✔
8000	70.6	69.7	-5.12	-13.52	8.4	✔
8889	70.7	70.4	11.23	-10.49	21.7	✔
10000	69.2	64.6	23.90	-9.69	33.6	✔
11429	67.5	59.3	20.16	-6.88	27.0	✔
noise level (dB SPL)	:: 0.0					

ECochG: ECochG 2: Cz-M2

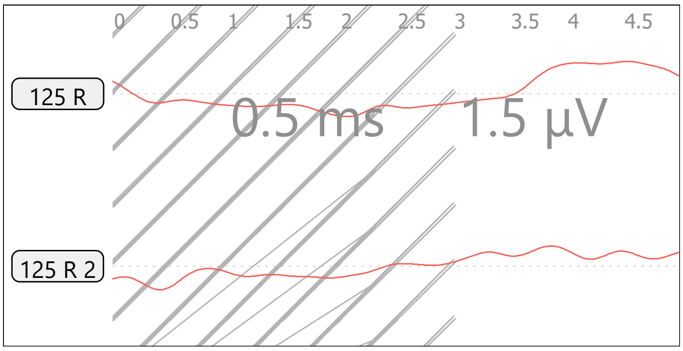

Trace parameters

N	Electr.	HPF, Hz	LPF, Hz	50 Hz	Rejection ±µV	Aver.	Rejec
125 R	Fpz-M1	5	2000		50	1500	68
125 R 2	Fpz-M1	5	2000		50	1500	62

CONCLUSION:

Doctor:
