# Supplementary material for: Exposure to high-altitude hypobaric hypoxic environment induces low-frequency hearing loss in C57BL/6J mice: Mediated by slowing down the postsynaptic electrical signal transmission speed in the cochlear-inferior colliculus auditory signaling pathway
Source: PLoS One. 2026 Mar 11;21(3):e0342321. doi: 10.1371/journal.pone.0342321 (PMC12978441; doi:10.1371/journal.pone.0342321)
Supplement: S1 File — (ZIP) [file pone.0342321.s001.zip › 2025.5.22-03 - normal.pdf]

Auditory Evoked Potential Test Report

2025.5.22-03, - normal (-)   May 23, 2025 **ABR**: ABR 2 CLI
CK 1: Cz-M1

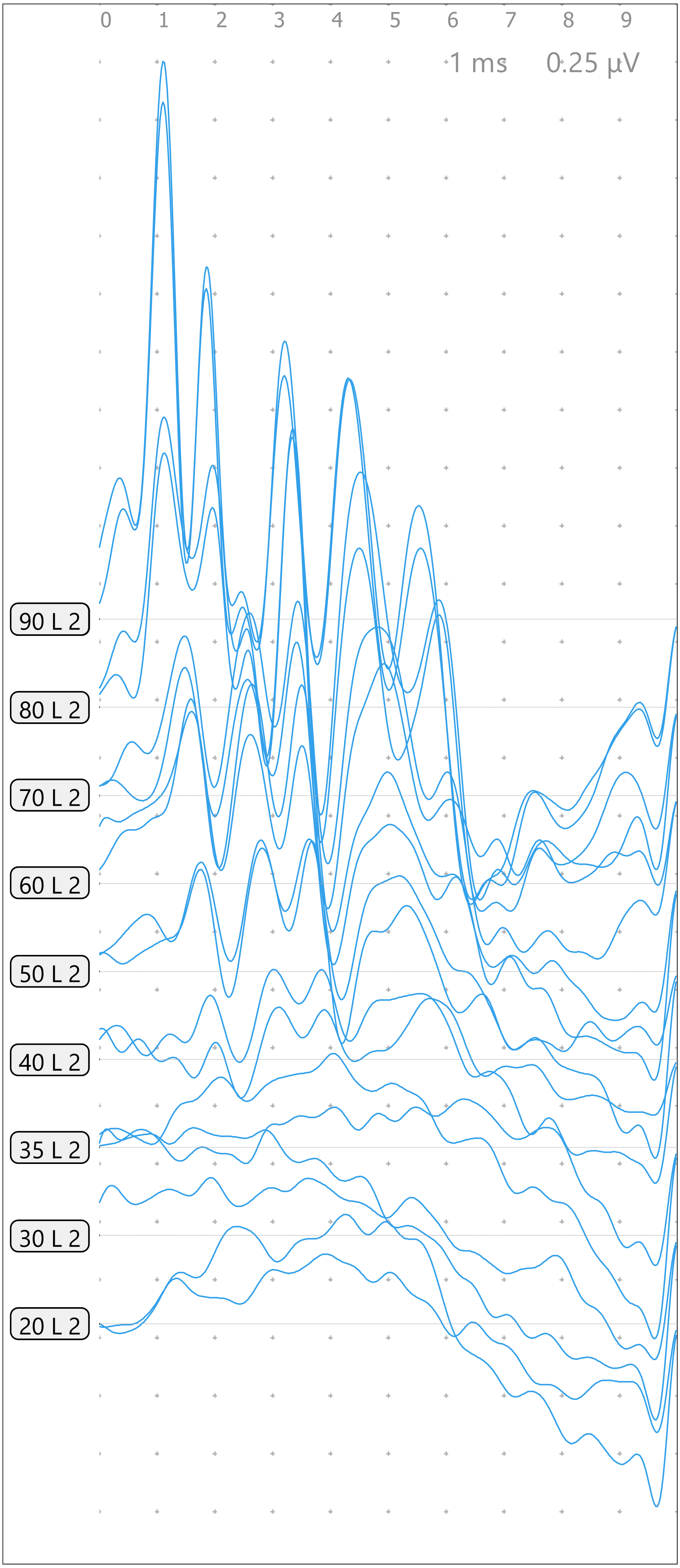

2025.5.22-03 2025/5/23

ABR: ABR 2 tone burst 4000Hz 1

: Cz-M1

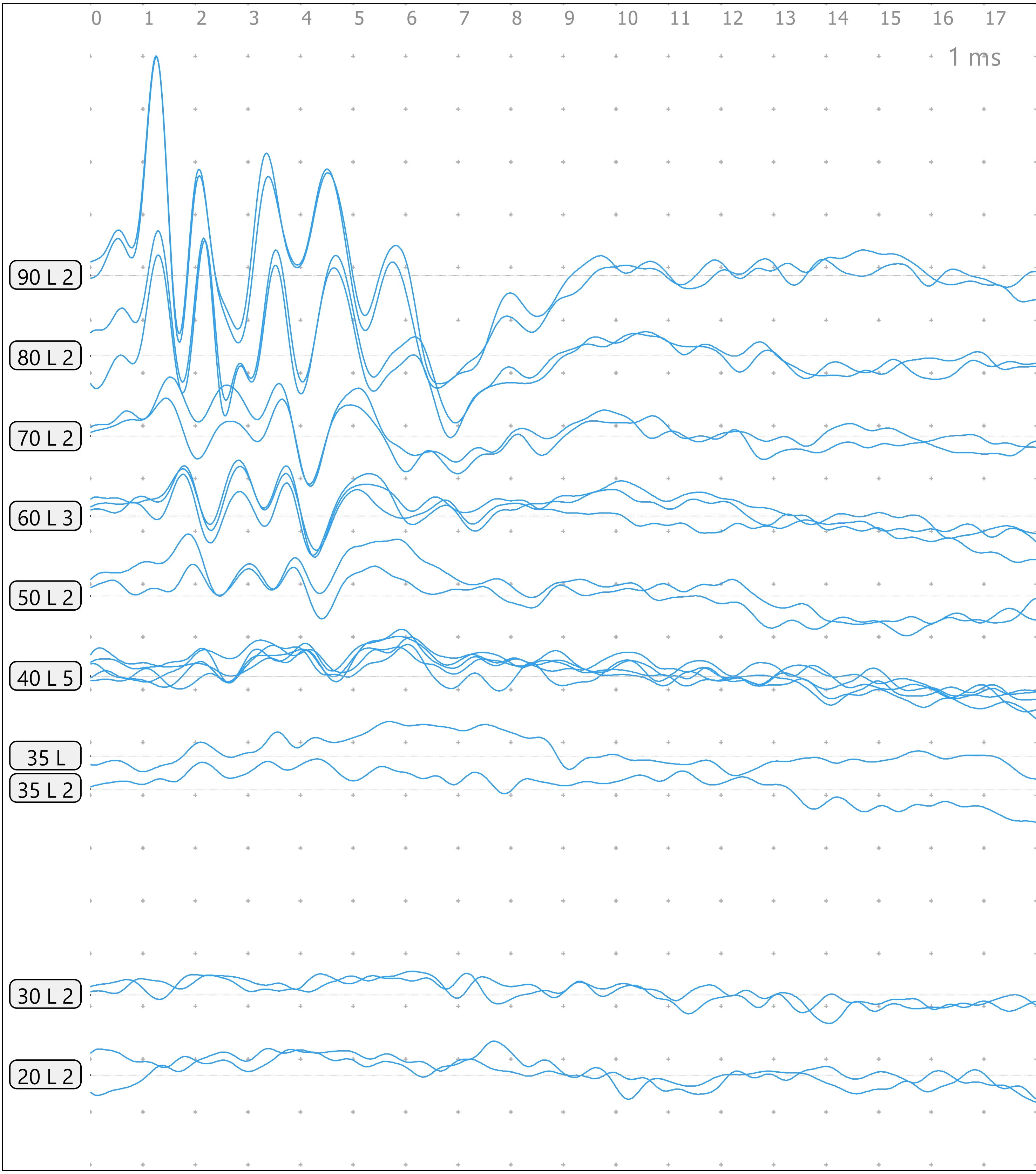

ABR: ABR 2 6000Hz 1: Cz-M1

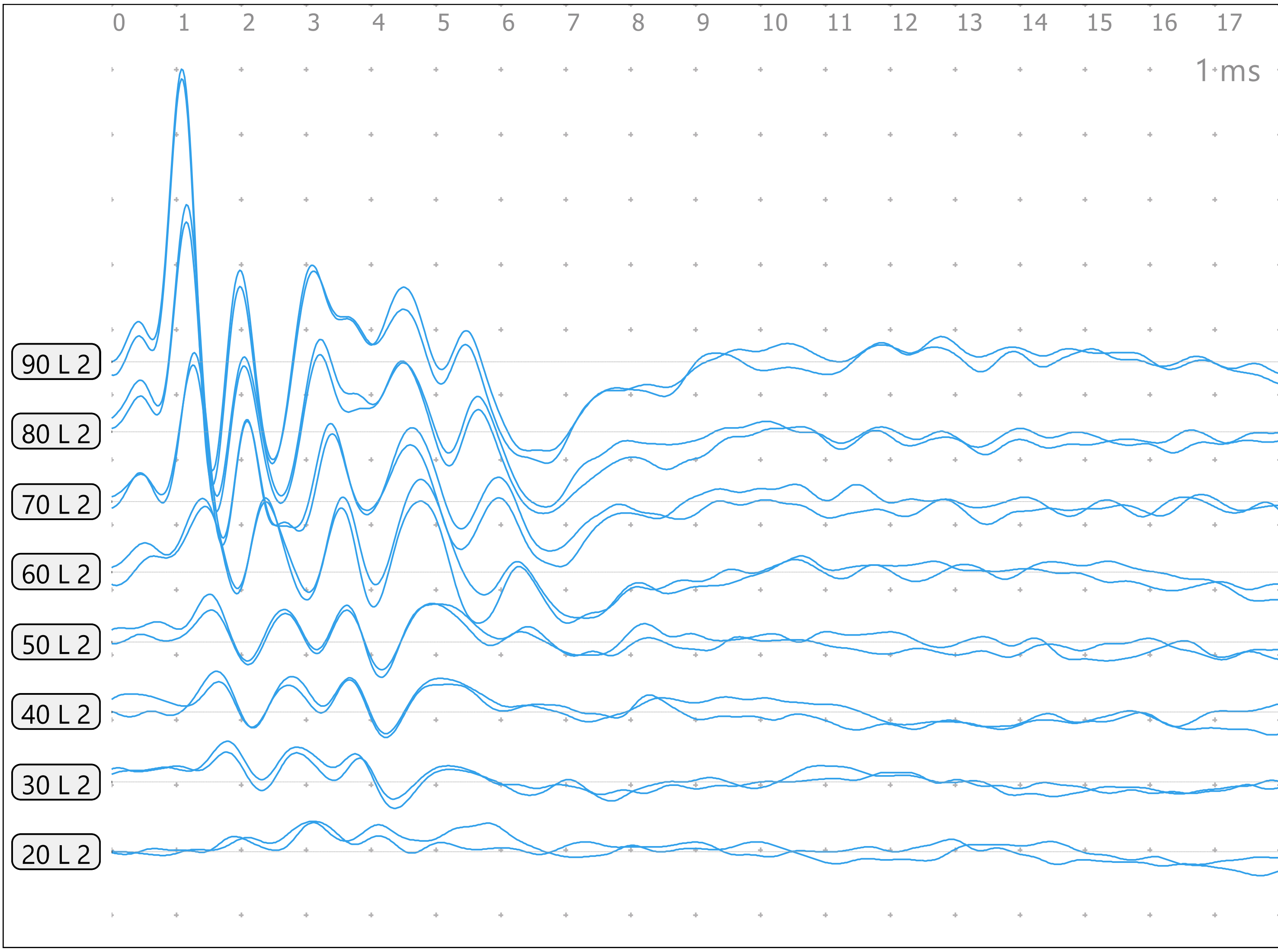

ABR: ABR 2 8000Hz 1: Cz-M1

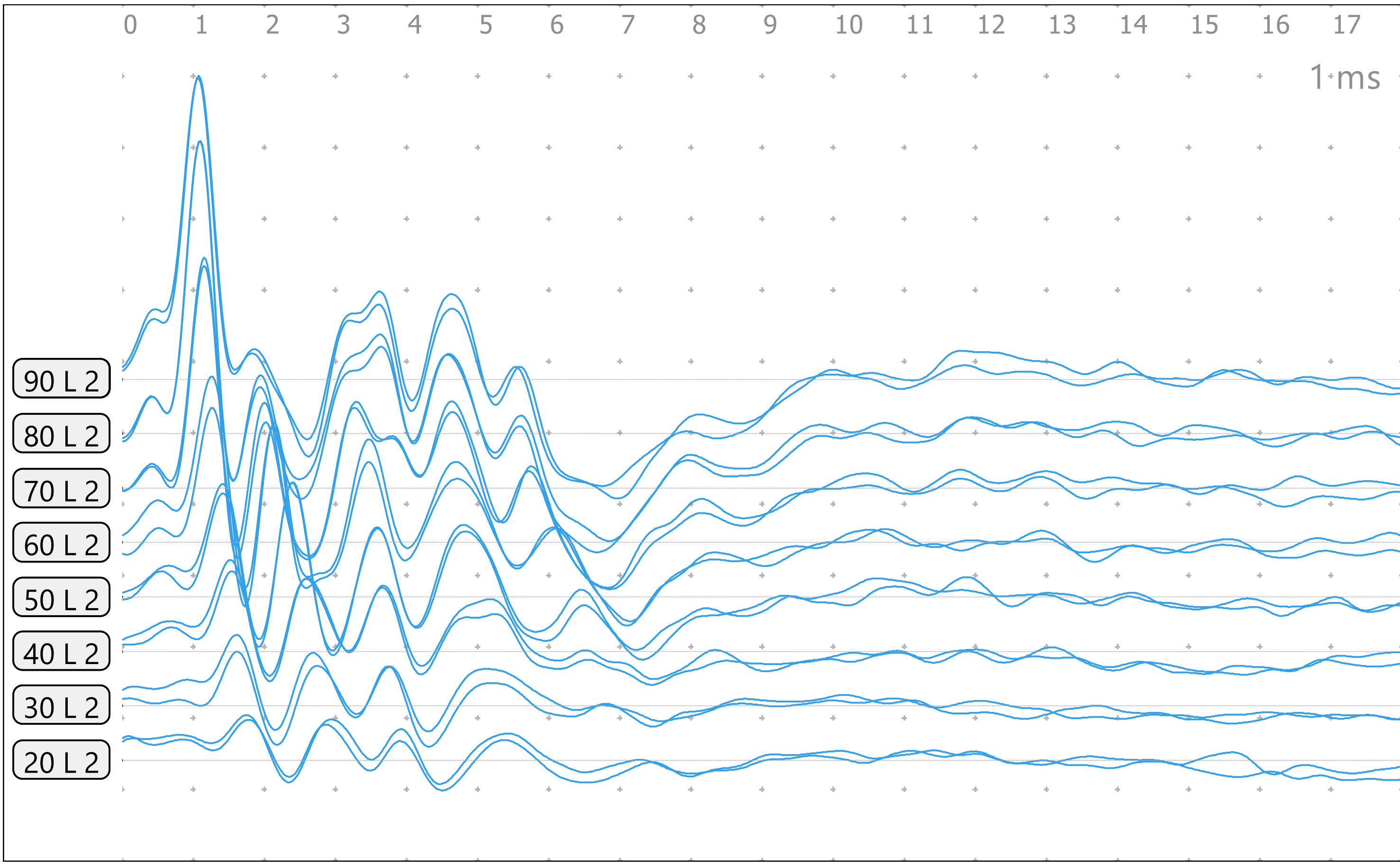

EcochG: ECochG 1: Cz-
M1

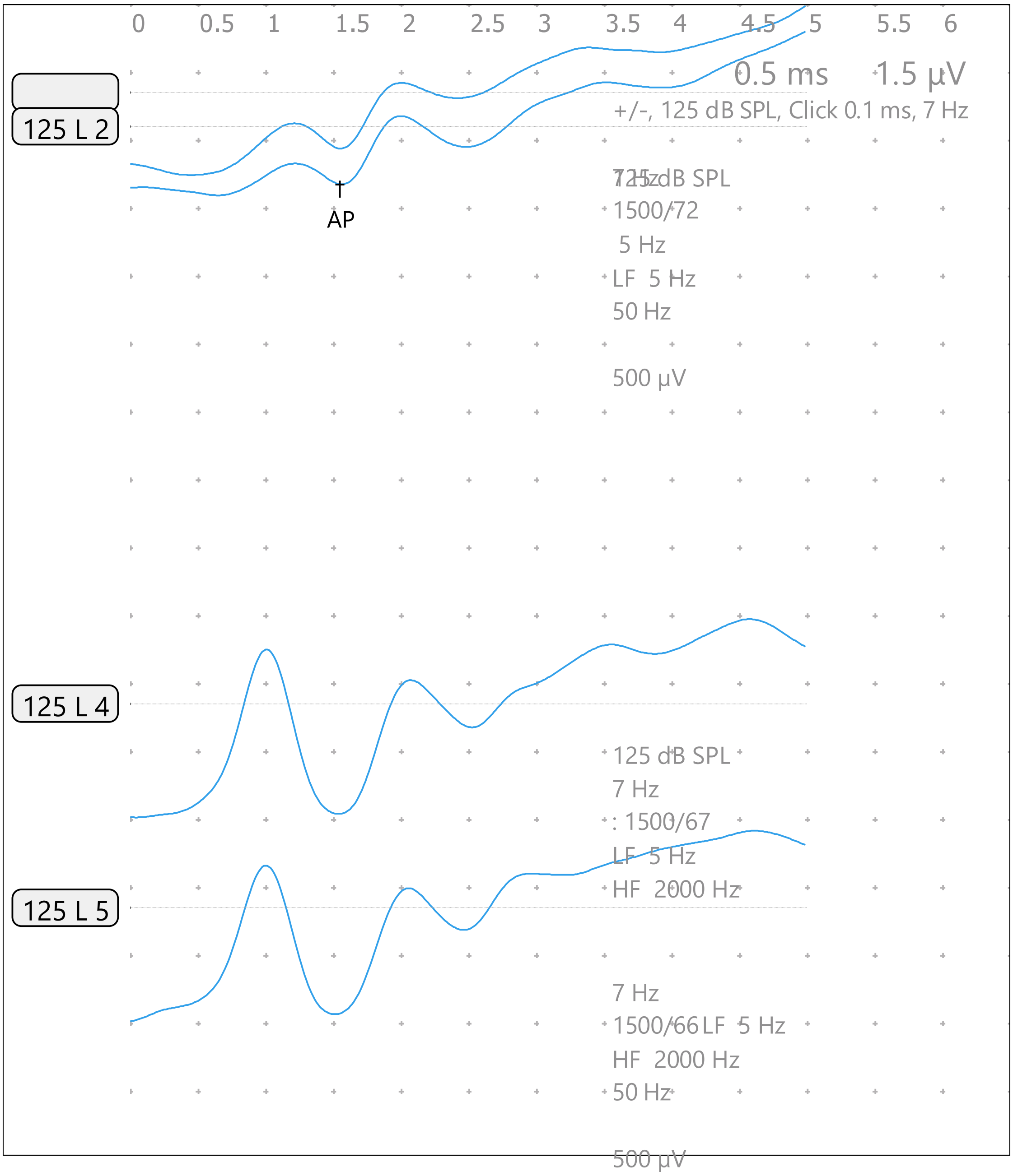

&&

N	Base (ms)	SP (ms)	AP (ms)	SP–Base (ms)	AP–Base (ms)	SP–Base (µV)	AP–Base (µV)	SP/AP amplitude ratio	SP/AP area ratio	SP area (ms·µV)	AP area (ms·µV)
125 L 2			1.55								

ECochG: ECochG 2: Cz-M2

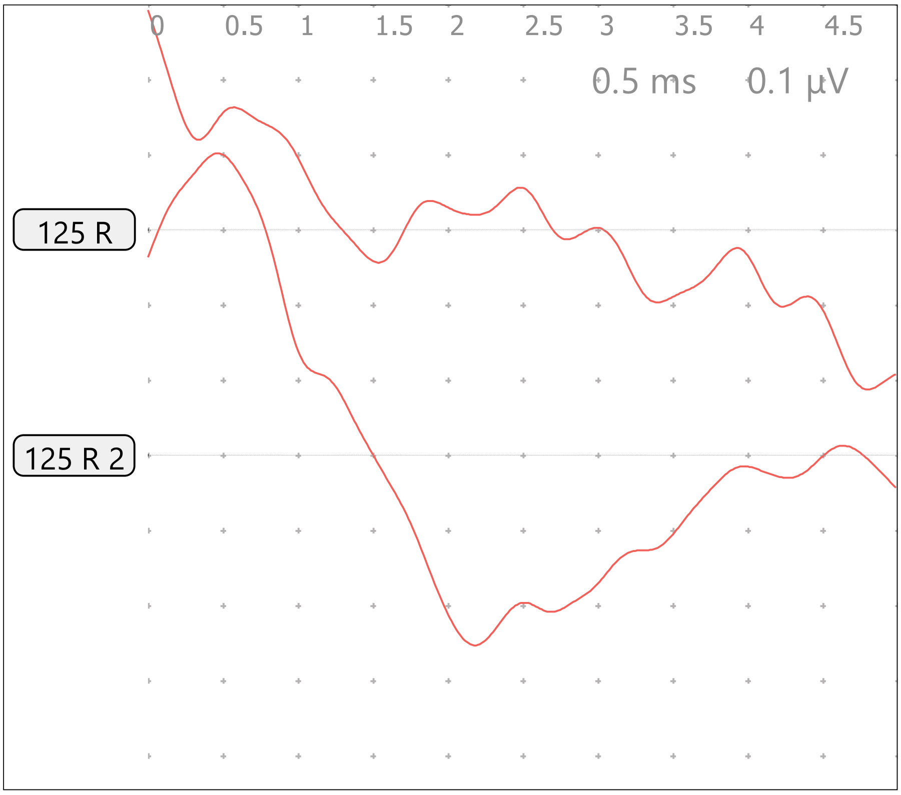
0
0.5
1
1.5
2
2.5
3
3.5
4
4.5
0.5 ms
0.1 µV
125 R
125 R 2
