# Supplementary material for: Exposure to high-altitude hypobaric hypoxic environment induces low-frequency hearing loss in C57BL/6J mice: Mediated by slowing down the postsynaptic electrical signal transmission speed in the cochlear-inferior colliculus auditory signaling pathway
Source: PLoS One. 2026 Mar 11;21(3):e0342321. doi: 10.1371/journal.pone.0342321 (PMC12978441; doi:10.1371/journal.pone.0342321)
Supplement: Attachment — Submitted filename: Response to Reviewers.docx [file pone.0342321.s003.docx]

**Response to Reviewers**

**1. Is the academic methodology rigorous? Do the data support the conclusions?**

The paper must adopt rigorous scientific research methods, and the data must fully support the conclusions; the experimental design should be standardized, including reasonable controls, repeated experiments, and an adequate sample size, with conclusions logically derived based on the presented data.

• Reviewer 1: Partially compliant

• Reviewer 2: Compliant

**2. Is the statistical analysis appropriate and rigorous?**

• Reviewer 1: Compliant

• Reviewer 2: Compliant

**3. Have the authors fully disclosed all raw data related to the research?**

In accordance with PLOS ONE’s data policy, except for very few exceptions, authors must unrestrictedly disclose all raw data relevant to the research conclusions (see the Data Availability Statement in the paper’s PDF). Data can be submitted as part of the main manuscript, supplementary materials, or uploaded to a public database. For example, in addition to providing statistical summaries, raw data points corresponding to statistics such as mean, median, and variance must be disclosed. If there are restrictions on data sharing (e.g., involving research participant privacy, use of third-party data, etc.), the nature of the restrictions must be clearly stated.

• Reviewer 1: Compliant

• Reviewer 2: Compliant

**4. Is the paper clearly expressed? Does the language conform to standard English norms?**

PLOS ONE does not provide manuscript editing or proofreading services. Submitted papers must be clear, accurate, and unambiguous. All spelling and grammatical errors should be corrected during the revision stage; if there are specific errors, please indicate them here.

• Reviewer 1: Compliant

• Reviewer 2: Compliant

**5. Detailed Reviewer Comments and Author Responses**

**Reviewer 1’s Comments**

Thank you for the opportunity to review this valuable paper. The research methods and results are presented in great detail. An area for further improvement is to supplement the discussion on the potential association with endolymphatic hydrops; appropriate elaboration will help readers understand its similarities, differences, and translational value relative to human physiological mechanisms.

**Response**: We appreciate the reviewer's recognition of the study's value and constructive suggestions. We have systematically reviewed literature related to endolymphatic hydrops and, combined with the core results of this study, specifically added discussions on its potential associations in the first paragraph of the Discussion section. We focus on sorting out the similarities, differences, and potential translational value relative to human physiological mechanisms to help readers fully understand the research significance.

**Reviewer 2’s Comments**

This study investigates the effects of simulated high-altitude hypobaric hypoxia on low-frequency hearing in mice using Auditory Brainstem Response (ABR), Electrocochleography (ECochG), and Distortion Product Otoacoustic Emissions (DPOAE) techniques. I have the following questions:

1. Only 4 mice were used per subgroup, resulting in a small sample size that may affect statistical power and the generalizability of the research results.

**Response**: We appreciate the reviewer's valuable comment. The sample size of 4 mice per subgroup was mainly constrained by the operational limitations of hypobaric hypoxia modeling: the duration of experimenters’ entry into the modeling chamber must be strictly controlled, as prolonged stay would disrupt the stability of the chamber environment and thus affect modeling effects; meanwhile, the time window for reoxygenation must be precisely regulated, making it difficult to include more experimental animals in a single experiment. We fully agree that a small sample size may have a certain impact on statistical power and the generalizability of the results. In future studies, we will expand the sample size by increasing the number of experimental batches and expanding the research team to further improve the reliability and generalizability of the research results. Thank you again for the insightful suggestion.

2. The conclusion states that "the slowing of postsynaptic electrical signal conduction may be related to changes in voltage or resistance... and may be associated with endolymphatic hydrops," but this claim lacks direct evidence-ECochG SP/AP ratio alone is insufficient to diagnose endolymphatic hydrops in mice.

**Response**: We appreciate the reviewer's pertinent comment. We fully acknowledge that this inference lacks direct evidence and that the ECochG SP/AP ratio alone is insufficient to diagnose endolymphatic hydrops in mice. Therefore, we have deleted the relevant inference stating that "the slowing of postsynaptic electrical signal conduction may be related to changes in voltage or resistance... and may be associated with endolymphatic hydrops" in the conclusion section to ensure that the conclusions are rigorous and within the scope supported by experimental evidence.

3. The control group was housed at a moderate altitude (2260 meters) rather than sea level, meaning the control group itself was exposed to a certain degree of hypobaric hypoxia, which may weaken the observed effect intensity and increase the complexity of result interpretation.

**Response**: We appreciate the reviewer's valuable comment. We agree that housing the control group at a moderate altitude (2260 meters) instead of sea level may weaken the observed effect intensity and increase the complexity of result interpretation. We have supplemented and cited research data from plain areas (sea level) for comparative analysis in the paper, and added a dedicated "Limitations" section in the Discussion to elaborate on the potential influencing factors related to this experimental design, ensuring the comprehensiveness and rigor of result interpretation.

4. The paper exceeds 10,000 words, with redundant content (e.g., the origin of ABR waveforms, ECochG physiological mechanisms) and lengthy figure legends that repeat result descriptions.

**Response**: We appreciate the reviewer's precise comment. We have systematically optimized the paper for excessive length and redundant content: first, deleted redundant elaborations such as the origin of ABR waveforms and ECochG physiological mechanisms in the first paragraph of the Discussion; second, streamlined the figure legends by removing repeated result descriptions to ensure they are concise, accurate, and focused, effectively reducing the paper length while enhancing content logic.

5. The Discussion section is overly long, descriptive in nature, and fails to fully address the study’s limitations.

**Response**: We appreciate the reviewer's reminder. We have added a limitation statement regarding "the absence of a plain-area control group" in the Discussion as required, and deleted redundant descriptions to strengthen the pertinence and depth of the Discussion.
